# Soil acidity, ecological stoichiometry and allometric scaling in grassland food webs

**DOI:** 10.1111/j.1365-2486.2009.01899.x

**Published:** 2009-11

**Authors:** CHRISTIAN MULDER, JAMES J ELSER

**Affiliations:** * Department of Ecology, RIVM9 Antonie van Leeuwenhoeklaan, Bilthoven, 3720 BA, The Netherlands; † School of Life Sciences, Arizona State UniversityTempe, AZ 85287-4501, USA

**Keywords:** allometry, biological stoichiometry, biomass-size spectra, carbon, land use change, mass–abundance relationships, nitrogen, phosphorus, soil food webs

## Abstract

The factors regulating the structure of food webs are a central focus of community and ecosystem ecology, as trophic interactions among species have important impacts on nutrient storage and cycling in many ecosystems. For soil invertebrates in grassland ecosystems in the Netherlands, the site-specific slopes of the faunal biomass to organism body mass relationships reflected basic biochemical and biogeochemical processes associated with soil acidity and soil C : N : P stoichiometry. That is, the higher the phosphorus availability in the soil, the higher, on average, the slope of the faunal biomass size spectrum (i.e., the higher the biomass of large-bodied invertebrates relative to the biomass of small invertebrates). While other factors may also be involved, these results are consistent with the growth rate hypothesis from biological stoichiometry that relates phosphorus demands to ribosomal RNA and protein production. Thus our data represent the first time that ecosystem phosphorus availability has been associated with allometry in soil food webs (supporting information available online). Our results have broad implications, as soil invertebrates of different size have different effects on soil processes.

## Introduction

Biological stoichiometry (Elser *et al.*, 1996, 2000a, b; Sterner & Elser, 2002) is the study of the balance of energy and multiple chemical elements in living systems. A central idea of biological stoichiometry is the growth rate hypothesis (GRH), which proposes that variation in C : N : P ratios among organisms reflects differential allocation to P-rich ribosomal RNA in support of rapid growth rate. Thus, fast-growing taxa have low C : P and N : P biomass ratios, making them more susceptible to P-based stoichiometric food quality constraints (Elser *et al.*, 2000b; Sterner & Elser, 2002;). As a result, ecosystem conditions that produce organic matter with high C : P and N : P ratios are likely to result in inefficient trophic transfer, reduced biomass of upper trophic levels, and potential deterministic extinction of P-rich herbivores (Perez-Moreno & Read, 2001; Sterner & Elser, 2002;). Many of these ideas have been extensively explored in aquatic systems (Blanco *et al.*, 1998; Sterner & Schulz, 1998; Gamble *et al.*, 2006; Eyto & Irvine, 2007;).

Body size relations are important for understanding ecological processes (Peters, 1983; Calder, 1984; Mulder, 2006; Damuth, 2007;), but ecological effects of C : P and N : P stoichiometric imbalance have not been widely considered for the differently sized soil invertebrates that make up belowground food webs. In aquatic habitats, in fact, allometric associations of nutrient content with abundance and biomass have already been established (e.g., Sheldon *et al.*, 1972; Blanco *et al.*, 1998;). In contrast, in terrestrial ecosystems the connections between nutrient stoichiometry and size scaling are only now beginning to be considered (Schade *et al.*, 2003; Kerkhoff *et al.*, 2005; Meehan, 2006; Enquist *et al.*, 2007; Martinson *et al.*, 2008; Reuman *et al.*, 2009;). Furthermore, the size spectra of different clades in terrestrial ecosystems have traditionally been studied separately, often from an entomological perspective (Siemann *et al.*, 1999), obscuring community-wide allometric patterns. Here we investigate if the distribution of biomass across body-size classes of all occurring soil invertebrates (henceforth, the faunal biomass spectrum) and their microbial resources are associated with nutrient concentrations and soil C : N : P ratios. In P-deficient sites, bacteria are expected to grow quite slowly and thus to have reduced P content due to lower RNA concentrations (Elser *et al.*, 2000a, 2003; Gillooly *et al.*, 2005), potentially imposing stoichiometric food quality constraints and impairing the development of higher trophic levels. The numerical abundance of bacteria reflects abiotic conditions and is positively correlated with soil pH (Mulder *et al.*, 2005a,b;). To evaluate the possible impacts of soil conditions on the structure of soil food webs we examined the relationships between soil pH and C : N : P ratios (in mass units) and the regression slopes of both the faunal biomass spectra (which plot log biomass by body–mass categories as a function of log body mass) and the mass–abundance planes (which plot log numerical abundance as a function of log body mass) for a large number of grassland soils encompassing broad ecological gradients in the Netherlands.

## Materials and methods

### Sites and organisms

**Fig. 1:**
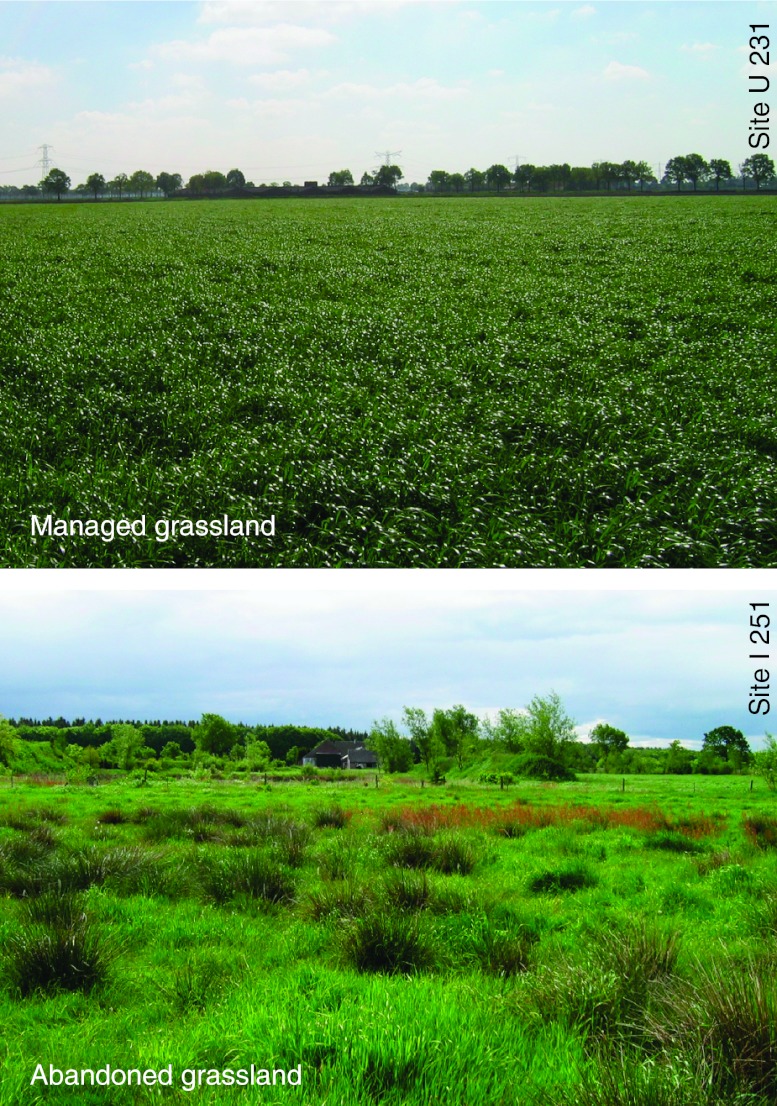
The development of mature grasslands is a primary concern of many restoration efforts in the Netherlands. We investigated both managed grasslands (average soil pH 5.3 and C : N : P ratio 41.1 : 2.8 : 1 in mass units) as abandoned grasslands (average pH 4.5 and C : N : P ratio 98 : 5.2 : 1 in mass units). *Holcus lanatus, Lolium perenne*, and *Dactylis glomerata* characterise the studied grasslands together with *Alopecurus geniculatus* and *Poa trivialis*.

One recent study showed that grasslands under organic management might exhibit a departure from power law behaviour due to organic fertilizer inputs (Reuman *et al.*, 2008). To avoid such effects, we selected, from all the locations monitored by the RIVM (Bilthoven, the Netherlands), 12 of the organic grasslands under low-intensive, bio-dynamic management and 10 ex-organic farms that were abandoned for at least a decade (Table S1). In contrast to soil acidity and P, which typically decrease following land abandonment (Fig. 1), soil nutrient ratios are expected to increase after abandonment (Knops & Tilman, 2000). Environmental gradients were chosen to obtain a certain overlap in all the investigated abiotic correlates (Table 1).

**Table 1:** Soil C : N : P stoichiometry and abiotic properties

Main abiotic properties	Abandoned	Managed
Carbon (g kg soil dry mass^−1^)	54.0±23.7	37.9±17.1
Nitrogen (g kg soil dry mass^−1^)	2.9±1.0	2.5±0.7
Phosphorus (g kg soil dry mass^−1^)	0.6±0.3	0.9±0.4
Carbon-to-phosphorus ratio	98.0±58.4	41.1±8.8
Carbon-to-nitrogen ratio	18.5±4.7	14.7±2.3
Nitrogen-to-phosphorus ratio	5.2±2.2	2.8±0.7
Soil organic matter (%)	9.3±4.1	6.5±2.9
Soil acidity (pH in KCl)	4.5±0.3	5.3±0.4

Means±SE for 10 abandoned grasslands (mature meadows) and 12 biomanaged grasslands under organic regime (complete data online in Table S1).

For this study, upper horizons (top 10 cm) of the 22 Pleistocene sandy soils selected across the Netherlands were randomly sampled between 2002 and 2005. Horizons have similar bulk density (1.20*ρ*_b_±0.29 SD) and organic matter content (7.78%±3.69 SD). Soil acidity of oven-dried soil samples was measured in 1 m potassium chloride solution (Table S1). Soils were analysed, at least in triplicate, after preliminary wet digestion. N_tot_ and P_tot_ (henceforth N and P) were determined by colorimetric analysis. Soil P content was also measured as phosphate (P_w_) by using aqueous extraction at water-to-soil ratio of 60 : 1 by volume, after 22 h of pre-equilibration with soil with water and 1 h of gentle shaking before filtration (Sissingh, 1971). Average values of C (dry combustion) were obtained by conversion of soil organic matter (SOM) using SOM=C_org_× 1.724 (the traditional Van Bemmelen conversion factor).

The total abundance of bacterial cells was determined in duplicate by fluorescent staining [5-(4, 6-dichlorotriazin-2-yl) aminofluorescein] and combined microscopy and automatic image analysis. For the conversion of bacterial cell volume (μm^3^) to dry biomass (μg) we used the biovolume-to-carbon factor of Van Veen & Paul (1979), assuming a bacterial carbon content of 50% (Herbert, 1976; Mulder *et al.*, 2005a;Table 2). Hyphae were viewed with fluorescent staining and direct microscopy at × 2500 and the total hyphal length was estimated by the line intercept method. The mycelium was inferred from the length of the counted branches, assuming a hyphal diameter of 2.5 μm (Table S2). All fungal measurements used direct microscopy and palynological treatments (Mulder *et al.*, 2005b).

**Table 2:** Faunal web structure, soil microbiology and biodiversity distribution

Main biotic properties	Abandoned	Managed
Total number of potential trophic links (linkages between genera)	710±159	706±225
Biodiversity of nematodes (Nematoda)	28±3	25±5
Biodiversity of soil mites (Acarina)	16±3	16±5
Biodiversity of springtails (Insecta)	6±3	10±3
Biodiversity of enchytraeids (Oligochaeta)	6±1	5±1
Fungal-to-bacterial ratio (μg C μg^−1^ C)	0.15±0.08	0.26±0.31

Means±SE for 10 abandoned grasslands (mature meadows) and 12 biomanaged grasslands under organic regime (complete data online in Table S2).

The soil invertebrates (Table S2) were measured as follows. For worm-like animals (nematodes and enchytraeids), the body length and width of at least 150 nematodes were measured to the nearest 5 μm with an eyepiece micrometer; all enchytraeids were measured individually, including juveniles and resting stages. The soil nematode samples were collected randomly across the investigated site. In each of the 22 locations, a composite sample was obtained by mixing 320 soil cores (diameter 2.3 cm × 10 cm) in a plastic container, and approximately 500 g of wet soil was collected in glass jars. Nematodes were extracted, within 1 week, from 100 g of wet soil using the funnel elutriation complemented with sieving and cottonwood extraction after crawling through a cotton filter over a 2-day period. Two clean suspensions in 10 mL water were screened with a stereoscope to count the individuals; the total of nematodes was estimated by counting 10% of the extracted animals twice. Permanent mounts in 4% formaldehyde were made on mass slides. Nematodes were identified by light microscopy at × 400–600 (Mulder *et al.*, 2003, 2005b). Microarthropods were extracted from the soil by placing six discs of the soil sample in a Tullgren funnel (Siepel & Van de Bund, 1988; Römbke *et al.*, 2006;). The temperature in the upper part of the funnel was set at 30 °C and kept at 5 °C in the lower part. The organisms moved downwards to escape the heat, dropped through a funnel and were collected into a bottle containing 70% ethanol. The total extraction time was 1 week. For each sample, 70 individuals were counted and identified at × 200–1000 via a gel-based subsampling method (Jagers op Akkerhuis *et al.*, 2008). Extracted arthropods were divided in body-size classes to estimate the corresponding individual body mass (*M* in microgram of dry weight). All values were averaged over the life-stages of all the counted individuals *N* (Mulder *et al.*, 2005a,b;). These body-mass averages at genus level offer the best available combination of high environmental information and low noise (Mulder *et al.*, 2006, 2008).

### Allometric scaling

Descriptors derived from allometric patterns require a brief explanation. If the species or genera of a local community are plotted as points in a plane with abscissa (horizontal axis) log(*M*), and with ordinate log(*N*), then the points have been found to fall approximately along a straight line with negative slope. This mass–abundance slope is the coefficient *a* in the linear model log(*N*)=*a*× log(*M*)+*b* fitted to data from a single soil community food web. The data can comprise organisms belonging to different kingdoms or other taxonomic groups. We computed the mass–abundance slopes for all soil invertebrates (A, from Animalia), and added two further aggregated points, one for Fungi (F, resulting in a scatter of all A+F points) and one for Eubacteria (E, resulting in a A+E+F scatter of all recovered soil eukaryotes and bacterial cells). Because the biomass of a taxon is its numerical abundance times its body mass, *B*=*NM*, and because increasing *M* is associated with increasing trophic height (Mulder *et al.*, 2005a, 2008; Reuman *et al.*, 2008), the mass–abundance slopes for A, A+F, and A+E+F are supposed to indicate how (faunal) biomass and population density change for (faunal) taxa of increasing trophic height. Mass–abundance relationships reveal how the size structure of a community food web interacts with its trophic structure and biomass distribution (White *et al.*, 2007). If the resulting mass–abundance slope is exactly −1, then the trend is for all taxa to have equal biomass. All identified genera (Table 2) fell into 22 trophic guilds. Trophic links among guilds (from resource to consumer) were inferred from published literature (Table S2). It was assumed that every taxon in a resource guild was trophically linked to every taxon in a consumer guild.

Merging the classic allometric formula log(*N*)=*a*× log(*M*)+*b* with log(*B*)=log(*M*)+log(*N*), we obtain log(*B*)=log(*M*)+*a*× log(*M*)+*b*=(1+*a*) × log(*M*)+*b* (e.g., Cyr *et al.*, 1997; Mulder *et al.*, 2008;) Theoretically, thus, the slope of a faunal biomass spectrum is 1 plus the slope of the relation between log(*N*) and log(*M*) for all the occurring animals; e.g., a zero slope of the biomass spectrum (constant biomass across trophic levels) corresponds to a slope of −1 in a plot of log(*N*) in bins as a function of log(*M*) (Rossberg *et al.*, 2008). To compute the faunal biomass spectrum slopes using bins of constant linear width, the range from the smallest log(*M*) to the largest log(*M*) occurring in any of the grasslands was divided into 10 equal bins. For each bin, the log of the total faunal biomass was computed. All values for bins containing fauna (bins with zero observations are excluded) were regressed against the log(*M*) at the centre of each bin. To test whether binning introduces a bias towards small or large organisms as suggested by White *et al.* (2008), we compared (binned) biomass spectrum slopes with three (unbinned) mass–abundance slopes. Computations used sas version 9.1.3, pc-ord version 4.20 and the excel Visual Basic optimization toolbox. We set 1% significance to detect violations.

## Results and discussion

There were significant differences in the concentrations of soil macronutrients. The C and N concentrations were closely correlated (Table S1) and highest, on average, in the abandoned grasslands (i.e., not fertilized for at least one decade) and lowest, on average, in the managed grasslands (Table 1). Total soil P contents, on the contrary, were highest in the managed grasslands, due to inputs of organic fertilizers, and lowest in the abandoned grasslands (939±400 vs. 627±277 SD mg P kg^−1^). Coefficients of variation (100 × SD/mean) of total soil P were 43% and 44% for managed and abandoned grasslands, respectively. Soil P content was uncorrelated with soil pH, in contrast to the phosphate content after water extraction [Pearson's correlation coefficients 0.024 for P and pH (*P*>0.05) and 0.53 for P_w_ and pH (*P*=0.0115), respectively]. Phosphate content after water extraction seemed to reflect the maximal possible concentration of phosphorus in the thin layer of water around soil particles (35.3±9.1 vs. 19.6±24.6 SD mg L^−1^ in managed and abandoned grasslands, respectively). Biologically available P is thought to increase within a pH range of 5–6 (Chapin & Eviner, 2003; Sims & Pierzynski, 2005; Cleveland & Liptzin, 2007;), as in our managed grasslands where fertilization and liming occurred regularly. In contrast, P becomes curtailed under lower soil pH (Bohn *et al.*, 1985), as in our abandoned grasslands.

**Fig. 2:**
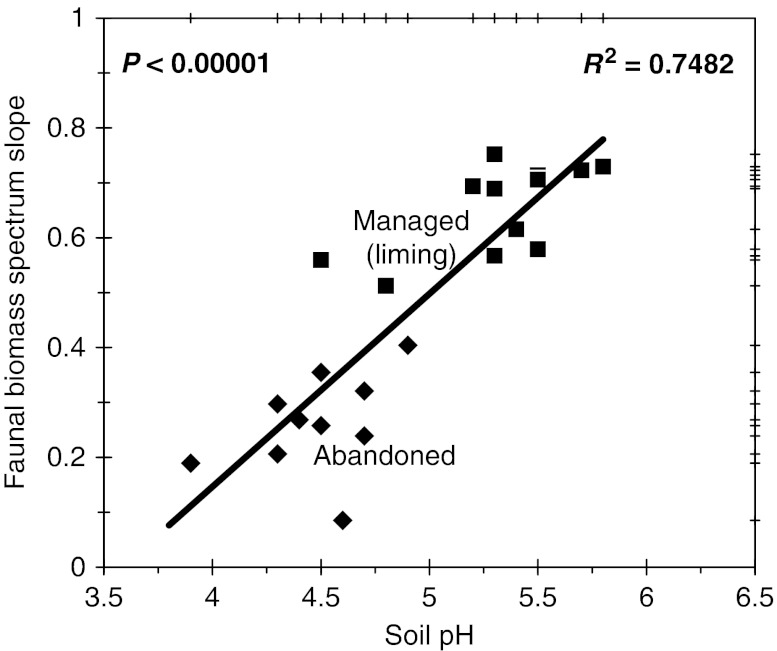
The slope of the biomass spectrum of soil invertebrates as predicted by soil acidity (pH in KCl). We combined the body masses of all individuals whose size fell within an equal interval of body size and plotted, separately for each grassland, the log summed biomass of all animal taxa within log(*M*) bins against bin centres. In this way, the slopes of the linear regression of log summed biomass become a function of the bin centres on a log(*M*) scale. Pearson's correlation coefficients show a significant correlation only between the soil pH and the logarithm of C : P (*P*=0.0010). Managed grasslands are squares and abandoned grasslands are diamonds.

Soil acidity of abandoned grasslands was almost seven times that of managed grasslands (pH averaged 4.48±0.28 and 5.32±0.36 SD, respectively). Soil pH contributes strongly to the explanation of the allometric variation within a pH range of 3.8–6.0 (Fig. 2 and Table S1). We tested the resulting correlation between soil pH and the biomass spectrum slope by adding 10 locations belonging to a completely different ecosystem, namely dry heathlands with very low soil pH (3.15±0.23 SD), to extend the investigated pH range down to 2.9. We obtained for this recalculated linear regression a slope of 0.375±0.025 SE, which is statistically indistinguishable from the original slope of 0.351±0.046 SE with a 99% CI.

Managed grasslands supported invertebrate communities in which biomass increased with the central log(*M*) values of bins in biomass spectra. Steep faunal biomass slopes were associated here with high population densities of enchytraeids that fall at the upper end of the range of body sizes (Table S2). In contrast, abandoned grasslands supported invertebrate communities in which biomass increased less with increasing central log(*M*) values of bins in biomass spectra. Shallow faunal biomass slopes were associated with higher population densities of nematodes that fall at the lower end of the size range, indicating that biomass within log(*M*) bins increased with average body size but increased less rapidly on average than after application of manure and lime. Similar associations of soil pH with different taxa and communities have been recorded recently (Mulder *et al.*, 2003, 2005b; Fierer & Jackson, 2006).

**Fig. 3:**
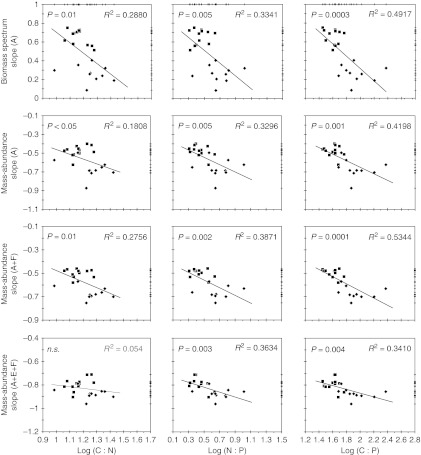
Different allometric scalings and soil nutrient ratios. Arranged according to a decreasing coefficient of variation in our sites (slopes rescaled in degrees, not shown), from top to bottom: faunal biomass spectrum slope (A=Animalia), faunal mass – abundance slope (A=Animalia), eukaryotes' mass – abundance slope (A+F=Animalia and Fungi), and mass – abundance slope of the complete community food web (A+E+F=Animalia, Eubacteria, and Fungi). The logarithm of the C : P ratio equals the sum of the log C : N and log N : P ratios and is the best sole predictor for allometric scaling after pH. Symbols as in Fig. 2.

Plotting biomass spectrum slopes and mass–abundance slopes against the logarithms of the soil nutrient ratios revealed strong relationships between nutrient ratios and faunal biomass size spectra (Fig. 3). The interpretation of the biomass spectrum slope is intuitive: negative trends indicate that faunal biomass under nutrient deficiency declined with increased body size. Biomass spectrum slopes were most significantly correlated with the logarithm of the soil C : P ratio (Pearson's correlation coefficient −0.7013, *P*=0.0003), but less with the logarithms of the N : P and C:N ratios (*P*=0.0049 and *P*=0.0099, respectively). As a matter of fact, in all cases of allometric scaling (*n*=22, four kinds of lumping, resulting in 88 investigated cases comprehending two ecosystem types), the logarithm of the C : P ratio was the best sole predictor (Fig. 3, right column).

For further interpretation of mass–abundance slopes, derived from unlumped and unbinned points with specific numerical abundance and body–mass average, one brief example may be useful. Let us imagine a simple food web consisting of four genera, namely one nematode, one mite, one springtail, and one enchytraeid. Let their respective dry weights be 0.1, 1, 10, and 100 μg on average (extensive empirical data in Table S2). After log-transformation, their log(*M*) will become −1, 0, 1, and 2. Seen that in a plane with abscissa log(*M*) and with ordinate log(*N*) most populations have been found to fall approximately along a straight line with negative slope, we assume for ease of computation that the population densities of these four genera are equal to 10 000, 1000, 100, and 10 individuals per square meter, respectively. After log-transformation, their log(*N*) will become 4, 3, 2, and 1. Their specific log(*B*) equals log(*M*)+log(*N*)=−1+4=0+3=1+2=2+1=3. Hence, these four populations will keep a dry biomass of 1 mg m^−2^ and, if plotted in a log(*M*)−log(*N*) plane, the resulting linear regression slope will be exactly equal to −1. Our mass–abundance slopes were always negative (−1<*a*<0), as expected from the positive biomass spectrum slope. The question which arises here is: how do slopes derived from such mass–abundance relationships reflect soil resource limitation?

The linear regressions of log(*N*) as function of log(*M*) account for nearly 50% of the variation in log(*N*) of organisms in most grasslands, but linear regressions may account for only 30% of the variation in some managed grasslands. The expected numerical abundances of ubiquitous smallest animals (nematodes) vary more than the numerical abundances of the larger animals (mites, springtails, and enchytraeids; see Table S2). Numerical abundances of smaller soil invertebrates differ up to four orders of magnitude from those of larger invertebrates. Moreover, the body–mass ranges of soil invertebrates reflect dominant feeding habits, since opposite functional types coming out of taxonomic inventories can be recognized: 55% of the microarthropods graze, browse or ingest fungal remains and 40% of nonparasitic nematodes graze on bacterial cells (Mulder *et al.*, 2005b and Table S2).

Therefore the allometric scaling strongly suggests that the microfauna (nematode populations) copes better with P-limited conditions than the mesofauna (microarthropod populations). Equivalently, the less the limitation by either nitrogen or carbon relative to phosphorus, or the greater the phosphorus limitation, the more rapidly the population density of invertebrate genera decreases as the average body mass of the invertebrate genera increases. All the negative trends along the environmental correlates of Fig. 3 indicate thus (i) that faunal biomass declined with increased body size within log(*M*) bins, and (ii) that faunal population density decreased with increased body size. Hence differences in soil P availability are reflected within our grasslands (and possibly in other biomes as well) in the body-size distribution of the biomass and density of the soil microfauna and mesofauna, since stoichiometric theory predicts that soil fauna with higher P demands would suffer a competitive disadvantage in lower P soils due to poorer stoichiometric food quality. The GRH (Elser *et al.*, 2000a, 2003) suggests that high P content and low C : P ratio in biomass reflect increased allocation to P-rich ribosomal RNA, which in turn enables increased protein synthesis, increased growth rates of individuals, and increased population growth rates. Empirical investigations support the GRH in varied contexts (e.g., Elser *et al.*, 2000a, 2003). The GRH provides a natural context for interpretation of at least some of our observations. Agricultural soils with higher P content, as a result of edaphic conditions, land use and history (in our case, increased P availability due to fertilization and liming effects on pH), have a twofold lower average soil C : P ratio than abandoned grasslands (Table 1).

As recently stated by Urabe & Waki (2009, p. 529), ‘herbivores tend to increase the ingestion rate when food with low nutrient content relative to C is supplied’. Many larger-bodied soil herbivores are further known for a certain combination of intrinsic and microbial sources of cellulolysis in their diet (e.g., Douglas, 2009, p. 42), and most growth and reproduction rates have been observed to decline as a function of body mass (Hendriks & Mulder, 2008; Beardall *et al.*, 2009;). We suggest that increase in P availability relaxes stoichiometric food quality constraints on small-bodied primary consumers (here, microbivores), which increases trophic transfer efficiency, and supports larger-bodied consumers (including herbivores) also at higher trophic levels. That is, soils richer in the key nutrient P have organic matter that is of higher quality for invertebrates, allowing the maintenance of food webs with increased abundances of larger taxa. As a result, biomass spectra in P-rich soils have steep positive slopes while relatively inefficient food webs in stoichiometrically imbalanced low-P soils support mostly small-bodied fauna and thus produce shallow (and theoretically even negative) biomass spectrum slopes. The extent to which low-P soils support the population density of the small-bodied fauna is shown by the more negative mass–abundance slopes.

## Conclusions

Alterations in soil C : N : P ratios, such as those driven by direct anthropogenic influences like nitrogen deposition, liming, and fertilizer application (Hunt & Wall, 2002; Wardle, 2002; Mulder *et al.*, 2005b, 2008; Persson *et al.*, 2008) and/or those driven by climatic changes like increased atmospheric CO_2_, global warming and torrential rainfall (Wardle *et al.*, 1998; Hunt & Wall, 2002; Voigt *et al.*, 2003, 2007; Sardans & Peñuelas, 2007; Urabe & Waki, 2009), may affect the rates at which soil biota carry out ecosystem services by affecting the faunal biomass distribution. However, these ideas require further investigation and we require more data on the C : N : P stoichiometry, threshold elemental ratios, growth rates, and RNA demands of soil fauna.

The association of the faunal biomass spectrum to soil acidity is more than a chemical reaction to a relative concentration of [H^+^]-ions since soil pH has a strong impact on the nutrient mobility, adsorption, and precipitation. Our 10 abandoned grasslands (lower soil pH) have a higher concentration of [H^+^]-ions than grasslands managed with lime, in which the cation exchange capacity increases. Furthermore, our data are correlative in nature. Thus, the hypothesis that stoichiometric imbalance imposed by P-limitation alters soil biomass spectra is in need of experimental test. Such a test would involve quantifying biomass spectra in experimental plots subjected to different levels of nutrient enrichment. Such experiments seem particularly timely given the recent indication that P-limitation of primary production may be more widespread in terrestrial ecosystems than previously appreciated (Elser *et al.*, 2007).

In summary, faunal biomass spectrum slopes were strongly associated with liming practice, soil nutrient conditions (as reflected by phosphate), and organic fertilizers (as reflected by the soil C : P ratio). Previous studies on faunal biomass size spectra in aquatic ecosystems reported lower slopes in systems subjected to heavy fishing and hypothesized that fishing decreased the slope of a biomass spectrum by preferentially removing larger organisms (Pope *et al.*, 1988; Jennings, 2005;), a ‘top down’ explanation. Our data showing that soil P availability is associated with increasingly positive slopes of the faunal biomass size spectrum in soil systems represent the first observation from soil food webs for a ‘bottom up’ effect on soil food-web size structure. The GRH, with or without increased trophic transfer efficiencies, provides a natural interpretation of why (artificially) P-enriched systems have food webs in which biomass increases with consumer body size while biomass declines with body size in P-limited habitats. These insights potentially connect biogeochemistry with the allometric relationships between biomass, abundance, and body size.

## Supporting Information

**Table S1.** Soil and faunal properties of the investigated grasslands. A cluster dendrogram of the environmental correlates is given in the second worksheet.

**Table S2.** Complete inventory of multitrophic interactions of soil food webs. All links consistent with literature-derived guilds are included. One summary listing all papers from which guilds were derived is given in the first worksheet.

## References

[b1] Beardall J, Allen D, Bragg J (2009). Allometry and stoichiometry of unicellular, colonial and multicellular phytoplankton. New Phytologist.

[b2] Blanco JM, Quiñones RA, Guerrero F, Rodríguez J (1998). The use of biomass spectra and allometric relations to estimate respiration of planktonic communities. Journal of Plankton Research.

[b3] Bohn H, McNeal B, O'Connor G (1985). Soil Chemistry.

[b4] Calder WA (1984). Size, Function, and Life History.

[b5] Chapin FS, Eviner VT (2003). Biogeochemistry of terrestrial net primary production. Treatise on Geochemistry.

[b6] Cleveland CC, Liptzin D (2007). C : N : P stoichiometry in soil: is there a “Redfield ratio” for the microbial biomass?. Biogeochemistry.

[b7] Cyr H, Downing JA, Peters RH (1997). Density-body size relationships in local aquatic communities. Oikos.

[b8] Damuth J (2007). A macroevolutionary explanation for energy equivalence in the scaling of body size and population density. American Naturalist.

[b9] Douglas AE (2009). The microbial dimension in insect nutritional ecology. Functional Ecology.

[b10] Elser JJ, Acharya K, Kyle M (2003). Growth rate – stoichiometry couplings in diverse biota. Ecology Letters.

[b11] Elser JJ, Bracken MES, Cleland EE (2007). Global analysis of nitrogen and phosphorus limitation of primary producers in freshwater, marine and terrestrial ecosystems. Ecology Letters.

[b12] Elser JJ, Dobberfuhl D, MacKay NA, Schampel J (1996). Organism size, life history, and N : P stoichiometry: towards a unified view of cellular and ecosystem processes. BioScience.

[b13] Elser JJ, Fagan WF, Denno RF (2000a). Nutritional constraints in terrestrial and freshwater food webs. Nature.

[b14] Elser JJ, Sterner RW, Gorokhova E (2000b). Biological stoichiometry from genes to ecosystems. Ecology Letters.

[b15] Enquist BJ, Kerkhoff AJ, Huxman TE, Economo EP (2007). Adaptive differences in plant physiology and ecosystem paradoxes: insights from metabolic scaling theory. Global Change Biology.

[b16] Eyto E, Irvine K (2007). Assessing the status of shallow lakes using an additive model of biomass size spectra. Aquatic Conservation: Marine and Freshwater Ecosystems.

[b17] Fierer N, Jackson RB (2006). The diversity and biogeography of soil bacterial communities. Proceedings of the National Academy of Science USA.

[b18] Gamble AE, Lloyd R, Aiken J, Johannsson OE, Mills EL (2006). Using zooplankton biomass size spectra to assess ecological change in a well-studied freshwater lake ecosystem: Oneida Lake, New York. Canadian Journal of Fisheries and Aquatic Sciences.

[b19] Gillooly JF, Allen AP, Brown JH (2005). The metabolic basis of whole-organism RNA and phosphorus content. Proceedings of the National Academy of Science USA.

[b20] Hendriks AJ, Mulder C (2008). Scaling of offspring number and mass to plant and animal size: model and meta-analysis. Oecologia.

[b21] Herbert D, Dean ACR, Ellwood DC, Evans CGT, Melling J (1976). Stoichiometric aspects of microbial growth. Continuous Culture 6: Application and New Fields.

[b22] Hunt HW, Wall DH (2002). Modelling the effects of loss of soil biodiversity on ecosystem function. Global Change Biology.

[b23] Jagers op Akkerhuis GAJM, Dimmers WJ, Van Vliet PCJ, Goedhart GFP, Martakis GFP, De Goede RGM (2008). Evaluating the use of gel-based sub-sampling for assessing responses of terrestrial micro-arthropods (Collembola and Acari) to different slurry applications and organic matter contents. Applied Soil Ecology.

[b24] Jennings S, Belgrano A, Scharler UM, Dunne J, Ulanowicz RE (2005). Size-based analyses of aquatic food webs. Aquatic Food Webs – An Ecosystem Approach.

[b25] Kerkhoff AJ, Enquist BJ, Elser JJ, Fagan WF (2005). Plant allometry, stoichiometry and the temperature-dependence of primary productivity. Global Ecology and Biogeography.

[b26] Knops JMH, Tilman D (2000). Dynamics of soil nitrogen and carbon accumulation for 61 years after agricultural abandonment. Ecology.

[b27] Martinson HM, Schneider K, Gilbert J, Hines JE, Hamba PA, Fagan WF (2008). Detritivory: stoichiometry of a neglected trophic level. Ecological Research.

[b28] Meehan TD (2006). Energy use and animal abundance in litter and soil communities. Ecology.

[b29] Mulder C (2006). Driving forces from soil invertebrates to ecosystem functioning: the allometric perspective. Naturwissenschaften.

[b30] Mulder C, Cohen JE, Setälä H, Bloem J, Breure AM (2005a). Bacterial traits, organism mass, and numerical abundance in the detrital soil food web of Dutch agricultural grasslands. Ecology Letters.

[b31] Mulder C, De Zwart D, Van Wijnen HJ, Schouten AJ, Breure AM (2003). Observational and simulated evidence of ecological shifts within the soil nematode community of agroecosystems under conventional and organic farming. Functional Ecology.

[b32] Mulder C, Den Hollander H, Schouten T, Rutgers M (2006). Allometry, biocomplexity, and web topology of hundred agro-environments in the Netherlands. Ecological Complexity.

[b33] Mulder C, Den Hollander HA, Hendriks AJ (2008). Aboveground herbivory shapes the biomass distribution and flux of soil invertebrates. Public Library of Science ONE.

[b34] Mulder C, Van Wijnen HJ, Van Wezel AP (2005b). Numerical abundance and biodiversity of below-ground taxocenes along a pH gradient across the Netherlands. Journal of Biogeography.

[b35] Perez-Moreno J, Read DJ (2001). Nutrient transfer from soil nematodes to plants: a direct pathway provided by the mycorrhizal mycelial network. Plant, Cell and Environment.

[b36] Persson J, Vrede T, Holmgren S (2008). Responses in zooplankton populations to food quality and quantity changes after whole lake nutrient enrichment of an oligotrophic sub-alpine reservoir. Aquatic Sciences.

[b37] Peters RH (1983). The Ecological Implications of Body Size.

[b38] Pope JG, Stokes TK, Murawski SA, Idoine SI, Wolff W, Soeder C, Drepper F (1988). A comparison of fish size composition in the North Sea and on Georges Bank. Ecodynamics.

[b39] Reuman DC, Cohen JE, Mulder C (2009). Human and environmental factors influence soil faunal abundance-mass allometry and structure. Advances in Ecological Research.

[b40] Reuman DC, Mulder C, Raffaelli D, Cohen JE (2008). Three allometric relations of population density to body mass: theoretical integration and empirical tests in 149 food webs. Ecology Letters.

[b41] Römbke J, Sousa J-P, Schouten T, Riepert F (2006). Monitoring of soil organisms: a set of standardized field methods proposed by ISO. European Journal of Soil Biology.

[b42] Rossberg AG, Ishii R, Amemiya T, Itoh K (2008). The top–down mechanism for body-mass abundance scaling. Ecology.

[b43] Sardans J, Peñuelas J (2007). Drought changes phosphorus and potassium accumulation patterns in an evergreen Mediterranean forest. Functional Ecology.

[b44] Schade JD, Kyle M, Hobbie SE, Fagan WF, Elser JJ (2003). Stoichiometric tracking of soil nutrients by a desert insect herbivore. Ecology Letters.

[b45] Sheldon RW, Prakash A, Sutcliffe WHJ (1972). The size distribution of particles in the ocean. Limnology and Oceanography.

[b46] Siemann E, Tilman D, Haarstad J (1999). Abundance, diversity and body size: patterns from a grassland arthropod community. Journal of Animal Ecology.

[b47] Siepel H, Van de Bund CF (1988). The influence of management practices on the microarthropod community of grassland. Pedobiologia.

[b48] Sims JT, Pierzynski GM, Tabatabai MA, Sparks DL (2005). Chemistry of phosphorus in soils. Chemical Processes in Soils.

[b49] Sissingh HA (1971). Analytical technique of the P_w_ method, used for the assessment of the phosphate status of arable soils in the Netherlands. Plant and Soil.

[b50] Sterner RW, Elser JJ (2002). Ecological Stoichiometry: The Biology of Elements from Molecules to the Biosphere.

[b51] Sterner RW, Schulz KL (1998). Zooplankton nutrition: recent progress and a reality check. Aquatic Ecology.

[b52] Urabe J, Waki N (2009). Mitigation of adverse effects of rising CO_2_ on a planktonic herbivore by mixed algal diets. Global Change Biology.

[b53] Van Veen JA, Paul EA (1979). Conversion of biovolume measurements of soil organisms, grown under various moisture tensions, to biomass and their nutrient content. Applied and Environmental Microbiology.

[b54] Voigt W, Perner J, Davis AJ (2003). Trophic levels are differentially sensitive to climate. Ecology.

[b55] Voigt W, Perner J, Jones TH (2007). Using functional groups to investigate community response to environmental changes: two grassland case studies. Global Change Biology.

[b56] Wardle DA (2002). Communities and Ecosystems: Linking the Aboveground and Belowground Components.

[b57] Wardle DA, Verhoef HA, Clarholm M (1998). Trophic relationships in the soil microfood-web: predicting the responses to a changing global environment. Global Change Biology.

[b58] White EP, Enquist BJ, Green JL (2008). On estimating the exponent of power-law frequency distributions. Ecology.

[b59] White EP, Ernest SKM, Kerkhoff AJ, Enquist BJ (2007). Relationships between body size and abundance in ecology. Trends in Ecology and Evolution.

