# Supplementary material for: Soil acidity, ecological stoichiometry and allometric scaling in grassland food webs
Source: Glob Chang Biol. 2009 Nov;15(11):2730–8. doi: 10.1111/j.1365-2486.2009.01899.x (PMC3597259; doi:10.1111/j.1365-2486.2009.01899.x)

	ID code	Faunal biomass spectrum slope	Mass-abundance slope (Animalia)	Mass-abundance slope (Animalia+Fungi)	Mass-abundance slope (Animalia+Eubacteria+Fungi)	pH in KCl	CtoN-ratio	CtoP-ratio	NtoP-ratio	logC-logN	logC-logP	logN-logP	C (mg/kg)	N (mg/kg)	P (mg/kg)	P-H_2O (mg/l)	SOM (% dry soil)	Total faunal density (logN)	Total faunal biomass (logB)	Total bacterial C ($logC_{mic}$)	Sampling period	Habitat description
243	A	0.2973	-0.5734	-0.6075	-0.8435	4.3	9.7	62.5	6.5	0.98	1.80	0.81	33721	3494	540	7	5.8	6.6121	6.5093	1.6517	May 6, 2004	Abandoned grassland
244	B	0.3209	-0.6228	-0.6312	-0.8562	4.7	22.9	236.8	10.3	1.36	2.37	1.01	50581	2207	214	4	8.7	6.6199	6.3742	1.5295	May 13, 2004	Abandoned grassland
245	C	0.3546	-0.4953	-0.5707	-0.7892	4.5	14.5	53.1	3.7	1.16	1.72	0.56	22674	1568	427	24	3.9	6.5396	6.6107	1.0311	June 11, 2004	Abandoned grassland
246	D	0.2390	-0.6503	-0.6639	-0.8542	4.7	21.8	48.4	2.2	1.34	1.69	0.35	50000	2299	1033	86	8.6	6.7306	6.4762	0.8391	June 1, 2004	Abandoned grassland
247	E	0.2681	-0.6836	-0.6837	-0.8749	4.4	17.8	70.6	4.0	1.25	1.85	0.60	34302	1931	486	16	5.9	6.7774	6.5525	1.1114	June 15, 2004	Abandoned grassland
248	F	0.2062	-0.6833	-0.6799	-0.8868	4.3	19.6	88.8	4.5	1.29	1.95	0.66	73256	3735	825	10	12.6	6.7097	6.4584	1.1482	May 25, 2004	Abandoned grassland
249	G	0.4041	-0.7132	-0.6946	-0.8742	4.9	18.2	80.6	4.4	1.26	1.91	0.65	43605	2391	541	16	7.5	6.7916	6.5634	0.9704	June 17, 2004	Abandoned grassland
250	H	0.0851	-0.8731	-0.7538	-0.9616	4.6	16.7	74.1	4.4	1.22	1.87	0.65	81395	4871	1098	4	14	6.8687	6.1457	1.7088	April 21, 2004	Abandoned grassland
251	I	0.2577	-0.6861	-0.7002	-0.8915	4.5	17.6	104.7	6.0	1.24	2.02	0.78	51744	2945	494	25	8.9	6.7490	6.4090	1.0488	May 26, 2004	Abandoned grassland
252	J	0.1892	-0.7058	-0.7007	-0.9012	3.9	26.5	160.7	6.1	1.42	2.21	0.78	98837	3725	615	4	17	6.6185	6.2354	1.1818	May 28, 2004	Abandoned grassland
264	K	0.5126	-0.4866	-0.5327	-0.7802	4.8	19.0	39.7	2.1	1.28	1.60	0.32	68605	3614	1730	42	11.8	6.9103	7.6100	2.2050	April 12, 2005	Managed grassland
265	L	0.5671	-0.3988	-0.4809	-0.7122	5.3	16.9	42.0	2.5	1.23	1.62	0.39	38372	2268	914	32	6.6	6.6085	7.3629	1.7738	April 13, 2005	Managed grassland
266	M	0.5594	-0.4126	-0.4741	-0.7112	4.5	18.3	43.5	2.4	1.26	1.64	0.38	77907	4266	1790	18	13.4	6.7427	7.7909	2.2123	April 15, 2005	Managed grassland
222	N	0.7295	-0.4656	-0.4965	-0.7888	5.8	14.8	43.1	2.9	1.17	1.63	0.46	28488	1921	661	50	4.9	6.2432	6.2965	1.4460	May 30, 2002	Managed grassland
223	O	0.6938	-0.5126	-0.5283	-0.8623	5.2	13.5	58.3	4.3	1.13	1.77	0.64	32791	2436	562	37	5.6	6.1136	6.4525	2.2172	May 15, 2002	Managed grassland
224	P	0.7140	-0.5005	-0.4968	-0.7741	5.5	14.8	43.5	2.9	1.17	1.64	0.47	34884	2351	803	39	6	6.1354	6.3601	1.1260	June 4, 2002	Managed grassland
225	Q	0.7059	-0.4252	-0.4598	-0.7859	5.5	14.2	49.8	3.5	1.15	1.70	0.55	32558	2300	654	27	5.6	6.0422	6.5093	1.7690	May 16, 2002	Managed grassland
227	R	0.7523	-0.4597	-0.4668	-0.7673	5.3	12.0	28.4	2.4	1.08	1.45	0.37	25581	2125	902	28	4.4	6.0005	6.5767	1.6683	May 28, 2002	Managed grassland
229	S	0.7229	-0.4507	-0.4803	-0.8080	5.7	14.6	29.8	2.0	1.16	1.47	0.31	27326	1873	918	37	4.7	6.1346	6.3066	2.0362	May 22, 2002	Managed grassland
230	T	0.6155	-0.4726	-0.4776	-0.8142	5.4	11.5	31.4	2.7	1.06	1.50	0.44	25581	2228	816	37	4.4	6.0614	5.9064	2.0115	May 14, 2002	Managed grassland
231	U	0.5793	-0.6229	-0.5797	-0.9042	5.5	13.3	48.0	3.6	1.12	1.68	0.56	33140	2495	690	48	5.7	6.3605	6.1981	2.2090	May 17, 2002	Managed grassland
232	V	0.6894	-0.5201	-0.5102	-0.8143	5.3	13.2	35.6	2.7	1.12	1.55	0.43	29651	2247	834	29	5.1	6.1198	6.0229	1.9466	May 21, 2002	Managed grassland

Clustering the soil abiotics of our 22 webs

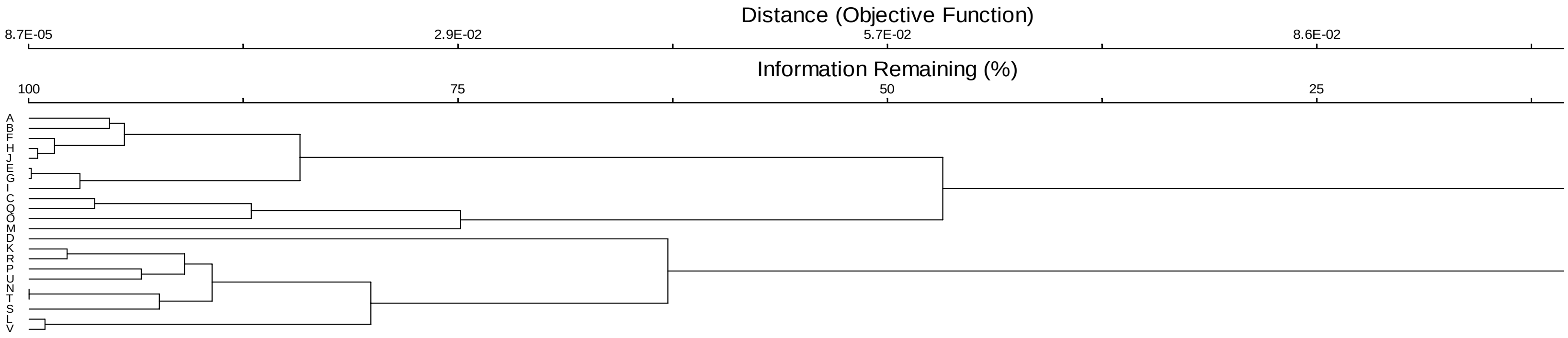

Supplement: Supplementary file 1 [file gcb0015-2730-SD1.pdf]
