# Supplementary material for: Soil acidity, ecological stoichiometry and allometric scaling in grassland food webs
Source: Glob Chang Biol. 2009 Nov;15(11):2730–8. doi: 10.1111/j.1365-2486.2009.01899.x (PMC3597259; doi:10.1111/j.1365-2486.2009.01899.x)
Supplement: Supplementary file 2 [file gcb0015-2730-SD2.pdf]

SOIL ACIDITY, ECOLOGICAL STOICHIOMETRY AND ALLOMETRIC SCALING IN GRASSLAND FOOD WEBS

Christian Mulder, James J. Elser

Correspondence should be addressed to christian.mulder@rivm.nl

Table S2 (each worksheet is one site)

Each row describes one trophic link inferred from published feeding relationships of trophic guilds.

COLUMN HEADING	DEFINITION
Resource	Most resolved taxonomic name of the animal eaten, the resource
Consumer	Most resolved taxonomic name of the eating animal, the consumer
Mres	Dry body mass in micrograms of the resource, measured directly or inferred from length and length-mass allometry
Mconsumer	Dry body mass in micrograms of the consumer, measured directly or inferred from length and length-mass allometry
Nres	Numerical abundance (individuals per m^2 in the top 0.1 m of soil) of the resource
Nconsumer	Numerical abundance (individuals per m^2 in the top 0.1 m of soil) of the consumer

Worksheet: Literature

Publications used to infer feeding relationships among the observed taxa

Agrawal, A. A., Kobayashi, C., Thaler, J. S. 1999. Influence of prey availability and induced host-plant resistance on omnivory by western flower thrips. Ecology 80:518-523

Axelsson, B., Lohm, U., Persson, T. 1984. Enchytraeids, lumbricids and soil arthropods in a northern deciduous woodland – a quantitative study. Holarctic Ecology 7:91-103

Bakonyi, G., Posta, K., Kiss, I., Fábián, M., Nagy, P., Nosek, J. N. 2002. Density-dependent regulation of arbuscular mycorrhiza by collembola. Soil Biology and Biochemistry 34:661-664

Bardgett, R. D., Chan, K. F. 1999. Experimental evidence that soil fauna enhance nutrient mineralization and plant nutrient uptake in montane grassland ecosystems. Soil Biology and Biochemistry 31:1007-1014

Bardgett, R. D., Cook, R. 1998. Functional aspects of soil animal diversity in agricultural grasslands. Applied Soil Ecology 10:263-276

Bardgett, R. D., Cook, R., Yeates, G. W., Denton, C. S. 1999. The influence of nematodes on below-ground processes in grassland ecosystems. Plant and Soil 212:23-33

Bardgett, R. D., Keiller, S., Cook, R., Gilburn, A. S. 1998. Dynamic interactions between soil animals and microorganisms in upland grassland soils amended with sheep dung: a microcosm experiment. Soil Biology and Biochemistry 30:531-539

Bardgett, R. D., Wardle, D. A., Yeates, G. W. 1998. Linking above-ground and below-ground interactions: how plant responses to foliar herbivory influence soil organisms. Soil Biology and Biochemistry 30:1867-1878

Berg, M. P., Stoffer, M., Van den Heuvel, H. H. 2002. Feeding guilds in Collembola based on digestive enzymes. Pedobiologia 48: 589-601

Chen, B. R., Wise, D. H. 1997. Responses of forest-floor fungivores to experimental food enhancement. Pedobiologia 41:316-326

Cross, E. A., Moser, J. C. 1975. A new dimorphic species of *Pyemotes* and a key to previously-described forms (Acarina: Tarsonemoidea). Annals of the Entomological Society of America 68:723-732

Cross, E. A., Moser, J. C., Rack, G. 1981. Some new forms of *Pyemotes* (Acarina: Pyemotidae) from forest insects, with remarks on polymorphism. International Journal of Acarology 7:179-196

De Ruiter, P. C., Van Veen, J. A., Moore, J. C., Brussaard, L., Hunt, H. W. 1993. Calculation of nitrogen mineralisation in soil food webs. Plant and Soil 157:263-273

Díaz, A., Okabe, K., Eckenrode, C. J., Villani, M. G., O'Connor, B. M. 2000. Biology, ecology, and management of the bulb mites of the genus *Rhizoglyphus* (Acari: Acaridae). Experimental and Applied Acarology 24:85-113

Dindal, D. L. 1990. Soil Biology Guide. John Wiley & Sons: New York, New York.

Enkegaard, A., Sardar, M. A., Brødsgaard, H. F. 1997. The predatory mite *Hypoaspis miles*: biological and demographic characteristics on two prey species, the mushroom sciarid fly, *Lycoriella solani*, and the mould mite, *Tyrophagus putrescentiae*. Entomologia Experimentalis et Applicata 82:135-146
Filser, J., Krogh, P. H. 2002. Interactions between *Enchytraeus crypticus*, collembolans, gamasid mites and barley plants: A greenhouse experiment. Natura Jutlandica Suppl. 2:32-42
Gange, A. C. 1993. Translocation of mycorrhizal fungi by earthworms during early plant succession. Soil Biology and Biochemistry 25:1021-1026
Gange, A. C. 2000. Species-specific responses of a root- and shoot-feeding insect to arbuscular mycorrhizal colonization of its host plant. New Phytologist 150:611-618
Gange, A. C. 2000. Arbuscular mycorrhizal fungi, Collembola and plant growth. Trends in Ecology and Evolution 15:369-372

Gange, A. C., Brown, V. K. 2002. Soil food web components affect plant community structure during early succession. Ecological Research 17:217-227
Gange, A. C., Stagg, P. G., Ward, L. K. 2002. Arbuscular mycorrhizal fungi affect phytophagous insect specialism. Ecology Letters 5:11-15
Gupta, M. C. 1988. Influence of carbonaceous and nitrogenous amendments on population dynamics of *Tylenchus* and *Criconemoides* in soil. Indian Journal of Nematology 18:207-211
Holterman, M., Van der Wurff, A., Van den Elsen, S., Van Megen, H., Bongers, T., Holovachov, O., Bakker, J., Helder, J. 2006. Phylum-wide analysis of SSU rDNA reveals deep phylogenetic relationships among nematodes and accelerated evolution toward crown clades. Molecular Biology and Evolution 23:1792–1800.
Holterman, M., Rybarczyk, K. , Van den Elsen, S., Van Megen, H., Mooyman, P., Peña Santiago, R., Bongers, T., Bakker, J., Helder, J. 2008. A ribosomal DNA-based framework for the detection and quantification of stress-sensitive nematode families in terrestrial habitats. Molecular Ecology Resources 8:23-34
Hunt, H. W., Coleman, D. C., Ingham, E. R., Ingham, R. E., Elliot, E. T., Moore, J. C., Rose, S. L., Reid, C. P. P., Morley C. R. 1987. The detrital food web in a shortgrass prairie. Biology and Fertility of Soils 3:57-68
Ingham, R. E., Trofymow, J. A., Ingham, E. R., Coleman, D. C. 1985. Interactions of bacteria, fungi, and their nematode grazers: Effects on nutrient cycling and plant growth. Ecological Monographs 55:119-140
Janssen, A., Pallini, A., Venzon, M., Sabelis, M. W. 1998. Behaviour and indirect interactions in food webs of plant-inhabiting arthropods. Experimental and Applied Acarology 22:497-521
Kay, F. R., Sobhy, H. M., Whitford, W. G. 1999. Soil microarthropods as indicators of exposure to environmental stress in Chihuahuan desert Rangelands. Biology and Fertility of Soils 28:121–128
King, K. L., Hutchinson, K. J., Greenslade, P. 1976. The effects of sheep numbers on associations of Collembola in sown pastures. Journal of Applied Ecology 13:731–739
Klironomos, J. N., Kendrick, B. 1995a. Relationships among microarthropods, fungi, and their environment. Plant and Soil 170:183-197
Klironomos, J. N., Kendrick, B. 1995b. Stimulative effects of arthropods on endomycorrhizas of sugar maple in the presence of decaying litter. Functional Ecology 9:528-536
Klironomos, J. N., Kendrick, W. B. 1996. Palatability of microfungi to soil arthropods in relation to the functioning of arbuscular mycorrhizae. Biology and Fertility of Soils 21:43-52
Klironomos, J. N., Bednarczuk, E. M., Neville, J. 1999. Reproductive significance of feeding on saprobic and arbuscular mycorrhizal fungi by the collembolan, *Folsomia candida*. Functional Ecology 13:756-761
Laakso, J., Setälä, H. 1999. Population- and ecosystem-level effects of predation on microbial-feeding nematodes. Oecologia 120:279-286

Laakso, J., Setälä, H., Palojärvi, A. 2000. Influence of decomposer food web structure and nitrogen availability on plant growth. Plant and Soil 225:153-165
Liiri, M., Setälä, H., Haimi, J., Pennanen, T., Fritze, H. 2002a. Relationship between soil microarthropod species diversity and plant growth does not change when the system is disturbed. Oikos 96:137-149

Liiri, M., Setälä, H., Haimi, J., Pennanen, T., Fritze, H. 2002b. Soil processes are not influenced by the functional complexity of soil decomposer food webs under disturbance. Soil Biology and Biochemistry 34:1009-1020
Magnusson, C. 1983. Abundance distribution and feeding relations of root fungal feeding nematodes in a Scotch pine forest *Pinus sylvestris*. Holarctic Ecology 6:183-193
Marshall, V. G., Reeves, R. M., Norton, R. A. 1987. Catalogue of the Oribatida (Acari) of continental United States and Canada. Memoirs of the Entomological Society of Canada 139:1-623
Moore, J. C., Walter, D. E., Hunt, H. W. 1988. Arthropod regulation of micro- and mesobiota in below-ground detrital food web. Annual Reviews of Entomology 33:419-439
Mulder, C. 2006. Driving forces from soil invertebrates to ecosystem functioning: the allometric perspective. Naturwissenschaften 93:467-479
Mulder, C., Breure, A. M., Joosten, J. H. J. 2003a, Fungal functional diversity inferred along Ellenberg's abiotic gradients: Palynological evidence from different soil microbiota. Grana 42:55-64
Mulder, C., De Zwart, D., Van Wijnen, H. J., Schouten, A. J., Breure, A. M. 2003b. Observational and simulated evidence of ecological shifts within the soil nematode community of agroecosystems under conventional and organic farming. Functional Ecology 17:516-525
Mulder, C., Cohen, J. E., Setälä, H., Bloem, J., Breure, A. M. 2005a. Bacterial traits, organism mass, and numerical abundance in the detrital soil food web of Dutch agricultural grasslands. Ecology Letters 8:80-90 (Parent Article; Supplementary Online Material free at http://www.blackwellpublishing.com/products/journals/suppmat/ELE/ELE704/ELE704sm.htm)
Mulder, C., Van Wijnen, H. J., Van Wezel, A. P. 2005b. Numerical abundance and biodiversity of below-ground taxocenes along a pH gradient across the Netherlands. Journal of Biogeography 32:1775-1790
Mulder, C., Dijkstra, J.B., Setälä, H. 2005c. Nonparasitic Nematoda provide evidence for a linear response of functionally important soil biota to increasing livestock density. Naturwissenschaften 92:314-318
Okada, H., Tsukiboshi, T., Kadota, I., 2002. Mycetophagy in *Filenchus misellus* (Andrássy, 1958) Lownsbery & Lownsbery, 1985 (Nematoda: Tylenchidae) with notes on its morphology. Nematology 4:795-801
Patt, J. M., Wainright, S. C., Hamilton, G. C., Whittinghill, D., Bosley, K., Dietrick, J., Lashomb, J. H. 2003. Assimilation of carbon and nitrogen from pollen and nectar by a predaceous larva and its effects on growth and development. Ecological Entomology 28:717-728
Perez-Moreno, J., Read, D. J. 2001. Nutrient transfer from soil nematodes to plants: a direct pathway provided by the mycorrhizal mycelial network. Plant, Cell and Environment 24:1219-1226
Petersen, H. 1981. The respiratory metabolism of Collembola species from a Danish beech wood. Oikos 37:273-286
Petersen, H. 1995a. Temporal and spatial dynamics of soil Collembola during secondary succession in Danish heathland. Acta Zoologica Fennica 196:190-194

Petersen, H. 1995b. Energy flow and trophic relations in soil communities: state of knowledge two decades after the International Biological Programme. In: Edwards, C. A., Abe, T., Striganova, B. R. (eds.) Structure and Function of Soil Communities. Kyoto University Press: Kyoto, p. 111-130
Petersen, H. 2000. Collembola populations in an organic crop rotation: population dynamics and metabolism after conversion from clover-grass ley to spring barley. Pedobiologia 44:502-515
Petersen, H. 2002. Effects of non-inverting deep tillage vs. conventional ploughing on collembolan populations in an organic wheat field. European Journal of Soil Biology 38:177-180
Petersen, H., Gjelstrup, P. 1998. Effects of the insecticide dimethoate on the behaviour of *Folsomia fimetaria* (L.) (Collembola: Isotomidae). Applied Soil Ecology 9:389-392
Petersen, H., Luxton, M. 1982. A comparative analysis of soil fauna populations and their role in decomposition process. Oikos 39:288-388
Petersen, H., Jucevica, E., Gjelstrup, P. 2004. Long-term changes in collembolan communities in grazed and non-grazed abandoned arable fields in Denmark. Pedobiologia 48:559-573

Phillipson, J., Abel, R., Steel, J., Woodell, S. R. J. 1977. Nematode numbers, biomass and respiratory metabolism in a beech woodland – Wytham Woods, Oxford. Oecologia 27:141-155
Radwan, J., Bogacz, I. 2000. Comparison of life-history traits of the two male morphs of the bulb mite, *Rhizoglyphus robini*. Experimental and Applied Acarology 24:115-121

Rantalainen, M.-L., Fritze, H., Haimi, J., Kiikkilä, O., Pennanen, T., Setälä, H. 2004. Do enchytraeid worms and habitat corridors facilitate the colonization of habitat patches by soil microbes? Biology and Fertility of Soils 39:200-208
Scheu, S., Falca, M. 2000. The soil food web of two beech forests (*Fagus sylvatica*) of contrasting humus type: stable isotope analysis of a macro- and a mesofauna-dominated community. Oecologia, 123:285-296

Scheu, S., Folger, M. 2004. Single and mixed diets in Collembola: effects on reproduction and stable isotope fractionation. Functional Ecology 18:94-102
Scheu, S., Setälä, H. 2002. Multitrophic interactions in decomposer food-webs. In: Tscharntke, T., Hawkins B. A. (eds.) Multitrophic Level Interactions. Cambridge University Press: Cambridge, U.K., p. 223-264
Scheu, S., Simmerling, F. 2004. Growth and reproduction of fungal feeding Collembola as affected by fungal species, melanin and mixed diets. Oecologia 139:347-353
Scheu, S., Theenhaus, A., Jones, T. H. 1999. Links between the detrivore and the herbivore system: effects of earthworms and Collembola on plant growth and aphid development. Oecologia 119:541-551
Schouten, A. J., Van Esbroek, M. L. P., Alkemade, J. R. M. 1998. Dynamics and stratification of functional groups of nematodes in the organic layer of a Scots pine forest in relation to temperature and moisture. Biology and Fertility of Soils 26:293-304
Setälä, H. 1995. Growth of birch and pine seedlings in relation to grazing by soil fauna on ectomycorrhizal fungi. Ecology 76:1844-1851
Setälä, H. 2002. Sensitivity of ecosystem functioning to changes in trophic structure, functional group composition and species diversity in belowground food webs. Ecological Research 17: 207-215
Setälä, H., Laakso, J., Mikola, J., Huhta, V. 1998. Functional diversity of decomposer organisms in relation to primary production. Applied Soil Ecology 9:25-31
Siddiqui, M. R., Hawksworth, D. L. 1982. Nematodes associated with galls on *Cladonia glauca* including 2 new species. Lichenologist 14:175-184
Siemann, E., Tilman, D., Haarstad, J. 1999. Abundance, diversity and body size: patterns from a grassland arthropod community. Journal of Animal Ecology 68:824-835
Siepel, H. 1994. Life-history tactics of soil microarthropods. Biology and Fertility of Soils 18:263-278
Siepel, H., De Ruiter-Dijkman, E. M. 1993. Feeding guilds of oribatid mites based on carbohydrase enzyme activities. Soil Biology and Biochemistry 25:1491-1497

Siepel, H., Maaskamp, F. 1994. Mites of different feeding guilds affect decomposition of organic matter. Soil Biology and Biochemistry 26:1389–1394
Small, R. W. 1987. A review of the prey of predatory soil nematodes. Pedobiologia 30:179-206
Sohlenius, B., Persson, H., Magnusson, C. 1977. Distribution of root and soil nematodes in a young Scots pine stand in central Sweden. Ecological Bulletin 25:340-347
Stefan, V. 1990. Some studies on the relations between Enchytraeidae and soil microflora. Revue Roumaine de Biologie Serie de Biologie Animale 35:101-104
Stollarova, I. 2001. Free-living and plant parasitic nematode communities of two forest nurseries in Slovakia. Biologia 56:131-139
Van Straalen, N. M. 1982. Demographic analysis of arthropod populations using a continuous stage-variable. Journal of Animal Ecology 51:769-783
Van Straalen, N. M. 1985. Comparative demography of forest floor Collembola populations. Oikos 45:253-265
Van Straalen, N. M. 1987. Turnover of accumulating substances in populations with weight-structure. Ecological Modelling 36:195-209

Van Straalen, N. M. 1989. Production and biomass turnover in two populations of forest floor Collembola. Netherlands Journal of Zoology 39:156-168

Van Straalen, N. M., Verhoef, H. A. 1997. The development of a bioindicator system for soil acidity based on arthropod pH preferences. Journal of Applied Ecology 34:217-232
Vegter, J. J. 1983. Food and habitat specialization in coexisting springtails (Collembola, Entomobryidae). Pedobiologia 25:253-262
Vegter, J. J. 1987. Phenology and seasonal resource partitioning in forest floor Collembola. Oikos 48:175-185
Vegter, J. J., De Bie, P., Dop, H. 1988a. Distributional ecology of forest floor Collembola (Entomobryidae) in the Netherlands. Pedobiologia 31:65-73
Vegter, J. J., Joosse, E. N. G., Ernsting, G. 1988b. Community structure, distribution and population dynamics of Entomobryidae (Collembola). Journal of Animal Ecology 57:971-981
Verhoef, H. A. 1995. Animal ecophysiology: cornerstone for soil ecosystem studies as exemplified by studies on arthropods. Acta Zoologica Fennica 196:176-182
Vreeken Buijs, M. J., Geurs, M., De Ruiter, P. C., Brussaard, L. 1997. The effects of bacterivorous mites and amoebae on mineralization in a detrital based below-ground food web; microcosm experiment and simulation of interactions. Pedobiologia 41:481-493
Wallace, M. K., Rust, R. H., Hawkins, D. M., Macdonald, D. H. 1993. Correlation of edaphic factors with plant-parasitic nematode population densities in a forage field. Journal of Nematology 25:642-653.
Wardle, D. A., Yeates, G. W., Watson, R. N., Nicholson, K. S. 1995. development of the decomposer food-web, trophic relationships, and ecosystem properties during a three year primary succession in sawdust. Oikos 73:155-166
Wardle, D. A., Barker, G. M., Yeates, G. W., Bonner, K. I., Ghani, A. 2001. Introduced browsing mammals in New Zealand natural forests: Aboveground and belowground consequences. Ecological Monographs 71:587-614
Wolters, V. 1985. Resource allocation in *Tomocerus flavescens* (Insecta, Collembola): a study with C-14-labelled food. Oecologia 65:229-235
Wolters, V. 1998. Long-term dynamics of a collembolan community. Applied Soil Ecology 9:221-227
Yeates, G. W. 1979. Soil nematodes in terrestrial ecosystems. Journal of Nematology 11:213-229
Yeates, G. W. 2003. Nematodes as soil indicators: functional and biodiversity aspects. Biology and Fertility of Soils 37:199-210
Yeates, G. W., Bongers, T., De Goede, R. G. M., Freckman, D. W., Georgieva, S. S. 1993. Feeding habits in soil nematode families and genera – an outline for soil ecologists. Journal of Nematology 25:315-331
Yeates, G. W., Saggar, S., Denton, C. S., Mercer, C. F. 1998. Impact of clover cyst nematode (*Heterodera trifolii*) infection on soil microbial activity in the rhizosphere of white clover (*Trifolium repens*): a pulse labelling experiment. Nematologica 44:81–90
Zolda, P. 2006. Nematode communities of grazed and ungrazed semi-natural steppe grasslands in Eastern Austria. Pedobiologia 50:11-22

Resource	Consumer	Mres	Mconsumer	Nres	Nconsumer
Aglenchus	Tripyla	**-1.053**	**-0.420**	**5.505**	**4.727**
Aglenchus	Hypoaspis	**-1.053**	**0.334**	**5.505**	**3.189**
Aglenchus	Lysigamasus	**-1.053**	**0.407**	**5.505**	**3.189**
Aglenchus	Aporcelaimellus	**-1.053**	**0.548**	**5.505**	**5.125**
Aglenchus	Dorylaimoidea	**-1.053**	**-0.604**	**5.505**	**5.204**
Aglenchus	Mesodorylaimus	**-1.053**	**-0.277**	**5.505**	**4.426**
Aglenchus	Prodorylaimus	**-1.053**	**-0.836**	**5.505**	**5.028**
Aglenchus	Pungentus	**-1.053**	**0.263**	**5.505**	**4.426**
Aglenchus	Qudsianematidae	**-1.053**	**-0.207**	**5.505**	**4.727**
Aglenchus	Thornematidae	**-1.053**	**-0.470**	**5.505**	**4.903**
Aglenchus	Eupodes	**-1.053**	**0.005**	**5.505**	**4.393**
Aglenchus	Mesostigmata	**-1.053**	**-0.411**	**5.505**	**3.189**
Aglenchus	Oribatida	**-1.053**	**-0.411**	**5.505**	**3.189**
Aglenchus	Scheloribates	**-1.053**	**0.202**	**5.505**	**3.666**
Aglenchus	Stigmaeidae	**-1.053**	**0.229**	**5.505**	**3.490**
Criconematidae	Tripyla	**-0.753**	**-0.420**	**4.426**	**4.727**
Criconematidae	Hypoaspis	**-0.753**	**0.334**	**4.426**	**3.189**
Criconematidae	Lysigamasus	**-0.753**	**0.407**	**4.426**	**3.189**
Criconematidae	Aporcelaimellus	**-0.753**	**0.548**	**4.426**	**5.125**
Criconematidae	Dorylaimoidea	**-0.753**	**-0.604**	**4.426**	**5.204**
Criconematidae	Mesodorylaimus	**-0.753**	**-0.277**	**4.426**	**4.426**
Criconematidae	Prodorylaimus	**-0.753**	**-0.836**	**4.426**	**5.028**
Criconematidae	Pungentus	**-0.753**	**0.263**	**4.426**	**4.426**
Criconematidae	Qudsianematidae	**-0.753**	**-0.207**	**4.426**	**4.727**
Criconematidae	Thornematidae	**-0.753**	**-0.470**	**4.426**	**4.903**
Criconematidae	Eupodes	**-0.753**	**0.005**	**4.426**	**4.393**
Criconematidae	Mesostigmata	**-0.753**	**-0.411**	**4.426**	**3.189**
Criconematidae	Oribatida	**-0.753**	**-0.411**	**4.426**	**3.189**
Criconematidae	Scheloribates	**-0.753**	**0.202**	**4.426**	**3.666**
Criconematidae	Stigmaeidae	**-0.753**	**0.229**	**4.426**	**3.490**
Dolichodoridae	Tripyla	**-0.885**	**-0.420**	**4.727**	**4.727**
Dolichodoridae	Hypoaspis	**-0.885**	**0.334**	**4.727**	**3.189**
Dolichodoridae	Lysigamasus	**-0.885**	**0.407**	**4.727**	**3.189**
Dolichodoridae	Aporcelaimellus	**-0.885**	**0.548**	**4.727**	**5.125**
Dolichodoridae	Dorylaimoidea	**-0.885**	**-0.604**	**4.727**	**5.204**
Dolichodoridae	Mesodorylaimus	**-0.885**	**-0.277**	**4.727**	**4.426**
Dolichodoridae	Prodorylaimus	**-0.885**	**-0.836**	**4.727**	**5.028**
Dolichodoridae	Pungentus	**-0.885**	**0.263**	**4.727**	**4.426**
Dolichodoridae	Qudsianematidae	**-0.885**	**-0.207**	**4.727**	**4.727**
Dolichodoridae	Thornematidae	**-0.885**	**-0.470**	**4.727**	**4.903**
Dolichodoridae	Eupodes	**-0.885**	**0.005**	**4.727**	**4.393**
Dolichodoridae	Mesostigmata	**-0.885**	**-0.411**	**4.727**	**3.189**
Dolichodoridae	Oribatida	**-0.885**	**-0.411**	**4.727**	**3.189**
Dolichodoridae	Scheloribates	**-0.885**	**0.202**	**4.727**	**3.666**
Dolichodoridae	Stigmaeidae	**-0.885**	**0.229**	**4.727**	**3.490**
Helicotylenchus	Tripyla	**-0.792**	**-0.420**	**4.727**	**4.727**
Helicotylenchus	Hypoaspis	**-0.792**	**0.334**	**4.727**	**3.189**
Helicotylenchus	Lysigamasus	**-0.792**	**0.407**	**4.727**	**3.189**
Helicotylenchus	Aporcelaimellus	**-0.792**	**0.548**	**4.727**	**5.125**
Helicotylenchus	Dorylaimoidea	**-0.792**	**-0.604**	**4.727**	**5.204**
Helicotylenchus	Mesodorylaimus	**-0.792**	**-0.277**	**4.727**	**4.426**
Helicotylenchus	Prodorylaimus	**-0.792**	**-0.836**	**4.727**	**5.028**
Helicotylenchus	Pungentus	**-0.792**	**0.263**	**4.727**	**4.426**
Helicotylenchus	Qudsianematidae	**-0.792**	**-0.207**	**4.727**	**4.727**
Helicotylenchus	Thornematidae	**-0.792**	**-0.470**	**4.727**	**4.903**
Helicotylenchus	Eupodes	**-0.792**	**0.005**	**4.727**	**4.393**
Helicotylenchus	Mesostigmata	**-0.792**	**-0.411**	**4.727**	**3.189**
Helicotylenchus	Oribatida	**-0.792**	**-0.411**	**4.727**	**3.189**
Helicotylenchus	Scheloribates	**-0.792**	**0.202**	**4.727**	**3.666**
Helicotylenchus	Stigmaeidae	**-0.792**	**0.229**	**4.727**	**3.490**
Malenchus	Tripyla	**-1.330**	**-0.420**	**4.426**	**4.727**
Malenchus	Hypoaspis	**-1.330**	**0.334**	**4.426**	**3.189**
Malenchus	Lysigamasus	**-1.330**	**0.407**	**4.426**	**3.189**
Malenchus	Aporcelaimellus	**-1.330**	**0.548**	**4.426**	**5.125**
Malenchus	Dorylaimoidea	**-1.330**	**-0.604**	**4.426**	**5.204**
Malenchus	Mesodorylaimus	**-1.330**	**-0.277**	**4.426**	**4.426**
Malenchus	Prodorylaimus	**-1.330**	**-0.836**	**4.426**	**5.028**
Malenchus	Pungentus	**-1.330**	**0.263**	**4.426**	**4.426**
Malenchus	Qudsianematidae	**-1.330**	**-0.207**	**4.426**	**4.727**

Malenchus	Thornematidae	-1.330	-0.470	4.426	4.903
Malenchus	Eupodes	-1.330	0.005	4.426	4.393
Malenchus	Mesostigmata	-1.330	-0.411	4.426	3.189
Malenchus	Oribatida	-1.330	-0.411	4.426	3.189
Malenchus	Scheloribates	-1.330	0.202	4.426	3.666
Malenchus	Stigmaeidae	-1.330	0.229	4.426	3.490
Paratylenchus	Tripyla	-1.244	-0.420	4.426	4.727
Paratylenchus	Hypoaspis	-1.244	0.334	4.426	3.189
Paratylenchus	Lysigamasus	-1.244	0.407	4.426	3.189
Paratylenchus	Aporcelaimellus	-1.244	0.548	4.426	5.125
Paratylenchus	Dorylaimoidea	-1.244	-0.604	4.426	5.204
Paratylenchus	Mesodorylaimus	-1.244	-0.277	4.426	4.426
Paratylenchus	Prodorylaimus	-1.244	-0.836	4.426	5.028
Paratylenchus	Pungentus	-1.244	0.263	4.426	4.426
Paratylenchus	Qudsianematidae	-1.244	-0.207	4.426	4.727
Paratylenchus	Thornematidae	-1.244	-0.470	4.426	4.903
Paratylenchus	Eupodes	-1.244	0.005	4.426	4.393
Paratylenchus	Mesostigmata	-1.244	-0.411	4.426	3.189
Paratylenchus	Oribatida	-1.244	-0.411	4.426	3.189
Paratylenchus	Scheloribates	-1.244	0.202	4.426	3.666
Paratylenchus	Stigmaeidae	-1.244	0.229	4.426	3.490
Pratylenchus	Tripyla	-1.226	-0.420	4.426	4.727
Pratylenchus	Hypoaspis	-1.226	0.334	4.426	3.189
Pratylenchus	Lysigamasus	-1.226	0.407	4.426	3.189
Pratylenchus	Aporcelaimellus	-1.226	0.548	4.426	5.125
Pratylenchus	Dorylaimoidea	-1.226	-0.604	4.426	5.204
Pratylenchus	Mesodorylaimus	-1.226	-0.277	4.426	4.426
Pratylenchus	Prodorylaimus	-1.226	-0.836	4.426	5.028
Pratylenchus	Pungentus	-1.226	0.263	4.426	4.426
Pratylenchus	Qudsianematidae	-1.226	-0.207	4.426	4.727
Pratylenchus	Thornematidae	-1.226	-0.470	4.426	4.903
Pratylenchus	Eupodes	-1.226	0.005	4.426	4.393
Pratylenchus	Mesostigmata	-1.226	-0.411	4.426	3.189
Pratylenchus	Oribatida	-1.226	-0.411	4.426	3.189
Pratylenchus	Scheloribates	-1.226	0.202	4.426	3.666
Pratylenchus	Stigmaeidae	-1.226	0.229	4.426	3.490
Trichodorus	Tripyla	-0.744	-0.420	4.426	4.727
Trichodorus	Hypoaspis	-0.744	0.334	4.426	3.189
Trichodorus	Lysigamasus	-0.744	0.407	4.426	3.189
Trichodorus	Aporcelaimellus	-0.744	0.548	4.426	5.125
Trichodorus	Dorylaimoidea	-0.744	-0.604	4.426	5.204
Trichodorus	Mesodorylaimus	-0.744	-0.277	4.426	4.426
Trichodorus	Prodorylaimus	-0.744	-0.836	4.426	5.028
Trichodorus	Pungentus	-0.744	0.263	4.426	4.426
Trichodorus	Qudsianematidae	-0.744	-0.207	4.426	4.727
Trichodorus	Thornematidae	-0.744	-0.470	4.426	4.903
Trichodorus	Eupodes	-0.744	0.005	4.426	4.393
Trichodorus	Mesostigmata	-0.744	-0.411	4.426	3.189
Trichodorus	Oribatida	-0.744	-0.411	4.426	3.189
Trichodorus	Scheloribates	-0.744	0.202	4.426	3.666
Trichodorus	Stigmaeidae	-0.744	0.229	4.426	3.490
Achipteria	Hypoaspis	0.341	0.334	3.189	3.189
Achipteria	Lysigamasus	0.341	0.407	3.189	3.189
Achipteria	Aporcelaimellus	0.341	0.548	3.189	5.125
Achipteria	Dorylaimoidea	0.341	-0.604	3.189	5.204
Achipteria	Mesodorylaimus	0.341	-0.277	3.189	4.426
Achipteria	Prodorylaimus	0.341	-0.836	3.189	5.028
Achipteria	Pungentus	0.341	0.263	3.189	4.426
Achipteria	Qudsianematidae	0.341	-0.207	3.189	4.727
Achipteria	Thornematidae	0.341	-0.470	3.189	4.903
Achipteria	Eupodes	0.341	0.005	3.189	4.393
Achipteria	Mesostigmata	0.341	-0.411	3.189	3.189
Achipteria	Oribatida	0.341	-0.411	3.189	3.189
Achipteria	Scheloribates	0.341	0.202	3.189	3.666
Achipteria	Stigmaeidae	0.341	0.229	3.189	3.490
Achipteria	Trombidiidae	0.341	1.787	3.189	3.490
Galumna	Hypoaspis	0.474	0.334	3.189	3.189
Galumna	Lysigamasus	0.474	0.407	3.189	3.189
Galumna	Aporcelaimellus	0.474	0.548	3.189	5.125
Galumna	Dorylaimoidea	0.474	-0.604	3.189	5.204

Galumna	Mesodorylaimus	**0.474**	**-0.277**	**3.189**	**4.426**
Galumna	Prodorylaimus	**0.474**	**-0.836**	**3.189**	**5.028**
Galumna	Pungentus	**0.474**	**0.263**	**3.189**	**4.426**
Galumna	Qudsianematidae	**0.474**	**-0.207**	**3.189**	**4.727**
Galumna	Thornematidae	**0.474**	**-0.470**	**3.189**	**4.903**
Galumna	Eupodes	**0.474**	**0.005**	**3.189**	**4.393**
Galumna	Mesostigmata	**0.474**	**-0.411**	**3.189**	**3.189**
Galumna	Oribatida	**0.474**	**-0.411**	**3.189**	**3.189**
Galumna	Scheloribates	**0.474**	**0.202**	**3.189**	**3.666**
Galumna	Stigmaeidae	**0.474**	**0.229**	**3.189**	**3.490**
Galumna	Trombidiidae	**0.474**	**1.787**	**3.189**	**3.490**
Platynothrus	Hypoaspis	**0.710**	**0.334**	**4.034**	**3.189**
Platynothrus	Lysigamasus	**0.710**	**0.407**	**4.034**	**3.189**
Platynothrus	Aporcelaimellus	**0.710**	**0.548**	**4.034**	**5.125**
Platynothrus	Dorylaimoidea	**0.710**	**-0.604**	**4.034**	**5.204**
Platynothrus	Mesodorylaimus	**0.710**	**-0.277**	**4.034**	**4.426**
Platynothrus	Prodorylaimus	**0.710**	**-0.836**	**4.034**	**5.028**
Platynothrus	Pungentus	**0.710**	**0.263**	**4.034**	**4.426**
Platynothrus	Qudsianematidae	**0.710**	**-0.207**	**4.034**	**4.727**
Platynothrus	Thornematidae	**0.710**	**-0.470**	**4.034**	**4.903**
Platynothrus	Eupodes	**0.710**	**0.005**	**4.034**	**4.393**
Platynothrus	Mesostigmata	**0.710**	**-0.411**	**4.034**	**3.189**
Platynothrus	Oribatida	**0.710**	**-0.411**	**4.034**	**3.189**
Platynothrus	Scheloribates	**0.710**	**0.202**	**4.034**	**3.666**
Platynothrus	Stigmaeidae	**0.710**	**0.229**	**4.034**	**3.490**
Platynothrus	Trombidiidae	**0.710**	**1.787**	**4.034**	**3.490**
Tydeidae	Hypoaspis	**-0.608**	**0.334**	**3.967**	**3.189**
Tydeidae	Lysigamasus	**-0.608**	**0.407**	**3.967**	**3.189**
Tydeidae	Aporcelaimellus	**-0.608**	**0.548**	**3.967**	**5.125**
Tydeidae	Dorylaimoidea	**-0.608**	**-0.604**	**3.967**	**5.204**
Tydeidae	Mesodorylaimus	**-0.608**	**-0.277**	**3.967**	**4.426**
Tydeidae	Prodorylaimus	**-0.608**	**-0.836**	**3.967**	**5.028**
Tydeidae	Pungentus	**-0.608**	**0.263**	**3.967**	**4.426**
Tydeidae	Qudsianematidae	**-0.608**	**-0.207**	**3.967**	**4.727**
Tydeidae	Thornematidae	**-0.608**	**-0.470**	**3.967**	**4.903**
Tydeidae	Eupodes	**-0.608**	**0.005**	**3.967**	**4.393**
Tydeidae	Mesostigmata	**-0.608**	**-0.411**	**3.967**	**3.189**
Tydeidae	Oribatida	**-0.608**	**-0.411**	**3.967**	**3.189**
Tydeidae	Scheloribates	**-0.608**	**0.202**	**3.967**	**3.666**
Tydeidae	Stigmaeidae	**-0.608**	**0.229**	**3.967**	**3.490**
Tydeidae	Trombidiidae	**-0.608**	**1.787**	**3.967**	**3.490**
Sminthuridae	Hypoaspis	**-0.608**	**0.334**	**3.490**	**3.189**
Sminthuridae	Lysigamasus	**-0.608**	**0.407**	**3.490**	**3.189**
Sminthuridae	Aporcelaimellus	**-0.608**	**0.548**	**3.490**	**5.125**
Sminthuridae	Dorylaimoidea	**-0.608**	**-0.604**	**3.490**	**5.204**
Sminthuridae	Mesodorylaimus	**-0.608**	**-0.277**	**3.490**	**4.426**
Sminthuridae	Prodorylaimus	**-0.608**	**-0.836**	**3.490**	**5.028**
Sminthuridae	Pungentus	**-0.608**	**0.263**	**3.490**	**4.426**
Sminthuridae	Qudsianematidae	**-0.608**	**-0.207**	**3.490**	**4.727**
Sminthuridae	Thornematidae	**-0.608**	**-0.470**	**3.490**	**4.903**
Sminthuridae	Eupodes	**-0.608**	**0.005**	**3.490**	**4.393**
Sminthuridae	Mesostigmata	**-0.608**	**-0.411**	**3.490**	**3.189**
Sminthuridae	Oribatida	**-0.608**	**-0.411**	**3.490**	**3.189**
Sminthuridae	Scheloribates	**-0.608**	**0.202**	**3.490**	**3.666**
Sminthuridae	Stigmaeidae	**-0.608**	**0.229**	**3.490**	**3.490**
Sminthurinus	Hypoaspis	**0.618**	**0.334**	**3.666**	**3.189**
Sminthurinus	Lysigamasus	**0.618**	**0.407**	**3.666**	**3.189**
Sminthurinus	Aporcelaimellus	**0.618**	**0.548**	**3.666**	**5.125**
Sminthurinus	Dorylaimoidea	**0.618**	**-0.604**	**3.666**	**5.204**
Sminthurinus	Mesodorylaimus	**0.618**	**-0.277**	**3.666**	**4.426**
Sminthurinus	Prodorylaimus	**0.618**	**-0.836**	**3.666**	**5.028**
Sminthurinus	Pungentus	**0.618**	**0.263**	**3.666**	**4.426**
Sminthurinus	Qudsianematidae	**0.618**	**-0.207**	**3.666**	**4.727**
Sminthurinus	Thornematidae	**0.618**	**-0.470**	**3.666**	**4.903**
Sminthurinus	Eupodes	**0.618**	**0.005**	**3.666**	**4.393**
Sminthurinus	Mesostigmata	**0.618**	**-0.411**	**3.666**	**3.189**
Sminthurinus	Oribatida	**0.618**	**-0.411**	**3.666**	**3.189**
Sminthurinus	Scheloribates	**0.618**	**0.202**	**3.666**	**3.666**
Sminthurinus	Stigmaeidae	**0.618**	**0.229**	**3.666**	**3.490**
Aphelenchoides	Tripyla	**-1.496**	**-0.420**	**5.426**	**4.727**

Aphelenchoides	Hypoaspis	**-1.496**	**0.334**	**5.426**	**3.189**
Aphelenchoides	Lysigamasus	**-1.496**	**0.407**	**5.426**	**3.189**
Aphelenchoides	Aporcelaimellus	**-1.496**	**0.548**	**5.426**	**5.125**
Aphelenchoides	Dorylaimoidea	**-1.496**	**-0.604**	**5.426**	**5.204**
Aphelenchoides	Mesodorylaimus	**-1.496**	**-0.277**	**5.426**	**4.426**
Aphelenchoides	Prodorylaimus	**-1.496**	**-0.836**	**5.426**	**5.028**
Aphelenchoides	Pungentus	**-1.496**	**0.263**	**5.426**	**4.426**
Aphelenchoides	Qudsianematidae	**-1.496**	**-0.207**	**5.426**	**4.727**
Aphelenchoides	Thornematidae	**-1.496**	**-0.470**	**5.426**	**4.903**
Aphelenchoides	Eupodes	**-1.496**	**0.005**	**5.426**	**4.393**
Aphelenchoides	Mesostigmata	**-1.496**	**-0.411**	**5.426**	**3.189**
Aphelenchoides	Oribatida	**-1.496**	**-0.411**	**5.426**	**3.189**
Aphelenchoides	Scheloribates	**-1.496**	**0.202**	**5.426**	**3.666**
Aphelenchoides	Stigmaeidae	**-1.496**	**0.229**	**5.426**	**3.490**
Tylenchidae	Tripyla	**-1.360**	**-0.420**	**6.039**	**4.727**
Tylenchidae	Hypoaspis	**-1.360**	**0.334**	**6.039**	**3.189**
Tylenchidae	Lysigamasus	**-1.360**	**0.407**	**6.039**	**3.189**
Tylenchidae	Aporcelaimellus	**-1.360**	**0.548**	**6.039**	**5.125**
Tylenchidae	Dorylaimoidea	**-1.360**	**-0.604**	**6.039**	**5.204**
Tylenchidae	Mesodorylaimus	**-1.360**	**-0.277**	**6.039**	**4.426**
Tylenchidae	Prodorylaimus	**-1.360**	**-0.836**	**6.039**	**5.028**
Tylenchidae	Pungentus	**-1.360**	**0.263**	**6.039**	**4.426**
Tylenchidae	Qudsianematidae	**-1.360**	**-0.207**	**6.039**	**4.727**
Tylenchidae	Thornematidae	**-1.360**	**-0.470**	**6.039**	**4.903**
Tylenchidae	Eupodes	**-1.360**	**0.005**	**6.039**	**4.393**
Tylenchidae	Mesostigmata	**-1.360**	**-0.411**	**6.039**	**3.189**
Tylenchidae	Oribatida	**-1.360**	**-0.411**	**6.039**	**3.189**
Tylenchidae	Scheloribates	**-1.360**	**0.202**	**6.039**	**3.666**
Tylenchidae	Stigmaeidae	**-1.360**	**0.229**	**6.039**	**3.490**
Liochthonius	Hypoaspis	**-0.653**	**0.334**	**3.791**	**3.189**
Liochthonius	Lysigamasus	**-0.653**	**0.407**	**3.791**	**3.189**
Liochthonius	Aporcelaimellus	**-0.653**	**0.548**	**3.791**	**5.125**
Liochthonius	Dorylaimoidea	**-0.653**	**-0.604**	**3.791**	**5.204**
Liochthonius	Mesodorylaimus	**-0.653**	**-0.277**	**3.791**	**4.426**
Liochthonius	Prodorylaimus	**-0.653**	**-0.836**	**3.791**	**5.028**
Liochthonius	Pungentus	**-0.653**	**0.263**	**3.791**	**4.426**
Liochthonius	Qudsianematidae	**-0.653**	**-0.207**	**3.791**	**4.727**
Liochthonius	Thornematidae	**-0.653**	**-0.470**	**3.791**	**4.903**
Liochthonius	Eupodes	**-0.653**	**0.005**	**3.791**	**4.393**
Liochthonius	Mesostigmata	**-0.653**	**-0.411**	**3.791**	**3.189**
Liochthonius	Oribatida	**-0.653**	**-0.411**	**3.791**	**3.189**
Liochthonius	Scheloribates	**-0.653**	**0.202**	**3.791**	**3.666**
Liochthonius	Stigmaeidae	**-0.653**	**0.229**	**3.791**	**3.490**
Liochthonius	Trombidiidae	**-0.653**	**1.787**	**3.791**	**3.490**
Medioppia	Hypoaspis	**-0.235**	**0.334**	**3.490**	**3.189**
Medioppia	Lysigamasus	**-0.235**	**0.407**	**3.490**	**3.189**
Medioppia	Aporcelaimellus	**-0.235**	**0.548**	**3.490**	**5.125**
Medioppia	Dorylaimoidea	**-0.235**	**-0.604**	**3.490**	**5.204**
Medioppia	Mesodorylaimus	**-0.235**	**-0.277**	**3.490**	**4.426**
Medioppia	Prodorylaimus	**-0.235**	**-0.836**	**3.490**	**5.028**
Medioppia	Pungentus	**-0.235**	**0.263**	**3.490**	**4.426**
Medioppia	Qudsianematidae	**-0.235**	**-0.207**	**3.490**	**4.727**
Medioppia	Thornematidae	**-0.235**	**-0.470**	**3.490**	**4.903**
Medioppia	Eupodes	**-0.235**	**0.005**	**3.490**	**4.393**
Medioppia	Mesostigmata	**-0.235**	**-0.411**	**3.490**	**3.189**
Medioppia	Oribatida	**-0.235**	**-0.411**	**3.490**	**3.189**
Medioppia	Scheloribates	**-0.235**	**0.202**	**3.490**	**3.666**
Medioppia	Stigmaeidae	**-0.235**	**0.229**	**3.490**	**3.490**
Medioppia	Trombidiidae	**-0.235**	**1.787**	**3.490**	**3.490**
Microtydeus	Hypoaspis	**-0.863**	**0.334**	**3.189**	**3.189**
Microtydeus	Lysigamasus	**-0.863**	**0.407**	**3.189**	**3.189**
Microtydeus	Aporcelaimellus	**-0.863**	**0.548**	**3.189**	**5.125**
Microtydeus	Dorylaimoidea	**-0.863**	**-0.604**	**3.189**	**5.204**
Microtydeus	Mesodorylaimus	**-0.863**	**-0.277**	**3.189**	**4.426**
Microtydeus	Prodorylaimus	**-0.863**	**-0.836**	**3.189**	**5.028**
Microtydeus	Pungentus	**-0.863**	**0.263**	**3.189**	**4.426**
Microtydeus	Qudsianematidae	**-0.863**	**-0.207**	**3.189**	**4.727**
Microtydeus	Thornematidae	**-0.863**	**-0.470**	**3.189**	**4.903**
Microtydeus	Eupodes	**-0.863**	**0.005**	**3.189**	**4.393**
Microtydeus	Mesostigmata	**-0.863**	**-0.411**	**3.189**	**3.189**

Microtydeus	Oribatida	**-0.863**	**-0.411**	**3.189**	**3.189**
Microtydeus	Scheloribates	**-0.863**	**0.202**	**3.189**	**3.666**
Microtydeus	Stigmaeidae	**-0.863**	**0.229**	**3.189**	**3.490**
Microtydeus	Trombidiidae	**-0.863**	**1.787**	**3.189**	**3.490**
Pygmephorus	Hypoaspis	**-0.376**	**0.334**	**3.490**	**3.189**
Pygmephorus	Lysigamasus	**-0.376**	**0.407**	**3.490**	**3.189**
Pygmephorus	Aporcelaimellus	**-0.376**	**0.548**	**3.490**	**5.125**
Pygmephorus	Dorylaimoidea	**-0.376**	**-0.604**	**3.490**	**5.204**
Pygmephorus	Mesodorylaimus	**-0.376**	**-0.277**	**3.490**	**4.426**
Pygmephorus	Prodorylaimus	**-0.376**	**-0.836**	**3.490**	**5.028**
Pygmephorus	Pungentus	**-0.376**	**0.263**	**3.490**	**4.426**
Pygmephorus	Qudsianematidae	**-0.376**	**-0.207**	**3.490**	**4.727**
Pygmephorus	Thornematidae	**-0.376**	**-0.470**	**3.490**	**4.903**
Pygmephorus	Eupodes	**-0.376**	**0.005**	**3.490**	**4.393**
Pygmephorus	Mesostigmata	**-0.376**	**-0.411**	**3.490**	**3.189**
Pygmephorus	Oribatida	**-0.376**	**-0.411**	**3.490**	**3.189**
Pygmephorus	Scheloribates	**-0.376**	**0.202**	**3.490**	**3.666**
Pygmephorus	Stigmaeidae	**-0.376**	**0.229**	**3.490**	**3.490**
Pygmephorus	Trombidiidae	**-0.376**	**1.787**	**3.490**	**3.490**
Tectocepheus	Hypoaspis	**-0.220**	**0.334**	**3.666**	**3.189**
Tectocepheus	Lysigamasus	**-0.220**	**0.407**	**3.666**	**3.189**
Tectocepheus	Aporcelaimellus	**-0.220**	**0.548**	**3.666**	**5.125**
Tectocepheus	Dorylaimoidea	**-0.220**	**-0.604**	**3.666**	**5.204**
Tectocepheus	Mesodorylaimus	**-0.220**	**-0.277**	**3.666**	**4.426**
Tectocepheus	Prodorylaimus	**-0.220**	**-0.836**	**3.666**	**5.028**
Tectocepheus	Pungentus	**-0.220**	**0.263**	**3.666**	**4.426**
Tectocepheus	Qudsianematidae	**-0.220**	**-0.207**	**3.666**	**4.727**
Tectocepheus	Thornematidae	**-0.220**	**-0.470**	**3.666**	**4.903**
Tectocepheus	Eupodes	**-0.220**	**0.005**	**3.666**	**4.393**
Tectocepheus	Mesostigmata	**-0.220**	**-0.411**	**3.666**	**3.189**
Tectocepheus	Oribatida	**-0.220**	**-0.411**	**3.666**	**3.189**
Tectocepheus	Scheloribates	**-0.220**	**0.202**	**3.666**	**3.666**
Tectocepheus	Stigmaeidae	**-0.220**	**0.229**	**3.666**	**3.490**
Tectocepheus	Trombidiidae	**-0.220**	**1.787**	**3.666**	**3.490**
Trichoribates	Hypoaspis	**0.474**	**0.334**	**3.666**	**3.189**
Trichoribates	Lysigamasus	**0.474**	**0.407**	**3.666**	**3.189**
Trichoribates	Aporcelaimellus	**0.474**	**0.548**	**3.666**	**5.125**
Trichoribates	Dorylaimoidea	**0.474**	**-0.604**	**3.666**	**5.204**
Trichoribates	Mesodorylaimus	**0.474**	**-0.277**	**3.666**	**4.426**
Trichoribates	Prodorylaimus	**0.474**	**-0.836**	**3.666**	**5.028**
Trichoribates	Pungentus	**0.474**	**0.263**	**3.666**	**4.426**
Trichoribates	Qudsianematidae	**0.474**	**-0.207**	**3.666**	**4.727**
Trichoribates	Thornematidae	**0.474**	**-0.470**	**3.666**	**4.903**
Trichoribates	Eupodes	**0.474**	**0.005**	**3.666**	**4.393**
Trichoribates	Mesostigmata	**0.474**	**-0.411**	**3.666**	**3.189**
Trichoribates	Oribatida	**0.474**	**-0.411**	**3.666**	**3.189**
Trichoribates	Scheloribates	**0.474**	**0.202**	**3.666**	**3.666**
Trichoribates	Stigmaeidae	**0.474**	**0.229**	**3.666**	**3.490**
Trichoribates	Trombidiidae	**0.474**	**1.787**	**3.666**	**3.490**
Brachystomella	Hypoaspis	**0.977**	**0.334**	**3.189**	**3.189**
Brachystomella	Lysigamasus	**0.977**	**0.407**	**3.189**	**3.189**
Brachystomella	Aporcelaimellus	**0.977**	**0.548**	**3.189**	**5.125**
Brachystomella	Dorylaimoidea	**0.977**	**-0.604**	**3.189**	**5.204**
Brachystomella	Mesodorylaimus	**0.977**	**-0.277**	**3.189**	**4.426**
Brachystomella	Prodorylaimus	**0.977**	**-0.836**	**3.189**	**5.028**
Brachystomella	Pungentus	**0.977**	**0.263**	**3.189**	**4.426**
Brachystomella	Qudsianematidae	**0.977**	**-0.207**	**3.189**	**4.727**
Brachystomella	Thornematidae	**0.977**	**-0.470**	**3.189**	**4.903**
Brachystomella	Eupodes	**0.977**	**0.005**	**3.189**	**4.393**
Brachystomella	Mesostigmata	**0.977**	**-0.411**	**3.189**	**3.189**
Brachystomella	Oribatida	**0.977**	**-0.411**	**3.189**	**3.189**
Brachystomella	Scheloribates	**0.977**	**0.202**	**3.189**	**3.666**
Brachystomella	Stigmaeidae	**0.977**	**0.229**	**3.189**	**3.490**
Lepidocyrtus	Hypoaspis	**1.231**	**0.334**	**3.967**	**3.189**
Lepidocyrtus	Lysigamasus	**1.231**	**0.407**	**3.967**	**3.189**
Lepidocyrtus	Aporcelaimellus	**1.231**	**0.548**	**3.967**	**5.125**
Lepidocyrtus	Dorylaimoidea	**1.231**	**-0.604**	**3.967**	**5.204**
Lepidocyrtus	Mesodorylaimus	**1.231**	**-0.277**	**3.967**	**4.426**
Lepidocyrtus	Prodorylaimus	**1.231**	**-0.836**	**3.967**	**5.028**
Lepidocyrtus	Pungentus	**1.231**	**0.263**	**3.967**	**4.426**

Lepidocyrtus	Qudsianematidae	**1.231**	**-0.207**	**3.967**	**4.727**
Lepidocyrtus	Thornematidae	**1.231**	**-0.470**	**3.967**	**4.903**
Lepidocyrtus	Eupodes	**1.231**	**0.005**	**3.967**	**4.393**
Lepidocyrtus	Mesostigmata	**1.231**	**-0.411**	**3.967**	**3.189**
Lepidocyrtus	Oribatida	**1.231**	**-0.411**	**3.967**	**3.189**
Lepidocyrtus	Scheloribates	**1.231**	**0.202**	**3.967**	**3.666**
Lepidocyrtus	Stigmaeidae	**1.231**	**0.229**	**3.967**	**3.490**
Achaeta	Hypoaspis	**1.104**	**0.334**	**2.373**	**3.189**
Achaeta	Lysigamasus	**1.104**	**0.407**	**2.373**	**3.189**
Achaeta	Aporcelaimellus	**1.104**	**0.548**	**2.373**	**5.125**
Achaeta	Dorylaimoidea	**1.104**	**-0.604**	**2.373**	**5.204**
Achaeta	Mesodorylaimus	**1.104**	**-0.277**	**2.373**	**4.426**
Achaeta	Prodorylaimus	**1.104**	**-0.836**	**2.373**	**5.028**
Achaeta	Pungentus	**1.104**	**0.263**	**2.373**	**4.426**
Achaeta	Qudsianematidae	**1.104**	**-0.207**	**2.373**	**4.727**
Achaeta	Thornematidae	**1.104**	**-0.470**	**2.373**	**4.903**
Achaeta	Eupodes	**1.104**	**0.005**	**2.373**	**4.393**
Achaeta	Mesostigmata	**1.104**	**-0.411**	**2.373**	**3.189**
Achaeta	Oribatida	**1.104**	**-0.411**	**2.373**	**3.189**
Achaeta	Scheloribates	**1.104**	**0.202**	**2.373**	**3.666**
Achaeta	Stigmaeidae	**1.104**	**0.229**	**2.373**	**3.490**
Cognettia	Hypoaspis	**1.682**	**0.334**	**3.891**	**3.189**
Cognettia	Lysigamasus	**1.682**	**0.407**	**3.891**	**3.189**
Cognettia	Aporcelaimellus	**1.682**	**0.548**	**3.891**	**5.125**
Cognettia	Dorylaimoidea	**1.682**	**-0.604**	**3.891**	**5.204**
Cognettia	Mesodorylaimus	**1.682**	**-0.277**	**3.891**	**4.426**
Cognettia	Prodorylaimus	**1.682**	**-0.836**	**3.891**	**5.028**
Cognettia	Pungentus	**1.682**	**0.263**	**3.891**	**4.426**
Cognettia	Qudsianematidae	**1.682**	**-0.207**	**3.891**	**4.727**
Cognettia	Thornematidae	**1.682**	**-0.470**	**3.891**	**4.903**
Cognettia	Eupodes	**1.682**	**0.005**	**3.891**	**4.393**
Cognettia	Mesostigmata	**1.682**	**-0.411**	**3.891**	**3.189**
Cognettia	Oribatida	**1.682**	**-0.411**	**3.891**	**3.189**
Cognettia	Scheloribates	**1.682**	**0.202**	**3.891**	**3.666**
Cognettia	Stigmaeidae	**1.682**	**0.229**	**3.891**	**3.490**
Fridericia	Hypoaspis	**2.856**	**0.334**	**3.248**	**3.189**
Fridericia	Lysigamasus	**2.856**	**0.407**	**3.248**	**3.189**
Fridericia	Aporcelaimellus	**2.856**	**0.548**	**3.248**	**5.125**
Fridericia	Dorylaimoidea	**2.856**	**-0.604**	**3.248**	**5.204**
Fridericia	Mesodorylaimus	**2.856**	**-0.277**	**3.248**	**4.426**
Fridericia	Prodorylaimus	**2.856**	**-0.836**	**3.248**	**5.028**
Fridericia	Pungentus	**2.856**	**0.263**	**3.248**	**4.426**
Fridericia	Qudsianematidae	**2.856**	**-0.207**	**3.248**	**4.727**
Fridericia	Thornematidae	**2.856**	**-0.470**	**3.248**	**4.903**
Fridericia	Eupodes	**2.856**	**0.005**	**3.248**	**4.393**
Fridericia	Mesostigmata	**2.856**	**-0.411**	**3.248**	**3.189**
Fridericia	Oribatida	**2.856**	**-0.411**	**3.248**	**3.189**
Fridericia	Scheloribates	**2.856**	**0.202**	**3.248**	**3.666**
Fridericia	Stigmaeidae	**2.856**	**0.229**	**3.248**	**3.490**
Acrobeles	Tripyla	**-0.721**	**-0.420**	**4.426**	**4.727**
Acrobeles	Hypoaspis	**-0.721**	**0.334**	**4.426**	**3.189**
Acrobeles	Lysigamasus	**-0.721**	**0.407**	**4.426**	**3.189**
Acrobeles	Aporcelaimellus	**-0.721**	**0.548**	**4.426**	**5.125**
Acrobeles	Dorylaimoidea	**-0.721**	**-0.604**	**4.426**	**5.204**
Acrobeles	Mesodorylaimus	**-0.721**	**-0.277**	**4.426**	**4.426**
Acrobeles	Prodorylaimus	**-0.721**	**-0.836**	**4.426**	**5.028**
Acrobeles	Pungentus	**-0.721**	**0.263**	**4.426**	**4.426**
Acrobeles	Qudsianematidae	**-0.721**	**-0.207**	**4.426**	**4.727**
Acrobeles	Thornematidae	**-0.721**	**-0.470**	**4.426**	**4.903**
Acrobeles	Eupodes	**-0.721**	**0.005**	**4.426**	**4.393**
Acrobeles	Mesostigmata	**-0.721**	**-0.411**	**4.426**	**3.189**
Acrobeles	Oribatida	**-0.721**	**-0.411**	**4.426**	**3.189**
Acrobeles	Scheloribates	**-0.721**	**0.202**	**4.426**	**3.666**
Acrobeles	Stigmaeidae	**-0.721**	**0.229**	**4.426**	**3.490**
Acrobeloides	Tripyla	**-1.171**	**-0.420**	**5.204**	**4.727**
Acrobeloides	Hypoaspis	**-1.171**	**0.334**	**5.204**	**3.189**
Acrobeloides	Lysigamasus	**-1.171**	**0.407**	**5.204**	**3.189**
Acrobeloides	Aporcelaimellus	**-1.171**	**0.548**	**5.204**	**5.125**
Acrobeloides	Dorylaimoidea	**-1.171**	**-0.604**	**5.204**	**5.204**
Acrobeloides	Mesodorylaimus	**-1.171**	**-0.277**	**5.204**	**4.426**

Acrobeloides	Prodorylaimus	**-1.171**	**-0.836**	**5.204**	**5.028**
Acrobeloides	Pungentus	**-1.171**	**0.263**	**5.204**	**4.426**
Acrobeloides	Qudsianematidae	**-1.171**	**-0.207**	**5.204**	**4.727**
Acrobeloides	Thornematidae	**-1.171**	**-0.470**	**5.204**	**4.903**
Acrobeloides	Eupodes	**-1.171**	**0.005**	**5.204**	**4.393**
Acrobeloides	Mesostigmata	**-1.171**	**-0.411**	**5.204**	**3.189**
Acrobeloides	Oribatida	**-1.171**	**-0.411**	**5.204**	**3.189**
Acrobeloides	Scheloribates	**-1.171**	**0.202**	**5.204**	**3.666**
Acrobeloides	Stigmaeidae	**-1.171**	**0.229**	**5.204**	**3.490**
Alaimus	Tripyla	**-0.858**	**-0.420**	**4.426**	**4.727**
Alaimus	Hypoaspis	**-0.858**	**0.334**	**4.426**	**3.189**
Alaimus	Lysigamasus	**-0.858**	**0.407**	**4.426**	**3.189**
Alaimus	Aporcelaimellus	**-0.858**	**0.548**	**4.426**	**5.125**
Alaimus	Dorylaimoidea	**-0.858**	**-0.604**	**4.426**	**5.204**
Alaimus	Mesodorylaimus	**-0.858**	**-0.277**	**4.426**	**4.426**
Alaimus	Prodorylaimus	**-0.858**	**-0.836**	**4.426**	**5.028**
Alaimus	Pungentus	**-0.858**	**0.263**	**4.426**	**4.426**
Alaimus	Qudsianematidae	**-0.858**	**-0.207**	**4.426**	**4.727**
Alaimus	Thornematidae	**-0.858**	**-0.470**	**4.426**	**4.903**
Alaimus	Eupodes	**-0.858**	**0.005**	**4.426**	**4.393**
Alaimus	Mesostigmata	**-0.858**	**-0.411**	**4.426**	**3.189**
Alaimus	Oribatida	**-0.858**	**-0.411**	**4.426**	**3.189**
Alaimus	Scheloribates	**-0.858**	**0.202**	**4.426**	**3.666**
Alaimus	Stigmaeidae	**-0.858**	**0.229**	**4.426**	**3.490**
Anaplectus	Tripyla	**-0.519**	**-0.420**	**4.727**	**4.727**
Anaplectus	Hypoaspis	**-0.519**	**0.334**	**4.727**	**3.189**
Anaplectus	Lysigamasus	**-0.519**	**0.407**	**4.727**	**3.189**
Anaplectus	Aporcelaimellus	**-0.519**	**0.548**	**4.727**	**5.125**
Anaplectus	Dorylaimoidea	**-0.519**	**-0.604**	**4.727**	**5.204**
Anaplectus	Mesodorylaimus	**-0.519**	**-0.277**	**4.727**	**4.426**
Anaplectus	Prodorylaimus	**-0.519**	**-0.836**	**4.727**	**5.028**
Anaplectus	Pungentus	**-0.519**	**0.263**	**4.727**	**4.426**
Anaplectus	Qudsianematidae	**-0.519**	**-0.207**	**4.727**	**4.727**
Anaplectus	Thornematidae	**-0.519**	**-0.470**	**4.727**	**4.903**
Anaplectus	Eupodes	**-0.519**	**0.005**	**4.727**	**4.393**
Anaplectus	Mesostigmata	**-0.519**	**-0.411**	**4.727**	**3.189**
Anaplectus	Oribatida	**-0.519**	**-0.411**	**4.727**	**3.189**
Anaplectus	Scheloribates	**-0.519**	**0.202**	**4.727**	**3.666**
Anaplectus	Stigmaeidae	**-0.519**	**0.229**	**4.727**	**3.490**
Bunonema	Tripyla	**-0.676**	**-0.420**	**4.426**	**4.727**
Bunonema	Hypoaspis	**-0.676**	**0.334**	**4.426**	**3.189**
Bunonema	Lysigamasus	**-0.676**	**0.407**	**4.426**	**3.189**
Bunonema	Aporcelaimellus	**-0.676**	**0.548**	**4.426**	**5.125**
Bunonema	Dorylaimoidea	**-0.676**	**-0.604**	**4.426**	**5.204**
Bunonema	Mesodorylaimus	**-0.676**	**-0.277**	**4.426**	**4.426**
Bunonema	Prodorylaimus	**-0.676**	**-0.836**	**4.426**	**5.028**
Bunonema	Pungentus	**-0.676**	**0.263**	**4.426**	**4.426**
Bunonema	Qudsianematidae	**-0.676**	**-0.207**	**4.426**	**4.727**
Bunonema	Thornematidae	**-0.676**	**-0.470**	**4.426**	**4.903**
Bunonema	Eupodes	**-0.676**	**0.005**	**4.426**	**4.393**
Bunonema	Mesostigmata	**-0.676**	**-0.411**	**4.426**	**3.189**
Bunonema	Oribatida	**-0.676**	**-0.411**	**4.426**	**3.189**
Bunonema	Scheloribates	**-0.676**	**0.202**	**4.426**	**3.666**
Bunonema	Stigmaeidae	**-0.676**	**0.229**	**4.426**	**3.490**
Eucephalobus	Tripyla	**-0.855**	**-0.420**	**5.380**	**4.727**
Eucephalobus	Hypoaspis	**-0.855**	**0.334**	**5.380**	**3.189**
Eucephalobus	Lysigamasus	**-0.855**	**0.407**	**5.380**	**3.189**
Eucephalobus	Aporcelaimellus	**-0.855**	**0.548**	**5.380**	**5.125**
Eucephalobus	Dorylaimoidea	**-0.855**	**-0.604**	**5.380**	**5.204**
Eucephalobus	Mesodorylaimus	**-0.855**	**-0.277**	**5.380**	**4.426**
Eucephalobus	Prodorylaimus	**-0.855**	**-0.836**	**5.380**	**5.028**
Eucephalobus	Pungentus	**-0.855**	**0.263**	**5.380**	**4.426**
Eucephalobus	Qudsianematidae	**-0.855**	**-0.207**	**5.380**	**4.727**
Eucephalobus	Thornematidae	**-0.855**	**-0.470**	**5.380**	**4.903**
Eucephalobus	Eupodes	**-0.855**	**0.005**	**5.380**	**4.393**
Eucephalobus	Mesostigmata	**-0.855**	**-0.411**	**5.380**	**3.189**
Eucephalobus	Oribatida	**-0.855**	**-0.411**	**5.380**	**3.189**
Eucephalobus	Scheloribates	**-0.855**	**0.202**	**5.380**	**3.666**
Eucephalobus	Stigmaeidae	**-0.855**	**0.229**	**5.380**	**3.490**
Eumonhystera	Tripyla	**-0.613**	**-0.420**	**4.426**	**4.727**

Eumonhystera	Hypoaspis	**-0.613**	**0.334**	**4.426**	**3.189**
Eumonhystera	Lysigamasus	**-0.613**	**0.407**	**4.426**	**3.189**
Eumonhystera	Aporcelaimellus	**-0.613**	**0.548**	**4.426**	**5.125**
Eumonhystera	Dorylaimoidea	**-0.613**	**-0.604**	**4.426**	**5.204**
Eumonhystera	Mesodorylaimus	**-0.613**	**-0.277**	**4.426**	**4.426**
Eumonhystera	Prodorylaimus	**-0.613**	**-0.836**	**4.426**	**5.028**
Eumonhystera	Pungentus	**-0.613**	**0.263**	**4.426**	**4.426**
Eumonhystera	Qudsianematidae	**-0.613**	**-0.207**	**4.426**	**4.727**
Eumonhystera	Thornematidae	**-0.613**	**-0.470**	**4.426**	**4.903**
Eumonhystera	Eupodes	**-0.613**	**0.005**	**4.426**	**4.393**
Eumonhystera	Mesostigmata	**-0.613**	**-0.411**	**4.426**	**3.189**
Eumonhystera	Oribatida	**-0.613**	**-0.411**	**4.426**	**3.189**
Eumonhystera	Scheloribates	**-0.613**	**0.202**	**4.426**	**3.666**
Eumonhystera	Stigmaeidae	**-0.613**	**0.229**	**4.426**	**3.490**
Metateratocephalus	Tripyla	**-1.506**	**-0.420**	**5.329**	**4.727**
Metateratocephalus	Hypoaspis	**-1.506**	**0.334**	**5.329**	**3.189**
Metateratocephalus	Lysigamasus	**-1.506**	**0.407**	**5.329**	**3.189**
Metateratocephalus	Aporcelaimellus	**-1.506**	**0.548**	**5.329**	**5.125**
Metateratocephalus	Dorylaimoidea	**-1.506**	**-0.604**	**5.329**	**5.204**
Metateratocephalus	Mesodorylaimus	**-1.506**	**-0.277**	**5.329**	**4.426**
Metateratocephalus	Prodorylaimus	**-1.506**	**-0.836**	**5.329**	**5.028**
Metateratocephalus	Pungentus	**-1.506**	**0.263**	**5.329**	**4.426**
Metateratocephalus	Qudsianematidae	**-1.506**	**-0.207**	**5.329**	**4.727**
Metateratocephalus	Thornematidae	**-1.506**	**-0.470**	**5.329**	**4.903**
Metateratocephalus	Eupodes	**-1.506**	**0.005**	**5.329**	**4.393**
Metateratocephalus	Mesostigmata	**-1.506**	**-0.411**	**5.329**	**3.189**
Metateratocephalus	Oribatida	**-1.506**	**-0.411**	**5.329**	**3.189**
Metateratocephalus	Scheloribates	**-1.506**	**0.202**	**5.329**	**3.666**
Metateratocephalus	Stigmaeidae	**-1.506**	**0.229**	**5.329**	**3.490**
Panagrolaimus	Tripyla	**-0.945**	**-0.420**	**5.028**	**4.727**
Panagrolaimus	Hypoaspis	**-0.945**	**0.334**	**5.028**	**3.189**
Panagrolaimus	Lysigamasus	**-0.945**	**0.407**	**5.028**	**3.189**
Panagrolaimus	Aporcelaimellus	**-0.945**	**0.548**	**5.028**	**5.125**
Panagrolaimus	Dorylaimoidea	**-0.945**	**-0.604**	**5.028**	**5.204**
Panagrolaimus	Mesodorylaimus	**-0.945**	**-0.277**	**5.028**	**4.426**
Panagrolaimus	Prodorylaimus	**-0.945**	**-0.836**	**5.028**	**5.028**
Panagrolaimus	Pungentus	**-0.945**	**0.263**	**5.028**	**4.426**
Panagrolaimus	Qudsianematidae	**-0.945**	**-0.207**	**5.028**	**4.727**
Panagrolaimus	Thornematidae	**-0.945**	**-0.470**	**5.028**	**4.903**
Panagrolaimus	Eupodes	**-0.945**	**0.005**	**5.028**	**4.393**
Panagrolaimus	Mesostigmata	**-0.945**	**-0.411**	**5.028**	**3.189**
Panagrolaimus	Oribatida	**-0.945**	**-0.411**	**5.028**	**3.189**
Panagrolaimus	Scheloribates	**-0.945**	**0.202**	**5.028**	**3.666**
Panagrolaimus	Stigmaeidae	**-0.945**	**0.229**	**5.028**	**3.490**
Plectus	Tripyla	**-0.583**	**-0.420**	**5.572**	**4.727**
Plectus	Hypoaspis	**-0.583**	**0.334**	**5.572**	**3.189**
Plectus	Lysigamasus	**-0.583**	**0.407**	**5.572**	**3.189**
Plectus	Aporcelaimellus	**-0.583**	**0.548**	**5.572**	**5.125**
Plectus	Dorylaimoidea	**-0.583**	**-0.604**	**5.572**	**5.204**
Plectus	Mesodorylaimus	**-0.583**	**-0.277**	**5.572**	**4.426**
Plectus	Prodorylaimus	**-0.583**	**-0.836**	**5.572**	**5.028**
Plectus	Pungentus	**-0.583**	**0.263**	**5.572**	**4.426**
Plectus	Qudsianematidae	**-0.583**	**-0.207**	**5.572**	**4.727**
Plectus	Thornematidae	**-0.583**	**-0.470**	**5.572**	**4.903**
Plectus	Eupodes	**-0.583**	**0.005**	**5.572**	**4.393**
Plectus	Mesostigmata	**-0.583**	**-0.411**	**5.572**	**3.189**
Plectus	Oribatida	**-0.583**	**-0.411**	**5.572**	**3.189**
Plectus	Scheloribates	**-0.583**	**0.202**	**5.572**	**3.666**
Plectus	Stigmaeidae	**-0.583**	**0.229**	**5.572**	**3.490**
Rhabditidae	Tripyla	**-0.692**	**-0.420**	**4.727**	**4.727**
Rhabditidae	Hypoaspis	**-0.692**	**0.334**	**4.727**	**3.189**
Rhabditidae	Lysigamasus	**-0.692**	**0.407**	**4.727**	**3.189**
Rhabditidae	Aporcelaimellus	**-0.692**	**0.548**	**4.727**	**5.125**
Rhabditidae	Dorylaimoidea	**-0.692**	**-0.604**	**4.727**	**5.204**
Rhabditidae	Mesodorylaimus	**-0.692**	**-0.277**	**4.727**	**4.426**
Rhabditidae	Prodorylaimus	**-0.692**	**-0.836**	**4.727**	**5.028**
Rhabditidae	Pungentus	**-0.692**	**0.263**	**4.727**	**4.426**
Rhabditidae	Qudsianematidae	**-0.692**	**-0.207**	**4.727**	**4.727**
Rhabditidae	Thornematidae	**-0.692**	**-0.470**	**4.727**	**4.903**
Rhabditidae	Eupodes	**-0.692**	**0.005**	**4.727**	**4.393**

Rhabditidae	Mesostigmata	**-0.692**	**-0.411**	**4.727**	**3.189**
Rhabditidae	Oribatida	**-0.692**	**-0.411**	**4.727**	**3.189**
Rhabditidae	Scheloribates	**-0.692**	**0.202**	**4.727**	**3.666**
Rhabditidae	Stigmaeidae	**-0.692**	**0.229**	**4.727**	**3.490**
Teratocephalus	Tripyla	**-1.630**	**-0.420**	**4.903**	**4.727**
Teratocephalus	Hypoaspis	**-1.630**	**0.334**	**4.903**	**3.189**
Teratocephalus	Lysigamasus	**-1.630**	**0.407**	**4.903**	**3.189**
Teratocephalus	Aporcelaimellus	**-1.630**	**0.548**	**4.903**	**5.125**
Teratocephalus	Dorylaimoidea	**-1.630**	**-0.604**	**4.903**	**5.204**
Teratocephalus	Mesodorylaimus	**-1.630**	**-0.277**	**4.903**	**4.426**
Teratocephalus	Prodorylaimus	**-1.630**	**-0.836**	**4.903**	**5.028**
Teratocephalus	Pungentus	**-1.630**	**0.263**	**4.903**	**4.426**
Teratocephalus	Qudsianematidae	**-1.630**	**-0.207**	**4.903**	**4.727**
Teratocephalus	Thornematidae	**-1.630**	**-0.470**	**4.903**	**4.903**
Teratocephalus	Eupodes	**-1.630**	**0.005**	**4.903**	**4.393**
Teratocephalus	Mesostigmata	**-1.630**	**-0.411**	**4.903**	**3.189**
Teratocephalus	Oribatida	**-1.630**	**-0.411**	**4.903**	**3.189**
Teratocephalus	Scheloribates	**-1.630**	**0.202**	**4.903**	**3.666**
Teratocephalus	Stigmaeidae	**-1.630**	**0.229**	**4.903**	**3.490**
Enchytraeus	Hypoaspis	**1.014**	**0.334**	**3.302**	**3.189**
Enchytraeus	Lysigamasus	**1.014**	**0.407**	**3.302**	**3.189**
Enchytraeus	Aporcelaimellus	**1.014**	**0.548**	**3.302**	**5.125**
Enchytraeus	Dorylaimoidea	**1.014**	**-0.604**	**3.302**	**5.204**
Enchytraeus	Mesodorylaimus	**1.014**	**-0.277**	**3.302**	**4.426**
Enchytraeus	Prodorylaimus	**1.014**	**-0.836**	**3.302**	**5.028**
Enchytraeus	Pungentus	**1.014**	**0.263**	**3.302**	**4.426**
Enchytraeus	Qudsianematidae	**1.014**	**-0.207**	**3.302**	**4.727**
Enchytraeus	Thornematidae	**1.014**	**-0.470**	**3.302**	**4.903**
Enchytraeus	Eupodes	**1.014**	**0.005**	**3.302**	**4.393**
Enchytraeus	Mesostigmata	**1.014**	**-0.411**	**3.302**	**3.189**
Enchytraeus	Oribatida	**1.014**	**-0.411**	**3.302**	**3.189**
Enchytraeus	Scheloribates	**1.014**	**0.202**	**3.302**	**3.666**
Enchytraeus	Stigmaeidae	**1.014**	**0.229**	**3.302**	**3.490**
Eubacteria	Acrobeles	**-6.582**	**-0.721**	**12.958**	**4.426**
Eubacteria	Acrobeloides	**-6.582**	**-1.171**	**12.958**	**5.204**
Eubacteria	Alaimus	**-6.582**	**-0.858**	**12.958**	**4.426**
Eubacteria	Anaplectus	**-6.582**	**-0.519**	**12.958**	**4.727**
Eubacteria	Bunonema	**-6.582**	**-0.676**	**12.958**	**4.426**
Eubacteria	Eucephalobus	**-6.582**	**-0.855**	**12.958**	**5.380**
Eubacteria	Eumonhystera	**-6.582**	**-0.613**	**12.958**	**4.426**
Eubacteria	Metateratocephalus	**-6.582**	**-1.506**	**12.958**	**5.329**
Eubacteria	Panagrolaimus	**-6.582**	**-0.945**	**12.958**	**5.028**
Eubacteria	Plectus	**-6.582**	**-0.583**	**12.958**	**5.572**
Eubacteria	Rhabditidae	**-6.582**	**-0.692**	**12.958**	**4.727**
Eubacteria	Teratocephalus	**-6.582**	**-1.630**	**12.958**	**4.903**
Eubacteria	Enchytraeus	**-6.582**	**1.014**	**12.958**	**3.302**
Eubacteria	Dauerlarvae	**-6.582**	**-0.804**	**12.958**	**4.426**
Eubacteria	Henlea	**-6.582**	**1.750**	**12.958**	**2.916**
Eubacteria	Marionina	**-6.582**	**0.806**	**12.958**	**2.812**
Dauerlarvae	Tripyla	**-0.804**	**-0.420**	**4.426**	**4.727**
Dauerlarvae	Aporcelaimellus	**-0.804**	**0.548**	**4.426**	**5.125**
Dauerlarvae	Dorylaimoidea	**-0.804**	**-0.604**	**4.426**	**5.204**
Dauerlarvae	Mesodorylaimus	**-0.804**	**-0.277**	**4.426**	**4.426**
Dauerlarvae	Prodorylaimus	**-0.804**	**-0.836**	**4.426**	**5.028**
Dauerlarvae	Pungentus	**-0.804**	**0.263**	**4.426**	**4.426**
Dauerlarvae	Qudsianematidae	**-0.804**	**-0.207**	**4.426**	**4.727**
Dauerlarvae	Thornematidae	**-0.804**	**-0.470**	**4.426**	**4.903**
Dauerlarvae	Eupodes	**-0.804**	**0.005**	**4.426**	**4.393**
Dauerlarvae	Mesostigmata	**-0.804**	**-0.411**	**4.426**	**3.189**
Dauerlarvae	Oribatida	**-0.804**	**-0.411**	**4.426**	**3.189**
Dauerlarvae	Scheloribates	**-0.804**	**0.202**	**4.426**	**3.666**
Dauerlarvae	Stigmaeidae	**-0.804**	**0.229**	**4.426**	**3.490**
Henlea	Hypoaspis	**1.750**	**0.334**	**2.916**	**3.189**
Henlea	Lysigamasus	**1.750**	**0.407**	**2.916**	**3.189**
Henlea	Aporcelaimellus	**1.750**	**0.548**	**2.916**	**5.125**
Henlea	Dorylaimoidea	**1.750**	**-0.604**	**2.916**	**5.204**
Henlea	Mesodorylaimus	**1.750**	**-0.277**	**2.916**	**4.426**
Henlea	Prodorylaimus	**1.750**	**-0.836**	**2.916**	**5.028**
Henlea	Pungentus	**1.750**	**0.263**	**2.916**	**4.426**
Henlea	Qudsianematidae	**1.750**	**-0.207**	**2.916**	**4.727**

Henlea	Thornematidae	**1.750**	**-0.470**	**2.916**	**4.903**
Henlea	Eupodes	**1.750**	**0.005**	**2.916**	**4.393**
Henlea	Mesostigmata	**1.750**	**-0.411**	**2.916**	**3.189**
Henlea	Oribatida	**1.750**	**-0.411**	**2.916**	**3.189**
Henlea	Scheloribates	**1.750**	**0.202**	**2.916**	**3.666**
Henlea	Stigmaeidae	**1.750**	**0.229**	**2.916**	**3.490**
Marionina	Hypoaspis	**0.806**	**0.334**	**2.812**	**3.189**
Marionina	Lysigamasus	**0.806**	**0.407**	**2.812**	**3.189**
Marionina	Aporcelaimellus	**0.806**	**0.548**	**2.812**	**5.125**
Marionina	Dorylaimoidea	**0.806**	**-0.604**	**2.812**	**5.204**
Marionina	Mesodorylaimus	**0.806**	**-0.277**	**2.812**	**4.426**
Marionina	Prodorylaimus	**0.806**	**-0.836**	**2.812**	**5.028**
Marionina	Pungentus	**0.806**	**0.263**	**2.812**	**4.426**
Marionina	Qudsianematidae	**0.806**	**-0.207**	**2.812**	**4.727**
Marionina	Thornematidae	**0.806**	**-0.470**	**2.812**	**4.903**
Marionina	Eupodes	**0.806**	**0.005**	**2.812**	**4.393**
Marionina	Mesostigmata	**0.806**	**-0.411**	**2.812**	**3.189**
Marionina	Oribatida	**0.806**	**-0.411**	**2.812**	**3.189**
Marionina	Scheloribates	**0.806**	**0.202**	**2.812**	**3.666**
Marionina	Stigmaeidae	**0.806**	**0.229**	**2.812**	**3.490**
Hyphae and hair roots	Aglenchus	**6.224**	**-1.053**	**0.000**	**5.505**
Hyphae and hair roots	Criconematidae	**6.224**	**-0.753**	**0.000**	**4.426**
Hyphae and hair roots	Dolichodoridae	**6.224**	**-0.885**	**0.000**	**4.727**
Hyphae and hair roots	Helicotylenchus	**6.224**	**-0.792**	**0.000**	**4.727**
Hyphae and hair roots	Malenchus	**6.224**	**-1.330**	**0.000**	**4.426**
Hyphae and hair roots	Paratylenchus	**6.224**	**-1.244**	**0.000**	**4.426**
Hyphae and hair roots	Pratylenchus	**6.224**	**-1.226**	**0.000**	**4.426**
Hyphae and hair roots	Trichodorus	**6.224**	**-0.744**	**0.000**	**4.426**
Hyphae and hair roots	Achipteria	**6.224**	**0.341**	**0.000**	**3.189**
Hyphae and hair roots	Galumna	**6.224**	**0.474**	**0.000**	**3.189**
Hyphae and hair roots	Platynothrus	**6.224**	**0.710**	**0.000**	**4.034**
Hyphae and hair roots	Tydeidae	**6.224**	**-0.608**	**0.000**	**3.967**
Hyphae and hair roots	Sminthuridae	**6.224**	**-0.608**	**0.000**	**3.490**
Hyphae and hair roots	Sminthurinus	**6.224**	**0.618**	**0.000**	**3.666**
Hyphae and hair roots	Aphelenchoides	**6.224**	**-1.496**	**0.000**	**5.426**
Hyphae and hair roots	Tylenchidae	**6.224**	**-1.360**	**0.000**	**6.039**
Hyphae and hair roots	Liochthonius	**6.224**	**-0.653**	**0.000**	**3.791**
Hyphae and hair roots	Medioppia	**6.224**	**-0.235**	**0.000**	**3.490**
Hyphae and hair roots	Microtydeus	**6.224**	**-0.863**	**0.000**	**3.189**
Hyphae and hair roots	Pygmephorus	**6.224**	**-0.376**	**0.000**	**3.490**
Hyphae and hair roots	Tectocepheus	**6.224**	**-0.220**	**0.000**	**3.666**
Hyphae and hair roots	Trichoribates	**6.224**	**0.474**	**0.000**	**3.666**
Hyphae and hair roots	Brachystomella	**6.224**	**0.977**	**0.000**	**3.189**
Hyphae and hair roots	Lepidocyrtus	**6.224**	**1.231**	**0.000**	**3.967**
Hyphae and hair roots	Achaeta	**6.224**	**1.104**	**0.000**	**2.373**
Hyphae and hair roots	Cognettia	**6.224**	**1.682**	**0.000**	**3.891**
Hyphae and hair roots	Fridericia	**6.224**	**2.856**	**0.000**	**3.248**
Hyphae and hair roots	Aporcelaimellus	**6.224**	**0.548**	**0.000**	**5.125**
Hyphae and hair roots	Dorylaimoidea	**6.224**	**-0.604**	**0.000**	**5.204**
Hyphae and hair roots	Mesodorylaimus	**6.224**	**-0.277**	**0.000**	**4.426**
Hyphae and hair roots	Prodorylaimus	**6.224**	**-0.836**	**0.000**	**5.028**
Hyphae and hair roots	Pungentus	**6.224**	**0.263**	**0.000**	**4.426**
Hyphae and hair roots	Qudsianematidae	**6.224**	**-0.207**	**0.000**	**4.727**
Hyphae and hair roots	Thornematidae	**6.224**	**-0.470**	**0.000**	**4.903**
Hyphae and hair roots	Eupodes	**6.224**	**0.005**	**0.000**	**4.393**
Hyphae and hair roots	Mesostigmata	**6.224**	**-0.411**	**0.000**	**3.189**
Hyphae and hair roots	Oribatida	**6.224**	**-0.411**	**0.000**	**3.189**
Hyphae and hair roots	Scheloribates	**6.224**	**0.202**	**0.000**	**3.666**
Hyphae and hair roots	Stigmaeidae	**6.224**	**0.229**	**0.000**	**3.490**
Tripyla	Hypoaspis	**-0.420**	**0.334**	**4.727**	**3.189**
Tripyla	Lysigamasus	**-0.420**	**0.407**	**4.727**	**3.189**
Tripyla	Aporcelaimellus	**-0.420**	**0.548**	**4.727**	**5.125**
Tripyla	Dorylaimoidea	**-0.420**	**-0.604**	**4.727**	**5.204**
Tripyla	Mesodorylaimus	**-0.420**	**-0.277**	**4.727**	**4.426**
Tripyla	Prodorylaimus	**-0.420**	**-0.836**	**4.727**	**5.028**
Tripyla	Pungentus	**-0.420**	**0.263**	**4.727**	**4.426**
Tripyla	Qudsianematidae	**-0.420**	**-0.207**	**4.727**	**4.727**
Tripyla	Thornematidae	**-0.420**	**-0.470**	**4.727**	**4.903**
Tripyla	Eupodes	**-0.420**	**0.005**	**4.727**	**4.393**
Tripyla	Mesostigmata	**-0.420**	**-0.411**	**4.727**	**3.189**

Tripyla	Oribatida	**-0.420**	**-0.411**	**4.727**	**3.189**
Tripyla	Scheloribates	**-0.420**	**0.202**	**4.727**	**3.666**
Tripyla	Stigmaeidae	**-0.420**	**0.229**	**4.727**	**3.490**
Hypoaspis	Aporcelaimellus	**0.334**	**0.548**	**3.189**	**5.125**
Hypoaspis	Dorylaimoidea	**0.334**	**-0.604**	**3.189**	**5.204**
Hypoaspis	Mesodorylaimus	**0.334**	**-0.277**	**3.189**	**4.426**
Hypoaspis	Prodorylaimus	**0.334**	**-0.836**	**3.189**	**5.028**
Hypoaspis	Pungentus	**0.334**	**0.263**	**3.189**	**4.426**
Hypoaspis	Qudsianematidae	**0.334**	**-0.207**	**3.189**	**4.727**
Hypoaspis	Thornematidae	**0.334**	**-0.470**	**3.189**	**4.903**
Hypoaspis	Eupodes	**0.334**	**0.005**	**3.189**	**4.393**
Hypoaspis	Mesostigmata	**0.334**	**-0.411**	**3.189**	**3.189**
Hypoaspis	Oribatida	**0.334**	**-0.411**	**3.189**	**3.189**
Hypoaspis	Scheloribates	**0.334**	**0.202**	**3.189**	**3.666**
Hypoaspis	Stigmaeidae	**0.334**	**0.229**	**3.189**	**3.490**
Hypoaspis	Trombidiidae	**0.334**	**1.787**	**3.189**	**3.490**
Lysigamasus	Aporcelaimellus	**0.407**	**0.548**	**3.189**	**5.125**
Lysigamasus	Dorylaimoidea	**0.407**	**-0.604**	**3.189**	**5.204**
Lysigamasus	Mesodorylaimus	**0.407**	**-0.277**	**3.189**	**4.426**
Lysigamasus	Prodorylaimus	**0.407**	**-0.836**	**3.189**	**5.028**
Lysigamasus	Pungentus	**0.407**	**0.263**	**3.189**	**4.426**
Lysigamasus	Qudsianematidae	**0.407**	**-0.207**	**3.189**	**4.727**
Lysigamasus	Thornematidae	**0.407**	**-0.470**	**3.189**	**4.903**
Lysigamasus	Eupodes	**0.407**	**0.005**	**3.189**	**4.393**
Lysigamasus	Mesostigmata	**0.407**	**-0.411**	**3.189**	**3.189**
Lysigamasus	Oribatida	**0.407**	**-0.411**	**3.189**	**3.189**
Lysigamasus	Scheloribates	**0.407**	**0.202**	**3.189**	**3.666**
Lysigamasus	Stigmaeidae	**0.407**	**0.229**	**3.189**	**3.490**
Lysigamasus	Trombidiidae	**0.407**	**1.787**	**3.189**	**3.490**
Aporcelaimellus	Tripyla	**0.548**	**-0.420**	**5.125**	**4.727**
Aporcelaimellus	Hypoaspis	**0.548**	**0.334**	**5.125**	**3.189**
Aporcelaimellus	Lysigamasus	**0.548**	**0.407**	**5.125**	**3.189**
Aporcelaimellus	Aporcelaimellus	**0.548**	**0.548**	**5.125**	**5.125**
Aporcelaimellus	Dorylaimoidea	**0.548**	**-0.604**	**5.125**	**5.204**
Aporcelaimellus	Mesodorylaimus	**0.548**	**-0.277**	**5.125**	**4.426**
Aporcelaimellus	Prodorylaimus	**0.548**	**-0.836**	**5.125**	**5.028**
Aporcelaimellus	Pungentus	**0.548**	**0.263**	**5.125**	**4.426**
Aporcelaimellus	Qudsianematidae	**0.548**	**-0.207**	**5.125**	**4.727**
Aporcelaimellus	Thornematidae	**0.548**	**-0.470**	**5.125**	**4.903**
Aporcelaimellus	Eupodes	**0.548**	**0.005**	**5.125**	**4.393**
Aporcelaimellus	Mesostigmata	**0.548**	**-0.411**	**5.125**	**3.189**
Aporcelaimellus	Oribatida	**0.548**	**-0.411**	**5.125**	**3.189**
Aporcelaimellus	Scheloribates	**0.548**	**0.202**	**5.125**	**3.666**
Aporcelaimellus	Stigmaeidae	**0.548**	**0.229**	**5.125**	**3.490**
Dorylaimoidea	Tripyla	**-0.604**	**-0.420**	**5.204**	**4.727**
Dorylaimoidea	Hypoaspis	**-0.604**	**0.334**	**5.204**	**3.189**
Dorylaimoidea	Lysigamasus	**-0.604**	**0.407**	**5.204**	**3.189**
Dorylaimoidea	Aporcelaimellus	**-0.604**	**0.548**	**5.204**	**5.125**
Dorylaimoidea	Dorylaimoidea	**-0.604**	**-0.604**	**5.204**	**5.204**
Dorylaimoidea	Mesodorylaimus	**-0.604**	**-0.277**	**5.204**	**4.426**
Dorylaimoidea	Prodorylaimus	**-0.604**	**-0.836**	**5.204**	**5.028**
Dorylaimoidea	Pungentus	**-0.604**	**0.263**	**5.204**	**4.426**
Dorylaimoidea	Qudsianematidae	**-0.604**	**-0.207**	**5.204**	**4.727**
Dorylaimoidea	Thornematidae	**-0.604**	**-0.470**	**5.204**	**4.903**
Dorylaimoidea	Eupodes	**-0.604**	**0.005**	**5.204**	**4.393**
Dorylaimoidea	Mesostigmata	**-0.604**	**-0.411**	**5.204**	**3.189**
Dorylaimoidea	Oribatida	**-0.604**	**-0.411**	**5.204**	**3.189**
Dorylaimoidea	Scheloribates	**-0.604**	**0.202**	**5.204**	**3.666**
Dorylaimoidea	Stigmaeidae	**-0.604**	**0.229**	**5.204**	**3.490**
Mesodorylaimus	Tripyla	**-0.277**	**-0.420**	**4.426**	**4.727**
Mesodorylaimus	Hypoaspis	**-0.277**	**0.334**	**4.426**	**3.189**
Mesodorylaimus	Lysigamasus	**-0.277**	**0.407**	**4.426**	**3.189**
Mesodorylaimus	Aporcelaimellus	**-0.277**	**0.548**	**4.426**	**5.125**
Mesodorylaimus	Dorylaimoidea	**-0.277**	**-0.604**	**4.426**	**5.204**
Mesodorylaimus	Mesodorylaimus	**-0.277**	**-0.277**	**4.426**	**4.426**
Mesodorylaimus	Prodorylaimus	**-0.277**	**-0.836**	**4.426**	**5.028**
Mesodorylaimus	Pungentus	**-0.277**	**0.263**	**4.426**	**4.426**
Mesodorylaimus	Qudsianematidae	**-0.277**	**-0.207**	**4.426**	**4.727**
Mesodorylaimus	Thornematidae	**-0.277**	**-0.470**	**4.426**	**4.903**
Mesodorylaimus	Eupodes	**-0.277**	**0.005**	**4.426**	**4.393**

Mesodorylaimus	Mesostigmata	**-0.277**	**-0.411**	**4.426**	**3.189**
Mesodorylaimus	Oribatida	**-0.277**	**-0.411**	**4.426**	**3.189**
Mesodorylaimus	Scheloribates	**-0.277**	**0.202**	**4.426**	**3.666**
Mesodorylaimus	Stigmaeidae	**-0.277**	**0.229**	**4.426**	**3.490**
Prodorylaimus	Tripyla	**-0.836**	**-0.420**	**5.028**	**4.727**
Prodorylaimus	Hypoaspis	**-0.836**	**0.334**	**5.028**	**3.189**
Prodorylaimus	Lysigamasus	**-0.836**	**0.407**	**5.028**	**3.189**
Prodorylaimus	Aporcelaimellus	**-0.836**	**0.548**	**5.028**	**5.125**
Prodorylaimus	Dorylaimoidea	**-0.836**	**-0.604**	**5.028**	**5.204**
Prodorylaimus	Mesodorylaimus	**-0.836**	**-0.277**	**5.028**	**4.426**
Prodorylaimus	Prodorylaimus	**-0.836**	**-0.836**	**5.028**	**5.028**
Prodorylaimus	Pungentus	**-0.836**	**0.263**	**5.028**	**4.426**
Prodorylaimus	Qudsianematidae	**-0.836**	**-0.207**	**5.028**	**4.727**
Prodorylaimus	Thornematidae	**-0.836**	**-0.470**	**5.028**	**4.903**
Prodorylaimus	Eupodes	**-0.836**	**0.005**	**5.028**	**4.393**
Prodorylaimus	Mesostigmata	**-0.836**	**-0.411**	**5.028**	**3.189**
Prodorylaimus	Oribatida	**-0.836**	**-0.411**	**5.028**	**3.189**
Prodorylaimus	Scheloribates	**-0.836**	**0.202**	**5.028**	**3.666**
Prodorylaimus	Stigmaeidae	**-0.836**	**0.229**	**5.028**	**3.490**
Pungentus	Tripyla	**0.263**	**-0.420**	**4.426**	**4.727**
Pungentus	Hypoaspis	**0.263**	**0.334**	**4.426**	**3.189**
Pungentus	Lysigamasus	**0.263**	**0.407**	**4.426**	**3.189**
Pungentus	Aporcelaimellus	**0.263**	**0.548**	**4.426**	**5.125**
Pungentus	Dorylaimoidea	**0.263**	**-0.604**	**4.426**	**5.204**
Pungentus	Mesodorylaimus	**0.263**	**-0.277**	**4.426**	**4.426**
Pungentus	Prodorylaimus	**0.263**	**-0.836**	**4.426**	**5.028**
Pungentus	Pungentus	**0.263**	**0.263**	**4.426**	**4.426**
Pungentus	Qudsianematidae	**0.263**	**-0.207**	**4.426**	**4.727**
Pungentus	Thornematidae	**0.263**	**-0.470**	**4.426**	**4.903**
Pungentus	Eupodes	**0.263**	**0.005**	**4.426**	**4.393**
Pungentus	Mesostigmata	**0.263**	**-0.411**	**4.426**	**3.189**
Pungentus	Oribatida	**0.263**	**-0.411**	**4.426**	**3.189**
Pungentus	Scheloribates	**0.263**	**0.202**	**4.426**	**3.666**
Pungentus	Stigmaeidae	**0.263**	**0.229**	**4.426**	**3.490**
Qudsianematidae	Tripyla	**-0.207**	**-0.420**	**4.727**	**4.727**
Qudsianematidae	Hypoaspis	**-0.207**	**0.334**	**4.727**	**3.189**
Qudsianematidae	Lysigamasus	**-0.207**	**0.407**	**4.727**	**3.189**
Qudsianematidae	Aporcelaimellus	**-0.207**	**0.548**	**4.727**	**5.125**
Qudsianematidae	Dorylaimoidea	**-0.207**	**-0.604**	**4.727**	**5.204**
Qudsianematidae	Mesodorylaimus	**-0.207**	**-0.277**	**4.727**	**4.426**
Qudsianematidae	Prodorylaimus	**-0.207**	**-0.836**	**4.727**	**5.028**
Qudsianematidae	Pungentus	**-0.207**	**0.263**	**4.727**	**4.426**
Qudsianematidae	Qudsianematidae	**-0.207**	**-0.207**	**4.727**	**4.727**
Qudsianematidae	Thornematidae	**-0.207**	**-0.470**	**4.727**	**4.903**
Qudsianematidae	Eupodes	**-0.207**	**0.005**	**4.727**	**4.393**
Qudsianematidae	Mesostigmata	**-0.207**	**-0.411**	**4.727**	**3.189**
Qudsianematidae	Oribatida	**-0.207**	**-0.411**	**4.727**	**3.189**
Qudsianematidae	Scheloribates	**-0.207**	**0.202**	**4.727**	**3.666**
Qudsianematidae	Stigmaeidae	**-0.207**	**0.229**	**4.727**	**3.490**
Thornematidae	Tripyla	**-0.470**	**-0.420**	**4.903**	**4.727**
Thornematidae	Hypoaspis	**-0.470**	**0.334**	**4.903**	**3.189**
Thornematidae	Lysigamasus	**-0.470**	**0.407**	**4.903**	**3.189**
Thornematidae	Aporcelaimellus	**-0.470**	**0.548**	**4.903**	**5.125**
Thornematidae	Dorylaimoidea	**-0.470**	**-0.604**	**4.903**	**5.204**
Thornematidae	Mesodorylaimus	**-0.470**	**-0.277**	**4.903**	**4.426**
Thornematidae	Prodorylaimus	**-0.470**	**-0.836**	**4.903**	**5.028**
Thornematidae	Pungentus	**-0.470**	**0.263**	**4.903**	**4.426**
Thornematidae	Qudsianematidae	**-0.470**	**-0.207**	**4.903**	**4.727**
Thornematidae	Thornematidae	**-0.470**	**-0.470**	**4.903**	**4.903**
Thornematidae	Eupodes	**-0.470**	**0.005**	**4.903**	**4.393**
Thornematidae	Mesostigmata	**-0.470**	**-0.411**	**4.903**	**3.189**
Thornematidae	Oribatida	**-0.470**	**-0.411**	**4.903**	**3.189**
Thornematidae	Scheloribates	**-0.470**	**0.202**	**4.903**	**3.666**
Thornematidae	Stigmaeidae	**-0.470**	**0.229**	**4.903**	**3.490**
Eupodes	Hypoaspis	**0.005**	**0.334**	**4.393**	**3.189**
Eupodes	Lysigamasus	**0.005**	**0.407**	**4.393**	**3.189**
Eupodes	Aporcelaimellus	**0.005**	**0.548**	**4.393**	**5.125**
Eupodes	Dorylaimoidea	**0.005**	**-0.604**	**4.393**	**5.204**
Eupodes	Mesodorylaimus	**0.005**	**-0.277**	**4.393**	**4.426**
Eupodes	Prodorylaimus	**0.005**	**-0.836**	**4.393**	**5.028**

Eupodes	Pungentus	**0.005**	**0.263**	**4.393**	**4.426**
Eupodes	Qudsianematidae	**0.005**	**-0.207**	**4.393**	**4.727**
Eupodes	Thornematidae	**0.005**	**-0.470**	**4.393**	**4.903**
Eupodes	Eupodes	**0.005**	**0.005**	**4.393**	**4.393**
Eupodes	Mesostigmata	**0.005**	**-0.411**	**4.393**	**3.189**
Eupodes	Oribatida	**0.005**	**-0.411**	**4.393**	**3.189**
Eupodes	Scheloribates	**0.005**	**0.202**	**4.393**	**3.666**
Eupodes	Stigmaeidae	**0.005**	**0.229**	**4.393**	**3.490**
Mesostigmata	Hypoaspis	**-0.411**	**0.334**	**3.189**	**3.189**
Mesostigmata	Lysigamasus	**-0.411**	**0.407**	**3.189**	**3.189**
Mesostigmata	Aporcelaimellus	**-0.411**	**0.548**	**3.189**	**5.125**
Mesostigmata	Dorylaimoidea	**-0.411**	**-0.604**	**3.189**	**5.204**
Mesostigmata	Mesodorylaimus	**-0.411**	**-0.277**	**3.189**	**4.426**
Mesostigmata	Prodorylaimus	**-0.411**	**-0.836**	**3.189**	**5.028**
Mesostigmata	Pungentus	**-0.411**	**0.263**	**3.189**	**4.426**
Mesostigmata	Qudsianematidae	**-0.411**	**-0.207**	**3.189**	**4.727**
Mesostigmata	Thornematidae	**-0.411**	**-0.470**	**3.189**	**4.903**
Mesostigmata	Eupodes	**-0.411**	**0.005**	**3.189**	**4.393**
Mesostigmata	Mesostigmata	**-0.411**	**-0.411**	**3.189**	**3.189**
Mesostigmata	Oribatida	**-0.411**	**-0.411**	**3.189**	**3.189**
Mesostigmata	Scheloribates	**-0.411**	**0.202**	**3.189**	**3.666**
Mesostigmata	Stigmaeidae	**-0.411**	**0.229**	**3.189**	**3.490**
Oribatida	Hypoaspis	**-0.411**	**0.334**	**3.189**	**3.189**
Oribatida	Lysigamasus	**-0.411**	**0.407**	**3.189**	**3.189**
Oribatida	Aporcelaimellus	**-0.411**	**0.548**	**3.189**	**5.125**
Oribatida	Dorylaimoidea	**-0.411**	**-0.604**	**3.189**	**5.204**
Oribatida	Mesodorylaimus	**-0.411**	**-0.277**	**3.189**	**4.426**
Oribatida	Prodorylaimus	**-0.411**	**-0.836**	**3.189**	**5.028**
Oribatida	Pungentus	**-0.411**	**0.263**	**3.189**	**4.426**
Oribatida	Qudsianematidae	**-0.411**	**-0.207**	**3.189**	**4.727**
Oribatida	Thornematidae	**-0.411**	**-0.470**	**3.189**	**4.903**
Oribatida	Eupodes	**-0.411**	**0.005**	**3.189**	**4.393**
Oribatida	Mesostigmata	**-0.411**	**-0.411**	**3.189**	**3.189**
Oribatida	Oribatida	**-0.411**	**-0.411**	**3.189**	**3.189**
Oribatida	Scheloribates	**-0.411**	**0.202**	**3.189**	**3.666**
Oribatida	Stigmaeidae	**-0.411**	**0.229**	**3.189**	**3.490**
Scheloribates	Hypoaspis	**0.202**	**0.334**	**3.666**	**3.189**
Scheloribates	Lysigamasus	**0.202**	**0.407**	**3.666**	**3.189**
Scheloribates	Aporcelaimellus	**0.202**	**0.548**	**3.666**	**5.125**
Scheloribates	Dorylaimoidea	**0.202**	**-0.604**	**3.666**	**5.204**
Scheloribates	Mesodorylaimus	**0.202**	**-0.277**	**3.666**	**4.426**
Scheloribates	Prodorylaimus	**0.202**	**-0.836**	**3.666**	**5.028**
Scheloribates	Pungentus	**0.202**	**0.263**	**3.666**	**4.426**
Scheloribates	Qudsianematidae	**0.202**	**-0.207**	**3.666**	**4.727**
Scheloribates	Thornematidae	**0.202**	**-0.470**	**3.666**	**4.903**
Scheloribates	Eupodes	**0.202**	**0.005**	**3.666**	**4.393**
Scheloribates	Mesostigmata	**0.202**	**-0.411**	**3.666**	**3.189**
Scheloribates	Oribatida	**0.202**	**-0.411**	**3.666**	**3.189**
Scheloribates	Scheloribates	**0.202**	**0.202**	**3.666**	**3.666**
Scheloribates	Stigmaeidae	**0.202**	**0.229**	**3.666**	**3.490**
Stigmaeidae	Hypoaspis	**0.229**	**0.334**	**3.490**	**3.189**
Stigmaeidae	Lysigamasus	**0.229**	**0.407**	**3.490**	**3.189**
Stigmaeidae	Aporcelaimellus	**0.229**	**0.548**	**3.490**	**5.125**
Stigmaeidae	Dorylaimoidea	**0.229**	**-0.604**	**3.490**	**5.204**
Stigmaeidae	Mesodorylaimus	**0.229**	**-0.277**	**3.490**	**4.426**
Stigmaeidae	Prodorylaimus	**0.229**	**-0.836**	**3.490**	**5.028**
Stigmaeidae	Pungentus	**0.229**	**0.263**	**3.490**	**4.426**
Stigmaeidae	Qudsianematidae	**0.229**	**-0.207**	**3.490**	**4.727**
Stigmaeidae	Thornematidae	**0.229**	**-0.470**	**3.490**	**4.903**
Stigmaeidae	Eupodes	**0.229**	**0.005**	**3.490**	**4.393**
Stigmaeidae	Mesostigmata	**0.229**	**-0.411**	**3.490**	**3.189**
Stigmaeidae	Oribatida	**0.229**	**-0.411**	**3.490**	**3.189**
Stigmaeidae	Scheloribates	**0.229**	**0.202**	**3.490**	**3.666**
Stigmaeidae	Stigmaeidae	**0.229**	**0.229**	**3.490**	**3.490**
Trombidiidae	Eupodes	**1.787**	**0.005**	**3.490**	**4.393**
Trombidiidae	Mesostigmata	**1.787**	**-0.411**	**3.490**	**3.189**
Trombidiidae	Oribatida	**1.787**	**-0.411**	**3.490**	**3.189**
Trombidiidae	Scheloribates	**1.787**	**0.202**	**3.490**	**3.666**
Trombidiidae	Stigmaeidae	**1.787**	**0.229**	**3.490**	**3.490**

Resource	Consumer	Mres	Mconsumer	Nres	Nconsumer
Aglenchus	Mononchidae	**-1.053**	**-0.827**	**4.731**	**4.430**
Aglenchus	Mylonchulus	**-1.053**	**-0.005**	**4.731**	**4.430**
Aglenchus	Lysigamasus	**-1.053**	**0.407**	**4.731**	**3.564**
Aglenchus	Aporcelaimellus	**-1.053**	**0.548**	**4.731**	**5.129**
Aglenchus	Dorylaimoidea	**-1.053**	**-0.604**	**4.731**	**5.544**
Aglenchus	Eudorylaimus	**-1.053**	**-0.166**	**4.731**	**4.731**
Aglenchus	Prodorylaimus	**-1.053**	**-0.836**	**4.731**	**4.731**
Aglenchus	Qudsianematidae	**-1.053**	**-0.207**	**4.731**	**4.430**
Aglenchus	Thornematidae	**-1.053**	**-0.470**	**4.731**	**4.907**
Aglenchus	Eupodes	**-1.053**	**0.005**	**4.731**	**4.263**
Aglenchus	Humerobates	**-1.053**	**0.584**	**4.731**	**3.263**
Aglenchus	Oribatida	**-1.053**	**-0.411**	**4.731**	**4.041**
Aglenchus	Scutacarus	**-1.053**	**-0.608**	**4.731**	**3.740**
Aglenchus	Stigmaeidae	**-1.053**	**0.229**	**4.731**	**3.564**
Criconematidae	Mononchidae	**-0.753**	**-0.827**	**5.384**	**4.430**
Criconematidae	Mylonchulus	**-0.753**	**-0.005**	**5.384**	**4.430**
Criconematidae	Lysigamasus	**-0.753**	**0.407**	**5.384**	**3.564**
Criconematidae	Aporcelaimellus	**-0.753**	**0.548**	**5.384**	**5.129**
Criconematidae	Dorylaimoidea	**-0.753**	**-0.604**	**5.384**	**5.544**
Criconematidae	Eudorylaimus	**-0.753**	**-0.166**	**5.384**	**4.731**
Criconematidae	Prodorylaimus	**-0.753**	**-0.836**	**5.384**	**4.731**
Criconematidae	Qudsianematidae	**-0.753**	**-0.207**	**5.384**	**4.430**
Criconematidae	Thornematidae	**-0.753**	**-0.470**	**5.384**	**4.907**
Criconematidae	Eupodes	**-0.753**	**0.005**	**5.384**	**4.263**
Criconematidae	Humerobates	**-0.753**	**0.584**	**5.384**	**3.263**
Criconematidae	Oribatida	**-0.753**	**-0.411**	**5.384**	**4.041**
Criconematidae	Scutacarus	**-0.753**	**-0.608**	**5.384**	**3.740**
Criconematidae	Stigmaeidae	**-0.753**	**0.229**	**5.384**	**3.564**
Dolichodoridae	Mononchidae	**-0.885**	**-0.827**	**4.731**	**4.430**
Dolichodoridae	Mylonchulus	**-0.885**	**-0.005**	**4.731**	**4.430**
Dolichodoridae	Lysigamasus	**-0.885**	**0.407**	**4.731**	**3.564**
Dolichodoridae	Aporcelaimellus	**-0.885**	**0.548**	**4.731**	**5.129**
Dolichodoridae	Dorylaimoidea	**-0.885**	**-0.604**	**4.731**	**5.544**
Dolichodoridae	Eudorylaimus	**-0.885**	**-0.166**	**4.731**	**4.731**
Dolichodoridae	Prodorylaimus	**-0.885**	**-0.836**	**4.731**	**4.731**
Dolichodoridae	Qudsianematidae	**-0.885**	**-0.207**	**4.731**	**4.430**
Dolichodoridae	Thornematidae	**-0.885**	**-0.470**	**4.731**	**4.907**
Dolichodoridae	Eupodes	**-0.885**	**0.005**	**4.731**	**4.263**
Dolichodoridae	Humerobates	**-0.885**	**0.584**	**4.731**	**3.263**
Dolichodoridae	Oribatida	**-0.885**	**-0.411**	**4.731**	**4.041**
Dolichodoridae	Scutacarus	**-0.885**	**-0.608**	**4.731**	**3.740**
Dolichodoridae	Stigmaeidae	**-0.885**	**0.229**	**4.731**	**3.564**
Helicotylenchus	Mononchidae	**-0.792**	**-0.827**	**5.275**	**4.430**
Helicotylenchus	Mylonchulus	**-0.792**	**-0.005**	**5.275**	**4.430**
Helicotylenchus	Lysigamasus	**-0.792**	**0.407**	**5.275**	**3.564**
Helicotylenchus	Aporcelaimellus	**-0.792**	**0.548**	**5.275**	**5.129**
Helicotylenchus	Dorylaimoidea	**-0.792**	**-0.604**	**5.275**	**5.544**
Helicotylenchus	Eudorylaimus	**-0.792**	**-0.166**	**5.275**	**4.731**
Helicotylenchus	Prodorylaimus	**-0.792**	**-0.836**	**5.275**	**4.731**
Helicotylenchus	Qudsianematidae	**-0.792**	**-0.207**	**5.275**	**4.430**

Helicotylenchus	Thornematidae	**-0.792**	**-0.470**	**5.275**	**4.907**
Helicotylenchus	Eupodes	**-0.792**	**0.005**	**5.275**	**4.263**
Helicotylenchus	Humerobates	**-0.792**	**0.584**	**5.275**	**3.263**
Helicotylenchus	Oribatida	**-0.792**	**-0.411**	**5.275**	**4.041**
Helicotylenchus	Scutacarus	**-0.792**	**-0.608**	**5.275**	**3.740**
Helicotylenchus	Stigmaeidae	**-0.792**	**0.229**	**5.275**	**3.564**
Heterodera	Mononchidae	**-0.883**	**-0.827**	**4.430**	**4.430**
Heterodera	Mylonchulus	**-0.883**	**-0.005**	**4.430**	**4.430**
Heterodera	Lysigamasus	**-0.883**	**0.407**	**4.430**	**3.564**
Heterodera	Aporcelaimellus	**-0.883**	**0.548**	**4.430**	**5.129**
Heterodera	Dorylaimoidea	**-0.883**	**-0.604**	**4.430**	**5.544**
Heterodera	Eudorylaimus	**-0.883**	**-0.166**	**4.430**	**4.731**
Heterodera	Prodorylaimus	**-0.883**	**-0.836**	**4.430**	**4.731**
Heterodera	Qudsianematidae	**-0.883**	**-0.207**	**4.430**	**4.430**
Heterodera	Thornematidae	**-0.883**	**-0.470**	**4.430**	**4.907**
Heterodera	Eupodes	**-0.883**	**0.005**	**4.430**	**4.263**
Heterodera	Humerobates	**-0.883**	**0.584**	**4.430**	**3.263**
Heterodera	Oribatida	**-0.883**	**-0.411**	**4.430**	**4.041**
Heterodera	Scutacarus	**-0.883**	**-0.608**	**4.430**	**3.740**
Heterodera	Stigmaeidae	**-0.883**	**0.229**	**4.430**	**3.564**
Paratylenchus	Mononchidae	**-1.244**	**-0.827**	**5.129**	**4.430**
Paratylenchus	Mylonchulus	**-1.244**	**-0.005**	**5.129**	**4.430**
Paratylenchus	Lysigamasus	**-1.244**	**0.407**	**5.129**	**3.564**
Paratylenchus	Aporcelaimellus	**-1.244**	**0.548**	**5.129**	**5.129**
Paratylenchus	Dorylaimoidea	**-1.244**	**-0.604**	**5.129**	**5.544**
Paratylenchus	Eudorylaimus	**-1.244**	**-0.166**	**5.129**	**4.731**
Paratylenchus	Prodorylaimus	**-1.244**	**-0.836**	**5.129**	**4.731**
Paratylenchus	Qudsianematidae	**-1.244**	**-0.207**	**5.129**	**4.430**
Paratylenchus	Thornematidae	**-1.244**	**-0.470**	**5.129**	**4.907**
Paratylenchus	Eupodes	**-1.244**	**0.005**	**5.129**	**4.263**
Paratylenchus	Humerobates	**-1.244**	**0.584**	**5.129**	**3.263**
Paratylenchus	Oribatida	**-1.244**	**-0.411**	**5.129**	**4.041**
Paratylenchus	Scutacarus	**-1.244**	**-0.608**	**5.129**	**3.740**
Paratylenchus	Stigmaeidae	**-1.244**	**0.229**	**5.129**	**3.564**
Trichodorus	Mononchidae	**-0.744**	**-0.827**	**4.430**	**4.430**
Trichodorus	Mylonchulus	**-0.744**	**-0.005**	**4.430**	**4.430**
Trichodorus	Lysigamasus	**-0.744**	**0.407**	**4.430**	**3.564**
Trichodorus	Aporcelaimellus	**-0.744**	**0.548**	**4.430**	**5.129**
Trichodorus	Dorylaimoidea	**-0.744**	**-0.604**	**4.430**	**5.544**
Trichodorus	Eudorylaimus	**-0.744**	**-0.166**	**4.430**	**4.731**
Trichodorus	Prodorylaimus	**-0.744**	**-0.836**	**4.430**	**4.731**
Trichodorus	Qudsianematidae	**-0.744**	**-0.207**	**4.430**	**4.430**
Trichodorus	Thornematidae	**-0.744**	**-0.470**	**4.430**	**4.907**
Trichodorus	Eupodes	**-0.744**	**0.005**	**4.430**	**4.263**
Trichodorus	Humerobates	**-0.744**	**0.584**	**4.430**	**3.263**
Trichodorus	Oribatida	**-0.744**	**-0.411**	**4.430**	**4.041**
Trichodorus	Scutacarus	**-0.744**	**-0.608**	**4.430**	**3.740**
Trichodorus	Stigmaeidae	**-0.744**	**0.229**	**4.430**	**3.564**
Tylenchorhynchus	Mononchidae	**-0.664**	**-0.827**	**4.731**	**4.430**
Tylenchorhynchus	Mylonchulus	**-0.664**	**-0.005**	**4.731**	**4.430**
Tylenchorhynchus	Lysigamasus	**-0.664**	**0.407**	**4.731**	**3.564**

Tylenchorhynchus	Aporcelaimellus	**-0.664**	**0.548**	**4.731**	**5.129**
Tylenchorhynchus	Dorylaimoidea	**-0.664**	**-0.604**	**4.731**	**5.544**
Tylenchorhynchus	Eudorylaimus	**-0.664**	**-0.166**	**4.731**	**4.731**
Tylenchorhynchus	Prodorylaimus	**-0.664**	**-0.836**	**4.731**	**4.731**
Tylenchorhynchus	Qudsianematidae	**-0.664**	**-0.207**	**4.731**	**4.430**
Tylenchorhynchus	Thornematidae	**-0.664**	**-0.470**	**4.731**	**4.907**
Tylenchorhynchus	Eupodes	**-0.664**	**0.005**	**4.731**	**4.263**
Tylenchorhynchus	Humerobates	**-0.664**	**0.584**	**4.731**	**3.263**
Tylenchorhynchus	Oribatida	**-0.664**	**-0.411**	**4.731**	**4.041**
Tylenchorhynchus	Scutacarus	**-0.664**	**-0.608**	**4.731**	**3.740**
Tylenchorhynchus	Stigmaeidae	**-0.664**	**0.229**	**4.731**	**3.564**
Achipteria	Lysigamasus	**0.341**	**0.407**	**3.564**	**3.564**
Achipteria	Aporcelaimellus	**0.341**	**0.548**	**3.564**	**5.129**
Achipteria	Dorylaimoidea	**0.341**	**-0.604**	**3.564**	**5.544**
Achipteria	Eudorylaimus	**0.341**	**-0.166**	**3.564**	**4.731**
Achipteria	Prodorylaimus	**0.341**	**-0.836**	**3.564**	**4.731**
Achipteria	Qudsianematidae	**0.341**	**-0.207**	**3.564**	**4.430**
Achipteria	Thornematidae	**0.341**	**-0.470**	**3.564**	**4.907**
Achipteria	Eupodes	**0.341**	**0.005**	**3.564**	**4.263**
Achipteria	Humerobates	**0.341**	**0.584**	**3.564**	**3.263**
Achipteria	Oribatida	**0.341**	**-0.411**	**3.564**	**4.041**
Achipteria	Scutacarus	**0.341**	**-0.608**	**3.564**	**3.740**
Achipteria	Stigmaeidae	**0.341**	**0.229**	**3.564**	**3.564**
Achipteria	Trombidiidae	**0.341**	**1.787**	**3.564**	**3.263**
Galumna	Lysigamasus	**0.474**	**0.407**	**3.263**	**3.564**
Galumna	Aporcelaimellus	**0.474**	**0.548**	**3.263**	**5.129**
Galumna	Dorylaimoidea	**0.474**	**-0.604**	**3.263**	**5.544**
Galumna	Eudorylaimus	**0.474**	**-0.166**	**3.263**	**4.731**
Galumna	Prodorylaimus	**0.474**	**-0.836**	**3.263**	**4.731**
Galumna	Qudsianematidae	**0.474**	**-0.207**	**3.263**	**4.430**
Galumna	Thornematidae	**0.474**	**-0.470**	**3.263**	**4.907**
Galumna	Eupodes	**0.474**	**0.005**	**3.263**	**4.263**
Galumna	Humerobates	**0.474**	**0.584**	**3.263**	**3.263**
Galumna	Oribatida	**0.474**	**-0.411**	**3.263**	**4.041**
Galumna	Scutacarus	**0.474**	**-0.608**	**3.263**	**3.740**
Galumna	Stigmaeidae	**0.474**	**0.229**	**3.263**	**3.564**
Galumna	Trombidiidae	**0.474**	**1.787**	**3.263**	**3.263**
Platynothrus	Lysigamasus	**0.710**	**0.407**	**3.263**	**3.564**
Platynothrus	Aporcelaimellus	**0.710**	**0.548**	**3.263**	**5.129**
Platynothrus	Dorylaimoidea	**0.710**	**-0.604**	**3.263**	**5.544**
Platynothrus	Eudorylaimus	**0.710**	**-0.166**	**3.263**	**4.731**
Platynothrus	Prodorylaimus	**0.710**	**-0.836**	**3.263**	**4.731**
Platynothrus	Qudsianematidae	**0.710**	**-0.207**	**3.263**	**4.430**
Platynothrus	Thornematidae	**0.710**	**-0.470**	**3.263**	**4.907**
Platynothrus	Eupodes	**0.710**	**0.005**	**3.263**	**4.263**
Platynothrus	Humerobates	**0.710**	**0.584**	**3.263**	**3.263**
Platynothrus	Oribatida	**0.710**	**-0.411**	**3.263**	**4.041**
Platynothrus	Scutacarus	**0.710**	**-0.608**	**3.263**	**3.740**
Platynothrus	Stigmaeidae	**0.710**	**0.229**	**3.263**	**3.564**
Platynothrus	Trombidiidae	**0.710**	**1.787**	**3.263**	**3.263**
Tydeidae	Lysigamasus	**-0.608**	**0.407**	**3.740**	**3.564**

Tydeidae	Aporcelaimellus	**-0.608**	**0.548**	**3.740**	**5.129**
Tydeidae	Dorylaimoidea	**-0.608**	**-0.604**	**3.740**	**5.544**
Tydeidae	Eudorylaimus	**-0.608**	**-0.166**	**3.740**	**4.731**
Tydeidae	Prodorylaimus	**-0.608**	**-0.836**	**3.740**	**4.731**
Tydeidae	Qudsianematidae	**-0.608**	**-0.207**	**3.740**	**4.430**
Tydeidae	Thornematidae	**-0.608**	**-0.470**	**3.740**	**4.907**
Tydeidae	Eupodes	**-0.608**	**0.005**	**3.740**	**4.263**
Tydeidae	Humerobates	**-0.608**	**0.584**	**3.740**	**3.263**
Tydeidae	Oribatida	**-0.608**	**-0.411**	**3.740**	**4.041**
Tydeidae	Scutacarus	**-0.608**	**-0.608**	**3.740**	**3.740**
Tydeidae	Stigmaeidae	**-0.608**	**0.229**	**3.740**	**3.564**
Tydeidae	Trombidiidae	**-0.608**	**1.787**	**3.740**	**3.263**
Sminthuridae	Lysigamasus	**-0.608**	**0.407**	**3.564**	**3.564**
Sminthuridae	Aporcelaimellus	**-0.608**	**0.548**	**3.564**	**5.129**
Sminthuridae	Dorylaimoidea	**-0.608**	**-0.604**	**3.564**	**5.544**
Sminthuridae	Eudorylaimus	**-0.608**	**-0.166**	**3.564**	**4.731**
Sminthuridae	Prodorylaimus	**-0.608**	**-0.836**	**3.564**	**4.731**
Sminthuridae	Qudsianematidae	**-0.608**	**-0.207**	**3.564**	**4.430**
Sminthuridae	Thornematidae	**-0.608**	**-0.470**	**3.564**	**4.907**
Sminthuridae	Eupodes	**-0.608**	**0.005**	**3.564**	**4.263**
Sminthuridae	Humerobates	**-0.608**	**0.584**	**3.564**	**3.263**
Sminthuridae	Oribatida	**-0.608**	**-0.411**	**3.564**	**4.041**
Sminthuridae	Scutacarus	**-0.608**	**-0.608**	**3.564**	**3.740**
Sminthuridae	Stigmaeidae	**-0.608**	**0.229**	**3.564**	**3.564**
Sminthurides	Lysigamasus	**0.005**	**0.407**	**3.564**	**3.564**
Sminthurides	Aporcelaimellus	**0.005**	**0.548**	**3.564**	**5.129**
Sminthurides	Dorylaimoidea	**0.005**	**-0.604**	**3.564**	**5.544**
Sminthurides	Eudorylaimus	**0.005**	**-0.166**	**3.564**	**4.731**
Sminthurides	Prodorylaimus	**0.005**	**-0.836**	**3.564**	**4.731**
Sminthurides	Qudsianematidae	**0.005**	**-0.207**	**3.564**	**4.430**
Sminthurides	Thornematidae	**0.005**	**-0.470**	**3.564**	**4.907**
Sminthurides	Eupodes	**0.005**	**0.005**	**3.564**	**4.263**
Sminthurides	Humerobates	**0.005**	**0.584**	**3.564**	**3.263**
Sminthurides	Oribatida	**0.005**	**-0.411**	**3.564**	**4.041**
Sminthurides	Scutacarus	**0.005**	**-0.608**	**3.564**	**3.740**
Sminthurides	Stigmaeidae	**0.005**	**0.229**	**3.564**	**3.564**
Sminthurus	Lysigamasus	**1.429**	**0.407**	**3.263**	**3.564**
Sminthurus	Aporcelaimellus	**1.429**	**0.548**	**3.263**	**5.129**
Sminthurus	Dorylaimoidea	**1.429**	**-0.604**	**3.263**	**5.544**
Sminthurus	Eudorylaimus	**1.429**	**-0.166**	**3.263**	**4.731**
Sminthurus	Prodorylaimus	**1.429**	**-0.836**	**3.263**	**4.731**
Sminthurus	Qudsianematidae	**1.429**	**-0.207**	**3.263**	**4.430**
Sminthurus	Thornematidae	**1.429**	**-0.470**	**3.263**	**4.907**
Sminthurus	Eupodes	**1.429**	**0.005**	**3.263**	**4.263**
Sminthurus	Humerobates	**1.429**	**0.584**	**3.263**	**3.263**
Sminthurus	Oribatida	**1.429**	**-0.411**	**3.263**	**4.041**
Sminthurus	Scutacarus	**1.429**	**-0.608**	**3.263**	**3.740**
Sminthurus	Stigmaeidae	**1.429**	**0.229**	**3.263**	**3.564**
Aphelenchoides	Mononchidae	**-1.496**	**-0.827**	**5.471**	**4.430**
Aphelenchoides	Mylonchulus	**-1.496**	**-0.005**	**5.471**	**4.430**
Aphelenchoides	Lysigamasus	**-1.496**	**0.407**	**5.471**	**3.564**

Aphelenchoides	Aporcelaimellus	**-1.496**	**0.548**	**5.471**	**5.129**
Aphelenchoides	Dorylaimoidea	**-1.496**	**-0.604**	**5.471**	**5.544**
Aphelenchoides	Eudorylaimus	**-1.496**	**-0.166**	**5.471**	**4.731**
Aphelenchoides	Prodorylaimus	**-1.496**	**-0.836**	**5.471**	**4.731**
Aphelenchoides	Qudsianematidae	**-1.496**	**-0.207**	**5.471**	**4.430**
Aphelenchoides	Thornematidae	**-1.496**	**-0.470**	**5.471**	**4.907**
Aphelenchoides	Eupodes	**-1.496**	**0.005**	**5.471**	**4.263**
Aphelenchoides	Humerobates	**-1.496**	**0.584**	**5.471**	**3.263**
Aphelenchoides	Oribatida	**-1.496**	**-0.411**	**5.471**	**4.041**
Aphelenchoides	Scutacarus	**-1.496**	**-0.608**	**5.471**	**3.740**
Aphelenchoides	Stigmaeidae	**-1.496**	**0.229**	**5.471**	**3.564**
Diphtherophora	Mononchidae	**-1.080**	**-0.827**	**4.430**	**4.430**
Diphtherophora	Mylonchulus	**-1.080**	**-0.005**	**4.430**	**4.430**
Diphtherophora	Lysigamasus	**-1.080**	**0.407**	**4.430**	**3.564**
Diphtherophora	Aporcelaimellus	**-1.080**	**0.548**	**4.430**	**5.129**
Diphtherophora	Dorylaimoidea	**-1.080**	**-0.604**	**4.430**	**5.544**
Diphtherophora	Eudorylaimus	**-1.080**	**-0.166**	**4.430**	**4.731**
Diphtherophora	Prodorylaimus	**-1.080**	**-0.836**	**4.430**	**4.731**
Diphtherophora	Qudsianematidae	**-1.080**	**-0.207**	**4.430**	**4.430**
Diphtherophora	Thornematidae	**-1.080**	**-0.470**	**4.430**	**4.907**
Diphtherophora	Eupodes	**-1.080**	**0.005**	**4.430**	**4.263**
Diphtherophora	Humerobates	**-1.080**	**0.584**	**4.430**	**3.263**
Diphtherophora	Oribatida	**-1.080**	**-0.411**	**4.430**	**4.041**
Diphtherophora	Scutacarus	**-1.080**	**-0.608**	**4.430**	**3.740**
Diphtherophora	Stigmaeidae	**-1.080**	**0.229**	**4.430**	**3.564**
Tylenchidae	Mononchidae	**-1.360**	**-0.827**	**6.073**	**4.430**
Tylenchidae	Mylonchulus	**-1.360**	**-0.005**	**6.073**	**4.430**
Tylenchidae	Lysigamasus	**-1.360**	**0.407**	**6.073**	**3.564**
Tylenchidae	Aporcelaimellus	**-1.360**	**0.548**	**6.073**	**5.129**
Tylenchidae	Dorylaimoidea	**-1.360**	**-0.604**	**6.073**	**5.544**
Tylenchidae	Eudorylaimus	**-1.360**	**-0.166**	**6.073**	**4.731**
Tylenchidae	Prodorylaimus	**-1.360**	**-0.836**	**6.073**	**4.731**
Tylenchidae	Qudsianematidae	**-1.360**	**-0.207**	**6.073**	**4.430**
Tylenchidae	Thornematidae	**-1.360**	**-0.470**	**6.073**	**4.907**
Tylenchidae	Eupodes	**-1.360**	**0.005**	**6.073**	**4.263**
Tylenchidae	Humerobates	**-1.360**	**0.584**	**6.073**	**3.263**
Tylenchidae	Oribatida	**-1.360**	**-0.411**	**6.073**	**4.041**
Tylenchidae	Scutacarus	**-1.360**	**-0.608**	**6.073**	**3.740**
Tylenchidae	Stigmaeidae	**-1.360**	**0.229**	**6.073**	**3.564**
Brachychthonius	Lysigamasus	**-0.586**	**0.407**	**3.263**	**3.564**
Brachychthonius	Aporcelaimellus	**-0.586**	**0.548**	**3.263**	**5.129**
Brachychthonius	Dorylaimoidea	**-0.586**	**-0.604**	**3.263**	**5.544**
Brachychthonius	Eudorylaimus	**-0.586**	**-0.166**	**3.263**	**4.731**
Brachychthonius	Prodorylaimus	**-0.586**	**-0.836**	**3.263**	**4.731**
Brachychthonius	Qudsianematidae	**-0.586**	**-0.207**	**3.263**	**4.430**
Brachychthonius	Thornematidae	**-0.586**	**-0.470**	**3.263**	**4.907**
Brachychthonius	Eupodes	**-0.586**	**0.005**	**3.263**	**4.263**
Brachychthonius	Humerobates	**-0.586**	**0.584**	**3.263**	**3.263**
Brachychthonius	Oribatida	**-0.586**	**-0.411**	**3.263**	**4.041**
Brachychthonius	Scutacarus	**-0.586**	**-0.608**	**3.263**	**3.740**
Brachychthonius	Stigmaeidae	**-0.586**	**0.229**	**3.263**	**3.564**

Brachychthonius	Trombidiidae	**-0.586**	**1.787**	**3.263**	**3.263**
Microtydeus	Lysigamasus	**-0.863**	**0.407**	**3.263**	**3.564**
Microtydeus	Aporcelaimellus	**-0.863**	**0.548**	**3.263**	**5.129**
Microtydeus	Dorylaimoidea	**-0.863**	**-0.604**	**3.263**	**5.544**
Microtydeus	Eudorylaimus	**-0.863**	**-0.166**	**3.263**	**4.731**
Microtydeus	Prodorylaimus	**-0.863**	**-0.836**	**3.263**	**4.731**
Microtydeus	Qudsianematidae	**-0.863**	**-0.207**	**3.263**	**4.430**
Microtydeus	Thornematidae	**-0.863**	**-0.470**	**3.263**	**4.907**
Microtydeus	Eupodes	**-0.863**	**0.005**	**3.263**	**4.263**
Microtydeus	Humerobates	**-0.863**	**0.584**	**3.263**	**3.263**
Microtydeus	Oribatida	**-0.863**	**-0.411**	**3.263**	**4.041**
Microtydeus	Scutacarus	**-0.863**	**-0.608**	**3.263**	**3.740**
Microtydeus	Stigmaeidae	**-0.863**	**0.229**	**3.263**	**3.564**
Microtydeus	Trombidiidae	**-0.863**	**1.787**	**3.263**	**3.263**
Minunthozetes	Lysigamasus	**-0.249**	**0.407**	**3.740**	**3.564**
Minunthozetes	Aporcelaimellus	**-0.249**	**0.548**	**3.740**	**5.129**
Minunthozetes	Dorylaimoidea	**-0.249**	**-0.604**	**3.740**	**5.544**
Minunthozetes	Eudorylaimus	**-0.249**	**-0.166**	**3.740**	**4.731**
Minunthozetes	Prodorylaimus	**-0.249**	**-0.836**	**3.740**	**4.731**
Minunthozetes	Qudsianematidae	**-0.249**	**-0.207**	**3.740**	**4.430**
Minunthozetes	Thornematidae	**-0.249**	**-0.470**	**3.740**	**4.907**
Minunthozetes	Eupodes	**-0.249**	**0.005**	**3.740**	**4.263**
Minunthozetes	Humerobates	**-0.249**	**0.584**	**3.740**	**3.263**
Minunthozetes	Oribatida	**-0.249**	**-0.411**	**3.740**	**4.041**
Minunthozetes	Scutacarus	**-0.249**	**-0.608**	**3.740**	**3.740**
Minunthozetes	Stigmaeidae	**-0.249**	**0.229**	**3.740**	**3.564**
Minunthozetes	Trombidiidae	**-0.249**	**1.787**	**3.740**	**3.263**
Oppiella	Lysigamasus	**-0.447**	**0.407**	**3.865**	**3.564**
Oppiella	Aporcelaimellus	**-0.447**	**0.548**	**3.865**	**5.129**
Oppiella	Dorylaimoidea	**-0.447**	**-0.604**	**3.865**	**5.544**
Oppiella	Eudorylaimus	**-0.447**	**-0.166**	**3.865**	**4.731**
Oppiella	Prodorylaimus	**-0.447**	**-0.836**	**3.865**	**4.731**
Oppiella	Qudsianematidae	**-0.447**	**-0.207**	**3.865**	**4.430**
Oppiella	Thornematidae	**-0.447**	**-0.470**	**3.865**	**4.907**
Oppiella	Eupodes	**-0.447**	**0.005**	**3.865**	**4.263**
Oppiella	Humerobates	**-0.447**	**0.584**	**3.865**	**3.263**
Oppiella	Oribatida	**-0.447**	**-0.411**	**3.865**	**4.041**
Oppiella	Scutacarus	**-0.447**	**-0.608**	**3.865**	**3.740**
Oppiella	Stigmaeidae	**-0.447**	**0.229**	**3.865**	**3.564**
Oppiella	Trombidiidae	**-0.447**	**1.787**	**3.865**	**3.263**
Punctoribates	Lysigamasus	**-0.029**	**0.407**	**4.108**	**3.564**
Punctoribates	Aporcelaimellus	**-0.029**	**0.548**	**4.108**	**5.129**
Punctoribates	Dorylaimoidea	**-0.029**	**-0.604**	**4.108**	**5.544**
Punctoribates	Eudorylaimus	**-0.029**	**-0.166**	**4.108**	**4.731**
Punctoribates	Prodorylaimus	**-0.029**	**-0.836**	**4.108**	**4.731**
Punctoribates	Qudsianematidae	**-0.029**	**-0.207**	**4.108**	**4.430**
Punctoribates	Thornematidae	**-0.029**	**-0.470**	**4.108**	**4.907**
Punctoribates	Eupodes	**-0.029**	**0.005**	**4.108**	**4.263**
Punctoribates	Humerobates	**-0.029**	**0.584**	**4.108**	**3.263**
Punctoribates	Oribatida	**-0.029**	**-0.411**	**4.108**	**4.041**
Punctoribates	Scutacarus	**-0.029**	**-0.608**	**4.108**	**3.740**

Punctoribates	Stigmaeidae	**-0.029**	**0.229**	**4.108**	**3.564**
Punctoribates	Trombidiidae	**-0.029**	**1.787**	**4.108**	**3.263**
Isotoma	Lysigamasus	**1.898**	**0.407**	**3.263**	**3.564**
Isotoma	Aporcelaimellus	**1.898**	**0.548**	**3.263**	**5.129**
Isotoma	Dorylaimoidea	**1.898**	**-0.604**	**3.263**	**5.544**
Isotoma	Eudorylaimus	**1.898**	**-0.166**	**3.263**	**4.731**
Isotoma	Prodorylaimus	**1.898**	**-0.836**	**3.263**	**4.731**
Isotoma	Qudsianematidae	**1.898**	**-0.207**	**3.263**	**4.430**
Isotoma	Thornematidae	**1.898**	**-0.470**	**3.263**	**4.907**
Isotoma	Eupodes	**1.898**	**0.005**	**3.263**	**4.263**
Isotoma	Humerobates	**1.898**	**0.584**	**3.263**	**3.263**
Isotoma	Oribatida	**1.898**	**-0.411**	**3.263**	**4.041**
Isotoma	Scutacarus	**1.898**	**-0.608**	**3.263**	**3.740**
Isotoma	Stigmaeidae	**1.898**	**0.229**	**3.263**	**3.564**
Lepidocyrtus	Lysigamasus	**1.231**	**0.407**	**4.041**	**3.564**
Lepidocyrtus	Aporcelaimellus	**1.231**	**0.548**	**4.041**	**5.129**
Lepidocyrtus	Dorylaimoidea	**1.231**	**-0.604**	**4.041**	**5.544**
Lepidocyrtus	Eudorylaimus	**1.231**	**-0.166**	**4.041**	**4.731**
Lepidocyrtus	Prodorylaimus	**1.231**	**-0.836**	**4.041**	**4.731**
Lepidocyrtus	Qudsianematidae	**1.231**	**-0.207**	**4.041**	**4.430**
Lepidocyrtus	Thornematidae	**1.231**	**-0.470**	**4.041**	**4.907**
Lepidocyrtus	Eupodes	**1.231**	**0.005**	**4.041**	**4.263**
Lepidocyrtus	Humerobates	**1.231**	**0.584**	**4.041**	**3.263**
Lepidocyrtus	Oribatida	**1.231**	**-0.411**	**4.041**	**4.041**
Lepidocyrtus	Scutacarus	**1.231**	**-0.608**	**4.041**	**3.740**
Lepidocyrtus	Stigmaeidae	**1.231**	**0.229**	**4.041**	**3.564**
Proisotoma	Lysigamasus	**0.770**	**0.407**	**3.962**	**3.564**
Proisotoma	Aporcelaimellus	**0.770**	**0.548**	**3.962**	**5.129**
Proisotoma	Dorylaimoidea	**0.770**	**-0.604**	**3.962**	**5.544**
Proisotoma	Eudorylaimus	**0.770**	**-0.166**	**3.962**	**4.731**
Proisotoma	Prodorylaimus	**0.770**	**-0.836**	**3.962**	**4.731**
Proisotoma	Qudsianematidae	**0.770**	**-0.207**	**3.962**	**4.430**
Proisotoma	Thornematidae	**0.770**	**-0.470**	**3.962**	**4.907**
Proisotoma	Eupodes	**0.770**	**0.005**	**3.962**	**4.263**
Proisotoma	Humerobates	**0.770**	**0.584**	**3.962**	**3.263**
Proisotoma	Oribatida	**0.770**	**-0.411**	**3.962**	**4.041**
Proisotoma	Scutacarus	**0.770**	**-0.608**	**3.962**	**3.740**
Proisotoma	Stigmaeidae	**0.770**	**0.229**	**3.962**	**3.564**
Achaeta	Lysigamasus	**1.395**	**0.407**	**2.373**	**3.564**
Achaeta	Aporcelaimellus	**1.395**	**0.548**	**2.373**	**5.129**
Achaeta	Dorylaimoidea	**1.395**	**-0.604**	**2.373**	**5.544**
Achaeta	Eudorylaimus	**1.395**	**-0.166**	**2.373**	**4.731**
Achaeta	Prodorylaimus	**1.395**	**-0.836**	**2.373**	**4.731**
Achaeta	Qudsianematidae	**1.395**	**-0.207**	**2.373**	**4.430**
Achaeta	Thornematidae	**1.395**	**-0.470**	**2.373**	**4.907**
Achaeta	Eupodes	**1.395**	**0.005**	**2.373**	**4.263**
Achaeta	Humerobates	**1.395**	**0.584**	**2.373**	**3.263**
Achaeta	Oribatida	**1.395**	**-0.411**	**2.373**	**4.041**
Achaeta	Scutacarus	**1.395**	**-0.608**	**2.373**	**3.740**
Achaeta	Stigmaeidae	**1.395**	**0.229**	**2.373**	**3.564**
Cognettia	Lysigamasus	**1.727**	**0.407**	**4.111**	**3.564**

Cognettia	Aporcelaimellus	**1.727**	**0.548**	**4.111**	**5.129**
Cognettia	Dorylaimoidea	**1.727**	**-0.604**	**4.111**	**5.544**
Cognettia	Eudorylaimus	**1.727**	**-0.166**	**4.111**	**4.731**
Cognettia	Prodorylaimus	**1.727**	**-0.836**	**4.111**	**4.731**
Cognettia	Qudsianematidae	**1.727**	**-0.207**	**4.111**	**4.430**
Cognettia	Thornematidae	**1.727**	**-0.470**	**4.111**	**4.907**
Cognettia	Eupodes	**1.727**	**0.005**	**4.111**	**4.263**
Cognettia	Humerobates	**1.727**	**0.584**	**4.111**	**3.263**
Cognettia	Oribatida	**1.727**	**-0.411**	**4.111**	**4.041**
Cognettia	Scutacarus	**1.727**	**-0.608**	**4.111**	**3.740**
Cognettia	Stigmaeidae	**1.727**	**0.229**	**4.111**	**3.564**
Fridericia	Lysigamasus	**1.797**	**0.407**	**2.674**	**3.564**
Fridericia	Aporcelaimellus	**1.797**	**0.548**	**2.674**	**5.129**
Fridericia	Dorylaimoidea	**1.797**	**-0.604**	**2.674**	**5.544**
Fridericia	Eudorylaimus	**1.797**	**-0.166**	**2.674**	**4.731**
Fridericia	Prodorylaimus	**1.797**	**-0.836**	**2.674**	**4.731**
Fridericia	Qudsianematidae	**1.797**	**-0.207**	**2.674**	**4.430**
Fridericia	Thornematidae	**1.797**	**-0.470**	**2.674**	**4.907**
Fridericia	Eupodes	**1.797**	**0.005**	**2.674**	**4.263**
Fridericia	Humerobates	**1.797**	**0.584**	**2.674**	**3.263**
Fridericia	Oribatida	**1.797**	**-0.411**	**2.674**	**4.041**
Fridericia	Scutacarus	**1.797**	**-0.608**	**2.674**	**3.740**
Fridericia	Stigmaeidae	**1.797**	**0.229**	**2.674**	**3.564**
Acrobeloides	Mononchidae	**-1.171**	**-0.827**	**5.333**	**4.430**
Acrobeloides	Mylonchulus	**-1.171**	**-0.005**	**5.333**	**4.430**
Acrobeloides	Lysigamasus	**-1.171**	**0.407**	**5.333**	**3.564**
Acrobeloides	Aporcelaimellus	**-1.171**	**0.548**	**5.333**	**5.129**
Acrobeloides	Dorylaimoidea	**-1.171**	**-0.604**	**5.333**	**5.544**
Acrobeloides	Eudorylaimus	**-1.171**	**-0.166**	**5.333**	**4.731**
Acrobeloides	Prodorylaimus	**-1.171**	**-0.836**	**5.333**	**4.731**
Acrobeloides	Qudsianematidae	**-1.171**	**-0.207**	**5.333**	**4.430**
Acrobeloides	Thornematidae	**-1.171**	**-0.470**	**5.333**	**4.907**
Acrobeloides	Eupodes	**-1.171**	**0.005**	**5.333**	**4.263**
Acrobeloides	Humerobates	**-1.171**	**0.584**	**5.333**	**3.263**
Acrobeloides	Oribatida	**-1.171**	**-0.411**	**5.333**	**4.041**
Acrobeloides	Scutacarus	**-1.171**	**-0.608**	**5.333**	**3.740**
Acrobeloides	Stigmaeidae	**-1.171**	**0.229**	**5.333**	**3.564**
Anaplectus	Mononchidae	**-0.519**	**-0.827**	**4.430**	**4.430**
Anaplectus	Mylonchulus	**-0.519**	**-0.005**	**4.430**	**4.430**
Anaplectus	Lysigamasus	**-0.519**	**0.407**	**4.430**	**3.564**
Anaplectus	Aporcelaimellus	**-0.519**	**0.548**	**4.430**	**5.129**
Anaplectus	Dorylaimoidea	**-0.519**	**-0.604**	**4.430**	**5.544**
Anaplectus	Eudorylaimus	**-0.519**	**-0.166**	**4.430**	**4.731**
Anaplectus	Prodorylaimus	**-0.519**	**-0.836**	**4.430**	**4.731**
Anaplectus	Qudsianematidae	**-0.519**	**-0.207**	**4.430**	**4.430**
Anaplectus	Thornematidae	**-0.519**	**-0.470**	**4.430**	**4.907**
Anaplectus	Eupodes	**-0.519**	**0.005**	**4.430**	**4.263**
Anaplectus	Humerobates	**-0.519**	**0.584**	**4.430**	**3.263**
Anaplectus	Oribatida	**-0.519**	**-0.411**	**4.430**	**4.041**
Anaplectus	Scutacarus	**-0.519**	**-0.608**	**4.430**	**3.740**
Anaplectus	Stigmaeidae	**-0.519**	**0.229**	**4.430**	**3.564**

Bastiania	Mononchidae	**-0.946**	**-0.827**	**4.430**	**4.430**
Bastiania	Mylonchulus	**-0.946**	**-0.005**	**4.430**	**4.430**
Bastiania	Lysigamasus	**-0.946**	**0.407**	**4.430**	**3.564**
Bastiania	Aporcelaimellus	**-0.946**	**0.548**	**4.430**	**5.129**
Bastiania	Dorylaimoidea	**-0.946**	**-0.604**	**4.430**	**5.544**
Bastiania	Eudorylaimus	**-0.946**	**-0.166**	**4.430**	**4.731**
Bastiania	Prodorylaimus	**-0.946**	**-0.836**	**4.430**	**4.731**
Bastiania	Qudsianematidae	**-0.946**	**-0.207**	**4.430**	**4.430**
Bastiania	Thornematidae	**-0.946**	**-0.470**	**4.430**	**4.907**
Bastiania	Eupodes	**-0.946**	**0.005**	**4.430**	**4.263**
Bastiania	Humerobates	**-0.946**	**0.584**	**4.430**	**3.263**
Bastiania	Oribatida	**-0.946**	**-0.411**	**4.430**	**4.041**
Bastiania	Scutacarus	**-0.946**	**-0.608**	**4.430**	**3.740**
Bastiania	Stigmaeidae	**-0.946**	**0.229**	**4.430**	**3.564**
Eucephalobus	Mononchidae	**-0.855**	**-0.827**	**5.208**	**4.430**
Eucephalobus	Mylonchulus	**-0.855**	**-0.005**	**5.208**	**4.430**
Eucephalobus	Lysigamasus	**-0.855**	**0.407**	**5.208**	**3.564**
Eucephalobus	Aporcelaimellus	**-0.855**	**0.548**	**5.208**	**5.129**
Eucephalobus	Dorylaimoidea	**-0.855**	**-0.604**	**5.208**	**5.544**
Eucephalobus	Eudorylaimus	**-0.855**	**-0.166**	**5.208**	**4.731**
Eucephalobus	Prodorylaimus	**-0.855**	**-0.836**	**5.208**	**4.731**
Eucephalobus	Qudsianematidae	**-0.855**	**-0.207**	**5.208**	**4.430**
Eucephalobus	Thornematidae	**-0.855**	**-0.470**	**5.208**	**4.907**
Eucephalobus	Eupodes	**-0.855**	**0.005**	**5.208**	**4.263**
Eucephalobus	Humerobates	**-0.855**	**0.584**	**5.208**	**3.263**
Eucephalobus	Oribatida	**-0.855**	**-0.411**	**5.208**	**4.041**
Eucephalobus	Scutacarus	**-0.855**	**-0.608**	**5.208**	**3.740**
Eucephalobus	Stigmaeidae	**-0.855**	**0.229**	**5.208**	**3.564**
Eumonhystera	Mononchidae	**-0.613**	**-0.827**	**4.430**	**4.430**
Eumonhystera	Mylonchulus	**-0.613**	**-0.005**	**4.430**	**4.430**
Eumonhystera	Lysigamasus	**-0.613**	**0.407**	**4.430**	**3.564**
Eumonhystera	Aporcelaimellus	**-0.613**	**0.548**	**4.430**	**5.129**
Eumonhystera	Dorylaimoidea	**-0.613**	**-0.604**	**4.430**	**5.544**
Eumonhystera	Eudorylaimus	**-0.613**	**-0.166**	**4.430**	**4.731**
Eumonhystera	Prodorylaimus	**-0.613**	**-0.836**	**4.430**	**4.731**
Eumonhystera	Qudsianematidae	**-0.613**	**-0.207**	**4.430**	**4.430**
Eumonhystera	Thornematidae	**-0.613**	**-0.470**	**4.430**	**4.907**
Eumonhystera	Eupodes	**-0.613**	**0.005**	**4.430**	**4.263**
Eumonhystera	Humerobates	**-0.613**	**0.584**	**4.430**	**3.263**
Eumonhystera	Oribatida	**-0.613**	**-0.411**	**4.430**	**4.041**
Eumonhystera	Scutacarus	**-0.613**	**-0.608**	**4.430**	**3.740**
Eumonhystera	Stigmaeidae	**-0.613**	**0.229**	**4.430**	**3.564**
Metateratocephalus	Mononchidae	**-1.506**	**-0.827**	**4.430**	**4.430**
Metateratocephalus	Mylonchulus	**-1.506**	**-0.005**	**4.430**	**4.430**
Metateratocephalus	Lysigamasus	**-1.506**	**0.407**	**4.430**	**3.564**
Metateratocephalus	Aporcelaimellus	**-1.506**	**0.548**	**4.430**	**5.129**
Metateratocephalus	Dorylaimoidea	**-1.506**	**-0.604**	**4.430**	**5.544**
Metateratocephalus	Eudorylaimus	**-1.506**	**-0.166**	**4.430**	**4.731**
Metateratocephalus	Prodorylaimus	**-1.506**	**-0.836**	**4.430**	**4.731**
Metateratocephalus	Qudsianematidae	**-1.506**	**-0.207**	**4.430**	**4.430**
Metateratocephalus	Thornematidae	**-1.506**	**-0.470**	**4.430**	**4.907**

Metateratocephalus	Eupodes	**-1.506**	**0.005**	**4.430**	**4.263**
Metateratocephalus	Humerobates	**-1.506**	**0.584**	**4.430**	**3.263**
Metateratocephalus	Oribatida	**-1.506**	**-0.411**	**4.430**	**4.041**
Metateratocephalus	Scutacarus	**-1.506**	**-0.608**	**4.430**	**3.740**
Metateratocephalus	Stigmaeidae	**-1.506**	**0.229**	**4.430**	**3.564**
Plectus	Mononchidae	**-0.583**	**-0.827**	**5.430**	**4.430**
Plectus	Mylonchulus	**-0.583**	**-0.005**	**5.430**	**4.430**
Plectus	Lysigamasus	**-0.583**	**0.407**	**5.430**	**3.564**
Plectus	Aporcelaimellus	**-0.583**	**0.548**	**5.430**	**5.129**
Plectus	Dorylaimoidea	**-0.583**	**-0.604**	**5.430**	**5.544**
Plectus	Eudorylaimus	**-0.583**	**-0.166**	**5.430**	**4.731**
Plectus	Prodorylaimus	**-0.583**	**-0.836**	**5.430**	**4.731**
Plectus	Qudsianematidae	**-0.583**	**-0.207**	**5.430**	**4.430**
Plectus	Thornematidae	**-0.583**	**-0.470**	**5.430**	**4.907**
Plectus	Eupodes	**-0.583**	**0.005**	**5.430**	**4.263**
Plectus	Humerobates	**-0.583**	**0.584**	**5.430**	**3.263**
Plectus	Oribatida	**-0.583**	**-0.411**	**5.430**	**4.041**
Plectus	Scutacarus	**-0.583**	**-0.608**	**5.430**	**3.740**
Plectus	Stigmaeidae	**-0.583**	**0.229**	**5.430**	**3.564**
Prismatolaimus	Mononchidae	**-1.280**	**-0.827**	**4.430**	**4.430**
Prismatolaimus	Mylonchulus	**-1.280**	**-0.005**	**4.430**	**4.430**
Prismatolaimus	Lysigamasus	**-1.280**	**0.407**	**4.430**	**3.564**
Prismatolaimus	Aporcelaimellus	**-1.280**	**0.548**	**4.430**	**5.129**
Prismatolaimus	Dorylaimoidea	**-1.280**	**-0.604**	**4.430**	**5.544**
Prismatolaimus	Eudorylaimus	**-1.280**	**-0.166**	**4.430**	**4.731**
Prismatolaimus	Prodorylaimus	**-1.280**	**-0.836**	**4.430**	**4.731**
Prismatolaimus	Qudsianematidae	**-1.280**	**-0.207**	**4.430**	**4.430**
Prismatolaimus	Thornematidae	**-1.280**	**-0.470**	**4.430**	**4.907**
Prismatolaimus	Eupodes	**-1.280**	**0.005**	**4.430**	**4.263**
Prismatolaimus	Humerobates	**-1.280**	**0.584**	**4.430**	**3.263**
Prismatolaimus	Oribatida	**-1.280**	**-0.411**	**4.430**	**4.041**
Prismatolaimus	Scutacarus	**-1.280**	**-0.608**	**4.430**	**3.740**
Prismatolaimus	Stigmaeidae	**-1.280**	**0.229**	**4.430**	**3.564**
Teratocephalus	Mononchidae	**-1.630**	**-0.827**	**5.333**	**4.430**
Teratocephalus	Mylonchulus	**-1.630**	**-0.005**	**5.333**	**4.430**
Teratocephalus	Lysigamasus	**-1.630**	**0.407**	**5.333**	**3.564**
Teratocephalus	Aporcelaimellus	**-1.630**	**0.548**	**5.333**	**5.129**
Teratocephalus	Dorylaimoidea	**-1.630**	**-0.604**	**5.333**	**5.544**
Teratocephalus	Eudorylaimus	**-1.630**	**-0.166**	**5.333**	**4.731**
Teratocephalus	Prodorylaimus	**-1.630**	**-0.836**	**5.333**	**4.731**
Teratocephalus	Qudsianematidae	**-1.630**	**-0.207**	**5.333**	**4.430**
Teratocephalus	Thornematidae	**-1.630**	**-0.470**	**5.333**	**4.907**
Teratocephalus	Eupodes	**-1.630**	**0.005**	**5.333**	**4.263**
Teratocephalus	Humerobates	**-1.630**	**0.584**	**5.333**	**3.263**
Teratocephalus	Oribatida	**-1.630**	**-0.411**	**5.333**	**4.041**
Teratocephalus	Scutacarus	**-1.630**	**-0.608**	**5.333**	**3.740**
Teratocephalus	Stigmaeidae	**-1.630**	**0.229**	**5.333**	**3.564**
Enchytronia	Lysigamasus	**0.530**	**0.407**	**2.072**	**3.564**
Enchytronia	Aporcelaimellus	**0.530**	**0.548**	**2.072**	**5.129**
Enchytronia	Dorylaimoidea	**0.530**	**-0.604**	**2.072**	**5.544**
Enchytronia	Eudorylaimus	**0.530**	**-0.166**	**2.072**	**4.731**

Enchytronia	Prodorylaimus	**0.530**	**-0.836**	**2.072**	**4.731**
Enchytronia	Qudsianematidae	**0.530**	**-0.207**	**2.072**	**4.430**
Enchytronia	Thornematidae	**0.530**	**-0.470**	**2.072**	**4.907**
Enchytronia	Eupodes	**0.530**	**0.005**	**2.072**	**4.263**
Enchytronia	Humerobates	**0.530**	**0.584**	**2.072**	**3.263**
Enchytronia	Oribatida	**0.530**	**-0.411**	**2.072**	**4.041**
Enchytronia	Scutacarus	**0.530**	**-0.608**	**2.072**	**3.740**
Enchytronia	Stigmaeidae	**0.530**	**0.229**	**2.072**	**3.564**
Eubacteria	Acrobeloides	**-6.534**	**-1.171**	**12.788**	**5.333**
Eubacteria	Anaplectus	**-6.534**	**-0.519**	**12.788**	**4.430**
Eubacteria	Bastiania	**-6.534**	**-0.946**	**12.788**	**4.430**
Eubacteria	Eucephalobus	**-6.534**	**-0.855**	**12.788**	**5.208**
Eubacteria	Eumonhystera	**-6.534**	**-0.613**	**12.788**	**4.430**
Eubacteria	Metateratocephalus	**-6.534**	**-1.506**	**12.788**	**4.430**
Eubacteria	Plectus	**-6.534**	**-0.583**	**12.788**	**5.430**
Eubacteria	Prismatolaimus	**-6.534**	**-1.280**	**12.788**	**4.430**
Eubacteria	Teratocephalus	**-6.534**	**-1.630**	**12.788**	**5.333**
Eubacteria	Enchytronia	**-6.534**	**0.530**	**12.788**	**2.072**
Eubacteria	Marionina	**-6.534**	**0.911**	**12.788**	**3.168**
Marionina	Lysigamasus	**0.911**	**0.407**	**3.168**	**3.564**
Marionina	Aporcelaimellus	**0.911**	**0.548**	**3.168**	**5.129**
Marionina	Dorylaimoidea	**0.911**	**-0.604**	**3.168**	**5.544**
Marionina	Eudorylaimus	**0.911**	**-0.166**	**3.168**	**4.731**
Marionina	Prodorylaimus	**0.911**	**-0.836**	**3.168**	**4.731**
Marionina	Qudsianematidae	**0.911**	**-0.207**	**3.168**	**4.430**
Marionina	Thornematidae	**0.911**	**-0.470**	**3.168**	**4.907**
Marionina	Eupodes	**0.911**	**0.005**	**3.168**	**4.263**
Marionina	Humerobates	**0.911**	**0.584**	**3.168**	**3.263**
Marionina	Oribatida	**0.911**	**-0.411**	**3.168**	**4.041**
Marionina	Scutacarus	**0.911**	**-0.608**	**3.168**	**3.740**
Marionina	Stigmaeidae	**0.911**	**0.229**	**3.168**	**3.564**
Hyphae and hair roots	Aglenchus	**6.344**	**-1.053**	**0.000**	**4.731**
Hyphae and hair roots	Criconematidae	**6.344**	**-0.753**	**0.000**	**5.384**
Hyphae and hair roots	Dolichodoridae	**6.344**	**-0.885**	**0.000**	**4.731**
Hyphae and hair roots	Helicotylenchus	**6.344**	**-0.792**	**0.000**	**5.275**
Hyphae and hair roots	Heterodera	**6.344**	**-0.883**	**0.000**	**4.430**
Hyphae and hair roots	Paratylenchus	**6.344**	**-1.244**	**0.000**	**5.129**
Hyphae and hair roots	Trichodorus	**6.344**	**-0.744**	**0.000**	**4.430**
Hyphae and hair roots	Tylenchorhynchus	**6.344**	**-0.664**	**0.000**	**4.731**
Hyphae and hair roots	Achipteria	**6.344**	**0.341**	**0.000**	**3.564**
Hyphae and hair roots	Galumna	**6.344**	**0.474**	**0.000**	**3.263**
Hyphae and hair roots	Platynothrus	**6.344**	**0.710**	**0.000**	**3.263**
Hyphae and hair roots	Tydeidae	**6.344**	**-0.608**	**0.000**	**3.740**
Hyphae and hair roots	Sminthuridae	**6.344**	**-0.608**	**0.000**	**3.564**
Hyphae and hair roots	Sminthurides	**6.344**	**0.005**	**0.000**	**3.564**
Hyphae and hair roots	Sminthurus	**6.344**	**1.429**	**0.000**	**3.263**
Hyphae and hair roots	Aphelenchoides	**6.344**	**-1.496**	**0.000**	**5.471**
Hyphae and hair roots	Diphtherophora	**6.344**	**-1.080**	**0.000**	**4.430**
Hyphae and hair roots	Tylenchidae	**6.344**	**-1.360**	**0.000**	**6.073**
Hyphae and hair roots	Brachychthonius	**6.344**	**-0.586**	**0.000**	**3.263**
Hyphae and hair roots	Microtydeus	**6.344**	**-0.863**	**0.000**	**3.263**

Hyphae and hair roots	Minunthozetes	**6.344**	**-0.249**	**0.000**	**3.740**
Hyphae and hair roots	Oppiella	**6.344**	**-0.447**	**0.000**	**3.865**
Hyphae and hair roots	Punctoribates	**6.344**	**-0.029**	**0.000**	**4.108**
Hyphae and hair roots	Isotoma	**6.344**	**1.898**	**0.000**	**3.263**
Hyphae and hair roots	Lepidocyrtus	**6.344**	**1.231**	**0.000**	**4.041**
Hyphae and hair roots	Proisotoma	**6.344**	**0.770**	**0.000**	**3.962**
Hyphae and hair roots	Achaeta	**6.344**	**1.395**	**0.000**	**2.373**
Hyphae and hair roots	Cognettia	**6.344**	**1.727**	**0.000**	**4.111**
Hyphae and hair roots	Fridericia	**6.344**	**1.797**	**0.000**	**2.674**
Hyphae and hair roots	Aporcelaimellus	**6.344**	**0.548**	**0.000**	**5.129**
Hyphae and hair roots	Dorylaimoidea	**6.344**	**-0.604**	**0.000**	**5.544**
Hyphae and hair roots	Eudorylaimus	**6.344**	**-0.166**	**0.000**	**4.731**
Hyphae and hair roots	Prodorylaimus	**6.344**	**-0.836**	**0.000**	**4.731**
Hyphae and hair roots	Qudsianematidae	**6.344**	**-0.207**	**0.000**	**4.430**
Hyphae and hair roots	Thornematidae	**6.344**	**-0.470**	**0.000**	**4.907**
Hyphae and hair roots	Eupodes	**6.344**	**0.005**	**0.000**	**4.263**
Hyphae and hair roots	Humerobates	**6.344**	**0.584**	**0.000**	**3.263**
Hyphae and hair roots	Oribatida	**6.344**	**-0.411**	**0.000**	**4.041**
Hyphae and hair roots	Scutacarus	**6.344**	**-0.608**	**0.000**	**3.740**
Hyphae and hair roots	Stigmaeidae	**6.344**	**0.229**	**0.000**	**3.564**
Mononchidae	Lysigamasus	**-0.827**	**0.407**	**4.430**	**3.564**
Mononchidae	Aporcelaimellus	**-0.827**	**0.548**	**4.430**	**5.129**
Mononchidae	Dorylaimoidea	**-0.827**	**-0.604**	**4.430**	**5.544**
Mononchidae	Eudorylaimus	**-0.827**	**-0.166**	**4.430**	**4.731**
Mononchidae	Prodorylaimus	**-0.827**	**-0.836**	**4.430**	**4.731**
Mononchidae	Qudsianematidae	**-0.827**	**-0.207**	**4.430**	**4.430**
Mononchidae	Thornematidae	**-0.827**	**-0.470**	**4.430**	**4.907**
Mononchidae	Eupodes	**-0.827**	**0.005**	**4.430**	**4.263**
Mononchidae	Humerobates	**-0.827**	**0.584**	**4.430**	**3.263**
Mononchidae	Oribatida	**-0.827**	**-0.411**	**4.430**	**4.041**
Mononchidae	Scutacarus	**-0.827**	**-0.608**	**4.430**	**3.740**
Mononchidae	Stigmaeidae	**-0.827**	**0.229**	**4.430**	**3.564**
Mylonchulus	Lysigamasus	**-0.005**	**0.407**	**4.430**	**3.564**
Mylonchulus	Aporcelaimellus	**-0.005**	**0.548**	**4.430**	**5.129**
Mylonchulus	Dorylaimoidea	**-0.005**	**-0.604**	**4.430**	**5.544**
Mylonchulus	Eudorylaimus	**-0.005**	**-0.166**	**4.430**	**4.731**
Mylonchulus	Prodorylaimus	**-0.005**	**-0.836**	**4.430**	**4.731**
Mylonchulus	Qudsianematidae	**-0.005**	**-0.207**	**4.430**	**4.430**
Mylonchulus	Thornematidae	**-0.005**	**-0.470**	**4.430**	**4.907**
Mylonchulus	Eupodes	**-0.005**	**0.005**	**4.430**	**4.263**
Mylonchulus	Humerobates	**-0.005**	**0.584**	**4.430**	**3.263**
Mylonchulus	Oribatida	**-0.005**	**-0.411**	**4.430**	**4.041**
Mylonchulus	Scutacarus	**-0.005**	**-0.608**	**4.430**	**3.740**
Mylonchulus	Stigmaeidae	**-0.005**	**0.229**	**4.430**	**3.564**
Lysigamasus	Aporcelaimellus	**0.407**	**0.548**	**3.564**	**5.129**
Lysigamasus	Dorylaimoidea	**0.407**	**-0.604**	**3.564**	**5.544**
Lysigamasus	Eudorylaimus	**0.407**	**-0.166**	**3.564**	**4.731**
Lysigamasus	Prodorylaimus	**0.407**	**-0.836**	**3.564**	**4.731**
Lysigamasus	Qudsianematidae	**0.407**	**-0.207**	**3.564**	**4.430**
Lysigamasus	Thornematidae	**0.407**	**-0.470**	**3.564**	**4.907**
Lysigamasus	Eupodes	**0.407**	**0.005**	**3.564**	**4.263**

Lysigamasus	Humerobates	**0.407**	**0.584**	**3.564**	**3.263**
Lysigamasus	Oribatida	**0.407**	**-0.411**	**3.564**	**4.041**
Lysigamasus	Scutacarus	**0.407**	**-0.608**	**3.564**	**3.740**
Lysigamasus	Stigmaeidae	**0.407**	**0.229**	**3.564**	**3.564**
Lysigamasus	Trombidiidae	**0.407**	**1.787**	**3.564**	**3.263**
Aporcelaimellus	Mononchidae	**0.548**	**-0.827**	**5.129**	**4.430**
Aporcelaimellus	Mylonchulus	**0.548**	**-0.005**	**5.129**	**4.430**
Aporcelaimellus	Lysigamasus	**0.548**	**0.407**	**5.129**	**3.564**
Aporcelaimellus	Aporcelaimellus	**0.548**	**0.548**	**5.129**	**5.129**
Aporcelaimellus	Dorylaimoidea	**0.548**	**-0.604**	**5.129**	**5.544**
Aporcelaimellus	Eudorylaimus	**0.548**	**-0.166**	**5.129**	**4.731**
Aporcelaimellus	Prodorylaimus	**0.548**	**-0.836**	**5.129**	**4.731**
Aporcelaimellus	Qudsianematidae	**0.548**	**-0.207**	**5.129**	**4.430**
Aporcelaimellus	Thornematidae	**0.548**	**-0.470**	**5.129**	**4.907**
Aporcelaimellus	Eupodes	**0.548**	**0.005**	**5.129**	**4.263**
Aporcelaimellus	Humerobates	**0.548**	**0.584**	**5.129**	**3.263**
Aporcelaimellus	Oribatida	**0.548**	**-0.411**	**5.129**	**4.041**
Aporcelaimellus	Scutacarus	**0.548**	**-0.608**	**5.129**	**3.740**
Aporcelaimellus	Stigmaeidae	**0.548**	**0.229**	**5.129**	**3.564**
Dorylaimoidea	Mononchidae	**-0.604**	**-0.827**	**5.544**	**4.430**
Dorylaimoidea	Mylonchulus	**-0.604**	**-0.005**	**5.544**	**4.430**
Dorylaimoidea	Lysigamasus	**-0.604**	**0.407**	**5.544**	**3.564**
Dorylaimoidea	Aporcelaimellus	**-0.604**	**0.548**	**5.544**	**5.129**
Dorylaimoidea	Dorylaimoidea	**-0.604**	**-0.604**	**5.544**	**5.544**
Dorylaimoidea	Eudorylaimus	**-0.604**	**-0.166**	**5.544**	**4.731**
Dorylaimoidea	Prodorylaimus	**-0.604**	**-0.836**	**5.544**	**4.731**
Dorylaimoidea	Qudsianematidae	**-0.604**	**-0.207**	**5.544**	**4.430**
Dorylaimoidea	Thornematidae	**-0.604**	**-0.470**	**5.544**	**4.907**
Dorylaimoidea	Eupodes	**-0.604**	**0.005**	**5.544**	**4.263**
Dorylaimoidea	Humerobates	**-0.604**	**0.584**	**5.544**	**3.263**
Dorylaimoidea	Oribatida	**-0.604**	**-0.411**	**5.544**	**4.041**
Dorylaimoidea	Scutacarus	**-0.604**	**-0.608**	**5.544**	**3.740**
Dorylaimoidea	Stigmaeidae	**-0.604**	**0.229**	**5.544**	**3.564**
Eudorylaimus	Mononchidae	**-0.166**	**-0.827**	**4.731**	**4.430**
Eudorylaimus	Mylonchulus	**-0.166**	**-0.005**	**4.731**	**4.430**
Eudorylaimus	Lysigamasus	**-0.166**	**0.407**	**4.731**	**3.564**
Eudorylaimus	Aporcelaimellus	**-0.166**	**0.548**	**4.731**	**5.129**
Eudorylaimus	Dorylaimoidea	**-0.166**	**-0.604**	**4.731**	**5.544**
Eudorylaimus	Eudorylaimus	**-0.166**	**-0.166**	**4.731**	**4.731**
Eudorylaimus	Prodorylaimus	**-0.166**	**-0.836**	**4.731**	**4.731**
Eudorylaimus	Qudsianematidae	**-0.166**	**-0.207**	**4.731**	**4.430**
Eudorylaimus	Thornematidae	**-0.166**	**-0.470**	**4.731**	**4.907**
Eudorylaimus	Eupodes	**-0.166**	**0.005**	**4.731**	**4.263**
Eudorylaimus	Humerobates	**-0.166**	**0.584**	**4.731**	**3.263**
Eudorylaimus	Oribatida	**-0.166**	**-0.411**	**4.731**	**4.041**
Eudorylaimus	Scutacarus	**-0.166**	**-0.608**	**4.731**	**3.740**
Eudorylaimus	Stigmaeidae	**-0.166**	**0.229**	**4.731**	**3.564**
Prodorylaimus	Mononchidae	**-0.836**	**-0.827**	**4.731**	**4.430**
Prodorylaimus	Mylonchulus	**-0.836**	**-0.005**	**4.731**	**4.430**
Prodorylaimus	Lysigamasus	**-0.836**	**0.407**	**4.731**	**3.564**
Prodorylaimus	Aporcelaimellus	**-0.836**	**0.548**	**4.731**	**5.129**

Prodorylaimus	Dorylaimoidea	-0.836	-0.604	4.731	5.544
Prodorylaimus	Eudorylaimus	-0.836	-0.166	4.731	4.731
Prodorylaimus	Prodorylaimus	-0.836	-0.836	4.731	4.731
Prodorylaimus	Qudsianematidae	-0.836	-0.207	4.731	4.430
Prodorylaimus	Thornematidae	-0.836	-0.470	4.731	4.907
Prodorylaimus	Eupodes	-0.836	0.005	4.731	4.263
Prodorylaimus	Humerobates	-0.836	0.584	4.731	3.263
Prodorylaimus	Oribatida	-0.836	-0.411	4.731	4.041
Prodorylaimus	Scutacarus	-0.836	-0.608	4.731	3.740
Prodorylaimus	Stigmaeidae	-0.836	0.229	4.731	3.564
Qudsianematidae	Mononchidae	-0.207	-0.827	4.430	4.430
Qudsianematidae	Mylonchulus	-0.207	-0.005	4.430	4.430
Qudsianematidae	Lysigamasus	-0.207	0.407	4.430	3.564
Qudsianematidae	Aporcelaimellus	-0.207	0.548	4.430	5.129
Qudsianematidae	Dorylaimoidea	-0.207	-0.604	4.430	5.544
Qudsianematidae	Eudorylaimus	-0.207	-0.166	4.430	4.731
Qudsianematidae	Prodorylaimus	-0.207	-0.836	4.430	4.731
Qudsianematidae	Qudsianematidae	-0.207	-0.207	4.430	4.430
Qudsianematidae	Thornematidae	-0.207	-0.470	4.430	4.907
Qudsianematidae	Eupodes	-0.207	0.005	4.430	4.263
Qudsianematidae	Humerobates	-0.207	0.584	4.430	3.263
Qudsianematidae	Oribatida	-0.207	-0.411	4.430	4.041
Qudsianematidae	Scutacarus	-0.207	-0.608	4.430	3.740
Qudsianematidae	Stigmaeidae	-0.207	0.229	4.430	3.564
Thornematidae	Mononchidae	-0.470	-0.827	4.907	4.430
Thornematidae	Mylonchulus	-0.470	-0.005	4.907	4.430
Thornematidae	Lysigamasus	-0.470	0.407	4.907	3.564
Thornematidae	Aporcelaimellus	-0.470	0.548	4.907	5.129
Thornematidae	Dorylaimoidea	-0.470	-0.604	4.907	5.544
Thornematidae	Eudorylaimus	-0.470	-0.166	4.907	4.731
Thornematidae	Prodorylaimus	-0.470	-0.836	4.907	4.731
Thornematidae	Qudsianematidae	-0.470	-0.207	4.907	4.430
Thornematidae	Thornematidae	-0.470	-0.470	4.907	4.907
Thornematidae	Eupodes	-0.470	0.005	4.907	4.263
Thornematidae	Humerobates	-0.470	0.584	4.907	3.263
Thornematidae	Oribatida	-0.470	-0.411	4.907	4.041
Thornematidae	Scutacarus	-0.470	-0.608	4.907	3.740
Thornematidae	Stigmaeidae	-0.470	0.229	4.907	3.564
Eupodes	Lysigamasus	0.005	0.407	4.263	3.564
Eupodes	Aporcelaimellus	0.005	0.548	4.263	5.129
Eupodes	Dorylaimoidea	0.005	-0.604	4.263	5.544
Eupodes	Eudorylaimus	0.005	-0.166	4.263	4.731
Eupodes	Prodorylaimus	0.005	-0.836	4.263	4.731
Eupodes	Qudsianematidae	0.005	-0.207	4.263	4.430
Eupodes	Thornematidae	0.005	-0.470	4.263	4.907
Eupodes	Eupodes	0.005	0.005	4.263	4.263
Eupodes	Humerobates	0.005	0.584	4.263	3.263
Eupodes	Oribatida	0.005	-0.411	4.263	4.041
Eupodes	Scutacarus	0.005	-0.608	4.263	3.740
Eupodes	Stigmaeidae	0.005	0.229	4.263	3.564
Humerobates	Lysigamasus	0.584	0.407	3.263	3.564

Humerobates	Aporcelaimellus	**0.584**	**0.548**	**3.263**	**5.129**
Humerobates	Dorylaimoidea	**0.584**	**-0.604**	**3.263**	**5.544**
Humerobates	Eudorylaimus	**0.584**	**-0.166**	**3.263**	**4.731**
Humerobates	Prodorylaimus	**0.584**	**-0.836**	**3.263**	**4.731**
Humerobates	Qudsianematidae	**0.584**	**-0.207**	**3.263**	**4.430**
Humerobates	Thornematidae	**0.584**	**-0.470**	**3.263**	**4.907**
Humerobates	Eupodes	**0.584**	**0.005**	**3.263**	**4.263**
Humerobates	Humerobates	**0.584**	**0.584**	**3.263**	**3.263**
Humerobates	Oribatida	**0.584**	**-0.411**	**3.263**	**4.041**
Humerobates	Scutacarus	**0.584**	**-0.608**	**3.263**	**3.740**
Humerobates	Stigmaeidae	**0.584**	**0.229**	**3.263**	**3.564**
Oribatida	Lysigamasus	**-0.411**	**0.407**	**4.041**	**3.564**
Oribatida	Aporcelaimellus	**-0.411**	**0.548**	**4.041**	**5.129**
Oribatida	Dorylaimoidea	**-0.411**	**-0.604**	**4.041**	**5.544**
Oribatida	Eudorylaimus	**-0.411**	**-0.166**	**4.041**	**4.731**
Oribatida	Prodorylaimus	**-0.411**	**-0.836**	**4.041**	**4.731**
Oribatida	Qudsianematidae	**-0.411**	**-0.207**	**4.041**	**4.430**
Oribatida	Thornematidae	**-0.411**	**-0.470**	**4.041**	**4.907**
Oribatida	Eupodes	**-0.411**	**0.005**	**4.041**	**4.263**
Oribatida	Humerobates	**-0.411**	**0.584**	**4.041**	**3.263**
Oribatida	Oribatida	**-0.411**	**-0.411**	**4.041**	**4.041**
Oribatida	Scutacarus	**-0.411**	**-0.608**	**4.041**	**3.740**
Oribatida	Stigmaeidae	**-0.411**	**0.229**	**4.041**	**3.564**
Scutacarus	Lysigamasus	**-0.608**	**0.407**	**3.740**	**3.564**
Scutacarus	Aporcelaimellus	**-0.608**	**0.548**	**3.740**	**5.129**
Scutacarus	Dorylaimoidea	**-0.608**	**-0.604**	**3.740**	**5.544**
Scutacarus	Eudorylaimus	**-0.608**	**-0.166**	**3.740**	**4.731**
Scutacarus	Prodorylaimus	**-0.608**	**-0.836**	**3.740**	**4.731**
Scutacarus	Qudsianematidae	**-0.608**	**-0.207**	**3.740**	**4.430**
Scutacarus	Thornematidae	**-0.608**	**-0.470**	**3.740**	**4.907**
Scutacarus	Eupodes	**-0.608**	**0.005**	**3.740**	**4.263**
Scutacarus	Humerobates	**-0.608**	**0.584**	**3.740**	**3.263**
Scutacarus	Oribatida	**-0.608**	**-0.411**	**3.740**	**4.041**
Scutacarus	Scutacarus	**-0.608**	**-0.608**	**3.740**	**3.740**
Scutacarus	Stigmaeidae	**-0.608**	**0.229**	**3.740**	**3.564**
Stigmaeidae	Lysigamasus	**0.229**	**0.407**	**3.564**	**3.564**
Stigmaeidae	Aporcelaimellus	**0.229**	**0.548**	**3.564**	**5.129**
Stigmaeidae	Dorylaimoidea	**0.229**	**-0.604**	**3.564**	**5.544**
Stigmaeidae	Eudorylaimus	**0.229**	**-0.166**	**3.564**	**4.731**
Stigmaeidae	Prodorylaimus	**0.229**	**-0.836**	**3.564**	**4.731**
Stigmaeidae	Qudsianematidae	**0.229**	**-0.207**	**3.564**	**4.430**
Stigmaeidae	Thornematidae	**0.229**	**-0.470**	**3.564**	**4.907**
Stigmaeidae	Eupodes	**0.229**	**0.005**	**3.564**	**4.263**
Stigmaeidae	Humerobates	**0.229**	**0.584**	**3.564**	**3.263**
Stigmaeidae	Oribatida	**0.229**	**-0.411**	**3.564**	**4.041**
Stigmaeidae	Scutacarus	**0.229**	**-0.608**	**3.564**	**3.740**
Stigmaeidae	Stigmaeidae	**0.229**	**0.229**	**3.564**	**3.564**
Trombidiidae	Eupodes	**1.787**	**0.005**	**3.263**	**4.263**
Trombidiidae	Humerobates	**1.787**	**0.584**	**3.263**	**3.263**
Trombidiidae	Oribatida	**1.787**	**-0.411**	**3.263**	**4.041**
Trombidiidae	Scutacarus	**1.787**	**-0.608**	**3.263**	**3.740**

Trombidiidae	Stigmaeidae	**1.787**	**0.229**	**3.263**	**3.564**

Resource	Consumer	Mres	Mconsumer	Nres	Nconsumer
Aglenchus	Seinura	**-1.053**	**-0.814**	**4.649**	**4.348**
Aglenchus	Tripyla	**-1.053**	**-0.420**	**4.649**	**4.649**
Aglenchus	Arctoseius	**-1.053**	**-0.152**	**4.649**	**3.485**
Aglenchus	Cheiroseius	**-1.053**	**0.356**	**4.649**	**3.184**
Aglenchus	Aporcelaimellus	**-1.053**	**0.548**	**4.649**	**5.251**
Aglenchus	Dorylaimoidea	**-1.053**	**-0.604**	**4.649**	**4.825**
Aglenchus	Epidorylaimus	**-1.053**	**0.199**	**4.649**	**4.348**
Aglenchus	Qudsianematidae	**-1.053**	**-0.207**	**4.649**	**4.825**
Aglenchus	Thornematidae	**-1.053**	**-0.470**	**4.649**	**4.348**
Aglenchus	Eupodes	**-1.053**	**0.005**	**4.649**	**3.786**
Aglenchus	Scheloribates	**-1.053**	**0.202**	**4.649**	**3.184**
Aglenchus	Scutacarus	**-1.053**	**-0.608**	**4.649**	**3.962**
Aglenchus	Tarsonemus	**-1.053**	**-0.701**	**4.649**	**3.485**
Coslenchus	Seinura	**-0.821**	**-0.814**	**4.348**	**4.348**
Coslenchus	Tripyla	**-0.821**	**-0.420**	**4.348**	**4.649**
Coslenchus	Arctoseius	**-0.821**	**-0.152**	**4.348**	**3.485**
Coslenchus	Cheiroseius	**-0.821**	**0.356**	**4.348**	**3.184**
Coslenchus	Aporcelaimellus	**-0.821**	**0.548**	**4.348**	**5.251**
Coslenchus	Dorylaimoidea	**-0.821**	**-0.604**	**4.348**	**4.825**
Coslenchus	Epidorylaimus	**-0.821**	**0.199**	**4.348**	**4.348**
Coslenchus	Qudsianematidae	**-0.821**	**-0.207**	**4.348**	**4.825**
Coslenchus	Thornematidae	**-0.821**	**-0.470**	**4.348**	**4.348**
Coslenchus	Eupodes	**-0.821**	**0.005**	**4.348**	**3.786**
Coslenchus	Scheloribates	**-0.821**	**0.202**	**4.348**	**3.184**
Coslenchus	Scutacarus	**-0.821**	**-0.608**	**4.348**	**3.962**
Coslenchus	Tarsonemus	**-0.821**	**-0.701**	**4.348**	**3.485**
Dolichodoridae	Seinura	**-0.885**	**-0.814**	**5.047**	**4.348**
Dolichodoridae	Tripyla	**-0.885**	**-0.420**	**5.047**	**4.649**
Dolichodoridae	Arctoseius	**-0.885**	**-0.152**	**5.047**	**3.485**
Dolichodoridae	Cheiroseius	**-0.885**	**0.356**	**5.047**	**3.184**
Dolichodoridae	Aporcelaimellus	**-0.885**	**0.548**	**5.047**	**5.251**
Dolichodoridae	Dorylaimoidea	**-0.885**	**-0.604**	**5.047**	**4.825**
Dolichodoridae	Epidorylaimus	**-0.885**	**0.199**	**5.047**	**4.348**
Dolichodoridae	Qudsianematidae	**-0.885**	**-0.207**	**5.047**	**4.825**
Dolichodoridae	Thornematidae	**-0.885**	**-0.470**	**5.047**	**4.348**
Dolichodoridae	Eupodes	**-0.885**	**0.005**	**5.047**	**3.786**
Dolichodoridae	Scheloribates	**-0.885**	**0.202**	**5.047**	**3.184**
Dolichodoridae	Scutacarus	**-0.885**	**-0.608**	**5.047**	**3.962**
Dolichodoridae	Tarsonemus	**-0.885**	**-0.701**	**5.047**	**3.485**
Filenchus	Seinura	**-1.033**	**-0.814**	**4.348**	**4.348**
Filenchus	Tripyla	**-1.033**	**-0.420**	**4.348**	**4.649**
Filenchus	Arctoseius	**-1.033**	**-0.152**	**4.348**	**3.485**
Filenchus	Cheiroseius	**-1.033**	**0.356**	**4.348**	**3.184**
Filenchus	Aporcelaimellus	**-1.033**	**0.548**	**4.348**	**5.251**
Filenchus	Dorylaimoidea	**-1.033**	**-0.604**	**4.348**	**4.825**
Filenchus	Epidorylaimus	**-1.033**	**0.199**	**4.348**	**4.348**
Filenchus	Qudsianematidae	**-1.033**	**-0.207**	**4.348**	**4.825**
Filenchus	Thornematidae	**-1.033**	**-0.470**	**4.348**	**4.348**
Filenchus	Eupodes	**-1.033**	**0.005**	**4.348**	**3.786**
Filenchus	Scheloribates	**-1.033**	**0.202**	**4.348**	**3.184**

Filenchus	Scutacarus	**-1.033**	**-0.608**	**4.348**	**3.962**
Filenchus	Tarsonemus	**-1.033**	**-0.701**	**4.348**	**3.485**
Helicotylenchus	Seinura	**-0.792**	**-0.814**	**4.649**	**4.348**
Helicotylenchus	Tripyla	**-0.792**	**-0.420**	**4.649**	**4.649**
Helicotylenchus	Arctoseius	**-0.792**	**-0.152**	**4.649**	**3.485**
Helicotylenchus	Cheiroseius	**-0.792**	**0.356**	**4.649**	**3.184**
Helicotylenchus	Aporcelaimellus	**-0.792**	**0.548**	**4.649**	**5.251**
Helicotylenchus	Dorylaimoidea	**-0.792**	**-0.604**	**4.649**	**4.825**
Helicotylenchus	Epidorylaimus	**-0.792**	**0.199**	**4.649**	**4.348**
Helicotylenchus	Qudsianematidae	**-0.792**	**-0.207**	**4.649**	**4.825**
Helicotylenchus	Thornematidae	**-0.792**	**-0.470**	**4.649**	**4.348**
Helicotylenchus	Eupodes	**-0.792**	**0.005**	**4.649**	**3.786**
Helicotylenchus	Scheloribates	**-0.792**	**0.202**	**4.649**	**3.184**
Helicotylenchus	Scutacarus	**-0.792**	**-0.608**	**4.649**	**3.962**
Helicotylenchus	Tarsonemus	**-0.792**	**-0.701**	**4.649**	**3.485**
Malenchus	Seinura	**-1.330**	**-0.814**	**4.348**	**4.348**
Malenchus	Tripyla	**-1.330**	**-0.420**	**4.348**	**4.649**
Malenchus	Arctoseius	**-1.330**	**-0.152**	**4.348**	**3.485**
Malenchus	Cheiroseius	**-1.330**	**0.356**	**4.348**	**3.184**
Malenchus	Aporcelaimellus	**-1.330**	**0.548**	**4.348**	**5.251**
Malenchus	Dorylaimoidea	**-1.330**	**-0.604**	**4.348**	**4.825**
Malenchus	Epidorylaimus	**-1.330**	**0.199**	**4.348**	**4.348**
Malenchus	Qudsianematidae	**-1.330**	**-0.207**	**4.348**	**4.825**
Malenchus	Thornematidae	**-1.330**	**-0.470**	**4.348**	**4.348**
Malenchus	Eupodes	**-1.330**	**0.005**	**4.348**	**3.786**
Malenchus	Scheloribates	**-1.330**	**0.202**	**4.348**	**3.184**
Malenchus	Scutacarus	**-1.330**	**-0.608**	**4.348**	**3.962**
Malenchus	Tarsonemus	**-1.330**	**-0.701**	**4.348**	**3.485**
Meloidogyne	Seinura	**-1.287**	**-0.814**	**4.348**	**4.348**
Meloidogyne	Tripyla	**-1.287**	**-0.420**	**4.348**	**4.649**
Meloidogyne	Arctoseius	**-1.287**	**-0.152**	**4.348**	**3.485**
Meloidogyne	Cheiroseius	**-1.287**	**0.356**	**4.348**	**3.184**
Meloidogyne	Aporcelaimellus	**-1.287**	**0.548**	**4.348**	**5.251**
Meloidogyne	Dorylaimoidea	**-1.287**	**-0.604**	**4.348**	**4.825**
Meloidogyne	Epidorylaimus	**-1.287**	**0.199**	**4.348**	**4.348**
Meloidogyne	Qudsianematidae	**-1.287**	**-0.207**	**4.348**	**4.825**
Meloidogyne	Thornematidae	**-1.287**	**-0.470**	**4.348**	**4.348**
Meloidogyne	Eupodes	**-1.287**	**0.005**	**4.348**	**3.786**
Meloidogyne	Scheloribates	**-1.287**	**0.202**	**4.348**	**3.184**
Meloidogyne	Scutacarus	**-1.287**	**-0.608**	**4.348**	**3.962**
Meloidogyne	Tarsonemus	**-1.287**	**-0.701**	**4.348**	**3.485**
Paratylenchus	Seinura	**-1.244**	**-0.814**	**4.348**	**4.348**
Paratylenchus	Tripyla	**-1.244**	**-0.420**	**4.348**	**4.649**
Paratylenchus	Arctoseius	**-1.244**	**-0.152**	**4.348**	**3.485**
Paratylenchus	Cheiroseius	**-1.244**	**0.356**	**4.348**	**3.184**
Paratylenchus	Aporcelaimellus	**-1.244**	**0.548**	**4.348**	**5.251**
Paratylenchus	Dorylaimoidea	**-1.244**	**-0.604**	**4.348**	**4.825**
Paratylenchus	Epidorylaimus	**-1.244**	**0.199**	**4.348**	**4.348**
Paratylenchus	Qudsianematidae	**-1.244**	**-0.207**	**4.348**	**4.825**
Paratylenchus	Thornematidae	**-1.244**	**-0.470**	**4.348**	**4.348**
Paratylenchus	Eupodes	**-1.244**	**0.005**	**4.348**	**3.786**

Paratylenchus	Scheloribates	**-1.244**	**0.202**	**4.348**	**3.184**
Paratylenchus	Scutacarus	**-1.244**	**-0.608**	**4.348**	**3.962**
Paratylenchus	Tarsonemus	**-1.244**	**-0.701**	**4.348**	**3.485**
Pratylenchus	Seinura	**-1.226**	**-0.814**	**4.348**	**4.348**
Pratylenchus	Tripyla	**-1.226**	**-0.420**	**4.348**	**4.649**
Pratylenchus	Arctoseius	**-1.226**	**-0.152**	**4.348**	**3.485**
Pratylenchus	Cheiroseius	**-1.226**	**0.356**	**4.348**	**3.184**
Pratylenchus	Aporcelaimellus	**-1.226**	**0.548**	**4.348**	**5.251**
Pratylenchus	Dorylaimoidea	**-1.226**	**-0.604**	**4.348**	**4.825**
Pratylenchus	Epidorylaimus	**-1.226**	**0.199**	**4.348**	**4.348**
Pratylenchus	Qudsianematidae	**-1.226**	**-0.207**	**4.348**	**4.825**
Pratylenchus	Thornematidae	**-1.226**	**-0.470**	**4.348**	**4.348**
Pratylenchus	Eupodes	**-1.226**	**0.005**	**4.348**	**3.786**
Pratylenchus	Scheloribates	**-1.226**	**0.202**	**4.348**	**3.184**
Pratylenchus	Scutacarus	**-1.226**	**-0.608**	**4.348**	**3.962**
Pratylenchus	Tarsonemus	**-1.226**	**-0.701**	**4.348**	**3.485**
Trichodorus	Seinura	**-0.744**	**-0.814**	**5.302**	**4.348**
Trichodorus	Tripyla	**-0.744**	**-0.420**	**5.302**	**4.649**
Trichodorus	Arctoseius	**-0.744**	**-0.152**	**5.302**	**3.485**
Trichodorus	Cheiroseius	**-0.744**	**0.356**	**5.302**	**3.184**
Trichodorus	Aporcelaimellus	**-0.744**	**0.548**	**5.302**	**5.251**
Trichodorus	Dorylaimoidea	**-0.744**	**-0.604**	**5.302**	**4.825**
Trichodorus	Epidorylaimus	**-0.744**	**0.199**	**5.302**	**4.348**
Trichodorus	Qudsianematidae	**-0.744**	**-0.207**	**5.302**	**4.825**
Trichodorus	Thornematidae	**-0.744**	**-0.470**	**5.302**	**4.348**
Trichodorus	Eupodes	**-0.744**	**0.005**	**5.302**	**3.786**
Trichodorus	Scheloribates	**-0.744**	**0.202**	**5.302**	**3.184**
Trichodorus	Scutacarus	**-0.744**	**-0.608**	**5.302**	**3.962**
Trichodorus	Tarsonemus	**-0.744**	**-0.701**	**5.302**	**3.485**
Tylenchorhynchus	Seinura	**-0.664**	**-0.814**	**5.462**	**4.348**
Tylenchorhynchus	Tripyla	**-0.664**	**-0.420**	**5.462**	**4.649**
Tylenchorhynchus	Arctoseius	**-0.664**	**-0.152**	**5.462**	**3.485**
Tylenchorhynchus	Cheiroseius	**-0.664**	**0.356**	**5.462**	**3.184**
Tylenchorhynchus	Aporcelaimellus	**-0.664**	**0.548**	**5.462**	**5.251**
Tylenchorhynchus	Dorylaimoidea	**-0.664**	**-0.604**	**5.462**	**4.825**
Tylenchorhynchus	Epidorylaimus	**-0.664**	**0.199**	**5.462**	**4.348**
Tylenchorhynchus	Qudsianematidae	**-0.664**	**-0.207**	**5.462**	**4.825**
Tylenchorhynchus	Thornematidae	**-0.664**	**-0.470**	**5.462**	**4.348**
Tylenchorhynchus	Eupodes	**-0.664**	**0.005**	**5.462**	**3.786**
Tylenchorhynchus	Scheloribates	**-0.664**	**0.202**	**5.462**	**3.184**
Tylenchorhynchus	Scutacarus	**-0.664**	**-0.608**	**5.462**	**3.962**
Tylenchorhynchus	Tarsonemus	**-0.664**	**-0.701**	**5.462**	**3.485**
Pachygnatidae	Arctoseius	**-0.113**	**-0.152**	**3.661**	**3.485**
Pachygnatidae	Cheiroseius	**-0.113**	**0.356**	**3.661**	**3.184**
Pachygnatidae	Aporcelaimellus	**-0.113**	**0.548**	**3.661**	**5.251**
Pachygnatidae	Dorylaimoidea	**-0.113**	**-0.604**	**3.661**	**4.825**
Pachygnatidae	Epidorylaimus	**-0.113**	**0.199**	**3.661**	**4.348**
Pachygnatidae	Qudsianematidae	**-0.113**	**-0.207**	**3.661**	**4.825**
Pachygnatidae	Thornematidae	**-0.113**	**-0.470**	**3.661**	**4.348**
Pachygnatidae	Eupodes	**-0.113**	**0.005**	**3.661**	**3.786**
Pachygnatidae	Scheloribates	**-0.113**	**0.202**	**3.661**	**3.184**

Pachygnatidae	Scutacarus	**-0.113**	**-0.608**	**3.661**	**3.962**
Pachygnatidae	Tarsonemus	**-0.113**	**-0.701**	**3.661**	**3.485**
Platynothrus	Arctoseius	**0.710**	**-0.152**	**3.485**	**3.485**
Platynothrus	Cheiroseius	**0.710**	**0.356**	**3.485**	**3.184**
Platynothrus	Aporcelaimellus	**0.710**	**0.548**	**3.485**	**5.251**
Platynothrus	Dorylaimoidea	**0.710**	**-0.604**	**3.485**	**4.825**
Platynothrus	Epidorylaimus	**0.710**	**0.199**	**3.485**	**4.348**
Platynothrus	Qudsianematidae	**0.710**	**-0.207**	**3.485**	**4.825**
Platynothrus	Thornematidae	**0.710**	**-0.470**	**3.485**	**4.348**
Platynothrus	Eupodes	**0.710**	**0.005**	**3.485**	**3.786**
Platynothrus	Scheloribates	**0.710**	**0.202**	**3.485**	**3.184**
Platynothrus	Scutacarus	**0.710**	**-0.608**	**3.485**	**3.962**
Platynothrus	Tarsonemus	**0.710**	**-0.701**	**3.485**	**3.485**
Tydeidae	Arctoseius	**-0.608**	**-0.152**	**3.184**	**3.485**
Tydeidae	Cheiroseius	**-0.608**	**0.356**	**3.184**	**3.184**
Tydeidae	Aporcelaimellus	**-0.608**	**0.548**	**3.184**	**5.251**
Tydeidae	Dorylaimoidea	**-0.608**	**-0.604**	**3.184**	**4.825**
Tydeidae	Epidorylaimus	**-0.608**	**0.199**	**3.184**	**4.348**
Tydeidae	Qudsianematidae	**-0.608**	**-0.207**	**3.184**	**4.825**
Tydeidae	Thornematidae	**-0.608**	**-0.470**	**3.184**	**4.348**
Tydeidae	Eupodes	**-0.608**	**0.005**	**3.184**	**3.786**
Tydeidae	Scheloribates	**-0.608**	**0.202**	**3.184**	**3.184**
Tydeidae	Scutacarus	**-0.608**	**-0.608**	**3.184**	**3.962**
Tydeidae	Tarsonemus	**-0.608**	**-0.701**	**3.184**	**3.485**
Sminthuridae	Arctoseius	**-0.608**	**-0.152**	**3.786**	**3.485**
Sminthuridae	Cheiroseius	**-0.608**	**0.356**	**3.786**	**3.184**
Sminthuridae	Aporcelaimellus	**-0.608**	**0.548**	**3.786**	**5.251**
Sminthuridae	Dorylaimoidea	**-0.608**	**-0.604**	**3.786**	**4.825**
Sminthuridae	Epidorylaimus	**-0.608**	**0.199**	**3.786**	**4.348**
Sminthuridae	Qudsianematidae	**-0.608**	**-0.207**	**3.786**	**4.825**
Sminthuridae	Thornematidae	**-0.608**	**-0.470**	**3.786**	**4.348**
Sminthuridae	Eupodes	**-0.608**	**0.005**	**3.786**	**3.786**
Sminthuridae	Scheloribates	**-0.608**	**0.202**	**3.786**	**3.184**
Sminthuridae	Scutacarus	**-0.608**	**-0.608**	**3.786**	**3.962**
Sminthuridae	Tarsonemus	**-0.608**	**-0.701**	**3.786**	**3.485**
Sminthurinus	Arctoseius	**0.618**	**-0.152**	**3.485**	**3.485**
Sminthurinus	Cheiroseius	**0.618**	**0.356**	**3.485**	**3.184**
Sminthurinus	Aporcelaimellus	**0.618**	**0.548**	**3.485**	**5.251**
Sminthurinus	Dorylaimoidea	**0.618**	**-0.604**	**3.485**	**4.825**
Sminthurinus	Epidorylaimus	**0.618**	**0.199**	**3.485**	**4.348**
Sminthurinus	Qudsianematidae	**0.618**	**-0.207**	**3.485**	**4.825**
Sminthurinus	Thornematidae	**0.618**	**-0.470**	**3.485**	**4.348**
Sminthurinus	Eupodes	**0.618**	**0.005**	**3.485**	**3.786**
Sminthurinus	Scheloribates	**0.618**	**0.202**	**3.485**	**3.184**
Sminthurinus	Scutacarus	**0.618**	**-0.608**	**3.485**	**3.962**
Sminthurinus	Tarsonemus	**0.618**	**-0.701**	**3.485**	**3.485**
Aphelenchoides	Seinura	**-1.496**	**-0.814**	**5.126**	**4.348**
Aphelenchoides	Tripyla	**-1.496**	**-0.420**	**5.126**	**4.649**
Aphelenchoides	Arctoseius	**-1.496**	**-0.152**	**5.126**	**3.485**
Aphelenchoides	Cheiroseius	**-1.496**	**0.356**	**5.126**	**3.184**
Aphelenchoides	Aporcelaimellus	**-1.496**	**0.548**	**5.126**	**5.251**

Aphelenchoides	Dorylaimoidea	**-1.496**	**-0.604**	**5.126**	**4.825**
Aphelenchoides	Epidorylaimus	**-1.496**	**0.199**	**5.126**	**4.348**
Aphelenchoides	Qudsianematidae	**-1.496**	**-0.207**	**5.126**	**4.825**
Aphelenchoides	Thornematidae	**-1.496**	**-0.470**	**5.126**	**4.348**
Aphelenchoides	Eupodes	**-1.496**	**0.005**	**5.126**	**3.786**
Aphelenchoides	Scheloribates	**-1.496**	**0.202**	**5.126**	**3.184**
Aphelenchoides	Scutacarus	**-1.496**	**-0.608**	**5.126**	**3.962**
Aphelenchoides	Tarsonemus	**-1.496**	**-0.701**	**5.126**	**3.485**
Tylenchidae	Seinura	**-1.360**	**-0.814**	**5.524**	**4.348**
Tylenchidae	Tripyla	**-1.360**	**-0.420**	**5.524**	**4.649**
Tylenchidae	Arctoseius	**-1.360**	**-0.152**	**5.524**	**3.485**
Tylenchidae	Cheiroseius	**-1.360**	**0.356**	**5.524**	**3.184**
Tylenchidae	Aporcelaimellus	**-1.360**	**0.548**	**5.524**	**5.251**
Tylenchidae	Dorylaimoidea	**-1.360**	**-0.604**	**5.524**	**4.825**
Tylenchidae	Epidorylaimus	**-1.360**	**0.199**	**5.524**	**4.348**
Tylenchidae	Qudsianematidae	**-1.360**	**-0.207**	**5.524**	**4.825**
Tylenchidae	Thornematidae	**-1.360**	**-0.470**	**5.524**	**4.348**
Tylenchidae	Eupodes	**-1.360**	**0.005**	**5.524**	**3.786**
Tylenchidae	Scheloribates	**-1.360**	**0.202**	**5.524**	**3.184**
Tylenchidae	Scutacarus	**-1.360**	**-0.608**	**5.524**	**3.962**
Tylenchidae	Tarsonemus	**-1.360**	**-0.701**	**5.524**	**3.485**
Medioppia	Arctoseius	**-0.235**	**-0.152**	**3.485**	**3.485**
Medioppia	Cheiroseius	**-0.235**	**0.356**	**3.485**	**3.184**
Medioppia	Aporcelaimellus	**-0.235**	**0.548**	**3.485**	**5.251**
Medioppia	Dorylaimoidea	**-0.235**	**-0.604**	**3.485**	**4.825**
Medioppia	Epidorylaimus	**-0.235**	**0.199**	**3.485**	**4.348**
Medioppia	Qudsianematidae	**-0.235**	**-0.207**	**3.485**	**4.825**
Medioppia	Thornematidae	**-0.235**	**-0.470**	**3.485**	**4.348**
Medioppia	Eupodes	**-0.235**	**0.005**	**3.485**	**3.786**
Medioppia	Scheloribates	**-0.235**	**0.202**	**3.485**	**3.184**
Medioppia	Scutacarus	**-0.235**	**-0.608**	**3.485**	**3.962**
Medioppia	Tarsonemus	**-0.235**	**-0.701**	**3.485**	**3.485**
Microtydeus	Arctoseius	**-0.863**	**-0.152**	**4.184**	**3.485**
Microtydeus	Cheiroseius	**-0.863**	**0.356**	**4.184**	**3.184**
Microtydeus	Aporcelaimellus	**-0.863**	**0.548**	**4.184**	**5.251**
Microtydeus	Dorylaimoidea	**-0.863**	**-0.604**	**4.184**	**4.825**
Microtydeus	Epidorylaimus	**-0.863**	**0.199**	**4.184**	**4.348**
Microtydeus	Qudsianematidae	**-0.863**	**-0.207**	**4.184**	**4.825**
Microtydeus	Thornematidae	**-0.863**	**-0.470**	**4.184**	**4.348**
Microtydeus	Eupodes	**-0.863**	**0.005**	**4.184**	**3.786**
Microtydeus	Scheloribates	**-0.863**	**0.202**	**4.184**	**3.184**
Microtydeus	Scutacarus	**-0.863**	**-0.608**	**4.184**	**3.962**
Microtydeus	Tarsonemus	**-0.863**	**-0.701**	**4.184**	**3.485**
Minunthozetes	Arctoseius	**-0.249**	**-0.152**	**3.485**	**3.485**
Minunthozetes	Cheiroseius	**-0.249**	**0.356**	**3.485**	**3.184**
Minunthozetes	Aporcelaimellus	**-0.249**	**0.548**	**3.485**	**5.251**
Minunthozetes	Dorylaimoidea	**-0.249**	**-0.604**	**3.485**	**4.825**
Minunthozetes	Epidorylaimus	**-0.249**	**0.199**	**3.485**	**4.348**
Minunthozetes	Qudsianematidae	**-0.249**	**-0.207**	**3.485**	**4.825**
Minunthozetes	Thornematidae	**-0.249**	**-0.470**	**3.485**	**4.348**
Minunthozetes	Eupodes	**-0.249**	**0.005**	**3.485**	**3.786**

Minunthozetes	Scheloribates	**-0.249**	**0.202**	**3.485**	**3.184**
Minunthozetes	Scutacarus	**-0.249**	**-0.608**	**3.485**	**3.962**
Minunthozetes	Tarsonemus	**-0.249**	**-0.701**	**3.485**	**3.485**
Pygmephorus	Arctoseius	**-0.376**	**-0.152**	**3.184**	**3.485**
Pygmephorus	Cheiroseius	**-0.376**	**0.356**	**3.184**	**3.184**
Pygmephorus	Aporcelaimellus	**-0.376**	**0.548**	**3.184**	**5.251**
Pygmephorus	Dorylaimoidea	**-0.376**	**-0.604**	**3.184**	**4.825**
Pygmephorus	Epidorylaimus	**-0.376**	**0.199**	**3.184**	**4.348**
Pygmephorus	Qudsianematidae	**-0.376**	**-0.207**	**3.184**	**4.825**
Pygmephorus	Thornematidae	**-0.376**	**-0.470**	**3.184**	**4.348**
Pygmephorus	Eupodes	**-0.376**	**0.005**	**3.184**	**3.786**
Pygmephorus	Scheloribates	**-0.376**	**0.202**	**3.184**	**3.184**
Pygmephorus	Scutacarus	**-0.376**	**-0.608**	**3.184**	**3.962**
Pygmephorus	Tarsonemus	**-0.376**	**-0.701**	**3.184**	**3.485**
Tectocepheus	Arctoseius	**-0.220**	**-0.152**	**3.786**	**3.485**
Tectocepheus	Cheiroseius	**-0.220**	**0.356**	**3.786**	**3.184**
Tectocepheus	Aporcelaimellus	**-0.220**	**0.548**	**3.786**	**5.251**
Tectocepheus	Dorylaimoidea	**-0.220**	**-0.604**	**3.786**	**4.825**
Tectocepheus	Epidorylaimus	**-0.220**	**0.199**	**3.786**	**4.348**
Tectocepheus	Qudsianematidae	**-0.220**	**-0.207**	**3.786**	**4.825**
Tectocepheus	Thornematidae	**-0.220**	**-0.470**	**3.786**	**4.348**
Tectocepheus	Eupodes	**-0.220**	**0.005**	**3.786**	**3.786**
Tectocepheus	Scheloribates	**-0.220**	**0.202**	**3.786**	**3.184**
Tectocepheus	Scutacarus	**-0.220**	**-0.608**	**3.786**	**3.962**
Tectocepheus	Tarsonemus	**-0.220**	**-0.701**	**3.786**	**3.485**
Tyrophagus	Arctoseius	**0.005**	**-0.152**	**3.883**	**3.485**
Tyrophagus	Cheiroseius	**0.005**	**0.356**	**3.883**	**3.184**
Tyrophagus	Aporcelaimellus	**0.005**	**0.548**	**3.883**	**5.251**
Tyrophagus	Dorylaimoidea	**0.005**	**-0.604**	**3.883**	**4.825**
Tyrophagus	Epidorylaimus	**0.005**	**0.199**	**3.883**	**4.348**
Tyrophagus	Qudsianematidae	**0.005**	**-0.207**	**3.883**	**4.825**
Tyrophagus	Thornematidae	**0.005**	**-0.470**	**3.883**	**4.348**
Tyrophagus	Eupodes	**0.005**	**0.005**	**3.883**	**3.786**
Tyrophagus	Scheloribates	**0.005**	**0.202**	**3.883**	**3.184**
Tyrophagus	Scutacarus	**0.005**	**-0.608**	**3.883**	**3.962**
Tyrophagus	Tarsonemus	**0.005**	**-0.701**	**3.883**	**3.485**
Brachystomella	Arctoseius	**0.977**	**-0.152**	**3.184**	**3.485**
Brachystomella	Cheiroseius	**0.977**	**0.356**	**3.184**	**3.184**
Brachystomella	Aporcelaimellus	**0.977**	**0.548**	**3.184**	**5.251**
Brachystomella	Dorylaimoidea	**0.977**	**-0.604**	**3.184**	**4.825**
Brachystomella	Epidorylaimus	**0.977**	**0.199**	**3.184**	**4.348**
Brachystomella	Qudsianematidae	**0.977**	**-0.207**	**3.184**	**4.825**
Brachystomella	Thornematidae	**0.977**	**-0.470**	**3.184**	**4.348**
Brachystomella	Eupodes	**0.977**	**0.005**	**3.184**	**3.786**
Brachystomella	Scheloribates	**0.977**	**0.202**	**3.184**	**3.184**
Brachystomella	Scutacarus	**0.977**	**-0.608**	**3.184**	**3.962**
Brachystomella	Tarsonemus	**0.977**	**-0.701**	**3.184**	**3.485**
Isotoma	Arctoseius	**1.898**	**-0.152**	**4.029**	**3.485**
Isotoma	Cheiroseius	**1.898**	**0.356**	**4.029**	**3.184**
Isotoma	Aporcelaimellus	**1.898**	**0.548**	**4.029**	**5.251**
Isotoma	Dorylaimoidea	**1.898**	**-0.604**	**4.029**	**4.825**

Isotoma	Epidorylaimus	1.898	0.199	4.029	4.348
Isotoma	Qudsianematidae	1.898	-0.207	4.029	4.825
Isotoma	Thornematidae	1.898	-0.470	4.029	4.348
Isotoma	Eupodes	1.898	0.005	4.029	3.786
Isotoma	Scheloribates	1.898	0.202	4.029	3.184
Isotoma	Scutacarus	1.898	-0.608	4.029	3.962
Isotoma	Tarsonemus	1.898	-0.701	4.029	3.485
Lepidocyrtus	Arctoseius	1.231	-0.152	3.184	3.485
Lepidocyrtus	Cheiroseius	1.231	0.356	3.184	3.184
Lepidocyrtus	Aporcelaimellus	1.231	0.548	3.184	5.251
Lepidocyrtus	Dorylaimoidea	1.231	-0.604	3.184	4.825
Lepidocyrtus	Epidorylaimus	1.231	0.199	3.184	4.348
Lepidocyrtus	Qudsianematidae	1.231	-0.207	3.184	4.825
Lepidocyrtus	Thornematidae	1.231	-0.470	3.184	4.348
Lepidocyrtus	Eupodes	1.231	0.005	3.184	3.786
Lepidocyrtus	Scheloribates	1.231	0.202	3.184	3.184
Lepidocyrtus	Scutacarus	1.231	-0.608	3.184	3.962
Lepidocyrtus	Tarsonemus	1.231	-0.701	3.184	3.485
Proisotoma	Arctoseius	0.770	-0.152	4.138	3.485
Proisotoma	Cheiroseius	0.770	0.356	4.138	3.184
Proisotoma	Aporcelaimellus	0.770	0.548	4.138	5.251
Proisotoma	Dorylaimoidea	0.770	-0.604	4.138	4.825
Proisotoma	Epidorylaimus	0.770	0.199	4.138	4.348
Proisotoma	Qudsianematidae	0.770	-0.207	4.138	4.825
Proisotoma	Thornematidae	0.770	-0.470	4.138	4.348
Proisotoma	Eupodes	0.770	0.005	4.138	3.786
Proisotoma	Scheloribates	0.770	0.202	4.138	3.184
Proisotoma	Scutacarus	0.770	-0.608	4.138	3.962
Proisotoma	Tarsonemus	0.770	-0.701	4.138	3.485
Achaeta	Arctoseius	1.046	-0.152	2.947	3.485
Achaeta	Cheiroseius	1.046	0.356	2.947	3.184
Achaeta	Aporcelaimellus	1.046	0.548	2.947	5.251
Achaeta	Dorylaimoidea	1.046	-0.604	2.947	4.825
Achaeta	Epidorylaimus	1.046	0.199	2.947	4.348
Achaeta	Qudsianematidae	1.046	-0.207	2.947	4.825
Achaeta	Thornematidae	1.046	-0.470	2.947	4.348
Achaeta	Eupodes	1.046	0.005	2.947	3.786
Achaeta	Scheloribates	1.046	0.202	2.947	3.184
Achaeta	Scutacarus	1.046	-0.608	2.947	3.962
Achaeta	Tarsonemus	1.046	-0.701	2.947	3.485
Cognettia	Arctoseius	1.666	-0.152	3.820	3.485
Cognettia	Cheiroseius	1.666	0.356	3.820	3.184
Cognettia	Aporcelaimellus	1.666	0.548	3.820	5.251
Cognettia	Dorylaimoidea	1.666	-0.604	3.820	4.825
Cognettia	Epidorylaimus	1.666	0.199	3.820	4.348
Cognettia	Qudsianematidae	1.666	-0.207	3.820	4.825
Cognettia	Thornematidae	1.666	-0.470	3.820	4.348
Cognettia	Eupodes	1.666	0.005	3.820	3.786
Cognettia	Scheloribates	1.666	0.202	3.820	3.184
Cognettia	Scutacarus	1.666	-0.608	3.820	3.962
Cognettia	Tarsonemus	1.666	-0.701	3.820	3.485

Fridericia	Arctoseius	**2.627**	**-0.152**	**3.556**	**3.485**
Fridericia	Cheiroseius	**2.627**	**0.356**	**3.556**	**3.184**
Fridericia	Aporcelaimellus	**2.627**	**0.548**	**3.556**	**5.251**
Fridericia	Dorylaimoidea	**2.627**	**-0.604**	**3.556**	**4.825**
Fridericia	Epidorylaimus	**2.627**	**0.199**	**3.556**	**4.348**
Fridericia	Qudsianematidae	**2.627**	**-0.207**	**3.556**	**4.825**
Fridericia	Thornematidae	**2.627**	**-0.470**	**3.556**	**4.348**
Fridericia	Eupodes	**2.627**	**0.005**	**3.556**	**3.786**
Fridericia	Scheloribates	**2.627**	**0.202**	**3.556**	**3.184**
Fridericia	Scutacarus	**2.627**	**-0.608**	**3.556**	**3.962**
Fridericia	Tarsonemus	**2.627**	**-0.701**	**3.556**	**3.485**
Acrobeles	Seinura	**-0.721**	**-0.814**	**5.552**	**4.348**
Acrobeles	Tripyla	**-0.721**	**-0.420**	**5.552**	**4.649**
Acrobeles	Arctoseius	**-0.721**	**-0.152**	**5.552**	**3.485**
Acrobeles	Cheiroseius	**-0.721**	**0.356**	**5.552**	**3.184**
Acrobeles	Aporcelaimellus	**-0.721**	**0.548**	**5.552**	**5.251**
Acrobeles	Dorylaimoidea	**-0.721**	**-0.604**	**5.552**	**4.825**
Acrobeles	Epidorylaimus	**-0.721**	**0.199**	**5.552**	**4.348**
Acrobeles	Qudsianematidae	**-0.721**	**-0.207**	**5.552**	**4.825**
Acrobeles	Thornematidae	**-0.721**	**-0.470**	**5.552**	**4.348**
Acrobeles	Eupodes	**-0.721**	**0.005**	**5.552**	**3.786**
Acrobeles	Scheloribates	**-0.721**	**0.202**	**5.552**	**3.184**
Acrobeles	Scutacarus	**-0.721**	**-0.608**	**5.552**	**3.962**
Acrobeles	Tarsonemus	**-0.721**	**-0.701**	**5.552**	**3.485**
Acrobeloides	Seinura	**-1.171**	**-0.814**	**4.950**	**4.348**
Acrobeloides	Tripyla	**-1.171**	**-0.420**	**4.950**	**4.649**
Acrobeloides	Arctoseius	**-1.171**	**-0.152**	**4.950**	**3.485**
Acrobeloides	Cheiroseius	**-1.171**	**0.356**	**4.950**	**3.184**
Acrobeloides	Aporcelaimellus	**-1.171**	**0.548**	**4.950**	**5.251**
Acrobeloides	Dorylaimoidea	**-1.171**	**-0.604**	**4.950**	**4.825**
Acrobeloides	Epidorylaimus	**-1.171**	**0.199**	**4.950**	**4.348**
Acrobeloides	Qudsianematidae	**-1.171**	**-0.207**	**4.950**	**4.825**
Acrobeloides	Thornematidae	**-1.171**	**-0.470**	**4.950**	**4.348**
Acrobeloides	Eupodes	**-1.171**	**0.005**	**4.950**	**3.786**
Acrobeloides	Scheloribates	**-1.171**	**0.202**	**4.950**	**3.184**
Acrobeloides	Scutacarus	**-1.171**	**-0.608**	**4.950**	**3.962**
Acrobeloides	Tarsonemus	**-1.171**	**-0.701**	**4.950**	**3.485**
Cephalobidae	Seinura	**-1.055**	**-0.814**	**4.348**	**4.348**
Cephalobidae	Tripyla	**-1.055**	**-0.420**	**4.348**	**4.649**
Cephalobidae	Arctoseius	**-1.055**	**-0.152**	**4.348**	**3.485**
Cephalobidae	Cheiroseius	**-1.055**	**0.356**	**4.348**	**3.184**
Cephalobidae	Aporcelaimellus	**-1.055**	**0.548**	**4.348**	**5.251**
Cephalobidae	Dorylaimoidea	**-1.055**	**-0.604**	**4.348**	**4.825**
Cephalobidae	Epidorylaimus	**-1.055**	**0.199**	**4.348**	**4.348**
Cephalobidae	Qudsianematidae	**-1.055**	**-0.207**	**4.348**	**4.825**
Cephalobidae	Thornematidae	**-1.055**	**-0.470**	**4.348**	**4.348**
Cephalobidae	Eupodes	**-1.055**	**0.005**	**4.348**	**3.786**
Cephalobidae	Scheloribates	**-1.055**	**0.202**	**4.348**	**3.184**
Cephalobidae	Scutacarus	**-1.055**	**-0.608**	**4.348**	**3.962**
Cephalobidae	Tarsonemus	**-1.055**	**-0.701**	**4.348**	**3.485**
Cervidellus	Seinura	**-1.244**	**-0.814**	**4.649**	**4.348**

Cervidellus	Tripyla	**-1.244**	**-0.420**	**4.649**	**4.649**
Cervidellus	Arctoseius	**-1.244**	**-0.152**	**4.649**	**3.485**
Cervidellus	Cheiroseius	**-1.244**	**0.356**	**4.649**	**3.184**
Cervidellus	Aporcelaimellus	**-1.244**	**0.548**	**4.649**	**5.251**
Cervidellus	Dorylaimoidea	**-1.244**	**-0.604**	**4.649**	**4.825**
Cervidellus	Epidorylaimus	**-1.244**	**0.199**	**4.649**	**4.348**
Cervidellus	Qudsianematidae	**-1.244**	**-0.207**	**4.649**	**4.825**
Cervidellus	Thornematidae	**-1.244**	**-0.470**	**4.649**	**4.348**
Cervidellus	Eupodes	**-1.244**	**0.005**	**4.649**	**3.786**
Cervidellus	Scheloribates	**-1.244**	**0.202**	**4.649**	**3.184**
Cervidellus	Scutacarus	**-1.244**	**-0.608**	**4.649**	**3.962**
Cervidellus	Tarsonemus	**-1.244**	**-0.701**	**4.649**	**3.485**
Eucephalobus	Seinura	**-0.855**	**-0.814**	**5.746**	**4.348**
Eucephalobus	Tripyla	**-0.855**	**-0.420**	**5.746**	**4.649**
Eucephalobus	Arctoseius	**-0.855**	**-0.152**	**5.746**	**3.485**
Eucephalobus	Cheiroseius	**-0.855**	**0.356**	**5.746**	**3.184**
Eucephalobus	Aporcelaimellus	**-0.855**	**0.548**	**5.746**	**5.251**
Eucephalobus	Dorylaimoidea	**-0.855**	**-0.604**	**5.746**	**4.825**
Eucephalobus	Epidorylaimus	**-0.855**	**0.199**	**5.746**	**4.348**
Eucephalobus	Qudsianematidae	**-0.855**	**-0.207**	**5.746**	**4.825**
Eucephalobus	Thornematidae	**-0.855**	**-0.470**	**5.746**	**4.348**
Eucephalobus	Eupodes	**-0.855**	**0.005**	**5.746**	**3.786**
Eucephalobus	Scheloribates	**-0.855**	**0.202**	**5.746**	**3.184**
Eucephalobus	Scutacarus	**-0.855**	**-0.608**	**5.746**	**3.962**
Eucephalobus	Tarsonemus	**-0.855**	**-0.701**	**5.746**	**3.485**
Metateratocephalus	Seinura	**-1.506**	**-0.814**	**5.302**	**4.348**
Metateratocephalus	Tripyla	**-1.506**	**-0.420**	**5.302**	**4.649**
Metateratocephalus	Arctoseius	**-1.506**	**-0.152**	**5.302**	**3.485**
Metateratocephalus	Cheiroseius	**-1.506**	**0.356**	**5.302**	**3.184**
Metateratocephalus	Aporcelaimellus	**-1.506**	**0.548**	**5.302**	**5.251**
Metateratocephalus	Dorylaimoidea	**-1.506**	**-0.604**	**5.302**	**4.825**
Metateratocephalus	Epidorylaimus	**-1.506**	**0.199**	**5.302**	**4.348**
Metateratocephalus	Qudsianematidae	**-1.506**	**-0.207**	**5.302**	**4.825**
Metateratocephalus	Thornematidae	**-1.506**	**-0.470**	**5.302**	**4.348**
Metateratocephalus	Eupodes	**-1.506**	**0.005**	**5.302**	**3.786**
Metateratocephalus	Scheloribates	**-1.506**	**0.202**	**5.302**	**3.184**
Metateratocephalus	Scutacarus	**-1.506**	**-0.608**	**5.302**	**3.962**
Metateratocephalus	Tarsonemus	**-1.506**	**-0.701**	**5.302**	**3.485**
Panagrolaimus	Seinura	**-0.945**	**-0.814**	**4.950**	**4.348**
Panagrolaimus	Tripyla	**-0.945**	**-0.420**	**4.950**	**4.649**
Panagrolaimus	Arctoseius	**-0.945**	**-0.152**	**4.950**	**3.485**
Panagrolaimus	Cheiroseius	**-0.945**	**0.356**	**4.950**	**3.184**
Panagrolaimus	Aporcelaimellus	**-0.945**	**0.548**	**4.950**	**5.251**
Panagrolaimus	Dorylaimoidea	**-0.945**	**-0.604**	**4.950**	**4.825**
Panagrolaimus	Epidorylaimus	**-0.945**	**0.199**	**4.950**	**4.348**
Panagrolaimus	Qudsianematidae	**-0.945**	**-0.207**	**4.950**	**4.825**
Panagrolaimus	Thornematidae	**-0.945**	**-0.470**	**4.950**	**4.348**
Panagrolaimus	Eupodes	**-0.945**	**0.005**	**4.950**	**3.786**
Panagrolaimus	Scheloribates	**-0.945**	**0.202**	**4.950**	**3.184**
Panagrolaimus	Scutacarus	**-0.945**	**-0.608**	**4.950**	**3.962**
Panagrolaimus	Tarsonemus	**-0.945**	**-0.701**	**4.950**	**3.485**

Plectus	Seinura	**-0.583**	**-0.814**	**4.825**	**4.348**
Plectus	Tripyla	**-0.583**	**-0.420**	**4.825**	**4.649**
Plectus	Arctoseius	**-0.583**	**-0.152**	**4.825**	**3.485**
Plectus	Cheiroseius	**-0.583**	**0.356**	**4.825**	**3.184**
Plectus	Aporcelaimellus	**-0.583**	**0.548**	**4.825**	**5.251**
Plectus	Dorylaimoidea	**-0.583**	**-0.604**	**4.825**	**4.825**
Plectus	Epidorylaimus	**-0.583**	**0.199**	**4.825**	**4.348**
Plectus	Qudsianematidae	**-0.583**	**-0.207**	**4.825**	**4.825**
Plectus	Thornematidae	**-0.583**	**-0.470**	**4.825**	**4.348**
Plectus	Eupodes	**-0.583**	**0.005**	**4.825**	**3.786**
Plectus	Scheloribates	**-0.583**	**0.202**	**4.825**	**3.184**
Plectus	Scutacarus	**-0.583**	**-0.608**	**4.825**	**3.962**
Plectus	Tarsonemus	**-0.583**	**-0.701**	**4.825**	**3.485**
Prismatolaimus	Seinura	**-1.280**	**-0.814**	**4.649**	**4.348**
Prismatolaimus	Tripyla	**-1.280**	**-0.420**	**4.649**	**4.649**
Prismatolaimus	Arctoseius	**-1.280**	**-0.152**	**4.649**	**3.485**
Prismatolaimus	Cheiroseius	**-1.280**	**0.356**	**4.649**	**3.184**
Prismatolaimus	Aporcelaimellus	**-1.280**	**0.548**	**4.649**	**5.251**
Prismatolaimus	Dorylaimoidea	**-1.280**	**-0.604**	**4.649**	**4.825**
Prismatolaimus	Epidorylaimus	**-1.280**	**0.199**	**4.649**	**4.348**
Prismatolaimus	Qudsianematidae	**-1.280**	**-0.207**	**4.649**	**4.825**
Prismatolaimus	Thornematidae	**-1.280**	**-0.470**	**4.649**	**4.348**
Prismatolaimus	Eupodes	**-1.280**	**0.005**	**4.649**	**3.786**
Prismatolaimus	Scheloribates	**-1.280**	**0.202**	**4.649**	**3.184**
Prismatolaimus	Scutacarus	**-1.280**	**-0.608**	**4.649**	**3.962**
Prismatolaimus	Tarsonemus	**-1.280**	**-0.701**	**4.649**	**3.485**
Rhabditidae	Seinura	**-0.692**	**-0.814**	**5.047**	**4.348**
Rhabditidae	Tripyla	**-0.692**	**-0.420**	**5.047**	**4.649**
Rhabditidae	Arctoseius	**-0.692**	**-0.152**	**5.047**	**3.485**
Rhabditidae	Cheiroseius	**-0.692**	**0.356**	**5.047**	**3.184**
Rhabditidae	Aporcelaimellus	**-0.692**	**0.548**	**5.047**	**5.251**
Rhabditidae	Dorylaimoidea	**-0.692**	**-0.604**	**5.047**	**4.825**
Rhabditidae	Epidorylaimus	**-0.692**	**0.199**	**5.047**	**4.348**
Rhabditidae	Qudsianematidae	**-0.692**	**-0.207**	**5.047**	**4.825**
Rhabditidae	Thornematidae	**-0.692**	**-0.470**	**5.047**	**4.348**
Rhabditidae	Eupodes	**-0.692**	**0.005**	**5.047**	**3.786**
Rhabditidae	Scheloribates	**-0.692**	**0.202**	**5.047**	**3.184**
Rhabditidae	Scutacarus	**-0.692**	**-0.608**	**5.047**	**3.962**
Rhabditidae	Tarsonemus	**-0.692**	**-0.701**	**5.047**	**3.485**
Teratocephalus	Seinura	**-1.630**	**-0.814**	**4.649**	**4.348**
Teratocephalus	Tripyla	**-1.630**	**-0.420**	**4.649**	**4.649**
Teratocephalus	Arctoseius	**-1.630**	**-0.152**	**4.649**	**3.485**
Teratocephalus	Cheiroseius	**-1.630**	**0.356**	**4.649**	**3.184**
Teratocephalus	Aporcelaimellus	**-1.630**	**0.548**	**4.649**	**5.251**
Teratocephalus	Dorylaimoidea	**-1.630**	**-0.604**	**4.649**	**4.825**
Teratocephalus	Epidorylaimus	**-1.630**	**0.199**	**4.649**	**4.348**
Teratocephalus	Qudsianematidae	**-1.630**	**-0.207**	**4.649**	**4.825**
Teratocephalus	Thornematidae	**-1.630**	**-0.470**	**4.649**	**4.348**
Teratocephalus	Eupodes	**-1.630**	**0.005**	**4.649**	**3.786**
Teratocephalus	Scheloribates	**-1.630**	**0.202**	**4.649**	**3.184**
Teratocephalus	Scutacarus	**-1.630**	**-0.608**	**4.649**	**3.962**

Teratocephalus	Tarsonemus	**-1.630**	**-0.701**	**4.649**	**3.485**
Enchytraeus	Arctoseius	**1.546**	**-0.152**	**2.674**	**3.485**
Enchytraeus	Cheiroseius	**1.546**	**0.356**	**2.674**	**3.184**
Enchytraeus	Aporcelaimellus	**1.546**	**0.548**	**2.674**	**5.251**
Enchytraeus	Dorylaimoidea	**1.546**	**-0.604**	**2.674**	**4.825**
Enchytraeus	Epidorylaimus	**1.546**	**0.199**	**2.674**	**4.348**
Enchytraeus	Qudsianematidae	**1.546**	**-0.207**	**2.674**	**4.825**
Enchytraeus	Thornematidae	**1.546**	**-0.470**	**2.674**	**4.348**
Enchytraeus	Eupodes	**1.546**	**0.005**	**2.674**	**3.786**
Enchytraeus	Scheloribates	**1.546**	**0.202**	**2.674**	**3.184**
Enchytraeus	Scutacarus	**1.546**	**-0.608**	**2.674**	**3.962**
Enchytraeus	Tarsonemus	**1.546**	**-0.701**	**2.674**	**3.485**
Eubacteria	Acrobeles	**-6.643**	**-0.721**	**12.398**	**5.552**
Eubacteria	Acrobeloides	**-6.643**	**-1.171**	**12.398**	**4.950**
Eubacteria	Cephalobidae	**-6.643**	**-1.055**	**12.398**	**4.348**
Eubacteria	Cervidellus	**-6.643**	**-1.244**	**12.398**	**4.649**
Eubacteria	Eucephalobus	**-6.643**	**-0.855**	**12.398**	**5.746**
Eubacteria	Metateratocephalus	**-6.643**	**-1.506**	**12.398**	**5.302**
Eubacteria	Panagrolaimus	**-6.643**	**-0.945**	**12.398**	**4.950**
Eubacteria	Plectus	**-6.643**	**-0.583**	**12.398**	**4.825**
Eubacteria	Prismatolaimus	**-6.643**	**-1.280**	**12.398**	**4.649**
Eubacteria	Rhabditidae	**-6.643**	**-0.692**	**12.398**	**5.047**
Eubacteria	Teratocephalus	**-6.643**	**-1.630**	**12.398**	**4.649**
Eubacteria	Enchytraeus	**-6.643**	**1.546**	**12.398**	**2.674**
Eubacteria	Henlea	**-6.643**	**1.686**	**12.398**	**2.975**
Eubacteria	Marionina	**-6.643**	**1.156**	**12.398**	**3.202**
Henlea	Arctoseius	**1.686**	**-0.152**	**2.975**	**3.485**
Henlea	Cheiroseius	**1.686**	**0.356**	**2.975**	**3.184**
Henlea	Aporcelaimellus	**1.686**	**0.548**	**2.975**	**5.251**
Henlea	Dorylaimoidea	**1.686**	**-0.604**	**2.975**	**4.825**
Henlea	Epidorylaimus	**1.686**	**0.199**	**2.975**	**4.348**
Henlea	Qudsianematidae	**1.686**	**-0.207**	**2.975**	**4.825**
Henlea	Thornematidae	**1.686**	**-0.470**	**2.975**	**4.348**
Henlea	Eupodes	**1.686**	**0.005**	**2.975**	**3.786**
Henlea	Scheloribates	**1.686**	**0.202**	**2.975**	**3.184**
Henlea	Scutacarus	**1.686**	**-0.608**	**2.975**	**3.962**
Henlea	Tarsonemus	**1.686**	**-0.701**	**2.975**	**3.485**
Marionina	Arctoseius	**1.156**	**-0.152**	**3.202**	**3.485**
Marionina	Cheiroseius	**1.156**	**0.356**	**3.202**	**3.184**
Marionina	Aporcelaimellus	**1.156**	**0.548**	**3.202**	**5.251**
Marionina	Dorylaimoidea	**1.156**	**-0.604**	**3.202**	**4.825**
Marionina	Epidorylaimus	**1.156**	**0.199**	**3.202**	**4.348**
Marionina	Qudsianematidae	**1.156**	**-0.207**	**3.202**	**4.825**
Marionina	Thornematidae	**1.156**	**-0.470**	**3.202**	**4.348**
Marionina	Eupodes	**1.156**	**0.005**	**3.202**	**3.786**
Marionina	Scheloribates	**1.156**	**0.202**	**3.202**	**3.184**
Marionina	Scutacarus	**1.156**	**-0.608**	**3.202**	**3.962**
Marionina	Tarsonemus	**1.156**	**-0.701**	**3.202**	**3.485**
Hyphae and hair roots	Aglenchus	**5.894**	**-1.053**	**0.000**	**4.649**
Hyphae and hair roots	Coslenchus	**5.894**	**-0.821**	**0.000**	**4.348**
Hyphae and hair roots	Dolichodoridae	**5.894**	**-0.885**	**0.000**	**5.047**

Hyphae and hair roots	Filenchus	**5.894**	**-1.033**	**0.000**	**4.348**
Hyphae and hair roots	Helicotylenchus	**5.894**	**-0.792**	**0.000**	**4.649**
Hyphae and hair roots	Malenchus	**5.894**	**-1.330**	**0.000**	**4.348**
Hyphae and hair roots	Meloidogyne	**5.894**	**-1.287**	**0.000**	**4.348**
Hyphae and hair roots	Paratylenchus	**5.894**	**-1.244**	**0.000**	**4.348**
Hyphae and hair roots	Pratylenchus	**5.894**	**-1.226**	**0.000**	**4.348**
Hyphae and hair roots	Trichodorus	**5.894**	**-0.744**	**0.000**	**5.302**
Hyphae and hair roots	Tylenchorhynchus	**5.894**	**-0.664**	**0.000**	**5.462**
Hyphae and hair roots	Pachygnatidae	**5.894**	**-0.113**	**0.000**	**3.661**
Hyphae and hair roots	Platynothrus	**5.894**	**0.710**	**0.000**	**3.485**
Hyphae and hair roots	Tydeidae	**5.894**	**-0.608**	**0.000**	**3.184**
Hyphae and hair roots	Sminthuridae	**5.894**	**-0.608**	**0.000**	**3.786**
Hyphae and hair roots	Sminthurinus	**5.894**	**0.618**	**0.000**	**3.485**
Hyphae and hair roots	Aphelenchoides	**5.894**	**-1.496**	**0.000**	**5.126**
Hyphae and hair roots	Tylenchidae	**5.894**	**-1.360**	**0.000**	**5.524**
Hyphae and hair roots	Medioppia	**5.894**	**-0.235**	**0.000**	**3.485**
Hyphae and hair roots	Microtydeus	**5.894**	**-0.863**	**0.000**	**4.184**
Hyphae and hair roots	Minunthozetes	**5.894**	**-0.249**	**0.000**	**3.485**
Hyphae and hair roots	Pygmephorus	**5.894**	**-0.376**	**0.000**	**3.184**
Hyphae and hair roots	Tectocepheus	**5.894**	**-0.220**	**0.000**	**3.786**
Hyphae and hair roots	Tyrophagus	**5.894**	**0.005**	**0.000**	**3.883**
Hyphae and hair roots	Brachystomella	**5.894**	**0.977**	**0.000**	**3.184**
Hyphae and hair roots	Isotoma	**5.894**	**1.898**	**0.000**	**4.029**
Hyphae and hair roots	Lepidocyrtus	**5.894**	**1.231**	**0.000**	**3.184**
Hyphae and hair roots	Proisotoma	**5.894**	**0.770**	**0.000**	**4.138**
Hyphae and hair roots	Achaeta	**5.894**	**1.046**	**0.000**	**2.947**
Hyphae and hair roots	Cognettia	**5.894**	**1.666**	**0.000**	**3.820**
Hyphae and hair roots	Fridericia	**5.894**	**2.627**	**0.000**	**3.556**
Hyphae and hair roots	Aporcelaimellus	**5.894**	**0.548**	**0.000**	**5.251**
Hyphae and hair roots	Dorylaimoidea	**5.894**	**-0.604**	**0.000**	**4.825**
Hyphae and hair roots	Epidorylaimus	**5.894**	**0.199**	**0.000**	**4.348**
Hyphae and hair roots	Qudsianematidae	**5.894**	**-0.207**	**0.000**	**4.825**
Hyphae and hair roots	Thornematidae	**5.894**	**-0.470**	**0.000**	**4.348**
Hyphae and hair roots	Eupodes	**5.894**	**0.005**	**0.000**	**3.786**
Hyphae and hair roots	Scheloribates	**5.894**	**0.202**	**0.000**	**3.184**
Hyphae and hair roots	Scutacarus	**5.894**	**-0.608**	**0.000**	**3.962**
Hyphae and hair roots	Tarsonemus	**5.894**	**-0.701**	**0.000**	**3.485**
Seinura	Arctoseius	**-0.814**	**-0.152**	**4.348**	**3.485**
Seinura	Cheiroseius	**-0.814**	**0.356**	**4.348**	**3.184**
Seinura	Aporcelaimellus	**-0.814**	**0.548**	**4.348**	**5.251**
Seinura	Dorylaimoidea	**-0.814**	**-0.604**	**4.348**	**4.825**
Seinura	Epidorylaimus	**-0.814**	**0.199**	**4.348**	**4.348**
Seinura	Qudsianematidae	**-0.814**	**-0.207**	**4.348**	**4.825**
Seinura	Thornematidae	**-0.814**	**-0.470**	**4.348**	**4.348**
Seinura	Eupodes	**-0.814**	**0.005**	**4.348**	**3.786**
Seinura	Scheloribates	**-0.814**	**0.202**	**4.348**	**3.184**
Seinura	Scutacarus	**-0.814**	**-0.608**	**4.348**	**3.962**
Seinura	Tarsonemus	**-0.814**	**-0.701**	**4.348**	**3.485**
Tripyla	Arctoseius	**-0.420**	**-0.152**	**4.649**	**3.485**
Tripyla	Cheiroseius	**-0.420**	**0.356**	**4.649**	**3.184**
Tripyla	Aporcelaimellus	**-0.420**	**0.548**	**4.649**	**5.251**

Tripyla	Dorylaimoidea	**-0.420**	**-0.604**	**4.649**	**4.825**
Tripyla	Epidorylaimus	**-0.420**	**0.199**	**4.649**	**4.348**
Tripyla	Qudsianematidae	**-0.420**	**-0.207**	**4.649**	**4.825**
Tripyla	Thornematidae	**-0.420**	**-0.470**	**4.649**	**4.348**
Tripyla	Eupodes	**-0.420**	**0.005**	**4.649**	**3.786**
Tripyla	Scheloribates	**-0.420**	**0.202**	**4.649**	**3.184**
Tripyla	Scutacarus	**-0.420**	**-0.608**	**4.649**	**3.962**
Tripyla	Tarsonemus	**-0.420**	**-0.701**	**4.649**	**3.485**
Arctoseius	Aporcelaimellus	**-0.152**	**0.548**	**3.485**	**5.251**
Arctoseius	Dorylaimoidea	**-0.152**	**-0.604**	**3.485**	**4.825**
Arctoseius	Epidorylaimus	**-0.152**	**0.199**	**3.485**	**4.348**
Arctoseius	Qudsianematidae	**-0.152**	**-0.207**	**3.485**	**4.825**
Arctoseius	Thornematidae	**-0.152**	**-0.470**	**3.485**	**4.348**
Arctoseius	Eupodes	**-0.152**	**0.005**	**3.485**	**3.786**
Arctoseius	Scheloribates	**-0.152**	**0.202**	**3.485**	**3.184**
Arctoseius	Scutacarus	**-0.152**	**-0.608**	**3.485**	**3.962**
Arctoseius	Tarsonemus	**-0.152**	**-0.701**	**3.485**	**3.485**
Cheiroseius	Aporcelaimellus	**0.356**	**0.548**	**3.184**	**5.251**
Cheiroseius	Dorylaimoidea	**0.356**	**-0.604**	**3.184**	**4.825**
Cheiroseius	Epidorylaimus	**0.356**	**0.199**	**3.184**	**4.348**
Cheiroseius	Qudsianematidae	**0.356**	**-0.207**	**3.184**	**4.825**
Cheiroseius	Thornematidae	**0.356**	**-0.470**	**3.184**	**4.348**
Cheiroseius	Eupodes	**0.356**	**0.005**	**3.184**	**3.786**
Cheiroseius	Scheloribates	**0.356**	**0.202**	**3.184**	**3.184**
Cheiroseius	Scutacarus	**0.356**	**-0.608**	**3.184**	**3.962**
Cheiroseius	Tarsonemus	**0.356**	**-0.701**	**3.184**	**3.485**
Aporcelaimellus	Seinura	**0.548**	**-0.814**	**5.251**	**4.348**
Aporcelaimellus	Tripyla	**0.548**	**-0.420**	**5.251**	**4.649**
Aporcelaimellus	Arctoseius	**0.548**	**-0.152**	**5.251**	**3.485**
Aporcelaimellus	Cheiroseius	**0.548**	**0.356**	**5.251**	**3.184**
Aporcelaimellus	Aporcelaimellus	**0.548**	**0.548**	**5.251**	**5.251**
Aporcelaimellus	Dorylaimoidea	**0.548**	**-0.604**	**5.251**	**4.825**
Aporcelaimellus	Epidorylaimus	**0.548**	**0.199**	**5.251**	**4.348**
Aporcelaimellus	Qudsianematidae	**0.548**	**-0.207**	**5.251**	**4.825**
Aporcelaimellus	Thornematidae	**0.548**	**-0.470**	**5.251**	**4.348**
Aporcelaimellus	Eupodes	**0.548**	**0.005**	**5.251**	**3.786**
Aporcelaimellus	Scheloribates	**0.548**	**0.202**	**5.251**	**3.184**
Aporcelaimellus	Scutacarus	**0.548**	**-0.608**	**5.251**	**3.962**
Aporcelaimellus	Tarsonemus	**0.548**	**-0.701**	**5.251**	**3.485**
Dorylaimoidea	Seinura	**-0.604**	**-0.814**	**4.825**	**4.348**
Dorylaimoidea	Tripyla	**-0.604**	**-0.420**	**4.825**	**4.649**
Dorylaimoidea	Arctoseius	**-0.604**	**-0.152**	**4.825**	**3.485**
Dorylaimoidea	Cheiroseius	**-0.604**	**0.356**	**4.825**	**3.184**
Dorylaimoidea	Aporcelaimellus	**-0.604**	**0.548**	**4.825**	**5.251**
Dorylaimoidea	Dorylaimoidea	**-0.604**	**-0.604**	**4.825**	**4.825**
Dorylaimoidea	Epidorylaimus	**-0.604**	**0.199**	**4.825**	**4.348**
Dorylaimoidea	Qudsianematidae	**-0.604**	**-0.207**	**4.825**	**4.825**
Dorylaimoidea	Thornematidae	**-0.604**	**-0.470**	**4.825**	**4.348**
Dorylaimoidea	Eupodes	**-0.604**	**0.005**	**4.825**	**3.786**
Dorylaimoidea	Scheloribates	**-0.604**	**0.202**	**4.825**	**3.184**
Dorylaimoidea	Scutacarus	**-0.604**	**-0.608**	**4.825**	**3.962**

Dorylaimoidea	Tarsonemus	**-0.604**	**-0.701**	**4.825**	**3.485**
Epidorylaimus	Seinura	**0.199**	**-0.814**	**4.348**	**4.348**
Epidorylaimus	Tripyla	**0.199**	**-0.420**	**4.348**	**4.649**
Epidorylaimus	Arctoseius	**0.199**	**-0.152**	**4.348**	**3.485**
Epidorylaimus	Cheiroseius	**0.199**	**0.356**	**4.348**	**3.184**
Epidorylaimus	Aporcelaimellus	**0.199**	**0.548**	**4.348**	**5.251**
Epidorylaimus	Dorylaimoidea	**0.199**	**-0.604**	**4.348**	**4.825**
Epidorylaimus	Epidorylaimus	**0.199**	**0.199**	**4.348**	**4.348**
Epidorylaimus	Qudsianematidae	**0.199**	**-0.207**	**4.348**	**4.825**
Epidorylaimus	Thornematidae	**0.199**	**-0.470**	**4.348**	**4.348**
Epidorylaimus	Eupodes	**0.199**	**0.005**	**4.348**	**3.786**
Epidorylaimus	Scheloribates	**0.199**	**0.202**	**4.348**	**3.184**
Epidorylaimus	Scutacarus	**0.199**	**-0.608**	**4.348**	**3.962**
Epidorylaimus	Tarsonemus	**0.199**	**-0.701**	**4.348**	**3.485**
Qudsianematidae	Seinura	**-0.207**	**-0.814**	**4.825**	**4.348**
Qudsianematidae	Tripyla	**-0.207**	**-0.420**	**4.825**	**4.649**
Qudsianematidae	Arctoseius	**-0.207**	**-0.152**	**4.825**	**3.485**
Qudsianematidae	Cheiroseius	**-0.207**	**0.356**	**4.825**	**3.184**
Qudsianematidae	Aporcelaimellus	**-0.207**	**0.548**	**4.825**	**5.251**
Qudsianematidae	Dorylaimoidea	**-0.207**	**-0.604**	**4.825**	**4.825**
Qudsianematidae	Epidorylaimus	**-0.207**	**0.199**	**4.825**	**4.348**
Qudsianematidae	Qudsianematidae	**-0.207**	**-0.207**	**4.825**	**4.825**
Qudsianematidae	Thornematidae	**-0.207**	**-0.470**	**4.825**	**4.348**
Qudsianematidae	Eupodes	**-0.207**	**0.005**	**4.825**	**3.786**
Qudsianematidae	Scheloribates	**-0.207**	**0.202**	**4.825**	**3.184**
Qudsianematidae	Scutacarus	**-0.207**	**-0.608**	**4.825**	**3.962**
Qudsianematidae	Tarsonemus	**-0.207**	**-0.701**	**4.825**	**3.485**
Thornematidae	Seinura	**-0.470**	**-0.814**	**4.348**	**4.348**
Thornematidae	Tripyla	**-0.470**	**-0.420**	**4.348**	**4.649**
Thornematidae	Arctoseius	**-0.470**	**-0.152**	**4.348**	**3.485**
Thornematidae	Cheiroseius	**-0.470**	**0.356**	**4.348**	**3.184**
Thornematidae	Aporcelaimellus	**-0.470**	**0.548**	**4.348**	**5.251**
Thornematidae	Dorylaimoidea	**-0.470**	**-0.604**	**4.348**	**4.825**
Thornematidae	Epidorylaimus	**-0.470**	**0.199**	**4.348**	**4.348**
Thornematidae	Qudsianematidae	**-0.470**	**-0.207**	**4.348**	**4.825**
Thornematidae	Thornematidae	**-0.470**	**-0.470**	**4.348**	**4.348**
Thornematidae	Eupodes	**-0.470**	**0.005**	**4.348**	**3.786**
Thornematidae	Scheloribates	**-0.470**	**0.202**	**4.348**	**3.184**
Thornematidae	Scutacarus	**-0.470**	**-0.608**	**4.348**	**3.962**
Thornematidae	Tarsonemus	**-0.470**	**-0.701**	**4.348**	**3.485**
Eupodes	Arctoseius	**0.005**	**-0.152**	**3.786**	**3.485**
Eupodes	Cheiroseius	**0.005**	**0.356**	**3.786**	**3.184**
Eupodes	Aporcelaimellus	**0.005**	**0.548**	**3.786**	**5.251**
Eupodes	Dorylaimoidea	**0.005**	**-0.604**	**3.786**	**4.825**
Eupodes	Epidorylaimus	**0.005**	**0.199**	**3.786**	**4.348**
Eupodes	Qudsianematidae	**0.005**	**-0.207**	**3.786**	**4.825**
Eupodes	Thornematidae	**0.005**	**-0.470**	**3.786**	**4.348**
Eupodes	Eupodes	**0.005**	**0.005**	**3.786**	**3.786**
Eupodes	Scheloribates	**0.005**	**0.202**	**3.786**	**3.184**
Eupodes	Scutacarus	**0.005**	**-0.608**	**3.786**	**3.962**
Eupodes	Tarsonemus	**0.005**	**-0.701**	**3.786**	**3.485**

Scheloribates	Arctoseius	**0.202**	**-0.152**	**3.184**	**3.485**
Scheloribates	Cheiroseius	**0.202**	**0.356**	**3.184**	**3.184**
Scheloribates	Aporcelaimellus	**0.202**	**0.548**	**3.184**	**5.251**
Scheloribates	Dorylaimoidea	**0.202**	**-0.604**	**3.184**	**4.825**
Scheloribates	Epidorylaimus	**0.202**	**0.199**	**3.184**	**4.348**
Scheloribates	Qudsianematidae	**0.202**	**-0.207**	**3.184**	**4.825**
Scheloribates	Thornematidae	**0.202**	**-0.470**	**3.184**	**4.348**
Scheloribates	Eupodes	**0.202**	**0.005**	**3.184**	**3.786**
Scheloribates	Scheloribates	**0.202**	**0.202**	**3.184**	**3.184**
Scheloribates	Scutacarus	**0.202**	**-0.608**	**3.184**	**3.962**
Scheloribates	Tarsonemus	**0.202**	**-0.701**	**3.184**	**3.485**
Scutacarus	Arctoseius	**-0.608**	**-0.152**	**3.962**	**3.485**
Scutacarus	Cheiroseius	**-0.608**	**0.356**	**3.962**	**3.184**
Scutacarus	Aporcelaimellus	**-0.608**	**0.548**	**3.962**	**5.251**
Scutacarus	Dorylaimoidea	**-0.608**	**-0.604**	**3.962**	**4.825**
Scutacarus	Epidorylaimus	**-0.608**	**0.199**	**3.962**	**4.348**
Scutacarus	Qudsianematidae	**-0.608**	**-0.207**	**3.962**	**4.825**
Scutacarus	Thornematidae	**-0.608**	**-0.470**	**3.962**	**4.348**
Scutacarus	Eupodes	**-0.608**	**0.005**	**3.962**	**3.786**
Scutacarus	Scheloribates	**-0.608**	**0.202**	**3.962**	**3.184**
Scutacarus	Scutacarus	**-0.608**	**-0.608**	**3.962**	**3.962**
Scutacarus	Tarsonemus	**-0.608**	**-0.701**	**3.962**	**3.485**
Tarsonemus	Arctoseius	**-0.701**	**-0.152**	**3.485**	**3.485**
Tarsonemus	Cheiroseius	**-0.701**	**0.356**	**3.485**	**3.184**
Tarsonemus	Aporcelaimellus	**-0.701**	**0.548**	**3.485**	**5.251**
Tarsonemus	Dorylaimoidea	**-0.701**	**-0.604**	**3.485**	**4.825**
Tarsonemus	Epidorylaimus	**-0.701**	**0.199**	**3.485**	**4.348**
Tarsonemus	Qudsianematidae	**-0.701**	**-0.207**	**3.485**	**4.825**
Tarsonemus	Thornematidae	**-0.701**	**-0.470**	**3.485**	**4.348**
Tarsonemus	Eupodes	**-0.701**	**0.005**	**3.485**	**3.786**
Tarsonemus	Scheloribates	**-0.701**	**0.202**	**3.485**	**3.184**
Tarsonemus	Scutacarus	**-0.701**	**-0.608**	**3.485**	**3.962**
Tarsonemus	Tarsonemus	**-0.701**	**-0.701**	**3.485**	**3.485**

Resource	Consumer	Mres	Mconsumer	Nres	Nconsumer
Aglenchus	Mylonchulus	**-1.053**	**-0.005**	**4.850**	**4.548**
Aglenchus	Alliphis	**-1.053**	**0.053**	**4.850**	**2.968**
Aglenchus	Aporcelaimellus	**-1.053**	**0.548**	**4.850**	**5.247**
Aglenchus	Dorylaimoidea	**-1.053**	**-0.604**	**4.850**	**5.247**
Aglenchus	Qudsianematidae	**-1.053**	**-0.207**	**4.850**	**4.548**
Aglenchus	Thornematidae	**-1.053**	**-0.470**	**4.850**	**4.548**
Aglenchus	Eupodes	**-1.053**	**0.005**	**4.850**	**4.009**
Aglenchus	Scutacarus	**-1.053**	**-0.608**	**4.850**	**3.445**
Aglenchus	Stigmaeidae	**-1.053**	**0.229**	**4.850**	**2.968**
Aglenchus	Tarsonemus	**-1.053**	**-0.701**	**4.850**	**3.570**
Coslenchus	Mylonchulus	**-0.821**	**-0.005**	**4.548**	**4.548**
Coslenchus	Alliphis	**-0.821**	**0.053**	**4.548**	**2.968**
Coslenchus	Aporcelaimellus	**-0.821**	**0.548**	**4.548**	**5.247**
Coslenchus	Dorylaimoidea	**-0.821**	**-0.604**	**4.548**	**5.247**
Coslenchus	Qudsianematidae	**-0.821**	**-0.207**	**4.548**	**4.548**
Coslenchus	Thornematidae	**-0.821**	**-0.470**	**4.548**	**4.548**
Coslenchus	Eupodes	**-0.821**	**0.005**	**4.548**	**4.009**
Coslenchus	Scutacarus	**-0.821**	**-0.608**	**4.548**	**3.445**
Coslenchus	Stigmaeidae	**-0.821**	**0.229**	**4.548**	**2.968**
Coslenchus	Tarsonemus	**-0.821**	**-0.701**	**4.548**	**3.570**
Dolichodoridae	Mylonchulus	**-0.885**	**-0.005**	**5.026**	**4.548**
Dolichodoridae	Alliphis	**-0.885**	**0.053**	**5.026**	**2.968**
Dolichodoridae	Aporcelaimellus	**-0.885**	**0.548**	**5.026**	**5.247**
Dolichodoridae	Dorylaimoidea	**-0.885**	**-0.604**	**5.026**	**5.247**
Dolichodoridae	Qudsianematidae	**-0.885**	**-0.207**	**5.026**	**4.548**
Dolichodoridae	Thornematidae	**-0.885**	**-0.470**	**5.026**	**4.548**
Dolichodoridae	Eupodes	**-0.885**	**0.005**	**5.026**	**4.009**
Dolichodoridae	Scutacarus	**-0.885**	**-0.608**	**5.026**	**3.445**
Dolichodoridae	Stigmaeidae	**-0.885**	**0.229**	**5.026**	**2.968**
Dolichodoridae	Tarsonemus	**-0.885**	**-0.701**	**5.026**	**3.570**
Filenchus	Mylonchulus	**-1.033**	**-0.005**	**4.548**	**4.548**
Filenchus	Alliphis	**-1.033**	**0.053**	**4.548**	**2.968**
Filenchus	Aporcelaimellus	**-1.033**	**0.548**	**4.548**	**5.247**
Filenchus	Dorylaimoidea	**-1.033**	**-0.604**	**4.548**	**5.247**
Filenchus	Qudsianematidae	**-1.033**	**-0.207**	**4.548**	**4.548**
Filenchus	Thornematidae	**-1.033**	**-0.470**	**4.548**	**4.548**
Filenchus	Eupodes	**-1.033**	**0.005**	**4.548**	**4.009**
Filenchus	Scutacarus	**-1.033**	**-0.608**	**4.548**	**3.445**
Filenchus	Stigmaeidae	**-1.033**	**0.229**	**4.548**	**2.968**
Filenchus	Tarsonemus	**-1.033**	**-0.701**	**4.548**	**3.570**
Helicotylenchus	Mylonchulus	**-0.792**	**-0.005**	**4.850**	**4.548**
Helicotylenchus	Alliphis	**-0.792**	**0.053**	**4.850**	**2.968**
Helicotylenchus	Aporcelaimellus	**-0.792**	**0.548**	**4.850**	**5.247**
Helicotylenchus	Dorylaimoidea	**-0.792**	**-0.604**	**4.850**	**5.247**
Helicotylenchus	Qudsianematidae	**-0.792**	**-0.207**	**4.850**	**4.548**
Helicotylenchus	Thornematidae	**-0.792**	**-0.470**	**4.850**	**4.548**
Helicotylenchus	Eupodes	**-0.792**	**0.005**	**4.850**	**4.009**
Helicotylenchus	Scutacarus	**-0.792**	**-0.608**	**4.850**	**3.445**
Helicotylenchus	Stigmaeidae	**-0.792**	**0.229**	**4.850**	**2.968**
Helicotylenchus	Tarsonemus	**-0.792**	**-0.701**	**4.850**	**3.570**

Meloidogyne	Mylonchulus	**-1.287**	**-0.005**	**4.548**	**4.548**
Meloidogyne	Alliphis	**-1.287**	**0.053**	**4.548**	**2.968**
Meloidogyne	Aporcelaimellus	**-1.287**	**0.548**	**4.548**	**5.247**
Meloidogyne	Dorylaimoidea	**-1.287**	**-0.604**	**4.548**	**5.247**
Meloidogyne	Qudsianematidae	**-1.287**	**-0.207**	**4.548**	**4.548**
Meloidogyne	Thornematidae	**-1.287**	**-0.470**	**4.548**	**4.548**
Meloidogyne	Eupodes	**-1.287**	**0.005**	**4.548**	**4.009**
Meloidogyne	Scutacarus	**-1.287**	**-0.608**	**4.548**	**3.445**
Meloidogyne	Stigmaeidae	**-1.287**	**0.229**	**4.548**	**2.968**
Meloidogyne	Tarsonemus	**-1.287**	**-0.701**	**4.548**	**3.570**
Paratylenchus	Mylonchulus	**-1.244**	**-0.005**	**5.503**	**4.548**
Paratylenchus	Alliphis	**-1.244**	**0.053**	**5.503**	**2.968**
Paratylenchus	Aporcelaimellus	**-1.244**	**0.548**	**5.503**	**5.247**
Paratylenchus	Dorylaimoidea	**-1.244**	**-0.604**	**5.503**	**5.247**
Paratylenchus	Qudsianematidae	**-1.244**	**-0.207**	**5.503**	**4.548**
Paratylenchus	Thornematidae	**-1.244**	**-0.470**	**5.503**	**4.548**
Paratylenchus	Eupodes	**-1.244**	**0.005**	**5.503**	**4.009**
Paratylenchus	Scutacarus	**-1.244**	**-0.608**	**5.503**	**3.445**
Paratylenchus	Stigmaeidae	**-1.244**	**0.229**	**5.503**	**2.968**
Paratylenchus	Tarsonemus	**-1.244**	**-0.701**	**5.503**	**3.570**
Pratylenchus	Mylonchulus	**-1.226**	**-0.005**	**4.548**	**4.548**
Pratylenchus	Alliphis	**-1.226**	**0.053**	**4.548**	**2.968**
Pratylenchus	Aporcelaimellus	**-1.226**	**0.548**	**4.548**	**5.247**
Pratylenchus	Dorylaimoidea	**-1.226**	**-0.604**	**4.548**	**5.247**
Pratylenchus	Qudsianematidae	**-1.226**	**-0.207**	**4.548**	**4.548**
Pratylenchus	Thornematidae	**-1.226**	**-0.470**	**4.548**	**4.548**
Pratylenchus	Eupodes	**-1.226**	**0.005**	**4.548**	**4.009**
Pratylenchus	Scutacarus	**-1.226**	**-0.608**	**4.548**	**3.445**
Pratylenchus	Stigmaeidae	**-1.226**	**0.229**	**4.548**	**2.968**
Pratylenchus	Tarsonemus	**-1.226**	**-0.701**	**4.548**	**3.570**
Tylenchorhynchus	Mylonchulus	**-0.664**	**-0.005**	**5.628**	**4.548**
Tylenchorhynchus	Alliphis	**-0.664**	**0.053**	**5.628**	**2.968**
Tylenchorhynchus	Aporcelaimellus	**-0.664**	**0.548**	**5.628**	**5.247**
Tylenchorhynchus	Dorylaimoidea	**-0.664**	**-0.604**	**5.628**	**5.247**
Tylenchorhynchus	Qudsianematidae	**-0.664**	**-0.207**	**5.628**	**4.548**
Tylenchorhynchus	Thornematidae	**-0.664**	**-0.470**	**5.628**	**4.548**
Tylenchorhynchus	Eupodes	**-0.664**	**0.005**	**5.628**	**4.009**
Tylenchorhynchus	Scutacarus	**-0.664**	**-0.608**	**5.628**	**3.445**
Tylenchorhynchus	Stigmaeidae	**-0.664**	**0.229**	**5.628**	**2.968**
Tylenchorhynchus	Tarsonemus	**-0.664**	**-0.701**	**5.628**	**3.570**
Pachygnatidae	Aporcelaimellus	**-0.113**	**0.548**	**2.968**	**5.247**
Pachygnatidae	Dorylaimoidea	**-0.113**	**-0.604**	**2.968**	**5.247**
Pachygnatidae	Qudsianematidae	**-0.113**	**-0.207**	**2.968**	**4.548**
Pachygnatidae	Thornematidae	**-0.113**	**-0.470**	**2.968**	**4.548**
Pachygnatidae	Eupodes	**-0.113**	**0.005**	**2.968**	**4.009**
Pachygnatidae	Scutacarus	**-0.113**	**-0.608**	**2.968**	**3.445**
Pachygnatidae	Stigmaeidae	**-0.113**	**0.229**	**2.968**	**2.968**
Pachygnatidae	Tarsonemus	**-0.113**	**-0.701**	**2.968**	**3.570**
Tydeidae	Aporcelaimellus	**-0.608**	**0.548**	**3.813**	**5.247**
Tydeidae	Dorylaimoidea	**-0.608**	**-0.604**	**3.813**	**5.247**
Tydeidae	Qudsianematidae	**-0.608**	**-0.207**	**3.813**	**4.548**

Tydeidae	Thornematidae	**-0.608**	**-0.470**	**3.813**	**4.548**
Tydeidae	Eupodes	**-0.608**	**0.005**	**3.813**	**4.009**
Tydeidae	Scutacarus	**-0.608**	**-0.608**	**3.813**	**3.445**
Tydeidae	Stigmaeidae	**-0.608**	**0.229**	**3.813**	**2.968**
Tydeidae	Tarsonemus	**-0.608**	**-0.701**	**3.813**	**3.570**
Sminthuridae	Aporcelaimellus	**-0.608**	**0.548**	**2.968**	**5.247**
Sminthuridae	Dorylaimoidea	**-0.608**	**-0.604**	**2.968**	**5.247**
Sminthuridae	Qudsianematidae	**-0.608**	**-0.207**	**2.968**	**4.548**
Sminthuridae	Thornematidae	**-0.608**	**-0.470**	**2.968**	**4.548**
Sminthuridae	Eupodes	**-0.608**	**0.005**	**2.968**	**4.009**
Sminthuridae	Scutacarus	**-0.608**	**-0.608**	**2.968**	**3.445**
Sminthuridae	Stigmaeidae	**-0.608**	**0.229**	**2.968**	**2.968**
Sminthuridae	Tarsonemus	**-0.608**	**-0.701**	**2.968**	**3.570**
Sminthurinus	Aporcelaimellus	**0.618**	**0.548**	**3.746**	**5.247**
Sminthurinus	Dorylaimoidea	**0.618**	**-0.604**	**3.746**	**5.247**
Sminthurinus	Qudsianematidae	**0.618**	**-0.207**	**3.746**	**4.548**
Sminthurinus	Thornematidae	**0.618**	**-0.470**	**3.746**	**4.548**
Sminthurinus	Eupodes	**0.618**	**0.005**	**3.746**	**4.009**
Sminthurinus	Scutacarus	**0.618**	**-0.608**	**3.746**	**3.445**
Sminthurinus	Stigmaeidae	**0.618**	**0.229**	**3.746**	**2.968**
Sminthurinus	Tarsonemus	**0.618**	**-0.701**	**3.746**	**3.570**
Sminthurus	Aporcelaimellus	**1.429**	**0.548**	**2.968**	**5.247**
Sminthurus	Dorylaimoidea	**1.429**	**-0.604**	**2.968**	**5.247**
Sminthurus	Qudsianematidae	**1.429**	**-0.207**	**2.968**	**4.548**
Sminthurus	Thornematidae	**1.429**	**-0.470**	**2.968**	**4.548**
Sminthurus	Eupodes	**1.429**	**0.005**	**2.968**	**4.009**
Sminthurus	Scutacarus	**1.429**	**-0.608**	**2.968**	**3.445**
Sminthurus	Stigmaeidae	**1.429**	**0.229**	**2.968**	**2.968**
Sminthurus	Tarsonemus	**1.429**	**-0.701**	**2.968**	**3.570**
Sphaeridia	Aporcelaimellus	**0.202**	**0.548**	**3.570**	**5.247**
Sphaeridia	Dorylaimoidea	**0.202**	**-0.604**	**3.570**	**5.247**
Sphaeridia	Qudsianematidae	**0.202**	**-0.207**	**3.570**	**4.548**
Sphaeridia	Thornematidae	**0.202**	**-0.470**	**3.570**	**4.548**
Sphaeridia	Eupodes	**0.202**	**0.005**	**3.570**	**4.009**
Sphaeridia	Scutacarus	**0.202**	**-0.608**	**3.570**	**3.445**
Sphaeridia	Stigmaeidae	**0.202**	**0.229**	**3.570**	**2.968**
Sphaeridia	Tarsonemus	**0.202**	**-0.701**	**3.570**	**3.570**
Aphelenchoides	Mylonchulus	**-1.496**	**-0.005**	**5.247**	**4.548**
Aphelenchoides	Alliphis	**-1.496**	**0.053**	**5.247**	**2.968**
Aphelenchoides	Aporcelaimellus	**-1.496**	**0.548**	**5.247**	**5.247**
Aphelenchoides	Dorylaimoidea	**-1.496**	**-0.604**	**5.247**	**5.247**
Aphelenchoides	Qudsianematidae	**-1.496**	**-0.207**	**5.247**	**4.548**
Aphelenchoides	Thornematidae	**-1.496**	**-0.470**	**5.247**	**4.548**
Aphelenchoides	Eupodes	**-1.496**	**0.005**	**5.247**	**4.009**
Aphelenchoides	Scutacarus	**-1.496**	**-0.608**	**5.247**	**3.445**
Aphelenchoides	Stigmaeidae	**-1.496**	**0.229**	**5.247**	**2.968**
Aphelenchoides	Tarsonemus	**-1.496**	**-0.701**	**5.247**	**3.570**
Tylenchidae	Mylonchulus	**-1.360**	**-0.005**	**5.827**	**4.548**
Tylenchidae	Alliphis	**-1.360**	**0.053**	**5.827**	**2.968**
Tylenchidae	Aporcelaimellus	**-1.360**	**0.548**	**5.827**	**5.247**
Tylenchidae	Dorylaimoidea	**-1.360**	**-0.604**	**5.827**	**5.247**

Tylenchidae	Qudsianematidae	**-1.360**	**-0.207**	**5.827**	**4.548**
Tylenchidae	Thornematidae	**-1.360**	**-0.470**	**5.827**	**4.548**
Tylenchidae	Eupodes	**-1.360**	**0.005**	**5.827**	**4.009**
Tylenchidae	Scutacarus	**-1.360**	**-0.608**	**5.827**	**3.445**
Tylenchidae	Stigmaeidae	**-1.360**	**0.229**	**5.827**	**2.968**
Tylenchidae	Tarsonemus	**-1.360**	**-0.701**	**5.827**	**3.570**
Brachychthoniidae	Aporcelaimellus	**-0.586**	**0.548**	**3.269**	**5.247**
Brachychthoniidae	Dorylaimoidea	**-0.586**	**-0.604**	**3.269**	**5.247**
Brachychthoniidae	Qudsianematidae	**-0.586**	**-0.207**	**3.269**	**4.548**
Brachychthoniidae	Thornematidae	**-0.586**	**-0.470**	**3.269**	**4.548**
Brachychthoniidae	Eupodes	**-0.586**	**0.005**	**3.269**	**4.009**
Brachychthoniidae	Scutacarus	**-0.586**	**-0.608**	**3.269**	**3.445**
Brachychthoniidae	Stigmaeidae	**-0.586**	**0.229**	**3.269**	**2.968**
Brachychthoniidae	Tarsonemus	**-0.586**	**-0.701**	**3.269**	**3.570**
Microtydeus	Aporcelaimellus	**-0.863**	**0.548**	**2.968**	**5.247**
Microtydeus	Dorylaimoidea	**-0.863**	**-0.604**	**2.968**	**5.247**
Microtydeus	Qudsianematidae	**-0.863**	**-0.207**	**2.968**	**4.548**
Microtydeus	Thornematidae	**-0.863**	**-0.470**	**2.968**	**4.548**
Microtydeus	Eupodes	**-0.863**	**0.005**	**2.968**	**4.009**
Microtydeus	Scutacarus	**-0.863**	**-0.608**	**2.968**	**3.445**
Microtydeus	Stigmaeidae	**-0.863**	**0.229**	**2.968**	**2.968**
Microtydeus	Tarsonemus	**-0.863**	**-0.701**	**2.968**	**3.570**
Pygmephorus	Aporcelaimellus	**-0.376**	**0.548**	**3.445**	**5.247**
Pygmephorus	Dorylaimoidea	**-0.376**	**-0.604**	**3.445**	**5.247**
Pygmephorus	Qudsianematidae	**-0.376**	**-0.207**	**3.445**	**4.548**
Pygmephorus	Thornematidae	**-0.376**	**-0.470**	**3.445**	**4.548**
Pygmephorus	Eupodes	**-0.376**	**0.005**	**3.445**	**4.009**
Pygmephorus	Scutacarus	**-0.376**	**-0.608**	**3.445**	**3.445**
Pygmephorus	Stigmaeidae	**-0.376**	**0.229**	**3.445**	**2.968**
Pygmephorus	Tarsonemus	**-0.376**	**-0.701**	**3.445**	**3.570**
Tectocepheus	Aporcelaimellus	**-0.220**	**0.548**	**2.968**	**5.247**
Tectocepheus	Dorylaimoidea	**-0.220**	**-0.604**	**2.968**	**5.247**
Tectocepheus	Qudsianematidae	**-0.220**	**-0.207**	**2.968**	**4.548**
Tectocepheus	Thornematidae	**-0.220**	**-0.470**	**2.968**	**4.548**
Tectocepheus	Eupodes	**-0.220**	**0.005**	**2.968**	**4.009**
Tectocepheus	Scutacarus	**-0.220**	**-0.608**	**2.968**	**3.445**
Tectocepheus	Stigmaeidae	**-0.220**	**0.229**	**2.968**	**2.968**
Tectocepheus	Tarsonemus	**-0.220**	**-0.701**	**2.968**	**3.570**
Tyrophagus	Aporcelaimellus	**0.005**	**0.548**	**3.813**	**5.247**
Tyrophagus	Dorylaimoidea	**0.005**	**-0.604**	**3.813**	**5.247**
Tyrophagus	Qudsianematidae	**0.005**	**-0.207**	**3.813**	**4.548**
Tyrophagus	Thornematidae	**0.005**	**-0.470**	**3.813**	**4.548**
Tyrophagus	Eupodes	**0.005**	**0.005**	**3.813**	**4.009**
Tyrophagus	Scutacarus	**0.005**	**-0.608**	**3.813**	**3.445**
Tyrophagus	Stigmaeidae	**0.005**	**0.229**	**3.813**	**2.968**
Tyrophagus	Tarsonemus	**0.005**	**-0.701**	**3.813**	**3.570**
Ceratophysella	Aporcelaimellus	**1.335**	**0.548**	**2.968**	**5.247**
Ceratophysella	Dorylaimoidea	**1.335**	**-0.604**	**2.968**	**5.247**
Ceratophysella	Qudsianematidae	**1.335**	**-0.207**	**2.968**	**4.548**
Ceratophysella	Thornematidae	**1.335**	**-0.470**	**2.968**	**4.548**
Ceratophysella	Eupodes	**1.335**	**0.005**	**2.968**	**4.009**

Ceratophysella	Scutacarus	**1.335**	**-0.608**	**2.968**	**3.445**
Ceratophysella	Stigmaeidae	**1.335**	**0.229**	**2.968**	**2.968**
Ceratophysella	Tarsonemus	**1.335**	**-0.701**	**2.968**	**3.570**
Isotoma	Aporcelaimellus	**1.898**	**0.548**	**3.871**	**5.247**
Isotoma	Dorylaimoidea	**1.898**	**-0.604**	**3.871**	**5.247**
Isotoma	Qudsianematidae	**1.898**	**-0.207**	**3.871**	**4.548**
Isotoma	Thornematidae	**1.898**	**-0.470**	**3.871**	**4.548**
Isotoma	Eupodes	**1.898**	**0.005**	**3.871**	**4.009**
Isotoma	Scutacarus	**1.898**	**-0.608**	**3.871**	**3.445**
Isotoma	Stigmaeidae	**1.898**	**0.229**	**3.871**	**2.968**
Isotoma	Tarsonemus	**1.898**	**-0.701**	**3.871**	**3.570**
Isotomiella	Aporcelaimellus	**0.816**	**0.548**	**2.968**	**5.247**
Isotomiella	Dorylaimoidea	**0.816**	**-0.604**	**2.968**	**5.247**
Isotomiella	Qudsianematidae	**0.816**	**-0.207**	**2.968**	**4.548**
Isotomiella	Thornematidae	**0.816**	**-0.470**	**2.968**	**4.548**
Isotomiella	Eupodes	**0.816**	**0.005**	**2.968**	**4.009**
Isotomiella	Scutacarus	**0.816**	**-0.608**	**2.968**	**3.445**
Isotomiella	Stigmaeidae	**0.816**	**0.229**	**2.968**	**2.968**
Isotomiella	Tarsonemus	**0.816**	**-0.701**	**2.968**	**3.570**
Proisotoma	Aporcelaimellus	**0.770**	**0.548**	**3.746**	**5.247**
Proisotoma	Dorylaimoidea	**0.770**	**-0.604**	**3.746**	**5.247**
Proisotoma	Qudsianematidae	**0.770**	**-0.207**	**3.746**	**4.548**
Proisotoma	Thornematidae	**0.770**	**-0.470**	**3.746**	**4.548**
Proisotoma	Eupodes	**0.770**	**0.005**	**3.746**	**4.009**
Proisotoma	Scutacarus	**0.770**	**-0.608**	**3.746**	**3.445**
Proisotoma	Stigmaeidae	**0.770**	**0.229**	**3.746**	**2.968**
Proisotoma	Tarsonemus	**0.770**	**-0.701**	**3.746**	**3.570**
Achaeta	Aporcelaimellus	**0.826**	**0.548**	**2.884**	**5.247**
Achaeta	Dorylaimoidea	**0.826**	**-0.604**	**2.884**	**5.247**
Achaeta	Qudsianematidae	**0.826**	**-0.207**	**2.884**	**4.548**
Achaeta	Thornematidae	**0.826**	**-0.470**	**2.884**	**4.548**
Achaeta	Eupodes	**0.826**	**0.005**	**2.884**	**4.009**
Achaeta	Scutacarus	**0.826**	**-0.608**	**2.884**	**3.445**
Achaeta	Stigmaeidae	**0.826**	**0.229**	**2.884**	**2.968**
Achaeta	Tarsonemus	**0.826**	**-0.701**	**2.884**	**3.570**
Fridericia	Aporcelaimellus	**2.296**	**0.548**	**3.556**	**5.247**
Fridericia	Dorylaimoidea	**2.296**	**-0.604**	**3.556**	**5.247**
Fridericia	Qudsianematidae	**2.296**	**-0.207**	**3.556**	**4.548**
Fridericia	Thornematidae	**2.296**	**-0.470**	**3.556**	**4.548**
Fridericia	Eupodes	**2.296**	**0.005**	**3.556**	**4.009**
Fridericia	Scutacarus	**2.296**	**-0.608**	**3.556**	**3.445**
Fridericia	Stigmaeidae	**2.296**	**0.229**	**3.556**	**2.968**
Fridericia	Tarsonemus	**2.296**	**-0.701**	**3.556**	**3.570**
Acrobeles	Mylonchulus	**-0.721**	**-0.005**	**5.026**	**4.548**
Acrobeles	Alliphis	**-0.721**	**0.053**	**5.026**	**2.968**
Acrobeles	Aporcelaimellus	**-0.721**	**0.548**	**5.026**	**5.247**
Acrobeles	Dorylaimoidea	**-0.721**	**-0.604**	**5.026**	**5.247**
Acrobeles	Qudsianematidae	**-0.721**	**-0.207**	**5.026**	**4.548**
Acrobeles	Thornematidae	**-0.721**	**-0.470**	**5.026**	**4.548**
Acrobeles	Eupodes	**-0.721**	**0.005**	**5.026**	**4.009**
Acrobeles	Scutacarus	**-0.721**	**-0.608**	**5.026**	**3.445**

Acrobeles	Stigmaeidae	**-0.721**	**0.229**	**5.026**	**2.968**
Acrobeles	Tarsonemus	**-0.721**	**-0.701**	**5.026**	**3.570**
Acrobeloides	Mylonchulus	**-1.171**	**-0.005**	**5.247**	**4.548**
Acrobeloides	Alliphis	**-1.171**	**0.053**	**5.247**	**2.968**
Acrobeloides	Aporcelaimellus	**-1.171**	**0.548**	**5.247**	**5.247**
Acrobeloides	Dorylaimoidea	**-1.171**	**-0.604**	**5.247**	**5.247**
Acrobeloides	Qudsianematidae	**-1.171**	**-0.207**	**5.247**	**4.548**
Acrobeloides	Thornematidae	**-1.171**	**-0.470**	**5.247**	**4.548**
Acrobeloides	Eupodes	**-1.171**	**0.005**	**5.247**	**4.009**
Acrobeloides	Scutacarus	**-1.171**	**-0.608**	**5.247**	**3.445**
Acrobeloides	Stigmaeidae	**-1.171**	**0.229**	**5.247**	**2.968**
Acrobeloides	Tarsonemus	**-1.171**	**-0.701**	**5.247**	**3.570**
Anaplectus	Mylonchulus	**-0.519**	**-0.005**	**5.026**	**4.548**
Anaplectus	Alliphis	**-0.519**	**0.053**	**5.026**	**2.968**
Anaplectus	Aporcelaimellus	**-0.519**	**0.548**	**5.026**	**5.247**
Anaplectus	Dorylaimoidea	**-0.519**	**-0.604**	**5.026**	**5.247**
Anaplectus	Qudsianematidae	**-0.519**	**-0.207**	**5.026**	**4.548**
Anaplectus	Thornematidae	**-0.519**	**-0.470**	**5.026**	**4.548**
Anaplectus	Eupodes	**-0.519**	**0.005**	**5.026**	**4.009**
Anaplectus	Scutacarus	**-0.519**	**-0.608**	**5.026**	**3.445**
Anaplectus	Stigmaeidae	**-0.519**	**0.229**	**5.026**	**2.968**
Anaplectus	Tarsonemus	**-0.519**	**-0.701**	**5.026**	**3.570**
Cephalobidae	Mylonchulus	**-1.055**	**-0.005**	**5.247**	**4.548**
Cephalobidae	Alliphis	**-1.055**	**0.053**	**5.247**	**2.968**
Cephalobidae	Aporcelaimellus	**-1.055**	**0.548**	**5.247**	**5.247**
Cephalobidae	Dorylaimoidea	**-1.055**	**-0.604**	**5.247**	**5.247**
Cephalobidae	Qudsianematidae	**-1.055**	**-0.207**	**5.247**	**4.548**
Cephalobidae	Thornematidae	**-1.055**	**-0.470**	**5.247**	**4.548**
Cephalobidae	Eupodes	**-1.055**	**0.005**	**5.247**	**4.009**
Cephalobidae	Scutacarus	**-1.055**	**-0.608**	**5.247**	**3.445**
Cephalobidae	Stigmaeidae	**-1.055**	**0.229**	**5.247**	**2.968**
Cephalobidae	Tarsonemus	**-1.055**	**-0.701**	**5.247**	**3.570**
Eucephalobus	Mylonchulus	**-0.855**	**-0.005**	**5.590**	**4.548**
Eucephalobus	Alliphis	**-0.855**	**0.053**	**5.590**	**2.968**
Eucephalobus	Aporcelaimellus	**-0.855**	**0.548**	**5.590**	**5.247**
Eucephalobus	Dorylaimoidea	**-0.855**	**-0.604**	**5.590**	**5.247**
Eucephalobus	Qudsianematidae	**-0.855**	**-0.207**	**5.590**	**4.548**
Eucephalobus	Thornematidae	**-0.855**	**-0.470**	**5.590**	**4.548**
Eucephalobus	Eupodes	**-0.855**	**0.005**	**5.590**	**4.009**
Eucephalobus	Scutacarus	**-0.855**	**-0.608**	**5.590**	**3.445**
Eucephalobus	Stigmaeidae	**-0.855**	**0.229**	**5.590**	**2.968**
Eucephalobus	Tarsonemus	**-0.855**	**-0.701**	**5.590**	**3.570**
Metateratocephalus	Mylonchulus	**-1.506**	**-0.005**	**4.548**	**4.548**
Metateratocephalus	Alliphis	**-1.506**	**0.053**	**4.548**	**2.968**
Metateratocephalus	Aporcelaimellus	**-1.506**	**0.548**	**4.548**	**5.247**
Metateratocephalus	Dorylaimoidea	**-1.506**	**-0.604**	**4.548**	**5.247**
Metateratocephalus	Qudsianematidae	**-1.506**	**-0.207**	**4.548**	**4.548**
Metateratocephalus	Thornematidae	**-1.506**	**-0.470**	**4.548**	**4.548**
Metateratocephalus	Eupodes	**-1.506**	**0.005**	**4.548**	**4.009**
Metateratocephalus	Scutacarus	**-1.506**	**-0.608**	**4.548**	**3.445**
Metateratocephalus	Stigmaeidae	**-1.506**	**0.229**	**4.548**	**2.968**

Metateratocephalus	Tarsonemus	**-1.506**	**-0.701**	**4.548**	**3.570**
Panagrolaimus	Mylonchulus	**-0.945**	**-0.005**	**5.503**	**4.548**
Panagrolaimus	Alliphis	**-0.945**	**0.053**	**5.503**	**2.968**
Panagrolaimus	Aporcelaimellus	**-0.945**	**0.548**	**5.503**	**5.247**
Panagrolaimus	Dorylaimoidea	**-0.945**	**-0.604**	**5.503**	**5.247**
Panagrolaimus	Qudsianematidae	**-0.945**	**-0.207**	**5.503**	**4.548**
Panagrolaimus	Thornematidae	**-0.945**	**-0.470**	**5.503**	**4.548**
Panagrolaimus	Eupodes	**-0.945**	**0.005**	**5.503**	**4.009**
Panagrolaimus	Scutacarus	**-0.945**	**-0.608**	**5.503**	**3.445**
Panagrolaimus	Stigmaeidae	**-0.945**	**0.229**	**5.503**	**2.968**
Panagrolaimus	Tarsonemus	**-0.945**	**-0.701**	**5.503**	**3.570**
Paramphidelus	Mylonchulus	**-1.055**	**-0.005**	**4.548**	**4.548**
Paramphidelus	Alliphis	**-1.055**	**0.053**	**4.548**	**2.968**
Paramphidelus	Aporcelaimellus	**-1.055**	**0.548**	**4.548**	**5.247**
Paramphidelus	Dorylaimoidea	**-1.055**	**-0.604**	**4.548**	**5.247**
Paramphidelus	Qudsianematidae	**-1.055**	**-0.207**	**4.548**	**4.548**
Paramphidelus	Thornematidae	**-1.055**	**-0.470**	**4.548**	**4.548**
Paramphidelus	Eupodes	**-1.055**	**0.005**	**4.548**	**4.009**
Paramphidelus	Scutacarus	**-1.055**	**-0.608**	**4.548**	**3.445**
Paramphidelus	Stigmaeidae	**-1.055**	**0.229**	**4.548**	**2.968**
Paramphidelus	Tarsonemus	**-1.055**	**-0.701**	**4.548**	**3.570**
Plectus	Mylonchulus	**-0.583**	**-0.005**	**5.327**	**4.548**
Plectus	Alliphis	**-0.583**	**0.053**	**5.327**	**2.968**
Plectus	Aporcelaimellus	**-0.583**	**0.548**	**5.327**	**5.247**
Plectus	Dorylaimoidea	**-0.583**	**-0.604**	**5.327**	**5.247**
Plectus	Qudsianematidae	**-0.583**	**-0.207**	**5.327**	**4.548**
Plectus	Thornematidae	**-0.583**	**-0.470**	**5.327**	**4.548**
Plectus	Eupodes	**-0.583**	**0.005**	**5.327**	**4.009**
Plectus	Scutacarus	**-0.583**	**-0.608**	**5.327**	**3.445**
Plectus	Stigmaeidae	**-0.583**	**0.229**	**5.327**	**2.968**
Plectus	Tarsonemus	**-0.583**	**-0.701**	**5.327**	**3.570**
Prismatolaimus	Mylonchulus	**-1.280**	**-0.005**	**5.327**	**4.548**
Prismatolaimus	Alliphis	**-1.280**	**0.053**	**5.327**	**2.968**
Prismatolaimus	Aporcelaimellus	**-1.280**	**0.548**	**5.327**	**5.247**
Prismatolaimus	Dorylaimoidea	**-1.280**	**-0.604**	**5.327**	**5.247**
Prismatolaimus	Qudsianematidae	**-1.280**	**-0.207**	**5.327**	**4.548**
Prismatolaimus	Thornematidae	**-1.280**	**-0.470**	**5.327**	**4.548**
Prismatolaimus	Eupodes	**-1.280**	**0.005**	**5.327**	**4.009**
Prismatolaimus	Scutacarus	**-1.280**	**-0.608**	**5.327**	**3.445**
Prismatolaimus	Stigmaeidae	**-1.280**	**0.229**	**5.327**	**2.968**
Prismatolaimus	Tarsonemus	**-1.280**	**-0.701**	**5.327**	**3.570**
Rhabditidae	Mylonchulus	**-0.692**	**-0.005**	**5.725**	**4.548**
Rhabditidae	Alliphis	**-0.692**	**0.053**	**5.725**	**2.968**
Rhabditidae	Aporcelaimellus	**-0.692**	**0.548**	**5.725**	**5.247**
Rhabditidae	Dorylaimoidea	**-0.692**	**-0.604**	**5.725**	**5.247**
Rhabditidae	Qudsianematidae	**-0.692**	**-0.207**	**5.725**	**4.548**
Rhabditidae	Thornematidae	**-0.692**	**-0.470**	**5.725**	**4.548**
Rhabditidae	Eupodes	**-0.692**	**0.005**	**5.725**	**4.009**
Rhabditidae	Scutacarus	**-0.692**	**-0.608**	**5.725**	**3.445**
Rhabditidae	Stigmaeidae	**-0.692**	**0.229**	**5.725**	**2.968**
Rhabditidae	Tarsonemus	**-0.692**	**-0.701**	**5.725**	**3.570**

Teratocephalus	Mylonchulus	**-1.630**	**-0.005**	**5.394**	**4.548**
Teratocephalus	Alliphis	**-1.630**	**0.053**	**5.394**	**2.968**
Teratocephalus	Aporcelaimellus	**-1.630**	**0.548**	**5.394**	**5.247**
Teratocephalus	Dorylaimoidea	**-1.630**	**-0.604**	**5.394**	**5.247**
Teratocephalus	Qudsianematidae	**-1.630**	**-0.207**	**5.394**	**4.548**
Teratocephalus	Thornematidae	**-1.630**	**-0.470**	**5.394**	**4.548**
Teratocephalus	Eupodes	**-1.630**	**0.005**	**5.394**	**4.009**
Teratocephalus	Scutacarus	**-1.630**	**-0.608**	**5.394**	**3.445**
Teratocephalus	Stigmaeidae	**-1.630**	**0.229**	**5.394**	**2.968**
Teratocephalus	Tarsonemus	**-1.630**	**-0.701**	**5.394**	**3.570**
Enchytraeus	Aporcelaimellus	**1.009**	**0.548**	**3.001**	**5.247**
Enchytraeus	Dorylaimoidea	**1.009**	**-0.604**	**3.001**	**5.247**
Enchytraeus	Qudsianematidae	**1.009**	**-0.207**	**3.001**	**4.548**
Enchytraeus	Thornematidae	**1.009**	**-0.470**	**3.001**	**4.548**
Enchytraeus	Eupodes	**1.009**	**0.005**	**3.001**	**4.009**
Enchytraeus	Scutacarus	**1.009**	**-0.608**	**3.001**	**3.445**
Enchytraeus	Stigmaeidae	**1.009**	**0.229**	**3.001**	**2.968**
Enchytraeus	Tarsonemus	**1.009**	**-0.701**	**3.001**	**3.570**
Eubacteria	Acrobeles	**-6.673**	**-0.721**	**12.237**	**5.026**
Eubacteria	Acrobeloides	**-6.673**	**-1.171**	**12.237**	**5.247**
Eubacteria	Anaplectus	**-6.673**	**-0.519**	**12.237**	**5.026**
Eubacteria	Cephalobidae	**-6.673**	**-1.055**	**12.237**	**5.247**
Eubacteria	Eucephalobus	**-6.673**	**-0.855**	**12.237**	**5.590**
Eubacteria	Metateratocephalus	**-6.673**	**-1.506**	**12.237**	**4.548**
Eubacteria	Panagrolaimus	**-6.673**	**-0.945**	**12.237**	**5.503**
Eubacteria	Paramphidelus	**-6.673**	**-1.055**	**12.237**	**4.548**
Eubacteria	Plectus	**-6.673**	**-0.583**	**12.237**	**5.327**
Eubacteria	Prismatolaimus	**-6.673**	**-1.280**	**12.237**	**5.327**
Eubacteria	Rhabditidae	**-6.673**	**-0.692**	**12.237**	**5.725**
Eubacteria	Teratocephalus	**-6.673**	**-1.630**	**12.237**	**5.394**
Eubacteria	Enchytraeus	**-6.673**	**1.009**	**12.237**	**3.001**
Eubacteria	Dauerlarvae	**-6.673**	**-0.804**	**12.237**	**5.503**
Eubacteria	Henlea	**-6.673**	**1.773**	**12.237**	**3.549**
Eubacteria	Marionina	**-6.673**	**0.530**	**12.237**	**2.373**
Dauerlarvae	Mylonchulus	**-0.804**	**-0.005**	**5.503**	**4.548**
Dauerlarvae	Alliphis	**-0.804**	**0.053**	**5.503**	**2.968**
Dauerlarvae	Aporcelaimellus	**-0.804**	**0.548**	**5.503**	**5.247**
Dauerlarvae	Dorylaimoidea	**-0.804**	**-0.604**	**5.503**	**5.247**
Dauerlarvae	Qudsianematidae	**-0.804**	**-0.207**	**5.503**	**4.548**
Dauerlarvae	Thornematidae	**-0.804**	**-0.470**	**5.503**	**4.548**
Dauerlarvae	Eupodes	**-0.804**	**0.005**	**5.503**	**4.009**
Dauerlarvae	Scutacarus	**-0.804**	**-0.608**	**5.503**	**3.445**
Dauerlarvae	Stigmaeidae	**-0.804**	**0.229**	**5.503**	**2.968**
Dauerlarvae	Tarsonemus	**-0.804**	**-0.701**	**5.503**	**3.570**
Henlea	Aporcelaimellus	**1.773**	**0.548**	**3.549**	**5.247**
Henlea	Dorylaimoidea	**1.773**	**-0.604**	**3.549**	**5.247**
Henlea	Qudsianematidae	**1.773**	**-0.207**	**3.549**	**4.548**
Henlea	Thornematidae	**1.773**	**-0.470**	**3.549**	**4.548**
Henlea	Eupodes	**1.773**	**0.005**	**3.549**	**4.009**
Henlea	Scutacarus	**1.773**	**-0.608**	**3.549**	**3.445**
Henlea	Stigmaeidae	**1.773**	**0.229**	**3.549**	**2.968**

Henlea	Tarsonemus	**1.773**	**-0.701**	**3.549**	**3.570**
Marionina	Aporcelaimellus	**0.530**	**0.548**	**2.373**	**5.247**
Marionina	Dorylaimoidea	**0.530**	**-0.604**	**2.373**	**5.247**
Marionina	Qudsianematidae	**0.530**	**-0.207**	**2.373**	**4.548**
Marionina	Thornematidae	**0.530**	**-0.470**	**2.373**	**4.548**
Marionina	Eupodes	**0.530**	**0.005**	**2.373**	**4.009**
Marionina	Scutacarus	**0.530**	**-0.608**	**2.373**	**3.445**
Marionina	Stigmaeidae	**0.530**	**0.229**	**2.373**	**2.968**
Marionina	Tarsonemus	**0.530**	**-0.701**	**2.373**	**3.570**
Hyphae and hair roots	Aglenchus	**5.951**	**-1.053**	**0.000**	**4.850**
Hyphae and hair roots	Coslenchus	**5.951**	**-0.821**	**0.000**	**4.548**
Hyphae and hair roots	Dolichodoridae	**5.951**	**-0.885**	**0.000**	**5.026**
Hyphae and hair roots	Filenchus	**5.951**	**-1.033**	**0.000**	**4.548**
Hyphae and hair roots	Helicotylenchus	**5.951**	**-0.792**	**0.000**	**4.850**
Hyphae and hair roots	Meloidogyne	**5.951**	**-1.287**	**0.000**	**4.548**
Hyphae and hair roots	Paratylenchus	**5.951**	**-1.244**	**0.000**	**5.503**
Hyphae and hair roots	Pratylenchus	**5.951**	**-1.226**	**0.000**	**4.548**
Hyphae and hair roots	Tylenchorhynchus	**5.951**	**-0.664**	**0.000**	**5.628**
Hyphae and hair roots	Pachygnatidae	**5.951**	**-0.113**	**0.000**	**2.968**
Hyphae and hair roots	Tydeidae	**5.951**	**-0.608**	**0.000**	**3.813**
Hyphae and hair roots	Sminthuridae	**5.951**	**-0.608**	**0.000**	**2.968**
Hyphae and hair roots	Sminthurinus	**5.951**	**0.618**	**0.000**	**3.746**
Hyphae and hair roots	Sminthurus	**5.951**	**1.429**	**0.000**	**2.968**
Hyphae and hair roots	Sphaeridia	**5.951**	**0.202**	**0.000**	**3.570**
Hyphae and hair roots	Aphelenchoides	**5.951**	**-1.496**	**0.000**	**5.247**
Hyphae and hair roots	Tylenchidae	**5.951**	**-1.360**	**0.000**	**5.827**
Hyphae and hair roots	Brachychthoniidae	**5.951**	**-0.586**	**0.000**	**3.269**
Hyphae and hair roots	Microtydeus	**5.951**	**-0.863**	**0.000**	**2.968**
Hyphae and hair roots	Pygmephorus	**5.951**	**-0.376**	**0.000**	**3.445**
Hyphae and hair roots	Tectocepheus	**5.951**	**-0.220**	**0.000**	**2.968**
Hyphae and hair roots	Tyrophagus	**5.951**	**0.005**	**0.000**	**3.813**
Hyphae and hair roots	Ceratophysella	**5.951**	**1.335**	**0.000**	**2.968**
Hyphae and hair roots	Isotoma	**5.951**	**1.898**	**0.000**	**3.871**
Hyphae and hair roots	Isotomiella	**5.951**	**0.816**	**0.000**	**2.968**
Hyphae and hair roots	Proisotoma	**5.951**	**0.770**	**0.000**	**3.746**
Hyphae and hair roots	Achaeta	**5.951**	**0.826**	**0.000**	**2.884**
Hyphae and hair roots	Fridericia	**5.951**	**2.296**	**0.000**	**3.556**
Hyphae and hair roots	Aporcelaimellus	**5.951**	**0.548**	**0.000**	**5.247**
Hyphae and hair roots	Dorylaimoidea	**5.951**	**-0.604**	**0.000**	**5.247**
Hyphae and hair roots	Qudsianematidae	**5.951**	**-0.207**	**0.000**	**4.548**
Hyphae and hair roots	Thornematidae	**5.951**	**-0.470**	**0.000**	**4.548**
Hyphae and hair roots	Eupodes	**5.951**	**0.005**	**0.000**	**4.009**
Hyphae and hair roots	Scutacarus	**5.951**	**-0.608**	**0.000**	**3.445**
Hyphae and hair roots	Stigmaeidae	**5.951**	**0.229**	**0.000**	**2.968**
Hyphae and hair roots	Tarsonemus	**5.951**	**-0.701**	**0.000**	**3.570**
Mylonchulus	Aporcelaimellus	**-0.005**	**0.548**	**4.548**	**5.247**
Mylonchulus	Dorylaimoidea	**-0.005**	**-0.604**	**4.548**	**5.247**
Mylonchulus	Qudsianematidae	**-0.005**	**-0.207**	**4.548**	**4.548**
Mylonchulus	Thornematidae	**-0.005**	**-0.470**	**4.548**	**4.548**
Mylonchulus	Eupodes	**-0.005**	**0.005**	**4.548**	**4.009**
Mylonchulus	Scutacarus	**-0.005**	**-0.608**	**4.548**	**3.445**

Mylonchulus	Stigmaeidae	**-0.005**	**0.229**	**4.548**	**2.968**
Mylonchulus	Tarsonemus	**-0.005**	**-0.701**	**4.548**	**3.570**
Alliphis	Aporcelaimellus	**0.053**	**0.548**	**2.968**	**5.247**
Alliphis	Dorylaimoidea	**0.053**	**-0.604**	**2.968**	**5.247**
Alliphis	Qudsianematidae	**0.053**	**-0.207**	**2.968**	**4.548**
Alliphis	Thornematidae	**0.053**	**-0.470**	**2.968**	**4.548**
Alliphis	Eupodes	**0.053**	**0.005**	**2.968**	**4.009**
Alliphis	Scutacarus	**0.053**	**-0.608**	**2.968**	**3.445**
Alliphis	Stigmaeidae	**0.053**	**0.229**	**2.968**	**2.968**
Alliphis	Tarsonemus	**0.053**	**-0.701**	**2.968**	**3.570**
Aporcelaimellus	Mylonchulus	**0.548**	**-0.005**	**5.247**	**4.548**
Aporcelaimellus	Alliphis	**0.548**	**0.053**	**5.247**	**2.968**
Aporcelaimellus	Aporcelaimellus	**0.548**	**0.548**	**5.247**	**5.247**
Aporcelaimellus	Dorylaimoidea	**0.548**	**-0.604**	**5.247**	**5.247**
Aporcelaimellus	Qudsianematidae	**0.548**	**-0.207**	**5.247**	**4.548**
Aporcelaimellus	Thornematidae	**0.548**	**-0.470**	**5.247**	**4.548**
Aporcelaimellus	Eupodes	**0.548**	**0.005**	**5.247**	**4.009**
Aporcelaimellus	Scutacarus	**0.548**	**-0.608**	**5.247**	**3.445**
Aporcelaimellus	Stigmaeidae	**0.548**	**0.229**	**5.247**	**2.968**
Aporcelaimellus	Tarsonemus	**0.548**	**-0.701**	**5.247**	**3.570**
Dorylaimoidea	Mylonchulus	**-0.604**	**-0.005**	**5.247**	**4.548**
Dorylaimoidea	Alliphis	**-0.604**	**0.053**	**5.247**	**2.968**
Dorylaimoidea	Aporcelaimellus	**-0.604**	**0.548**	**5.247**	**5.247**
Dorylaimoidea	Dorylaimoidea	**-0.604**	**-0.604**	**5.247**	**5.247**
Dorylaimoidea	Qudsianematidae	**-0.604**	**-0.207**	**5.247**	**4.548**
Dorylaimoidea	Thornematidae	**-0.604**	**-0.470**	**5.247**	**4.548**
Dorylaimoidea	Eupodes	**-0.604**	**0.005**	**5.247**	**4.009**
Dorylaimoidea	Scutacarus	**-0.604**	**-0.608**	**5.247**	**3.445**
Dorylaimoidea	Stigmaeidae	**-0.604**	**0.229**	**5.247**	**2.968**
Dorylaimoidea	Tarsonemus	**-0.604**	**-0.701**	**5.247**	**3.570**
Qudsianematidae	Mylonchulus	**-0.207**	**-0.005**	**4.548**	**4.548**
Qudsianematidae	Alliphis	**-0.207**	**0.053**	**4.548**	**2.968**
Qudsianematidae	Aporcelaimellus	**-0.207**	**0.548**	**4.548**	**5.247**
Qudsianematidae	Dorylaimoidea	**-0.207**	**-0.604**	**4.548**	**5.247**
Qudsianematidae	Qudsianematidae	**-0.207**	**-0.207**	**4.548**	**4.548**
Qudsianematidae	Thornematidae	**-0.207**	**-0.470**	**4.548**	**4.548**
Qudsianematidae	Eupodes	**-0.207**	**0.005**	**4.548**	**4.009**
Qudsianematidae	Scutacarus	**-0.207**	**-0.608**	**4.548**	**3.445**
Qudsianematidae	Stigmaeidae	**-0.207**	**0.229**	**4.548**	**2.968**
Qudsianematidae	Tarsonemus	**-0.207**	**-0.701**	**4.548**	**3.570**
Thornematidae	Mylonchulus	**-0.470**	**-0.005**	**4.548**	**4.548**
Thornematidae	Alliphis	**-0.470**	**0.053**	**4.548**	**2.968**
Thornematidae	Aporcelaimellus	**-0.470**	**0.548**	**4.548**	**5.247**
Thornematidae	Dorylaimoidea	**-0.470**	**-0.604**	**4.548**	**5.247**
Thornematidae	Qudsianematidae	**-0.470**	**-0.207**	**4.548**	**4.548**
Thornematidae	Thornematidae	**-0.470**	**-0.470**	**4.548**	**4.548**
Thornematidae	Eupodes	**-0.470**	**0.005**	**4.548**	**4.009**
Thornematidae	Scutacarus	**-0.470**	**-0.608**	**4.548**	**3.445**
Thornematidae	Stigmaeidae	**-0.470**	**0.229**	**4.548**	**2.968**
Thornematidae	Tarsonemus	**-0.470**	**-0.701**	**4.548**	**3.570**
Eupodes	Aporcelaimellus	**0.005**	**0.548**	**4.009**	**5.247**

Eupodes	Dorylaimoidea	**0.005**	**-0.604**	**4.009**	**5.247**
Eupodes	Qudsianematidae	**0.005**	**-0.207**	**4.009**	**4.548**
Eupodes	Thornematidae	**0.005**	**-0.470**	**4.009**	**4.548**
Eupodes	Eupodes	**0.005**	**0.005**	**4.009**	**4.009**
Eupodes	Scutacarus	**0.005**	**-0.608**	**4.009**	**3.445**
Eupodes	Stigmaeidae	**0.005**	**0.229**	**4.009**	**2.968**
Eupodes	Tarsonemus	**0.005**	**-0.701**	**4.009**	**3.570**
Scutacarus	Aporcelaimellus	**-0.608**	**0.548**	**3.445**	**5.247**
Scutacarus	Dorylaimoidea	**-0.608**	**-0.604**	**3.445**	**5.247**
Scutacarus	Qudsianematidae	**-0.608**	**-0.207**	**3.445**	**4.548**
Scutacarus	Thornematidae	**-0.608**	**-0.470**	**3.445**	**4.548**
Scutacarus	Eupodes	**-0.608**	**0.005**	**3.445**	**4.009**
Scutacarus	Scutacarus	**-0.608**	**-0.608**	**3.445**	**3.445**
Scutacarus	Stigmaeidae	**-0.608**	**0.229**	**3.445**	**2.968**
Scutacarus	Tarsonemus	**-0.608**	**-0.701**	**3.445**	**3.570**
Stigmaeidae	Aporcelaimellus	**0.229**	**0.548**	**2.968**	**5.247**
Stigmaeidae	Dorylaimoidea	**0.229**	**-0.604**	**2.968**	**5.247**
Stigmaeidae	Qudsianematidae	**0.229**	**-0.207**	**2.968**	**4.548**
Stigmaeidae	Thornematidae	**0.229**	**-0.470**	**2.968**	**4.548**
Stigmaeidae	Eupodes	**0.229**	**0.005**	**2.968**	**4.009**
Stigmaeidae	Scutacarus	**0.229**	**-0.608**	**2.968**	**3.445**
Stigmaeidae	Stigmaeidae	**0.229**	**0.229**	**2.968**	**2.968**
Stigmaeidae	Tarsonemus	**0.229**	**-0.701**	**2.968**	**3.570**
Tarsonemus	Aporcelaimellus	**-0.701**	**0.548**	**3.570**	**5.247**
Tarsonemus	Dorylaimoidea	**-0.701**	**-0.604**	**3.570**	**5.247**
Tarsonemus	Qudsianematidae	**-0.701**	**-0.207**	**3.570**	**4.548**
Tarsonemus	Thornematidae	**-0.701**	**-0.470**	**3.570**	**4.548**
Tarsonemus	Eupodes	**-0.701**	**0.005**	**3.570**	**4.009**
Tarsonemus	Scutacarus	**-0.701**	**-0.608**	**3.570**	**3.445**
Tarsonemus	Stigmaeidae	**-0.701**	**0.229**	**3.570**	**2.968**
Tarsonemus	Tarsonemus	**-0.701**	**-0.701**	**3.570**	**3.570**

Resource	Consumer	Mres	Mconsumer	Nres	Nconsumer
Aglenchus	Anatonchus	**-1.053**	**0.406**	**5.199**	**4.597**
Aglenchus	Clarkus	**-1.053**	**-0.310**	**5.199**	**4.597**
Aglenchus	Mylonchulus	**-1.053**	**-0.005**	**5.199**	**4.597**
Aglenchus	Tripyla	**-1.053**	**-0.420**	**5.199**	**5.199**
Aglenchus	Dendrolaelaps	**-1.053**	**0.027**	**5.199**	**2.775**
Aglenchus	Aporcelaimellus	**-1.053**	**0.548**	**5.199**	**5.199**
Aglenchus	Dorylaimoidea	**-1.053**	**-0.604**	**5.199**	**5.500**
Aglenchus	Epidorylaimus	**-1.053**	**0.199**	**5.199**	**4.597**
Aglenchus	Eudorylaimus	**-1.053**	**-0.166**	**5.199**	**5.296**
Aglenchus	Mesodorylaimus	**-1.053**	**-0.277**	**5.199**	**4.597**
Aglenchus	Nordiidae	**-1.053**	**-0.765**	**5.199**	**4.898**
Aglenchus	Pungentus	**-1.053**	**0.263**	**5.199**	**4.898**
Aglenchus	Qudsianematidae	**-1.053**	**-0.207**	**5.199**	**4.898**
Aglenchus	Thornematidae	**-1.053**	**-0.470**	**5.199**	**5.199**
Aglenchus	Eupodes	**-1.053**	**0.005**	**5.199**	**3.474**
Aglenchus	Mesostigmata	**-1.053**	**-0.411**	**5.199**	**3.076**
Aglenchus	Oribatida	**-1.053**	**-0.411**	**5.199**	**2.775**
Aglenchus	Scheloribates	**-1.053**	**0.202**	**5.199**	**3.474**
Aglenchus	Scutacarus	**-1.053**	**-0.608**	**5.199**	**2.775**
Dolichodoridae	Anatonchus	**-0.885**	**0.406**	**5.074**	**4.597**
Dolichodoridae	Clarkus	**-0.885**	**-0.310**	**5.074**	**4.597**
Dolichodoridae	Mylonchulus	**-0.885**	**-0.005**	**5.074**	**4.597**
Dolichodoridae	Tripyla	**-0.885**	**-0.420**	**5.074**	**5.199**
Dolichodoridae	Dendrolaelaps	**-0.885**	**0.027**	**5.074**	**2.775**
Dolichodoridae	Aporcelaimellus	**-0.885**	**0.548**	**5.074**	**5.199**
Dolichodoridae	Dorylaimoidea	**-0.885**	**-0.604**	**5.074**	**5.500**
Dolichodoridae	Epidorylaimus	**-0.885**	**0.199**	**5.074**	**4.597**
Dolichodoridae	Eudorylaimus	**-0.885**	**-0.166**	**5.074**	**5.296**
Dolichodoridae	Mesodorylaimus	**-0.885**	**-0.277**	**5.074**	**4.597**
Dolichodoridae	Nordiidae	**-0.885**	**-0.765**	**5.074**	**4.898**
Dolichodoridae	Pungentus	**-0.885**	**0.263**	**5.074**	**4.898**
Dolichodoridae	Qudsianematidae	**-0.885**	**-0.207**	**5.074**	**4.898**
Dolichodoridae	Thornematidae	**-0.885**	**-0.470**	**5.074**	**5.199**
Dolichodoridae	Eupodes	**-0.885**	**0.005**	**5.074**	**3.474**
Dolichodoridae	Mesostigmata	**-0.885**	**-0.411**	**5.074**	**3.076**
Dolichodoridae	Oribatida	**-0.885**	**-0.411**	**5.074**	**2.775**
Dolichodoridae	Scheloribates	**-0.885**	**0.202**	**5.074**	**3.474**
Dolichodoridae	Scutacarus	**-0.885**	**-0.608**	**5.074**	**2.775**
Helicotylenchus	Anatonchus	**-0.792**	**0.406**	**5.898**	**4.597**
Helicotylenchus	Clarkus	**-0.792**	**-0.310**	**5.898**	**4.597**
Helicotylenchus	Mylonchulus	**-0.792**	**-0.005**	**5.898**	**4.597**
Helicotylenchus	Tripyla	**-0.792**	**-0.420**	**5.898**	**5.199**
Helicotylenchus	Dendrolaelaps	**-0.792**	**0.027**	**5.898**	**2.775**
Helicotylenchus	Aporcelaimellus	**-0.792**	**0.548**	**5.898**	**5.199**
Helicotylenchus	Dorylaimoidea	**-0.792**	**-0.604**	**5.898**	**5.500**
Helicotylenchus	Epidorylaimus	**-0.792**	**0.199**	**5.898**	**4.597**
Helicotylenchus	Eudorylaimus	**-0.792**	**-0.166**	**5.898**	**5.296**
Helicotylenchus	Mesodorylaimus	**-0.792**	**-0.277**	**5.898**	**4.597**
Helicotylenchus	Nordiidae	**-0.792**	**-0.765**	**5.898**	**4.898**
Helicotylenchus	Pungentus	**-0.792**	**0.263**	**5.898**	**4.898**

Helicotylenchus	Qudsianematidae	**-0.792**	**-0.207**	**5.898**	**4.898**
Helicotylenchus	Thornematidae	**-0.792**	**-0.470**	**5.898**	**5.199**
Helicotylenchus	Eupodes	**-0.792**	**0.005**	**5.898**	**3.474**
Helicotylenchus	Mesostigmata	**-0.792**	**-0.411**	**5.898**	**3.076**
Helicotylenchus	Oribatida	**-0.792**	**-0.411**	**5.898**	**2.775**
Helicotylenchus	Scheloribates	**-0.792**	**0.202**	**5.898**	**3.474**
Helicotylenchus	Scutacarus	**-0.792**	**-0.608**	**5.898**	**2.775**
Malenchus	Anatonchus	**-1.330**	**0.406**	**4.597**	**4.597**
Malenchus	Clarkus	**-1.330**	**-0.310**	**4.597**	**4.597**
Malenchus	Mylonchulus	**-1.330**	**-0.005**	**4.597**	**4.597**
Malenchus	Tripyla	**-1.330**	**-0.420**	**4.597**	**5.199**
Malenchus	Dendrolaelaps	**-1.330**	**0.027**	**4.597**	**2.775**
Malenchus	Aporcelaimellus	**-1.330**	**0.548**	**4.597**	**5.199**
Malenchus	Dorylaimoidea	**-1.330**	**-0.604**	**4.597**	**5.500**
Malenchus	Epidorylaimus	**-1.330**	**0.199**	**4.597**	**4.597**
Malenchus	Eudorylaimus	**-1.330**	**-0.166**	**4.597**	**5.296**
Malenchus	Mesodorylaimus	**-1.330**	**-0.277**	**4.597**	**4.597**
Malenchus	Nordiidae	**-1.330**	**-0.765**	**4.597**	**4.898**
Malenchus	Pungentus	**-1.330**	**0.263**	**4.597**	**4.898**
Malenchus	Qudsianematidae	**-1.330**	**-0.207**	**4.597**	**4.898**
Malenchus	Thornematidae	**-1.330**	**-0.470**	**4.597**	**5.199**
Malenchus	Eupodes	**-1.330**	**0.005**	**4.597**	**3.474**
Malenchus	Mesostigmata	**-1.330**	**-0.411**	**4.597**	**3.076**
Malenchus	Oribatida	**-1.330**	**-0.411**	**4.597**	**2.775**
Malenchus	Scheloribates	**-1.330**	**0.202**	**4.597**	**3.474**
Malenchus	Scutacarus	**-1.330**	**-0.608**	**4.597**	**2.775**
Pratylenchus	Anatonchus	**-1.226**	**0.406**	**5.199**	**4.597**
Pratylenchus	Clarkus	**-1.226**	**-0.310**	**5.199**	**4.597**
Pratylenchus	Mylonchulus	**-1.226**	**-0.005**	**5.199**	**4.597**
Pratylenchus	Tripyla	**-1.226**	**-0.420**	**5.199**	**5.199**
Pratylenchus	Dendrolaelaps	**-1.226**	**0.027**	**5.199**	**2.775**
Pratylenchus	Aporcelaimellus	**-1.226**	**0.548**	**5.199**	**5.199**
Pratylenchus	Dorylaimoidea	**-1.226**	**-0.604**	**5.199**	**5.500**
Pratylenchus	Epidorylaimus	**-1.226**	**0.199**	**5.199**	**4.597**
Pratylenchus	Eudorylaimus	**-1.226**	**-0.166**	**5.199**	**5.296**
Pratylenchus	Mesodorylaimus	**-1.226**	**-0.277**	**5.199**	**4.597**
Pratylenchus	Nordiidae	**-1.226**	**-0.765**	**5.199**	**4.898**
Pratylenchus	Pungentus	**-1.226**	**0.263**	**5.199**	**4.898**
Pratylenchus	Qudsianematidae	**-1.226**	**-0.207**	**5.199**	**4.898**
Pratylenchus	Thornematidae	**-1.226**	**-0.470**	**5.199**	**5.199**
Pratylenchus	Eupodes	**-1.226**	**0.005**	**5.199**	**3.474**
Pratylenchus	Mesostigmata	**-1.226**	**-0.411**	**5.199**	**3.076**
Pratylenchus	Oribatida	**-1.226**	**-0.411**	**5.199**	**2.775**
Pratylenchus	Scheloribates	**-1.226**	**0.202**	**5.199**	**3.474**
Pratylenchus	Scutacarus	**-1.226**	**-0.608**	**5.199**	**2.775**
Tylenchorhynchus	Anatonchus	**-0.664**	**0.406**	**5.296**	**4.597**
Tylenchorhynchus	Clarkus	**-0.664**	**-0.310**	**5.296**	**4.597**
Tylenchorhynchus	Mylonchulus	**-0.664**	**-0.005**	**5.296**	**4.597**
Tylenchorhynchus	Tripyla	**-0.664**	**-0.420**	**5.296**	**5.199**
Tylenchorhynchus	Dendrolaelaps	**-0.664**	**0.027**	**5.296**	**2.775**
Tylenchorhynchus	Aporcelaimellus	**-0.664**	**0.548**	**5.296**	**5.199**

Tylenchorhynchus	Dorylaimoidea	**-0.664**	**-0.604**	**5.296**	**5.500**
Tylenchorhynchus	Epidorylaimus	**-0.664**	**0.199**	**5.296**	**4.597**
Tylenchorhynchus	Eudorylaimus	**-0.664**	**-0.166**	**5.296**	**5.296**
Tylenchorhynchus	Mesodorylaimus	**-0.664**	**-0.277**	**5.296**	**4.597**
Tylenchorhynchus	Nordiidae	**-0.664**	**-0.765**	**5.296**	**4.898**
Tylenchorhynchus	Pungentus	**-0.664**	**0.263**	**5.296**	**4.898**
Tylenchorhynchus	Qudsianematidae	**-0.664**	**-0.207**	**5.296**	**4.898**
Tylenchorhynchus	Thornematidae	**-0.664**	**-0.470**	**5.296**	**5.199**
Tylenchorhynchus	Eupodes	**-0.664**	**0.005**	**5.296**	**3.474**
Tylenchorhynchus	Mesostigmata	**-0.664**	**-0.411**	**5.296**	**3.076**
Tylenchorhynchus	Oribatida	**-0.664**	**-0.411**	**5.296**	**2.775**
Tylenchorhynchus	Scheloribates	**-0.664**	**0.202**	**5.296**	**3.474**
Tylenchorhynchus	Scutacarus	**-0.664**	**-0.608**	**5.296**	**2.775**
Achipteria	Dendrolaelaps	**0.341**	**0.027**	**3.076**	**2.775**
Achipteria	Aporcelaimellus	**0.341**	**0.548**	**3.076**	**5.199**
Achipteria	Dorylaimoidea	**0.341**	**-0.604**	**3.076**	**5.500**
Achipteria	Epidorylaimus	**0.341**	**0.199**	**3.076**	**4.597**
Achipteria	Eudorylaimus	**0.341**	**-0.166**	**3.076**	**5.296**
Achipteria	Mesodorylaimus	**0.341**	**-0.277**	**3.076**	**4.597**
Achipteria	Nordiidae	**0.341**	**-0.765**	**3.076**	**4.898**
Achipteria	Pungentus	**0.341**	**0.263**	**3.076**	**4.898**
Achipteria	Qudsianematidae	**0.341**	**-0.207**	**3.076**	**4.898**
Achipteria	Thornematidae	**0.341**	**-0.470**	**3.076**	**5.199**
Achipteria	Eupodes	**0.341**	**0.005**	**3.076**	**3.474**
Achipteria	Mesostigmata	**0.341**	**-0.411**	**3.076**	**3.076**
Achipteria	Oribatida	**0.341**	**-0.411**	**3.076**	**2.775**
Achipteria	Scheloribates	**0.341**	**0.202**	**3.076**	**3.474**
Achipteria	Scutacarus	**0.341**	**-0.608**	**3.076**	**2.775**
Galumna	Dendrolaelaps	**0.474**	**0.027**	**3.076**	**2.775**
Galumna	Aporcelaimellus	**0.474**	**0.548**	**3.076**	**5.199**
Galumna	Dorylaimoidea	**0.474**	**-0.604**	**3.076**	**5.500**
Galumna	Epidorylaimus	**0.474**	**0.199**	**3.076**	**4.597**
Galumna	Eudorylaimus	**0.474**	**-0.166**	**3.076**	**5.296**
Galumna	Mesodorylaimus	**0.474**	**-0.277**	**3.076**	**4.597**
Galumna	Nordiidae	**0.474**	**-0.765**	**3.076**	**4.898**
Galumna	Pungentus	**0.474**	**0.263**	**3.076**	**4.898**
Galumna	Qudsianematidae	**0.474**	**-0.207**	**3.076**	**4.898**
Galumna	Thornematidae	**0.474**	**-0.470**	**3.076**	**5.199**
Galumna	Eupodes	**0.474**	**0.005**	**3.076**	**3.474**
Galumna	Mesostigmata	**0.474**	**-0.411**	**3.076**	**3.076**
Galumna	Oribatida	**0.474**	**-0.411**	**3.076**	**2.775**
Galumna	Scheloribates	**0.474**	**0.202**	**3.076**	**3.474**
Galumna	Scutacarus	**0.474**	**-0.608**	**3.076**	**2.775**
Platynothrus	Dendrolaelaps	**0.710**	**0.027**	**3.076**	**2.775**
Platynothrus	Aporcelaimellus	**0.710**	**0.548**	**3.076**	**5.199**
Platynothrus	Dorylaimoidea	**0.710**	**-0.604**	**3.076**	**5.500**
Platynothrus	Epidorylaimus	**0.710**	**0.199**	**3.076**	**4.597**
Platynothrus	Eudorylaimus	**0.710**	**-0.166**	**3.076**	**5.296**
Platynothrus	Mesodorylaimus	**0.710**	**-0.277**	**3.076**	**4.597**
Platynothrus	Nordiidae	**0.710**	**-0.765**	**3.076**	**4.898**
Platynothrus	Pungentus	**0.710**	**0.263**	**3.076**	**4.898**

Platynothrus	Qudsianematidae	**0.710**	**-0.207**	**3.076**	**4.898**
Platynothrus	Thornematidae	**0.710**	**-0.470**	**3.076**	**5.199**
Platynothrus	Eupodes	**0.710**	**0.005**	**3.076**	**3.474**
Platynothrus	Mesostigmata	**0.710**	**-0.411**	**3.076**	**3.076**
Platynothrus	Oribatida	**0.710**	**-0.411**	**3.076**	**2.775**
Platynothrus	Scheloribates	**0.710**	**0.202**	**3.076**	**3.474**
Platynothrus	Scutacarus	**0.710**	**-0.608**	**3.076**	**2.775**
Sminthuridae	Dendrolaelaps	**-0.608**	**0.027**	**3.377**	**2.775**
Sminthuridae	Aporcelaimellus	**-0.608**	**0.548**	**3.377**	**5.199**
Sminthuridae	Dorylaimoidea	**-0.608**	**-0.604**	**3.377**	**5.500**
Sminthuridae	Epidorylaimus	**-0.608**	**0.199**	**3.377**	**4.597**
Sminthuridae	Eudorylaimus	**-0.608**	**-0.166**	**3.377**	**5.296**
Sminthuridae	Mesodorylaimus	**-0.608**	**-0.277**	**3.377**	**4.597**
Sminthuridae	Nordiidae	**-0.608**	**-0.765**	**3.377**	**4.898**
Sminthuridae	Pungentus	**-0.608**	**0.263**	**3.377**	**4.898**
Sminthuridae	Qudsianematidae	**-0.608**	**-0.207**	**3.377**	**4.898**
Sminthuridae	Thornematidae	**-0.608**	**-0.470**	**3.377**	**5.199**
Sminthuridae	Eupodes	**-0.608**	**0.005**	**3.377**	**3.474**
Sminthuridae	Mesostigmata	**-0.608**	**-0.411**	**3.377**	**3.076**
Sminthuridae	Oribatida	**-0.608**	**-0.411**	**3.377**	**2.775**
Sminthuridae	Scheloribates	**-0.608**	**0.202**	**3.377**	**3.474**
Sminthuridae	Scutacarus	**-0.608**	**-0.608**	**3.377**	**2.775**
Sminthurinus	Dendrolaelaps	**0.618**	**0.027**	**2.775**	**2.775**
Sminthurinus	Aporcelaimellus	**0.618**	**0.548**	**2.775**	**5.199**
Sminthurinus	Dorylaimoidea	**0.618**	**-0.604**	**2.775**	**5.500**
Sminthurinus	Epidorylaimus	**0.618**	**0.199**	**2.775**	**4.597**
Sminthurinus	Eudorylaimus	**0.618**	**-0.166**	**2.775**	**5.296**
Sminthurinus	Mesodorylaimus	**0.618**	**-0.277**	**2.775**	**4.597**
Sminthurinus	Nordiidae	**0.618**	**-0.765**	**2.775**	**4.898**
Sminthurinus	Pungentus	**0.618**	**0.263**	**2.775**	**4.898**
Sminthurinus	Qudsianematidae	**0.618**	**-0.207**	**2.775**	**4.898**
Sminthurinus	Thornematidae	**0.618**	**-0.470**	**2.775**	**5.199**
Sminthurinus	Eupodes	**0.618**	**0.005**	**2.775**	**3.474**
Sminthurinus	Mesostigmata	**0.618**	**-0.411**	**2.775**	**3.076**
Sminthurinus	Oribatida	**0.618**	**-0.411**	**2.775**	**2.775**
Sminthurinus	Scheloribates	**0.618**	**0.202**	**2.775**	**3.474**
Sminthurinus	Scutacarus	**0.618**	**-0.608**	**2.775**	**2.775**
Sminthurus	Dendrolaelaps	**1.429**	**0.027**	**3.076**	**2.775**
Sminthurus	Aporcelaimellus	**1.429**	**0.548**	**3.076**	**5.199**
Sminthurus	Dorylaimoidea	**1.429**	**-0.604**	**3.076**	**5.500**
Sminthurus	Epidorylaimus	**1.429**	**0.199**	**3.076**	**4.597**
Sminthurus	Eudorylaimus	**1.429**	**-0.166**	**3.076**	**5.296**
Sminthurus	Mesodorylaimus	**1.429**	**-0.277**	**3.076**	**4.597**
Sminthurus	Nordiidae	**1.429**	**-0.765**	**3.076**	**4.898**
Sminthurus	Pungentus	**1.429**	**0.263**	**3.076**	**4.898**
Sminthurus	Qudsianematidae	**1.429**	**-0.207**	**3.076**	**4.898**
Sminthurus	Thornematidae	**1.429**	**-0.470**	**3.076**	**5.199**
Sminthurus	Eupodes	**1.429**	**0.005**	**3.076**	**3.474**
Sminthurus	Mesostigmata	**1.429**	**-0.411**	**3.076**	**3.076**
Sminthurus	Oribatida	**1.429**	**-0.411**	**3.076**	**2.775**
Sminthurus	Scheloribates	**1.429**	**0.202**	**3.076**	**3.474**

Sminthurus	Scutacarus	**1.429**	**-0.608**	**3.076**	**2.775**
Sphaeridia	Dendrolaelaps	**0.202**	**0.027**	**3.252**	**2.775**
Sphaeridia	Aporcelaimellus	**0.202**	**0.548**	**3.252**	**5.199**
Sphaeridia	Dorylaimoidea	**0.202**	**-0.604**	**3.252**	**5.500**
Sphaeridia	Epidorylaimus	**0.202**	**0.199**	**3.252**	**4.597**
Sphaeridia	Eudorylaimus	**0.202**	**-0.166**	**3.252**	**5.296**
Sphaeridia	Mesodorylaimus	**0.202**	**-0.277**	**3.252**	**4.597**
Sphaeridia	Nordiidae	**0.202**	**-0.765**	**3.252**	**4.898**
Sphaeridia	Pungentus	**0.202**	**0.263**	**3.252**	**4.898**
Sphaeridia	Qudsianematidae	**0.202**	**-0.207**	**3.252**	**4.898**
Sphaeridia	Thornematidae	**0.202**	**-0.470**	**3.252**	**5.199**
Sphaeridia	Eupodes	**0.202**	**0.005**	**3.252**	**3.474**
Sphaeridia	Mesostigmata	**0.202**	**-0.411**	**3.252**	**3.076**
Sphaeridia	Oribatida	**0.202**	**-0.411**	**3.252**	**2.775**
Sphaeridia	Scheloribates	**0.202**	**0.202**	**3.252**	**3.474**
Sphaeridia	Scutacarus	**0.202**	**-0.608**	**3.252**	**2.775**
Achromadora	Anatonchus	**-1.396**	**0.406**	**4.597**	**4.597**
Achromadora	Clarkus	**-1.396**	**-0.310**	**4.597**	**4.597**
Achromadora	Mylonchulus	**-1.396**	**-0.005**	**4.597**	**4.597**
Achromadora	Tripyla	**-1.396**	**-0.420**	**4.597**	**5.199**
Achromadora	Dendrolaelaps	**-1.396**	**0.027**	**4.597**	**2.775**
Achromadora	Aporcelaimellus	**-1.396**	**0.548**	**4.597**	**5.199**
Achromadora	Dorylaimoidea	**-1.396**	**-0.604**	**4.597**	**5.500**
Achromadora	Epidorylaimus	**-1.396**	**0.199**	**4.597**	**4.597**
Achromadora	Eudorylaimus	**-1.396**	**-0.166**	**4.597**	**5.296**
Achromadora	Mesodorylaimus	**-1.396**	**-0.277**	**4.597**	**4.597**
Achromadora	Nordiidae	**-1.396**	**-0.765**	**4.597**	**4.898**
Achromadora	Pungentus	**-1.396**	**0.263**	**4.597**	**4.898**
Achromadora	Qudsianematidae	**-1.396**	**-0.207**	**4.597**	**4.898**
Achromadora	Thornematidae	**-1.396**	**-0.470**	**4.597**	**5.199**
Achromadora	Eupodes	**-1.396**	**0.005**	**4.597**	**3.474**
Achromadora	Mesostigmata	**-1.396**	**-0.411**	**4.597**	**3.076**
Achromadora	Oribatida	**-1.396**	**-0.411**	**4.597**	**2.775**
Achromadora	Scheloribates	**-1.396**	**0.202**	**4.597**	**3.474**
Achromadora	Scutacarus	**-1.396**	**-0.608**	**4.597**	**2.775**
Aphelenchoides	Anatonchus	**-1.496**	**0.406**	**4.898**	**4.597**
Aphelenchoides	Clarkus	**-1.496**	**-0.310**	**4.898**	**4.597**
Aphelenchoides	Mylonchulus	**-1.496**	**-0.005**	**4.898**	**4.597**
Aphelenchoides	Tripyla	**-1.496**	**-0.420**	**4.898**	**5.199**
Aphelenchoides	Dendrolaelaps	**-1.496**	**0.027**	**4.898**	**2.775**
Aphelenchoides	Aporcelaimellus	**-1.496**	**0.548**	**4.898**	**5.199**
Aphelenchoides	Dorylaimoidea	**-1.496**	**-0.604**	**4.898**	**5.500**
Aphelenchoides	Epidorylaimus	**-1.496**	**0.199**	**4.898**	**4.597**
Aphelenchoides	Eudorylaimus	**-1.496**	**-0.166**	**4.898**	**5.296**
Aphelenchoides	Mesodorylaimus	**-1.496**	**-0.277**	**4.898**	**4.597**
Aphelenchoides	Nordiidae	**-1.496**	**-0.765**	**4.898**	**4.898**
Aphelenchoides	Pungentus	**-1.496**	**0.263**	**4.898**	**4.898**
Aphelenchoides	Qudsianematidae	**-1.496**	**-0.207**	**4.898**	**4.898**
Aphelenchoides	Thornematidae	**-1.496**	**-0.470**	**4.898**	**5.199**
Aphelenchoides	Eupodes	**-1.496**	**0.005**	**4.898**	**3.474**
Aphelenchoides	Mesostigmata	**-1.496**	**-0.411**	**4.898**	**3.076**

Aphelenchoides	Oribatida	**-1.496**	**-0.411**	**4.898**	**2.775**
Aphelenchoides	Scheloribates	**-1.496**	**0.202**	**4.898**	**3.474**
Aphelenchoides	Scutacarus	**-1.496**	**-0.608**	**4.898**	**2.775**
Tylenchidae	Anatonchus	**-1.360**	**0.406**	**6.029**	**4.597**
Tylenchidae	Clarkus	**-1.360**	**-0.310**	**6.029**	**4.597**
Tylenchidae	Mylonchulus	**-1.360**	**-0.005**	**6.029**	**4.597**
Tylenchidae	Tripyla	**-1.360**	**-0.420**	**6.029**	**5.199**
Tylenchidae	Dendrolaelaps	**-1.360**	**0.027**	**6.029**	**2.775**
Tylenchidae	Aporcelaimellus	**-1.360**	**0.548**	**6.029**	**5.199**
Tylenchidae	Dorylaimoidea	**-1.360**	**-0.604**	**6.029**	**5.500**
Tylenchidae	Epidorylaimus	**-1.360**	**0.199**	**6.029**	**4.597**
Tylenchidae	Eudorylaimus	**-1.360**	**-0.166**	**6.029**	**5.296**
Tylenchidae	Mesodorylaimus	**-1.360**	**-0.277**	**6.029**	**4.597**
Tylenchidae	Nordiidae	**-1.360**	**-0.765**	**6.029**	**4.898**
Tylenchidae	Pungentus	**-1.360**	**0.263**	**6.029**	**4.898**
Tylenchidae	Qudsianematidae	**-1.360**	**-0.207**	**6.029**	**4.898**
Tylenchidae	Thornematidae	**-1.360**	**-0.470**	**6.029**	**5.199**
Tylenchidae	Eupodes	**-1.360**	**0.005**	**6.029**	**3.474**
Tylenchidae	Mesostigmata	**-1.360**	**-0.411**	**6.029**	**3.076**
Tylenchidae	Oribatida	**-1.360**	**-0.411**	**6.029**	**2.775**
Tylenchidae	Scheloribates	**-1.360**	**0.202**	**6.029**	**3.474**
Tylenchidae	Scutacarus	**-1.360**	**-0.608**	**6.029**	**2.775**
Medioppia	Dendrolaelaps	**-0.235**	**0.027**	**3.076**	**2.775**
Medioppia	Aporcelaimellus	**-0.235**	**0.548**	**3.076**	**5.199**
Medioppia	Dorylaimoidea	**-0.235**	**-0.604**	**3.076**	**5.500**
Medioppia	Epidorylaimus	**-0.235**	**0.199**	**3.076**	**4.597**
Medioppia	Eudorylaimus	**-0.235**	**-0.166**	**3.076**	**5.296**
Medioppia	Mesodorylaimus	**-0.235**	**-0.277**	**3.076**	**4.597**
Medioppia	Nordiidae	**-0.235**	**-0.765**	**3.076**	**4.898**
Medioppia	Pungentus	**-0.235**	**0.263**	**3.076**	**4.898**
Medioppia	Qudsianematidae	**-0.235**	**-0.207**	**3.076**	**4.898**
Medioppia	Thornematidae	**-0.235**	**-0.470**	**3.076**	**5.199**
Medioppia	Eupodes	**-0.235**	**0.005**	**3.076**	**3.474**
Medioppia	Mesostigmata	**-0.235**	**-0.411**	**3.076**	**3.076**
Medioppia	Oribatida	**-0.235**	**-0.411**	**3.076**	**2.775**
Medioppia	Scheloribates	**-0.235**	**0.202**	**3.076**	**3.474**
Medioppia	Scutacarus	**-0.235**	**-0.608**	**3.076**	**2.775**
Microppia	Dendrolaelaps	**-0.544**	**0.027**	**2.775**	**2.775**
Microppia	Aporcelaimellus	**-0.544**	**0.548**	**2.775**	**5.199**
Microppia	Dorylaimoidea	**-0.544**	**-0.604**	**2.775**	**5.500**
Microppia	Epidorylaimus	**-0.544**	**0.199**	**2.775**	**4.597**
Microppia	Eudorylaimus	**-0.544**	**-0.166**	**2.775**	**5.296**
Microppia	Mesodorylaimus	**-0.544**	**-0.277**	**2.775**	**4.597**
Microppia	Nordiidae	**-0.544**	**-0.765**	**2.775**	**4.898**
Microppia	Pungentus	**-0.544**	**0.263**	**2.775**	**4.898**
Microppia	Qudsianematidae	**-0.544**	**-0.207**	**2.775**	**4.898**
Microppia	Thornematidae	**-0.544**	**-0.470**	**2.775**	**5.199**
Microppia	Eupodes	**-0.544**	**0.005**	**2.775**	**3.474**
Microppia	Mesostigmata	**-0.544**	**-0.411**	**2.775**	**3.076**
Microppia	Oribatida	**-0.544**	**-0.411**	**2.775**	**2.775**
Microppia	Scheloribates	**-0.544**	**0.202**	**2.775**	**3.474**

Microppia	Scutacarus	**-0.544**	**-0.608**	**2.775**	**2.775**
Minunthozetes	Dendrolaelaps	**-0.249**	**0.027**	**2.775**	**2.775**
Minunthozetes	Aporcelaimellus	**-0.249**	**0.548**	**2.775**	**5.199**
Minunthozetes	Dorylaimoidea	**-0.249**	**-0.604**	**2.775**	**5.500**
Minunthozetes	Epidorylaimus	**-0.249**	**0.199**	**2.775**	**4.597**
Minunthozetes	Eudorylaimus	**-0.249**	**-0.166**	**2.775**	**5.296**
Minunthozetes	Mesodorylaimus	**-0.249**	**-0.277**	**2.775**	**4.597**
Minunthozetes	Nordiidae	**-0.249**	**-0.765**	**2.775**	**4.898**
Minunthozetes	Pungentus	**-0.249**	**0.263**	**2.775**	**4.898**
Minunthozetes	Qudsianematidae	**-0.249**	**-0.207**	**2.775**	**4.898**
Minunthozetes	Thornematidae	**-0.249**	**-0.470**	**2.775**	**5.199**
Minunthozetes	Eupodes	**-0.249**	**0.005**	**2.775**	**3.474**
Minunthozetes	Mesostigmata	**-0.249**	**-0.411**	**2.775**	**3.076**
Minunthozetes	Oribatida	**-0.249**	**-0.411**	**2.775**	**2.775**
Minunthozetes	Scheloribates	**-0.249**	**0.202**	**2.775**	**3.474**
Minunthozetes	Scutacarus	**-0.249**	**-0.608**	**2.775**	**2.775**
Tectocepheus	Dendrolaelaps	**-0.220**	**0.027**	**2.775**	**2.775**
Tectocepheus	Aporcelaimellus	**-0.220**	**0.548**	**2.775**	**5.199**
Tectocepheus	Dorylaimoidea	**-0.220**	**-0.604**	**2.775**	**5.500**
Tectocepheus	Epidorylaimus	**-0.220**	**0.199**	**2.775**	**4.597**
Tectocepheus	Eudorylaimus	**-0.220**	**-0.166**	**2.775**	**5.296**
Tectocepheus	Mesodorylaimus	**-0.220**	**-0.277**	**2.775**	**4.597**
Tectocepheus	Nordiidae	**-0.220**	**-0.765**	**2.775**	**4.898**
Tectocepheus	Pungentus	**-0.220**	**0.263**	**2.775**	**4.898**
Tectocepheus	Qudsianematidae	**-0.220**	**-0.207**	**2.775**	**4.898**
Tectocepheus	Thornematidae	**-0.220**	**-0.470**	**2.775**	**5.199**
Tectocepheus	Eupodes	**-0.220**	**0.005**	**2.775**	**3.474**
Tectocepheus	Mesostigmata	**-0.220**	**-0.411**	**2.775**	**3.076**
Tectocepheus	Oribatida	**-0.220**	**-0.411**	**2.775**	**2.775**
Tectocepheus	Scheloribates	**-0.220**	**0.202**	**2.775**	**3.474**
Tectocepheus	Scutacarus	**-0.220**	**-0.608**	**2.775**	**2.775**
Folsomia	Dendrolaelaps	**0.900**	**0.027**	**2.775**	**2.775**
Folsomia	Aporcelaimellus	**0.900**	**0.548**	**2.775**	**5.199**
Folsomia	Dorylaimoidea	**0.900**	**-0.604**	**2.775**	**5.500**
Folsomia	Epidorylaimus	**0.900**	**0.199**	**2.775**	**4.597**
Folsomia	Eudorylaimus	**0.900**	**-0.166**	**2.775**	**5.296**
Folsomia	Mesodorylaimus	**0.900**	**-0.277**	**2.775**	**4.597**
Folsomia	Nordiidae	**0.900**	**-0.765**	**2.775**	**4.898**
Folsomia	Pungentus	**0.900**	**0.263**	**2.775**	**4.898**
Folsomia	Qudsianematidae	**0.900**	**-0.207**	**2.775**	**4.898**
Folsomia	Thornematidae	**0.900**	**-0.470**	**2.775**	**5.199**
Folsomia	Eupodes	**0.900**	**0.005**	**2.775**	**3.474**
Folsomia	Mesostigmata	**0.900**	**-0.411**	**2.775**	**3.076**
Folsomia	Oribatida	**0.900**	**-0.411**	**2.775**	**2.775**
Folsomia	Scheloribates	**0.900**	**0.202**	**2.775**	**3.474**
Folsomia	Scutacarus	**0.900**	**-0.608**	**2.775**	**2.775**
Friesea	Dendrolaelaps	**0.434**	**0.027**	**3.076**	**2.775**
Friesea	Aporcelaimellus	**0.434**	**0.548**	**3.076**	**5.199**
Friesea	Dorylaimoidea	**0.434**	**-0.604**	**3.076**	**5.500**
Friesea	Epidorylaimus	**0.434**	**0.199**	**3.076**	**4.597**
Friesea	Eudorylaimus	**0.434**	**-0.166**	**3.076**	**5.296**

Friesea	Mesodorylaimus	**0.434**	**-0.277**	**3.076**	**4.597**
Friesea	Nordiidae	**0.434**	**-0.765**	**3.076**	**4.898**
Friesea	Pungentus	**0.434**	**0.263**	**3.076**	**4.898**
Friesea	Qudsianematidae	**0.434**	**-0.207**	**3.076**	**4.898**
Friesea	Thornematidae	**0.434**	**-0.470**	**3.076**	**5.199**
Friesea	Eupodes	**0.434**	**0.005**	**3.076**	**3.474**
Friesea	Mesostigmata	**0.434**	**-0.411**	**3.076**	**3.076**
Friesea	Oribatida	**0.434**	**-0.411**	**3.076**	**2.775**
Friesea	Scheloribates	**0.434**	**0.202**	**3.076**	**3.474**
Friesea	Scutacarus	**0.434**	**-0.608**	**3.076**	**2.775**
Isotoma	Dendrolaelaps	**1.898**	**0.027**	**3.816**	**2.775**
Isotoma	Aporcelaimellus	**1.898**	**0.548**	**3.816**	**5.199**
Isotoma	Dorylaimoidea	**1.898**	**-0.604**	**3.816**	**5.500**
Isotoma	Epidorylaimus	**1.898**	**0.199**	**3.816**	**4.597**
Isotoma	Eudorylaimus	**1.898**	**-0.166**	**3.816**	**5.296**
Isotoma	Mesodorylaimus	**1.898**	**-0.277**	**3.816**	**4.597**
Isotoma	Nordiidae	**1.898**	**-0.765**	**3.816**	**4.898**
Isotoma	Pungentus	**1.898**	**0.263**	**3.816**	**4.898**
Isotoma	Qudsianematidae	**1.898**	**-0.207**	**3.816**	**4.898**
Isotoma	Thornematidae	**1.898**	**-0.470**	**3.816**	**5.199**
Isotoma	Eupodes	**1.898**	**0.005**	**3.816**	**3.474**
Isotoma	Mesostigmata	**1.898**	**-0.411**	**3.816**	**3.076**
Isotoma	Oribatida	**1.898**	**-0.411**	**3.816**	**2.775**
Isotoma	Scheloribates	**1.898**	**0.202**	**3.816**	**3.474**
Isotoma	Scutacarus	**1.898**	**-0.608**	**3.816**	**2.775**
Isotomurus	Dendrolaelaps	**1.787**	**0.027**	**3.252**	**2.775**
Isotomurus	Aporcelaimellus	**1.787**	**0.548**	**3.252**	**5.199**
Isotomurus	Dorylaimoidea	**1.787**	**-0.604**	**3.252**	**5.500**
Isotomurus	Epidorylaimus	**1.787**	**0.199**	**3.252**	**4.597**
Isotomurus	Eudorylaimus	**1.787**	**-0.166**	**3.252**	**5.296**
Isotomurus	Mesodorylaimus	**1.787**	**-0.277**	**3.252**	**4.597**
Isotomurus	Nordiidae	**1.787**	**-0.765**	**3.252**	**4.898**
Isotomurus	Pungentus	**1.787**	**0.263**	**3.252**	**4.898**
Isotomurus	Qudsianematidae	**1.787**	**-0.207**	**3.252**	**4.898**
Isotomurus	Thornematidae	**1.787**	**-0.470**	**3.252**	**5.199**
Isotomurus	Eupodes	**1.787**	**0.005**	**3.252**	**3.474**
Isotomurus	Mesostigmata	**1.787**	**-0.411**	**3.252**	**3.076**
Isotomurus	Oribatida	**1.787**	**-0.411**	**3.252**	**2.775**
Isotomurus	Scheloribates	**1.787**	**0.202**	**3.252**	**3.474**
Isotomurus	Scutacarus	**1.787**	**-0.608**	**3.252**	**2.775**
Lepidocyrtus	Dendrolaelaps	**1.231**	**0.027**	**3.474**	**2.775**
Lepidocyrtus	Aporcelaimellus	**1.231**	**0.548**	**3.474**	**5.199**
Lepidocyrtus	Dorylaimoidea	**1.231**	**-0.604**	**3.474**	**5.500**
Lepidocyrtus	Epidorylaimus	**1.231**	**0.199**	**3.474**	**4.597**
Lepidocyrtus	Eudorylaimus	**1.231**	**-0.166**	**3.474**	**5.296**
Lepidocyrtus	Mesodorylaimus	**1.231**	**-0.277**	**3.474**	**4.597**
Lepidocyrtus	Nordiidae	**1.231**	**-0.765**	**3.474**	**4.898**
Lepidocyrtus	Pungentus	**1.231**	**0.263**	**3.474**	**4.898**
Lepidocyrtus	Qudsianematidae	**1.231**	**-0.207**	**3.474**	**4.898**
Lepidocyrtus	Thornematidae	**1.231**	**-0.470**	**3.474**	**5.199**
Lepidocyrtus	Eupodes	**1.231**	**0.005**	**3.474**	**3.474**

Lepidocyrtus	Mesostigmata	**1.231**	**-0.411**	**3.474**	**3.076**
Lepidocyrtus	Oribatida	**1.231**	**-0.411**	**3.474**	**2.775**
Lepidocyrtus	Scheloribates	**1.231**	**0.202**	**3.474**	**3.474**
Lepidocyrtus	Scutacarus	**1.231**	**-0.608**	**3.474**	**2.775**
Onychiurus	Dendrolaelaps	**0.977**	**0.027**	**2.775**	**2.775**
Onychiurus	Aporcelaimellus	**0.977**	**0.548**	**2.775**	**5.199**
Onychiurus	Dorylaimoidea	**0.977**	**-0.604**	**2.775**	**5.500**
Onychiurus	Epidorylaimus	**0.977**	**0.199**	**2.775**	**4.597**
Onychiurus	Eudorylaimus	**0.977**	**-0.166**	**2.775**	**5.296**
Onychiurus	Mesodorylaimus	**0.977**	**-0.277**	**2.775**	**4.597**
Onychiurus	Nordiidae	**0.977**	**-0.765**	**2.775**	**4.898**
Onychiurus	Pungentus	**0.977**	**0.263**	**2.775**	**4.898**
Onychiurus	Qudsianematidae	**0.977**	**-0.207**	**2.775**	**4.898**
Onychiurus	Thornematidae	**0.977**	**-0.470**	**2.775**	**5.199**
Onychiurus	Eupodes	**0.977**	**0.005**	**2.775**	**3.474**
Onychiurus	Mesostigmata	**0.977**	**-0.411**	**2.775**	**3.076**
Onychiurus	Oribatida	**0.977**	**-0.411**	**2.775**	**2.775**
Onychiurus	Scheloribates	**0.977**	**0.202**	**2.775**	**3.474**
Onychiurus	Scutacarus	**0.977**	**-0.608**	**2.775**	**2.775**
Parisotoma	Dendrolaelaps	**0.722**	**0.027**	**3.678**	**2.775**
Parisotoma	Aporcelaimellus	**0.722**	**0.548**	**3.678**	**5.199**
Parisotoma	Dorylaimoidea	**0.722**	**-0.604**	**3.678**	**5.500**
Parisotoma	Epidorylaimus	**0.722**	**0.199**	**3.678**	**4.597**
Parisotoma	Eudorylaimus	**0.722**	**-0.166**	**3.678**	**5.296**
Parisotoma	Mesodorylaimus	**0.722**	**-0.277**	**3.678**	**4.597**
Parisotoma	Nordiidae	**0.722**	**-0.765**	**3.678**	**4.898**
Parisotoma	Pungentus	**0.722**	**0.263**	**3.678**	**4.898**
Parisotoma	Qudsianematidae	**0.722**	**-0.207**	**3.678**	**4.898**
Parisotoma	Thornematidae	**0.722**	**-0.470**	**3.678**	**5.199**
Parisotoma	Eupodes	**0.722**	**0.005**	**3.678**	**3.474**
Parisotoma	Mesostigmata	**0.722**	**-0.411**	**3.678**	**3.076**
Parisotoma	Oribatida	**0.722**	**-0.411**	**3.678**	**2.775**
Parisotoma	Scheloribates	**0.722**	**0.202**	**3.678**	**3.474**
Parisotoma	Scutacarus	**0.722**	**-0.608**	**3.678**	**2.775**
Proisotoma	Dendrolaelaps	**0.770**	**0.027**	**2.775**	**2.775**
Proisotoma	Aporcelaimellus	**0.770**	**0.548**	**2.775**	**5.199**
Proisotoma	Dorylaimoidea	**0.770**	**-0.604**	**2.775**	**5.500**
Proisotoma	Epidorylaimus	**0.770**	**0.199**	**2.775**	**4.597**
Proisotoma	Eudorylaimus	**0.770**	**-0.166**	**2.775**	**5.296**
Proisotoma	Mesodorylaimus	**0.770**	**-0.277**	**2.775**	**4.597**
Proisotoma	Nordiidae	**0.770**	**-0.765**	**2.775**	**4.898**
Proisotoma	Pungentus	**0.770**	**0.263**	**2.775**	**4.898**
Proisotoma	Qudsianematidae	**0.770**	**-0.207**	**2.775**	**4.898**
Proisotoma	Thornematidae	**0.770**	**-0.470**	**2.775**	**5.199**
Proisotoma	Eupodes	**0.770**	**0.005**	**2.775**	**3.474**
Proisotoma	Mesostigmata	**0.770**	**-0.411**	**2.775**	**3.076**
Proisotoma	Oribatida	**0.770**	**-0.411**	**2.775**	**2.775**
Proisotoma	Scheloribates	**0.770**	**0.202**	**2.775**	**3.474**
Proisotoma	Scutacarus	**0.770**	**-0.608**	**2.775**	**2.775**
Achaeta	Dendrolaelaps	**1.320**	**0.027**	**2.616**	**2.775**
Achaeta	Aporcelaimellus	**1.320**	**0.548**	**2.616**	**5.199**

Achaeta	Dorylaimoidea	**1.320**	**-0.604**	**2.616**	**5.500**
Achaeta	Epidorylaimus	**1.320**	**0.199**	**2.616**	**4.597**
Achaeta	Eudorylaimus	**1.320**	**-0.166**	**2.616**	**5.296**
Achaeta	Mesodorylaimus	**1.320**	**-0.277**	**2.616**	**4.597**
Achaeta	Nordiidae	**1.320**	**-0.765**	**2.616**	**4.898**
Achaeta	Pungentus	**1.320**	**0.263**	**2.616**	**4.898**
Achaeta	Qudsianematidae	**1.320**	**-0.207**	**2.616**	**4.898**
Achaeta	Thornematidae	**1.320**	**-0.470**	**2.616**	**5.199**
Achaeta	Eupodes	**1.320**	**0.005**	**2.616**	**3.474**
Achaeta	Mesostigmata	**1.320**	**-0.411**	**2.616**	**3.076**
Achaeta	Oribatida	**1.320**	**-0.411**	**2.616**	**2.775**
Achaeta	Scheloribates	**1.320**	**0.202**	**2.616**	**3.474**
Achaeta	Scutacarus	**1.320**	**-0.608**	**2.616**	**2.775**
Cognettia	Dendrolaelaps	**1.559**	**0.027**	**3.556**	**2.775**
Cognettia	Aporcelaimellus	**1.559**	**0.548**	**3.556**	**5.199**
Cognettia	Dorylaimoidea	**1.559**	**-0.604**	**3.556**	**5.500**
Cognettia	Epidorylaimus	**1.559**	**0.199**	**3.556**	**4.597**
Cognettia	Eudorylaimus	**1.559**	**-0.166**	**3.556**	**5.296**
Cognettia	Mesodorylaimus	**1.559**	**-0.277**	**3.556**	**4.597**
Cognettia	Nordiidae	**1.559**	**-0.765**	**3.556**	**4.898**
Cognettia	Pungentus	**1.559**	**0.263**	**3.556**	**4.898**
Cognettia	Qudsianematidae	**1.559**	**-0.207**	**3.556**	**4.898**
Cognettia	Thornematidae	**1.559**	**-0.470**	**3.556**	**5.199**
Cognettia	Eupodes	**1.559**	**0.005**	**3.556**	**3.474**
Cognettia	Mesostigmata	**1.559**	**-0.411**	**3.556**	**3.076**
Cognettia	Oribatida	**1.559**	**-0.411**	**3.556**	**2.775**
Cognettia	Scheloribates	**1.559**	**0.202**	**3.556**	**3.474**
Cognettia	Scutacarus	**1.559**	**-0.608**	**3.556**	**2.775**
Fridericia	Dendrolaelaps	**2.453**	**0.027**	**3.351**	**2.775**
Fridericia	Aporcelaimellus	**2.453**	**0.548**	**3.351**	**5.199**
Fridericia	Dorylaimoidea	**2.453**	**-0.604**	**3.351**	**5.500**
Fridericia	Epidorylaimus	**2.453**	**0.199**	**3.351**	**4.597**
Fridericia	Eudorylaimus	**2.453**	**-0.166**	**3.351**	**5.296**
Fridericia	Mesodorylaimus	**2.453**	**-0.277**	**3.351**	**4.597**
Fridericia	Nordiidae	**2.453**	**-0.765**	**3.351**	**4.898**
Fridericia	Pungentus	**2.453**	**0.263**	**3.351**	**4.898**
Fridericia	Qudsianematidae	**2.453**	**-0.207**	**3.351**	**4.898**
Fridericia	Thornematidae	**2.453**	**-0.470**	**3.351**	**5.199**
Fridericia	Eupodes	**2.453**	**0.005**	**3.351**	**3.474**
Fridericia	Mesostigmata	**2.453**	**-0.411**	**3.351**	**3.076**
Fridericia	Oribatida	**2.453**	**-0.411**	**3.351**	**2.775**
Fridericia	Scheloribates	**2.453**	**0.202**	**3.351**	**3.474**
Fridericia	Scutacarus	**2.453**	**-0.608**	**3.351**	**2.775**
Alaimus	Anatonchus	**-0.858**	**0.406**	**4.597**	**4.597**
Alaimus	Clarkus	**-0.858**	**-0.310**	**4.597**	**4.597**
Alaimus	Mylonchulus	**-0.858**	**-0.005**	**4.597**	**4.597**
Alaimus	Tripyla	**-0.858**	**-0.420**	**4.597**	**5.199**
Alaimus	Dendrolaelaps	**-0.858**	**0.027**	**4.597**	**2.775**
Alaimus	Aporcelaimellus	**-0.858**	**0.548**	**4.597**	**5.199**
Alaimus	Dorylaimoidea	**-0.858**	**-0.604**	**4.597**	**5.500**
Alaimus	Epidorylaimus	**-0.858**	**0.199**	**4.597**	**4.597**

Alaimus	Eudorylaimus	**-0.858**	**-0.166**	**4.597**	**5.296**
Alaimus	Mesodorylaimus	**-0.858**	**-0.277**	**4.597**	**4.597**
Alaimus	Nordiidae	**-0.858**	**-0.765**	**4.597**	**4.898**
Alaimus	Pungentus	**-0.858**	**0.263**	**4.597**	**4.898**
Alaimus	Qudsianematidae	**-0.858**	**-0.207**	**4.597**	**4.898**
Alaimus	Thornematidae	**-0.858**	**-0.470**	**4.597**	**5.199**
Alaimus	Eupodes	**-0.858**	**0.005**	**4.597**	**3.474**
Alaimus	Mesostigmata	**-0.858**	**-0.411**	**4.597**	**3.076**
Alaimus	Oribatida	**-0.858**	**-0.411**	**4.597**	**2.775**
Alaimus	Scheloribates	**-0.858**	**0.202**	**4.597**	**3.474**
Alaimus	Scutacarus	**-0.858**	**-0.608**	**4.597**	**2.775**
Anaplectus	Anatonchus	**-0.519**	**0.406**	**5.074**	**4.597**
Anaplectus	Clarkus	**-0.519**	**-0.310**	**5.074**	**4.597**
Anaplectus	Mylonchulus	**-0.519**	**-0.005**	**5.074**	**4.597**
Anaplectus	Tripyla	**-0.519**	**-0.420**	**5.074**	**5.199**
Anaplectus	Dendrolaelaps	**-0.519**	**0.027**	**5.074**	**2.775**
Anaplectus	Aporcelaimellus	**-0.519**	**0.548**	**5.074**	**5.199**
Anaplectus	Dorylaimoidea	**-0.519**	**-0.604**	**5.074**	**5.500**
Anaplectus	Epidorylaimus	**-0.519**	**0.199**	**5.074**	**4.597**
Anaplectus	Eudorylaimus	**-0.519**	**-0.166**	**5.074**	**5.296**
Anaplectus	Mesodorylaimus	**-0.519**	**-0.277**	**5.074**	**4.597**
Anaplectus	Nordiidae	**-0.519**	**-0.765**	**5.074**	**4.898**
Anaplectus	Pungentus	**-0.519**	**0.263**	**5.074**	**4.898**
Anaplectus	Qudsianematidae	**-0.519**	**-0.207**	**5.074**	**4.898**
Anaplectus	Thornematidae	**-0.519**	**-0.470**	**5.074**	**5.199**
Anaplectus	Eupodes	**-0.519**	**0.005**	**5.074**	**3.474**
Anaplectus	Mesostigmata	**-0.519**	**-0.411**	**5.074**	**3.076**
Anaplectus	Oribatida	**-0.519**	**-0.411**	**5.074**	**2.775**
Anaplectus	Scheloribates	**-0.519**	**0.202**	**5.074**	**3.474**
Anaplectus	Scutacarus	**-0.519**	**-0.608**	**5.074**	**2.775**
Eucephalobus	Anatonchus	**-0.855**	**0.406**	**5.801**	**4.597**
Eucephalobus	Clarkus	**-0.855**	**-0.310**	**5.801**	**4.597**
Eucephalobus	Mylonchulus	**-0.855**	**-0.005**	**5.801**	**4.597**
Eucephalobus	Tripyla	**-0.855**	**-0.420**	**5.801**	**5.199**
Eucephalobus	Dendrolaelaps	**-0.855**	**0.027**	**5.801**	**2.775**
Eucephalobus	Aporcelaimellus	**-0.855**	**0.548**	**5.801**	**5.199**
Eucephalobus	Dorylaimoidea	**-0.855**	**-0.604**	**5.801**	**5.500**
Eucephalobus	Epidorylaimus	**-0.855**	**0.199**	**5.801**	**4.597**
Eucephalobus	Eudorylaimus	**-0.855**	**-0.166**	**5.801**	**5.296**
Eucephalobus	Mesodorylaimus	**-0.855**	**-0.277**	**5.801**	**4.597**
Eucephalobus	Nordiidae	**-0.855**	**-0.765**	**5.801**	**4.898**
Eucephalobus	Pungentus	**-0.855**	**0.263**	**5.801**	**4.898**
Eucephalobus	Qudsianematidae	**-0.855**	**-0.207**	**5.801**	**4.898**
Eucephalobus	Thornematidae	**-0.855**	**-0.470**	**5.801**	**5.199**
Eucephalobus	Eupodes	**-0.855**	**0.005**	**5.801**	**3.474**
Eucephalobus	Mesostigmata	**-0.855**	**-0.411**	**5.801**	**3.076**
Eucephalobus	Oribatida	**-0.855**	**-0.411**	**5.801**	**2.775**
Eucephalobus	Scheloribates	**-0.855**	**0.202**	**5.801**	**3.474**
Eucephalobus	Scutacarus	**-0.855**	**-0.608**	**5.801**	**2.775**
Metateratocephalus	Anatonchus	**-1.506**	**0.406**	**5.074**	**4.597**
Metateratocephalus	Clarkus	**-1.506**	**-0.310**	**5.074**	**4.597**

Metateratocephalus	Mylonchulus	**-1.506**	**-0.005**	**5.074**	**4.597**
Metateratocephalus	Tripyla	**-1.506**	**-0.420**	**5.074**	**5.199**
Metateratocephalus	Dendrolaelaps	**-1.506**	**0.027**	**5.074**	**2.775**
Metateratocephalus	Aporcelaimellus	**-1.506**	**0.548**	**5.074**	**5.199**
Metateratocephalus	Dorylaimoidea	**-1.506**	**-0.604**	**5.074**	**5.500**
Metateratocephalus	Epidorylaimus	**-1.506**	**0.199**	**5.074**	**4.597**
Metateratocephalus	Eudorylaimus	**-1.506**	**-0.166**	**5.074**	**5.296**
Metateratocephalus	Mesodorylaimus	**-1.506**	**-0.277**	**5.074**	**4.597**
Metateratocephalus	Nordiidae	**-1.506**	**-0.765**	**5.074**	**4.898**
Metateratocephalus	Pungentus	**-1.506**	**0.263**	**5.074**	**4.898**
Metateratocephalus	Qudsianematidae	**-1.506**	**-0.207**	**5.074**	**4.898**
Metateratocephalus	Thornematidae	**-1.506**	**-0.470**	**5.074**	**5.199**
Metateratocephalus	Eupodes	**-1.506**	**0.005**	**5.074**	**3.474**
Metateratocephalus	Mesostigmata	**-1.506**	**-0.411**	**5.074**	**3.076**
Metateratocephalus	Oribatida	**-1.506**	**-0.411**	**5.074**	**2.775**
Metateratocephalus	Scheloribates	**-1.506**	**0.202**	**5.074**	**3.474**
Metateratocephalus	Scutacarus	**-1.506**	**-0.608**	**5.074**	**2.775**
Panagrolaimus	Anatonchus	**-0.945**	**0.406**	**4.898**	**4.597**
Panagrolaimus	Clarkus	**-0.945**	**-0.310**	**4.898**	**4.597**
Panagrolaimus	Mylonchulus	**-0.945**	**-0.005**	**4.898**	**4.597**
Panagrolaimus	Tripyla	**-0.945**	**-0.420**	**4.898**	**5.199**
Panagrolaimus	Dendrolaelaps	**-0.945**	**0.027**	**4.898**	**2.775**
Panagrolaimus	Aporcelaimellus	**-0.945**	**0.548**	**4.898**	**5.199**
Panagrolaimus	Dorylaimoidea	**-0.945**	**-0.604**	**4.898**	**5.500**
Panagrolaimus	Epidorylaimus	**-0.945**	**0.199**	**4.898**	**4.597**
Panagrolaimus	Eudorylaimus	**-0.945**	**-0.166**	**4.898**	**5.296**
Panagrolaimus	Mesodorylaimus	**-0.945**	**-0.277**	**4.898**	**4.597**
Panagrolaimus	Nordiidae	**-0.945**	**-0.765**	**4.898**	**4.898**
Panagrolaimus	Pungentus	**-0.945**	**0.263**	**4.898**	**4.898**
Panagrolaimus	Qudsianematidae	**-0.945**	**-0.207**	**4.898**	**4.898**
Panagrolaimus	Thornematidae	**-0.945**	**-0.470**	**4.898**	**5.199**
Panagrolaimus	Eupodes	**-0.945**	**0.005**	**4.898**	**3.474**
Panagrolaimus	Mesostigmata	**-0.945**	**-0.411**	**4.898**	**3.076**
Panagrolaimus	Oribatida	**-0.945**	**-0.411**	**4.898**	**2.775**
Panagrolaimus	Scheloribates	**-0.945**	**0.202**	**4.898**	**3.474**
Panagrolaimus	Scutacarus	**-0.945**	**-0.608**	**4.898**	**2.775**
Plectus	Anatonchus	**-0.583**	**0.406**	**5.743**	**4.597**
Plectus	Clarkus	**-0.583**	**-0.310**	**5.743**	**4.597**
Plectus	Mylonchulus	**-0.583**	**-0.005**	**5.743**	**4.597**
Plectus	Tripyla	**-0.583**	**-0.420**	**5.743**	**5.199**
Plectus	Dendrolaelaps	**-0.583**	**0.027**	**5.743**	**2.775**
Plectus	Aporcelaimellus	**-0.583**	**0.548**	**5.743**	**5.199**
Plectus	Dorylaimoidea	**-0.583**	**-0.604**	**5.743**	**5.500**
Plectus	Epidorylaimus	**-0.583**	**0.199**	**5.743**	**4.597**
Plectus	Eudorylaimus	**-0.583**	**-0.166**	**5.743**	**5.296**
Plectus	Mesodorylaimus	**-0.583**	**-0.277**	**5.743**	**4.597**
Plectus	Nordiidae	**-0.583**	**-0.765**	**5.743**	**4.898**
Plectus	Pungentus	**-0.583**	**0.263**	**5.743**	**4.898**
Plectus	Qudsianematidae	**-0.583**	**-0.207**	**5.743**	**4.898**
Plectus	Thornematidae	**-0.583**	**-0.470**	**5.743**	**5.199**
Plectus	Eupodes	**-0.583**	**0.005**	**5.743**	**3.474**

Plectus	Mesostigmata	**-0.583**	**-0.411**	**5.743**	**3.076**
Plectus	Oribatida	**-0.583**	**-0.411**	**5.743**	**2.775**
Plectus	Scheloribates	**-0.583**	**0.202**	**5.743**	**3.474**
Plectus	Scutacarus	**-0.583**	**-0.608**	**5.743**	**2.775**
Prismatolaimus	Anatonchus	**-1.280**	**0.406**	**4.898**	**4.597**
Prismatolaimus	Clarkus	**-1.280**	**-0.310**	**4.898**	**4.597**
Prismatolaimus	Mylonchulus	**-1.280**	**-0.005**	**4.898**	**4.597**
Prismatolaimus	Tripyla	**-1.280**	**-0.420**	**4.898**	**5.199**
Prismatolaimus	Dendrolaelaps	**-1.280**	**0.027**	**4.898**	**2.775**
Prismatolaimus	Aporcelaimellus	**-1.280**	**0.548**	**4.898**	**5.199**
Prismatolaimus	Dorylaimoidea	**-1.280**	**-0.604**	**4.898**	**5.500**
Prismatolaimus	Epidorylaimus	**-1.280**	**0.199**	**4.898**	**4.597**
Prismatolaimus	Eudorylaimus	**-1.280**	**-0.166**	**4.898**	**5.296**
Prismatolaimus	Mesodorylaimus	**-1.280**	**-0.277**	**4.898**	**4.597**
Prismatolaimus	Nordiidae	**-1.280**	**-0.765**	**4.898**	**4.898**
Prismatolaimus	Pungentus	**-1.280**	**0.263**	**4.898**	**4.898**
Prismatolaimus	Qudsianematidae	**-1.280**	**-0.207**	**4.898**	**4.898**
Prismatolaimus	Thornematidae	**-1.280**	**-0.470**	**4.898**	**5.199**
Prismatolaimus	Eupodes	**-1.280**	**0.005**	**4.898**	**3.474**
Prismatolaimus	Mesostigmata	**-1.280**	**-0.411**	**4.898**	**3.076**
Prismatolaimus	Oribatida	**-1.280**	**-0.411**	**4.898**	**2.775**
Prismatolaimus	Scheloribates	**-1.280**	**0.202**	**4.898**	**3.474**
Prismatolaimus	Scutacarus	**-1.280**	**-0.608**	**4.898**	**2.775**
Teratocephalus	Anatonchus	**-1.630**	**0.406**	**5.375**	**4.597**
Teratocephalus	Clarkus	**-1.630**	**-0.310**	**5.375**	**4.597**
Teratocephalus	Mylonchulus	**-1.630**	**-0.005**	**5.375**	**4.597**
Teratocephalus	Tripyla	**-1.630**	**-0.420**	**5.375**	**5.199**
Teratocephalus	Dendrolaelaps	**-1.630**	**0.027**	**5.375**	**2.775**
Teratocephalus	Aporcelaimellus	**-1.630**	**0.548**	**5.375**	**5.199**
Teratocephalus	Dorylaimoidea	**-1.630**	**-0.604**	**5.375**	**5.500**
Teratocephalus	Epidorylaimus	**-1.630**	**0.199**	**5.375**	**4.597**
Teratocephalus	Eudorylaimus	**-1.630**	**-0.166**	**5.375**	**5.296**
Teratocephalus	Mesodorylaimus	**-1.630**	**-0.277**	**5.375**	**4.597**
Teratocephalus	Nordiidae	**-1.630**	**-0.765**	**5.375**	**4.898**
Teratocephalus	Pungentus	**-1.630**	**0.263**	**5.375**	**4.898**
Teratocephalus	Qudsianematidae	**-1.630**	**-0.207**	**5.375**	**4.898**
Teratocephalus	Thornematidae	**-1.630**	**-0.470**	**5.375**	**5.199**
Teratocephalus	Eupodes	**-1.630**	**0.005**	**5.375**	**3.474**
Teratocephalus	Mesostigmata	**-1.630**	**-0.411**	**5.375**	**3.076**
Teratocephalus	Oribatida	**-1.630**	**-0.411**	**5.375**	**2.775**
Teratocephalus	Scheloribates	**-1.630**	**0.202**	**5.375**	**3.474**
Teratocephalus	Scutacarus	**-1.630**	**-0.608**	**5.375**	**2.775**
Enchytraeus	Dendrolaelaps	**1.268**	**0.027**	**3.478**	**2.775**
Enchytraeus	Aporcelaimellus	**1.268**	**0.548**	**3.478**	**5.199**
Enchytraeus	Dorylaimoidea	**1.268**	**-0.604**	**3.478**	**5.500**
Enchytraeus	Epidorylaimus	**1.268**	**0.199**	**3.478**	**4.597**
Enchytraeus	Eudorylaimus	**1.268**	**-0.166**	**3.478**	**5.296**
Enchytraeus	Mesodorylaimus	**1.268**	**-0.277**	**3.478**	**4.597**
Enchytraeus	Nordiidae	**1.268**	**-0.765**	**3.478**	**4.898**
Enchytraeus	Pungentus	**1.268**	**0.263**	**3.478**	**4.898**
Enchytraeus	Qudsianematidae	**1.268**	**-0.207**	**3.478**	**4.898**

Enchytraeus	Thornematidae	**1.268**	**-0.470**	**3.478**	**5.199**
Enchytraeus	Eupodes	**1.268**	**0.005**	**3.478**	**3.474**
Enchytraeus	Mesostigmata	**1.268**	**-0.411**	**3.478**	**3.076**
Enchytraeus	Oribatida	**1.268**	**-0.411**	**3.478**	**2.775**
Enchytraeus	Scheloribates	**1.268**	**0.202**	**3.478**	**3.474**
Enchytraeus	Scutacarus	**1.268**	**-0.608**	**3.478**	**2.775**
Eubacteria	Alaimus	**-6.579**	**-0.858**	**12.414**	**4.597**
Eubacteria	Anaplectus	**-6.579**	**-0.519**	**12.414**	**5.074**
Eubacteria	Eucephalobus	**-6.579**	**-0.855**	**12.414**	**5.801**
Eubacteria	Metateratocephalus	**-6.579**	**-1.506**	**12.414**	**5.074**
Eubacteria	Panagrolaimus	**-6.579**	**-0.945**	**12.414**	**4.898**
Eubacteria	Plectus	**-6.579**	**-0.583**	**12.414**	**5.743**
Eubacteria	Prismatolaimus	**-6.579**	**-1.280**	**12.414**	**4.898**
Eubacteria	Teratocephalus	**-6.579**	**-1.630**	**12.414**	**5.375**
Eubacteria	Enchytraeus	**-6.579**	**1.268**	**12.414**	**3.478**
Eubacteria	Buchholzia	**-6.579**	**0.752**	**12.414**	**2.248**
Eubacteria	Henlea	**-6.579**	**1.087**	**12.414**	**2.946**
Eubacteria	Marionina	**-6.579**	**0.967**	**12.414**	**3.725**
Buchholzia	Dendrolaelaps	**0.752**	**0.027**	**2.248**	**2.775**
Buchholzia	Aporcelaimellus	**0.752**	**0.548**	**2.248**	**5.199**
Buchholzia	Dorylaimoidea	**0.752**	**-0.604**	**2.248**	**5.500**
Buchholzia	Epidorylaimus	**0.752**	**0.199**	**2.248**	**4.597**
Buchholzia	Eudorylaimus	**0.752**	**-0.166**	**2.248**	**5.296**
Buchholzia	Mesodorylaimus	**0.752**	**-0.277**	**2.248**	**4.597**
Buchholzia	Nordiidae	**0.752**	**-0.765**	**2.248**	**4.898**
Buchholzia	Pungentus	**0.752**	**0.263**	**2.248**	**4.898**
Buchholzia	Qudsianematidae	**0.752**	**-0.207**	**2.248**	**4.898**
Buchholzia	Thornematidae	**0.752**	**-0.470**	**2.248**	**5.199**
Buchholzia	Eupodes	**0.752**	**0.005**	**2.248**	**3.474**
Buchholzia	Mesostigmata	**0.752**	**-0.411**	**2.248**	**3.076**
Buchholzia	Oribatida	**0.752**	**-0.411**	**2.248**	**2.775**
Buchholzia	Scheloribates	**0.752**	**0.202**	**2.248**	**3.474**
Buchholzia	Scutacarus	**0.752**	**-0.608**	**2.248**	**2.775**
Henlea	Dendrolaelaps	**1.087**	**0.027**	**2.946**	**2.775**
Henlea	Aporcelaimellus	**1.087**	**0.548**	**2.946**	**5.199**
Henlea	Dorylaimoidea	**1.087**	**-0.604**	**2.946**	**5.500**
Henlea	Epidorylaimus	**1.087**	**0.199**	**2.946**	**4.597**
Henlea	Eudorylaimus	**1.087**	**-0.166**	**2.946**	**5.296**
Henlea	Mesodorylaimus	**1.087**	**-0.277**	**2.946**	**4.597**
Henlea	Nordiidae	**1.087**	**-0.765**	**2.946**	**4.898**
Henlea	Pungentus	**1.087**	**0.263**	**2.946**	**4.898**
Henlea	Qudsianematidae	**1.087**	**-0.207**	**2.946**	**4.898**
Henlea	Thornematidae	**1.087**	**-0.470**	**2.946**	**5.199**
Henlea	Eupodes	**1.087**	**0.005**	**2.946**	**3.474**
Henlea	Mesostigmata	**1.087**	**-0.411**	**2.946**	**3.076**
Henlea	Oribatida	**1.087**	**-0.411**	**2.946**	**2.775**
Henlea	Scheloribates	**1.087**	**0.202**	**2.946**	**3.474**
Henlea	Scutacarus	**1.087**	**-0.608**	**2.946**	**2.775**
Marionina	Dendrolaelaps	**0.967**	**0.027**	**3.725**	**2.775**
Marionina	Aporcelaimellus	**0.967**	**0.548**	**3.725**	**5.199**
Marionina	Dorylaimoidea	**0.967**	**-0.604**	**3.725**	**5.500**

Marionina	Epidorylaimus	**0.967**	**0.199**	**3.725**	**4.597**
Marionina	Eudorylaimus	**0.967**	**-0.166**	**3.725**	**5.296**
Marionina	Mesodorylaimus	**0.967**	**-0.277**	**3.725**	**4.597**
Marionina	Nordiidae	**0.967**	**-0.765**	**3.725**	**4.898**
Marionina	Pungentus	**0.967**	**0.263**	**3.725**	**4.898**
Marionina	Qudsianematidae	**0.967**	**-0.207**	**3.725**	**4.898**
Marionina	Thornematidae	**0.967**	**-0.470**	**3.725**	**5.199**
Marionina	Eupodes	**0.967**	**0.005**	**3.725**	**3.474**
Marionina	Mesostigmata	**0.967**	**-0.411**	**3.725**	**3.076**
Marionina	Oribatida	**0.967**	**-0.411**	**3.725**	**2.775**
Marionina	Scheloribates	**0.967**	**0.202**	**3.725**	**3.474**
Marionina	Scutacarus	**0.967**	**-0.608**	**3.725**	**2.775**
Hyphae and hair roots	Aglenchus	**5.905**	**-1.053**	**0.000**	**5.199**
Hyphae and hair roots	Dolichodoridae	**5.905**	**-0.885**	**0.000**	**5.074**
Hyphae and hair roots	Helicotylenchus	**5.905**	**-0.792**	**0.000**	**5.898**
Hyphae and hair roots	Malenchus	**5.905**	**-1.330**	**0.000**	**4.597**
Hyphae and hair roots	Pratylenchus	**5.905**	**-1.226**	**0.000**	**5.199**
Hyphae and hair roots	Tylenchorhynchus	**5.905**	**-0.664**	**0.000**	**5.296**
Hyphae and hair roots	Achipteria	**5.905**	**0.341**	**0.000**	**3.076**
Hyphae and hair roots	Galumna	**5.905**	**0.474**	**0.000**	**3.076**
Hyphae and hair roots	Platynothrus	**5.905**	**0.710**	**0.000**	**3.076**
Hyphae and hair roots	Sminthuridae	**5.905**	**-0.608**	**0.000**	**3.377**
Hyphae and hair roots	Sminthurinus	**5.905**	**0.618**	**0.000**	**2.775**
Hyphae and hair roots	Sminthurus	**5.905**	**1.429**	**0.000**	**3.076**
Hyphae and hair roots	Sphaeridia	**5.905**	**0.202**	**0.000**	**3.252**
Hyphae and hair roots	Achromadora	**5.905**	**-1.396**	**0.000**	**4.597**
Hyphae and hair roots	Aphelenchoides	**5.905**	**-1.496**	**0.000**	**4.898**
Hyphae and hair roots	Tylenchidae	**5.905**	**-1.360**	**0.000**	**6.029**
Hyphae and hair roots	Medioppia	**5.905**	**-0.235**	**0.000**	**3.076**
Hyphae and hair roots	Microppia	**5.905**	**-0.544**	**0.000**	**2.775**
Hyphae and hair roots	Minunthozetes	**5.905**	**-0.249**	**0.000**	**2.775**
Hyphae and hair roots	Tectocepheus	**5.905**	**-0.220**	**0.000**	**2.775**
Hyphae and hair roots	Folsomia	**5.905**	**0.900**	**0.000**	**2.775**
Hyphae and hair roots	Friesea	**5.905**	**0.434**	**0.000**	**3.076**
Hyphae and hair roots	Isotoma	**5.905**	**1.898**	**0.000**	**3.816**
Hyphae and hair roots	Isotomurus	**5.905**	**1.787**	**0.000**	**3.252**
Hyphae and hair roots	Lepidocyrtus	**5.905**	**1.231**	**0.000**	**3.474**
Hyphae and hair roots	Onychiurus	**5.905**	**0.977**	**0.000**	**2.775**
Hyphae and hair roots	Parisotoma	**5.905**	**0.722**	**0.000**	**3.678**
Hyphae and hair roots	Proisotoma	**5.905**	**0.770**	**0.000**	**2.775**
Hyphae and hair roots	Achaeta	**5.905**	**1.320**	**0.000**	**2.616**
Hyphae and hair roots	Cognettia	**5.905**	**1.559**	**0.000**	**3.556**
Hyphae and hair roots	Fridericia	**5.905**	**2.453**	**0.000**	**3.351**
Hyphae and hair roots	Aporcelaimellus	**5.905**	**0.548**	**0.000**	**5.199**
Hyphae and hair roots	Dorylaimoidea	**5.905**	**-0.604**	**0.000**	**5.500**
Hyphae and hair roots	Epidorylaimus	**5.905**	**0.199**	**0.000**	**4.597**
Hyphae and hair roots	Eudorylaimus	**5.905**	**-0.166**	**0.000**	**5.296**
Hyphae and hair roots	Mesodorylaimus	**5.905**	**-0.277**	**0.000**	**4.597**
Hyphae and hair roots	Nordiidae	**5.905**	**-0.765**	**0.000**	**4.898**
Hyphae and hair roots	Pungentus	**5.905**	**0.263**	**0.000**	**4.898**
Hyphae and hair roots	Qudsianematidae	**5.905**	**-0.207**	**0.000**	**4.898**

Hyphae and hair roots	Thornematidae	**5.905**	**-0.470**	**0.000**	**5.199**
Hyphae and hair roots	Eupodes	**5.905**	**0.005**	**0.000**	**3.474**
Hyphae and hair roots	Mesostigmata	**5.905**	**-0.411**	**0.000**	**3.076**
Hyphae and hair roots	Oribatida	**5.905**	**-0.411**	**0.000**	**2.775**
Hyphae and hair roots	Scheloribates	**5.905**	**0.202**	**0.000**	**3.474**
Hyphae and hair roots	Scutacarus	**5.905**	**-0.608**	**0.000**	**2.775**
Anatonchus	Dendrolaelaps	**0.406**	**0.027**	**4.597**	**2.775**
Anatonchus	Aporcelaimellus	**0.406**	**0.548**	**4.597**	**5.199**
Anatonchus	Dorylaimoidea	**0.406**	**-0.604**	**4.597**	**5.500**
Anatonchus	Epidorylaimus	**0.406**	**0.199**	**4.597**	**4.597**
Anatonchus	Eudorylaimus	**0.406**	**-0.166**	**4.597**	**5.296**
Anatonchus	Mesodorylaimus	**0.406**	**-0.277**	**4.597**	**4.597**
Anatonchus	Nordiidae	**0.406**	**-0.765**	**4.597**	**4.898**
Anatonchus	Pungentus	**0.406**	**0.263**	**4.597**	**4.898**
Anatonchus	Qudsianematidae	**0.406**	**-0.207**	**4.597**	**4.898**
Anatonchus	Thornematidae	**0.406**	**-0.470**	**4.597**	**5.199**
Anatonchus	Eupodes	**0.406**	**0.005**	**4.597**	**3.474**
Anatonchus	Mesostigmata	**0.406**	**-0.411**	**4.597**	**3.076**
Anatonchus	Oribatida	**0.406**	**-0.411**	**4.597**	**2.775**
Anatonchus	Scheloribates	**0.406**	**0.202**	**4.597**	**3.474**
Anatonchus	Scutacarus	**0.406**	**-0.608**	**4.597**	**2.775**
Clarkus	Dendrolaelaps	**-0.310**	**0.027**	**4.597**	**2.775**
Clarkus	Aporcelaimellus	**-0.310**	**0.548**	**4.597**	**5.199**
Clarkus	Dorylaimoidea	**-0.310**	**-0.604**	**4.597**	**5.500**
Clarkus	Epidorylaimus	**-0.310**	**0.199**	**4.597**	**4.597**
Clarkus	Eudorylaimus	**-0.310**	**-0.166**	**4.597**	**5.296**
Clarkus	Mesodorylaimus	**-0.310**	**-0.277**	**4.597**	**4.597**
Clarkus	Nordiidae	**-0.310**	**-0.765**	**4.597**	**4.898**
Clarkus	Pungentus	**-0.310**	**0.263**	**4.597**	**4.898**
Clarkus	Qudsianematidae	**-0.310**	**-0.207**	**4.597**	**4.898**
Clarkus	Thornematidae	**-0.310**	**-0.470**	**4.597**	**5.199**
Clarkus	Eupodes	**-0.310**	**0.005**	**4.597**	**3.474**
Clarkus	Mesostigmata	**-0.310**	**-0.411**	**4.597**	**3.076**
Clarkus	Oribatida	**-0.310**	**-0.411**	**4.597**	**2.775**
Clarkus	Scheloribates	**-0.310**	**0.202**	**4.597**	**3.474**
Clarkus	Scutacarus	**-0.310**	**-0.608**	**4.597**	**2.775**
Mylonchulus	Dendrolaelaps	**-0.005**	**0.027**	**4.597**	**2.775**
Mylonchulus	Aporcelaimellus	**-0.005**	**0.548**	**4.597**	**5.199**
Mylonchulus	Dorylaimoidea	**-0.005**	**-0.604**	**4.597**	**5.500**
Mylonchulus	Epidorylaimus	**-0.005**	**0.199**	**4.597**	**4.597**
Mylonchulus	Eudorylaimus	**-0.005**	**-0.166**	**4.597**	**5.296**
Mylonchulus	Mesodorylaimus	**-0.005**	**-0.277**	**4.597**	**4.597**
Mylonchulus	Nordiidae	**-0.005**	**-0.765**	**4.597**	**4.898**
Mylonchulus	Pungentus	**-0.005**	**0.263**	**4.597**	**4.898**
Mylonchulus	Qudsianematidae	**-0.005**	**-0.207**	**4.597**	**4.898**
Mylonchulus	Thornematidae	**-0.005**	**-0.470**	**4.597**	**5.199**
Mylonchulus	Eupodes	**-0.005**	**0.005**	**4.597**	**3.474**
Mylonchulus	Mesostigmata	**-0.005**	**-0.411**	**4.597**	**3.076**
Mylonchulus	Oribatida	**-0.005**	**-0.411**	**4.597**	**2.775**
Mylonchulus	Scheloribates	**-0.005**	**0.202**	**4.597**	**3.474**
Mylonchulus	Scutacarus	**-0.005**	**-0.608**	**4.597**	**2.775**

Tripyla	Dendrolaelaps	**-0.420**	**0.027**	**5.199**	**2.775**
Tripyla	Aporcelaimellus	**-0.420**	**0.548**	**5.199**	**5.199**
Tripyla	Dorylaimoidea	**-0.420**	**-0.604**	**5.199**	**5.500**
Tripyla	Epidorylaimus	**-0.420**	**0.199**	**5.199**	**4.597**
Tripyla	Eudorylaimus	**-0.420**	**-0.166**	**5.199**	**5.296**
Tripyla	Mesodorylaimus	**-0.420**	**-0.277**	**5.199**	**4.597**
Tripyla	Nordiidae	**-0.420**	**-0.765**	**5.199**	**4.898**
Tripyla	Pungentus	**-0.420**	**0.263**	**5.199**	**4.898**
Tripyla	Qudsianematidae	**-0.420**	**-0.207**	**5.199**	**4.898**
Tripyla	Thornematidae	**-0.420**	**-0.470**	**5.199**	**5.199**
Tripyla	Eupodes	**-0.420**	**0.005**	**5.199**	**3.474**
Tripyla	Mesostigmata	**-0.420**	**-0.411**	**5.199**	**3.076**
Tripyla	Oribatida	**-0.420**	**-0.411**	**5.199**	**2.775**
Tripyla	Scheloribates	**-0.420**	**0.202**	**5.199**	**3.474**
Tripyla	Scutacarus	**-0.420**	**-0.608**	**5.199**	**2.775**
Dendrolaelaps	Aporcelaimellus	**0.027**	**0.548**	**2.775**	**5.199**
Dendrolaelaps	Dorylaimoidea	**0.027**	**-0.604**	**2.775**	**5.500**
Dendrolaelaps	Epidorylaimus	**0.027**	**0.199**	**2.775**	**4.597**
Dendrolaelaps	Eudorylaimus	**0.027**	**-0.166**	**2.775**	**5.296**
Dendrolaelaps	Mesodorylaimus	**0.027**	**-0.277**	**2.775**	**4.597**
Dendrolaelaps	Nordiidae	**0.027**	**-0.765**	**2.775**	**4.898**
Dendrolaelaps	Pungentus	**0.027**	**0.263**	**2.775**	**4.898**
Dendrolaelaps	Qudsianematidae	**0.027**	**-0.207**	**2.775**	**4.898**
Dendrolaelaps	Thornematidae	**0.027**	**-0.470**	**2.775**	**5.199**
Dendrolaelaps	Eupodes	**0.027**	**0.005**	**2.775**	**3.474**
Dendrolaelaps	Mesostigmata	**0.027**	**-0.411**	**2.775**	**3.076**
Dendrolaelaps	Oribatida	**0.027**	**-0.411**	**2.775**	**2.775**
Dendrolaelaps	Scheloribates	**0.027**	**0.202**	**2.775**	**3.474**
Dendrolaelaps	Scutacarus	**0.027**	**-0.608**	**2.775**	**2.775**
Aporcelaimellus	Anatonchus	**0.548**	**0.406**	**5.199**	**4.597**
Aporcelaimellus	Clarkus	**0.548**	**-0.310**	**5.199**	**4.597**
Aporcelaimellus	Mylonchulus	**0.548**	**-0.005**	**5.199**	**4.597**
Aporcelaimellus	Tripyla	**0.548**	**-0.420**	**5.199**	**5.199**
Aporcelaimellus	Dendrolaelaps	**0.548**	**0.027**	**5.199**	**2.775**
Aporcelaimellus	Aporcelaimellus	**0.548**	**0.548**	**5.199**	**5.199**
Aporcelaimellus	Dorylaimoidea	**0.548**	**-0.604**	**5.199**	**5.500**
Aporcelaimellus	Epidorylaimus	**0.548**	**0.199**	**5.199**	**4.597**
Aporcelaimellus	Eudorylaimus	**0.548**	**-0.166**	**5.199**	**5.296**
Aporcelaimellus	Mesodorylaimus	**0.548**	**-0.277**	**5.199**	**4.597**
Aporcelaimellus	Nordiidae	**0.548**	**-0.765**	**5.199**	**4.898**
Aporcelaimellus	Pungentus	**0.548**	**0.263**	**5.199**	**4.898**
Aporcelaimellus	Qudsianematidae	**0.548**	**-0.207**	**5.199**	**4.898**
Aporcelaimellus	Thornematidae	**0.548**	**-0.470**	**5.199**	**5.199**
Aporcelaimellus	Eupodes	**0.548**	**0.005**	**5.199**	**3.474**
Aporcelaimellus	Mesostigmata	**0.548**	**-0.411**	**5.199**	**3.076**
Aporcelaimellus	Oribatida	**0.548**	**-0.411**	**5.199**	**2.775**
Aporcelaimellus	Scheloribates	**0.548**	**0.202**	**5.199**	**3.474**
Aporcelaimellus	Scutacarus	**0.548**	**-0.608**	**5.199**	**2.775**
Dorylaimoidea	Anatonchus	**-0.604**	**0.406**	**5.500**	**4.597**
Dorylaimoidea	Clarkus	**-0.604**	**-0.310**	**5.500**	**4.597**
Dorylaimoidea	Mylonchulus	**-0.604**	**-0.005**	**5.500**	**4.597**

Dorylaimoidea	Tripyla	**-0.604**	**-0.420**	**5.500**	**5.199**
Dorylaimoidea	Dendrolaelaps	**-0.604**	**0.027**	**5.500**	**2.775**
Dorylaimoidea	Aporcelaimellus	**-0.604**	**0.548**	**5.500**	**5.199**
Dorylaimoidea	Dorylaimoidea	**-0.604**	**-0.604**	**5.500**	**5.500**
Dorylaimoidea	Epidorylaimus	**-0.604**	**0.199**	**5.500**	**4.597**
Dorylaimoidea	Eudorylaimus	**-0.604**	**-0.166**	**5.500**	**5.296**
Dorylaimoidea	Mesodorylaimus	**-0.604**	**-0.277**	**5.500**	**4.597**
Dorylaimoidea	Nordiidae	**-0.604**	**-0.765**	**5.500**	**4.898**
Dorylaimoidea	Pungentus	**-0.604**	**0.263**	**5.500**	**4.898**
Dorylaimoidea	Qudsianematidae	**-0.604**	**-0.207**	**5.500**	**4.898**
Dorylaimoidea	Thornematidae	**-0.604**	**-0.470**	**5.500**	**5.199**
Dorylaimoidea	Eupodes	**-0.604**	**0.005**	**5.500**	**3.474**
Dorylaimoidea	Mesostigmata	**-0.604**	**-0.411**	**5.500**	**3.076**
Dorylaimoidea	Oribatida	**-0.604**	**-0.411**	**5.500**	**2.775**
Dorylaimoidea	Scheloribates	**-0.604**	**0.202**	**5.500**	**3.474**
Dorylaimoidea	Scutacarus	**-0.604**	**-0.608**	**5.500**	**2.775**
Epidorylaimus	Anatonchus	**0.199**	**0.406**	**4.597**	**4.597**
Epidorylaimus	Clarkus	**0.199**	**-0.310**	**4.597**	**4.597**
Epidorylaimus	Mylonchulus	**0.199**	**-0.005**	**4.597**	**4.597**
Epidorylaimus	Tripyla	**0.199**	**-0.420**	**4.597**	**5.199**
Epidorylaimus	Dendrolaelaps	**0.199**	**0.027**	**4.597**	**2.775**
Epidorylaimus	Aporcelaimellus	**0.199**	**0.548**	**4.597**	**5.199**
Epidorylaimus	Dorylaimoidea	**0.199**	**-0.604**	**4.597**	**5.500**
Epidorylaimus	Epidorylaimus	**0.199**	**0.199**	**4.597**	**4.597**
Epidorylaimus	Eudorylaimus	**0.199**	**-0.166**	**4.597**	**5.296**
Epidorylaimus	Mesodorylaimus	**0.199**	**-0.277**	**4.597**	**4.597**
Epidorylaimus	Nordiidae	**0.199**	**-0.765**	**4.597**	**4.898**
Epidorylaimus	Pungentus	**0.199**	**0.263**	**4.597**	**4.898**
Epidorylaimus	Qudsianematidae	**0.199**	**-0.207**	**4.597**	**4.898**
Epidorylaimus	Thornematidae	**0.199**	**-0.470**	**4.597**	**5.199**
Epidorylaimus	Eupodes	**0.199**	**0.005**	**4.597**	**3.474**
Epidorylaimus	Mesostigmata	**0.199**	**-0.411**	**4.597**	**3.076**
Epidorylaimus	Oribatida	**0.199**	**-0.411**	**4.597**	**2.775**
Epidorylaimus	Scheloribates	**0.199**	**0.202**	**4.597**	**3.474**
Epidorylaimus	Scutacarus	**0.199**	**-0.608**	**4.597**	**2.775**
Eudorylaimus	Anatonchus	**-0.166**	**0.406**	**5.296**	**4.597**
Eudorylaimus	Clarkus	**-0.166**	**-0.310**	**5.296**	**4.597**
Eudorylaimus	Mylonchulus	**-0.166**	**-0.005**	**5.296**	**4.597**
Eudorylaimus	Tripyla	**-0.166**	**-0.420**	**5.296**	**5.199**
Eudorylaimus	Dendrolaelaps	**-0.166**	**0.027**	**5.296**	**2.775**
Eudorylaimus	Aporcelaimellus	**-0.166**	**0.548**	**5.296**	**5.199**
Eudorylaimus	Dorylaimoidea	**-0.166**	**-0.604**	**5.296**	**5.500**
Eudorylaimus	Epidorylaimus	**-0.166**	**0.199**	**5.296**	**4.597**
Eudorylaimus	Eudorylaimus	**-0.166**	**-0.166**	**5.296**	**5.296**
Eudorylaimus	Mesodorylaimus	**-0.166**	**-0.277**	**5.296**	**4.597**
Eudorylaimus	Nordiidae	**-0.166**	**-0.765**	**5.296**	**4.898**
Eudorylaimus	Pungentus	**-0.166**	**0.263**	**5.296**	**4.898**
Eudorylaimus	Qudsianematidae	**-0.166**	**-0.207**	**5.296**	**4.898**
Eudorylaimus	Thornematidae	**-0.166**	**-0.470**	**5.296**	**5.199**
Eudorylaimus	Eupodes	**-0.166**	**0.005**	**5.296**	**3.474**
Eudorylaimus	Mesostigmata	**-0.166**	**-0.411**	**5.296**	**3.076**

Eudorylaimus	Oribatida	**-0.166**	**-0.411**	**5.296**	**2.775**
Eudorylaimus	Scheloribates	**-0.166**	**0.202**	**5.296**	**3.474**
Eudorylaimus	Scutacarus	**-0.166**	**-0.608**	**5.296**	**2.775**
Mesodorylaimus	Anatonchus	**-0.277**	**0.406**	**4.597**	**4.597**
Mesodorylaimus	Clarkus	**-0.277**	**-0.310**	**4.597**	**4.597**
Mesodorylaimus	Mylonchulus	**-0.277**	**-0.005**	**4.597**	**4.597**
Mesodorylaimus	Tripyla	**-0.277**	**-0.420**	**4.597**	**5.199**
Mesodorylaimus	Dendrolaelaps	**-0.277**	**0.027**	**4.597**	**2.775**
Mesodorylaimus	Aporcelaimellus	**-0.277**	**0.548**	**4.597**	**5.199**
Mesodorylaimus	Dorylaimoidea	**-0.277**	**-0.604**	**4.597**	**5.500**
Mesodorylaimus	Epidorylaimus	**-0.277**	**0.199**	**4.597**	**4.597**
Mesodorylaimus	Eudorylaimus	**-0.277**	**-0.166**	**4.597**	**5.296**
Mesodorylaimus	Mesodorylaimus	**-0.277**	**-0.277**	**4.597**	**4.597**
Mesodorylaimus	Nordiidae	**-0.277**	**-0.765**	**4.597**	**4.898**
Mesodorylaimus	Pungentus	**-0.277**	**0.263**	**4.597**	**4.898**
Mesodorylaimus	Qudsianematidae	**-0.277**	**-0.207**	**4.597**	**4.898**
Mesodorylaimus	Thornematidae	**-0.277**	**-0.470**	**4.597**	**5.199**
Mesodorylaimus	Eupodes	**-0.277**	**0.005**	**4.597**	**3.474**
Mesodorylaimus	Mesostigmata	**-0.277**	**-0.411**	**4.597**	**3.076**
Mesodorylaimus	Oribatida	**-0.277**	**-0.411**	**4.597**	**2.775**
Mesodorylaimus	Scheloribates	**-0.277**	**0.202**	**4.597**	**3.474**
Mesodorylaimus	Scutacarus	**-0.277**	**-0.608**	**4.597**	**2.775**
Nordiidae	Anatonchus	**-0.765**	**0.406**	**4.898**	**4.597**
Nordiidae	Clarkus	**-0.765**	**-0.310**	**4.898**	**4.597**
Nordiidae	Mylonchulus	**-0.765**	**-0.005**	**4.898**	**4.597**
Nordiidae	Tripyla	**-0.765**	**-0.420**	**4.898**	**5.199**
Nordiidae	Dendrolaelaps	**-0.765**	**0.027**	**4.898**	**2.775**
Nordiidae	Aporcelaimellus	**-0.765**	**0.548**	**4.898**	**5.199**
Nordiidae	Dorylaimoidea	**-0.765**	**-0.604**	**4.898**	**5.500**
Nordiidae	Epidorylaimus	**-0.765**	**0.199**	**4.898**	**4.597**
Nordiidae	Eudorylaimus	**-0.765**	**-0.166**	**4.898**	**5.296**
Nordiidae	Mesodorylaimus	**-0.765**	**-0.277**	**4.898**	**4.597**
Nordiidae	Nordiidae	**-0.765**	**-0.765**	**4.898**	**4.898**
Nordiidae	Pungentus	**-0.765**	**0.263**	**4.898**	**4.898**
Nordiidae	Qudsianematidae	**-0.765**	**-0.207**	**4.898**	**4.898**
Nordiidae	Thornematidae	**-0.765**	**-0.470**	**4.898**	**5.199**
Nordiidae	Eupodes	**-0.765**	**0.005**	**4.898**	**3.474**
Nordiidae	Mesostigmata	**-0.765**	**-0.411**	**4.898**	**3.076**
Nordiidae	Oribatida	**-0.765**	**-0.411**	**4.898**	**2.775**
Nordiidae	Scheloribates	**-0.765**	**0.202**	**4.898**	**3.474**
Nordiidae	Scutacarus	**-0.765**	**-0.608**	**4.898**	**2.775**
Pungentus	Anatonchus	**0.263**	**0.406**	**4.898**	**4.597**
Pungentus	Clarkus	**0.263**	**-0.310**	**4.898**	**4.597**
Pungentus	Mylonchulus	**0.263**	**-0.005**	**4.898**	**4.597**
Pungentus	Tripyla	**0.263**	**-0.420**	**4.898**	**5.199**
Pungentus	Dendrolaelaps	**0.263**	**0.027**	**4.898**	**2.775**
Pungentus	Aporcelaimellus	**0.263**	**0.548**	**4.898**	**5.199**
Pungentus	Dorylaimoidea	**0.263**	**-0.604**	**4.898**	**5.500**
Pungentus	Epidorylaimus	**0.263**	**0.199**	**4.898**	**4.597**
Pungentus	Eudorylaimus	**0.263**	**-0.166**	**4.898**	**5.296**
Pungentus	Mesodorylaimus	**0.263**	**-0.277**	**4.898**	**4.597**

Pungentus	Nordiidae	**0.263**	**-0.765**	**4.898**	**4.898**
Pungentus	Pungentus	**0.263**	**0.263**	**4.898**	**4.898**
Pungentus	Qudsianematidae	**0.263**	**-0.207**	**4.898**	**4.898**
Pungentus	Thornematidae	**0.263**	**-0.470**	**4.898**	**5.199**
Pungentus	Eupodes	**0.263**	**0.005**	**4.898**	**3.474**
Pungentus	Mesostigmata	**0.263**	**-0.411**	**4.898**	**3.076**
Pungentus	Oribatida	**0.263**	**-0.411**	**4.898**	**2.775**
Pungentus	Scheloribates	**0.263**	**0.202**	**4.898**	**3.474**
Pungentus	Scutacarus	**0.263**	**-0.608**	**4.898**	**2.775**
Qudsianematidae	Anatonchus	**-0.207**	**0.406**	**4.898**	**4.597**
Qudsianematidae	Clarkus	**-0.207**	**-0.310**	**4.898**	**4.597**
Qudsianematidae	Mylonchulus	**-0.207**	**-0.005**	**4.898**	**4.597**
Qudsianematidae	Tripyla	**-0.207**	**-0.420**	**4.898**	**5.199**
Qudsianematidae	Dendrolaelaps	**-0.207**	**0.027**	**4.898**	**2.775**
Qudsianematidae	Aporcelaimellus	**-0.207**	**0.548**	**4.898**	**5.199**
Qudsianematidae	Dorylaimoidea	**-0.207**	**-0.604**	**4.898**	**5.500**
Qudsianematidae	Epidorylaimus	**-0.207**	**0.199**	**4.898**	**4.597**
Qudsianematidae	Eudorylaimus	**-0.207**	**-0.166**	**4.898**	**5.296**
Qudsianematidae	Mesodorylaimus	**-0.207**	**-0.277**	**4.898**	**4.597**
Qudsianematidae	Nordiidae	**-0.207**	**-0.765**	**4.898**	**4.898**
Qudsianematidae	Pungentus	**-0.207**	**0.263**	**4.898**	**4.898**
Qudsianematidae	Qudsianematidae	**-0.207**	**-0.207**	**4.898**	**4.898**
Qudsianematidae	Thornematidae	**-0.207**	**-0.470**	**4.898**	**5.199**
Qudsianematidae	Eupodes	**-0.207**	**0.005**	**4.898**	**3.474**
Qudsianematidae	Mesostigmata	**-0.207**	**-0.411**	**4.898**	**3.076**
Qudsianematidae	Oribatida	**-0.207**	**-0.411**	**4.898**	**2.775**
Qudsianematidae	Scheloribates	**-0.207**	**0.202**	**4.898**	**3.474**
Qudsianematidae	Scutacarus	**-0.207**	**-0.608**	**4.898**	**2.775**
Thornematidae	Anatonchus	**-0.470**	**0.406**	**5.199**	**4.597**
Thornematidae	Clarkus	**-0.470**	**-0.310**	**5.199**	**4.597**
Thornematidae	Mylonchulus	**-0.470**	**-0.005**	**5.199**	**4.597**
Thornematidae	Tripyla	**-0.470**	**-0.420**	**5.199**	**5.199**
Thornematidae	Dendrolaelaps	**-0.470**	**0.027**	**5.199**	**2.775**
Thornematidae	Aporcelaimellus	**-0.470**	**0.548**	**5.199**	**5.199**
Thornematidae	Dorylaimoidea	**-0.470**	**-0.604**	**5.199**	**5.500**
Thornematidae	Epidorylaimus	**-0.470**	**0.199**	**5.199**	**4.597**
Thornematidae	Eudorylaimus	**-0.470**	**-0.166**	**5.199**	**5.296**
Thornematidae	Mesodorylaimus	**-0.470**	**-0.277**	**5.199**	**4.597**
Thornematidae	Nordiidae	**-0.470**	**-0.765**	**5.199**	**4.898**
Thornematidae	Pungentus	**-0.470**	**0.263**	**5.199**	**4.898**
Thornematidae	Qudsianematidae	**-0.470**	**-0.207**	**5.199**	**4.898**
Thornematidae	Thornematidae	**-0.470**	**-0.470**	**5.199**	**5.199**
Thornematidae	Eupodes	**-0.470**	**0.005**	**5.199**	**3.474**
Thornematidae	Mesostigmata	**-0.470**	**-0.411**	**5.199**	**3.076**
Thornematidae	Oribatida	**-0.470**	**-0.411**	**5.199**	**2.775**
Thornematidae	Scheloribates	**-0.470**	**0.202**	**5.199**	**3.474**
Thornematidae	Scutacarus	**-0.470**	**-0.608**	**5.199**	**2.775**
Eupodes	Dendrolaelaps	**0.005**	**0.027**	**3.474**	**2.775**
Eupodes	Aporcelaimellus	**0.005**	**0.548**	**3.474**	**5.199**
Eupodes	Dorylaimoidea	**0.005**	**-0.604**	**3.474**	**5.500**
Eupodes	Epidorylaimus	**0.005**	**0.199**	**3.474**	**4.597**

Eupodes	Eudorylaimus	**0.005**	**-0.166**	**3.474**	**5.296**
Eupodes	Mesodorylaimus	**0.005**	**-0.277**	**3.474**	**4.597**
Eupodes	Nordiidae	**0.005**	**-0.765**	**3.474**	**4.898**
Eupodes	Pungentus	**0.005**	**0.263**	**3.474**	**4.898**
Eupodes	Qudsianematidae	**0.005**	**-0.207**	**3.474**	**4.898**
Eupodes	Thornematidae	**0.005**	**-0.470**	**3.474**	**5.199**
Eupodes	Eupodes	**0.005**	**0.005**	**3.474**	**3.474**
Eupodes	Mesostigmata	**0.005**	**-0.411**	**3.474**	**3.076**
Eupodes	Oribatida	**0.005**	**-0.411**	**3.474**	**2.775**
Eupodes	Scheloribates	**0.005**	**0.202**	**3.474**	**3.474**
Eupodes	Scutacarus	**0.005**	**-0.608**	**3.474**	**2.775**
Mesostigmata	Dendrolaelaps	**-0.411**	**0.027**	**3.076**	**2.775**
Mesostigmata	Aporcelaimellus	**-0.411**	**0.548**	**3.076**	**5.199**
Mesostigmata	Dorylaimoidea	**-0.411**	**-0.604**	**3.076**	**5.500**
Mesostigmata	Epidorylaimus	**-0.411**	**0.199**	**3.076**	**4.597**
Mesostigmata	Eudorylaimus	**-0.411**	**-0.166**	**3.076**	**5.296**
Mesostigmata	Mesodorylaimus	**-0.411**	**-0.277**	**3.076**	**4.597**
Mesostigmata	Nordiidae	**-0.411**	**-0.765**	**3.076**	**4.898**
Mesostigmata	Pungentus	**-0.411**	**0.263**	**3.076**	**4.898**
Mesostigmata	Qudsianematidae	**-0.411**	**-0.207**	**3.076**	**4.898**
Mesostigmata	Thornematidae	**-0.411**	**-0.470**	**3.076**	**5.199**
Mesostigmata	Eupodes	**-0.411**	**0.005**	**3.076**	**3.474**
Mesostigmata	Mesostigmata	**-0.411**	**-0.411**	**3.076**	**3.076**
Mesostigmata	Oribatida	**-0.411**	**-0.411**	**3.076**	**2.775**
Mesostigmata	Scheloribates	**-0.411**	**0.202**	**3.076**	**3.474**
Mesostigmata	Scutacarus	**-0.411**	**-0.608**	**3.076**	**2.775**
Oribatida	Dendrolaelaps	**-0.411**	**0.027**	**2.775**	**2.775**
Oribatida	Aporcelaimellus	**-0.411**	**0.548**	**2.775**	**5.199**
Oribatida	Dorylaimoidea	**-0.411**	**-0.604**	**2.775**	**5.500**
Oribatida	Epidorylaimus	**-0.411**	**0.199**	**2.775**	**4.597**
Oribatida	Eudorylaimus	**-0.411**	**-0.166**	**2.775**	**5.296**
Oribatida	Mesodorylaimus	**-0.411**	**-0.277**	**2.775**	**4.597**
Oribatida	Nordiidae	**-0.411**	**-0.765**	**2.775**	**4.898**
Oribatida	Pungentus	**-0.411**	**0.263**	**2.775**	**4.898**
Oribatida	Qudsianematidae	**-0.411**	**-0.207**	**2.775**	**4.898**
Oribatida	Thornematidae	**-0.411**	**-0.470**	**2.775**	**5.199**
Oribatida	Eupodes	**-0.411**	**0.005**	**2.775**	**3.474**
Oribatida	Mesostigmata	**-0.411**	**-0.411**	**2.775**	**3.076**
Oribatida	Oribatida	**-0.411**	**-0.411**	**2.775**	**2.775**
Oribatida	Scheloribates	**-0.411**	**0.202**	**2.775**	**3.474**
Oribatida	Scutacarus	**-0.411**	**-0.608**	**2.775**	**2.775**
Scheloribates	Dendrolaelaps	**0.202**	**0.027**	**3.474**	**2.775**
Scheloribates	Aporcelaimellus	**0.202**	**0.548**	**3.474**	**5.199**
Scheloribates	Dorylaimoidea	**0.202**	**-0.604**	**3.474**	**5.500**
Scheloribates	Epidorylaimus	**0.202**	**0.199**	**3.474**	**4.597**
Scheloribates	Eudorylaimus	**0.202**	**-0.166**	**3.474**	**5.296**
Scheloribates	Mesodorylaimus	**0.202**	**-0.277**	**3.474**	**4.597**
Scheloribates	Nordiidae	**0.202**	**-0.765**	**3.474**	**4.898**
Scheloribates	Pungentus	**0.202**	**0.263**	**3.474**	**4.898**
Scheloribates	Qudsianematidae	**0.202**	**-0.207**	**3.474**	**4.898**
Scheloribates	Thornematidae	**0.202**	**-0.470**	**3.474**	**5.199**

Scheloribates	Eupodes	**0.202**	**0.005**	**3.474**	**3.474**
Scheloribates	Mesostigmata	**0.202**	**-0.411**	**3.474**	**3.076**
Scheloribates	Oribatida	**0.202**	**-0.411**	**3.474**	**2.775**
Scheloribates	Scheloribates	**0.202**	**0.202**	**3.474**	**3.474**
Scheloribates	Scutacarus	**0.202**	**-0.608**	**3.474**	**2.775**
Scutacarus	Dendrolaelaps	**-0.608**	**0.027**	**2.775**	**2.775**
Scutacarus	Aporcelaimellus	**-0.608**	**0.548**	**2.775**	**5.199**
Scutacarus	Dorylaimoidea	**-0.608**	**-0.604**	**2.775**	**5.500**
Scutacarus	Epidorylaimus	**-0.608**	**0.199**	**2.775**	**4.597**
Scutacarus	Eudorylaimus	**-0.608**	**-0.166**	**2.775**	**5.296**
Scutacarus	Mesodorylaimus	**-0.608**	**-0.277**	**2.775**	**4.597**
Scutacarus	Nordiidae	**-0.608**	**-0.765**	**2.775**	**4.898**
Scutacarus	Pungentus	**-0.608**	**0.263**	**2.775**	**4.898**
Scutacarus	Qudsianematidae	**-0.608**	**-0.207**	**2.775**	**4.898**
Scutacarus	Thornematidae	**-0.608**	**-0.470**	**2.775**	**5.199**
Scutacarus	Eupodes	**-0.608**	**0.005**	**2.775**	**3.474**
Scutacarus	Mesostigmata	**-0.608**	**-0.411**	**2.775**	**3.076**
Scutacarus	Oribatida	**-0.608**	**-0.411**	**2.775**	**2.775**
Scutacarus	Scheloribates	**-0.608**	**0.202**	**2.775**	**3.474**
Scutacarus	Scutacarus	**-0.608**	**-0.608**	**2.775**	**2.775**

Resource	Consumer	Mres	Mconsumer	Nres	Nconsumer
Aglenchus	Dendrolaelaps	**-1.053**	**0.027**	**5.375**	**3.150**
Aglenchus	Lysigamasus	**-1.053**	**0.407**	**5.375**	**3.150**
Aglenchus	Aporcelaimellus	**-1.053**	**0.548**	**5.375**	**5.484**
Aglenchus	Dorylaimoidea	**-1.053**	**-0.604**	**5.375**	**5.609**
Aglenchus	Eudorylaimus	**-1.053**	**-0.166**	**5.375**	**5.007**
Aglenchus	Mesodorylaimus	**-1.053**	**-0.277**	**5.375**	**4.530**
Aglenchus	Pungentus	**-1.053**	**0.263**	**5.375**	**5.132**
Aglenchus	Qudsianematidae	**-1.053**	**-0.207**	**5.375**	**4.831**
Aglenchus	Thornematidae	**-1.053**	**-0.470**	**5.375**	**4.530**
Aglenchus	Eupodes	**-1.053**	**0.005**	**5.375**	**4.451**
Aglenchus	Scutacarus	**-1.053**	**-0.608**	**5.375**	**3.627**
Dolichodoridae	Dendrolaelaps	**-0.885**	**0.027**	**5.229**	**3.150**
Dolichodoridae	Lysigamasus	**-0.885**	**0.407**	**5.229**	**3.150**
Dolichodoridae	Aporcelaimellus	**-0.885**	**0.548**	**5.229**	**5.484**
Dolichodoridae	Dorylaimoidea	**-0.885**	**-0.604**	**5.229**	**5.609**
Dolichodoridae	Eudorylaimus	**-0.885**	**-0.166**	**5.229**	**5.007**
Dolichodoridae	Mesodorylaimus	**-0.885**	**-0.277**	**5.229**	**4.530**
Dolichodoridae	Pungentus	**-0.885**	**0.263**	**5.229**	**5.132**
Dolichodoridae	Qudsianematidae	**-0.885**	**-0.207**	**5.229**	**4.831**
Dolichodoridae	Thornematidae	**-0.885**	**-0.470**	**5.229**	**4.530**
Dolichodoridae	Eupodes	**-0.885**	**0.005**	**5.229**	**4.451**
Dolichodoridae	Scutacarus	**-0.885**	**-0.608**	**5.229**	**3.627**
Helicotylenchus	Dendrolaelaps	**-0.792**	**0.027**	**5.676**	**3.150**
Helicotylenchus	Lysigamasus	**-0.792**	**0.407**	**5.676**	**3.150**
Helicotylenchus	Aporcelaimellus	**-0.792**	**0.548**	**5.676**	**5.484**
Helicotylenchus	Dorylaimoidea	**-0.792**	**-0.604**	**5.676**	**5.609**
Helicotylenchus	Eudorylaimus	**-0.792**	**-0.166**	**5.676**	**5.007**
Helicotylenchus	Mesodorylaimus	**-0.792**	**-0.277**	**5.676**	**4.530**
Helicotylenchus	Pungentus	**-0.792**	**0.263**	**5.676**	**5.132**
Helicotylenchus	Qudsianematidae	**-0.792**	**-0.207**	**5.676**	**4.831**
Helicotylenchus	Thornematidae	**-0.792**	**-0.470**	**5.676**	**4.530**
Helicotylenchus	Eupodes	**-0.792**	**0.005**	**5.676**	**4.451**
Helicotylenchus	Scutacarus	**-0.792**	**-0.608**	**5.676**	**3.627**
Paratylenchus	Dendrolaelaps	**-1.244**	**0.027**	**4.530**	**3.150**
Paratylenchus	Lysigamasus	**-1.244**	**0.407**	**4.530**	**3.150**
Paratylenchus	Aporcelaimellus	**-1.244**	**0.548**	**4.530**	**5.484**
Paratylenchus	Dorylaimoidea	**-1.244**	**-0.604**	**4.530**	**5.609**
Paratylenchus	Eudorylaimus	**-1.244**	**-0.166**	**4.530**	**5.007**
Paratylenchus	Mesodorylaimus	**-1.244**	**-0.277**	**4.530**	**4.530**
Paratylenchus	Pungentus	**-1.244**	**0.263**	**4.530**	**5.132**
Paratylenchus	Qudsianematidae	**-1.244**	**-0.207**	**4.530**	**4.831**
Paratylenchus	Thornematidae	**-1.244**	**-0.470**	**4.530**	**4.530**
Paratylenchus	Eupodes	**-1.244**	**0.005**	**4.530**	**4.451**
Paratylenchus	Scutacarus	**-1.244**	**-0.608**	**4.530**	**3.627**
Pratylenchus	Dendrolaelaps	**-1.226**	**0.027**	**5.132**	**3.150**
Pratylenchus	Lysigamasus	**-1.226**	**0.407**	**5.132**	**3.150**
Pratylenchus	Aporcelaimellus	**-1.226**	**0.548**	**5.132**	**5.484**
Pratylenchus	Dorylaimoidea	**-1.226**	**-0.604**	**5.132**	**5.609**
Pratylenchus	Eudorylaimus	**-1.226**	**-0.166**	**5.132**	**5.007**
Pratylenchus	Mesodorylaimus	**-1.226**	**-0.277**	**5.132**	**4.530**

Pratylenchus	Pungentus	**-1.226**	**0.263**	**5.132**	**5.132**
Pratylenchus	Qudsianematidae	**-1.226**	**-0.207**	**5.132**	**4.831**
Pratylenchus	Thornematidae	**-1.226**	**-0.470**	**5.132**	**4.530**
Pratylenchus	Eupodes	**-1.226**	**0.005**	**5.132**	**4.451**
Pratylenchus	Scutacarus	**-1.226**	**-0.608**	**5.132**	**3.627**
Trichodorus	Dendrolaelaps	**-0.744**	**0.027**	**4.530**	**3.150**
Trichodorus	Lysigamasus	**-0.744**	**0.407**	**4.530**	**3.150**
Trichodorus	Aporcelaimellus	**-0.744**	**0.548**	**4.530**	**5.484**
Trichodorus	Dorylaimoidea	**-0.744**	**-0.604**	**4.530**	**5.609**
Trichodorus	Eudorylaimus	**-0.744**	**-0.166**	**4.530**	**5.007**
Trichodorus	Mesodorylaimus	**-0.744**	**-0.277**	**4.530**	**4.530**
Trichodorus	Pungentus	**-0.744**	**0.263**	**4.530**	**5.132**
Trichodorus	Qudsianematidae	**-0.744**	**-0.207**	**4.530**	**4.831**
Trichodorus	Thornematidae	**-0.744**	**-0.470**	**4.530**	**4.530**
Trichodorus	Eupodes	**-0.744**	**0.005**	**4.530**	**4.451**
Trichodorus	Scutacarus	**-0.744**	**-0.608**	**4.530**	**3.627**
Tylenchorhynchus	Dendrolaelaps	**-0.664**	**0.027**	**4.831**	**3.150**
Tylenchorhynchus	Lysigamasus	**-0.664**	**0.407**	**4.831**	**3.150**
Tylenchorhynchus	Aporcelaimellus	**-0.664**	**0.548**	**4.831**	**5.484**
Tylenchorhynchus	Dorylaimoidea	**-0.664**	**-0.604**	**4.831**	**5.609**
Tylenchorhynchus	Eudorylaimus	**-0.664**	**-0.166**	**4.831**	**5.007**
Tylenchorhynchus	Mesodorylaimus	**-0.664**	**-0.277**	**4.831**	**4.530**
Tylenchorhynchus	Pungentus	**-0.664**	**0.263**	**4.831**	**5.132**
Tylenchorhynchus	Qudsianematidae	**-0.664**	**-0.207**	**4.831**	**4.831**
Tylenchorhynchus	Thornematidae	**-0.664**	**-0.470**	**4.831**	**4.530**
Tylenchorhynchus	Eupodes	**-0.664**	**0.005**	**4.831**	**4.451**
Tylenchorhynchus	Scutacarus	**-0.664**	**-0.608**	**4.831**	**3.627**
Achipteria	Dendrolaelaps	**0.341**	**0.027**	**3.995**	**3.150**
Achipteria	Lysigamasus	**0.341**	**0.407**	**3.995**	**3.150**
Achipteria	Aporcelaimellus	**0.341**	**0.548**	**3.995**	**5.484**
Achipteria	Dorylaimoidea	**0.341**	**-0.604**	**3.995**	**5.609**
Achipteria	Eudorylaimus	**0.341**	**-0.166**	**3.995**	**5.007**
Achipteria	Mesodorylaimus	**0.341**	**-0.277**	**3.995**	**4.530**
Achipteria	Pungentus	**0.341**	**0.263**	**3.995**	**5.132**
Achipteria	Qudsianematidae	**0.341**	**-0.207**	**3.995**	**4.831**
Achipteria	Thornematidae	**0.341**	**-0.470**	**3.995**	**4.530**
Achipteria	Eupodes	**0.341**	**0.005**	**3.995**	**4.451**
Achipteria	Scutacarus	**0.341**	**-0.608**	**3.995**	**3.627**
Penthalodidae	Dendrolaelaps	**0.246**	**0.027**	**3.150**	**3.150**
Penthalodidae	Lysigamasus	**0.246**	**0.407**	**3.150**	**3.150**
Penthalodidae	Aporcelaimellus	**0.246**	**0.548**	**3.150**	**5.484**
Penthalodidae	Dorylaimoidea	**0.246**	**-0.604**	**3.150**	**5.609**
Penthalodidae	Eudorylaimus	**0.246**	**-0.166**	**3.150**	**5.007**
Penthalodidae	Mesodorylaimus	**0.246**	**-0.277**	**3.150**	**4.530**
Penthalodidae	Pungentus	**0.246**	**0.263**	**3.150**	**5.132**
Penthalodidae	Qudsianematidae	**0.246**	**-0.207**	**3.150**	**4.831**
Penthalodidae	Thornematidae	**0.246**	**-0.470**	**3.150**	**4.530**
Penthalodidae	Eupodes	**0.246**	**0.005**	**3.150**	**4.451**
Penthalodidae	Scutacarus	**0.246**	**-0.608**	**3.150**	**3.627**
Platynothrus	Dendrolaelaps	**0.710**	**0.027**	**3.451**	**3.150**
Platynothrus	Lysigamasus	**0.710**	**0.407**	**3.451**	**3.150**

Platynothrus	Aporcelaimellus	**0.710**	**0.548**	**3.451**	**5.484**
Platynothrus	Dorylaimoidea	**0.710**	**-0.604**	**3.451**	**5.609**
Platynothrus	Eudorylaimus	**0.710**	**-0.166**	**3.451**	**5.007**
Platynothrus	Mesodorylaimus	**0.710**	**-0.277**	**3.451**	**4.530**
Platynothrus	Pungentus	**0.710**	**0.263**	**3.451**	**5.132**
Platynothrus	Qudsianematidae	**0.710**	**-0.207**	**3.451**	**4.831**
Platynothrus	Thornematidae	**0.710**	**-0.470**	**3.451**	**4.530**
Platynothrus	Eupodes	**0.710**	**0.005**	**3.451**	**4.451**
Platynothrus	Scutacarus	**0.710**	**-0.608**	**3.451**	**3.627**
Tydeidae	Dendrolaelaps	**-0.608**	**0.027**	**3.627**	**3.150**
Tydeidae	Lysigamasus	**-0.608**	**0.407**	**3.627**	**3.150**
Tydeidae	Aporcelaimellus	**-0.608**	**0.548**	**3.627**	**5.484**
Tydeidae	Dorylaimoidea	**-0.608**	**-0.604**	**3.627**	**5.609**
Tydeidae	Eudorylaimus	**-0.608**	**-0.166**	**3.627**	**5.007**
Tydeidae	Mesodorylaimus	**-0.608**	**-0.277**	**3.627**	**4.530**
Tydeidae	Pungentus	**-0.608**	**0.263**	**3.627**	**5.132**
Tydeidae	Qudsianematidae	**-0.608**	**-0.207**	**3.627**	**4.831**
Tydeidae	Thornematidae	**-0.608**	**-0.470**	**3.627**	**4.530**
Tydeidae	Eupodes	**-0.608**	**0.005**	**3.627**	**4.451**
Tydeidae	Scutacarus	**-0.608**	**-0.608**	**3.627**	**3.627**
Sminthurinus	Dendrolaelaps	**0.618**	**0.027**	**3.627**	**3.150**
Sminthurinus	Lysigamasus	**0.618**	**0.407**	**3.627**	**3.150**
Sminthurinus	Aporcelaimellus	**0.618**	**0.548**	**3.627**	**5.484**
Sminthurinus	Dorylaimoidea	**0.618**	**-0.604**	**3.627**	**5.609**
Sminthurinus	Eudorylaimus	**0.618**	**-0.166**	**3.627**	**5.007**
Sminthurinus	Mesodorylaimus	**0.618**	**-0.277**	**3.627**	**4.530**
Sminthurinus	Pungentus	**0.618**	**0.263**	**3.627**	**5.132**
Sminthurinus	Qudsianematidae	**0.618**	**-0.207**	**3.627**	**4.831**
Sminthurinus	Thornematidae	**0.618**	**-0.470**	**3.627**	**4.530**
Sminthurinus	Eupodes	**0.618**	**0.005**	**3.627**	**4.451**
Sminthurinus	Scutacarus	**0.618**	**-0.608**	**3.627**	**3.627**
Aphelenchoides	Dendrolaelaps	**-1.496**	**0.027**	**4.831**	**3.150**
Aphelenchoides	Lysigamasus	**-1.496**	**0.407**	**4.831**	**3.150**
Aphelenchoides	Aporcelaimellus	**-1.496**	**0.548**	**4.831**	**5.484**
Aphelenchoides	Dorylaimoidea	**-1.496**	**-0.604**	**4.831**	**5.609**
Aphelenchoides	Eudorylaimus	**-1.496**	**-0.166**	**4.831**	**5.007**
Aphelenchoides	Mesodorylaimus	**-1.496**	**-0.277**	**4.831**	**4.530**
Aphelenchoides	Pungentus	**-1.496**	**0.263**	**4.831**	**5.132**
Aphelenchoides	Qudsianematidae	**-1.496**	**-0.207**	**4.831**	**4.831**
Aphelenchoides	Thornematidae	**-1.496**	**-0.470**	**4.831**	**4.530**
Aphelenchoides	Eupodes	**-1.496**	**0.005**	**4.831**	**4.451**
Aphelenchoides	Scutacarus	**-1.496**	**-0.608**	**4.831**	**3.627**
Tylenchidae	Dendrolaelaps	**-1.360**	**0.027**	**6.048**	**3.150**
Tylenchidae	Lysigamasus	**-1.360**	**0.407**	**6.048**	**3.150**
Tylenchidae	Aporcelaimellus	**-1.360**	**0.548**	**6.048**	**5.484**
Tylenchidae	Dorylaimoidea	**-1.360**	**-0.604**	**6.048**	**5.609**
Tylenchidae	Eudorylaimus	**-1.360**	**-0.166**	**6.048**	**5.007**
Tylenchidae	Mesodorylaimus	**-1.360**	**-0.277**	**6.048**	**4.530**
Tylenchidae	Pungentus	**-1.360**	**0.263**	**6.048**	**5.132**
Tylenchidae	Qudsianematidae	**-1.360**	**-0.207**	**6.048**	**4.831**
Tylenchidae	Thornematidae	**-1.360**	**-0.470**	**6.048**	**4.530**

Tylenchidae	Eupodes	**-1.360**	**0.005**	**6.048**	**4.451**
Tylenchidae	Scutacarus	**-1.360**	**-0.608**	**6.048**	**3.627**
Liebstadia	Dendrolaelaps	**0.270**	**0.027**	**3.451**	**3.150**
Liebstadia	Lysigamasus	**0.270**	**0.407**	**3.451**	**3.150**
Liebstadia	Aporcelaimellus	**0.270**	**0.548**	**3.451**	**5.484**
Liebstadia	Dorylaimoidea	**0.270**	**-0.604**	**3.451**	**5.609**
Liebstadia	Eudorylaimus	**0.270**	**-0.166**	**3.451**	**5.007**
Liebstadia	Mesodorylaimus	**0.270**	**-0.277**	**3.451**	**4.530**
Liebstadia	Pungentus	**0.270**	**0.263**	**3.451**	**5.132**
Liebstadia	Qudsianematidae	**0.270**	**-0.207**	**3.451**	**4.831**
Liebstadia	Thornematidae	**0.270**	**-0.470**	**3.451**	**4.530**
Liebstadia	Eupodes	**0.270**	**0.005**	**3.451**	**4.451**
Liebstadia	Scutacarus	**0.270**	**-0.608**	**3.451**	**3.627**
Medioppia	Dendrolaelaps	**-0.235**	**0.027**	**3.752**	**3.150**
Medioppia	Lysigamasus	**-0.235**	**0.407**	**3.752**	**3.150**
Medioppia	Aporcelaimellus	**-0.235**	**0.548**	**3.752**	**5.484**
Medioppia	Dorylaimoidea	**-0.235**	**-0.604**	**3.752**	**5.609**
Medioppia	Eudorylaimus	**-0.235**	**-0.166**	**3.752**	**5.007**
Medioppia	Mesodorylaimus	**-0.235**	**-0.277**	**3.752**	**4.530**
Medioppia	Pungentus	**-0.235**	**0.263**	**3.752**	**5.132**
Medioppia	Qudsianematidae	**-0.235**	**-0.207**	**3.752**	**4.831**
Medioppia	Thornematidae	**-0.235**	**-0.470**	**3.752**	**4.530**
Medioppia	Eupodes	**-0.235**	**0.005**	**3.752**	**4.451**
Medioppia	Scutacarus	**-0.235**	**-0.608**	**3.752**	**3.627**
Microtydeus	Dendrolaelaps	**-0.863**	**0.027**	**3.150**	**3.150**
Microtydeus	Lysigamasus	**-0.863**	**0.407**	**3.150**	**3.150**
Microtydeus	Aporcelaimellus	**-0.863**	**0.548**	**3.150**	**5.484**
Microtydeus	Dorylaimoidea	**-0.863**	**-0.604**	**3.150**	**5.609**
Microtydeus	Eudorylaimus	**-0.863**	**-0.166**	**3.150**	**5.007**
Microtydeus	Mesodorylaimus	**-0.863**	**-0.277**	**3.150**	**4.530**
Microtydeus	Pungentus	**-0.863**	**0.263**	**3.150**	**5.132**
Microtydeus	Qudsianematidae	**-0.863**	**-0.207**	**3.150**	**4.831**
Microtydeus	Thornematidae	**-0.863**	**-0.470**	**3.150**	**4.530**
Microtydeus	Eupodes	**-0.863**	**0.005**	**3.150**	**4.451**
Microtydeus	Scutacarus	**-0.863**	**-0.608**	**3.150**	**3.627**
Minunthozetes	Dendrolaelaps	**-0.249**	**0.027**	**3.451**	**3.150**
Minunthozetes	Lysigamasus	**-0.249**	**0.407**	**3.451**	**3.150**
Minunthozetes	Aporcelaimellus	**-0.249**	**0.548**	**3.451**	**5.484**
Minunthozetes	Dorylaimoidea	**-0.249**	**-0.604**	**3.451**	**5.609**
Minunthozetes	Eudorylaimus	**-0.249**	**-0.166**	**3.451**	**5.007**
Minunthozetes	Mesodorylaimus	**-0.249**	**-0.277**	**3.451**	**4.530**
Minunthozetes	Pungentus	**-0.249**	**0.263**	**3.451**	**5.132**
Minunthozetes	Qudsianematidae	**-0.249**	**-0.207**	**3.451**	**4.831**
Minunthozetes	Thornematidae	**-0.249**	**-0.470**	**3.451**	**4.530**
Minunthozetes	Eupodes	**-0.249**	**0.005**	**3.451**	**4.451**
Minunthozetes	Scutacarus	**-0.249**	**-0.608**	**3.451**	**3.627**
Pygmephorus	Dendrolaelaps	**-0.376**	**0.027**	**3.451**	**3.150**
Pygmephorus	Lysigamasus	**-0.376**	**0.407**	**3.451**	**3.150**
Pygmephorus	Aporcelaimellus	**-0.376**	**0.548**	**3.451**	**5.484**
Pygmephorus	Dorylaimoidea	**-0.376**	**-0.604**	**3.451**	**5.609**
Pygmephorus	Eudorylaimus	**-0.376**	**-0.166**	**3.451**	**5.007**

Pygmephorus	Mesodorylaimus	**-0.376**	**-0.277**	**3.451**	**4.530**
Pygmephorus	Pungentus	**-0.376**	**0.263**	**3.451**	**5.132**
Pygmephorus	Qudsianematidae	**-0.376**	**-0.207**	**3.451**	**4.831**
Pygmephorus	Thornematidae	**-0.376**	**-0.470**	**3.451**	**4.530**
Pygmephorus	Eupodes	**-0.376**	**0.005**	**3.451**	**4.451**
Pygmephorus	Scutacarus	**-0.376**	**-0.608**	**3.451**	**3.627**
Tectocepheus	Dendrolaelaps	**-0.220**	**0.027**	**3.752**	**3.150**
Tectocepheus	Lysigamasus	**-0.220**	**0.407**	**3.752**	**3.150**
Tectocepheus	Aporcelaimellus	**-0.220**	**0.548**	**3.752**	**5.484**
Tectocepheus	Dorylaimoidea	**-0.220**	**-0.604**	**3.752**	**5.609**
Tectocepheus	Eudorylaimus	**-0.220**	**-0.166**	**3.752**	**5.007**
Tectocepheus	Mesodorylaimus	**-0.220**	**-0.277**	**3.752**	**4.530**
Tectocepheus	Pungentus	**-0.220**	**0.263**	**3.752**	**5.132**
Tectocepheus	Qudsianematidae	**-0.220**	**-0.207**	**3.752**	**4.831**
Tectocepheus	Thornematidae	**-0.220**	**-0.470**	**3.752**	**4.530**
Tectocepheus	Eupodes	**-0.220**	**0.005**	**3.752**	**4.451**
Tectocepheus	Scutacarus	**-0.220**	**-0.608**	**3.752**	**3.627**
Brachystomella	Dendrolaelaps	**0.977**	**0.027**	**3.451**	**3.150**
Brachystomella	Lysigamasus	**0.977**	**0.407**	**3.451**	**3.150**
Brachystomella	Aporcelaimellus	**0.977**	**0.548**	**3.451**	**5.484**
Brachystomella	Dorylaimoidea	**0.977**	**-0.604**	**3.451**	**5.609**
Brachystomella	Eudorylaimus	**0.977**	**-0.166**	**3.451**	**5.007**
Brachystomella	Mesodorylaimus	**0.977**	**-0.277**	**3.451**	**4.530**
Brachystomella	Pungentus	**0.977**	**0.263**	**3.451**	**5.132**
Brachystomella	Qudsianematidae	**0.977**	**-0.207**	**3.451**	**4.831**
Brachystomella	Thornematidae	**0.977**	**-0.470**	**3.451**	**4.530**
Brachystomella	Eupodes	**0.977**	**0.005**	**3.451**	**4.451**
Brachystomella	Scutacarus	**0.977**	**-0.608**	**3.451**	**3.627**
Isotoma	Dendrolaelaps	**1.898**	**0.027**	**3.150**	**3.150**
Isotoma	Lysigamasus	**1.898**	**0.407**	**3.150**	**3.150**
Isotoma	Aporcelaimellus	**1.898**	**0.548**	**3.150**	**5.484**
Isotoma	Dorylaimoidea	**1.898**	**-0.604**	**3.150**	**5.609**
Isotoma	Eudorylaimus	**1.898**	**-0.166**	**3.150**	**5.007**
Isotoma	Mesodorylaimus	**1.898**	**-0.277**	**3.150**	**4.530**
Isotoma	Pungentus	**1.898**	**0.263**	**3.150**	**5.132**
Isotoma	Qudsianematidae	**1.898**	**-0.207**	**3.150**	**4.831**
Isotoma	Thornematidae	**1.898**	**-0.470**	**3.150**	**4.530**
Isotoma	Eupodes	**1.898**	**0.005**	**3.150**	**4.451**
Isotoma	Scutacarus	**1.898**	**-0.608**	**3.150**	**3.627**
Lepidocyrtus	Dendrolaelaps	**1.231**	**0.027**	**3.928**	**3.150**
Lepidocyrtus	Lysigamasus	**1.231**	**0.407**	**3.928**	**3.150**
Lepidocyrtus	Aporcelaimellus	**1.231**	**0.548**	**3.928**	**5.484**
Lepidocyrtus	Dorylaimoidea	**1.231**	**-0.604**	**3.928**	**5.609**
Lepidocyrtus	Eudorylaimus	**1.231**	**-0.166**	**3.928**	**5.007**
Lepidocyrtus	Mesodorylaimus	**1.231**	**-0.277**	**3.928**	**4.530**
Lepidocyrtus	Pungentus	**1.231**	**0.263**	**3.928**	**5.132**
Lepidocyrtus	Qudsianematidae	**1.231**	**-0.207**	**3.928**	**4.831**
Lepidocyrtus	Thornematidae	**1.231**	**-0.470**	**3.928**	**4.530**
Lepidocyrtus	Eupodes	**1.231**	**0.005**	**3.928**	**4.451**
Lepidocyrtus	Scutacarus	**1.231**	**-0.608**	**3.928**	**3.627**
Parisotoma	Dendrolaelaps	**0.722**	**0.027**	**3.150**	**3.150**

Parisotoma	Lysigamasus	**0.722**	**0.407**	**3.150**	**3.150**
Parisotoma	Aporcelaimellus	**0.722**	**0.548**	**3.150**	**5.484**
Parisotoma	Dorylaimoidea	**0.722**	**-0.604**	**3.150**	**5.609**
Parisotoma	Eudorylaimus	**0.722**	**-0.166**	**3.150**	**5.007**
Parisotoma	Mesodorylaimus	**0.722**	**-0.277**	**3.150**	**4.530**
Parisotoma	Pungentus	**0.722**	**0.263**	**3.150**	**5.132**
Parisotoma	Qudsianematidae	**0.722**	**-0.207**	**3.150**	**4.831**
Parisotoma	Thornematidae	**0.722**	**-0.470**	**3.150**	**4.530**
Parisotoma	Eupodes	**0.722**	**0.005**	**3.150**	**4.451**
Parisotoma	Scutacarus	**0.722**	**-0.608**	**3.150**	**3.627**
Achaeta	Dendrolaelaps	**0.706**	**0.027**	**2.549**	**3.150**
Achaeta	Lysigamasus	**0.706**	**0.407**	**2.549**	**3.150**
Achaeta	Aporcelaimellus	**0.706**	**0.548**	**2.549**	**5.484**
Achaeta	Dorylaimoidea	**0.706**	**-0.604**	**2.549**	**5.609**
Achaeta	Eudorylaimus	**0.706**	**-0.166**	**2.549**	**5.007**
Achaeta	Mesodorylaimus	**0.706**	**-0.277**	**2.549**	**4.530**
Achaeta	Pungentus	**0.706**	**0.263**	**2.549**	**5.132**
Achaeta	Qudsianematidae	**0.706**	**-0.207**	**2.549**	**4.831**
Achaeta	Thornematidae	**0.706**	**-0.470**	**2.549**	**4.530**
Achaeta	Eupodes	**0.706**	**0.005**	**2.549**	**4.451**
Achaeta	Scutacarus	**0.706**	**-0.608**	**2.549**	**3.627**
Cognettia	Dendrolaelaps	**1.302**	**0.027**	**3.443**	**3.150**
Cognettia	Lysigamasus	**1.302**	**0.407**	**3.443**	**3.150**
Cognettia	Aporcelaimellus	**1.302**	**0.548**	**3.443**	**5.484**
Cognettia	Dorylaimoidea	**1.302**	**-0.604**	**3.443**	**5.609**
Cognettia	Eudorylaimus	**1.302**	**-0.166**	**3.443**	**5.007**
Cognettia	Mesodorylaimus	**1.302**	**-0.277**	**3.443**	**4.530**
Cognettia	Pungentus	**1.302**	**0.263**	**3.443**	**5.132**
Cognettia	Qudsianematidae	**1.302**	**-0.207**	**3.443**	**4.831**
Cognettia	Thornematidae	**1.302**	**-0.470**	**3.443**	**4.530**
Cognettia	Eupodes	**1.302**	**0.005**	**3.443**	**4.451**
Cognettia	Scutacarus	**1.302**	**-0.608**	**3.443**	**3.627**
Fridericia	Dendrolaelaps	**2.149**	**0.027**	**3.373**	**3.150**
Fridericia	Lysigamasus	**2.149**	**0.407**	**3.373**	**3.150**
Fridericia	Aporcelaimellus	**2.149**	**0.548**	**3.373**	**5.484**
Fridericia	Dorylaimoidea	**2.149**	**-0.604**	**3.373**	**5.609**
Fridericia	Eudorylaimus	**2.149**	**-0.166**	**3.373**	**5.007**
Fridericia	Mesodorylaimus	**2.149**	**-0.277**	**3.373**	**4.530**
Fridericia	Pungentus	**2.149**	**0.263**	**3.373**	**5.132**
Fridericia	Qudsianematidae	**2.149**	**-0.207**	**3.373**	**4.831**
Fridericia	Thornematidae	**2.149**	**-0.470**	**3.373**	**4.530**
Fridericia	Eupodes	**2.149**	**0.005**	**3.373**	**4.451**
Fridericia	Scutacarus	**2.149**	**-0.608**	**3.373**	**3.627**
Acrobeloides	Dendrolaelaps	**-1.171**	**0.027**	**5.571**	**3.150**
Acrobeloides	Lysigamasus	**-1.171**	**0.407**	**5.571**	**3.150**
Acrobeloides	Aporcelaimellus	**-1.171**	**0.548**	**5.571**	**5.484**
Acrobeloides	Dorylaimoidea	**-1.171**	**-0.604**	**5.571**	**5.609**
Acrobeloides	Eudorylaimus	**-1.171**	**-0.166**	**5.571**	**5.007**
Acrobeloides	Mesodorylaimus	**-1.171**	**-0.277**	**5.571**	**4.530**
Acrobeloides	Pungentus	**-1.171**	**0.263**	**5.571**	**5.132**
Acrobeloides	Qudsianematidae	**-1.171**	**-0.207**	**5.571**	**4.831**

Acrobeloides	Thornematidae	**-1.171**	**-0.470**	**5.571**	**4.530**
Acrobeloides	Eupodes	**-1.171**	**0.005**	**5.571**	**4.451**
Acrobeloides	Scutacarus	**-1.171**	**-0.608**	**5.571**	**3.627**
Cephalobidae	Dendrolaelaps	**-1.055**	**0.027**	**4.831**	**3.150**
Cephalobidae	Lysigamasus	**-1.055**	**0.407**	**4.831**	**3.150**
Cephalobidae	Aporcelaimellus	**-1.055**	**0.548**	**4.831**	**5.484**
Cephalobidae	Dorylaimoidea	**-1.055**	**-0.604**	**4.831**	**5.609**
Cephalobidae	Eudorylaimus	**-1.055**	**-0.166**	**4.831**	**5.007**
Cephalobidae	Mesodorylaimus	**-1.055**	**-0.277**	**4.831**	**4.530**
Cephalobidae	Pungentus	**-1.055**	**0.263**	**4.831**	**5.132**
Cephalobidae	Qudsianematidae	**-1.055**	**-0.207**	**4.831**	**4.831**
Cephalobidae	Thornematidae	**-1.055**	**-0.470**	**4.831**	**4.530**
Cephalobidae	Eupodes	**-1.055**	**0.005**	**4.831**	**4.451**
Cephalobidae	Scutacarus	**-1.055**	**-0.608**	**4.831**	**3.627**
Eucephalobus	Dendrolaelaps	**-0.855**	**0.027**	**5.433**	**3.150**
Eucephalobus	Lysigamasus	**-0.855**	**0.407**	**5.433**	**3.150**
Eucephalobus	Aporcelaimellus	**-0.855**	**0.548**	**5.433**	**5.484**
Eucephalobus	Dorylaimoidea	**-0.855**	**-0.604**	**5.433**	**5.609**
Eucephalobus	Eudorylaimus	**-0.855**	**-0.166**	**5.433**	**5.007**
Eucephalobus	Mesodorylaimus	**-0.855**	**-0.277**	**5.433**	**4.530**
Eucephalobus	Pungentus	**-0.855**	**0.263**	**5.433**	**5.132**
Eucephalobus	Qudsianematidae	**-0.855**	**-0.207**	**5.433**	**4.831**
Eucephalobus	Thornematidae	**-0.855**	**-0.470**	**5.433**	**4.530**
Eucephalobus	Eupodes	**-0.855**	**0.005**	**5.433**	**4.451**
Eucephalobus	Scutacarus	**-0.855**	**-0.608**	**5.433**	**3.627**
Panagrolaimus	Dendrolaelaps	**-0.945**	**0.027**	**5.007**	**3.150**
Panagrolaimus	Lysigamasus	**-0.945**	**0.407**	**5.007**	**3.150**
Panagrolaimus	Aporcelaimellus	**-0.945**	**0.548**	**5.007**	**5.484**
Panagrolaimus	Dorylaimoidea	**-0.945**	**-0.604**	**5.007**	**5.609**
Panagrolaimus	Eudorylaimus	**-0.945**	**-0.166**	**5.007**	**5.007**
Panagrolaimus	Mesodorylaimus	**-0.945**	**-0.277**	**5.007**	**4.530**
Panagrolaimus	Pungentus	**-0.945**	**0.263**	**5.007**	**5.132**
Panagrolaimus	Qudsianematidae	**-0.945**	**-0.207**	**5.007**	**4.831**
Panagrolaimus	Thornematidae	**-0.945**	**-0.470**	**5.007**	**4.530**
Panagrolaimus	Eupodes	**-0.945**	**0.005**	**5.007**	**4.451**
Panagrolaimus	Scutacarus	**-0.945**	**-0.608**	**5.007**	**3.627**
Plectus	Dendrolaelaps	**-0.583**	**0.027**	**5.734**	**3.150**
Plectus	Lysigamasus	**-0.583**	**0.407**	**5.734**	**3.150**
Plectus	Aporcelaimellus	**-0.583**	**0.548**	**5.734**	**5.484**
Plectus	Dorylaimoidea	**-0.583**	**-0.604**	**5.734**	**5.609**
Plectus	Eudorylaimus	**-0.583**	**-0.166**	**5.734**	**5.007**
Plectus	Mesodorylaimus	**-0.583**	**-0.277**	**5.734**	**4.530**
Plectus	Pungentus	**-0.583**	**0.263**	**5.734**	**5.132**
Plectus	Qudsianematidae	**-0.583**	**-0.207**	**5.734**	**4.831**
Plectus	Thornematidae	**-0.583**	**-0.470**	**5.734**	**4.530**
Plectus	Eupodes	**-0.583**	**0.005**	**5.734**	**4.451**
Plectus	Scutacarus	**-0.583**	**-0.608**	**5.734**	**3.627**
Prismatolaimus	Dendrolaelaps	**-1.280**	**0.027**	**4.530**	**3.150**
Prismatolaimus	Lysigamasus	**-1.280**	**0.407**	**4.530**	**3.150**
Prismatolaimus	Aporcelaimellus	**-1.280**	**0.548**	**4.530**	**5.484**
Prismatolaimus	Dorylaimoidea	**-1.280**	**-0.604**	**4.530**	**5.609**

Prismatolaimus	Eudorylaimus	**-1.280**	**-0.166**	**4.530**	**5.007**
Prismatolaimus	Mesodorylaimus	**-1.280**	**-0.277**	**4.530**	**4.530**
Prismatolaimus	Pungentus	**-1.280**	**0.263**	**4.530**	**5.132**
Prismatolaimus	Qudsianematidae	**-1.280**	**-0.207**	**4.530**	**4.831**
Prismatolaimus	Thornematidae	**-1.280**	**-0.470**	**4.530**	**4.530**
Prismatolaimus	Eupodes	**-1.280**	**0.005**	**4.530**	**4.451**
Prismatolaimus	Scutacarus	**-1.280**	**-0.608**	**4.530**	**3.627**
Rhabditidae	Dendrolaelaps	**-0.692**	**0.027**	**4.530**	**3.150**
Rhabditidae	Lysigamasus	**-0.692**	**0.407**	**4.530**	**3.150**
Rhabditidae	Aporcelaimellus	**-0.692**	**0.548**	**4.530**	**5.484**
Rhabditidae	Dorylaimoidea	**-0.692**	**-0.604**	**4.530**	**5.609**
Rhabditidae	Eudorylaimus	**-0.692**	**-0.166**	**4.530**	**5.007**
Rhabditidae	Mesodorylaimus	**-0.692**	**-0.277**	**4.530**	**4.530**
Rhabditidae	Pungentus	**-0.692**	**0.263**	**4.530**	**5.132**
Rhabditidae	Qudsianematidae	**-0.692**	**-0.207**	**4.530**	**4.831**
Rhabditidae	Thornematidae	**-0.692**	**-0.470**	**4.530**	**4.530**
Rhabditidae	Eupodes	**-0.692**	**0.005**	**4.530**	**4.451**
Rhabditidae	Scutacarus	**-0.692**	**-0.608**	**4.530**	**3.627**
Teratocephalus	Dendrolaelaps	**-1.630**	**0.027**	**5.132**	**3.150**
Teratocephalus	Lysigamasus	**-1.630**	**0.407**	**5.132**	**3.150**
Teratocephalus	Aporcelaimellus	**-1.630**	**0.548**	**5.132**	**5.484**
Teratocephalus	Dorylaimoidea	**-1.630**	**-0.604**	**5.132**	**5.609**
Teratocephalus	Eudorylaimus	**-1.630**	**-0.166**	**5.132**	**5.007**
Teratocephalus	Mesodorylaimus	**-1.630**	**-0.277**	**5.132**	**4.530**
Teratocephalus	Pungentus	**-1.630**	**0.263**	**5.132**	**5.132**
Teratocephalus	Qudsianematidae	**-1.630**	**-0.207**	**5.132**	**4.831**
Teratocephalus	Thornematidae	**-1.630**	**-0.470**	**5.132**	**4.530**
Teratocephalus	Eupodes	**-1.630**	**0.005**	**5.132**	**4.451**
Teratocephalus	Scutacarus	**-1.630**	**-0.608**	**5.132**	**3.627**
Wilsonema	Dendrolaelaps	**-0.562**	**0.027**	**4.530**	**3.150**
Wilsonema	Lysigamasus	**-0.562**	**0.407**	**4.530**	**3.150**
Wilsonema	Aporcelaimellus	**-0.562**	**0.548**	**4.530**	**5.484**
Wilsonema	Dorylaimoidea	**-0.562**	**-0.604**	**4.530**	**5.609**
Wilsonema	Eudorylaimus	**-0.562**	**-0.166**	**4.530**	**5.007**
Wilsonema	Mesodorylaimus	**-0.562**	**-0.277**	**4.530**	**4.530**
Wilsonema	Pungentus	**-0.562**	**0.263**	**4.530**	**5.132**
Wilsonema	Qudsianematidae	**-0.562**	**-0.207**	**4.530**	**4.831**
Wilsonema	Thornematidae	**-0.562**	**-0.470**	**4.530**	**4.530**
Wilsonema	Eupodes	**-0.562**	**0.005**	**4.530**	**4.451**
Wilsonema	Scutacarus	**-0.562**	**-0.608**	**4.530**	**3.627**
Eupelops	Dendrolaelaps	**0.326**	**0.027**	**3.150**	**3.150**
Eupelops	Lysigamasus	**0.326**	**0.407**	**3.150**	**3.150**
Eupelops	Eupodes	**0.326**	**0.005**	**3.150**	**4.451**
Eupelops	Scutacarus	**0.326**	**-0.608**	**3.150**	**3.627**
Histiostoma	Dendrolaelaps	**-0.805**	**0.027**	**3.150**	**3.150**
Histiostoma	Lysigamasus	**-0.805**	**0.407**	**3.150**	**3.150**
Histiostoma	Eupodes	**-0.805**	**0.005**	**3.150**	**4.451**
Histiostoma	Scutacarus	**-0.805**	**-0.608**	**3.150**	**3.627**
Enchytraeus	Dendrolaelaps	**1.012**	**0.027**	**2.975**	**3.150**
Enchytraeus	Lysigamasus	**1.012**	**0.407**	**2.975**	**3.150**
Enchytraeus	Aporcelaimellus	**1.012**	**0.548**	**2.975**	**5.484**

Enchytraeus	Dorylaimoidea	**1.012**	**-0.604**	**2.975**	**5.609**
Enchytraeus	Eudorylaimus	**1.012**	**-0.166**	**2.975**	**5.007**
Enchytraeus	Mesodorylaimus	**1.012**	**-0.277**	**2.975**	**4.530**
Enchytraeus	Pungentus	**1.012**	**0.263**	**2.975**	**5.132**
Enchytraeus	Qudsianematidae	**1.012**	**-0.207**	**2.975**	**4.831**
Enchytraeus	Thornematidae	**1.012**	**-0.470**	**2.975**	**4.530**
Enchytraeus	Eupodes	**1.012**	**0.005**	**2.975**	**4.451**
Enchytraeus	Scutacarus	**1.012**	**-0.608**	**2.975**	**3.627**
Eubacteria	Acrobeloides	**-6.557**	**-1.171**	**12.430**	**5.571**
Eubacteria	Cephalobidae	**-6.557**	**-1.055**	**12.430**	**4.831**
Eubacteria	Eucephalobus	**-6.557**	**-0.855**	**12.430**	**5.433**
Eubacteria	Panagrolaimus	**-6.557**	**-0.945**	**12.430**	**5.007**
Eubacteria	Plectus	**-6.557**	**-0.583**	**12.430**	**5.734**
Eubacteria	Prismatolaimus	**-6.557**	**-1.280**	**12.430**	**4.530**
Eubacteria	Rhabditidae	**-6.557**	**-0.692**	**12.430**	**4.530**
Eubacteria	Teratocephalus	**-6.557**	**-1.630**	**12.430**	**5.132**
Eubacteria	Wilsonema	**-6.557**	**-0.562**	**12.430**	**4.530**
Eubacteria	Eupelops	**-6.557**	**0.326**	**12.430**	**3.150**
Eubacteria	Histiostoma	**-6.557**	**-0.805**	**12.430**	**3.150**
Eubacteria	Enchytraeus	**-6.557**	**1.012**	**12.430**	**2.975**
Eubacteria	Buchholzia	**-6.557**	**0.855**	**12.430**	**2.725**
Eubacteria	Henlea	**-6.557**	**1.067**	**12.430**	**2.725**
Eubacteria	Marionina	**-6.557**	**0.849**	**12.430**	**3.860**
Buchholzia	Dendrolaelaps	**0.855**	**0.027**	**2.725**	**3.150**
Buchholzia	Lysigamasus	**0.855**	**0.407**	**2.725**	**3.150**
Buchholzia	Aporcelaimellus	**0.855**	**0.548**	**2.725**	**5.484**
Buchholzia	Dorylaimoidea	**0.855**	**-0.604**	**2.725**	**5.609**
Buchholzia	Eudorylaimus	**0.855**	**-0.166**	**2.725**	**5.007**
Buchholzia	Mesodorylaimus	**0.855**	**-0.277**	**2.725**	**4.530**
Buchholzia	Pungentus	**0.855**	**0.263**	**2.725**	**5.132**
Buchholzia	Qudsianematidae	**0.855**	**-0.207**	**2.725**	**4.831**
Buchholzia	Thornematidae	**0.855**	**-0.470**	**2.725**	**4.530**
Buchholzia	Eupodes	**0.855**	**0.005**	**2.725**	**4.451**
Buchholzia	Scutacarus	**0.855**	**-0.608**	**2.725**	**3.627**
Henlea	Dendrolaelaps	**1.067**	**0.027**	**2.725**	**3.150**
Henlea	Lysigamasus	**1.067**	**0.407**	**2.725**	**3.150**
Henlea	Aporcelaimellus	**1.067**	**0.548**	**2.725**	**5.484**
Henlea	Dorylaimoidea	**1.067**	**-0.604**	**2.725**	**5.609**
Henlea	Eudorylaimus	**1.067**	**-0.166**	**2.725**	**5.007**
Henlea	Mesodorylaimus	**1.067**	**-0.277**	**2.725**	**4.530**
Henlea	Pungentus	**1.067**	**0.263**	**2.725**	**5.132**
Henlea	Qudsianematidae	**1.067**	**-0.207**	**2.725**	**4.831**
Henlea	Thornematidae	**1.067**	**-0.470**	**2.725**	**4.530**
Henlea	Eupodes	**1.067**	**0.005**	**2.725**	**4.451**
Henlea	Scutacarus	**1.067**	**-0.608**	**2.725**	**3.627**
Marionina	Dendrolaelaps	**0.849**	**0.027**	**3.860**	**3.150**
Marionina	Lysigamasus	**0.849**	**0.407**	**3.860**	**3.150**
Marionina	Aporcelaimellus	**0.849**	**0.548**	**3.860**	**5.484**
Marionina	Dorylaimoidea	**0.849**	**-0.604**	**3.860**	**5.609**
Marionina	Eudorylaimus	**0.849**	**-0.166**	**3.860**	**5.007**
Marionina	Mesodorylaimus	**0.849**	**-0.277**	**3.860**	**4.530**

Marionina	Pungentus	**0.849**	**0.263**	**3.860**	**5.132**
Marionina	Qudsianematidae	**0.849**	**-0.207**	**3.860**	**4.831**
Marionina	Thornematidae	**0.849**	**-0.470**	**3.860**	**4.530**
Marionina	Eupodes	**0.849**	**0.005**	**3.860**	**4.451**
Marionina	Scutacarus	**0.849**	**-0.608**	**3.860**	**3.627**
Hyphae and hair roots	Aglenchus	**6.040**	**-1.053**	**0.000**	**5.375**
Hyphae and hair roots	Dolichodoridae	**6.040**	**-0.885**	**0.000**	**5.229**
Hyphae and hair roots	Helicotylenchus	**6.040**	**-0.792**	**0.000**	**5.676**
Hyphae and hair roots	Paratylenchus	**6.040**	**-1.244**	**0.000**	**4.530**
Hyphae and hair roots	Pratylenchus	**6.040**	**-1.226**	**0.000**	**5.132**
Hyphae and hair roots	Trichodorus	**6.040**	**-0.744**	**0.000**	**4.530**
Hyphae and hair roots	Tylenchorhynchus	**6.040**	**-0.664**	**0.000**	**4.831**
Hyphae and hair roots	Achipteria	**6.040**	**0.341**	**0.000**	**3.995**
Hyphae and hair roots	Penthalodidae	**6.040**	**0.246**	**0.000**	**3.150**
Hyphae and hair roots	Platynothrus	**6.040**	**0.710**	**0.000**	**3.451**
Hyphae and hair roots	Tydeidae	**6.040**	**-0.608**	**0.000**	**3.627**
Hyphae and hair roots	Sminthurinus	**6.040**	**0.618**	**0.000**	**3.627**
Hyphae and hair roots	Aphelenchoides	**6.040**	**-1.496**	**0.000**	**4.831**
Hyphae and hair roots	Tylenchidae	**6.040**	**-1.360**	**0.000**	**6.048**
Hyphae and hair roots	Liebstadia	**6.040**	**0.270**	**0.000**	**3.451**
Hyphae and hair roots	Medioppia	**6.040**	**-0.235**	**0.000**	**3.752**
Hyphae and hair roots	Microtydeus	**6.040**	**-0.863**	**0.000**	**3.150**
Hyphae and hair roots	Minunthozetes	**6.040**	**-0.249**	**0.000**	**3.451**
Hyphae and hair roots	Pygmephorus	**6.040**	**-0.376**	**0.000**	**3.451**
Hyphae and hair roots	Tectocepheus	**6.040**	**-0.220**	**0.000**	**3.752**
Hyphae and hair roots	Brachystomella	**6.040**	**0.977**	**0.000**	**3.451**
Hyphae and hair roots	Isotoma	**6.040**	**1.898**	**0.000**	**3.150**
Hyphae and hair roots	Lepidocyrtus	**6.040**	**1.231**	**0.000**	**3.928**
Hyphae and hair roots	Parisotoma	**6.040**	**0.722**	**0.000**	**3.150**
Hyphae and hair roots	Achaeta	**6.040**	**0.706**	**0.000**	**2.549**
Hyphae and hair roots	Cognettia	**6.040**	**1.302**	**0.000**	**3.443**
Hyphae and hair roots	Fridericia	**6.040**	**2.149**	**0.000**	**3.373**
Hyphae and hair roots	Aporcelaimellus	**6.040**	**0.548**	**0.000**	**5.484**
Hyphae and hair roots	Dorylaimoidea	**6.040**	**-0.604**	**0.000**	**5.609**
Hyphae and hair roots	Eudorylaimus	**6.040**	**-0.166**	**0.000**	**5.007**
Hyphae and hair roots	Mesodorylaimus	**6.040**	**-0.277**	**0.000**	**4.530**
Hyphae and hair roots	Pungentus	**6.040**	**0.263**	**0.000**	**5.132**
Hyphae and hair roots	Qudsianematidae	**6.040**	**-0.207**	**0.000**	**4.831**
Hyphae and hair roots	Thornematidae	**6.040**	**-0.470**	**0.000**	**4.530**
Hyphae and hair roots	Eupodes	**6.040**	**0.005**	**0.000**	**4.451**
Hyphae and hair roots	Scutacarus	**6.040**	**-0.608**	**0.000**	**3.627**
Dendrolaelaps	Aporcelaimellus	**0.027**	**0.548**	**3.150**	**5.484**
Dendrolaelaps	Dorylaimoidea	**0.027**	**-0.604**	**3.150**	**5.609**
Dendrolaelaps	Eudorylaimus	**0.027**	**-0.166**	**3.150**	**5.007**
Dendrolaelaps	Mesodorylaimus	**0.027**	**-0.277**	**3.150**	**4.530**
Dendrolaelaps	Pungentus	**0.027**	**0.263**	**3.150**	**5.132**
Dendrolaelaps	Qudsianematidae	**0.027**	**-0.207**	**3.150**	**4.831**
Dendrolaelaps	Thornematidae	**0.027**	**-0.470**	**3.150**	**4.530**
Dendrolaelaps	Eupodes	**0.027**	**0.005**	**3.150**	**4.451**
Dendrolaelaps	Scutacarus	**0.027**	**-0.608**	**3.150**	**3.627**
Lysigamasus	Aporcelaimellus	**0.407**	**0.548**	**3.150**	**5.484**

Lysigamasus	Dorylaimoidea	**0.407**	**-0.604**	**3.150**	**5.609**
Lysigamasus	Eudorylaimus	**0.407**	**-0.166**	**3.150**	**5.007**
Lysigamasus	Mesodorylaimus	**0.407**	**-0.277**	**3.150**	**4.530**
Lysigamasus	Pungentus	**0.407**	**0.263**	**3.150**	**5.132**
Lysigamasus	Qudsianematidae	**0.407**	**-0.207**	**3.150**	**4.831**
Lysigamasus	Thornematidae	**0.407**	**-0.470**	**3.150**	**4.530**
Lysigamasus	Eupodes	**0.407**	**0.005**	**3.150**	**4.451**
Lysigamasus	Scutacarus	**0.407**	**-0.608**	**3.150**	**3.627**
Aporcelaimellus	Dendrolaelaps	**0.548**	**0.027**	**5.484**	**3.150**
Aporcelaimellus	Lysigamasus	**0.548**	**0.407**	**5.484**	**3.150**
Aporcelaimellus	Aporcelaimellus	**0.548**	**0.548**	**5.484**	**5.484**
Aporcelaimellus	Dorylaimoidea	**0.548**	**-0.604**	**5.484**	**5.609**
Aporcelaimellus	Eudorylaimus	**0.548**	**-0.166**	**5.484**	**5.007**
Aporcelaimellus	Mesodorylaimus	**0.548**	**-0.277**	**5.484**	**4.530**
Aporcelaimellus	Pungentus	**0.548**	**0.263**	**5.484**	**5.132**
Aporcelaimellus	Qudsianematidae	**0.548**	**-0.207**	**5.484**	**4.831**
Aporcelaimellus	Thornematidae	**0.548**	**-0.470**	**5.484**	**4.530**
Aporcelaimellus	Eupodes	**0.548**	**0.005**	**5.484**	**4.451**
Aporcelaimellus	Scutacarus	**0.548**	**-0.608**	**5.484**	**3.627**
Dorylaimoidea	Dendrolaelaps	**-0.604**	**0.027**	**5.609**	**3.150**
Dorylaimoidea	Lysigamasus	**-0.604**	**0.407**	**5.609**	**3.150**
Dorylaimoidea	Aporcelaimellus	**-0.604**	**0.548**	**5.609**	**5.484**
Dorylaimoidea	Dorylaimoidea	**-0.604**	**-0.604**	**5.609**	**5.609**
Dorylaimoidea	Eudorylaimus	**-0.604**	**-0.166**	**5.609**	**5.007**
Dorylaimoidea	Mesodorylaimus	**-0.604**	**-0.277**	**5.609**	**4.530**
Dorylaimoidea	Pungentus	**-0.604**	**0.263**	**5.609**	**5.132**
Dorylaimoidea	Qudsianematidae	**-0.604**	**-0.207**	**5.609**	**4.831**
Dorylaimoidea	Thornematidae	**-0.604**	**-0.470**	**5.609**	**4.530**
Dorylaimoidea	Eupodes	**-0.604**	**0.005**	**5.609**	**4.451**
Dorylaimoidea	Scutacarus	**-0.604**	**-0.608**	**5.609**	**3.627**
Eudorylaimus	Dendrolaelaps	**-0.166**	**0.027**	**5.007**	**3.150**
Eudorylaimus	Lysigamasus	**-0.166**	**0.407**	**5.007**	**3.150**
Eudorylaimus	Aporcelaimellus	**-0.166**	**0.548**	**5.007**	**5.484**
Eudorylaimus	Dorylaimoidea	**-0.166**	**-0.604**	**5.007**	**5.609**
Eudorylaimus	Eudorylaimus	**-0.166**	**-0.166**	**5.007**	**5.007**
Eudorylaimus	Mesodorylaimus	**-0.166**	**-0.277**	**5.007**	**4.530**
Eudorylaimus	Pungentus	**-0.166**	**0.263**	**5.007**	**5.132**
Eudorylaimus	Qudsianematidae	**-0.166**	**-0.207**	**5.007**	**4.831**
Eudorylaimus	Thornematidae	**-0.166**	**-0.470**	**5.007**	**4.530**
Eudorylaimus	Eupodes	**-0.166**	**0.005**	**5.007**	**4.451**
Eudorylaimus	Scutacarus	**-0.166**	**-0.608**	**5.007**	**3.627**
Mesodorylaimus	Dendrolaelaps	**-0.277**	**0.027**	**4.530**	**3.150**
Mesodorylaimus	Lysigamasus	**-0.277**	**0.407**	**4.530**	**3.150**
Mesodorylaimus	Aporcelaimellus	**-0.277**	**0.548**	**4.530**	**5.484**
Mesodorylaimus	Dorylaimoidea	**-0.277**	**-0.604**	**4.530**	**5.609**
Mesodorylaimus	Eudorylaimus	**-0.277**	**-0.166**	**4.530**	**5.007**
Mesodorylaimus	Mesodorylaimus	**-0.277**	**-0.277**	**4.530**	**4.530**
Mesodorylaimus	Pungentus	**-0.277**	**0.263**	**4.530**	**5.132**
Mesodorylaimus	Qudsianematidae	**-0.277**	**-0.207**	**4.530**	**4.831**
Mesodorylaimus	Thornematidae	**-0.277**	**-0.470**	**4.530**	**4.530**
Mesodorylaimus	Eupodes	**-0.277**	**0.005**	**4.530**	**4.451**

Mesodorylaimus	Scutacarus	**-0.277**	**-0.608**	**4.530**	**3.627**
Pungentus	Dendrolaelaps	**0.263**	**0.027**	**5.132**	**3.150**
Pungentus	Lysigamasus	**0.263**	**0.407**	**5.132**	**3.150**
Pungentus	Aporcelaimellus	**0.263**	**0.548**	**5.132**	**5.484**
Pungentus	Dorylaimoidea	**0.263**	**-0.604**	**5.132**	**5.609**
Pungentus	Eudorylaimus	**0.263**	**-0.166**	**5.132**	**5.007**
Pungentus	Mesodorylaimus	**0.263**	**-0.277**	**5.132**	**4.530**
Pungentus	Pungentus	**0.263**	**0.263**	**5.132**	**5.132**
Pungentus	Qudsianematidae	**0.263**	**-0.207**	**5.132**	**4.831**
Pungentus	Thornematidae	**0.263**	**-0.470**	**5.132**	**4.530**
Pungentus	Eupodes	**0.263**	**0.005**	**5.132**	**4.451**
Pungentus	Scutacarus	**0.263**	**-0.608**	**5.132**	**3.627**
Qudsianematidae	Dendrolaelaps	**-0.207**	**0.027**	**4.831**	**3.150**
Qudsianematidae	Lysigamasus	**-0.207**	**0.407**	**4.831**	**3.150**
Qudsianematidae	Aporcelaimellus	**-0.207**	**0.548**	**4.831**	**5.484**
Qudsianematidae	Dorylaimoidea	**-0.207**	**-0.604**	**4.831**	**5.609**
Qudsianematidae	Eudorylaimus	**-0.207**	**-0.166**	**4.831**	**5.007**
Qudsianematidae	Mesodorylaimus	**-0.207**	**-0.277**	**4.831**	**4.530**
Qudsianematidae	Pungentus	**-0.207**	**0.263**	**4.831**	**5.132**
Qudsianematidae	Qudsianematidae	**-0.207**	**-0.207**	**4.831**	**4.831**
Qudsianematidae	Thornematidae	**-0.207**	**-0.470**	**4.831**	**4.530**
Qudsianematidae	Eupodes	**-0.207**	**0.005**	**4.831**	**4.451**
Qudsianematidae	Scutacarus	**-0.207**	**-0.608**	**4.831**	**3.627**
Thornematidae	Dendrolaelaps	**-0.470**	**0.027**	**4.530**	**3.150**
Thornematidae	Lysigamasus	**-0.470**	**0.407**	**4.530**	**3.150**
Thornematidae	Aporcelaimellus	**-0.470**	**0.548**	**4.530**	**5.484**
Thornematidae	Dorylaimoidea	**-0.470**	**-0.604**	**4.530**	**5.609**
Thornematidae	Eudorylaimus	**-0.470**	**-0.166**	**4.530**	**5.007**
Thornematidae	Mesodorylaimus	**-0.470**	**-0.277**	**4.530**	**4.530**
Thornematidae	Pungentus	**-0.470**	**0.263**	**4.530**	**5.132**
Thornematidae	Qudsianematidae	**-0.470**	**-0.207**	**4.530**	**4.831**
Thornematidae	Thornematidae	**-0.470**	**-0.470**	**4.530**	**4.530**
Thornematidae	Eupodes	**-0.470**	**0.005**	**4.530**	**4.451**
Thornematidae	Scutacarus	**-0.470**	**-0.608**	**4.530**	**3.627**
Eupodes	Dendrolaelaps	**0.005**	**0.027**	**4.451**	**3.150**
Eupodes	Lysigamasus	**0.005**	**0.407**	**4.451**	**3.150**
Eupodes	Aporcelaimellus	**0.005**	**0.548**	**4.451**	**5.484**
Eupodes	Dorylaimoidea	**0.005**	**-0.604**	**4.451**	**5.609**
Eupodes	Eudorylaimus	**0.005**	**-0.166**	**4.451**	**5.007**
Eupodes	Mesodorylaimus	**0.005**	**-0.277**	**4.451**	**4.530**
Eupodes	Pungentus	**0.005**	**0.263**	**4.451**	**5.132**
Eupodes	Qudsianematidae	**0.005**	**-0.207**	**4.451**	**4.831**
Eupodes	Thornematidae	**0.005**	**-0.470**	**4.451**	**4.530**
Eupodes	Eupodes	**0.005**	**0.005**	**4.451**	**4.451**
Eupodes	Scutacarus	**0.005**	**-0.608**	**4.451**	**3.627**
Scutacarus	Dendrolaelaps	**-0.608**	**0.027**	**3.627**	**3.150**
Scutacarus	Lysigamasus	**-0.608**	**0.407**	**3.627**	**3.150**
Scutacarus	Aporcelaimellus	**-0.608**	**0.548**	**3.627**	**5.484**
Scutacarus	Dorylaimoidea	**-0.608**	**-0.604**	**3.627**	**5.609**
Scutacarus	Eudorylaimus	**-0.608**	**-0.166**	**3.627**	**5.007**
Scutacarus	Mesodorylaimus	**-0.608**	**-0.277**	**3.627**	**4.530**

Scutacarus	Pungentus	**-0.608**	**0.263**	**3.627**	**5.132**
Scutacarus	Qudsianematidae	**-0.608**	**-0.207**	**3.627**	**4.831**
Scutacarus	Thornematidae	**-0.608**	**-0.470**	**3.627**	**4.530**
Scutacarus	Eupodes	**-0.608**	**0.005**	**3.627**	**4.451**
Scutacarus	Scutacarus	**-0.608**	**-0.608**	**3.627**	**3.627**

Resource	Consumer	Mres	Mconsumer	Nres	Nconsumer
Aglenchus	Arctoseius	**-1.053**	**-0.152**	**5.864**	**3.572**
Aglenchus	Dendrolaelaps	**-1.053**	**0.027**	**5.864**	**3.095**
Aglenchus	Hypoaspis	**-1.053**	**0.334**	**5.864**	**3.095**
Aglenchus	Lysigamasus	**-1.053**	**0.407**	**5.864**	**3.396**
Aglenchus	Uropoda	**-1.053**	**0.481**	**5.864**	**3.095**
Aglenchus	Aporcelaimellus	**-1.053**	**0.548**	**5.864**	**5.386**
Aglenchus	Dorylaimoidea	**-1.053**	**-0.604**	**5.864**	**5.650**
Aglenchus	Epidorylaimus	**-1.053**	**0.199**	**5.864**	**4.608**
Aglenchus	Prodorylaimus	**-1.053**	**-0.836**	**5.864**	**4.608**
Aglenchus	Pungentus	**-1.053**	**0.263**	**5.864**	**4.608**
Aglenchus	Thornematidae	**-1.053**	**-0.470**	**5.864**	**5.085**
Aglenchus	Eupodes	**-1.053**	**0.005**	**5.864**	**3.697**
Aglenchus	Protodinychus	**-1.053**	**0.549**	**5.864**	**3.095**
Aglenchus	Scutacarus	**-1.053**	**-0.608**	**5.864**	**3.396**
Aglenchus	Stigmaeidae	**-1.053**	**0.229**	**5.864**	**3.095**
Dolichodoridae	Arctoseius	**-0.885**	**-0.152**	**4.608**	**3.572**
Dolichodoridae	Dendrolaelaps	**-0.885**	**0.027**	**4.608**	**3.095**
Dolichodoridae	Hypoaspis	**-0.885**	**0.334**	**4.608**	**3.095**
Dolichodoridae	Lysigamasus	**-0.885**	**0.407**	**4.608**	**3.396**
Dolichodoridae	Uropoda	**-0.885**	**0.481**	**4.608**	**3.095**
Dolichodoridae	Aporcelaimellus	**-0.885**	**0.548**	**4.608**	**5.386**
Dolichodoridae	Dorylaimoidea	**-0.885**	**-0.604**	**4.608**	**5.650**
Dolichodoridae	Epidorylaimus	**-0.885**	**0.199**	**4.608**	**4.608**
Dolichodoridae	Prodorylaimus	**-0.885**	**-0.836**	**4.608**	**4.608**
Dolichodoridae	Pungentus	**-0.885**	**0.263**	**4.608**	**4.608**
Dolichodoridae	Thornematidae	**-0.885**	**-0.470**	**4.608**	**5.085**
Dolichodoridae	Eupodes	**-0.885**	**0.005**	**4.608**	**3.697**
Dolichodoridae	Protodinychus	**-0.885**	**0.549**	**4.608**	**3.095**
Dolichodoridae	Scutacarus	**-0.885**	**-0.608**	**4.608**	**3.396**
Dolichodoridae	Stigmaeidae	**-0.885**	**0.229**	**4.608**	**3.095**
Filenchus	Arctoseius	**-1.033**	**-0.152**	**4.909**	**3.572**
Filenchus	Dendrolaelaps	**-1.033**	**0.027**	**4.909**	**3.095**
Filenchus	Hypoaspis	**-1.033**	**0.334**	**4.909**	**3.095**
Filenchus	Lysigamasus	**-1.033**	**0.407**	**4.909**	**3.396**
Filenchus	Uropoda	**-1.033**	**0.481**	**4.909**	**3.095**
Filenchus	Aporcelaimellus	**-1.033**	**0.548**	**4.909**	**5.386**
Filenchus	Dorylaimoidea	**-1.033**	**-0.604**	**4.909**	**5.650**
Filenchus	Epidorylaimus	**-1.033**	**0.199**	**4.909**	**4.608**
Filenchus	Prodorylaimus	**-1.033**	**-0.836**	**4.909**	**4.608**
Filenchus	Pungentus	**-1.033**	**0.263**	**4.909**	**4.608**
Filenchus	Thornematidae	**-1.033**	**-0.470**	**4.909**	**5.085**
Filenchus	Eupodes	**-1.033**	**0.005**	**4.909**	**3.697**
Filenchus	Protodinychus	**-1.033**	**0.549**	**4.909**	**3.095**
Filenchus	Scutacarus	**-1.033**	**-0.608**	**4.909**	**3.396**
Filenchus	Stigmaeidae	**-1.033**	**0.229**	**4.909**	**3.095**
Helicotylenchus	Arctoseius	**-0.792**	**-0.152**	**5.453**	**3.572**
Helicotylenchus	Dendrolaelaps	**-0.792**	**0.027**	**5.453**	**3.095**
Helicotylenchus	Hypoaspis	**-0.792**	**0.334**	**5.453**	**3.095**
Helicotylenchus	Lysigamasus	**-0.792**	**0.407**	**5.453**	**3.396**
Helicotylenchus	Uropoda	**-0.792**	**0.481**	**5.453**	**3.095**

Helicotylenchus	Aporcelaimellus	**-0.792**	**0.548**	**5.453**	**5.386**
Helicotylenchus	Dorylaimoidea	**-0.792**	**-0.604**	**5.453**	**5.650**
Helicotylenchus	Epidorylaimus	**-0.792**	**0.199**	**5.453**	**4.608**
Helicotylenchus	Prodorylaimus	**-0.792**	**-0.836**	**5.453**	**4.608**
Helicotylenchus	Pungentus	**-0.792**	**0.263**	**5.453**	**4.608**
Helicotylenchus	Thornematidae	**-0.792**	**-0.470**	**5.453**	**5.085**
Helicotylenchus	Eupodes	**-0.792**	**0.005**	**5.453**	**3.697**
Helicotylenchus	Protodinychus	**-0.792**	**0.549**	**5.453**	**3.095**
Helicotylenchus	Scutacarus	**-0.792**	**-0.608**	**5.453**	**3.396**
Helicotylenchus	Stigmaeidae	**-0.792**	**0.229**	**5.453**	**3.095**
Malenchus	Arctoseius	**-1.330**	**-0.152**	**5.085**	**3.572**
Malenchus	Dendrolaelaps	**-1.330**	**0.027**	**5.085**	**3.095**
Malenchus	Hypoaspis	**-1.330**	**0.334**	**5.085**	**3.095**
Malenchus	Lysigamasus	**-1.330**	**0.407**	**5.085**	**3.396**
Malenchus	Uropoda	**-1.330**	**0.481**	**5.085**	**3.095**
Malenchus	Aporcelaimellus	**-1.330**	**0.548**	**5.085**	**5.386**
Malenchus	Dorylaimoidea	**-1.330**	**-0.604**	**5.085**	**5.650**
Malenchus	Epidorylaimus	**-1.330**	**0.199**	**5.085**	**4.608**
Malenchus	Prodorylaimus	**-1.330**	**-0.836**	**5.085**	**4.608**
Malenchus	Pungentus	**-1.330**	**0.263**	**5.085**	**4.608**
Malenchus	Thornematidae	**-1.330**	**-0.470**	**5.085**	**5.085**
Malenchus	Eupodes	**-1.330**	**0.005**	**5.085**	**3.697**
Malenchus	Protodinychus	**-1.330**	**0.549**	**5.085**	**3.095**
Malenchus	Scutacarus	**-1.330**	**-0.608**	**5.085**	**3.396**
Malenchus	Stigmaeidae	**-1.330**	**0.229**	**5.085**	**3.095**
Meloidogyne	Arctoseius	**-1.287**	**-0.152**	**4.608**	**3.572**
Meloidogyne	Dendrolaelaps	**-1.287**	**0.027**	**4.608**	**3.095**
Meloidogyne	Hypoaspis	**-1.287**	**0.334**	**4.608**	**3.095**
Meloidogyne	Lysigamasus	**-1.287**	**0.407**	**4.608**	**3.396**
Meloidogyne	Uropoda	**-1.287**	**0.481**	**4.608**	**3.095**
Meloidogyne	Aporcelaimellus	**-1.287**	**0.548**	**4.608**	**5.386**
Meloidogyne	Dorylaimoidea	**-1.287**	**-0.604**	**4.608**	**5.650**
Meloidogyne	Epidorylaimus	**-1.287**	**0.199**	**4.608**	**4.608**
Meloidogyne	Prodorylaimus	**-1.287**	**-0.836**	**4.608**	**4.608**
Meloidogyne	Pungentus	**-1.287**	**0.263**	**4.608**	**4.608**
Meloidogyne	Thornematidae	**-1.287**	**-0.470**	**4.608**	**5.085**
Meloidogyne	Eupodes	**-1.287**	**0.005**	**4.608**	**3.697**
Meloidogyne	Protodinychus	**-1.287**	**0.549**	**4.608**	**3.095**
Meloidogyne	Scutacarus	**-1.287**	**-0.608**	**4.608**	**3.396**
Meloidogyne	Stigmaeidae	**-1.287**	**0.229**	**4.608**	**3.095**
Paratylenchus	Arctoseius	**-1.244**	**-0.152**	**5.085**	**3.572**
Paratylenchus	Dendrolaelaps	**-1.244**	**0.027**	**5.085**	**3.095**
Paratylenchus	Hypoaspis	**-1.244**	**0.334**	**5.085**	**3.095**
Paratylenchus	Lysigamasus	**-1.244**	**0.407**	**5.085**	**3.396**
Paratylenchus	Uropoda	**-1.244**	**0.481**	**5.085**	**3.095**
Paratylenchus	Aporcelaimellus	**-1.244**	**0.548**	**5.085**	**5.386**
Paratylenchus	Dorylaimoidea	**-1.244**	**-0.604**	**5.085**	**5.650**
Paratylenchus	Epidorylaimus	**-1.244**	**0.199**	**5.085**	**4.608**
Paratylenchus	Prodorylaimus	**-1.244**	**-0.836**	**5.085**	**4.608**
Paratylenchus	Pungentus	**-1.244**	**0.263**	**5.085**	**4.608**
Paratylenchus	Thornematidae	**-1.244**	**-0.470**	**5.085**	**5.085**

Paratylenchus	Eupodes	**-1.244**	**0.005**	**5.085**	**3.697**
Paratylenchus	Protodinychus	**-1.244**	**0.549**	**5.085**	**3.095**
Paratylenchus	Scutacarus	**-1.244**	**-0.608**	**5.085**	**3.396**
Paratylenchus	Stigmaeidae	**-1.244**	**0.229**	**5.085**	**3.095**
Pratylenchus	Arctoseius	**-1.226**	**-0.152**	**4.608**	**3.572**
Pratylenchus	Dendrolaelaps	**-1.226**	**0.027**	**4.608**	**3.095**
Pratylenchus	Hypoaspis	**-1.226**	**0.334**	**4.608**	**3.095**
Pratylenchus	Lysigamasus	**-1.226**	**0.407**	**4.608**	**3.396**
Pratylenchus	Uropoda	**-1.226**	**0.481**	**4.608**	**3.095**
Pratylenchus	Aporcelaimellus	**-1.226**	**0.548**	**4.608**	**5.386**
Pratylenchus	Dorylaimoidea	**-1.226**	**-0.604**	**4.608**	**5.650**
Pratylenchus	Epidorylaimus	**-1.226**	**0.199**	**4.608**	**4.608**
Pratylenchus	Prodorylaimus	**-1.226**	**-0.836**	**4.608**	**4.608**
Pratylenchus	Pungentus	**-1.226**	**0.263**	**4.608**	**4.608**
Pratylenchus	Thornematidae	**-1.226**	**-0.470**	**4.608**	**5.085**
Pratylenchus	Eupodes	**-1.226**	**0.005**	**4.608**	**3.697**
Pratylenchus	Protodinychus	**-1.226**	**0.549**	**4.608**	**3.095**
Pratylenchus	Scutacarus	**-1.226**	**-0.608**	**4.608**	**3.396**
Pratylenchus	Stigmaeidae	**-1.226**	**0.229**	**4.608**	**3.095**
Trichodorus	Arctoseius	**-0.744**	**-0.152**	**4.608**	**3.572**
Trichodorus	Dendrolaelaps	**-0.744**	**0.027**	**4.608**	**3.095**
Trichodorus	Hypoaspis	**-0.744**	**0.334**	**4.608**	**3.095**
Trichodorus	Lysigamasus	**-0.744**	**0.407**	**4.608**	**3.396**
Trichodorus	Uropoda	**-0.744**	**0.481**	**4.608**	**3.095**
Trichodorus	Aporcelaimellus	**-0.744**	**0.548**	**4.608**	**5.386**
Trichodorus	Dorylaimoidea	**-0.744**	**-0.604**	**4.608**	**5.650**
Trichodorus	Epidorylaimus	**-0.744**	**0.199**	**4.608**	**4.608**
Trichodorus	Prodorylaimus	**-0.744**	**-0.836**	**4.608**	**4.608**
Trichodorus	Pungentus	**-0.744**	**0.263**	**4.608**	**4.608**
Trichodorus	Thornematidae	**-0.744**	**-0.470**	**4.608**	**5.085**
Trichodorus	Eupodes	**-0.744**	**0.005**	**4.608**	**3.697**
Trichodorus	Protodinychus	**-0.744**	**0.549**	**4.608**	**3.095**
Trichodorus	Scutacarus	**-0.744**	**-0.608**	**4.608**	**3.396**
Trichodorus	Stigmaeidae	**-0.744**	**0.229**	**4.608**	**3.095**
Tylenchorhynchus	Arctoseius	**-0.664**	**-0.152**	**5.386**	**3.572**
Tylenchorhynchus	Dendrolaelaps	**-0.664**	**0.027**	**5.386**	**3.095**
Tylenchorhynchus	Hypoaspis	**-0.664**	**0.334**	**5.386**	**3.095**
Tylenchorhynchus	Lysigamasus	**-0.664**	**0.407**	**5.386**	**3.396**
Tylenchorhynchus	Uropoda	**-0.664**	**0.481**	**5.386**	**3.095**
Tylenchorhynchus	Aporcelaimellus	**-0.664**	**0.548**	**5.386**	**5.386**
Tylenchorhynchus	Dorylaimoidea	**-0.664**	**-0.604**	**5.386**	**5.650**
Tylenchorhynchus	Epidorylaimus	**-0.664**	**0.199**	**5.386**	**4.608**
Tylenchorhynchus	Prodorylaimus	**-0.664**	**-0.836**	**5.386**	**4.608**
Tylenchorhynchus	Pungentus	**-0.664**	**0.263**	**5.386**	**4.608**
Tylenchorhynchus	Thornematidae	**-0.664**	**-0.470**	**5.386**	**5.085**
Tylenchorhynchus	Eupodes	**-0.664**	**0.005**	**5.386**	**3.697**
Tylenchorhynchus	Protodinychus	**-0.664**	**0.549**	**5.386**	**3.095**
Tylenchorhynchus	Scutacarus	**-0.664**	**-0.608**	**5.386**	**3.396**
Tylenchorhynchus	Stigmaeidae	**-0.664**	**0.229**	**5.386**	**3.095**
Achipteria	Arctoseius	**0.341**	**-0.152**	**4.299**	**3.572**
Achipteria	Dendrolaelaps	**0.341**	**0.027**	**4.299**	**3.095**

Achipteria	Hypoaspis	**0.341**	**0.334**	**4.299**	**3.095**
Achipteria	Lysigamasus	**0.341**	**0.407**	**4.299**	**3.396**
Achipteria	Uropoda	**0.341**	**0.481**	**4.299**	**3.095**
Achipteria	Aporcelaimellus	**0.341**	**0.548**	**4.299**	**5.386**
Achipteria	Dorylaimoidea	**0.341**	**-0.604**	**4.299**	**5.650**
Achipteria	Epidorylaimus	**0.341**	**0.199**	**4.299**	**4.608**
Achipteria	Prodorylaimus	**0.341**	**-0.836**	**4.299**	**4.608**
Achipteria	Pungentus	**0.341**	**0.263**	**4.299**	**4.608**
Achipteria	Thornematidae	**0.341**	**-0.470**	**4.299**	**5.085**
Achipteria	Eupodes	**0.341**	**0.005**	**4.299**	**3.697**
Achipteria	Protodinychus	**0.341**	**0.549**	**4.299**	**3.095**
Achipteria	Scutacarus	**0.341**	**-0.608**	**4.299**	**3.396**
Achipteria	Stigmaeidae	**0.341**	**0.229**	**4.299**	**3.095**
Pachygnatidae	Arctoseius	**-0.113**	**-0.152**	**3.095**	**3.572**
Pachygnatidae	Dendrolaelaps	**-0.113**	**0.027**	**3.095**	**3.095**
Pachygnatidae	Hypoaspis	**-0.113**	**0.334**	**3.095**	**3.095**
Pachygnatidae	Lysigamasus	**-0.113**	**0.407**	**3.095**	**3.396**
Pachygnatidae	Uropoda	**-0.113**	**0.481**	**3.095**	**3.095**
Pachygnatidae	Aporcelaimellus	**-0.113**	**0.548**	**3.095**	**5.386**
Pachygnatidae	Dorylaimoidea	**-0.113**	**-0.604**	**3.095**	**5.650**
Pachygnatidae	Epidorylaimus	**-0.113**	**0.199**	**3.095**	**4.608**
Pachygnatidae	Prodorylaimus	**-0.113**	**-0.836**	**3.095**	**4.608**
Pachygnatidae	Pungentus	**-0.113**	**0.263**	**3.095**	**4.608**
Pachygnatidae	Thornematidae	**-0.113**	**-0.470**	**3.095**	**5.085**
Pachygnatidae	Eupodes	**-0.113**	**0.005**	**3.095**	**3.697**
Pachygnatidae	Protodinychus	**-0.113**	**0.549**	**3.095**	**3.095**
Pachygnatidae	Scutacarus	**-0.113**	**-0.608**	**3.095**	**3.396**
Pachygnatidae	Stigmaeidae	**-0.113**	**0.229**	**3.095**	**3.095**
Platynothrus	Arctoseius	**0.710**	**-0.152**	**3.697**	**3.572**
Platynothrus	Dendrolaelaps	**0.710**	**0.027**	**3.697**	**3.095**
Platynothrus	Hypoaspis	**0.710**	**0.334**	**3.697**	**3.095**
Platynothrus	Lysigamasus	**0.710**	**0.407**	**3.697**	**3.396**
Platynothrus	Uropoda	**0.710**	**0.481**	**3.697**	**3.095**
Platynothrus	Aporcelaimellus	**0.710**	**0.548**	**3.697**	**5.386**
Platynothrus	Dorylaimoidea	**0.710**	**-0.604**	**3.697**	**5.650**
Platynothrus	Epidorylaimus	**0.710**	**0.199**	**3.697**	**4.608**
Platynothrus	Prodorylaimus	**0.710**	**-0.836**	**3.697**	**4.608**
Platynothrus	Pungentus	**0.710**	**0.263**	**3.697**	**4.608**
Platynothrus	Thornematidae	**0.710**	**-0.470**	**3.697**	**5.085**
Platynothrus	Eupodes	**0.710**	**0.005**	**3.697**	**3.697**
Platynothrus	Protodinychus	**0.710**	**0.549**	**3.697**	**3.095**
Platynothrus	Scutacarus	**0.710**	**-0.608**	**3.697**	**3.396**
Platynothrus	Stigmaeidae	**0.710**	**0.229**	**3.697**	**3.095**
Tydeidae	Arctoseius	**-0.608**	**-0.152**	**3.396**	**3.572**
Tydeidae	Dendrolaelaps	**-0.608**	**0.027**	**3.396**	**3.095**
Tydeidae	Hypoaspis	**-0.608**	**0.334**	**3.396**	**3.095**
Tydeidae	Lysigamasus	**-0.608**	**0.407**	**3.396**	**3.396**
Tydeidae	Uropoda	**-0.608**	**0.481**	**3.396**	**3.095**
Tydeidae	Aporcelaimellus	**-0.608**	**0.548**	**3.396**	**5.386**
Tydeidae	Dorylaimoidea	**-0.608**	**-0.604**	**3.396**	**5.650**
Tydeidae	Epidorylaimus	**-0.608**	**0.199**	**3.396**	**4.608**

Tydeidae	Prodorylaimus	**-0.608**	**-0.836**	**3.396**	**4.608**
Tydeidae	Pungentus	**-0.608**	**0.263**	**3.396**	**4.608**
Tydeidae	Thornematidae	**-0.608**	**-0.470**	**3.396**	**5.085**
Tydeidae	Eupodes	**-0.608**	**0.005**	**3.396**	**3.697**
Tydeidae	Protodinychus	**-0.608**	**0.549**	**3.396**	**3.095**
Tydeidae	Scutacarus	**-0.608**	**-0.608**	**3.396**	**3.396**
Tydeidae	Stigmaeidae	**-0.608**	**0.229**	**3.396**	**3.095**
Sphaeridia	Arctoseius	**0.202**	**-0.152**	**3.095**	**3.572**
Sphaeridia	Dendrolaelaps	**0.202**	**0.027**	**3.095**	**3.095**
Sphaeridia	Hypoaspis	**0.202**	**0.334**	**3.095**	**3.095**
Sphaeridia	Lysigamasus	**0.202**	**0.407**	**3.095**	**3.396**
Sphaeridia	Uropoda	**0.202**	**0.481**	**3.095**	**3.095**
Sphaeridia	Aporcelaimellus	**0.202**	**0.548**	**3.095**	**5.386**
Sphaeridia	Dorylaimoidea	**0.202**	**-0.604**	**3.095**	**5.650**
Sphaeridia	Epidorylaimus	**0.202**	**0.199**	**3.095**	**4.608**
Sphaeridia	Prodorylaimus	**0.202**	**-0.836**	**3.095**	**4.608**
Sphaeridia	Pungentus	**0.202**	**0.263**	**3.095**	**4.608**
Sphaeridia	Thornematidae	**0.202**	**-0.470**	**3.095**	**5.085**
Sphaeridia	Eupodes	**0.202**	**0.005**	**3.095**	**3.697**
Sphaeridia	Protodinychus	**0.202**	**0.549**	**3.095**	**3.095**
Sphaeridia	Scutacarus	**0.202**	**-0.608**	**3.095**	**3.396**
Sphaeridia	Stigmaeidae	**0.202**	**0.229**	**3.095**	**3.095**
Aphelenchoides	Arctoseius	**-1.496**	**-0.152**	**5.386**	**3.572**
Aphelenchoides	Dendrolaelaps	**-1.496**	**0.027**	**5.386**	**3.095**
Aphelenchoides	Hypoaspis	**-1.496**	**0.334**	**5.386**	**3.095**
Aphelenchoides	Lysigamasus	**-1.496**	**0.407**	**5.386**	**3.396**
Aphelenchoides	Uropoda	**-1.496**	**0.481**	**5.386**	**3.095**
Aphelenchoides	Aporcelaimellus	**-1.496**	**0.548**	**5.386**	**5.386**
Aphelenchoides	Dorylaimoidea	**-1.496**	**-0.604**	**5.386**	**5.650**
Aphelenchoides	Epidorylaimus	**-1.496**	**0.199**	**5.386**	**4.608**
Aphelenchoides	Prodorylaimus	**-1.496**	**-0.836**	**5.386**	**4.608**
Aphelenchoides	Pungentus	**-1.496**	**0.263**	**5.386**	**4.608**
Aphelenchoides	Thornematidae	**-1.496**	**-0.470**	**5.386**	**5.085**
Aphelenchoides	Eupodes	**-1.496**	**0.005**	**5.386**	**3.697**
Aphelenchoides	Protodinychus	**-1.496**	**0.549**	**5.386**	**3.095**
Aphelenchoides	Scutacarus	**-1.496**	**-0.608**	**5.386**	**3.396**
Aphelenchoides	Stigmaeidae	**-1.496**	**0.229**	**5.386**	**3.095**
Aphelenchus	Arctoseius	**-1.129**	**-0.152**	**4.909**	**3.572**
Aphelenchus	Dendrolaelaps	**-1.129**	**0.027**	**4.909**	**3.095**
Aphelenchus	Hypoaspis	**-1.129**	**0.334**	**4.909**	**3.095**
Aphelenchus	Lysigamasus	**-1.129**	**0.407**	**4.909**	**3.396**
Aphelenchus	Uropoda	**-1.129**	**0.481**	**4.909**	**3.095**
Aphelenchus	Aporcelaimellus	**-1.129**	**0.548**	**4.909**	**5.386**
Aphelenchus	Dorylaimoidea	**-1.129**	**-0.604**	**4.909**	**5.650**
Aphelenchus	Epidorylaimus	**-1.129**	**0.199**	**4.909**	**4.608**
Aphelenchus	Prodorylaimus	**-1.129**	**-0.836**	**4.909**	**4.608**
Aphelenchus	Pungentus	**-1.129**	**0.263**	**4.909**	**4.608**
Aphelenchus	Thornematidae	**-1.129**	**-0.470**	**4.909**	**5.085**
Aphelenchus	Eupodes	**-1.129**	**0.005**	**4.909**	**3.697**
Aphelenchus	Protodinychus	**-1.129**	**0.549**	**4.909**	**3.095**
Aphelenchus	Scutacarus	**-1.129**	**-0.608**	**4.909**	**3.396**

Aphelenchus	Stigmaeidae	**-1.129**	**0.229**	**4.909**	**3.095**
Tylenchidae	Arctoseius	**-1.360**	**-0.152**	**6.071**	**3.572**
Tylenchidae	Dendrolaelaps	**-1.360**	**0.027**	**6.071**	**3.095**
Tylenchidae	Hypoaspis	**-1.360**	**0.334**	**6.071**	**3.095**
Tylenchidae	Lysigamasus	**-1.360**	**0.407**	**6.071**	**3.396**
Tylenchidae	Uropoda	**-1.360**	**0.481**	**6.071**	**3.095**
Tylenchidae	Aporcelaimellus	**-1.360**	**0.548**	**6.071**	**5.386**
Tylenchidae	Dorylaimoidea	**-1.360**	**-0.604**	**6.071**	**5.650**
Tylenchidae	Epidorylaimus	**-1.360**	**0.199**	**6.071**	**4.608**
Tylenchidae	Prodorylaimus	**-1.360**	**-0.836**	**6.071**	**4.608**
Tylenchidae	Pungentus	**-1.360**	**0.263**	**6.071**	**4.608**
Tylenchidae	Thornematidae	**-1.360**	**-0.470**	**6.071**	**5.085**
Tylenchidae	Eupodes	**-1.360**	**0.005**	**6.071**	**3.697**
Tylenchidae	Protodinychus	**-1.360**	**0.549**	**6.071**	**3.095**
Tylenchidae	Scutacarus	**-1.360**	**-0.608**	**6.071**	**3.396**
Tylenchidae	Stigmaeidae	**-1.360**	**0.229**	**6.071**	**3.095**
Liebstadia	Arctoseius	**0.270**	**-0.152**	**3.095**	**3.572**
Liebstadia	Dendrolaelaps	**0.270**	**0.027**	**3.095**	**3.095**
Liebstadia	Hypoaspis	**0.270**	**0.334**	**3.095**	**3.095**
Liebstadia	Lysigamasus	**0.270**	**0.407**	**3.095**	**3.396**
Liebstadia	Uropoda	**0.270**	**0.481**	**3.095**	**3.095**
Liebstadia	Aporcelaimellus	**0.270**	**0.548**	**3.095**	**5.386**
Liebstadia	Dorylaimoidea	**0.270**	**-0.604**	**3.095**	**5.650**
Liebstadia	Epidorylaimus	**0.270**	**0.199**	**3.095**	**4.608**
Liebstadia	Prodorylaimus	**0.270**	**-0.836**	**3.095**	**4.608**
Liebstadia	Pungentus	**0.270**	**0.263**	**3.095**	**4.608**
Liebstadia	Thornematidae	**0.270**	**-0.470**	**3.095**	**5.085**
Liebstadia	Eupodes	**0.270**	**0.005**	**3.095**	**3.697**
Liebstadia	Protodinychus	**0.270**	**0.549**	**3.095**	**3.095**
Liebstadia	Scutacarus	**0.270**	**-0.608**	**3.095**	**3.396**
Liebstadia	Stigmaeidae	**0.270**	**0.229**	**3.095**	**3.095**
Pygmephorus	Arctoseius	**-0.376**	**-0.152**	**3.095**	**3.572**
Pygmephorus	Dendrolaelaps	**-0.376**	**0.027**	**3.095**	**3.095**
Pygmephorus	Hypoaspis	**-0.376**	**0.334**	**3.095**	**3.095**
Pygmephorus	Lysigamasus	**-0.376**	**0.407**	**3.095**	**3.396**
Pygmephorus	Uropoda	**-0.376**	**0.481**	**3.095**	**3.095**
Pygmephorus	Aporcelaimellus	**-0.376**	**0.548**	**3.095**	**5.386**
Pygmephorus	Dorylaimoidea	**-0.376**	**-0.604**	**3.095**	**5.650**
Pygmephorus	Epidorylaimus	**-0.376**	**0.199**	**3.095**	**4.608**
Pygmephorus	Prodorylaimus	**-0.376**	**-0.836**	**3.095**	**4.608**
Pygmephorus	Pungentus	**-0.376**	**0.263**	**3.095**	**4.608**
Pygmephorus	Thornematidae	**-0.376**	**-0.470**	**3.095**	**5.085**
Pygmephorus	Eupodes	**-0.376**	**0.005**	**3.095**	**3.697**
Pygmephorus	Protodinychus	**-0.376**	**0.549**	**3.095**	**3.095**
Pygmephorus	Scutacarus	**-0.376**	**-0.608**	**3.095**	**3.396**
Pygmephorus	Stigmaeidae	**-0.376**	**0.229**	**3.095**	**3.095**
Ceratophysella	Arctoseius	**1.335**	**-0.152**	**3.095**	**3.572**
Ceratophysella	Dendrolaelaps	**1.335**	**0.027**	**3.095**	**3.095**
Ceratophysella	Hypoaspis	**1.335**	**0.334**	**3.095**	**3.095**
Ceratophysella	Lysigamasus	**1.335**	**0.407**	**3.095**	**3.396**
Ceratophysella	Uropoda	**1.335**	**0.481**	**3.095**	**3.095**

Ceratophysella	Aporcelaimellus	**1.335**	**0.548**	**3.095**	**5.386**
Ceratophysella	Dorylaimoidea	**1.335**	**-0.604**	**3.095**	**5.650**
Ceratophysella	Epidorylaimus	**1.335**	**0.199**	**3.095**	**4.608**
Ceratophysella	Prodorylaimus	**1.335**	**-0.836**	**3.095**	**4.608**
Ceratophysella	Pungentus	**1.335**	**0.263**	**3.095**	**4.608**
Ceratophysella	Thornematidae	**1.335**	**-0.470**	**3.095**	**5.085**
Ceratophysella	Eupodes	**1.335**	**0.005**	**3.095**	**3.697**
Ceratophysella	Protodinychus	**1.335**	**0.549**	**3.095**	**3.095**
Ceratophysella	Scutacarus	**1.335**	**-0.608**	**3.095**	**3.396**
Ceratophysella	Stigmaeidae	**1.335**	**0.229**	**3.095**	**3.095**
Folsomia	Arctoseius	**0.900**	**-0.152**	**3.095**	**3.572**
Folsomia	Dendrolaelaps	**0.900**	**0.027**	**3.095**	**3.095**
Folsomia	Hypoaspis	**0.900**	**0.334**	**3.095**	**3.095**
Folsomia	Lysigamasus	**0.900**	**0.407**	**3.095**	**3.396**
Folsomia	Uropoda	**0.900**	**0.481**	**3.095**	**3.095**
Folsomia	Aporcelaimellus	**0.900**	**0.548**	**3.095**	**5.386**
Folsomia	Dorylaimoidea	**0.900**	**-0.604**	**3.095**	**5.650**
Folsomia	Epidorylaimus	**0.900**	**0.199**	**3.095**	**4.608**
Folsomia	Prodorylaimus	**0.900**	**-0.836**	**3.095**	**4.608**
Folsomia	Pungentus	**0.900**	**0.263**	**3.095**	**4.608**
Folsomia	Thornematidae	**0.900**	**-0.470**	**3.095**	**5.085**
Folsomia	Eupodes	**0.900**	**0.005**	**3.095**	**3.697**
Folsomia	Protodinychus	**0.900**	**0.549**	**3.095**	**3.095**
Folsomia	Scutacarus	**0.900**	**-0.608**	**3.095**	**3.396**
Folsomia	Stigmaeidae	**0.900**	**0.229**	**3.095**	**3.095**
Isotoma	Arctoseius	**1.898**	**-0.152**	**4.049**	**3.572**
Isotoma	Dendrolaelaps	**1.898**	**0.027**	**4.049**	**3.095**
Isotoma	Hypoaspis	**1.898**	**0.334**	**4.049**	**3.095**
Isotoma	Lysigamasus	**1.898**	**0.407**	**4.049**	**3.396**
Isotoma	Uropoda	**1.898**	**0.481**	**4.049**	**3.095**
Isotoma	Aporcelaimellus	**1.898**	**0.548**	**4.049**	**5.386**
Isotoma	Dorylaimoidea	**1.898**	**-0.604**	**4.049**	**5.650**
Isotoma	Epidorylaimus	**1.898**	**0.199**	**4.049**	**4.608**
Isotoma	Prodorylaimus	**1.898**	**-0.836**	**4.049**	**4.608**
Isotoma	Pungentus	**1.898**	**0.263**	**4.049**	**4.608**
Isotoma	Thornematidae	**1.898**	**-0.470**	**4.049**	**5.085**
Isotoma	Eupodes	**1.898**	**0.005**	**4.049**	**3.697**
Isotoma	Protodinychus	**1.898**	**0.549**	**4.049**	**3.095**
Isotoma	Scutacarus	**1.898**	**-0.608**	**4.049**	**3.396**
Isotoma	Stigmaeidae	**1.898**	**0.229**	**4.049**	**3.095**
Lepidocyrtus	Arctoseius	**1.231**	**-0.152**	**3.396**	**3.572**
Lepidocyrtus	Dendrolaelaps	**1.231**	**0.027**	**3.396**	**3.095**
Lepidocyrtus	Hypoaspis	**1.231**	**0.334**	**3.396**	**3.095**
Lepidocyrtus	Lysigamasus	**1.231**	**0.407**	**3.396**	**3.396**
Lepidocyrtus	Uropoda	**1.231**	**0.481**	**3.396**	**3.095**
Lepidocyrtus	Aporcelaimellus	**1.231**	**0.548**	**3.396**	**5.386**
Lepidocyrtus	Dorylaimoidea	**1.231**	**-0.604**	**3.396**	**5.650**
Lepidocyrtus	Epidorylaimus	**1.231**	**0.199**	**3.396**	**4.608**
Lepidocyrtus	Prodorylaimus	**1.231**	**-0.836**	**3.396**	**4.608**
Lepidocyrtus	Pungentus	**1.231**	**0.263**	**3.396**	**4.608**
Lepidocyrtus	Thornematidae	**1.231**	**-0.470**	**3.396**	**5.085**

Lepidocyrtus	Eupodes	**1.231**	**0.005**	**3.396**	**3.697**
Lepidocyrtus	Protodinychus	**1.231**	**0.549**	**3.396**	**3.095**
Lepidocyrtus	Scutacarus	**1.231**	**-0.608**	**3.396**	**3.396**
Lepidocyrtus	Stigmaeidae	**1.231**	**0.229**	**3.396**	**3.095**
Mesaphorura	Arctoseius	**0.618**	**-0.152**	**3.095**	**3.572**
Mesaphorura	Dendrolaelaps	**0.618**	**0.027**	**3.095**	**3.095**
Mesaphorura	Hypoaspis	**0.618**	**0.334**	**3.095**	**3.095**
Mesaphorura	Lysigamasus	**0.618**	**0.407**	**3.095**	**3.396**
Mesaphorura	Uropoda	**0.618**	**0.481**	**3.095**	**3.095**
Mesaphorura	Aporcelaimellus	**0.618**	**0.548**	**3.095**	**5.386**
Mesaphorura	Dorylaimoidea	**0.618**	**-0.604**	**3.095**	**5.650**
Mesaphorura	Epidorylaimus	**0.618**	**0.199**	**3.095**	**4.608**
Mesaphorura	Prodorylaimus	**0.618**	**-0.836**	**3.095**	**4.608**
Mesaphorura	Pungentus	**0.618**	**0.263**	**3.095**	**4.608**
Mesaphorura	Thornematidae	**0.618**	**-0.470**	**3.095**	**5.085**
Mesaphorura	Eupodes	**0.618**	**0.005**	**3.095**	**3.697**
Mesaphorura	Protodinychus	**0.618**	**0.549**	**3.095**	**3.095**
Mesaphorura	Scutacarus	**0.618**	**-0.608**	**3.095**	**3.396**
Mesaphorura	Stigmaeidae	**0.618**	**0.229**	**3.095**	**3.095**
Parisotoma	Arctoseius	**0.722**	**-0.152**	**3.998**	**3.572**
Parisotoma	Dendrolaelaps	**0.722**	**0.027**	**3.998**	**3.095**
Parisotoma	Hypoaspis	**0.722**	**0.334**	**3.998**	**3.095**
Parisotoma	Lysigamasus	**0.722**	**0.407**	**3.998**	**3.396**
Parisotoma	Uropoda	**0.722**	**0.481**	**3.998**	**3.095**
Parisotoma	Aporcelaimellus	**0.722**	**0.548**	**3.998**	**5.386**
Parisotoma	Dorylaimoidea	**0.722**	**-0.604**	**3.998**	**5.650**
Parisotoma	Epidorylaimus	**0.722**	**0.199**	**3.998**	**4.608**
Parisotoma	Prodorylaimus	**0.722**	**-0.836**	**3.998**	**4.608**
Parisotoma	Pungentus	**0.722**	**0.263**	**3.998**	**4.608**
Parisotoma	Thornematidae	**0.722**	**-0.470**	**3.998**	**5.085**
Parisotoma	Eupodes	**0.722**	**0.005**	**3.998**	**3.697**
Parisotoma	Protodinychus	**0.722**	**0.549**	**3.998**	**3.095**
Parisotoma	Scutacarus	**0.722**	**-0.608**	**3.998**	**3.396**
Parisotoma	Stigmaeidae	**0.722**	**0.229**	**3.998**	**3.095**
Proisotoma	Arctoseius	**0.770**	**-0.152**	**3.794**	**3.572**
Proisotoma	Dendrolaelaps	**0.770**	**0.027**	**3.794**	**3.095**
Proisotoma	Hypoaspis	**0.770**	**0.334**	**3.794**	**3.095**
Proisotoma	Lysigamasus	**0.770**	**0.407**	**3.794**	**3.396**
Proisotoma	Uropoda	**0.770**	**0.481**	**3.794**	**3.095**
Proisotoma	Aporcelaimellus	**0.770**	**0.548**	**3.794**	**5.386**
Proisotoma	Dorylaimoidea	**0.770**	**-0.604**	**3.794**	**5.650**
Proisotoma	Epidorylaimus	**0.770**	**0.199**	**3.794**	**4.608**
Proisotoma	Prodorylaimus	**0.770**	**-0.836**	**3.794**	**4.608**
Proisotoma	Pungentus	**0.770**	**0.263**	**3.794**	**4.608**
Proisotoma	Thornematidae	**0.770**	**-0.470**	**3.794**	**5.085**
Proisotoma	Eupodes	**0.770**	**0.005**	**3.794**	**3.697**
Proisotoma	Protodinychus	**0.770**	**0.549**	**3.794**	**3.095**
Proisotoma	Scutacarus	**0.770**	**-0.608**	**3.794**	**3.396**
Proisotoma	Stigmaeidae	**0.770**	**0.229**	**3.794**	**3.095**
Achaeta	Arctoseius	**1.375**	**-0.152**	**2.470**	**3.572**
Achaeta	Dendrolaelaps	**1.375**	**0.027**	**2.470**	**3.095**

Achaeta	Hypoaspis	**1.375**	**0.334**	**2.470**	**3.095**
Achaeta	Lysigamasus	**1.375**	**0.407**	**2.470**	**3.396**
Achaeta	Uropoda	**1.375**	**0.481**	**2.470**	**3.095**
Achaeta	Aporcelaimellus	**1.375**	**0.548**	**2.470**	**5.386**
Achaeta	Dorylaimoidea	**1.375**	**-0.604**	**2.470**	**5.650**
Achaeta	Epidorylaimus	**1.375**	**0.199**	**2.470**	**4.608**
Achaeta	Prodorylaimus	**1.375**	**-0.836**	**2.470**	**4.608**
Achaeta	Pungentus	**1.375**	**0.263**	**2.470**	**4.608**
Achaeta	Thornematidae	**1.375**	**-0.470**	**2.470**	**5.085**
Achaeta	Eupodes	**1.375**	**0.005**	**2.470**	**3.697**
Achaeta	Protodinychus	**1.375**	**0.549**	**2.470**	**3.095**
Achaeta	Scutacarus	**1.375**	**-0.608**	**2.470**	**3.396**
Achaeta	Stigmaeidae	**1.375**	**0.229**	**2.470**	**3.095**
Fridericia	Arctoseius	**1.999**	**-0.152**	**3.757**	**3.572**
Fridericia	Dendrolaelaps	**1.999**	**0.027**	**3.757**	**3.095**
Fridericia	Hypoaspis	**1.999**	**0.334**	**3.757**	**3.095**
Fridericia	Lysigamasus	**1.999**	**0.407**	**3.757**	**3.396**
Fridericia	Uropoda	**1.999**	**0.481**	**3.757**	**3.095**
Fridericia	Aporcelaimellus	**1.999**	**0.548**	**3.757**	**5.386**
Fridericia	Dorylaimoidea	**1.999**	**-0.604**	**3.757**	**5.650**
Fridericia	Epidorylaimus	**1.999**	**0.199**	**3.757**	**4.608**
Fridericia	Prodorylaimus	**1.999**	**-0.836**	**3.757**	**4.608**
Fridericia	Pungentus	**1.999**	**0.263**	**3.757**	**4.608**
Fridericia	Thornematidae	**1.999**	**-0.470**	**3.757**	**5.085**
Fridericia	Eupodes	**1.999**	**0.005**	**3.757**	**3.697**
Fridericia	Protodinychus	**1.999**	**0.549**	**3.757**	**3.095**
Fridericia	Scutacarus	**1.999**	**-0.608**	**3.757**	**3.396**
Fridericia	Stigmaeidae	**1.999**	**0.229**	**3.757**	**3.095**
Acrobeloides	Arctoseius	**-1.171**	**-0.152**	**5.511**	**3.572**
Acrobeloides	Dendrolaelaps	**-1.171**	**0.027**	**5.511**	**3.095**
Acrobeloides	Hypoaspis	**-1.171**	**0.334**	**5.511**	**3.095**
Acrobeloides	Lysigamasus	**-1.171**	**0.407**	**5.511**	**3.396**
Acrobeloides	Uropoda	**-1.171**	**0.481**	**5.511**	**3.095**
Acrobeloides	Aporcelaimellus	**-1.171**	**0.548**	**5.511**	**5.386**
Acrobeloides	Dorylaimoidea	**-1.171**	**-0.604**	**5.511**	**5.650**
Acrobeloides	Epidorylaimus	**-1.171**	**0.199**	**5.511**	**4.608**
Acrobeloides	Prodorylaimus	**-1.171**	**-0.836**	**5.511**	**4.608**
Acrobeloides	Pungentus	**-1.171**	**0.263**	**5.511**	**4.608**
Acrobeloides	Thornematidae	**-1.171**	**-0.470**	**5.511**	**5.085**
Acrobeloides	Eupodes	**-1.171**	**0.005**	**5.511**	**3.697**
Acrobeloides	Protodinychus	**-1.171**	**0.549**	**5.511**	**3.095**
Acrobeloides	Scutacarus	**-1.171**	**-0.608**	**5.511**	**3.396**
Acrobeloides	Stigmaeidae	**-1.171**	**0.229**	**5.511**	**3.095**
Anaplectus	Arctoseius	**-0.519**	**-0.152**	**4.608**	**3.572**
Anaplectus	Dendrolaelaps	**-0.519**	**0.027**	**4.608**	**3.095**
Anaplectus	Hypoaspis	**-0.519**	**0.334**	**4.608**	**3.095**
Anaplectus	Lysigamasus	**-0.519**	**0.407**	**4.608**	**3.396**
Anaplectus	Uropoda	**-0.519**	**0.481**	**4.608**	**3.095**
Anaplectus	Aporcelaimellus	**-0.519**	**0.548**	**4.608**	**5.386**
Anaplectus	Dorylaimoidea	**-0.519**	**-0.604**	**4.608**	**5.650**
Anaplectus	Epidorylaimus	**-0.519**	**0.199**	**4.608**	**4.608**

Anaplectus	Prodorylaimus	**-0.519**	**-0.836**	**4.608**	**4.608**
Anaplectus	Pungentus	**-0.519**	**0.263**	**4.608**	**4.608**
Anaplectus	Thornematidae	**-0.519**	**-0.470**	**4.608**	**5.085**
Anaplectus	Eupodes	**-0.519**	**0.005**	**4.608**	**3.697**
Anaplectus	Protodinychus	**-0.519**	**0.549**	**4.608**	**3.095**
Anaplectus	Scutacarus	**-0.519**	**-0.608**	**4.608**	**3.396**
Anaplectus	Stigmaeidae	**-0.519**	**0.229**	**4.608**	**3.095**
Cephalobidae	Arctoseius	**-1.055**	**-0.152**	**5.210**	**3.572**
Cephalobidae	Dendrolaelaps	**-1.055**	**0.027**	**5.210**	**3.095**
Cephalobidae	Hypoaspis	**-1.055**	**0.334**	**5.210**	**3.095**
Cephalobidae	Lysigamasus	**-1.055**	**0.407**	**5.210**	**3.396**
Cephalobidae	Uropoda	**-1.055**	**0.481**	**5.210**	**3.095**
Cephalobidae	Aporcelaimellus	**-1.055**	**0.548**	**5.210**	**5.386**
Cephalobidae	Dorylaimoidea	**-1.055**	**-0.604**	**5.210**	**5.650**
Cephalobidae	Epidorylaimus	**-1.055**	**0.199**	**5.210**	**4.608**
Cephalobidae	Prodorylaimus	**-1.055**	**-0.836**	**5.210**	**4.608**
Cephalobidae	Pungentus	**-1.055**	**0.263**	**5.210**	**4.608**
Cephalobidae	Thornematidae	**-1.055**	**-0.470**	**5.210**	**5.085**
Cephalobidae	Eupodes	**-1.055**	**0.005**	**5.210**	**3.697**
Cephalobidae	Protodinychus	**-1.055**	**0.549**	**5.210**	**3.095**
Cephalobidae	Scutacarus	**-1.055**	**-0.608**	**5.210**	**3.396**
Cephalobidae	Stigmaeidae	**-1.055**	**0.229**	**5.210**	**3.095**
Eucephalobus	Arctoseius	**-0.855**	**-0.152**	**5.687**	**3.572**
Eucephalobus	Dendrolaelaps	**-0.855**	**0.027**	**5.687**	**3.095**
Eucephalobus	Hypoaspis	**-0.855**	**0.334**	**5.687**	**3.095**
Eucephalobus	Lysigamasus	**-0.855**	**0.407**	**5.687**	**3.396**
Eucephalobus	Uropoda	**-0.855**	**0.481**	**5.687**	**3.095**
Eucephalobus	Aporcelaimellus	**-0.855**	**0.548**	**5.687**	**5.386**
Eucephalobus	Dorylaimoidea	**-0.855**	**-0.604**	**5.687**	**5.650**
Eucephalobus	Epidorylaimus	**-0.855**	**0.199**	**5.687**	**4.608**
Eucephalobus	Prodorylaimus	**-0.855**	**-0.836**	**5.687**	**4.608**
Eucephalobus	Pungentus	**-0.855**	**0.263**	**5.687**	**4.608**
Eucephalobus	Thornematidae	**-0.855**	**-0.470**	**5.687**	**5.085**
Eucephalobus	Eupodes	**-0.855**	**0.005**	**5.687**	**3.697**
Eucephalobus	Protodinychus	**-0.855**	**0.549**	**5.687**	**3.095**
Eucephalobus	Scutacarus	**-0.855**	**-0.608**	**5.687**	**3.396**
Eucephalobus	Stigmaeidae	**-0.855**	**0.229**	**5.687**	**3.095**
Eumonhystera	Arctoseius	**-0.613**	**-0.152**	**4.608**	**3.572**
Eumonhystera	Dendrolaelaps	**-0.613**	**0.027**	**4.608**	**3.095**
Eumonhystera	Hypoaspis	**-0.613**	**0.334**	**4.608**	**3.095**
Eumonhystera	Lysigamasus	**-0.613**	**0.407**	**4.608**	**3.396**
Eumonhystera	Uropoda	**-0.613**	**0.481**	**4.608**	**3.095**
Eumonhystera	Aporcelaimellus	**-0.613**	**0.548**	**4.608**	**5.386**
Eumonhystera	Dorylaimoidea	**-0.613**	**-0.604**	**4.608**	**5.650**
Eumonhystera	Epidorylaimus	**-0.613**	**0.199**	**4.608**	**4.608**
Eumonhystera	Prodorylaimus	**-0.613**	**-0.836**	**4.608**	**4.608**
Eumonhystera	Pungentus	**-0.613**	**0.263**	**4.608**	**4.608**
Eumonhystera	Thornematidae	**-0.613**	**-0.470**	**4.608**	**5.085**
Eumonhystera	Eupodes	**-0.613**	**0.005**	**4.608**	**3.697**
Eumonhystera	Protodinychus	**-0.613**	**0.549**	**4.608**	**3.095**
Eumonhystera	Scutacarus	**-0.613**	**-0.608**	**4.608**	**3.396**

Eumonhystera	Stigmaeidae	**-0.613**	**0.229**	**4.608**	**3.095**
Panagrolaimus	Arctoseius	**-0.945**	**-0.152**	**5.085**	**3.572**
Panagrolaimus	Dendrolaelaps	**-0.945**	**0.027**	**5.085**	**3.095**
Panagrolaimus	Hypoaspis	**-0.945**	**0.334**	**5.085**	**3.095**
Panagrolaimus	Lysigamasus	**-0.945**	**0.407**	**5.085**	**3.396**
Panagrolaimus	Uropoda	**-0.945**	**0.481**	**5.085**	**3.095**
Panagrolaimus	Aporcelaimellus	**-0.945**	**0.548**	**5.085**	**5.386**
Panagrolaimus	Dorylaimoidea	**-0.945**	**-0.604**	**5.085**	**5.650**
Panagrolaimus	Epidorylaimus	**-0.945**	**0.199**	**5.085**	**4.608**
Panagrolaimus	Prodorylaimus	**-0.945**	**-0.836**	**5.085**	**4.608**
Panagrolaimus	Pungentus	**-0.945**	**0.263**	**5.085**	**4.608**
Panagrolaimus	Thornematidae	**-0.945**	**-0.470**	**5.085**	**5.085**
Panagrolaimus	Eupodes	**-0.945**	**0.005**	**5.085**	**3.697**
Panagrolaimus	Protodinychus	**-0.945**	**0.549**	**5.085**	**3.095**
Panagrolaimus	Scutacarus	**-0.945**	**-0.608**	**5.085**	**3.396**
Panagrolaimus	Stigmaeidae	**-0.945**	**0.229**	**5.085**	**3.095**
Plectus	Arctoseius	**-0.583**	**-0.152**	**5.386**	**3.572**
Plectus	Dendrolaelaps	**-0.583**	**0.027**	**5.386**	**3.095**
Plectus	Hypoaspis	**-0.583**	**0.334**	**5.386**	**3.095**
Plectus	Lysigamasus	**-0.583**	**0.407**	**5.386**	**3.396**
Plectus	Uropoda	**-0.583**	**0.481**	**5.386**	**3.095**
Plectus	Aporcelaimellus	**-0.583**	**0.548**	**5.386**	**5.386**
Plectus	Dorylaimoidea	**-0.583**	**-0.604**	**5.386**	**5.650**
Plectus	Epidorylaimus	**-0.583**	**0.199**	**5.386**	**4.608**
Plectus	Prodorylaimus	**-0.583**	**-0.836**	**5.386**	**4.608**
Plectus	Pungentus	**-0.583**	**0.263**	**5.386**	**4.608**
Plectus	Thornematidae	**-0.583**	**-0.470**	**5.386**	**5.085**
Plectus	Eupodes	**-0.583**	**0.005**	**5.386**	**3.697**
Plectus	Protodinychus	**-0.583**	**0.549**	**5.386**	**3.095**
Plectus	Scutacarus	**-0.583**	**-0.608**	**5.386**	**3.396**
Plectus	Stigmaeidae	**-0.583**	**0.229**	**5.386**	**3.095**
Prismatolaimus	Arctoseius	**-1.280**	**-0.152**	**4.608**	**3.572**
Prismatolaimus	Dendrolaelaps	**-1.280**	**0.027**	**4.608**	**3.095**
Prismatolaimus	Hypoaspis	**-1.280**	**0.334**	**4.608**	**3.095**
Prismatolaimus	Lysigamasus	**-1.280**	**0.407**	**4.608**	**3.396**
Prismatolaimus	Uropoda	**-1.280**	**0.481**	**4.608**	**3.095**
Prismatolaimus	Aporcelaimellus	**-1.280**	**0.548**	**4.608**	**5.386**
Prismatolaimus	Dorylaimoidea	**-1.280**	**-0.604**	**4.608**	**5.650**
Prismatolaimus	Epidorylaimus	**-1.280**	**0.199**	**4.608**	**4.608**
Prismatolaimus	Prodorylaimus	**-1.280**	**-0.836**	**4.608**	**4.608**
Prismatolaimus	Pungentus	**-1.280**	**0.263**	**4.608**	**4.608**
Prismatolaimus	Thornematidae	**-1.280**	**-0.470**	**4.608**	**5.085**
Prismatolaimus	Eupodes	**-1.280**	**0.005**	**4.608**	**3.697**
Prismatolaimus	Protodinychus	**-1.280**	**0.549**	**4.608**	**3.095**
Prismatolaimus	Scutacarus	**-1.280**	**-0.608**	**4.608**	**3.396**
Prismatolaimus	Stigmaeidae	**-1.280**	**0.229**	**4.608**	**3.095**
Rhabditidae	Arctoseius	**-0.692**	**-0.152**	**5.650**	**3.572**
Rhabditidae	Dendrolaelaps	**-0.692**	**0.027**	**5.650**	**3.095**
Rhabditidae	Hypoaspis	**-0.692**	**0.334**	**5.650**	**3.095**
Rhabditidae	Lysigamasus	**-0.692**	**0.407**	**5.650**	**3.396**
Rhabditidae	Uropoda	**-0.692**	**0.481**	**5.650**	**3.095**

Rhabditidae	Aporcelaimellus	**-0.692**	**0.548**	**5.650**	**5.386**
Rhabditidae	Dorylaimoidea	**-0.692**	**-0.604**	**5.650**	**5.650**
Rhabditidae	Epidorylaimus	**-0.692**	**0.199**	**5.650**	**4.608**
Rhabditidae	Prodorylaimus	**-0.692**	**-0.836**	**5.650**	**4.608**
Rhabditidae	Pungentus	**-0.692**	**0.263**	**5.650**	**4.608**
Rhabditidae	Thornematidae	**-0.692**	**-0.470**	**5.650**	**5.085**
Rhabditidae	Eupodes	**-0.692**	**0.005**	**5.650**	**3.697**
Rhabditidae	Protodinychus	**-0.692**	**0.549**	**5.650**	**3.095**
Rhabditidae	Scutacarus	**-0.692**	**-0.608**	**5.650**	**3.396**
Rhabditidae	Stigmaeidae	**-0.692**	**0.229**	**5.650**	**3.095**
Enchytraeus	Arctoseius	**1.281**	**-0.152**	**3.720**	**3.572**
Enchytraeus	Dendrolaelaps	**1.281**	**0.027**	**3.720**	**3.095**
Enchytraeus	Hypoaspis	**1.281**	**0.334**	**3.720**	**3.095**
Enchytraeus	Lysigamasus	**1.281**	**0.407**	**3.720**	**3.396**
Enchytraeus	Uropoda	**1.281**	**0.481**	**3.720**	**3.095**
Enchytraeus	Aporcelaimellus	**1.281**	**0.548**	**3.720**	**5.386**
Enchytraeus	Dorylaimoidea	**1.281**	**-0.604**	**3.720**	**5.650**
Enchytraeus	Epidorylaimus	**1.281**	**0.199**	**3.720**	**4.608**
Enchytraeus	Prodorylaimus	**1.281**	**-0.836**	**3.720**	**4.608**
Enchytraeus	Pungentus	**1.281**	**0.263**	**3.720**	**4.608**
Enchytraeus	Thornematidae	**1.281**	**-0.470**	**3.720**	**5.085**
Enchytraeus	Eupodes	**1.281**	**0.005**	**3.720**	**3.697**
Enchytraeus	Protodinychus	**1.281**	**0.549**	**3.720**	**3.095**
Enchytraeus	Scutacarus	**1.281**	**-0.608**	**3.720**	**3.396**
Enchytraeus	Stigmaeidae	**1.281**	**0.229**	**3.720**	**3.095**
Eubacteria	Acrobeloides	**-6.618**	**-1.171**	**12.312**	**5.511**
Eubacteria	Anaplectus	**-6.618**	**-0.519**	**12.312**	**4.608**
Eubacteria	Cephalobidae	**-6.618**	**-1.055**	**12.312**	**5.210**
Eubacteria	Eucephalobus	**-6.618**	**-0.855**	**12.312**	**5.687**
Eubacteria	Eumonhystera	**-6.618**	**-0.613**	**12.312**	**4.608**
Eubacteria	Panagrolaimus	**-6.618**	**-0.945**	**12.312**	**5.085**
Eubacteria	Plectus	**-6.618**	**-0.583**	**12.312**	**5.386**
Eubacteria	Prismatolaimus	**-6.618**	**-1.280**	**12.312**	**4.608**
Eubacteria	Rhabditidae	**-6.618**	**-0.692**	**12.312**	**5.650**
Eubacteria	Enchytraeus	**-6.618**	**1.281**	**12.312**	**3.720**
Eubacteria	Henlea	**-6.618**	**1.413**	**12.312**	**3.511**
Eubacteria	Marionina	**-6.618**	**0.903**	**12.312**	**3.248**
Henlea	Arctoseius	**1.413**	**-0.152**	**3.511**	**3.572**
Henlea	Dendrolaelaps	**1.413**	**0.027**	**3.511**	**3.095**
Henlea	Hypoaspis	**1.413**	**0.334**	**3.511**	**3.095**
Henlea	Lysigamasus	**1.413**	**0.407**	**3.511**	**3.396**
Henlea	Uropoda	**1.413**	**0.481**	**3.511**	**3.095**
Henlea	Aporcelaimellus	**1.413**	**0.548**	**3.511**	**5.386**
Henlea	Dorylaimoidea	**1.413**	**-0.604**	**3.511**	**5.650**
Henlea	Epidorylaimus	**1.413**	**0.199**	**3.511**	**4.608**
Henlea	Prodorylaimus	**1.413**	**-0.836**	**3.511**	**4.608**
Henlea	Pungentus	**1.413**	**0.263**	**3.511**	**4.608**
Henlea	Thornematidae	**1.413**	**-0.470**	**3.511**	**5.085**
Henlea	Eupodes	**1.413**	**0.005**	**3.511**	**3.697**
Henlea	Protodinychus	**1.413**	**0.549**	**3.511**	**3.095**
Henlea	Scutacarus	**1.413**	**-0.608**	**3.511**	**3.396**

Henlea	Stigmaeidae	**1.413**	**0.229**	**3.511**	**3.095**
Marionina	Arctoseius	**0.903**	**-0.152**	**3.248**	**3.572**
Marionina	Dendrolaelaps	**0.903**	**0.027**	**3.248**	**3.095**
Marionina	Hypoaspis	**0.903**	**0.334**	**3.248**	**3.095**
Marionina	Lysigamasus	**0.903**	**0.407**	**3.248**	**3.396**
Marionina	Uropoda	**0.903**	**0.481**	**3.248**	**3.095**
Marionina	Aporcelaimellus	**0.903**	**0.548**	**3.248**	**5.386**
Marionina	Dorylaimoidea	**0.903**	**-0.604**	**3.248**	**5.650**
Marionina	Epidorylaimus	**0.903**	**0.199**	**3.248**	**4.608**
Marionina	Prodorylaimus	**0.903**	**-0.836**	**3.248**	**4.608**
Marionina	Pungentus	**0.903**	**0.263**	**3.248**	**4.608**
Marionina	Thornematidae	**0.903**	**-0.470**	**3.248**	**5.085**
Marionina	Eupodes	**0.903**	**0.005**	**3.248**	**3.697**
Marionina	Protodinychus	**0.903**	**0.549**	**3.248**	**3.095**
Marionina	Scutacarus	**0.903**	**-0.608**	**3.248**	**3.396**
Marionina	Stigmaeidae	**0.903**	**0.229**	**3.248**	**3.095**
Hyphae and hair roots	Aglenchus	**6.187**	**-1.053**	**0.000**	**5.864**
Hyphae and hair roots	Dolichodoridae	**6.187**	**-0.885**	**0.000**	**4.608**
Hyphae and hair roots	Filenchus	**6.187**	**-1.033**	**0.000**	**4.909**
Hyphae and hair roots	Helicotylenchus	**6.187**	**-0.792**	**0.000**	**5.453**
Hyphae and hair roots	Malenchus	**6.187**	**-1.330**	**0.000**	**5.085**
Hyphae and hair roots	Meloidogyne	**6.187**	**-1.287**	**0.000**	**4.608**
Hyphae and hair roots	Paratylenchus	**6.187**	**-1.244**	**0.000**	**5.085**
Hyphae and hair roots	Pratylenchus	**6.187**	**-1.226**	**0.000**	**4.608**
Hyphae and hair roots	Trichodorus	**6.187**	**-0.744**	**0.000**	**4.608**
Hyphae and hair roots	Tylenchorhynchus	**6.187**	**-0.664**	**0.000**	**5.386**
Hyphae and hair roots	Achipteria	**6.187**	**0.341**	**0.000**	**4.299**
Hyphae and hair roots	Pachygnatidae	**6.187**	**-0.113**	**0.000**	**3.095**
Hyphae and hair roots	Platynothrus	**6.187**	**0.710**	**0.000**	**3.697**
Hyphae and hair roots	Tydeidae	**6.187**	**-0.608**	**0.000**	**3.396**
Hyphae and hair roots	Sphaeridia	**6.187**	**0.202**	**0.000**	**3.095**
Hyphae and hair roots	Aphelenchoides	**6.187**	**-1.496**	**0.000**	**5.386**
Hyphae and hair roots	Aphelenchus	**6.187**	**-1.129**	**0.000**	**4.909**
Hyphae and hair roots	Tylenchidae	**6.187**	**-1.360**	**0.000**	**6.071**
Hyphae and hair roots	Liebstadia	**6.187**	**0.270**	**0.000**	**3.095**
Hyphae and hair roots	Pygmephorus	**6.187**	**-0.376**	**0.000**	**3.095**
Hyphae and hair roots	Ceratophysella	**6.187**	**1.335**	**0.000**	**3.095**
Hyphae and hair roots	Folsomia	**6.187**	**0.900**	**0.000**	**3.095**
Hyphae and hair roots	Isotoma	**6.187**	**1.898**	**0.000**	**4.049**
Hyphae and hair roots	Lepidocyrtus	**6.187**	**1.231**	**0.000**	**3.396**
Hyphae and hair roots	Mesaphorura	**6.187**	**0.618**	**0.000**	**3.095**
Hyphae and hair roots	Parisotoma	**6.187**	**0.722**	**0.000**	**3.998**
Hyphae and hair roots	Proisotoma	**6.187**	**0.770**	**0.000**	**3.794**
Hyphae and hair roots	Achaeta	**6.187**	**1.375**	**0.000**	**2.470**
Hyphae and hair roots	Fridericia	**6.187**	**1.999**	**0.000**	**3.757**
Hyphae and hair roots	Aporcelaimellus	**6.187**	**0.548**	**0.000**	**5.386**
Hyphae and hair roots	Dorylaimoidea	**6.187**	**-0.604**	**0.000**	**5.650**
Hyphae and hair roots	Epidorylaimus	**6.187**	**0.199**	**0.000**	**4.608**
Hyphae and hair roots	Prodorylaimus	**6.187**	**-0.836**	**0.000**	**4.608**
Hyphae and hair roots	Pungentus	**6.187**	**0.263**	**0.000**	**4.608**
Hyphae and hair roots	Thornematidae	**6.187**	**-0.470**	**0.000**	**5.085**

Hyphae and hair roots	Eupodes	**6.187**	**0.005**	**0.000**	**3.697**
Hyphae and hair roots	Protodinychus	**6.187**	**0.549**	**0.000**	**3.095**
Hyphae and hair roots	Scutacarus	**6.187**	**-0.608**	**0.000**	**3.396**
Hyphae and hair roots	Stigmaeidae	**6.187**	**0.229**	**0.000**	**3.095**
Arctoseius	Aporcelaimellus	**-0.152**	**0.548**	**3.572**	**5.386**
Arctoseius	Dorylaimoidea	**-0.152**	**-0.604**	**3.572**	**5.650**
Arctoseius	Epidorylaimus	**-0.152**	**0.199**	**3.572**	**4.608**
Arctoseius	Prodorylaimus	**-0.152**	**-0.836**	**3.572**	**4.608**
Arctoseius	Pungentus	**-0.152**	**0.263**	**3.572**	**4.608**
Arctoseius	Thornematidae	**-0.152**	**-0.470**	**3.572**	**5.085**
Arctoseius	Eupodes	**-0.152**	**0.005**	**3.572**	**3.697**
Arctoseius	Protodinychus	**-0.152**	**0.549**	**3.572**	**3.095**
Arctoseius	Scutacarus	**-0.152**	**-0.608**	**3.572**	**3.396**
Arctoseius	Stigmaeidae	**-0.152**	**0.229**	**3.572**	**3.095**
Dendrolaelaps	Aporcelaimellus	**0.027**	**0.548**	**3.095**	**5.386**
Dendrolaelaps	Dorylaimoidea	**0.027**	**-0.604**	**3.095**	**5.650**
Dendrolaelaps	Epidorylaimus	**0.027**	**0.199**	**3.095**	**4.608**
Dendrolaelaps	Prodorylaimus	**0.027**	**-0.836**	**3.095**	**4.608**
Dendrolaelaps	Pungentus	**0.027**	**0.263**	**3.095**	**4.608**
Dendrolaelaps	Thornematidae	**0.027**	**-0.470**	**3.095**	**5.085**
Dendrolaelaps	Eupodes	**0.027**	**0.005**	**3.095**	**3.697**
Dendrolaelaps	Protodinychus	**0.027**	**0.549**	**3.095**	**3.095**
Dendrolaelaps	Scutacarus	**0.027**	**-0.608**	**3.095**	**3.396**
Dendrolaelaps	Stigmaeidae	**0.027**	**0.229**	**3.095**	**3.095**
Hypoaspis	Aporcelaimellus	**0.334**	**0.548**	**3.095**	**5.386**
Hypoaspis	Dorylaimoidea	**0.334**	**-0.604**	**3.095**	**5.650**
Hypoaspis	Epidorylaimus	**0.334**	**0.199**	**3.095**	**4.608**
Hypoaspis	Prodorylaimus	**0.334**	**-0.836**	**3.095**	**4.608**
Hypoaspis	Pungentus	**0.334**	**0.263**	**3.095**	**4.608**
Hypoaspis	Thornematidae	**0.334**	**-0.470**	**3.095**	**5.085**
Hypoaspis	Eupodes	**0.334**	**0.005**	**3.095**	**3.697**
Hypoaspis	Protodinychus	**0.334**	**0.549**	**3.095**	**3.095**
Hypoaspis	Scutacarus	**0.334**	**-0.608**	**3.095**	**3.396**
Hypoaspis	Stigmaeidae	**0.334**	**0.229**	**3.095**	**3.095**
Lysigamasus	Aporcelaimellus	**0.407**	**0.548**	**3.396**	**5.386**
Lysigamasus	Dorylaimoidea	**0.407**	**-0.604**	**3.396**	**5.650**
Lysigamasus	Epidorylaimus	**0.407**	**0.199**	**3.396**	**4.608**
Lysigamasus	Prodorylaimus	**0.407**	**-0.836**	**3.396**	**4.608**
Lysigamasus	Pungentus	**0.407**	**0.263**	**3.396**	**4.608**
Lysigamasus	Thornematidae	**0.407**	**-0.470**	**3.396**	**5.085**
Lysigamasus	Eupodes	**0.407**	**0.005**	**3.396**	**3.697**
Lysigamasus	Protodinychus	**0.407**	**0.549**	**3.396**	**3.095**
Lysigamasus	Scutacarus	**0.407**	**-0.608**	**3.396**	**3.396**
Lysigamasus	Stigmaeidae	**0.407**	**0.229**	**3.396**	**3.095**
Uropoda	Aporcelaimellus	**0.481**	**0.548**	**3.095**	**5.386**
Uropoda	Dorylaimoidea	**0.481**	**-0.604**	**3.095**	**5.650**
Uropoda	Epidorylaimus	**0.481**	**0.199**	**3.095**	**4.608**
Uropoda	Prodorylaimus	**0.481**	**-0.836**	**3.095**	**4.608**
Uropoda	Pungentus	**0.481**	**0.263**	**3.095**	**4.608**
Uropoda	Thornematidae	**0.481**	**-0.470**	**3.095**	**5.085**
Uropoda	Eupodes	**0.481**	**0.005**	**3.095**	**3.697**

Uropoda	Protodinychus	**0.481**	**0.549**	**3.095**	**3.095**
Uropoda	Scutacarus	**0.481**	**-0.608**	**3.095**	**3.396**
Uropoda	Stigmaeidae	**0.481**	**0.229**	**3.095**	**3.095**
Aporcelaimellus	Arctoseius	**0.548**	**-0.152**	**5.386**	**3.572**
Aporcelaimellus	Dendrolaelaps	**0.548**	**0.027**	**5.386**	**3.095**
Aporcelaimellus	Hypoaspis	**0.548**	**0.334**	**5.386**	**3.095**
Aporcelaimellus	Lysigamasus	**0.548**	**0.407**	**5.386**	**3.396**
Aporcelaimellus	Uropoda	**0.548**	**0.481**	**5.386**	**3.095**
Aporcelaimellus	Aporcelaimellus	**0.548**	**0.548**	**5.386**	**5.386**
Aporcelaimellus	Dorylaimoidea	**0.548**	**-0.604**	**5.386**	**5.650**
Aporcelaimellus	Epidorylaimus	**0.548**	**0.199**	**5.386**	**4.608**
Aporcelaimellus	Prodorylaimus	**0.548**	**-0.836**	**5.386**	**4.608**
Aporcelaimellus	Pungentus	**0.548**	**0.263**	**5.386**	**4.608**
Aporcelaimellus	Thornematidae	**0.548**	**-0.470**	**5.386**	**5.085**
Aporcelaimellus	Eupodes	**0.548**	**0.005**	**5.386**	**3.697**
Aporcelaimellus	Protodinychus	**0.548**	**0.549**	**5.386**	**3.095**
Aporcelaimellus	Scutacarus	**0.548**	**-0.608**	**5.386**	**3.396**
Aporcelaimellus	Stigmaeidae	**0.548**	**0.229**	**5.386**	**3.095**
Dorylaimoidea	Arctoseius	**-0.604**	**-0.152**	**5.650**	**3.572**
Dorylaimoidea	Dendrolaelaps	**-0.604**	**0.027**	**5.650**	**3.095**
Dorylaimoidea	Hypoaspis	**-0.604**	**0.334**	**5.650**	**3.095**
Dorylaimoidea	Lysigamasus	**-0.604**	**0.407**	**5.650**	**3.396**
Dorylaimoidea	Uropoda	**-0.604**	**0.481**	**5.650**	**3.095**
Dorylaimoidea	Aporcelaimellus	**-0.604**	**0.548**	**5.650**	**5.386**
Dorylaimoidea	Dorylaimoidea	**-0.604**	**-0.604**	**5.650**	**5.650**
Dorylaimoidea	Epidorylaimus	**-0.604**	**0.199**	**5.650**	**4.608**
Dorylaimoidea	Prodorylaimus	**-0.604**	**-0.836**	**5.650**	**4.608**
Dorylaimoidea	Pungentus	**-0.604**	**0.263**	**5.650**	**4.608**
Dorylaimoidea	Thornematidae	**-0.604**	**-0.470**	**5.650**	**5.085**
Dorylaimoidea	Eupodes	**-0.604**	**0.005**	**5.650**	**3.697**
Dorylaimoidea	Protodinychus	**-0.604**	**0.549**	**5.650**	**3.095**
Dorylaimoidea	Scutacarus	**-0.604**	**-0.608**	**5.650**	**3.396**
Dorylaimoidea	Stigmaeidae	**-0.604**	**0.229**	**5.650**	**3.095**
Epidorylaimus	Arctoseius	**0.199**	**-0.152**	**4.608**	**3.572**
Epidorylaimus	Dendrolaelaps	**0.199**	**0.027**	**4.608**	**3.095**
Epidorylaimus	Hypoaspis	**0.199**	**0.334**	**4.608**	**3.095**
Epidorylaimus	Lysigamasus	**0.199**	**0.407**	**4.608**	**3.396**
Epidorylaimus	Uropoda	**0.199**	**0.481**	**4.608**	**3.095**
Epidorylaimus	Aporcelaimellus	**0.199**	**0.548**	**4.608**	**5.386**
Epidorylaimus	Dorylaimoidea	**0.199**	**-0.604**	**4.608**	**5.650**
Epidorylaimus	Epidorylaimus	**0.199**	**0.199**	**4.608**	**4.608**
Epidorylaimus	Prodorylaimus	**0.199**	**-0.836**	**4.608**	**4.608**
Epidorylaimus	Pungentus	**0.199**	**0.263**	**4.608**	**4.608**
Epidorylaimus	Thornematidae	**0.199**	**-0.470**	**4.608**	**5.085**
Epidorylaimus	Eupodes	**0.199**	**0.005**	**4.608**	**3.697**
Epidorylaimus	Protodinychus	**0.199**	**0.549**	**4.608**	**3.095**
Epidorylaimus	Scutacarus	**0.199**	**-0.608**	**4.608**	**3.396**
Epidorylaimus	Stigmaeidae	**0.199**	**0.229**	**4.608**	**3.095**
Prodorylaimus	Arctoseius	**-0.836**	**-0.152**	**4.608**	**3.572**
Prodorylaimus	Dendrolaelaps	**-0.836**	**0.027**	**4.608**	**3.095**
Prodorylaimus	Hypoaspis	**-0.836**	**0.334**	**4.608**	**3.095**

Prodorylaimus	Lysigamasus	**-0.836**	**0.407**	**4.608**	**3.396**
Prodorylaimus	Uropoda	**-0.836**	**0.481**	**4.608**	**3.095**
Prodorylaimus	Aporcelaimellus	**-0.836**	**0.548**	**4.608**	**5.386**
Prodorylaimus	Dorylaimoidea	**-0.836**	**-0.604**	**4.608**	**5.650**
Prodorylaimus	Epidorylaimus	**-0.836**	**0.199**	**4.608**	**4.608**
Prodorylaimus	Prodorylaimus	**-0.836**	**-0.836**	**4.608**	**4.608**
Prodorylaimus	Pungentus	**-0.836**	**0.263**	**4.608**	**4.608**
Prodorylaimus	Thornematidae	**-0.836**	**-0.470**	**4.608**	**5.085**
Prodorylaimus	Eupodes	**-0.836**	**0.005**	**4.608**	**3.697**
Prodorylaimus	Protodinychus	**-0.836**	**0.549**	**4.608**	**3.095**
Prodorylaimus	Scutacarus	**-0.836**	**-0.608**	**4.608**	**3.396**
Prodorylaimus	Stigmaeidae	**-0.836**	**0.229**	**4.608**	**3.095**
Pungentus	Arctoseius	**0.263**	**-0.152**	**4.608**	**3.572**
Pungentus	Dendrolaelaps	**0.263**	**0.027**	**4.608**	**3.095**
Pungentus	Hypoaspis	**0.263**	**0.334**	**4.608**	**3.095**
Pungentus	Lysigamasus	**0.263**	**0.407**	**4.608**	**3.396**
Pungentus	Uropoda	**0.263**	**0.481**	**4.608**	**3.095**
Pungentus	Aporcelaimellus	**0.263**	**0.548**	**4.608**	**5.386**
Pungentus	Dorylaimoidea	**0.263**	**-0.604**	**4.608**	**5.650**
Pungentus	Epidorylaimus	**0.263**	**0.199**	**4.608**	**4.608**
Pungentus	Prodorylaimus	**0.263**	**-0.836**	**4.608**	**4.608**
Pungentus	Pungentus	**0.263**	**0.263**	**4.608**	**4.608**
Pungentus	Thornematidae	**0.263**	**-0.470**	**4.608**	**5.085**
Pungentus	Eupodes	**0.263**	**0.005**	**4.608**	**3.697**
Pungentus	Protodinychus	**0.263**	**0.549**	**4.608**	**3.095**
Pungentus	Scutacarus	**0.263**	**-0.608**	**4.608**	**3.396**
Pungentus	Stigmaeidae	**0.263**	**0.229**	**4.608**	**3.095**
Thornematidae	Arctoseius	**-0.470**	**-0.152**	**5.085**	**3.572**
Thornematidae	Dendrolaelaps	**-0.470**	**0.027**	**5.085**	**3.095**
Thornematidae	Hypoaspis	**-0.470**	**0.334**	**5.085**	**3.095**
Thornematidae	Lysigamasus	**-0.470**	**0.407**	**5.085**	**3.396**
Thornematidae	Uropoda	**-0.470**	**0.481**	**5.085**	**3.095**
Thornematidae	Aporcelaimellus	**-0.470**	**0.548**	**5.085**	**5.386**
Thornematidae	Dorylaimoidea	**-0.470**	**-0.604**	**5.085**	**5.650**
Thornematidae	Epidorylaimus	**-0.470**	**0.199**	**5.085**	**4.608**
Thornematidae	Prodorylaimus	**-0.470**	**-0.836**	**5.085**	**4.608**
Thornematidae	Pungentus	**-0.470**	**0.263**	**5.085**	**4.608**
Thornematidae	Thornematidae	**-0.470**	**-0.470**	**5.085**	**5.085**
Thornematidae	Eupodes	**-0.470**	**0.005**	**5.085**	**3.697**
Thornematidae	Protodinychus	**-0.470**	**0.549**	**5.085**	**3.095**
Thornematidae	Scutacarus	**-0.470**	**-0.608**	**5.085**	**3.396**
Thornematidae	Stigmaeidae	**-0.470**	**0.229**	**5.085**	**3.095**
Eupodes	Arctoseius	**0.005**	**-0.152**	**3.697**	**3.572**
Eupodes	Dendrolaelaps	**0.005**	**0.027**	**3.697**	**3.095**
Eupodes	Hypoaspis	**0.005**	**0.334**	**3.697**	**3.095**
Eupodes	Lysigamasus	**0.005**	**0.407**	**3.697**	**3.396**
Eupodes	Uropoda	**0.005**	**0.481**	**3.697**	**3.095**
Eupodes	Aporcelaimellus	**0.005**	**0.548**	**3.697**	**5.386**
Eupodes	Dorylaimoidea	**0.005**	**-0.604**	**3.697**	**5.650**
Eupodes	Epidorylaimus	**0.005**	**0.199**	**3.697**	**4.608**
Eupodes	Prodorylaimus	**0.005**	**-0.836**	**3.697**	**4.608**

Eupodes	Pungentus	**0.005**	**0.263**	**3.697**	**4.608**
Eupodes	Thornematidae	**0.005**	**-0.470**	**3.697**	**5.085**
Eupodes	Eupodes	**0.005**	**0.005**	**3.697**	**3.697**
Eupodes	Protodinychus	**0.005**	**0.549**	**3.697**	**3.095**
Eupodes	Scutacarus	**0.005**	**-0.608**	**3.697**	**3.396**
Eupodes	Stigmaeidae	**0.005**	**0.229**	**3.697**	**3.095**
Protodinychus	Arctoseius	**0.549**	**-0.152**	**3.095**	**3.572**
Protodinychus	Dendrolaelaps	**0.549**	**0.027**	**3.095**	**3.095**
Protodinychus	Hypoaspis	**0.549**	**0.334**	**3.095**	**3.095**
Protodinychus	Lysigamasus	**0.549**	**0.407**	**3.095**	**3.396**
Protodinychus	Uropoda	**0.549**	**0.481**	**3.095**	**3.095**
Protodinychus	Aporcelaimellus	**0.549**	**0.548**	**3.095**	**5.386**
Protodinychus	Dorylaimoidea	**0.549**	**-0.604**	**3.095**	**5.650**
Protodinychus	Epidorylaimus	**0.549**	**0.199**	**3.095**	**4.608**
Protodinychus	Prodorylaimus	**0.549**	**-0.836**	**3.095**	**4.608**
Protodinychus	Pungentus	**0.549**	**0.263**	**3.095**	**4.608**
Protodinychus	Thornematidae	**0.549**	**-0.470**	**3.095**	**5.085**
Protodinychus	Eupodes	**0.549**	**0.005**	**3.095**	**3.697**
Protodinychus	Protodinychus	**0.549**	**0.549**	**3.095**	**3.095**
Protodinychus	Scutacarus	**0.549**	**-0.608**	**3.095**	**3.396**
Protodinychus	Stigmaeidae	**0.549**	**0.229**	**3.095**	**3.095**
Scutacarus	Arctoseius	**-0.608**	**-0.152**	**3.396**	**3.572**
Scutacarus	Dendrolaelaps	**-0.608**	**0.027**	**3.396**	**3.095**
Scutacarus	Hypoaspis	**-0.608**	**0.334**	**3.396**	**3.095**
Scutacarus	Lysigamasus	**-0.608**	**0.407**	**3.396**	**3.396**
Scutacarus	Uropoda	**-0.608**	**0.481**	**3.396**	**3.095**
Scutacarus	Aporcelaimellus	**-0.608**	**0.548**	**3.396**	**5.386**
Scutacarus	Dorylaimoidea	**-0.608**	**-0.604**	**3.396**	**5.650**
Scutacarus	Epidorylaimus	**-0.608**	**0.199**	**3.396**	**4.608**
Scutacarus	Prodorylaimus	**-0.608**	**-0.836**	**3.396**	**4.608**
Scutacarus	Pungentus	**-0.608**	**0.263**	**3.396**	**4.608**
Scutacarus	Thornematidae	**-0.608**	**-0.470**	**3.396**	**5.085**
Scutacarus	Eupodes	**-0.608**	**0.005**	**3.396**	**3.697**
Scutacarus	Protodinychus	**-0.608**	**0.549**	**3.396**	**3.095**
Scutacarus	Scutacarus	**-0.608**	**-0.608**	**3.396**	**3.396**
Scutacarus	Stigmaeidae	**-0.608**	**0.229**	**3.396**	**3.095**
Stigmaeidae	Arctoseius	**0.229**	**-0.152**	**3.095**	**3.572**
Stigmaeidae	Dendrolaelaps	**0.229**	**0.027**	**3.095**	**3.095**
Stigmaeidae	Hypoaspis	**0.229**	**0.334**	**3.095**	**3.095**
Stigmaeidae	Lysigamasus	**0.229**	**0.407**	**3.095**	**3.396**
Stigmaeidae	Uropoda	**0.229**	**0.481**	**3.095**	**3.095**
Stigmaeidae	Aporcelaimellus	**0.229**	**0.548**	**3.095**	**5.386**
Stigmaeidae	Dorylaimoidea	**0.229**	**-0.604**	**3.095**	**5.650**
Stigmaeidae	Epidorylaimus	**0.229**	**0.199**	**3.095**	**4.608**
Stigmaeidae	Prodorylaimus	**0.229**	**-0.836**	**3.095**	**4.608**
Stigmaeidae	Pungentus	**0.229**	**0.263**	**3.095**	**4.608**
Stigmaeidae	Thornematidae	**0.229**	**-0.470**	**3.095**	**5.085**
Stigmaeidae	Eupodes	**0.229**	**0.005**	**3.095**	**3.697**
Stigmaeidae	Protodinychus	**0.229**	**0.549**	**3.095**	**3.095**
Stigmaeidae	Scutacarus	**0.229**	**-0.608**	**3.095**	**3.396**
Stigmaeidae	Stigmaeidae	**0.229**	**0.229**	**3.095**	**3.095**

Resource	Consumer	Mres	Mconsumer	Nres	Nconsumer
Criconematidae	Anatonchus	**-0.753**	**0.406**	**5.287**	**4.685**
Criconematidae	Mononchus	**-0.753**	**-0.938**	**5.287**	**4.685**
Criconematidae	Mylonchulus	**-0.753**	**-0.005**	**5.287**	**4.685**
Criconematidae	Tripyla	**-0.753**	**-0.420**	**5.287**	**5.162**
Criconematidae	Dendrolaelaps	**-0.753**	**0.027**	**5.287**	**3.570**
Criconematidae	Hypoaspis	**-0.753**	**0.334**	**5.287**	**3.871**
Criconematidae	Pergamasus	**-0.753**	**1.081**	**5.287**	**3.269**
Criconematidae	Dorylaimoidea	**-0.753**	**-0.604**	**5.287**	**5.162**
Criconematidae	Eudorylaimus	**-0.753**	**-0.166**	**5.287**	**4.685**
Criconematidae	Pungentus	**-0.753**	**0.263**	**5.287**	**4.685**
Criconematidae	Thornematidae	**-0.753**	**-0.470**	**5.287**	**4.685**
Criconematidae	Eupodes	**-0.753**	**0.005**	**5.287**	**3.746**
Criconematidae	Scutacarus	**-0.753**	**-0.608**	**5.287**	**4.114**
Criconematidae	Tarsonemus	**-0.753**	**-0.701**	**5.287**	**3.570**
Filenchus	Anatonchus	**-1.033**	**0.406**	**4.986**	**4.685**
Filenchus	Mononchus	**-1.033**	**-0.938**	**4.986**	**4.685**
Filenchus	Mylonchulus	**-1.033**	**-0.005**	**4.986**	**4.685**
Filenchus	Tripyla	**-1.033**	**-0.420**	**4.986**	**5.162**
Filenchus	Dendrolaelaps	**-1.033**	**0.027**	**4.986**	**3.570**
Filenchus	Hypoaspis	**-1.033**	**0.334**	**4.986**	**3.871**
Filenchus	Pergamasus	**-1.033**	**1.081**	**4.986**	**3.269**
Filenchus	Dorylaimoidea	**-1.033**	**-0.604**	**4.986**	**5.162**
Filenchus	Eudorylaimus	**-1.033**	**-0.166**	**4.986**	**4.685**
Filenchus	Pungentus	**-1.033**	**0.263**	**4.986**	**4.685**
Filenchus	Thornematidae	**-1.033**	**-0.470**	**4.986**	**4.685**
Filenchus	Eupodes	**-1.033**	**0.005**	**4.986**	**3.746**
Filenchus	Scutacarus	**-1.033**	**-0.608**	**4.986**	**4.114**
Filenchus	Tarsonemus	**-1.033**	**-0.701**	**4.986**	**3.570**
Helicotylenchus	Anatonchus	**-0.792**	**0.406**	**5.383**	**4.685**
Helicotylenchus	Mononchus	**-0.792**	**-0.938**	**5.383**	**4.685**
Helicotylenchus	Mylonchulus	**-0.792**	**-0.005**	**5.383**	**4.685**
Helicotylenchus	Tripyla	**-0.792**	**-0.420**	**5.383**	**5.162**
Helicotylenchus	Dendrolaelaps	**-0.792**	**0.027**	**5.383**	**3.570**
Helicotylenchus	Hypoaspis	**-0.792**	**0.334**	**5.383**	**3.871**
Helicotylenchus	Pergamasus	**-0.792**	**1.081**	**5.383**	**3.269**
Helicotylenchus	Dorylaimoidea	**-0.792**	**-0.604**	**5.383**	**5.162**
Helicotylenchus	Eudorylaimus	**-0.792**	**-0.166**	**5.383**	**4.685**
Helicotylenchus	Pungentus	**-0.792**	**0.263**	**5.383**	**4.685**
Helicotylenchus	Thornematidae	**-0.792**	**-0.470**	**5.383**	**4.685**
Helicotylenchus	Eupodes	**-0.792**	**0.005**	**5.383**	**3.746**
Helicotylenchus	Scutacarus	**-0.792**	**-0.608**	**5.383**	**4.114**
Helicotylenchus	Tarsonemus	**-0.792**	**-0.701**	**5.383**	**3.570**
Heterodera	Anatonchus	**-0.883**	**0.406**	**5.463**	**4.685**
Heterodera	Mononchus	**-0.883**	**-0.938**	**5.463**	**4.685**
Heterodera	Mylonchulus	**-0.883**	**-0.005**	**5.463**	**4.685**
Heterodera	Tripyla	**-0.883**	**-0.420**	**5.463**	**5.162**
Heterodera	Dendrolaelaps	**-0.883**	**0.027**	**5.463**	**3.570**
Heterodera	Hypoaspis	**-0.883**	**0.334**	**5.463**	**3.871**
Heterodera	Pergamasus	**-0.883**	**1.081**	**5.463**	**3.269**
Heterodera	Dorylaimoidea	**-0.883**	**-0.604**	**5.463**	**5.162**

Heterodera	Eudorylaimus	**-0.883**	**-0.166**	**5.463**	**4.685**
Heterodera	Pungentus	**-0.883**	**0.263**	**5.463**	**4.685**
Heterodera	Thornematidae	**-0.883**	**-0.470**	**5.463**	**4.685**
Heterodera	Eupodes	**-0.883**	**0.005**	**5.463**	**3.746**
Heterodera	Scutacarus	**-0.883**	**-0.608**	**5.463**	**4.114**
Heterodera	Tarsonemus	**-0.883**	**-0.701**	**5.463**	**3.570**
Meloidogyne	Anatonchus	**-1.287**	**0.406**	**5.162**	**4.685**
Meloidogyne	Mononchus	**-1.287**	**-0.938**	**5.162**	**4.685**
Meloidogyne	Mylonchulus	**-1.287**	**-0.005**	**5.162**	**4.685**
Meloidogyne	Tripyla	**-1.287**	**-0.420**	**5.162**	**5.162**
Meloidogyne	Dendrolaelaps	**-1.287**	**0.027**	**5.162**	**3.570**
Meloidogyne	Hypoaspis	**-1.287**	**0.334**	**5.162**	**3.871**
Meloidogyne	Pergamasus	**-1.287**	**1.081**	**5.162**	**3.269**
Meloidogyne	Dorylaimoidea	**-1.287**	**-0.604**	**5.162**	**5.162**
Meloidogyne	Eudorylaimus	**-1.287**	**-0.166**	**5.162**	**4.685**
Meloidogyne	Pungentus	**-1.287**	**0.263**	**5.162**	**4.685**
Meloidogyne	Thornematidae	**-1.287**	**-0.470**	**5.162**	**4.685**
Meloidogyne	Eupodes	**-1.287**	**0.005**	**5.162**	**3.746**
Meloidogyne	Scutacarus	**-1.287**	**-0.608**	**5.162**	**4.114**
Meloidogyne	Tarsonemus	**-1.287**	**-0.701**	**5.162**	**3.570**
Paratylenchus	Anatonchus	**-1.244**	**0.406**	**4.986**	**4.685**
Paratylenchus	Mononchus	**-1.244**	**-0.938**	**4.986**	**4.685**
Paratylenchus	Mylonchulus	**-1.244**	**-0.005**	**4.986**	**4.685**
Paratylenchus	Tripyla	**-1.244**	**-0.420**	**4.986**	**5.162**
Paratylenchus	Dendrolaelaps	**-1.244**	**0.027**	**4.986**	**3.570**
Paratylenchus	Hypoaspis	**-1.244**	**0.334**	**4.986**	**3.871**
Paratylenchus	Pergamasus	**-1.244**	**1.081**	**4.986**	**3.269**
Paratylenchus	Dorylaimoidea	**-1.244**	**-0.604**	**4.986**	**5.162**
Paratylenchus	Eudorylaimus	**-1.244**	**-0.166**	**4.986**	**4.685**
Paratylenchus	Pungentus	**-1.244**	**0.263**	**4.986**	**4.685**
Paratylenchus	Thornematidae	**-1.244**	**-0.470**	**4.986**	**4.685**
Paratylenchus	Eupodes	**-1.244**	**0.005**	**4.986**	**3.746**
Paratylenchus	Scutacarus	**-1.244**	**-0.608**	**4.986**	**4.114**
Paratylenchus	Tarsonemus	**-1.244**	**-0.701**	**4.986**	**3.570**
Pratylenchus	Anatonchus	**-1.226**	**0.406**	**5.162**	**4.685**
Pratylenchus	Mononchus	**-1.226**	**-0.938**	**5.162**	**4.685**
Pratylenchus	Mylonchulus	**-1.226**	**-0.005**	**5.162**	**4.685**
Pratylenchus	Tripyla	**-1.226**	**-0.420**	**5.162**	**5.162**
Pratylenchus	Dendrolaelaps	**-1.226**	**0.027**	**5.162**	**3.570**
Pratylenchus	Hypoaspis	**-1.226**	**0.334**	**5.162**	**3.871**
Pratylenchus	Pergamasus	**-1.226**	**1.081**	**5.162**	**3.269**
Pratylenchus	Dorylaimoidea	**-1.226**	**-0.604**	**5.162**	**5.162**
Pratylenchus	Eudorylaimus	**-1.226**	**-0.166**	**5.162**	**4.685**
Pratylenchus	Pungentus	**-1.226**	**0.263**	**5.162**	**4.685**
Pratylenchus	Thornematidae	**-1.226**	**-0.470**	**5.162**	**4.685**
Pratylenchus	Eupodes	**-1.226**	**0.005**	**5.162**	**3.746**
Pratylenchus	Scutacarus	**-1.226**	**-0.608**	**5.162**	**4.114**
Pratylenchus	Tarsonemus	**-1.226**	**-0.701**	**5.162**	**3.570**
Tylenchorhynchus	Anatonchus	**-0.664**	**0.406**	**4.685**	**4.685**
Tylenchorhynchus	Mononchus	**-0.664**	**-0.938**	**4.685**	**4.685**
Tylenchorhynchus	Mylonchulus	**-0.664**	**-0.005**	**4.685**	**4.685**

Tylenchorhynchus	Tripyla	**-0.664**	**-0.420**	**4.685**	**5.162**
Tylenchorhynchus	Dendrolaelaps	**-0.664**	**0.027**	**4.685**	**3.570**
Tylenchorhynchus	Hypoaspis	**-0.664**	**0.334**	**4.685**	**3.871**
Tylenchorhynchus	Pergamasus	**-0.664**	**1.081**	**4.685**	**3.269**
Tylenchorhynchus	Dorylaimoidea	**-0.664**	**-0.604**	**4.685**	**5.162**
Tylenchorhynchus	Eudorylaimus	**-0.664**	**-0.166**	**4.685**	**4.685**
Tylenchorhynchus	Pungentus	**-0.664**	**0.263**	**4.685**	**4.685**
Tylenchorhynchus	Thornematidae	**-0.664**	**-0.470**	**4.685**	**4.685**
Tylenchorhynchus	Eupodes	**-0.664**	**0.005**	**4.685**	**3.746**
Tylenchorhynchus	Scutacarus	**-0.664**	**-0.608**	**4.685**	**4.114**
Tylenchorhynchus	Tarsonemus	**-0.664**	**-0.701**	**4.685**	**3.570**
Galumna	Bdella	**0.474**	**0.816**	**3.269**	**3.269**
Galumna	Dendrolaelaps	**0.474**	**0.027**	**3.269**	**3.570**
Galumna	Hypoaspis	**0.474**	**0.334**	**3.269**	**3.871**
Galumna	Pergamasus	**0.474**	**1.081**	**3.269**	**3.269**
Galumna	Dorylaimoidea	**0.474**	**-0.604**	**3.269**	**5.162**
Galumna	Eudorylaimus	**0.474**	**-0.166**	**3.269**	**4.685**
Galumna	Pungentus	**0.474**	**0.263**	**3.269**	**4.685**
Galumna	Thornematidae	**0.474**	**-0.470**	**3.269**	**4.685**
Galumna	Eupodes	**0.474**	**0.005**	**3.269**	**3.746**
Galumna	Scutacarus	**0.474**	**-0.608**	**3.269**	**4.114**
Galumna	Tarsonemus	**0.474**	**-0.701**	**3.269**	**3.570**
Galumna	Pyemotes	**0.474**	**-0.608**	**3.269**	**3.269**
Platynothrus	Bdella	**0.710**	**0.816**	**3.871**	**3.269**
Platynothrus	Dendrolaelaps	**0.710**	**0.027**	**3.871**	**3.570**
Platynothrus	Hypoaspis	**0.710**	**0.334**	**3.871**	**3.871**
Platynothrus	Pergamasus	**0.710**	**1.081**	**3.871**	**3.269**
Platynothrus	Dorylaimoidea	**0.710**	**-0.604**	**3.871**	**5.162**
Platynothrus	Eudorylaimus	**0.710**	**-0.166**	**3.871**	**4.685**
Platynothrus	Pungentus	**0.710**	**0.263**	**3.871**	**4.685**
Platynothrus	Thornematidae	**0.710**	**-0.470**	**3.871**	**4.685**
Platynothrus	Eupodes	**0.710**	**0.005**	**3.871**	**3.746**
Platynothrus	Scutacarus	**0.710**	**-0.608**	**3.871**	**4.114**
Platynothrus	Tarsonemus	**0.710**	**-0.701**	**3.871**	**3.570**
Platynothrus	Pyemotes	**0.710**	**-0.608**	**3.871**	**3.269**
Tydeidae	Bdella	**-0.608**	**0.816**	**4.172**	**3.269**
Tydeidae	Dendrolaelaps	**-0.608**	**0.027**	**4.172**	**3.570**
Tydeidae	Hypoaspis	**-0.608**	**0.334**	**4.172**	**3.871**
Tydeidae	Pergamasus	**-0.608**	**1.081**	**4.172**	**3.269**
Tydeidae	Dorylaimoidea	**-0.608**	**-0.604**	**4.172**	**5.162**
Tydeidae	Eudorylaimus	**-0.608**	**-0.166**	**4.172**	**4.685**
Tydeidae	Pungentus	**-0.608**	**0.263**	**4.172**	**4.685**
Tydeidae	Thornematidae	**-0.608**	**-0.470**	**4.172**	**4.685**
Tydeidae	Eupodes	**-0.608**	**0.005**	**4.172**	**3.746**
Tydeidae	Scutacarus	**-0.608**	**-0.608**	**4.172**	**4.114**
Tydeidae	Tarsonemus	**-0.608**	**-0.701**	**4.172**	**3.570**
Tydeidae	Pyemotes	**-0.608**	**-0.608**	**4.172**	**3.269**
Sminthuridae	Dendrolaelaps	**-0.608**	**0.027**	**3.871**	**3.570**
Sminthuridae	Hypoaspis	**-0.608**	**0.334**	**3.871**	**3.871**
Sminthuridae	Pergamasus	**-0.608**	**1.081**	**3.871**	**3.269**
Sminthuridae	Dorylaimoidea	**-0.608**	**-0.604**	**3.871**	**5.162**

Sminthuridae	Eudorylaimus	**-0.608**	**-0.166**	**3.871**	**4.685**
Sminthuridae	Pungentus	**-0.608**	**0.263**	**3.871**	**4.685**
Sminthuridae	Thornematidae	**-0.608**	**-0.470**	**3.871**	**4.685**
Sminthuridae	Eupodes	**-0.608**	**0.005**	**3.871**	**3.746**
Sminthuridae	Scutacarus	**-0.608**	**-0.608**	**3.871**	**4.114**
Sminthuridae	Tarsonemus	**-0.608**	**-0.701**	**3.871**	**3.570**
Sminthurinus	Dendrolaelaps	**0.618**	**0.027**	**3.269**	**3.570**
Sminthurinus	Hypoaspis	**0.618**	**0.334**	**3.269**	**3.871**
Sminthurinus	Pergamasus	**0.618**	**1.081**	**3.269**	**3.269**
Sminthurinus	Dorylaimoidea	**0.618**	**-0.604**	**3.269**	**5.162**
Sminthurinus	Eudorylaimus	**0.618**	**-0.166**	**3.269**	**4.685**
Sminthurinus	Pungentus	**0.618**	**0.263**	**3.269**	**4.685**
Sminthurinus	Thornematidae	**0.618**	**-0.470**	**3.269**	**4.685**
Sminthurinus	Eupodes	**0.618**	**0.005**	**3.269**	**3.746**
Sminthurinus	Scutacarus	**0.618**	**-0.608**	**3.269**	**4.114**
Sminthurinus	Tarsonemus	**0.618**	**-0.701**	**3.269**	**3.570**
Aphelenchoides	Anatonchus	**-1.496**	**0.406**	**5.162**	**4.685**
Aphelenchoides	Mononchus	**-1.496**	**-0.938**	**5.162**	**4.685**
Aphelenchoides	Mylonchulus	**-1.496**	**-0.005**	**5.162**	**4.685**
Aphelenchoides	Tripyla	**-1.496**	**-0.420**	**5.162**	**5.162**
Aphelenchoides	Dendrolaelaps	**-1.496**	**0.027**	**5.162**	**3.570**
Aphelenchoides	Hypoaspis	**-1.496**	**0.334**	**5.162**	**3.871**
Aphelenchoides	Pergamasus	**-1.496**	**1.081**	**5.162**	**3.269**
Aphelenchoides	Dorylaimoidea	**-1.496**	**-0.604**	**5.162**	**5.162**
Aphelenchoides	Eudorylaimus	**-1.496**	**-0.166**	**5.162**	**4.685**
Aphelenchoides	Pungentus	**-1.496**	**0.263**	**5.162**	**4.685**
Aphelenchoides	Thornematidae	**-1.496**	**-0.470**	**5.162**	**4.685**
Aphelenchoides	Eupodes	**-1.496**	**0.005**	**5.162**	**3.746**
Aphelenchoides	Scutacarus	**-1.496**	**-0.608**	**5.162**	**4.114**
Aphelenchoides	Tarsonemus	**-1.496**	**-0.701**	**5.162**	**3.570**
Tylenchidae	Anatonchus	**-1.360**	**0.406**	**6.530**	**4.685**
Tylenchidae	Mononchus	**-1.360**	**-0.938**	**6.530**	**4.685**
Tylenchidae	Mylonchulus	**-1.360**	**-0.005**	**6.530**	**4.685**
Tylenchidae	Tripyla	**-1.360**	**-0.420**	**6.530**	**5.162**
Tylenchidae	Dendrolaelaps	**-1.360**	**0.027**	**6.530**	**3.570**
Tylenchidae	Hypoaspis	**-1.360**	**0.334**	**6.530**	**3.871**
Tylenchidae	Pergamasus	**-1.360**	**1.081**	**6.530**	**3.269**
Tylenchidae	Dorylaimoidea	**-1.360**	**-0.604**	**6.530**	**5.162**
Tylenchidae	Eudorylaimus	**-1.360**	**-0.166**	**6.530**	**4.685**
Tylenchidae	Pungentus	**-1.360**	**0.263**	**6.530**	**4.685**
Tylenchidae	Thornematidae	**-1.360**	**-0.470**	**6.530**	**4.685**
Tylenchidae	Eupodes	**-1.360**	**0.005**	**6.530**	**3.746**
Tylenchidae	Scutacarus	**-1.360**	**-0.608**	**6.530**	**4.114**
Tylenchidae	Tarsonemus	**-1.360**	**-0.701**	**6.530**	**3.570**
Brachychthoniidae	Bdella	**-0.586**	**0.816**	**3.871**	**3.269**
Brachychthoniidae	Dendrolaelaps	**-0.586**	**0.027**	**3.871**	**3.570**
Brachychthoniidae	Hypoaspis	**-0.586**	**0.334**	**3.871**	**3.871**
Brachychthoniidae	Pergamasus	**-0.586**	**1.081**	**3.871**	**3.269**
Brachychthoniidae	Dorylaimoidea	**-0.586**	**-0.604**	**3.871**	**5.162**
Brachychthoniidae	Eudorylaimus	**-0.586**	**-0.166**	**3.871**	**4.685**
Brachychthoniidae	Pungentus	**-0.586**	**0.263**	**3.871**	**4.685**

Brachychthoniidae	Thornematidae	**-0.586**	**-0.470**	**3.871**	**4.685**
Brachychthoniidae	Eupodes	**-0.586**	**0.005**	**3.871**	**3.746**
Brachychthoniidae	Scutacarus	**-0.586**	**-0.608**	**3.871**	**4.114**
Brachychthoniidae	Tarsonemus	**-0.586**	**-0.701**	**3.871**	**3.570**
Brachychthoniidae	Pyemotes	**-0.586**	**-0.608**	**3.871**	**3.269**
Medioppia	Bdella	**-0.235**	**0.816**	**3.570**	**3.269**
Medioppia	Dendrolaelaps	**-0.235**	**0.027**	**3.570**	**3.570**
Medioppia	Hypoaspis	**-0.235**	**0.334**	**3.570**	**3.871**
Medioppia	Pergamasus	**-0.235**	**1.081**	**3.570**	**3.269**
Medioppia	Dorylaimoidea	**-0.235**	**-0.604**	**3.570**	**5.162**
Medioppia	Eudorylaimus	**-0.235**	**-0.166**	**3.570**	**4.685**
Medioppia	Pungentus	**-0.235**	**0.263**	**3.570**	**4.685**
Medioppia	Thornematidae	**-0.235**	**-0.470**	**3.570**	**4.685**
Medioppia	Eupodes	**-0.235**	**0.005**	**3.570**	**3.746**
Medioppia	Scutacarus	**-0.235**	**-0.608**	**3.570**	**4.114**
Medioppia	Tarsonemus	**-0.235**	**-0.701**	**3.570**	**3.570**
Medioppia	Pyemotes	**-0.235**	**-0.608**	**3.570**	**3.269**
Microppia	Bdella	**-0.544**	**0.816**	**4.114**	**3.269**
Microppia	Dendrolaelaps	**-0.544**	**0.027**	**4.114**	**3.570**
Microppia	Hypoaspis	**-0.544**	**0.334**	**4.114**	**3.871**
Microppia	Pergamasus	**-0.544**	**1.081**	**4.114**	**3.269**
Microppia	Dorylaimoidea	**-0.544**	**-0.604**	**4.114**	**5.162**
Microppia	Eudorylaimus	**-0.544**	**-0.166**	**4.114**	**4.685**
Microppia	Pungentus	**-0.544**	**0.263**	**4.114**	**4.685**
Microppia	Thornematidae	**-0.544**	**-0.470**	**4.114**	**4.685**
Microppia	Eupodes	**-0.544**	**0.005**	**4.114**	**3.746**
Microppia	Scutacarus	**-0.544**	**-0.608**	**4.114**	**4.114**
Microppia	Tarsonemus	**-0.544**	**-0.701**	**4.114**	**3.570**
Microppia	Pyemotes	**-0.544**	**-0.608**	**4.114**	**3.269**
Minunthozetes	Bdella	**-0.249**	**0.816**	**3.570**	**3.269**
Minunthozetes	Dendrolaelaps	**-0.249**	**0.027**	**3.570**	**3.570**
Minunthozetes	Hypoaspis	**-0.249**	**0.334**	**3.570**	**3.871**
Minunthozetes	Pergamasus	**-0.249**	**1.081**	**3.570**	**3.269**
Minunthozetes	Dorylaimoidea	**-0.249**	**-0.604**	**3.570**	**5.162**
Minunthozetes	Eudorylaimus	**-0.249**	**-0.166**	**3.570**	**4.685**
Minunthozetes	Pungentus	**-0.249**	**0.263**	**3.570**	**4.685**
Minunthozetes	Thornematidae	**-0.249**	**-0.470**	**3.570**	**4.685**
Minunthozetes	Eupodes	**-0.249**	**0.005**	**3.570**	**3.746**
Minunthozetes	Scutacarus	**-0.249**	**-0.608**	**3.570**	**4.114**
Minunthozetes	Tarsonemus	**-0.249**	**-0.701**	**3.570**	**3.570**
Minunthozetes	Pyemotes	**-0.249**	**-0.608**	**3.570**	**3.269**
Oppiella	Bdella	**-0.447**	**0.816**	**3.871**	**3.269**
Oppiella	Dendrolaelaps	**-0.447**	**0.027**	**3.871**	**3.570**
Oppiella	Hypoaspis	**-0.447**	**0.334**	**3.871**	**3.871**
Oppiella	Pergamasus	**-0.447**	**1.081**	**3.871**	**3.269**
Oppiella	Dorylaimoidea	**-0.447**	**-0.604**	**3.871**	**5.162**
Oppiella	Eudorylaimus	**-0.447**	**-0.166**	**3.871**	**4.685**
Oppiella	Pungentus	**-0.447**	**0.263**	**3.871**	**4.685**
Oppiella	Thornematidae	**-0.447**	**-0.470**	**3.871**	**4.685**
Oppiella	Eupodes	**-0.447**	**0.005**	**3.871**	**3.746**
Oppiella	Scutacarus	**-0.447**	**-0.608**	**3.871**	**4.114**

Oppiella	Tarsonemus	**-0.447**	**-0.701**	**3.871**	**3.570**
Oppiella	Pyemotes	**-0.447**	**-0.608**	**3.871**	**3.269**
Pygmephorus	Bdella	**-0.376**	**0.816**	**3.746**	**3.269**
Pygmephorus	Dendrolaelaps	**-0.376**	**0.027**	**3.746**	**3.570**
Pygmephorus	Hypoaspis	**-0.376**	**0.334**	**3.746**	**3.871**
Pygmephorus	Pergamasus	**-0.376**	**1.081**	**3.746**	**3.269**
Pygmephorus	Dorylaimoidea	**-0.376**	**-0.604**	**3.746**	**5.162**
Pygmephorus	Eudorylaimus	**-0.376**	**-0.166**	**3.746**	**4.685**
Pygmephorus	Pungentus	**-0.376**	**0.263**	**3.746**	**4.685**
Pygmephorus	Thornematidae	**-0.376**	**-0.470**	**3.746**	**4.685**
Pygmephorus	Eupodes	**-0.376**	**0.005**	**3.746**	**3.746**
Pygmephorus	Scutacarus	**-0.376**	**-0.608**	**3.746**	**4.114**
Pygmephorus	Tarsonemus	**-0.376**	**-0.701**	**3.746**	**3.570**
Pygmephorus	Pyemotes	**-0.376**	**-0.608**	**3.746**	**3.269**
Speleorchestes	Bdella	**-0.249**	**0.816**	**3.570**	**3.269**
Speleorchestes	Dendrolaelaps	**-0.249**	**0.027**	**3.570**	**3.570**
Speleorchestes	Hypoaspis	**-0.249**	**0.334**	**3.570**	**3.871**
Speleorchestes	Pergamasus	**-0.249**	**1.081**	**3.570**	**3.269**
Speleorchestes	Dorylaimoidea	**-0.249**	**-0.604**	**3.570**	**5.162**
Speleorchestes	Eudorylaimus	**-0.249**	**-0.166**	**3.570**	**4.685**
Speleorchestes	Pungentus	**-0.249**	**0.263**	**3.570**	**4.685**
Speleorchestes	Thornematidae	**-0.249**	**-0.470**	**3.570**	**4.685**
Speleorchestes	Eupodes	**-0.249**	**0.005**	**3.570**	**3.746**
Speleorchestes	Scutacarus	**-0.249**	**-0.608**	**3.570**	**4.114**
Speleorchestes	Tarsonemus	**-0.249**	**-0.701**	**3.570**	**3.570**
Speleorchestes	Pyemotes	**-0.249**	**-0.608**	**3.570**	**3.269**
Trichoribates	Bdella	**0.474**	**0.816**	**3.570**	**3.269**
Trichoribates	Dendrolaelaps	**0.474**	**0.027**	**3.570**	**3.570**
Trichoribates	Hypoaspis	**0.474**	**0.334**	**3.570**	**3.871**
Trichoribates	Pergamasus	**0.474**	**1.081**	**3.570**	**3.269**
Trichoribates	Dorylaimoidea	**0.474**	**-0.604**	**3.570**	**5.162**
Trichoribates	Eudorylaimus	**0.474**	**-0.166**	**3.570**	**4.685**
Trichoribates	Pungentus	**0.474**	**0.263**	**3.570**	**4.685**
Trichoribates	Thornematidae	**0.474**	**-0.470**	**3.570**	**4.685**
Trichoribates	Eupodes	**0.474**	**0.005**	**3.570**	**3.746**
Trichoribates	Scutacarus	**0.474**	**-0.608**	**3.570**	**4.114**
Trichoribates	Tarsonemus	**0.474**	**-0.701**	**3.570**	**3.570**
Trichoribates	Pyemotes	**0.474**	**-0.608**	**3.570**	**3.269**
Tyrophagus	Bdella	**0.005**	**0.816**	**3.746**	**3.269**
Tyrophagus	Dendrolaelaps	**0.005**	**0.027**	**3.746**	**3.570**
Tyrophagus	Hypoaspis	**0.005**	**0.334**	**3.746**	**3.871**
Tyrophagus	Pergamasus	**0.005**	**1.081**	**3.746**	**3.269**
Tyrophagus	Dorylaimoidea	**0.005**	**-0.604**	**3.746**	**5.162**
Tyrophagus	Eudorylaimus	**0.005**	**-0.166**	**3.746**	**4.685**
Tyrophagus	Pungentus	**0.005**	**0.263**	**3.746**	**4.685**
Tyrophagus	Thornematidae	**0.005**	**-0.470**	**3.746**	**4.685**
Tyrophagus	Eupodes	**0.005**	**0.005**	**3.746**	**3.746**
Tyrophagus	Scutacarus	**0.005**	**-0.608**	**3.746**	**4.114**
Tyrophagus	Tarsonemus	**0.005**	**-0.701**	**3.746**	**3.570**
Tyrophagus	Pyemotes	**0.005**	**-0.608**	**3.746**	**3.269**
Parisotoma	Dendrolaelaps	**0.722**	**0.027**	**3.269**	**3.570**

Parisotoma	Hypoaspis	**0.722**	**0.334**	**3.269**	**3.871**
Parisotoma	Pergamasus	**0.722**	**1.081**	**3.269**	**3.269**
Parisotoma	Dorylaimoidea	**0.722**	**-0.604**	**3.269**	**5.162**
Parisotoma	Eudorylaimus	**0.722**	**-0.166**	**3.269**	**4.685**
Parisotoma	Pungentus	**0.722**	**0.263**	**3.269**	**4.685**
Parisotoma	Thornematidae	**0.722**	**-0.470**	**3.269**	**4.685**
Parisotoma	Eupodes	**0.722**	**0.005**	**3.269**	**3.746**
Parisotoma	Scutacarus	**0.722**	**-0.608**	**3.269**	**4.114**
Parisotoma	Tarsonemus	**0.722**	**-0.701**	**3.269**	**3.570**
Achaeta	Dendrolaelaps	**0.831**	**0.027**	**1.771**	**3.570**
Achaeta	Hypoaspis	**0.831**	**0.334**	**1.771**	**3.871**
Achaeta	Pergamasus	**0.831**	**1.081**	**1.771**	**3.269**
Achaeta	Dorylaimoidea	**0.831**	**-0.604**	**1.771**	**5.162**
Achaeta	Eudorylaimus	**0.831**	**-0.166**	**1.771**	**4.685**
Achaeta	Pungentus	**0.831**	**0.263**	**1.771**	**4.685**
Achaeta	Thornematidae	**0.831**	**-0.470**	**1.771**	**4.685**
Achaeta	Eupodes	**0.831**	**0.005**	**1.771**	**3.746**
Achaeta	Scutacarus	**0.831**	**-0.608**	**1.771**	**4.114**
Achaeta	Tarsonemus	**0.831**	**-0.701**	**1.771**	**3.570**
Cognettia	Dendrolaelaps	**1.215**	**0.027**	**2.849**	**3.570**
Cognettia	Hypoaspis	**1.215**	**0.334**	**2.849**	**3.871**
Cognettia	Pergamasus	**1.215**	**1.081**	**2.849**	**3.269**
Cognettia	Dorylaimoidea	**1.215**	**-0.604**	**2.849**	**5.162**
Cognettia	Eudorylaimus	**1.215**	**-0.166**	**2.849**	**4.685**
Cognettia	Pungentus	**1.215**	**0.263**	**2.849**	**4.685**
Cognettia	Thornematidae	**1.215**	**-0.470**	**2.849**	**4.685**
Cognettia	Eupodes	**1.215**	**0.005**	**2.849**	**3.746**
Cognettia	Scutacarus	**1.215**	**-0.608**	**2.849**	**4.114**
Cognettia	Tarsonemus	**1.215**	**-0.701**	**2.849**	**3.570**
Fridericia	Dendrolaelaps	**2.158**	**0.027**	**2.975**	**3.570**
Fridericia	Hypoaspis	**2.158**	**0.334**	**2.975**	**3.871**
Fridericia	Pergamasus	**2.158**	**1.081**	**2.975**	**3.269**
Fridericia	Dorylaimoidea	**2.158**	**-0.604**	**2.975**	**5.162**
Fridericia	Eudorylaimus	**2.158**	**-0.166**	**2.975**	**4.685**
Fridericia	Pungentus	**2.158**	**0.263**	**2.975**	**4.685**
Fridericia	Thornematidae	**2.158**	**-0.470**	**2.975**	**4.685**
Fridericia	Eupodes	**2.158**	**0.005**	**2.975**	**3.746**
Fridericia	Scutacarus	**2.158**	**-0.608**	**2.975**	**4.114**
Fridericia	Tarsonemus	**2.158**	**-0.701**	**2.975**	**3.570**
Acrobeloides	Anatonchus	**-1.171**	**0.406**	**5.463**	**4.685**
Acrobeloides	Mononchus	**-1.171**	**-0.938**	**5.463**	**4.685**
Acrobeloides	Mylonchulus	**-1.171**	**-0.005**	**5.463**	**4.685**
Acrobeloides	Tripyla	**-1.171**	**-0.420**	**5.463**	**5.162**
Acrobeloides	Dendrolaelaps	**-1.171**	**0.027**	**5.463**	**3.570**
Acrobeloides	Hypoaspis	**-1.171**	**0.334**	**5.463**	**3.871**
Acrobeloides	Pergamasus	**-1.171**	**1.081**	**5.463**	**3.269**
Acrobeloides	Dorylaimoidea	**-1.171**	**-0.604**	**5.463**	**5.162**
Acrobeloides	Eudorylaimus	**-1.171**	**-0.166**	**5.463**	**4.685**
Acrobeloides	Pungentus	**-1.171**	**0.263**	**5.463**	**4.685**
Acrobeloides	Thornematidae	**-1.171**	**-0.470**	**5.463**	**4.685**
Acrobeloides	Eupodes	**-1.171**	**0.005**	**5.463**	**3.746**

Acrobeloides	Scutacarus	**-1.171**	**-0.608**	**5.463**	**4.114**
Acrobeloides	Tarsonemus	**-1.171**	**-0.701**	**5.463**	**3.570**
Anaplectus	Anatonchus	**-0.519**	**0.406**	**4.685**	**4.685**
Anaplectus	Mononchus	**-0.519**	**-0.938**	**4.685**	**4.685**
Anaplectus	Mylonchulus	**-0.519**	**-0.005**	**4.685**	**4.685**
Anaplectus	Tripyla	**-0.519**	**-0.420**	**4.685**	**5.162**
Anaplectus	Dendrolaelaps	**-0.519**	**0.027**	**4.685**	**3.570**
Anaplectus	Hypoaspis	**-0.519**	**0.334**	**4.685**	**3.871**
Anaplectus	Pergamasus	**-0.519**	**1.081**	**4.685**	**3.269**
Anaplectus	Dorylaimoidea	**-0.519**	**-0.604**	**4.685**	**5.162**
Anaplectus	Eudorylaimus	**-0.519**	**-0.166**	**4.685**	**4.685**
Anaplectus	Pungentus	**-0.519**	**0.263**	**4.685**	**4.685**
Anaplectus	Thornematidae	**-0.519**	**-0.470**	**4.685**	**4.685**
Anaplectus	Eupodes	**-0.519**	**0.005**	**4.685**	**3.746**
Anaplectus	Scutacarus	**-0.519**	**-0.608**	**4.685**	**4.114**
Anaplectus	Tarsonemus	**-0.519**	**-0.701**	**4.685**	**3.570**
Cephalobidae	Anatonchus	**-1.055**	**0.406**	**4.986**	**4.685**
Cephalobidae	Mononchus	**-1.055**	**-0.938**	**4.986**	**4.685**
Cephalobidae	Mylonchulus	**-1.055**	**-0.005**	**4.986**	**4.685**
Cephalobidae	Tripyla	**-1.055**	**-0.420**	**4.986**	**5.162**
Cephalobidae	Dendrolaelaps	**-1.055**	**0.027**	**4.986**	**3.570**
Cephalobidae	Hypoaspis	**-1.055**	**0.334**	**4.986**	**3.871**
Cephalobidae	Pergamasus	**-1.055**	**1.081**	**4.986**	**3.269**
Cephalobidae	Dorylaimoidea	**-1.055**	**-0.604**	**4.986**	**5.162**
Cephalobidae	Eudorylaimus	**-1.055**	**-0.166**	**4.986**	**4.685**
Cephalobidae	Pungentus	**-1.055**	**0.263**	**4.986**	**4.685**
Cephalobidae	Thornematidae	**-1.055**	**-0.470**	**4.986**	**4.685**
Cephalobidae	Eupodes	**-1.055**	**0.005**	**4.986**	**3.746**
Cephalobidae	Scutacarus	**-1.055**	**-0.608**	**4.986**	**4.114**
Cephalobidae	Tarsonemus	**-1.055**	**-0.701**	**4.986**	**3.570**
Eucephalobus	Anatonchus	**-0.855**	**0.406**	**5.383**	**4.685**
Eucephalobus	Mononchus	**-0.855**	**-0.938**	**5.383**	**4.685**
Eucephalobus	Mylonchulus	**-0.855**	**-0.005**	**5.383**	**4.685**
Eucephalobus	Tripyla	**-0.855**	**-0.420**	**5.383**	**5.162**
Eucephalobus	Dendrolaelaps	**-0.855**	**0.027**	**5.383**	**3.570**
Eucephalobus	Hypoaspis	**-0.855**	**0.334**	**5.383**	**3.871**
Eucephalobus	Pergamasus	**-0.855**	**1.081**	**5.383**	**3.269**
Eucephalobus	Dorylaimoidea	**-0.855**	**-0.604**	**5.383**	**5.162**
Eucephalobus	Eudorylaimus	**-0.855**	**-0.166**	**5.383**	**4.685**
Eucephalobus	Pungentus	**-0.855**	**0.263**	**5.383**	**4.685**
Eucephalobus	Thornematidae	**-0.855**	**-0.470**	**5.383**	**4.685**
Eucephalobus	Eupodes	**-0.855**	**0.005**	**5.383**	**3.746**
Eucephalobus	Scutacarus	**-0.855**	**-0.608**	**5.383**	**4.114**
Eucephalobus	Tarsonemus	**-0.855**	**-0.701**	**5.383**	**3.570**
Eumonhystera	Anatonchus	**-0.613**	**0.406**	**4.986**	**4.685**
Eumonhystera	Mononchus	**-0.613**	**-0.938**	**4.986**	**4.685**
Eumonhystera	Mylonchulus	**-0.613**	**-0.005**	**4.986**	**4.685**
Eumonhystera	Tripyla	**-0.613**	**-0.420**	**4.986**	**5.162**
Eumonhystera	Dendrolaelaps	**-0.613**	**0.027**	**4.986**	**3.570**
Eumonhystera	Hypoaspis	**-0.613**	**0.334**	**4.986**	**3.871**
Eumonhystera	Pergamasus	**-0.613**	**1.081**	**4.986**	**3.269**

Eumonhystera	Dorylaimoidea	**-0.613**	**-0.604**	**4.986**	**5.162**
Eumonhystera	Eudorylaimus	**-0.613**	**-0.166**	**4.986**	**4.685**
Eumonhystera	Pungentus	**-0.613**	**0.263**	**4.986**	**4.685**
Eumonhystera	Thornematidae	**-0.613**	**-0.470**	**4.986**	**4.685**
Eumonhystera	Eupodes	**-0.613**	**0.005**	**4.986**	**3.746**
Eumonhystera	Scutacarus	**-0.613**	**-0.608**	**4.986**	**4.114**
Eumonhystera	Tarsonemus	**-0.613**	**-0.701**	**4.986**	**3.570**
Metateratocephalus	Anatonchus	**-1.506**	**0.406**	**4.685**	**4.685**
Metateratocephalus	Mononchus	**-1.506**	**-0.938**	**4.685**	**4.685**
Metateratocephalus	Mylonchulus	**-1.506**	**-0.005**	**4.685**	**4.685**
Metateratocephalus	Tripyla	**-1.506**	**-0.420**	**4.685**	**5.162**
Metateratocephalus	Dendrolaelaps	**-1.506**	**0.027**	**4.685**	**3.570**
Metateratocephalus	Hypoaspis	**-1.506**	**0.334**	**4.685**	**3.871**
Metateratocephalus	Pergamasus	**-1.506**	**1.081**	**4.685**	**3.269**
Metateratocephalus	Dorylaimoidea	**-1.506**	**-0.604**	**4.685**	**5.162**
Metateratocephalus	Eudorylaimus	**-1.506**	**-0.166**	**4.685**	**4.685**
Metateratocephalus	Pungentus	**-1.506**	**0.263**	**4.685**	**4.685**
Metateratocephalus	Thornematidae	**-1.506**	**-0.470**	**4.685**	**4.685**
Metateratocephalus	Eupodes	**-1.506**	**0.005**	**4.685**	**3.746**
Metateratocephalus	Scutacarus	**-1.506**	**-0.608**	**4.685**	**4.114**
Metateratocephalus	Tarsonemus	**-1.506**	**-0.701**	**4.685**	**3.570**
Panagrolaimus	Anatonchus	**-0.945**	**0.406**	**5.162**	**4.685**
Panagrolaimus	Mononchus	**-0.945**	**-0.938**	**5.162**	**4.685**
Panagrolaimus	Mylonchulus	**-0.945**	**-0.005**	**5.162**	**4.685**
Panagrolaimus	Tripyla	**-0.945**	**-0.420**	**5.162**	**5.162**
Panagrolaimus	Dendrolaelaps	**-0.945**	**0.027**	**5.162**	**3.570**
Panagrolaimus	Hypoaspis	**-0.945**	**0.334**	**5.162**	**3.871**
Panagrolaimus	Pergamasus	**-0.945**	**1.081**	**5.162**	**3.269**
Panagrolaimus	Dorylaimoidea	**-0.945**	**-0.604**	**5.162**	**5.162**
Panagrolaimus	Eudorylaimus	**-0.945**	**-0.166**	**5.162**	**4.685**
Panagrolaimus	Pungentus	**-0.945**	**0.263**	**5.162**	**4.685**
Panagrolaimus	Thornematidae	**-0.945**	**-0.470**	**5.162**	**4.685**
Panagrolaimus	Eupodes	**-0.945**	**0.005**	**5.162**	**3.746**
Panagrolaimus	Scutacarus	**-0.945**	**-0.608**	**5.162**	**4.114**
Panagrolaimus	Tarsonemus	**-0.945**	**-0.701**	**5.162**	**3.570**
Plectus	Anatonchus	**-0.583**	**0.406**	**5.588**	**4.685**
Plectus	Mononchus	**-0.583**	**-0.938**	**5.588**	**4.685**
Plectus	Mylonchulus	**-0.583**	**-0.005**	**5.588**	**4.685**
Plectus	Tripyla	**-0.583**	**-0.420**	**5.588**	**5.162**
Plectus	Dendrolaelaps	**-0.583**	**0.027**	**5.588**	**3.570**
Plectus	Hypoaspis	**-0.583**	**0.334**	**5.588**	**3.871**
Plectus	Pergamasus	**-0.583**	**1.081**	**5.588**	**3.269**
Plectus	Dorylaimoidea	**-0.583**	**-0.604**	**5.588**	**5.162**
Plectus	Eudorylaimus	**-0.583**	**-0.166**	**5.588**	**4.685**
Plectus	Pungentus	**-0.583**	**0.263**	**5.588**	**4.685**
Plectus	Thornematidae	**-0.583**	**-0.470**	**5.588**	**4.685**
Plectus	Eupodes	**-0.583**	**0.005**	**5.588**	**3.746**
Plectus	Scutacarus	**-0.583**	**-0.608**	**5.588**	**4.114**
Plectus	Tarsonemus	**-0.583**	**-0.701**	**5.588**	**3.570**
Prismatolaimus	Anatonchus	**-1.280**	**0.406**	**4.986**	**4.685**
Prismatolaimus	Mononchus	**-1.280**	**-0.938**	**4.986**	**4.685**

Prismatolaimus	Mylonchulus	**-1.280**	**-0.005**	**4.986**	**4.685**
Prismatolaimus	Tripyla	**-1.280**	**-0.420**	**4.986**	**5.162**
Prismatolaimus	Dendrolaelaps	**-1.280**	**0.027**	**4.986**	**3.570**
Prismatolaimus	Hypoaspis	**-1.280**	**0.334**	**4.986**	**3.871**
Prismatolaimus	Pergamasus	**-1.280**	**1.081**	**4.986**	**3.269**
Prismatolaimus	Dorylaimoidea	**-1.280**	**-0.604**	**4.986**	**5.162**
Prismatolaimus	Eudorylaimus	**-1.280**	**-0.166**	**4.986**	**4.685**
Prismatolaimus	Pungentus	**-1.280**	**0.263**	**4.986**	**4.685**
Prismatolaimus	Thornematidae	**-1.280**	**-0.470**	**4.986**	**4.685**
Prismatolaimus	Eupodes	**-1.280**	**0.005**	**4.986**	**3.746**
Prismatolaimus	Scutacarus	**-1.280**	**-0.608**	**4.986**	**4.114**
Prismatolaimus	Tarsonemus	**-1.280**	**-0.701**	**4.986**	**3.570**
Rhabditidae	Anatonchus	**-0.692**	**0.406**	**5.530**	**4.685**
Rhabditidae	Mononchus	**-0.692**	**-0.938**	**5.530**	**4.685**
Rhabditidae	Mylonchulus	**-0.692**	**-0.005**	**5.530**	**4.685**
Rhabditidae	Tripyla	**-0.692**	**-0.420**	**5.530**	**5.162**
Rhabditidae	Dendrolaelaps	**-0.692**	**0.027**	**5.530**	**3.570**
Rhabditidae	Hypoaspis	**-0.692**	**0.334**	**5.530**	**3.871**
Rhabditidae	Pergamasus	**-0.692**	**1.081**	**5.530**	**3.269**
Rhabditidae	Dorylaimoidea	**-0.692**	**-0.604**	**5.530**	**5.162**
Rhabditidae	Eudorylaimus	**-0.692**	**-0.166**	**5.530**	**4.685**
Rhabditidae	Pungentus	**-0.692**	**0.263**	**5.530**	**4.685**
Rhabditidae	Thornematidae	**-0.692**	**-0.470**	**5.530**	**4.685**
Rhabditidae	Eupodes	**-0.692**	**0.005**	**5.530**	**3.746**
Rhabditidae	Scutacarus	**-0.692**	**-0.608**	**5.530**	**4.114**
Rhabditidae	Tarsonemus	**-0.692**	**-0.701**	**5.530**	**3.570**
Wilsonema	Anatonchus	**-0.562**	**0.406**	**4.685**	**4.685**
Wilsonema	Mononchus	**-0.562**	**-0.938**	**4.685**	**4.685**
Wilsonema	Mylonchulus	**-0.562**	**-0.005**	**4.685**	**4.685**
Wilsonema	Tripyla	**-0.562**	**-0.420**	**4.685**	**5.162**
Wilsonema	Dendrolaelaps	**-0.562**	**0.027**	**4.685**	**3.570**
Wilsonema	Hypoaspis	**-0.562**	**0.334**	**4.685**	**3.871**
Wilsonema	Pergamasus	**-0.562**	**1.081**	**4.685**	**3.269**
Wilsonema	Dorylaimoidea	**-0.562**	**-0.604**	**4.685**	**5.162**
Wilsonema	Eudorylaimus	**-0.562**	**-0.166**	**4.685**	**4.685**
Wilsonema	Pungentus	**-0.562**	**0.263**	**4.685**	**4.685**
Wilsonema	Thornematidae	**-0.562**	**-0.470**	**4.685**	**4.685**
Wilsonema	Eupodes	**-0.562**	**0.005**	**4.685**	**3.746**
Wilsonema	Scutacarus	**-0.562**	**-0.608**	**4.685**	**4.114**
Wilsonema	Tarsonemus	**-0.562**	**-0.701**	**4.685**	**3.570**
Enchytraeus	Dendrolaelaps	**1.209**	**0.027**	**3.276**	**3.570**
Enchytraeus	Hypoaspis	**1.209**	**0.334**	**3.276**	**3.871**
Enchytraeus	Pergamasus	**1.209**	**1.081**	**3.276**	**3.269**
Enchytraeus	Dorylaimoidea	**1.209**	**-0.604**	**3.276**	**5.162**
Enchytraeus	Eudorylaimus	**1.209**	**-0.166**	**3.276**	**4.685**
Enchytraeus	Pungentus	**1.209**	**0.263**	**3.276**	**4.685**
Enchytraeus	Thornematidae	**1.209**	**-0.470**	**3.276**	**4.685**
Enchytraeus	Eupodes	**1.209**	**0.005**	**3.276**	**3.746**
Enchytraeus	Scutacarus	**1.209**	**-0.608**	**3.276**	**4.114**
Enchytraeus	Tarsonemus	**1.209**	**-0.701**	**3.276**	**3.570**
Eubacteria	Acrobeloides	**-6.574**	**-1.171**	**13.007**	**5.463**

Eubacteria	Anaplectus	**-6.574**	**-0.519**	**13.007**	**4.685**
Eubacteria	Cephalobidae	**-6.574**	**-1.055**	**13.007**	**4.986**
Eubacteria	Eucephalobus	**-6.574**	**-0.855**	**13.007**	**5.383**
Eubacteria	Eumonhystera	**-6.574**	**-0.613**	**13.007**	**4.986**
Eubacteria	Metateratocephalus	**-6.574**	**-1.506**	**13.007**	**4.685**
Eubacteria	Panagrolaimus	**-6.574**	**-0.945**	**13.007**	**5.162**
Eubacteria	Plectus	**-6.574**	**-0.583**	**13.007**	**5.588**
Eubacteria	Prismatolaimus	**-6.574**	**-1.280**	**13.007**	**4.986**
Eubacteria	Rhabditidae	**-6.574**	**-0.692**	**13.007**	**5.530**
Eubacteria	Wilsonema	**-6.574**	**-0.562**	**13.007**	**4.685**
Eubacteria	Enchytraeus	**-6.574**	**1.209**	**13.007**	**3.276**
Eubacteria	Dauerlarvae	**-6.574**	**-0.804**	**13.007**	**4.685**
Eubacteria	Henlea	**-6.574**	**1.053**	**13.007**	**2.725**
Eubacteria	Marionina	**-6.574**	**0.759**	**13.007**	**3.640**
Dauerlarvae	Anatonchus	**-0.804**	**0.406**	**4.685**	**4.685**
Dauerlarvae	Mononchus	**-0.804**	**-0.938**	**4.685**	**4.685**
Dauerlarvae	Mylonchulus	**-0.804**	**-0.005**	**4.685**	**4.685**
Dauerlarvae	Tripyla	**-0.804**	**-0.420**	**4.685**	**5.162**
Dauerlarvae	Dorylaimoidea	**-0.804**	**-0.604**	**4.685**	**5.162**
Dauerlarvae	Eudorylaimus	**-0.804**	**-0.166**	**4.685**	**4.685**
Dauerlarvae	Pungentus	**-0.804**	**0.263**	**4.685**	**4.685**
Dauerlarvae	Thornematidae	**-0.804**	**-0.470**	**4.685**	**4.685**
Dauerlarvae	Eupodes	**-0.804**	**0.005**	**4.685**	**3.746**
Dauerlarvae	Scutacarus	**-0.804**	**-0.608**	**4.685**	**4.114**
Dauerlarvae	Tarsonemus	**-0.804**	**-0.701**	**4.685**	**3.570**
Henlea	Dendrolaelaps	**1.053**	**0.027**	**2.725**	**3.570**
Henlea	Hypoaspis	**1.053**	**0.334**	**2.725**	**3.871**
Henlea	Pergamasus	**1.053**	**1.081**	**2.725**	**3.269**
Henlea	Dorylaimoidea	**1.053**	**-0.604**	**2.725**	**5.162**
Henlea	Eudorylaimus	**1.053**	**-0.166**	**2.725**	**4.685**
Henlea	Pungentus	**1.053**	**0.263**	**2.725**	**4.685**
Henlea	Thornematidae	**1.053**	**-0.470**	**2.725**	**4.685**
Henlea	Eupodes	**1.053**	**0.005**	**2.725**	**3.746**
Henlea	Scutacarus	**1.053**	**-0.608**	**2.725**	**4.114**
Henlea	Tarsonemus	**1.053**	**-0.701**	**2.725**	**3.570**
Marionina	Dendrolaelaps	**0.759**	**0.027**	**3.640**	**3.570**
Marionina	Hypoaspis	**0.759**	**0.334**	**3.640**	**3.871**
Marionina	Pergamasus	**0.759**	**1.081**	**3.640**	**3.269**
Marionina	Dorylaimoidea	**0.759**	**-0.604**	**3.640**	**5.162**
Marionina	Eudorylaimus	**0.759**	**-0.166**	**3.640**	**4.685**
Marionina	Pungentus	**0.759**	**0.263**	**3.640**	**4.685**
Marionina	Thornematidae	**0.759**	**-0.470**	**3.640**	**4.685**
Marionina	Eupodes	**0.759**	**0.005**	**3.640**	**3.746**
Marionina	Scutacarus	**0.759**	**-0.608**	**3.640**	**4.114**
Marionina	Tarsonemus	**0.759**	**-0.701**	**3.640**	**3.570**
Hyphae and hair roots	Criconematidae	**6.314**	**-0.753**	**0.000**	**5.287**
Hyphae and hair roots	Filenchus	**6.314**	**-1.033**	**0.000**	**4.986**
Hyphae and hair roots	Helicotylenchus	**6.314**	**-0.792**	**0.000**	**5.383**
Hyphae and hair roots	Heterodera	**6.314**	**-0.883**	**0.000**	**5.463**
Hyphae and hair roots	Meloidogyne	**6.314**	**-1.287**	**0.000**	**5.162**
Hyphae and hair roots	Paratylenchus	**6.314**	**-1.244**	**0.000**	**4.986**

Hyphae and hair roots	Pratylenchus	**6.314**	**-1.226**	**0.000**	**5.162**
Hyphae and hair roots	Tylenchorhynchus	**6.314**	**-0.664**	**0.000**	**4.685**
Hyphae and hair roots	Galumna	**6.314**	**0.474**	**0.000**	**3.269**
Hyphae and hair roots	Platynothrus	**6.314**	**0.710**	**0.000**	**3.871**
Hyphae and hair roots	Tydeidae	**6.314**	**-0.608**	**0.000**	**4.172**
Hyphae and hair roots	Sminthuridae	**6.314**	**-0.608**	**0.000**	**3.871**
Hyphae and hair roots	Sminthurinus	**6.314**	**0.618**	**0.000**	**3.269**
Hyphae and hair roots	Aphelenchoides	**6.314**	**-1.496**	**0.000**	**5.162**
Hyphae and hair roots	Tylenchidae	**6.314**	**-1.360**	**0.000**	**6.530**
Hyphae and hair roots	Brachychthoniidae	**6.314**	**-0.586**	**0.000**	**3.871**
Hyphae and hair roots	Medioppia	**6.314**	**-0.235**	**0.000**	**3.570**
Hyphae and hair roots	Microppia	**6.314**	**-0.544**	**0.000**	**4.114**
Hyphae and hair roots	Minunthozetes	**6.314**	**-0.249**	**0.000**	**3.570**
Hyphae and hair roots	Oppiella	**6.314**	**-0.447**	**0.000**	**3.871**
Hyphae and hair roots	Pygmephorus	**6.314**	**-0.376**	**0.000**	**3.746**
Hyphae and hair roots	Speleorchestes	**6.314**	**-0.249**	**0.000**	**3.570**
Hyphae and hair roots	Trichoribates	**6.314**	**0.474**	**0.000**	**3.570**
Hyphae and hair roots	Tyrophagus	**6.314**	**0.005**	**0.000**	**3.746**
Hyphae and hair roots	Parisotoma	**6.314**	**0.722**	**0.000**	**3.269**
Hyphae and hair roots	Achaeta	**6.314**	**0.831**	**0.000**	**1.771**
Hyphae and hair roots	Cognettia	**6.314**	**1.215**	**0.000**	**2.849**
Hyphae and hair roots	Fridericia	**6.314**	**2.158**	**0.000**	**2.975**
Hyphae and hair roots	Dorylaimoidea	**6.314**	**-0.604**	**0.000**	**5.162**
Hyphae and hair roots	Eudorylaimus	**6.314**	**-0.166**	**0.000**	**4.685**
Hyphae and hair roots	Pungentus	**6.314**	**0.263**	**0.000**	**4.685**
Hyphae and hair roots	Thornematidae	**6.314**	**-0.470**	**0.000**	**4.685**
Hyphae and hair roots	Eupodes	**6.314**	**0.005**	**0.000**	**3.746**
Hyphae and hair roots	Scutacarus	**6.314**	**-0.608**	**0.000**	**4.114**
Hyphae and hair roots	Tarsonemus	**6.314**	**-0.701**	**0.000**	**3.570**
Anatonchus	Dendrolaelaps	**0.406**	**0.027**	**4.685**	**3.570**
Anatonchus	Hypoaspis	**0.406**	**0.334**	**4.685**	**3.871**
Anatonchus	Pergamasus	**0.406**	**1.081**	**4.685**	**3.269**
Anatonchus	Dorylaimoidea	**0.406**	**-0.604**	**4.685**	**5.162**
Anatonchus	Eudorylaimus	**0.406**	**-0.166**	**4.685**	**4.685**
Anatonchus	Pungentus	**0.406**	**0.263**	**4.685**	**4.685**
Anatonchus	Thornematidae	**0.406**	**-0.470**	**4.685**	**4.685**
Anatonchus	Eupodes	**0.406**	**0.005**	**4.685**	**3.746**
Anatonchus	Scutacarus	**0.406**	**-0.608**	**4.685**	**4.114**
Anatonchus	Tarsonemus	**0.406**	**-0.701**	**4.685**	**3.570**
Mononchus	Dendrolaelaps	**-0.938**	**0.027**	**4.685**	**3.570**
Mononchus	Hypoaspis	**-0.938**	**0.334**	**4.685**	**3.871**
Mononchus	Pergamasus	**-0.938**	**1.081**	**4.685**	**3.269**
Mononchus	Dorylaimoidea	**-0.938**	**-0.604**	**4.685**	**5.162**
Mononchus	Eudorylaimus	**-0.938**	**-0.166**	**4.685**	**4.685**
Mononchus	Pungentus	**-0.938**	**0.263**	**4.685**	**4.685**
Mononchus	Thornematidae	**-0.938**	**-0.470**	**4.685**	**4.685**
Mononchus	Eupodes	**-0.938**	**0.005**	**4.685**	**3.746**
Mononchus	Scutacarus	**-0.938**	**-0.608**	**4.685**	**4.114**
Mononchus	Tarsonemus	**-0.938**	**-0.701**	**4.685**	**3.570**
Mylonchulus	Dendrolaelaps	**-0.005**	**0.027**	**4.685**	**3.570**
Mylonchulus	Hypoaspis	**-0.005**	**0.334**	**4.685**	**3.871**

Mylonchulus	Pergamasus	**-0.005**	**1.081**	**4.685**	**3.269**
Mylonchulus	Dorylaimoidea	**-0.005**	**-0.604**	**4.685**	**5.162**
Mylonchulus	Eudorylaimus	**-0.005**	**-0.166**	**4.685**	**4.685**
Mylonchulus	Pungentus	**-0.005**	**0.263**	**4.685**	**4.685**
Mylonchulus	Thornematidae	**-0.005**	**-0.470**	**4.685**	**4.685**
Mylonchulus	Eupodes	**-0.005**	**0.005**	**4.685**	**3.746**
Mylonchulus	Scutacarus	**-0.005**	**-0.608**	**4.685**	**4.114**
Mylonchulus	Tarsonemus	**-0.005**	**-0.701**	**4.685**	**3.570**
Tripyla	Dendrolaelaps	**-0.420**	**0.027**	**5.162**	**3.570**
Tripyla	Hypoaspis	**-0.420**	**0.334**	**5.162**	**3.871**
Tripyla	Pergamasus	**-0.420**	**1.081**	**5.162**	**3.269**
Tripyla	Dorylaimoidea	**-0.420**	**-0.604**	**5.162**	**5.162**
Tripyla	Eudorylaimus	**-0.420**	**-0.166**	**5.162**	**4.685**
Tripyla	Pungentus	**-0.420**	**0.263**	**5.162**	**4.685**
Tripyla	Thornematidae	**-0.420**	**-0.470**	**5.162**	**4.685**
Tripyla	Eupodes	**-0.420**	**0.005**	**5.162**	**3.746**
Tripyla	Scutacarus	**-0.420**	**-0.608**	**5.162**	**4.114**
Tripyla	Tarsonemus	**-0.420**	**-0.701**	**5.162**	**3.570**
Bdella	Bdella	**0.816**	**0.816**	**3.269**	**3.269**
Bdella	Dendrolaelaps	**0.816**	**0.027**	**3.269**	**3.570**
Bdella	Hypoaspis	**0.816**	**0.334**	**3.269**	**3.871**
Bdella	Pergamasus	**0.816**	**1.081**	**3.269**	**3.269**
Bdella	Eupodes	**0.816**	**0.005**	**3.269**	**3.746**
Bdella	Scutacarus	**0.816**	**-0.608**	**3.269**	**4.114**
Bdella	Tarsonemus	**0.816**	**-0.701**	**3.269**	**3.570**
Bdella	Pyemotes	**0.816**	**-0.608**	**3.269**	**3.269**
Dendrolaelaps	Bdella	**0.027**	**0.816**	**3.570**	**3.269**
Dendrolaelaps	Dorylaimoidea	**0.027**	**-0.604**	**3.570**	**5.162**
Dendrolaelaps	Eudorylaimus	**0.027**	**-0.166**	**3.570**	**4.685**
Dendrolaelaps	Pungentus	**0.027**	**0.263**	**3.570**	**4.685**
Dendrolaelaps	Thornematidae	**0.027**	**-0.470**	**3.570**	**4.685**
Dendrolaelaps	Eupodes	**0.027**	**0.005**	**3.570**	**3.746**
Dendrolaelaps	Scutacarus	**0.027**	**-0.608**	**3.570**	**4.114**
Dendrolaelaps	Tarsonemus	**0.027**	**-0.701**	**3.570**	**3.570**
Dendrolaelaps	Pyemotes	**0.027**	**-0.608**	**3.570**	**3.269**
Hypoaspis	Bdella	**0.334**	**0.816**	**3.871**	**3.269**
Hypoaspis	Dorylaimoidea	**0.334**	**-0.604**	**3.871**	**5.162**
Hypoaspis	Eudorylaimus	**0.334**	**-0.166**	**3.871**	**4.685**
Hypoaspis	Pungentus	**0.334**	**0.263**	**3.871**	**4.685**
Hypoaspis	Thornematidae	**0.334**	**-0.470**	**3.871**	**4.685**
Hypoaspis	Eupodes	**0.334**	**0.005**	**3.871**	**3.746**
Hypoaspis	Scutacarus	**0.334**	**-0.608**	**3.871**	**4.114**
Hypoaspis	Tarsonemus	**0.334**	**-0.701**	**3.871**	**3.570**
Hypoaspis	Pyemotes	**0.334**	**-0.608**	**3.871**	**3.269**
Pergamasus	Bdella	**1.081**	**0.816**	**3.269**	**3.269**
Pergamasus	Dorylaimoidea	**1.081**	**-0.604**	**3.269**	**5.162**
Pergamasus	Eudorylaimus	**1.081**	**-0.166**	**3.269**	**4.685**
Pergamasus	Pungentus	**1.081**	**0.263**	**3.269**	**4.685**
Pergamasus	Thornematidae	**1.081**	**-0.470**	**3.269**	**4.685**
Pergamasus	Eupodes	**1.081**	**0.005**	**3.269**	**3.746**
Pergamasus	Scutacarus	**1.081**	**-0.608**	**3.269**	**4.114**

Pergamasus	Tarsonemus	**1.081**	**-0.701**	**3.269**	**3.570**
Pergamasus	Pyemotes	**1.081**	**-0.608**	**3.269**	**3.269**
Dorylaimoidea	Anatonchus	**-0.604**	**0.406**	**5.162**	**4.685**
Dorylaimoidea	Mononchus	**-0.604**	**-0.938**	**5.162**	**4.685**
Dorylaimoidea	Mylonchulus	**-0.604**	**-0.005**	**5.162**	**4.685**
Dorylaimoidea	Tripyla	**-0.604**	**-0.420**	**5.162**	**5.162**
Dorylaimoidea	Dendrolaelaps	**-0.604**	**0.027**	**5.162**	**3.570**
Dorylaimoidea	Hypoaspis	**-0.604**	**0.334**	**5.162**	**3.871**
Dorylaimoidea	Pergamasus	**-0.604**	**1.081**	**5.162**	**3.269**
Dorylaimoidea	Dorylaimoidea	**-0.604**	**-0.604**	**5.162**	**5.162**
Dorylaimoidea	Eudorylaimus	**-0.604**	**-0.166**	**5.162**	**4.685**
Dorylaimoidea	Pungentus	**-0.604**	**0.263**	**5.162**	**4.685**
Dorylaimoidea	Thornematidae	**-0.604**	**-0.470**	**5.162**	**4.685**
Dorylaimoidea	Eupodes	**-0.604**	**0.005**	**5.162**	**3.746**
Dorylaimoidea	Scutacarus	**-0.604**	**-0.608**	**5.162**	**4.114**
Dorylaimoidea	Tarsonemus	**-0.604**	**-0.701**	**5.162**	**3.570**
Eudorylaimus	Anatonchus	**-0.166**	**0.406**	**4.685**	**4.685**
Eudorylaimus	Mononchus	**-0.166**	**-0.938**	**4.685**	**4.685**
Eudorylaimus	Mylonchulus	**-0.166**	**-0.005**	**4.685**	**4.685**
Eudorylaimus	Tripyla	**-0.166**	**-0.420**	**4.685**	**5.162**
Eudorylaimus	Dendrolaelaps	**-0.166**	**0.027**	**4.685**	**3.570**
Eudorylaimus	Hypoaspis	**-0.166**	**0.334**	**4.685**	**3.871**
Eudorylaimus	Pergamasus	**-0.166**	**1.081**	**4.685**	**3.269**
Eudorylaimus	Dorylaimoidea	**-0.166**	**-0.604**	**4.685**	**5.162**
Eudorylaimus	Eudorylaimus	**-0.166**	**-0.166**	**4.685**	**4.685**
Eudorylaimus	Pungentus	**-0.166**	**0.263**	**4.685**	**4.685**
Eudorylaimus	Thornematidae	**-0.166**	**-0.470**	**4.685**	**4.685**
Eudorylaimus	Eupodes	**-0.166**	**0.005**	**4.685**	**3.746**
Eudorylaimus	Scutacarus	**-0.166**	**-0.608**	**4.685**	**4.114**
Eudorylaimus	Tarsonemus	**-0.166**	**-0.701**	**4.685**	**3.570**
Pungentus	Anatonchus	**0.263**	**0.406**	**4.685**	**4.685**
Pungentus	Mononchus	**0.263**	**-0.938**	**4.685**	**4.685**
Pungentus	Mylonchulus	**0.263**	**-0.005**	**4.685**	**4.685**
Pungentus	Tripyla	**0.263**	**-0.420**	**4.685**	**5.162**
Pungentus	Dendrolaelaps	**0.263**	**0.027**	**4.685**	**3.570**
Pungentus	Hypoaspis	**0.263**	**0.334**	**4.685**	**3.871**
Pungentus	Pergamasus	**0.263**	**1.081**	**4.685**	**3.269**
Pungentus	Dorylaimoidea	**0.263**	**-0.604**	**4.685**	**5.162**
Pungentus	Eudorylaimus	**0.263**	**-0.166**	**4.685**	**4.685**
Pungentus	Pungentus	**0.263**	**0.263**	**4.685**	**4.685**
Pungentus	Thornematidae	**0.263**	**-0.470**	**4.685**	**4.685**
Pungentus	Eupodes	**0.263**	**0.005**	**4.685**	**3.746**
Pungentus	Scutacarus	**0.263**	**-0.608**	**4.685**	**4.114**
Pungentus	Tarsonemus	**0.263**	**-0.701**	**4.685**	**3.570**
Thornematidae	Anatonchus	**-0.470**	**0.406**	**4.685**	**4.685**
Thornematidae	Mononchus	**-0.470**	**-0.938**	**4.685**	**4.685**
Thornematidae	Mylonchulus	**-0.470**	**-0.005**	**4.685**	**4.685**
Thornematidae	Tripyla	**-0.470**	**-0.420**	**4.685**	**5.162**
Thornematidae	Dendrolaelaps	**-0.470**	**0.027**	**4.685**	**3.570**
Thornematidae	Hypoaspis	**-0.470**	**0.334**	**4.685**	**3.871**
Thornematidae	Pergamasus	**-0.470**	**1.081**	**4.685**	**3.269**

Thornematidae	Dorylaimoidea	**-0.470**	**-0.604**	**4.685**	**5.162**
Thornematidae	Eudorylaimus	**-0.470**	**-0.166**	**4.685**	**4.685**
Thornematidae	Pungentus	**-0.470**	**0.263**	**4.685**	**4.685**
Thornematidae	Thornematidae	**-0.470**	**-0.470**	**4.685**	**4.685**
Thornematidae	Eupodes	**-0.470**	**0.005**	**4.685**	**3.746**
Thornematidae	Scutacarus	**-0.470**	**-0.608**	**4.685**	**4.114**
Thornematidae	Tarsonemus	**-0.470**	**-0.701**	**4.685**	**3.570**
Eupodes	Bdella	**0.005**	**0.816**	**3.746**	**3.269**
Eupodes	Dendrolaelaps	**0.005**	**0.027**	**3.746**	**3.570**
Eupodes	Hypoaspis	**0.005**	**0.334**	**3.746**	**3.871**
Eupodes	Pergamasus	**0.005**	**1.081**	**3.746**	**3.269**
Eupodes	Dorylaimoidea	**0.005**	**-0.604**	**3.746**	**5.162**
Eupodes	Eudorylaimus	**0.005**	**-0.166**	**3.746**	**4.685**
Eupodes	Pungentus	**0.005**	**0.263**	**3.746**	**4.685**
Eupodes	Thornematidae	**0.005**	**-0.470**	**3.746**	**4.685**
Eupodes	Eupodes	**0.005**	**0.005**	**3.746**	**3.746**
Eupodes	Scutacarus	**0.005**	**-0.608**	**3.746**	**4.114**
Eupodes	Tarsonemus	**0.005**	**-0.701**	**3.746**	**3.570**
Scutacarus	Bdella	**-0.608**	**0.816**	**4.114**	**3.269**
Scutacarus	Dendrolaelaps	**-0.608**	**0.027**	**4.114**	**3.570**
Scutacarus	Hypoaspis	**-0.608**	**0.334**	**4.114**	**3.871**
Scutacarus	Pergamasus	**-0.608**	**1.081**	**4.114**	**3.269**
Scutacarus	Dorylaimoidea	**-0.608**	**-0.604**	**4.114**	**5.162**
Scutacarus	Eudorylaimus	**-0.608**	**-0.166**	**4.114**	**4.685**
Scutacarus	Pungentus	**-0.608**	**0.263**	**4.114**	**4.685**
Scutacarus	Thornematidae	**-0.608**	**-0.470**	**4.114**	**4.685**
Scutacarus	Eupodes	**-0.608**	**0.005**	**4.114**	**3.746**
Scutacarus	Scutacarus	**-0.608**	**-0.608**	**4.114**	**4.114**
Scutacarus	Tarsonemus	**-0.608**	**-0.701**	**4.114**	**3.570**
Tarsonemus	Bdella	**-0.701**	**0.816**	**3.570**	**3.269**
Tarsonemus	Dendrolaelaps	**-0.701**	**0.027**	**3.570**	**3.570**
Tarsonemus	Hypoaspis	**-0.701**	**0.334**	**3.570**	**3.871**
Tarsonemus	Pergamasus	**-0.701**	**1.081**	**3.570**	**3.269**
Tarsonemus	Dorylaimoidea	**-0.701**	**-0.604**	**3.570**	**5.162**
Tarsonemus	Eudorylaimus	**-0.701**	**-0.166**	**3.570**	**4.685**
Tarsonemus	Pungentus	**-0.701**	**0.263**	**3.570**	**4.685**
Tarsonemus	Thornematidae	**-0.701**	**-0.470**	**3.570**	**4.685**
Tarsonemus	Eupodes	**-0.701**	**0.005**	**3.570**	**3.746**
Tarsonemus	Scutacarus	**-0.701**	**-0.608**	**3.570**	**4.114**
Tarsonemus	Tarsonemus	**-0.701**	**-0.701**	**3.570**	**3.570**
Pyemotes	Bdella	**-0.608**	**0.816**	**3.269**	**3.269**
Pyemotes	Eupodes	**-0.608**	**0.005**	**3.269**	**3.746**
Pyemotes	Scutacarus	**-0.608**	**-0.608**	**3.269**	**4.114**
Pyemotes	Tarsonemus	**-0.608**	**-0.701**	**3.269**	**3.570**

Resource	Consumer	Mres	Mconsumer	Nres	Nconsumer
Coslenchus	Mylonchulus	**-0.821**	**-0.005**	**4.563**	**4.563**
Coslenchus	Tripyla	**-0.821**	**-0.420**	**4.563**	**4.563**
Coslenchus	Alliphis	**-0.821**	**0.053**	**4.563**	**3.215**
Coslenchus	Arctoseius	**-0.821**	**-0.152**	**4.563**	**3.516**
Coslenchus	Cheiroseius	**-0.821**	**0.356**	**4.563**	**3.215**
Coslenchus	Lysigamasus	**-0.821**	**0.407**	**4.563**	**3.692**
Coslenchus	Uropoda	**-0.821**	**0.481**	**4.563**	**3.215**
Coslenchus	Aporcelaimellus	**-0.821**	**0.548**	**4.563**	**5.341**
Coslenchus	Dorylaimoidea	**-0.821**	**-0.604**	**4.563**	**5.341**
Coslenchus	Eudorylaimus	**-0.821**	**-0.166**	**4.563**	**4.864**
Coslenchus	Thornematidae	**-0.821**	**-0.470**	**4.563**	**4.864**
Coslenchus	Eupodes	**-0.821**	**0.005**	**4.563**	**4.118**
Coslenchus	Nenteria	**-0.821**	**0.254**	**4.563**	**3.516**
Coslenchus	Scutacarus	**-0.821**	**-0.608**	**4.563**	**4.215**
Coslenchus	Tarsonemus	**-0.821**	**-0.701**	**4.563**	**3.516**
Dolichodoridae	Mylonchulus	**-0.885**	**-0.005**	**5.408**	**4.563**
Dolichodoridae	Tripyla	**-0.885**	**-0.420**	**5.408**	**4.563**
Dolichodoridae	Alliphis	**-0.885**	**0.053**	**5.408**	**3.215**
Dolichodoridae	Arctoseius	**-0.885**	**-0.152**	**5.408**	**3.516**
Dolichodoridae	Cheiroseius	**-0.885**	**0.356**	**5.408**	**3.215**
Dolichodoridae	Lysigamasus	**-0.885**	**0.407**	**5.408**	**3.692**
Dolichodoridae	Uropoda	**-0.885**	**0.481**	**5.408**	**3.215**
Dolichodoridae	Aporcelaimellus	**-0.885**	**0.548**	**5.408**	**5.341**
Dolichodoridae	Dorylaimoidea	**-0.885**	**-0.604**	**5.408**	**5.341**
Dolichodoridae	Eudorylaimus	**-0.885**	**-0.166**	**5.408**	**4.864**
Dolichodoridae	Thornematidae	**-0.885**	**-0.470**	**5.408**	**4.864**
Dolichodoridae	Eupodes	**-0.885**	**0.005**	**5.408**	**4.118**
Dolichodoridae	Nenteria	**-0.885**	**0.254**	**5.408**	**3.516**
Dolichodoridae	Scutacarus	**-0.885**	**-0.608**	**5.408**	**4.215**
Dolichodoridae	Tarsonemus	**-0.885**	**-0.701**	**5.408**	**3.516**
Helicotylenchus	Mylonchulus	**-0.792**	**-0.005**	**5.341**	**4.563**
Helicotylenchus	Tripyla	**-0.792**	**-0.420**	**5.341**	**4.563**
Helicotylenchus	Alliphis	**-0.792**	**0.053**	**5.341**	**3.215**
Helicotylenchus	Arctoseius	**-0.792**	**-0.152**	**5.341**	**3.516**
Helicotylenchus	Cheiroseius	**-0.792**	**0.356**	**5.341**	**3.215**
Helicotylenchus	Lysigamasus	**-0.792**	**0.407**	**5.341**	**3.692**
Helicotylenchus	Uropoda	**-0.792**	**0.481**	**5.341**	**3.215**
Helicotylenchus	Aporcelaimellus	**-0.792**	**0.548**	**5.341**	**5.341**
Helicotylenchus	Dorylaimoidea	**-0.792**	**-0.604**	**5.341**	**5.341**
Helicotylenchus	Eudorylaimus	**-0.792**	**-0.166**	**5.341**	**4.864**
Helicotylenchus	Thornematidae	**-0.792**	**-0.470**	**5.341**	**4.864**
Helicotylenchus	Eupodes	**-0.792**	**0.005**	**5.341**	**4.118**
Helicotylenchus	Nenteria	**-0.792**	**0.254**	**5.341**	**3.516**
Helicotylenchus	Scutacarus	**-0.792**	**-0.608**	**5.341**	**4.215**
Helicotylenchus	Tarsonemus	**-0.792**	**-0.701**	**5.341**	**3.516**
Paratylenchus	Mylonchulus	**-1.244**	**-0.005**	**4.864**	**4.563**
Paratylenchus	Tripyla	**-1.244**	**-0.420**	**4.864**	**4.563**
Paratylenchus	Alliphis	**-1.244**	**0.053**	**4.864**	**3.215**
Paratylenchus	Arctoseius	**-1.244**	**-0.152**	**4.864**	**3.516**
Paratylenchus	Cheiroseius	**-1.244**	**0.356**	**4.864**	**3.215**

Paratylenchus	Lysigamasus	**-1.244**	**0.407**	**4.864**	**3.692**
Paratylenchus	Uropoda	**-1.244**	**0.481**	**4.864**	**3.215**
Paratylenchus	Aporcelaimellus	**-1.244**	**0.548**	**4.864**	**5.341**
Paratylenchus	Dorylaimoidea	**-1.244**	**-0.604**	**4.864**	**5.341**
Paratylenchus	Eudorylaimus	**-1.244**	**-0.166**	**4.864**	**4.864**
Paratylenchus	Thornematidae	**-1.244**	**-0.470**	**4.864**	**4.864**
Paratylenchus	Eupodes	**-1.244**	**0.005**	**4.864**	**4.118**
Paratylenchus	Nenteria	**-1.244**	**0.254**	**4.864**	**3.516**
Paratylenchus	Scutacarus	**-1.244**	**-0.608**	**4.864**	**4.215**
Paratylenchus	Tarsonemus	**-1.244**	**-0.701**	**4.864**	**3.516**
Pratylenchus	Mylonchulus	**-1.226**	**-0.005**	**5.040**	**4.563**
Pratylenchus	Tripyla	**-1.226**	**-0.420**	**5.040**	**4.563**
Pratylenchus	Alliphis	**-1.226**	**0.053**	**5.040**	**3.215**
Pratylenchus	Arctoseius	**-1.226**	**-0.152**	**5.040**	**3.516**
Pratylenchus	Cheiroseius	**-1.226**	**0.356**	**5.040**	**3.215**
Pratylenchus	Lysigamasus	**-1.226**	**0.407**	**5.040**	**3.692**
Pratylenchus	Uropoda	**-1.226**	**0.481**	**5.040**	**3.215**
Pratylenchus	Aporcelaimellus	**-1.226**	**0.548**	**5.040**	**5.341**
Pratylenchus	Dorylaimoidea	**-1.226**	**-0.604**	**5.040**	**5.341**
Pratylenchus	Eudorylaimus	**-1.226**	**-0.166**	**5.040**	**4.864**
Pratylenchus	Thornematidae	**-1.226**	**-0.470**	**5.040**	**4.864**
Pratylenchus	Eupodes	**-1.226**	**0.005**	**5.040**	**4.118**
Pratylenchus	Nenteria	**-1.226**	**0.254**	**5.040**	**3.516**
Pratylenchus	Scutacarus	**-1.226**	**-0.608**	**5.040**	**4.215**
Pratylenchus	Tarsonemus	**-1.226**	**-0.701**	**5.040**	**3.516**
Tylenchorhynchus	Mylonchulus	**-0.664**	**-0.005**	**5.341**	**4.563**
Tylenchorhynchus	Tripyla	**-0.664**	**-0.420**	**5.341**	**4.563**
Tylenchorhynchus	Alliphis	**-0.664**	**0.053**	**5.341**	**3.215**
Tylenchorhynchus	Arctoseius	**-0.664**	**-0.152**	**5.341**	**3.516**
Tylenchorhynchus	Cheiroseius	**-0.664**	**0.356**	**5.341**	**3.215**
Tylenchorhynchus	Lysigamasus	**-0.664**	**0.407**	**5.341**	**3.692**
Tylenchorhynchus	Uropoda	**-0.664**	**0.481**	**5.341**	**3.215**
Tylenchorhynchus	Aporcelaimellus	**-0.664**	**0.548**	**5.341**	**5.341**
Tylenchorhynchus	Dorylaimoidea	**-0.664**	**-0.604**	**5.341**	**5.341**
Tylenchorhynchus	Eudorylaimus	**-0.664**	**-0.166**	**5.341**	**4.864**
Tylenchorhynchus	Thornematidae	**-0.664**	**-0.470**	**5.341**	**4.864**
Tylenchorhynchus	Eupodes	**-0.664**	**0.005**	**5.341**	**4.118**
Tylenchorhynchus	Nenteria	**-0.664**	**0.254**	**5.341**	**3.516**
Tylenchorhynchus	Scutacarus	**-0.664**	**-0.608**	**5.341**	**4.215**
Tylenchorhynchus	Tarsonemus	**-0.664**	**-0.701**	**5.341**	**3.516**
Pachygnatidae	Arctoseius	**-0.113**	**-0.152**	**3.516**	**3.516**
Pachygnatidae	Cheiroseius	**-0.113**	**0.356**	**3.516**	**3.215**
Pachygnatidae	Lysigamasus	**-0.113**	**0.407**	**3.516**	**3.692**
Pachygnatidae	Uropoda	**-0.113**	**0.481**	**3.516**	**3.215**
Pachygnatidae	Aporcelaimellus	**-0.113**	**0.548**	**3.516**	**5.341**
Pachygnatidae	Dorylaimoidea	**-0.113**	**-0.604**	**3.516**	**5.341**
Pachygnatidae	Eudorylaimus	**-0.113**	**-0.166**	**3.516**	**4.864**
Pachygnatidae	Thornematidae	**-0.113**	**-0.470**	**3.516**	**4.864**
Pachygnatidae	Eupodes	**-0.113**	**0.005**	**3.516**	**4.118**
Pachygnatidae	Nenteria	**-0.113**	**0.254**	**3.516**	**3.516**
Pachygnatidae	Scutacarus	**-0.113**	**-0.608**	**3.516**	**4.215**

Pachygnatidae	Tarsonemus	**-0.113**	**-0.701**	**3.516**	**3.516**
Platynothrus	Arctoseius	**0.710**	**-0.152**	**3.993**	**3.516**
Platynothrus	Cheiroseius	**0.710**	**0.356**	**3.993**	**3.215**
Platynothrus	Lysigamasus	**0.710**	**0.407**	**3.993**	**3.692**
Platynothrus	Uropoda	**0.710**	**0.481**	**3.993**	**3.215**
Platynothrus	Aporcelaimellus	**0.710**	**0.548**	**3.993**	**5.341**
Platynothrus	Dorylaimoidea	**0.710**	**-0.604**	**3.993**	**5.341**
Platynothrus	Eudorylaimus	**0.710**	**-0.166**	**3.993**	**4.864**
Platynothrus	Thornematidae	**0.710**	**-0.470**	**3.993**	**4.864**
Platynothrus	Eupodes	**0.710**	**0.005**	**3.993**	**4.118**
Platynothrus	Nenteria	**0.710**	**0.254**	**3.993**	**3.516**
Platynothrus	Scutacarus	**0.710**	**-0.608**	**3.993**	**4.215**
Platynothrus	Tarsonemus	**0.710**	**-0.701**	**3.993**	**3.516**
Tydeidae	Arctoseius	**-0.608**	**-0.152**	**3.692**	**3.516**
Tydeidae	Cheiroseius	**-0.608**	**0.356**	**3.692**	**3.215**
Tydeidae	Lysigamasus	**-0.608**	**0.407**	**3.692**	**3.692**
Tydeidae	Uropoda	**-0.608**	**0.481**	**3.692**	**3.215**
Tydeidae	Aporcelaimellus	**-0.608**	**0.548**	**3.692**	**5.341**
Tydeidae	Dorylaimoidea	**-0.608**	**-0.604**	**3.692**	**5.341**
Tydeidae	Eudorylaimus	**-0.608**	**-0.166**	**3.692**	**4.864**
Tydeidae	Thornematidae	**-0.608**	**-0.470**	**3.692**	**4.864**
Tydeidae	Eupodes	**-0.608**	**0.005**	**3.692**	**4.118**
Tydeidae	Nenteria	**-0.608**	**0.254**	**3.692**	**3.516**
Tydeidae	Scutacarus	**-0.608**	**-0.608**	**3.692**	**4.215**
Tydeidae	Tarsonemus	**-0.608**	**-0.701**	**3.692**	**3.516**
Sminthuridae	Arctoseius	**-0.608**	**-0.152**	**3.516**	**3.516**
Sminthuridae	Cheiroseius	**-0.608**	**0.356**	**3.516**	**3.215**
Sminthuridae	Lysigamasus	**-0.608**	**0.407**	**3.516**	**3.692**
Sminthuridae	Uropoda	**-0.608**	**0.481**	**3.516**	**3.215**
Sminthuridae	Aporcelaimellus	**-0.608**	**0.548**	**3.516**	**5.341**
Sminthuridae	Dorylaimoidea	**-0.608**	**-0.604**	**3.516**	**5.341**
Sminthuridae	Eudorylaimus	**-0.608**	**-0.166**	**3.516**	**4.864**
Sminthuridae	Thornematidae	**-0.608**	**-0.470**	**3.516**	**4.864**
Sminthuridae	Eupodes	**-0.608**	**0.005**	**3.516**	**4.118**
Sminthuridae	Nenteria	**-0.608**	**0.254**	**3.516**	**3.516**
Sminthuridae	Scutacarus	**-0.608**	**-0.608**	**3.516**	**4.215**
Sminthuridae	Tarsonemus	**-0.608**	**-0.701**	**3.516**	**3.516**
Sminthurinus	Arctoseius	**0.618**	**-0.152**	**3.692**	**3.516**
Sminthurinus	Cheiroseius	**0.618**	**0.356**	**3.692**	**3.215**
Sminthurinus	Lysigamasus	**0.618**	**0.407**	**3.692**	**3.692**
Sminthurinus	Uropoda	**0.618**	**0.481**	**3.692**	**3.215**
Sminthurinus	Aporcelaimellus	**0.618**	**0.548**	**3.692**	**5.341**
Sminthurinus	Dorylaimoidea	**0.618**	**-0.604**	**3.692**	**5.341**
Sminthurinus	Eudorylaimus	**0.618**	**-0.166**	**3.692**	**4.864**
Sminthurinus	Thornematidae	**0.618**	**-0.470**	**3.692**	**4.864**
Sminthurinus	Eupodes	**0.618**	**0.005**	**3.692**	**4.118**
Sminthurinus	Nenteria	**0.618**	**0.254**	**3.692**	**3.516**
Sminthurinus	Scutacarus	**0.618**	**-0.608**	**3.692**	**4.215**
Sminthurinus	Tarsonemus	**0.618**	**-0.701**	**3.692**	**3.516**
Sphaeridia	Arctoseius	**0.202**	**-0.152**	**3.516**	**3.516**
Sphaeridia	Cheiroseius	**0.202**	**0.356**	**3.516**	**3.215**

Sphaeridia	Lysigamasus	**0.202**	**0.407**	**3.516**	**3.692**
Sphaeridia	Uropoda	**0.202**	**0.481**	**3.516**	**3.215**
Sphaeridia	Aporcelaimellus	**0.202**	**0.548**	**3.516**	**5.341**
Sphaeridia	Dorylaimoidea	**0.202**	**-0.604**	**3.516**	**5.341**
Sphaeridia	Eudorylaimus	**0.202**	**-0.166**	**3.516**	**4.864**
Sphaeridia	Thornematidae	**0.202**	**-0.470**	**3.516**	**4.864**
Sphaeridia	Eupodes	**0.202**	**0.005**	**3.516**	**4.118**
Sphaeridia	Nenteria	**0.202**	**0.254**	**3.516**	**3.516**
Sphaeridia	Scutacarus	**0.202**	**-0.608**	**3.516**	**4.215**
Sphaeridia	Tarsonemus	**0.202**	**-0.701**	**3.516**	**3.516**
Aphelenchoides	Mylonchulus	**-1.496**	**-0.005**	**5.341**	**4.563**
Aphelenchoides	Tripyla	**-1.496**	**-0.420**	**5.341**	**4.563**
Aphelenchoides	Alliphis	**-1.496**	**0.053**	**5.341**	**3.215**
Aphelenchoides	Arctoseius	**-1.496**	**-0.152**	**5.341**	**3.516**
Aphelenchoides	Cheiroseius	**-1.496**	**0.356**	**5.341**	**3.215**
Aphelenchoides	Lysigamasus	**-1.496**	**0.407**	**5.341**	**3.692**
Aphelenchoides	Uropoda	**-1.496**	**0.481**	**5.341**	**3.215**
Aphelenchoides	Aporcelaimellus	**-1.496**	**0.548**	**5.341**	**5.341**
Aphelenchoides	Dorylaimoidea	**-1.496**	**-0.604**	**5.341**	**5.341**
Aphelenchoides	Eudorylaimus	**-1.496**	**-0.166**	**5.341**	**4.864**
Aphelenchoides	Thornematidae	**-1.496**	**-0.470**	**5.341**	**4.864**
Aphelenchoides	Eupodes	**-1.496**	**0.005**	**5.341**	**4.118**
Aphelenchoides	Nenteria	**-1.496**	**0.254**	**5.341**	**3.516**
Aphelenchoides	Scutacarus	**-1.496**	**-0.608**	**5.341**	**4.215**
Aphelenchoides	Tarsonemus	**-1.496**	**-0.701**	**5.341**	**3.516**
Tylenchidae	Mylonchulus	**-1.360**	**-0.005**	**5.864**	**4.563**
Tylenchidae	Tripyla	**-1.360**	**-0.420**	**5.864**	**4.563**
Tylenchidae	Alliphis	**-1.360**	**0.053**	**5.864**	**3.215**
Tylenchidae	Arctoseius	**-1.360**	**-0.152**	**5.864**	**3.516**
Tylenchidae	Cheiroseius	**-1.360**	**0.356**	**5.864**	**3.215**
Tylenchidae	Lysigamasus	**-1.360**	**0.407**	**5.864**	**3.692**
Tylenchidae	Uropoda	**-1.360**	**0.481**	**5.864**	**3.215**
Tylenchidae	Aporcelaimellus	**-1.360**	**0.548**	**5.864**	**5.341**
Tylenchidae	Dorylaimoidea	**-1.360**	**-0.604**	**5.864**	**5.341**
Tylenchidae	Eudorylaimus	**-1.360**	**-0.166**	**5.864**	**4.864**
Tylenchidae	Thornematidae	**-1.360**	**-0.470**	**5.864**	**4.864**
Tylenchidae	Eupodes	**-1.360**	**0.005**	**5.864**	**4.118**
Tylenchidae	Nenteria	**-1.360**	**0.254**	**5.864**	**3.516**
Tylenchidae	Scutacarus	**-1.360**	**-0.608**	**5.864**	**4.215**
Tylenchidae	Tarsonemus	**-1.360**	**-0.701**	**5.864**	**3.516**
Microtydeus	Arctoseius	**-0.863**	**-0.152**	**3.817**	**3.516**
Microtydeus	Cheiroseius	**-0.863**	**0.356**	**3.817**	**3.215**
Microtydeus	Lysigamasus	**-0.863**	**0.407**	**3.817**	**3.692**
Microtydeus	Uropoda	**-0.863**	**0.481**	**3.817**	**3.215**
Microtydeus	Aporcelaimellus	**-0.863**	**0.548**	**3.817**	**5.341**
Microtydeus	Dorylaimoidea	**-0.863**	**-0.604**	**3.817**	**5.341**
Microtydeus	Eudorylaimus	**-0.863**	**-0.166**	**3.817**	**4.864**
Microtydeus	Thornematidae	**-0.863**	**-0.470**	**3.817**	**4.864**
Microtydeus	Eupodes	**-0.863**	**0.005**	**3.817**	**4.118**
Microtydeus	Nenteria	**-0.863**	**0.254**	**3.817**	**3.516**
Microtydeus	Scutacarus	**-0.863**	**-0.608**	**3.817**	**4.215**

Microtydeus	Tarsonemus	**-0.863**	**-0.701**	**3.817**	**3.516**
Lepidocyrtus	Arctoseius	**1.231**	**-0.152**	**4.294**	**3.516**
Lepidocyrtus	Cheiroseius	**1.231**	**0.356**	**4.294**	**3.215**
Lepidocyrtus	Lysigamasus	**1.231**	**0.407**	**4.294**	**3.692**
Lepidocyrtus	Uropoda	**1.231**	**0.481**	**4.294**	**3.215**
Lepidocyrtus	Aporcelaimellus	**1.231**	**0.548**	**4.294**	**5.341**
Lepidocyrtus	Dorylaimoidea	**1.231**	**-0.604**	**4.294**	**5.341**
Lepidocyrtus	Eudorylaimus	**1.231**	**-0.166**	**4.294**	**4.864**
Lepidocyrtus	Thornematidae	**1.231**	**-0.470**	**4.294**	**4.864**
Lepidocyrtus	Eupodes	**1.231**	**0.005**	**4.294**	**4.118**
Lepidocyrtus	Nenteria	**1.231**	**0.254**	**4.294**	**3.516**
Lepidocyrtus	Scutacarus	**1.231**	**-0.608**	**4.294**	**4.215**
Lepidocyrtus	Tarsonemus	**1.231**	**-0.701**	**4.294**	**3.516**
Parisotoma	Arctoseius	**0.722**	**-0.152**	**3.692**	**3.516**
Parisotoma	Cheiroseius	**0.722**	**0.356**	**3.692**	**3.215**
Parisotoma	Lysigamasus	**0.722**	**0.407**	**3.692**	**3.692**
Parisotoma	Uropoda	**0.722**	**0.481**	**3.692**	**3.215**
Parisotoma	Aporcelaimellus	**0.722**	**0.548**	**3.692**	**5.341**
Parisotoma	Dorylaimoidea	**0.722**	**-0.604**	**3.692**	**5.341**
Parisotoma	Eudorylaimus	**0.722**	**-0.166**	**3.692**	**4.864**
Parisotoma	Thornematidae	**0.722**	**-0.470**	**3.692**	**4.864**
Parisotoma	Eupodes	**0.722**	**0.005**	**3.692**	**4.118**
Parisotoma	Nenteria	**0.722**	**0.254**	**3.692**	**3.516**
Parisotoma	Scutacarus	**0.722**	**-0.608**	**3.692**	**4.215**
Parisotoma	Tarsonemus	**0.722**	**-0.701**	**3.692**	**3.516**
Pseudachorutes	Arctoseius	**0.977**	**-0.152**	**3.692**	**3.516**
Pseudachorutes	Cheiroseius	**0.977**	**0.356**	**3.692**	**3.215**
Pseudachorutes	Lysigamasus	**0.977**	**0.407**	**3.692**	**3.692**
Pseudachorutes	Uropoda	**0.977**	**0.481**	**3.692**	**3.215**
Pseudachorutes	Aporcelaimellus	**0.977**	**0.548**	**3.692**	**5.341**
Pseudachorutes	Dorylaimoidea	**0.977**	**-0.604**	**3.692**	**5.341**
Pseudachorutes	Eudorylaimus	**0.977**	**-0.166**	**3.692**	**4.864**
Pseudachorutes	Thornematidae	**0.977**	**-0.470**	**3.692**	**4.864**
Pseudachorutes	Eupodes	**0.977**	**0.005**	**3.692**	**4.118**
Pseudachorutes	Nenteria	**0.977**	**0.254**	**3.692**	**3.516**
Pseudachorutes	Scutacarus	**0.977**	**-0.608**	**3.692**	**4.215**
Pseudachorutes	Tarsonemus	**0.977**	**-0.701**	**3.692**	**3.516**
Achaeta	Arctoseius	**0.515**	**-0.152**	**3.233**	**3.516**
Achaeta	Cheiroseius	**0.515**	**0.356**	**3.233**	**3.215**
Achaeta	Lysigamasus	**0.515**	**0.407**	**3.233**	**3.692**
Achaeta	Uropoda	**0.515**	**0.481**	**3.233**	**3.215**
Achaeta	Aporcelaimellus	**0.515**	**0.548**	**3.233**	**5.341**
Achaeta	Dorylaimoidea	**0.515**	**-0.604**	**3.233**	**5.341**
Achaeta	Eudorylaimus	**0.515**	**-0.166**	**3.233**	**4.864**
Achaeta	Thornematidae	**0.515**	**-0.470**	**3.233**	**4.864**
Achaeta	Eupodes	**0.515**	**0.005**	**3.233**	**4.118**
Achaeta	Nenteria	**0.515**	**0.254**	**3.233**	**3.516**
Achaeta	Scutacarus	**0.515**	**-0.608**	**3.233**	**4.215**
Achaeta	Tarsonemus	**0.515**	**-0.701**	**3.233**	**3.516**
Cognettia	Arctoseius	**1.231**	**-0.152**	**3.186**	**3.516**
Cognettia	Cheiroseius	**1.231**	**0.356**	**3.186**	**3.215**

Cognettia	Lysigamasus	**1.231**	**0.407**	**3.186**	**3.692**
Cognettia	Uropoda	**1.231**	**0.481**	**3.186**	**3.215**
Cognettia	Aporcelaimellus	**1.231**	**0.548**	**3.186**	**5.341**
Cognettia	Dorylaimoidea	**1.231**	**-0.604**	**3.186**	**5.341**
Cognettia	Eudorylaimus	**1.231**	**-0.166**	**3.186**	**4.864**
Cognettia	Thornematidae	**1.231**	**-0.470**	**3.186**	**4.864**
Cognettia	Eupodes	**1.231**	**0.005**	**3.186**	**4.118**
Cognettia	Nenteria	**1.231**	**0.254**	**3.186**	**3.516**
Cognettia	Scutacarus	**1.231**	**-0.608**	**3.186**	**4.215**
Cognettia	Tarsonemus	**1.231**	**-0.701**	**3.186**	**3.516**
Fridericia	Arctoseius	**2.002**	**-0.152**	**3.486**	**3.516**
Fridericia	Cheiroseius	**2.002**	**0.356**	**3.486**	**3.215**
Fridericia	Lysigamasus	**2.002**	**0.407**	**3.486**	**3.692**
Fridericia	Uropoda	**2.002**	**0.481**	**3.486**	**3.215**
Fridericia	Aporcelaimellus	**2.002**	**0.548**	**3.486**	**5.341**
Fridericia	Dorylaimoidea	**2.002**	**-0.604**	**3.486**	**5.341**
Fridericia	Eudorylaimus	**2.002**	**-0.166**	**3.486**	**4.864**
Fridericia	Thornematidae	**2.002**	**-0.470**	**3.486**	**4.864**
Fridericia	Eupodes	**2.002**	**0.005**	**3.486**	**4.118**
Fridericia	Nenteria	**2.002**	**0.254**	**3.486**	**3.516**
Fridericia	Scutacarus	**2.002**	**-0.608**	**3.486**	**4.215**
Fridericia	Tarsonemus	**2.002**	**-0.701**	**3.486**	**3.516**
Acrobeloides	Mylonchulus	**-1.171**	**-0.005**	**5.563**	**4.563**
Acrobeloides	Tripyla	**-1.171**	**-0.420**	**5.563**	**4.563**
Acrobeloides	Alliphis	**-1.171**	**0.053**	**5.563**	**3.215**
Acrobeloides	Arctoseius	**-1.171**	**-0.152**	**5.563**	**3.516**
Acrobeloides	Cheiroseius	**-1.171**	**0.356**	**5.563**	**3.215**
Acrobeloides	Lysigamasus	**-1.171**	**0.407**	**5.563**	**3.692**
Acrobeloides	Uropoda	**-1.171**	**0.481**	**5.563**	**3.215**
Acrobeloides	Aporcelaimellus	**-1.171**	**0.548**	**5.563**	**5.341**
Acrobeloides	Dorylaimoidea	**-1.171**	**-0.604**	**5.563**	**5.341**
Acrobeloides	Eudorylaimus	**-1.171**	**-0.166**	**5.563**	**4.864**
Acrobeloides	Thornematidae	**-1.171**	**-0.470**	**5.563**	**4.864**
Acrobeloides	Eupodes	**-1.171**	**0.005**	**5.563**	**4.118**
Acrobeloides	Nenteria	**-1.171**	**0.254**	**5.563**	**3.516**
Acrobeloides	Scutacarus	**-1.171**	**-0.608**	**5.563**	**4.215**
Acrobeloides	Tarsonemus	**-1.171**	**-0.701**	**5.563**	**3.516**
Anaplectus	Mylonchulus	**-0.519**	**-0.005**	**4.864**	**4.563**
Anaplectus	Tripyla	**-0.519**	**-0.420**	**4.864**	**4.563**
Anaplectus	Alliphis	**-0.519**	**0.053**	**4.864**	**3.215**
Anaplectus	Arctoseius	**-0.519**	**-0.152**	**4.864**	**3.516**
Anaplectus	Cheiroseius	**-0.519**	**0.356**	**4.864**	**3.215**
Anaplectus	Lysigamasus	**-0.519**	**0.407**	**4.864**	**3.692**
Anaplectus	Uropoda	**-0.519**	**0.481**	**4.864**	**3.215**
Anaplectus	Aporcelaimellus	**-0.519**	**0.548**	**4.864**	**5.341**
Anaplectus	Dorylaimoidea	**-0.519**	**-0.604**	**4.864**	**5.341**
Anaplectus	Eudorylaimus	**-0.519**	**-0.166**	**4.864**	**4.864**
Anaplectus	Thornematidae	**-0.519**	**-0.470**	**4.864**	**4.864**
Anaplectus	Eupodes	**-0.519**	**0.005**	**4.864**	**4.118**
Anaplectus	Nenteria	**-0.519**	**0.254**	**4.864**	**3.516**
Anaplectus	Scutacarus	**-0.519**	**-0.608**	**4.864**	**4.215**

Anaplectus	Tarsonemus	**-0.519**	**-0.701**	**4.864**	**3.516**
Cephalobidae	Mylonchulus	**-1.055**	**-0.005**	**4.563**	**4.563**
Cephalobidae	Tripyla	**-1.055**	**-0.420**	**4.563**	**4.563**
Cephalobidae	Alliphis	**-1.055**	**0.053**	**4.563**	**3.215**
Cephalobidae	Arctoseius	**-1.055**	**-0.152**	**4.563**	**3.516**
Cephalobidae	Cheiroseius	**-1.055**	**0.356**	**4.563**	**3.215**
Cephalobidae	Lysigamasus	**-1.055**	**0.407**	**4.563**	**3.692**
Cephalobidae	Uropoda	**-1.055**	**0.481**	**4.563**	**3.215**
Cephalobidae	Aporcelaimellus	**-1.055**	**0.548**	**4.563**	**5.341**
Cephalobidae	Dorylaimoidea	**-1.055**	**-0.604**	**4.563**	**5.341**
Cephalobidae	Eudorylaimus	**-1.055**	**-0.166**	**4.563**	**4.864**
Cephalobidae	Thornematidae	**-1.055**	**-0.470**	**4.563**	**4.864**
Cephalobidae	Eupodes	**-1.055**	**0.005**	**4.563**	**4.118**
Cephalobidae	Nenteria	**-1.055**	**0.254**	**4.563**	**3.516**
Cephalobidae	Scutacarus	**-1.055**	**-0.608**	**4.563**	**4.215**
Cephalobidae	Tarsonemus	**-1.055**	**-0.701**	**4.563**	**3.516**
Eucephalobus	Mylonchulus	**-0.855**	**-0.005**	**5.842**	**4.563**
Eucephalobus	Tripyla	**-0.855**	**-0.420**	**5.842**	**4.563**
Eucephalobus	Alliphis	**-0.855**	**0.053**	**5.842**	**3.215**
Eucephalobus	Arctoseius	**-0.855**	**-0.152**	**5.842**	**3.516**
Eucephalobus	Cheiroseius	**-0.855**	**0.356**	**5.842**	**3.215**
Eucephalobus	Lysigamasus	**-0.855**	**0.407**	**5.842**	**3.692**
Eucephalobus	Uropoda	**-0.855**	**0.481**	**5.842**	**3.215**
Eucephalobus	Aporcelaimellus	**-0.855**	**0.548**	**5.842**	**5.341**
Eucephalobus	Dorylaimoidea	**-0.855**	**-0.604**	**5.842**	**5.341**
Eucephalobus	Eudorylaimus	**-0.855**	**-0.166**	**5.842**	**4.864**
Eucephalobus	Thornematidae	**-0.855**	**-0.470**	**5.842**	**4.864**
Eucephalobus	Eupodes	**-0.855**	**0.005**	**5.842**	**4.118**
Eucephalobus	Nenteria	**-0.855**	**0.254**	**5.842**	**3.516**
Eucephalobus	Scutacarus	**-0.855**	**-0.608**	**5.842**	**4.215**
Eucephalobus	Tarsonemus	**-0.855**	**-0.701**	**5.842**	**3.516**
Metateratocephalus	Mylonchulus	**-1.506**	**-0.005**	**5.040**	**4.563**
Metateratocephalus	Tripyla	**-1.506**	**-0.420**	**5.040**	**4.563**
Metateratocephalus	Alliphis	**-1.506**	**0.053**	**5.040**	**3.215**
Metateratocephalus	Arctoseius	**-1.506**	**-0.152**	**5.040**	**3.516**
Metateratocephalus	Cheiroseius	**-1.506**	**0.356**	**5.040**	**3.215**
Metateratocephalus	Lysigamasus	**-1.506**	**0.407**	**5.040**	**3.692**
Metateratocephalus	Uropoda	**-1.506**	**0.481**	**5.040**	**3.215**
Metateratocephalus	Aporcelaimellus	**-1.506**	**0.548**	**5.040**	**5.341**
Metateratocephalus	Dorylaimoidea	**-1.506**	**-0.604**	**5.040**	**5.341**
Metateratocephalus	Eudorylaimus	**-1.506**	**-0.166**	**5.040**	**4.864**
Metateratocephalus	Thornematidae	**-1.506**	**-0.470**	**5.040**	**4.864**
Metateratocephalus	Eupodes	**-1.506**	**0.005**	**5.040**	**4.118**
Metateratocephalus	Nenteria	**-1.506**	**0.254**	**5.040**	**3.516**
Metateratocephalus	Scutacarus	**-1.506**	**-0.608**	**5.040**	**4.215**
Metateratocephalus	Tarsonemus	**-1.506**	**-0.701**	**5.040**	**3.516**
Monhysteridae	Mylonchulus	**-0.600**	**-0.005**	**4.563**	**4.563**
Monhysteridae	Tripyla	**-0.600**	**-0.420**	**4.563**	**4.563**
Monhysteridae	Alliphis	**-0.600**	**0.053**	**4.563**	**3.215**
Monhysteridae	Arctoseius	**-0.600**	**-0.152**	**4.563**	**3.516**
Monhysteridae	Cheiroseius	**-0.600**	**0.356**	**4.563**	**3.215**

Monhysteridae	Lysigamasus	**-0.600**	**0.407**	**4.563**	**3.692**
Monhysteridae	Uropoda	**-0.600**	**0.481**	**4.563**	**3.215**
Monhysteridae	Aporcelaimellus	**-0.600**	**0.548**	**4.563**	**5.341**
Monhysteridae	Dorylaimoidea	**-0.600**	**-0.604**	**4.563**	**5.341**
Monhysteridae	Eudorylaimus	**-0.600**	**-0.166**	**4.563**	**4.864**
Monhysteridae	Thornematidae	**-0.600**	**-0.470**	**4.563**	**4.864**
Monhysteridae	Eupodes	**-0.600**	**0.005**	**4.563**	**4.118**
Monhysteridae	Nenteria	**-0.600**	**0.254**	**4.563**	**3.516**
Monhysteridae	Scutacarus	**-0.600**	**-0.608**	**4.563**	**4.215**
Monhysteridae	Tarsonemus	**-0.600**	**-0.701**	**4.563**	**3.516**
Panagrolaimus	Mylonchulus	**-0.945**	**-0.005**	**5.563**	**4.563**
Panagrolaimus	Tripyla	**-0.945**	**-0.420**	**5.563**	**4.563**
Panagrolaimus	Alliphis	**-0.945**	**0.053**	**5.563**	**3.215**
Panagrolaimus	Arctoseius	**-0.945**	**-0.152**	**5.563**	**3.516**
Panagrolaimus	Cheiroseius	**-0.945**	**0.356**	**5.563**	**3.215**
Panagrolaimus	Lysigamasus	**-0.945**	**0.407**	**5.563**	**3.692**
Panagrolaimus	Uropoda	**-0.945**	**0.481**	**5.563**	**3.215**
Panagrolaimus	Aporcelaimellus	**-0.945**	**0.548**	**5.563**	**5.341**
Panagrolaimus	Dorylaimoidea	**-0.945**	**-0.604**	**5.563**	**5.341**
Panagrolaimus	Eudorylaimus	**-0.945**	**-0.166**	**5.563**	**4.864**
Panagrolaimus	Thornematidae	**-0.945**	**-0.470**	**5.563**	**4.864**
Panagrolaimus	Eupodes	**-0.945**	**0.005**	**5.563**	**4.118**
Panagrolaimus	Nenteria	**-0.945**	**0.254**	**5.563**	**3.516**
Panagrolaimus	Scutacarus	**-0.945**	**-0.608**	**5.563**	**4.215**
Panagrolaimus	Tarsonemus	**-0.945**	**-0.701**	**5.563**	**3.516**
Plectus	Mylonchulus	**-0.583**	**-0.005**	**5.642**	**4.563**
Plectus	Tripyla	**-0.583**	**-0.420**	**5.642**	**4.563**
Plectus	Alliphis	**-0.583**	**0.053**	**5.642**	**3.215**
Plectus	Arctoseius	**-0.583**	**-0.152**	**5.642**	**3.516**
Plectus	Cheiroseius	**-0.583**	**0.356**	**5.642**	**3.215**
Plectus	Lysigamasus	**-0.583**	**0.407**	**5.642**	**3.692**
Plectus	Uropoda	**-0.583**	**0.481**	**5.642**	**3.215**
Plectus	Aporcelaimellus	**-0.583**	**0.548**	**5.642**	**5.341**
Plectus	Dorylaimoidea	**-0.583**	**-0.604**	**5.642**	**5.341**
Plectus	Eudorylaimus	**-0.583**	**-0.166**	**5.642**	**4.864**
Plectus	Thornematidae	**-0.583**	**-0.470**	**5.642**	**4.864**
Plectus	Eupodes	**-0.583**	**0.005**	**5.642**	**4.118**
Plectus	Nenteria	**-0.583**	**0.254**	**5.642**	**3.516**
Plectus	Scutacarus	**-0.583**	**-0.608**	**5.642**	**4.215**
Plectus	Tarsonemus	**-0.583**	**-0.701**	**5.642**	**3.516**
Prismatolaimus	Mylonchulus	**-1.280**	**-0.005**	**4.864**	**4.563**
Prismatolaimus	Tripyla	**-1.280**	**-0.420**	**4.864**	**4.563**
Prismatolaimus	Alliphis	**-1.280**	**0.053**	**4.864**	**3.215**
Prismatolaimus	Arctoseius	**-1.280**	**-0.152**	**4.864**	**3.516**
Prismatolaimus	Cheiroseius	**-1.280**	**0.356**	**4.864**	**3.215**
Prismatolaimus	Lysigamasus	**-1.280**	**0.407**	**4.864**	**3.692**
Prismatolaimus	Uropoda	**-1.280**	**0.481**	**4.864**	**3.215**
Prismatolaimus	Aporcelaimellus	**-1.280**	**0.548**	**4.864**	**5.341**
Prismatolaimus	Dorylaimoidea	**-1.280**	**-0.604**	**4.864**	**5.341**
Prismatolaimus	Eudorylaimus	**-1.280**	**-0.166**	**4.864**	**4.864**
Prismatolaimus	Thornematidae	**-1.280**	**-0.470**	**4.864**	**4.864**

Prismatolaimus	Eupodes	**-1.280**	**0.005**	**4.864**	**4.118**
Prismatolaimus	Nenteria	**-1.280**	**0.254**	**4.864**	**3.516**
Prismatolaimus	Scutacarus	**-1.280**	**-0.608**	**4.864**	**4.215**
Prismatolaimus	Tarsonemus	**-1.280**	**-0.701**	**4.864**	**3.516**
Rhabditidae	Mylonchulus	**-0.692**	**-0.005**	**5.466**	**4.563**
Rhabditidae	Tripyla	**-0.692**	**-0.420**	**5.466**	**4.563**
Rhabditidae	Alliphis	**-0.692**	**0.053**	**5.466**	**3.215**
Rhabditidae	Arctoseius	**-0.692**	**-0.152**	**5.466**	**3.516**
Rhabditidae	Cheiroseius	**-0.692**	**0.356**	**5.466**	**3.215**
Rhabditidae	Lysigamasus	**-0.692**	**0.407**	**5.466**	**3.692**
Rhabditidae	Uropoda	**-0.692**	**0.481**	**5.466**	**3.215**
Rhabditidae	Aporcelaimellus	**-0.692**	**0.548**	**5.466**	**5.341**
Rhabditidae	Dorylaimoidea	**-0.692**	**-0.604**	**5.466**	**5.341**
Rhabditidae	Eudorylaimus	**-0.692**	**-0.166**	**5.466**	**4.864**
Rhabditidae	Thornematidae	**-0.692**	**-0.470**	**5.466**	**4.864**
Rhabditidae	Eupodes	**-0.692**	**0.005**	**5.466**	**4.118**
Rhabditidae	Nenteria	**-0.692**	**0.254**	**5.466**	**3.516**
Rhabditidae	Scutacarus	**-0.692**	**-0.608**	**5.466**	**4.215**
Rhabditidae	Tarsonemus	**-0.692**	**-0.701**	**5.466**	**3.516**
Teratocephalus	Mylonchulus	**-1.630**	**-0.005**	**5.604**	**4.563**
Teratocephalus	Tripyla	**-1.630**	**-0.420**	**5.604**	**4.563**
Teratocephalus	Alliphis	**-1.630**	**0.053**	**5.604**	**3.215**
Teratocephalus	Arctoseius	**-1.630**	**-0.152**	**5.604**	**3.516**
Teratocephalus	Cheiroseius	**-1.630**	**0.356**	**5.604**	**3.215**
Teratocephalus	Lysigamasus	**-1.630**	**0.407**	**5.604**	**3.692**
Teratocephalus	Uropoda	**-1.630**	**0.481**	**5.604**	**3.215**
Teratocephalus	Aporcelaimellus	**-1.630**	**0.548**	**5.604**	**5.341**
Teratocephalus	Dorylaimoidea	**-1.630**	**-0.604**	**5.604**	**5.341**
Teratocephalus	Eudorylaimus	**-1.630**	**-0.166**	**5.604**	**4.864**
Teratocephalus	Thornematidae	**-1.630**	**-0.470**	**5.604**	**4.864**
Teratocephalus	Eupodes	**-1.630**	**0.005**	**5.604**	**4.118**
Teratocephalus	Nenteria	**-1.630**	**0.254**	**5.604**	**3.516**
Teratocephalus	Scutacarus	**-1.630**	**-0.608**	**5.604**	**4.215**
Teratocephalus	Tarsonemus	**-1.630**	**-0.701**	**5.604**	**3.516**
Enchytraeus	Arctoseius	**1.090**	**-0.152**	**3.202**	**3.516**
Enchytraeus	Cheiroseius	**1.090**	**0.356**	**3.202**	**3.215**
Enchytraeus	Lysigamasus	**1.090**	**0.407**	**3.202**	**3.692**
Enchytraeus	Uropoda	**1.090**	**0.481**	**3.202**	**3.215**
Enchytraeus	Aporcelaimellus	**1.090**	**0.548**	**3.202**	**5.341**
Enchytraeus	Dorylaimoidea	**1.090**	**-0.604**	**3.202**	**5.341**
Enchytraeus	Eudorylaimus	**1.090**	**-0.166**	**3.202**	**4.864**
Enchytraeus	Thornematidae	**1.090**	**-0.470**	**3.202**	**4.864**
Enchytraeus	Eupodes	**1.090**	**0.005**	**3.202**	**4.118**
Enchytraeus	Nenteria	**1.090**	**0.254**	**3.202**	**3.516**
Enchytraeus	Scutacarus	**1.090**	**-0.608**	**3.202**	**4.215**
Enchytraeus	Tarsonemus	**1.090**	**-0.701**	**3.202**	**3.516**
Eubacteria	Acrobeloides	**-6.573**	**-1.171**	**12.346**	**5.563**
Eubacteria	Anaplectus	**-6.573**	**-0.519**	**12.346**	**4.864**
Eubacteria	Cephalobidae	**-6.573**	**-1.055**	**12.346**	**4.563**
Eubacteria	Eucephalobus	**-6.573**	**-0.855**	**12.346**	**5.842**
Eubacteria	Metateratocephalus	**-6.573**	**-1.506**	**12.346**	**5.040**

Eubacteria	Monhysteridae	**-6.573**	**-0.600**	**12.346**	**4.563**
Eubacteria	Panagrolaimus	**-6.573**	**-0.945**	**12.346**	**5.563**
Eubacteria	Plectus	**-6.573**	**-0.583**	**12.346**	**5.642**
Eubacteria	Prismatolaimus	**-6.573**	**-1.280**	**12.346**	**4.864**
Eubacteria	Rhabditidae	**-6.573**	**-0.692**	**12.346**	**5.466**
Eubacteria	Teratocephalus	**-6.573**	**-1.630**	**12.346**	**5.604**
Eubacteria	Enchytraeus	**-6.573**	**1.090**	**12.346**	**3.202**
Eubacteria	Dauerlarvae	**-6.573**	**-0.804**	**12.346**	**4.864**
Eubacteria	Henlea	**-6.573**	**1.621**	**12.346**	**3.262**
Eubacteria	Marionina	**-6.573**	**1.188**	**12.346**	**3.628**
Dauerlarvae	Mylonchulus	**-0.804**	**-0.005**	**4.864**	**4.563**
Dauerlarvae	Tripyla	**-0.804**	**-0.420**	**4.864**	**4.563**
Dauerlarvae	Alliphis	**-0.804**	**0.053**	**4.864**	**3.215**
Dauerlarvae	Aporcelaimellus	**-0.804**	**0.548**	**4.864**	**5.341**
Dauerlarvae	Dorylaimoidea	**-0.804**	**-0.604**	**4.864**	**5.341**
Dauerlarvae	Eudorylaimus	**-0.804**	**-0.166**	**4.864**	**4.864**
Dauerlarvae	Thornematidae	**-0.804**	**-0.470**	**4.864**	**4.864**
Dauerlarvae	Eupodes	**-0.804**	**0.005**	**4.864**	**4.118**
Dauerlarvae	Nenteria	**-0.804**	**0.254**	**4.864**	**3.516**
Dauerlarvae	Scutacarus	**-0.804**	**-0.608**	**4.864**	**4.215**
Dauerlarvae	Tarsonemus	**-0.804**	**-0.701**	**4.864**	**3.516**
Henlea	Arctoseius	**1.621**	**-0.152**	**3.262**	**3.516**
Henlea	Cheiroseius	**1.621**	**0.356**	**3.262**	**3.215**
Henlea	Lysigamasus	**1.621**	**0.407**	**3.262**	**3.692**
Henlea	Uropoda	**1.621**	**0.481**	**3.262**	**3.215**
Henlea	Aporcelaimellus	**1.621**	**0.548**	**3.262**	**5.341**
Henlea	Dorylaimoidea	**1.621**	**-0.604**	**3.262**	**5.341**
Henlea	Eudorylaimus	**1.621**	**-0.166**	**3.262**	**4.864**
Henlea	Thornematidae	**1.621**	**-0.470**	**3.262**	**4.864**
Henlea	Eupodes	**1.621**	**0.005**	**3.262**	**4.118**
Henlea	Nenteria	**1.621**	**0.254**	**3.262**	**3.516**
Henlea	Scutacarus	**1.621**	**-0.608**	**3.262**	**4.215**
Henlea	Tarsonemus	**1.621**	**-0.701**	**3.262**	**3.516**
Marionina	Arctoseius	**1.188**	**-0.152**	**3.628**	**3.516**
Marionina	Cheiroseius	**1.188**	**0.356**	**3.628**	**3.215**
Marionina	Lysigamasus	**1.188**	**0.407**	**3.628**	**3.692**
Marionina	Uropoda	**1.188**	**0.481**	**3.628**	**3.215**
Marionina	Aporcelaimellus	**1.188**	**0.548**	**3.628**	**5.341**
Marionina	Dorylaimoidea	**1.188**	**-0.604**	**3.628**	**5.341**
Marionina	Eudorylaimus	**1.188**	**-0.166**	**3.628**	**4.864**
Marionina	Thornematidae	**1.188**	**-0.470**	**3.628**	**4.864**
Marionina	Eupodes	**1.188**	**0.005**	**3.628**	**4.118**
Marionina	Nenteria	**1.188**	**0.254**	**3.628**	**3.516**
Marionina	Scutacarus	**1.188**	**-0.608**	**3.628**	**4.215**
Marionina	Tarsonemus	**1.188**	**-0.701**	**3.628**	**3.516**
Hyphae and hair roots	Coslenchus	**5.904**	**-0.821**	**0.000**	**4.563**
Hyphae and hair roots	Dolichodoridae	**5.904**	**-0.885**	**0.000**	**5.408**
Hyphae and hair roots	Helicotylenchus	**5.904**	**-0.792**	**0.000**	**5.341**
Hyphae and hair roots	Paratylenchus	**5.904**	**-1.244**	**0.000**	**4.864**
Hyphae and hair roots	Pratylenchus	**5.904**	**-1.226**	**0.000**	**5.040**
Hyphae and hair roots	Tylenchorhynchus	**5.904**	**-0.664**	**0.000**	**5.341**

Hyphae and hair roots	Pachygnatidae	**5.904**	**-0.113**	**0.000**	**3.516**
Hyphae and hair roots	Platynothrus	**5.904**	**0.710**	**0.000**	**3.993**
Hyphae and hair roots	Tydeidae	**5.904**	**-0.608**	**0.000**	**3.692**
Hyphae and hair roots	Sminthuridae	**5.904**	**-0.608**	**0.000**	**3.516**
Hyphae and hair roots	Sminthurinus	**5.904**	**0.618**	**0.000**	**3.692**
Hyphae and hair roots	Sphaeridia	**5.904**	**0.202**	**0.000**	**3.516**
Hyphae and hair roots	Aphelenchoides	**5.904**	**-1.496**	**0.000**	**5.341**
Hyphae and hair roots	Tylenchidae	**5.904**	**-1.360**	**0.000**	**5.864**
Hyphae and hair roots	Microtydeus	**5.904**	**-0.863**	**0.000**	**3.817**
Hyphae and hair roots	Lepidocyrtus	**5.904**	**1.231**	**0.000**	**4.294**
Hyphae and hair roots	Parisotoma	**5.904**	**0.722**	**0.000**	**3.692**
Hyphae and hair roots	Pseudachorutes	**5.904**	**0.977**	**0.000**	**3.692**
Hyphae and hair roots	Achaeta	**5.904**	**0.515**	**0.000**	**3.233**
Hyphae and hair roots	Cognettia	**5.904**	**1.231**	**0.000**	**3.186**
Hyphae and hair roots	Fridericia	**5.904**	**2.002**	**0.000**	**3.486**
Hyphae and hair roots	Aporcelaimellus	**5.904**	**0.548**	**0.000**	**5.341**
Hyphae and hair roots	Dorylaimoidea	**5.904**	**-0.604**	**0.000**	**5.341**
Hyphae and hair roots	Eudorylaimus	**5.904**	**-0.166**	**0.000**	**4.864**
Hyphae and hair roots	Thornematidae	**5.904**	**-0.470**	**0.000**	**4.864**
Hyphae and hair roots	Eupodes	**5.904**	**0.005**	**0.000**	**4.118**
Hyphae and hair roots	Nenteria	**5.904**	**0.254**	**0.000**	**3.516**
Hyphae and hair roots	Scutacarus	**5.904**	**-0.608**	**0.000**	**4.215**
Hyphae and hair roots	Tarsonemus	**5.904**	**-0.701**	**0.000**	**3.516**
Mylonchulus	Arctoseius	**-0.005**	**-0.152**	**4.563**	**3.516**
Mylonchulus	Cheiroseius	**-0.005**	**0.356**	**4.563**	**3.215**
Mylonchulus	Lysigamasus	**-0.005**	**0.407**	**4.563**	**3.692**
Mylonchulus	Uropoda	**-0.005**	**0.481**	**4.563**	**3.215**
Mylonchulus	Aporcelaimellus	**-0.005**	**0.548**	**4.563**	**5.341**
Mylonchulus	Dorylaimoidea	**-0.005**	**-0.604**	**4.563**	**5.341**
Mylonchulus	Eudorylaimus	**-0.005**	**-0.166**	**4.563**	**4.864**
Mylonchulus	Thornematidae	**-0.005**	**-0.470**	**4.563**	**4.864**
Mylonchulus	Eupodes	**-0.005**	**0.005**	**4.563**	**4.118**
Mylonchulus	Nenteria	**-0.005**	**0.254**	**4.563**	**3.516**
Mylonchulus	Scutacarus	**-0.005**	**-0.608**	**4.563**	**4.215**
Mylonchulus	Tarsonemus	**-0.005**	**-0.701**	**4.563**	**3.516**
Tripyla	Arctoseius	**-0.420**	**-0.152**	**4.563**	**3.516**
Tripyla	Cheiroseius	**-0.420**	**0.356**	**4.563**	**3.215**
Tripyla	Lysigamasus	**-0.420**	**0.407**	**4.563**	**3.692**
Tripyla	Uropoda	**-0.420**	**0.481**	**4.563**	**3.215**
Tripyla	Aporcelaimellus	**-0.420**	**0.548**	**4.563**	**5.341**
Tripyla	Dorylaimoidea	**-0.420**	**-0.604**	**4.563**	**5.341**
Tripyla	Eudorylaimus	**-0.420**	**-0.166**	**4.563**	**4.864**
Tripyla	Thornematidae	**-0.420**	**-0.470**	**4.563**	**4.864**
Tripyla	Eupodes	**-0.420**	**0.005**	**4.563**	**4.118**
Tripyla	Nenteria	**-0.420**	**0.254**	**4.563**	**3.516**
Tripyla	Scutacarus	**-0.420**	**-0.608**	**4.563**	**4.215**
Tripyla	Tarsonemus	**-0.420**	**-0.701**	**4.563**	**3.516**
Alliphis	Arctoseius	**0.053**	**-0.152**	**3.215**	**3.516**
Alliphis	Cheiroseius	**0.053**	**0.356**	**3.215**	**3.215**
Alliphis	Lysigamasus	**0.053**	**0.407**	**3.215**	**3.692**
Alliphis	Uropoda	**0.053**	**0.481**	**3.215**	**3.215**

Alliphis	Aporcelaimellus	**0.053**	**0.548**	**3.215**	**5.341**
Alliphis	Dorylaimoidea	**0.053**	**-0.604**	**3.215**	**5.341**
Alliphis	Eudorylaimus	**0.053**	**-0.166**	**3.215**	**4.864**
Alliphis	Thornematidae	**0.053**	**-0.470**	**3.215**	**4.864**
Alliphis	Eupodes	**0.053**	**0.005**	**3.215**	**4.118**
Alliphis	Nenteria	**0.053**	**0.254**	**3.215**	**3.516**
Alliphis	Scutacarus	**0.053**	**-0.608**	**3.215**	**4.215**
Alliphis	Tarsonemus	**0.053**	**-0.701**	**3.215**	**3.516**
Arctoseius	Aporcelaimellus	**-0.152**	**0.548**	**3.516**	**5.341**
Arctoseius	Dorylaimoidea	**-0.152**	**-0.604**	**3.516**	**5.341**
Arctoseius	Eudorylaimus	**-0.152**	**-0.166**	**3.516**	**4.864**
Arctoseius	Thornematidae	**-0.152**	**-0.470**	**3.516**	**4.864**
Arctoseius	Eupodes	**-0.152**	**0.005**	**3.516**	**4.118**
Arctoseius	Nenteria	**-0.152**	**0.254**	**3.516**	**3.516**
Arctoseius	Scutacarus	**-0.152**	**-0.608**	**3.516**	**4.215**
Arctoseius	Tarsonemus	**-0.152**	**-0.701**	**3.516**	**3.516**
Cheiroseius	Aporcelaimellus	**0.356**	**0.548**	**3.215**	**5.341**
Cheiroseius	Dorylaimoidea	**0.356**	**-0.604**	**3.215**	**5.341**
Cheiroseius	Eudorylaimus	**0.356**	**-0.166**	**3.215**	**4.864**
Cheiroseius	Thornematidae	**0.356**	**-0.470**	**3.215**	**4.864**
Cheiroseius	Eupodes	**0.356**	**0.005**	**3.215**	**4.118**
Cheiroseius	Nenteria	**0.356**	**0.254**	**3.215**	**3.516**
Cheiroseius	Scutacarus	**0.356**	**-0.608**	**3.215**	**4.215**
Cheiroseius	Tarsonemus	**0.356**	**-0.701**	**3.215**	**3.516**
Lysigamasus	Aporcelaimellus	**0.407**	**0.548**	**3.692**	**5.341**
Lysigamasus	Dorylaimoidea	**0.407**	**-0.604**	**3.692**	**5.341**
Lysigamasus	Eudorylaimus	**0.407**	**-0.166**	**3.692**	**4.864**
Lysigamasus	Thornematidae	**0.407**	**-0.470**	**3.692**	**4.864**
Lysigamasus	Eupodes	**0.407**	**0.005**	**3.692**	**4.118**
Lysigamasus	Nenteria	**0.407**	**0.254**	**3.692**	**3.516**
Lysigamasus	Scutacarus	**0.407**	**-0.608**	**3.692**	**4.215**
Lysigamasus	Tarsonemus	**0.407**	**-0.701**	**3.692**	**3.516**
Uropoda	Aporcelaimellus	**0.481**	**0.548**	**3.215**	**5.341**
Uropoda	Dorylaimoidea	**0.481**	**-0.604**	**3.215**	**5.341**
Uropoda	Eudorylaimus	**0.481**	**-0.166**	**3.215**	**4.864**
Uropoda	Thornematidae	**0.481**	**-0.470**	**3.215**	**4.864**
Uropoda	Eupodes	**0.481**	**0.005**	**3.215**	**4.118**
Uropoda	Nenteria	**0.481**	**0.254**	**3.215**	**3.516**
Uropoda	Scutacarus	**0.481**	**-0.608**	**3.215**	**4.215**
Uropoda	Tarsonemus	**0.481**	**-0.701**	**3.215**	**3.516**
Aporcelaimellus	Mylonchulus	**0.548**	**-0.005**	**5.341**	**4.563**
Aporcelaimellus	Tripyla	**0.548**	**-0.420**	**5.341**	**4.563**
Aporcelaimellus	Alliphis	**0.548**	**0.053**	**5.341**	**3.215**
Aporcelaimellus	Arctoseius	**0.548**	**-0.152**	**5.341**	**3.516**
Aporcelaimellus	Cheiroseius	**0.548**	**0.356**	**5.341**	**3.215**
Aporcelaimellus	Lysigamasus	**0.548**	**0.407**	**5.341**	**3.692**
Aporcelaimellus	Uropoda	**0.548**	**0.481**	**5.341**	**3.215**
Aporcelaimellus	Aporcelaimellus	**0.548**	**0.548**	**5.341**	**5.341**
Aporcelaimellus	Dorylaimoidea	**0.548**	**-0.604**	**5.341**	**5.341**
Aporcelaimellus	Eudorylaimus	**0.548**	**-0.166**	**5.341**	**4.864**
Aporcelaimellus	Thornematidae	**0.548**	**-0.470**	**5.341**	**4.864**

Aporcelaimellus	Eupodes	0.548	0.005	5.341	4.118
Aporcelaimellus	Nenteria	0.548	0.254	5.341	3.516
Aporcelaimellus	Scutacarus	0.548	-0.608	5.341	4.215
Aporcelaimellus	Tarsonemus	0.548	-0.701	5.341	3.516
Dorylaimoidea	Mylonchulus	-0.604	-0.005	5.341	4.563
Dorylaimoidea	Tripyla	-0.604	-0.420	5.341	4.563
Dorylaimoidea	Alliphis	-0.604	0.053	5.341	3.215
Dorylaimoidea	Arctoseius	-0.604	-0.152	5.341	3.516
Dorylaimoidea	Cheiroseius	-0.604	0.356	5.341	3.215
Dorylaimoidea	Lysigamasus	-0.604	0.407	5.341	3.692
Dorylaimoidea	Uropoda	-0.604	0.481	5.341	3.215
Dorylaimoidea	Aporcelaimellus	-0.604	0.548	5.341	5.341
Dorylaimoidea	Dorylaimoidea	-0.604	-0.604	5.341	5.341
Dorylaimoidea	Eudorylaimus	-0.604	-0.166	5.341	4.864
Dorylaimoidea	Thornematidae	-0.604	-0.470	5.341	4.864
Dorylaimoidea	Eupodes	-0.604	0.005	5.341	4.118
Dorylaimoidea	Nenteria	-0.604	0.254	5.341	3.516
Dorylaimoidea	Scutacarus	-0.604	-0.608	5.341	4.215
Dorylaimoidea	Tarsonemus	-0.604	-0.701	5.341	3.516
Eudorylaimus	Mylonchulus	-0.166	-0.005	4.864	4.563
Eudorylaimus	Tripyla	-0.166	-0.420	4.864	4.563
Eudorylaimus	Alliphis	-0.166	0.053	4.864	3.215
Eudorylaimus	Arctoseius	-0.166	-0.152	4.864	3.516
Eudorylaimus	Cheiroseius	-0.166	0.356	4.864	3.215
Eudorylaimus	Lysigamasus	-0.166	0.407	4.864	3.692
Eudorylaimus	Uropoda	-0.166	0.481	4.864	3.215
Eudorylaimus	Aporcelaimellus	-0.166	0.548	4.864	5.341
Eudorylaimus	Dorylaimoidea	-0.166	-0.604	4.864	5.341
Eudorylaimus	Eudorylaimus	-0.166	-0.166	4.864	4.864
Eudorylaimus	Thornematidae	-0.166	-0.470	4.864	4.864
Eudorylaimus	Eupodes	-0.166	0.005	4.864	4.118
Eudorylaimus	Nenteria	-0.166	0.254	4.864	3.516
Eudorylaimus	Scutacarus	-0.166	-0.608	4.864	4.215
Eudorylaimus	Tarsonemus	-0.166	-0.701	4.864	3.516
Thornematidae	Mylonchulus	-0.470	-0.005	4.864	4.563
Thornematidae	Tripyla	-0.470	-0.420	4.864	4.563
Thornematidae	Alliphis	-0.470	0.053	4.864	3.215
Thornematidae	Arctoseius	-0.470	-0.152	4.864	3.516
Thornematidae	Cheiroseius	-0.470	0.356	4.864	3.215
Thornematidae	Lysigamasus	-0.470	0.407	4.864	3.692
Thornematidae	Uropoda	-0.470	0.481	4.864	3.215
Thornematidae	Aporcelaimellus	-0.470	0.548	4.864	5.341
Thornematidae	Dorylaimoidea	-0.470	-0.604	4.864	5.341
Thornematidae	Eudorylaimus	-0.470	-0.166	4.864	4.864
Thornematidae	Thornematidae	-0.470	-0.470	4.864	4.864
Thornematidae	Eupodes	-0.470	0.005	4.864	4.118
Thornematidae	Nenteria	-0.470	0.254	4.864	3.516
Thornematidae	Scutacarus	-0.470	-0.608	4.864	4.215
Thornematidae	Tarsonemus	-0.470	-0.701	4.864	3.516
Eupodes	Arctoseius	0.005	-0.152	4.118	3.516
Eupodes	Cheiroseius	0.005	0.356	4.118	3.215

Eupodes	Lysigamasus	**0.005**	**0.407**	**4.118**	**3.692**
Eupodes	Uropoda	**0.005**	**0.481**	**4.118**	**3.215**
Eupodes	Aporcelaimellus	**0.005**	**0.548**	**4.118**	**5.341**
Eupodes	Dorylaimoidea	**0.005**	**-0.604**	**4.118**	**5.341**
Eupodes	Eudorylaimus	**0.005**	**-0.166**	**4.118**	**4.864**
Eupodes	Thornematidae	**0.005**	**-0.470**	**4.118**	**4.864**
Eupodes	Eupodes	**0.005**	**0.005**	**4.118**	**4.118**
Eupodes	Nenteria	**0.005**	**0.254**	**4.118**	**3.516**
Eupodes	Scutacarus	**0.005**	**-0.608**	**4.118**	**4.215**
Eupodes	Tarsonemus	**0.005**	**-0.701**	**4.118**	**3.516**
Nenteria	Arctoseius	**0.254**	**-0.152**	**3.516**	**3.516**
Nenteria	Cheiroseius	**0.254**	**0.356**	**3.516**	**3.215**
Nenteria	Lysigamasus	**0.254**	**0.407**	**3.516**	**3.692**
Nenteria	Uropoda	**0.254**	**0.481**	**3.516**	**3.215**
Nenteria	Aporcelaimellus	**0.254**	**0.548**	**3.516**	**5.341**
Nenteria	Dorylaimoidea	**0.254**	**-0.604**	**3.516**	**5.341**
Nenteria	Eudorylaimus	**0.254**	**-0.166**	**3.516**	**4.864**
Nenteria	Thornematidae	**0.254**	**-0.470**	**3.516**	**4.864**
Nenteria	Eupodes	**0.254**	**0.005**	**3.516**	**4.118**
Nenteria	Nenteria	**0.254**	**0.254**	**3.516**	**3.516**
Nenteria	Scutacarus	**0.254**	**-0.608**	**3.516**	**4.215**
Nenteria	Tarsonemus	**0.254**	**-0.701**	**3.516**	**3.516**
Scutacarus	Arctoseius	**-0.608**	**-0.152**	**4.215**	**3.516**
Scutacarus	Cheiroseius	**-0.608**	**0.356**	**4.215**	**3.215**
Scutacarus	Lysigamasus	**-0.608**	**0.407**	**4.215**	**3.692**
Scutacarus	Uropoda	**-0.608**	**0.481**	**4.215**	**3.215**
Scutacarus	Aporcelaimellus	**-0.608**	**0.548**	**4.215**	**5.341**
Scutacarus	Dorylaimoidea	**-0.608**	**-0.604**	**4.215**	**5.341**
Scutacarus	Eudorylaimus	**-0.608**	**-0.166**	**4.215**	**4.864**
Scutacarus	Thornematidae	**-0.608**	**-0.470**	**4.215**	**4.864**
Scutacarus	Eupodes	**-0.608**	**0.005**	**4.215**	**4.118**
Scutacarus	Nenteria	**-0.608**	**0.254**	**4.215**	**3.516**
Scutacarus	Scutacarus	**-0.608**	**-0.608**	**4.215**	**4.215**
Scutacarus	Tarsonemus	**-0.608**	**-0.701**	**4.215**	**3.516**
Tarsonemus	Arctoseius	**-0.701**	**-0.152**	**3.516**	**3.516**
Tarsonemus	Cheiroseius	**-0.701**	**0.356**	**3.516**	**3.215**
Tarsonemus	Lysigamasus	**-0.701**	**0.407**	**3.516**	**3.692**
Tarsonemus	Uropoda	**-0.701**	**0.481**	**3.516**	**3.215**
Tarsonemus	Aporcelaimellus	**-0.701**	**0.548**	**3.516**	**5.341**
Tarsonemus	Dorylaimoidea	**-0.701**	**-0.604**	**3.516**	**5.341**
Tarsonemus	Eudorylaimus	**-0.701**	**-0.166**	**3.516**	**4.864**
Tarsonemus	Thornematidae	**-0.701**	**-0.470**	**3.516**	**4.864**
Tarsonemus	Eupodes	**-0.701**	**0.005**	**3.516**	**4.118**
Tarsonemus	Nenteria	**-0.701**	**0.254**	**3.516**	**3.516**
Tarsonemus	Scutacarus	**-0.701**	**-0.608**	**3.516**	**4.215**
Tarsonemus	Tarsonemus	**-0.701**	**-0.701**	**3.516**	**3.516**

Resource	Consumer	Mres	Mconsumer	Nres	Nconsumer
Aglenchus	Arctoseius	**-1.053**	**-0.152**	**4.906**	**3.248**
Aglenchus	Hypoaspis	**-1.053**	**0.334**	**4.906**	**3.549**
Aglenchus	Lysigamasus	**-1.053**	**0.407**	**4.906**	**3.248**
Aglenchus	Pergamasus	**-1.053**	**1.081**	**4.906**	**3.248**
Aglenchus	Aporcelaimellus	**-1.053**	**0.548**	**4.906**	**5.030**
Aglenchus	Dorylaimoidea	**-1.053**	**-0.604**	**4.906**	**5.274**
Aglenchus	Qudsianematidae	**-1.053**	**-0.207**	**4.906**	**4.729**
Aglenchus	Eupodes	**-1.053**	**0.005**	**4.906**	**4.026**
Aglenchus	Protodinychus	**-1.053**	**0.549**	**4.906**	**3.549**
Aglenchus	Scheloribates	**-1.053**	**0.202**	**4.906**	**3.549**
Aglenchus	Scutacarus	**-1.053**	**-0.608**	**4.906**	**4.093**
Aglenchus	Tarsonemus	**-1.053**	**-0.701**	**4.906**	**3.248**
Coslenchus	Arctoseius	**-0.821**	**-0.152**	**4.729**	**3.248**
Coslenchus	Hypoaspis	**-0.821**	**0.334**	**4.729**	**3.549**
Coslenchus	Lysigamasus	**-0.821**	**0.407**	**4.729**	**3.248**
Coslenchus	Pergamasus	**-0.821**	**1.081**	**4.729**	**3.248**
Coslenchus	Aporcelaimellus	**-0.821**	**0.548**	**4.729**	**5.030**
Coslenchus	Dorylaimoidea	**-0.821**	**-0.604**	**4.729**	**5.274**
Coslenchus	Qudsianematidae	**-0.821**	**-0.207**	**4.729**	**4.729**
Coslenchus	Eupodes	**-0.821**	**0.005**	**4.729**	**4.026**
Coslenchus	Protodinychus	**-0.821**	**0.549**	**4.729**	**3.549**
Coslenchus	Scheloribates	**-0.821**	**0.202**	**4.729**	**3.549**
Coslenchus	Scutacarus	**-0.821**	**-0.608**	**4.729**	**4.093**
Coslenchus	Tarsonemus	**-0.821**	**-0.701**	**4.729**	**3.248**
Dolichodoridae	Arctoseius	**-0.885**	**-0.152**	**5.127**	**3.248**
Dolichodoridae	Hypoaspis	**-0.885**	**0.334**	**5.127**	**3.549**
Dolichodoridae	Lysigamasus	**-0.885**	**0.407**	**5.127**	**3.248**
Dolichodoridae	Pergamasus	**-0.885**	**1.081**	**5.127**	**3.248**
Dolichodoridae	Aporcelaimellus	**-0.885**	**0.548**	**5.127**	**5.030**
Dolichodoridae	Dorylaimoidea	**-0.885**	**-0.604**	**5.127**	**5.274**
Dolichodoridae	Qudsianematidae	**-0.885**	**-0.207**	**5.127**	**4.729**
Dolichodoridae	Eupodes	**-0.885**	**0.005**	**5.127**	**4.026**
Dolichodoridae	Protodinychus	**-0.885**	**0.549**	**5.127**	**3.549**
Dolichodoridae	Scheloribates	**-0.885**	**0.202**	**5.127**	**3.549**
Dolichodoridae	Scutacarus	**-0.885**	**-0.608**	**5.127**	**4.093**
Dolichodoridae	Tarsonemus	**-0.885**	**-0.701**	**5.127**	**3.248**
Helicotylenchus	Arctoseius	**-0.792**	**-0.152**	**4.906**	**3.248**
Helicotylenchus	Hypoaspis	**-0.792**	**0.334**	**4.906**	**3.549**
Helicotylenchus	Lysigamasus	**-0.792**	**0.407**	**4.906**	**3.248**
Helicotylenchus	Pergamasus	**-0.792**	**1.081**	**4.906**	**3.248**
Helicotylenchus	Aporcelaimellus	**-0.792**	**0.548**	**4.906**	**5.030**
Helicotylenchus	Dorylaimoidea	**-0.792**	**-0.604**	**4.906**	**5.274**
Helicotylenchus	Qudsianematidae	**-0.792**	**-0.207**	**4.906**	**4.729**
Helicotylenchus	Eupodes	**-0.792**	**0.005**	**4.906**	**4.026**
Helicotylenchus	Protodinychus	**-0.792**	**0.549**	**4.906**	**3.549**
Helicotylenchus	Scheloribates	**-0.792**	**0.202**	**4.906**	**3.549**
Helicotylenchus	Scutacarus	**-0.792**	**-0.608**	**4.906**	**4.093**
Helicotylenchus	Tarsonemus	**-0.792**	**-0.701**	**4.906**	**3.248**
Meloidogyne	Arctoseius	**-1.287**	**-0.152**	**4.729**	**3.248**
Meloidogyne	Hypoaspis	**-1.287**	**0.334**	**4.729**	**3.549**

Meloidogyne	Lysigamasus	**-1.287**	**0.407**	**4.729**	**3.248**
Meloidogyne	Pergamasus	**-1.287**	**1.081**	**4.729**	**3.248**
Meloidogyne	Aporcelaimellus	**-1.287**	**0.548**	**4.729**	**5.030**
Meloidogyne	Dorylaimoidea	**-1.287**	**-0.604**	**4.729**	**5.274**
Meloidogyne	Qudsianematidae	**-1.287**	**-0.207**	**4.729**	**4.729**
Meloidogyne	Eupodes	**-1.287**	**0.005**	**4.729**	**4.026**
Meloidogyne	Protodinychus	**-1.287**	**0.549**	**4.729**	**3.549**
Meloidogyne	Scheloribates	**-1.287**	**0.202**	**4.729**	**3.549**
Meloidogyne	Scutacarus	**-1.287**	**-0.608**	**4.729**	**4.093**
Meloidogyne	Tarsonemus	**-1.287**	**-0.701**	**4.729**	**3.248**
Paratylenchus	Arctoseius	**-1.244**	**-0.152**	**5.332**	**3.248**
Paratylenchus	Hypoaspis	**-1.244**	**0.334**	**5.332**	**3.549**
Paratylenchus	Lysigamasus	**-1.244**	**0.407**	**5.332**	**3.248**
Paratylenchus	Pergamasus	**-1.244**	**1.081**	**5.332**	**3.248**
Paratylenchus	Aporcelaimellus	**-1.244**	**0.548**	**5.332**	**5.030**
Paratylenchus	Dorylaimoidea	**-1.244**	**-0.604**	**5.332**	**5.274**
Paratylenchus	Qudsianematidae	**-1.244**	**-0.207**	**5.332**	**4.729**
Paratylenchus	Eupodes	**-1.244**	**0.005**	**5.332**	**4.026**
Paratylenchus	Protodinychus	**-1.244**	**0.549**	**5.332**	**3.549**
Paratylenchus	Scheloribates	**-1.244**	**0.202**	**5.332**	**3.549**
Paratylenchus	Scutacarus	**-1.244**	**-0.608**	**5.332**	**4.093**
Paratylenchus	Tarsonemus	**-1.244**	**-0.701**	**5.332**	**3.248**
Pratylenchus	Arctoseius	**-1.226**	**-0.152**	**4.729**	**3.248**
Pratylenchus	Hypoaspis	**-1.226**	**0.334**	**4.729**	**3.549**
Pratylenchus	Lysigamasus	**-1.226**	**0.407**	**4.729**	**3.248**
Pratylenchus	Pergamasus	**-1.226**	**1.081**	**4.729**	**3.248**
Pratylenchus	Aporcelaimellus	**-1.226**	**0.548**	**4.729**	**5.030**
Pratylenchus	Dorylaimoidea	**-1.226**	**-0.604**	**4.729**	**5.274**
Pratylenchus	Qudsianematidae	**-1.226**	**-0.207**	**4.729**	**4.729**
Pratylenchus	Eupodes	**-1.226**	**0.005**	**4.729**	**4.026**
Pratylenchus	Protodinychus	**-1.226**	**0.549**	**4.729**	**3.549**
Pratylenchus	Scheloribates	**-1.226**	**0.202**	**4.729**	**3.549**
Pratylenchus	Scutacarus	**-1.226**	**-0.608**	**4.729**	**4.093**
Pratylenchus	Tarsonemus	**-1.226**	**-0.701**	**4.729**	**3.248**
Tylenchorhynchus	Arctoseius	**-0.664**	**-0.152**	**5.030**	**3.248**
Tylenchorhynchus	Hypoaspis	**-0.664**	**0.334**	**5.030**	**3.549**
Tylenchorhynchus	Lysigamasus	**-0.664**	**0.407**	**5.030**	**3.248**
Tylenchorhynchus	Pergamasus	**-0.664**	**1.081**	**5.030**	**3.248**
Tylenchorhynchus	Aporcelaimellus	**-0.664**	**0.548**	**5.030**	**5.030**
Tylenchorhynchus	Dorylaimoidea	**-0.664**	**-0.604**	**5.030**	**5.274**
Tylenchorhynchus	Qudsianematidae	**-0.664**	**-0.207**	**5.030**	**4.729**
Tylenchorhynchus	Eupodes	**-0.664**	**0.005**	**5.030**	**4.026**
Tylenchorhynchus	Protodinychus	**-0.664**	**0.549**	**5.030**	**3.549**
Tylenchorhynchus	Scheloribates	**-0.664**	**0.202**	**5.030**	**3.549**
Tylenchorhynchus	Scutacarus	**-0.664**	**-0.608**	**5.030**	**4.093**
Tylenchorhynchus	Tarsonemus	**-0.664**	**-0.701**	**5.030**	**3.248**
Pachygnatidae	Arctoseius	**-0.113**	**-0.152**	**3.248**	**3.248**
Pachygnatidae	Hypoaspis	**-0.113**	**0.334**	**3.248**	**3.549**
Pachygnatidae	Lysigamasus	**-0.113**	**0.407**	**3.248**	**3.248**
Pachygnatidae	Pergamasus	**-0.113**	**1.081**	**3.248**	**3.248**
Pachygnatidae	Aporcelaimellus	**-0.113**	**0.548**	**3.248**	**5.030**

Pachygnatidae	Dorylaimoidea	**-0.113**	**-0.604**	**3.248**	**5.274**
Pachygnatidae	Qudsianematidae	**-0.113**	**-0.207**	**3.248**	**4.729**
Pachygnatidae	Eupodes	**-0.113**	**0.005**	**3.248**	**4.026**
Pachygnatidae	Protodinychus	**-0.113**	**0.549**	**3.248**	**3.549**
Pachygnatidae	Scheloribates	**-0.113**	**0.202**	**3.248**	**3.549**
Pachygnatidae	Scutacarus	**-0.113**	**-0.608**	**3.248**	**4.093**
Pachygnatidae	Tarsonemus	**-0.113**	**-0.701**	**3.248**	**3.248**
Platynothrus	Arctoseius	**0.710**	**-0.152**	**3.248**	**3.248**
Platynothrus	Hypoaspis	**0.710**	**0.334**	**3.248**	**3.549**
Platynothrus	Lysigamasus	**0.710**	**0.407**	**3.248**	**3.248**
Platynothrus	Pergamasus	**0.710**	**1.081**	**3.248**	**3.248**
Platynothrus	Aporcelaimellus	**0.710**	**0.548**	**3.248**	**5.030**
Platynothrus	Dorylaimoidea	**0.710**	**-0.604**	**3.248**	**5.274**
Platynothrus	Qudsianematidae	**0.710**	**-0.207**	**3.248**	**4.729**
Platynothrus	Eupodes	**0.710**	**0.005**	**3.248**	**4.026**
Platynothrus	Protodinychus	**0.710**	**0.549**	**3.248**	**3.549**
Platynothrus	Scheloribates	**0.710**	**0.202**	**3.248**	**3.549**
Platynothrus	Scutacarus	**0.710**	**-0.608**	**3.248**	**4.093**
Platynothrus	Tarsonemus	**0.710**	**-0.701**	**3.248**	**3.248**
Aphelenchoides	Arctoseius	**-1.496**	**-0.152**	**5.428**	**3.248**
Aphelenchoides	Hypoaspis	**-1.496**	**0.334**	**5.428**	**3.549**
Aphelenchoides	Lysigamasus	**-1.496**	**0.407**	**5.428**	**3.248**
Aphelenchoides	Pergamasus	**-1.496**	**1.081**	**5.428**	**3.248**
Aphelenchoides	Aporcelaimellus	**-1.496**	**0.548**	**5.428**	**5.030**
Aphelenchoides	Dorylaimoidea	**-1.496**	**-0.604**	**5.428**	**5.274**
Aphelenchoides	Qudsianematidae	**-1.496**	**-0.207**	**5.428**	**4.729**
Aphelenchoides	Eupodes	**-1.496**	**0.005**	**5.428**	**4.026**
Aphelenchoides	Protodinychus	**-1.496**	**0.549**	**5.428**	**3.549**
Aphelenchoides	Scheloribates	**-1.496**	**0.202**	**5.428**	**3.549**
Aphelenchoides	Scutacarus	**-1.496**	**-0.608**	**5.428**	**4.093**
Aphelenchoides	Tarsonemus	**-1.496**	**-0.701**	**5.428**	**3.248**
Tylenchidae	Arctoseius	**-1.360**	**-0.152**	**5.920**	**3.248**
Tylenchidae	Hypoaspis	**-1.360**	**0.334**	**5.920**	**3.549**
Tylenchidae	Lysigamasus	**-1.360**	**0.407**	**5.920**	**3.248**
Tylenchidae	Pergamasus	**-1.360**	**1.081**	**5.920**	**3.248**
Tylenchidae	Aporcelaimellus	**-1.360**	**0.548**	**5.920**	**5.030**
Tylenchidae	Dorylaimoidea	**-1.360**	**-0.604**	**5.920**	**5.274**
Tylenchidae	Qudsianematidae	**-1.360**	**-0.207**	**5.920**	**4.729**
Tylenchidae	Eupodes	**-1.360**	**0.005**	**5.920**	**4.026**
Tylenchidae	Protodinychus	**-1.360**	**0.549**	**5.920**	**3.549**
Tylenchidae	Scheloribates	**-1.360**	**0.202**	**5.920**	**3.549**
Tylenchidae	Scutacarus	**-1.360**	**-0.608**	**5.920**	**4.093**
Tylenchidae	Tarsonemus	**-1.360**	**-0.701**	**5.920**	**3.248**
Medioppia	Arctoseius	**-0.235**	**-0.152**	**3.850**	**3.248**
Medioppia	Hypoaspis	**-0.235**	**0.334**	**3.850**	**3.549**
Medioppia	Lysigamasus	**-0.235**	**0.407**	**3.850**	**3.248**
Medioppia	Pergamasus	**-0.235**	**1.081**	**3.850**	**3.248**
Medioppia	Aporcelaimellus	**-0.235**	**0.548**	**3.850**	**5.030**
Medioppia	Dorylaimoidea	**-0.235**	**-0.604**	**3.850**	**5.274**
Medioppia	Qudsianematidae	**-0.235**	**-0.207**	**3.850**	**4.729**
Medioppia	Eupodes	**-0.235**	**0.005**	**3.850**	**4.026**

Medioppia	Protodinychus	**-0.235**	**0.549**	**3.850**	**3.549**
Medioppia	Scheloribates	**-0.235**	**0.202**	**3.850**	**3.549**
Medioppia	Scutacarus	**-0.235**	**-0.608**	**3.850**	**4.093**
Medioppia	Tarsonemus	**-0.235**	**-0.701**	**3.850**	**3.248**
Microppia	Arctoseius	**-0.544**	**-0.152**	**4.549**	**3.248**
Microppia	Hypoaspis	**-0.544**	**0.334**	**4.549**	**3.549**
Microppia	Lysigamasus	**-0.544**	**0.407**	**4.549**	**3.248**
Microppia	Pergamasus	**-0.544**	**1.081**	**4.549**	**3.248**
Microppia	Aporcelaimellus	**-0.544**	**0.548**	**4.549**	**5.030**
Microppia	Dorylaimoidea	**-0.544**	**-0.604**	**4.549**	**5.274**
Microppia	Qudsianematidae	**-0.544**	**-0.207**	**4.549**	**4.729**
Microppia	Eupodes	**-0.544**	**0.005**	**4.549**	**4.026**
Microppia	Protodinychus	**-0.544**	**0.549**	**4.549**	**3.549**
Microppia	Scheloribates	**-0.544**	**0.202**	**4.549**	**3.549**
Microppia	Scutacarus	**-0.544**	**-0.608**	**4.549**	**4.093**
Microppia	Tarsonemus	**-0.544**	**-0.701**	**4.549**	**3.248**
Minunthozetes	Arctoseius	**-0.249**	**-0.152**	**3.248**	**3.248**
Minunthozetes	Hypoaspis	**-0.249**	**0.334**	**3.248**	**3.549**
Minunthozetes	Lysigamasus	**-0.249**	**0.407**	**3.248**	**3.248**
Minunthozetes	Pergamasus	**-0.249**	**1.081**	**3.248**	**3.248**
Minunthozetes	Aporcelaimellus	**-0.249**	**0.548**	**3.248**	**5.030**
Minunthozetes	Dorylaimoidea	**-0.249**	**-0.604**	**3.248**	**5.274**
Minunthozetes	Qudsianematidae	**-0.249**	**-0.207**	**3.248**	**4.729**
Minunthozetes	Eupodes	**-0.249**	**0.005**	**3.248**	**4.026**
Minunthozetes	Protodinychus	**-0.249**	**0.549**	**3.248**	**3.549**
Minunthozetes	Scheloribates	**-0.249**	**0.202**	**3.248**	**3.549**
Minunthozetes	Scutacarus	**-0.249**	**-0.608**	**3.248**	**4.093**
Minunthozetes	Tarsonemus	**-0.249**	**-0.701**	**3.248**	**3.248**
Oppiella	Arctoseius	**-0.447**	**-0.152**	**3.947**	**3.248**
Oppiella	Hypoaspis	**-0.447**	**0.334**	**3.947**	**3.549**
Oppiella	Lysigamasus	**-0.447**	**0.407**	**3.947**	**3.248**
Oppiella	Pergamasus	**-0.447**	**1.081**	**3.947**	**3.248**
Oppiella	Aporcelaimellus	**-0.447**	**0.548**	**3.947**	**5.030**
Oppiella	Dorylaimoidea	**-0.447**	**-0.604**	**3.947**	**5.274**
Oppiella	Qudsianematidae	**-0.447**	**-0.207**	**3.947**	**4.729**
Oppiella	Eupodes	**-0.447**	**0.005**	**3.947**	**4.026**
Oppiella	Protodinychus	**-0.447**	**0.549**	**3.947**	**3.549**
Oppiella	Scheloribates	**-0.447**	**0.202**	**3.947**	**3.549**
Oppiella	Scutacarus	**-0.447**	**-0.608**	**3.947**	**4.093**
Oppiella	Tarsonemus	**-0.447**	**-0.701**	**3.947**	**3.248**
Punctoribates	Arctoseius	**-0.029**	**-0.152**	**3.549**	**3.248**
Punctoribates	Hypoaspis	**-0.029**	**0.334**	**3.549**	**3.549**
Punctoribates	Lysigamasus	**-0.029**	**0.407**	**3.549**	**3.248**
Punctoribates	Pergamasus	**-0.029**	**1.081**	**3.549**	**3.248**
Punctoribates	Aporcelaimellus	**-0.029**	**0.548**	**3.549**	**5.030**
Punctoribates	Dorylaimoidea	**-0.029**	**-0.604**	**3.549**	**5.274**
Punctoribates	Qudsianematidae	**-0.029**	**-0.207**	**3.549**	**4.729**
Punctoribates	Eupodes	**-0.029**	**0.005**	**3.549**	**4.026**
Punctoribates	Protodinychus	**-0.029**	**0.549**	**3.549**	**3.549**
Punctoribates	Scheloribates	**-0.029**	**0.202**	**3.549**	**3.549**
Punctoribates	Scutacarus	**-0.029**	**-0.608**	**3.549**	**4.093**

Punctoribates	Tarsonemus	**-0.029**	**-0.701**	**3.549**	**3.248**
Pygmephorus	Arctoseius	**-0.376**	**-0.152**	**3.549**	**3.248**
Pygmephorus	Hypoaspis	**-0.376**	**0.334**	**3.549**	**3.549**
Pygmephorus	Lysigamasus	**-0.376**	**0.407**	**3.549**	**3.248**
Pygmephorus	Pergamasus	**-0.376**	**1.081**	**3.549**	**3.248**
Pygmephorus	Aporcelaimellus	**-0.376**	**0.548**	**3.549**	**5.030**
Pygmephorus	Dorylaimoidea	**-0.376**	**-0.604**	**3.549**	**5.274**
Pygmephorus	Qudsianematidae	**-0.376**	**-0.207**	**3.549**	**4.729**
Pygmephorus	Eupodes	**-0.376**	**0.005**	**3.549**	**4.026**
Pygmephorus	Protodinychus	**-0.376**	**0.549**	**3.549**	**3.549**
Pygmephorus	Scheloribates	**-0.376**	**0.202**	**3.549**	**3.549**
Pygmephorus	Scutacarus	**-0.376**	**-0.608**	**3.549**	**4.093**
Pygmephorus	Tarsonemus	**-0.376**	**-0.701**	**3.549**	**3.248**
Tectocepheus	Arctoseius	**-0.220**	**-0.152**	**3.947**	**3.248**
Tectocepheus	Hypoaspis	**-0.220**	**0.334**	**3.947**	**3.549**
Tectocepheus	Lysigamasus	**-0.220**	**0.407**	**3.947**	**3.248**
Tectocepheus	Pergamasus	**-0.220**	**1.081**	**3.947**	**3.248**
Tectocepheus	Aporcelaimellus	**-0.220**	**0.548**	**3.947**	**5.030**
Tectocepheus	Dorylaimoidea	**-0.220**	**-0.604**	**3.947**	**5.274**
Tectocepheus	Qudsianematidae	**-0.220**	**-0.207**	**3.947**	**4.729**
Tectocepheus	Eupodes	**-0.220**	**0.005**	**3.947**	**4.026**
Tectocepheus	Protodinychus	**-0.220**	**0.549**	**3.947**	**3.549**
Tectocepheus	Scheloribates	**-0.220**	**0.202**	**3.947**	**3.549**
Tectocepheus	Scutacarus	**-0.220**	**-0.608**	**3.947**	**4.093**
Tectocepheus	Tarsonemus	**-0.220**	**-0.701**	**3.947**	**3.248**
Isotoma	Arctoseius	**1.898**	**-0.152**	**3.549**	**3.248**
Isotoma	Hypoaspis	**1.898**	**0.334**	**3.549**	**3.549**
Isotoma	Lysigamasus	**1.898**	**0.407**	**3.549**	**3.248**
Isotoma	Pergamasus	**1.898**	**1.081**	**3.549**	**3.248**
Isotoma	Aporcelaimellus	**1.898**	**0.548**	**3.549**	**5.030**
Isotoma	Dorylaimoidea	**1.898**	**-0.604**	**3.549**	**5.274**
Isotoma	Qudsianematidae	**1.898**	**-0.207**	**3.549**	**4.729**
Isotoma	Eupodes	**1.898**	**0.005**	**3.549**	**4.026**
Isotoma	Protodinychus	**1.898**	**0.549**	**3.549**	**3.549**
Isotoma	Scheloribates	**1.898**	**0.202**	**3.549**	**3.549**
Isotoma	Scutacarus	**1.898**	**-0.608**	**3.549**	**4.093**
Isotoma	Tarsonemus	**1.898**	**-0.701**	**3.549**	**3.248**
Lepidocyrtus	Arctoseius	**1.231**	**-0.152**	**3.850**	**3.248**
Lepidocyrtus	Hypoaspis	**1.231**	**0.334**	**3.850**	**3.549**
Lepidocyrtus	Lysigamasus	**1.231**	**0.407**	**3.850**	**3.248**
Lepidocyrtus	Pergamasus	**1.231**	**1.081**	**3.850**	**3.248**
Lepidocyrtus	Aporcelaimellus	**1.231**	**0.548**	**3.850**	**5.030**
Lepidocyrtus	Dorylaimoidea	**1.231**	**-0.604**	**3.850**	**5.274**
Lepidocyrtus	Qudsianematidae	**1.231**	**-0.207**	**3.850**	**4.729**
Lepidocyrtus	Eupodes	**1.231**	**0.005**	**3.850**	**4.026**
Lepidocyrtus	Protodinychus	**1.231**	**0.549**	**3.850**	**3.549**
Lepidocyrtus	Scheloribates	**1.231**	**0.202**	**3.850**	**3.549**
Lepidocyrtus	Scutacarus	**1.231**	**-0.608**	**3.850**	**4.093**
Lepidocyrtus	Tarsonemus	**1.231**	**-0.701**	**3.850**	**3.248**
Proisotoma	Arctoseius	**0.770**	**-0.152**	**3.248**	**3.248**
Proisotoma	Hypoaspis	**0.770**	**0.334**	**3.248**	**3.549**

Proisotoma	Lysigamasus	**0.770**	**0.407**	**3.248**	**3.248**
Proisotoma	Pergamasus	**0.770**	**1.081**	**3.248**	**3.248**
Proisotoma	Aporcelaimellus	**0.770**	**0.548**	**3.248**	**5.030**
Proisotoma	Dorylaimoidea	**0.770**	**-0.604**	**3.248**	**5.274**
Proisotoma	Qudsianematidae	**0.770**	**-0.207**	**3.248**	**4.729**
Proisotoma	Eupodes	**0.770**	**0.005**	**3.248**	**4.026**
Proisotoma	Protodinychus	**0.770**	**0.549**	**3.248**	**3.549**
Proisotoma	Scheloribates	**0.770**	**0.202**	**3.248**	**3.549**
Proisotoma	Scutacarus	**0.770**	**-0.608**	**3.248**	**4.093**
Proisotoma	Tarsonemus	**0.770**	**-0.701**	**3.248**	**3.248**
Achaeta	Arctoseius	**-0.072**	**-0.152**	**2.373**	**3.248**
Achaeta	Hypoaspis	**-0.072**	**0.334**	**2.373**	**3.549**
Achaeta	Lysigamasus	**-0.072**	**0.407**	**2.373**	**3.248**
Achaeta	Pergamasus	**-0.072**	**1.081**	**2.373**	**3.248**
Achaeta	Aporcelaimellus	**-0.072**	**0.548**	**2.373**	**5.030**
Achaeta	Dorylaimoidea	**-0.072**	**-0.604**	**2.373**	**5.274**
Achaeta	Qudsianematidae	**-0.072**	**-0.207**	**2.373**	**4.729**
Achaeta	Eupodes	**-0.072**	**0.005**	**2.373**	**4.026**
Achaeta	Protodinychus	**-0.072**	**0.549**	**2.373**	**3.549**
Achaeta	Scheloribates	**-0.072**	**0.202**	**2.373**	**3.549**
Achaeta	Scutacarus	**-0.072**	**-0.608**	**2.373**	**4.093**
Achaeta	Tarsonemus	**-0.072**	**-0.701**	**2.373**	**3.248**
Cognettia	Arctoseius	**1.311**	**-0.152**	**3.609**	**3.248**
Cognettia	Hypoaspis	**1.311**	**0.334**	**3.609**	**3.549**
Cognettia	Lysigamasus	**1.311**	**0.407**	**3.609**	**3.248**
Cognettia	Pergamasus	**1.311**	**1.081**	**3.609**	**3.248**
Cognettia	Aporcelaimellus	**1.311**	**0.548**	**3.609**	**5.030**
Cognettia	Dorylaimoidea	**1.311**	**-0.604**	**3.609**	**5.274**
Cognettia	Qudsianematidae	**1.311**	**-0.207**	**3.609**	**4.729**
Cognettia	Eupodes	**1.311**	**0.005**	**3.609**	**4.026**
Cognettia	Protodinychus	**1.311**	**0.549**	**3.609**	**3.549**
Cognettia	Scheloribates	**1.311**	**0.202**	**3.609**	**3.549**
Cognettia	Scutacarus	**1.311**	**-0.608**	**3.609**	**4.093**
Cognettia	Tarsonemus	**1.311**	**-0.701**	**3.609**	**3.248**
Fridericia	Arctoseius	**2.396**	**-0.152**	**2.947**	**3.248**
Fridericia	Hypoaspis	**2.396**	**0.334**	**2.947**	**3.549**
Fridericia	Lysigamasus	**2.396**	**0.407**	**2.947**	**3.248**
Fridericia	Pergamasus	**2.396**	**1.081**	**2.947**	**3.248**
Fridericia	Aporcelaimellus	**2.396**	**0.548**	**2.947**	**5.030**
Fridericia	Dorylaimoidea	**2.396**	**-0.604**	**2.947**	**5.274**
Fridericia	Qudsianematidae	**2.396**	**-0.207**	**2.947**	**4.729**
Fridericia	Eupodes	**2.396**	**0.005**	**2.947**	**4.026**
Fridericia	Protodinychus	**2.396**	**0.549**	**2.947**	**3.549**
Fridericia	Scheloribates	**2.396**	**0.202**	**2.947**	**3.549**
Fridericia	Scutacarus	**2.396**	**-0.608**	**2.947**	**4.093**
Fridericia	Tarsonemus	**2.396**	**-0.701**	**2.947**	**3.248**
Acrobeloides	Arctoseius	**-1.171**	**-0.152**	**5.575**	**3.248**
Acrobeloides	Hypoaspis	**-1.171**	**0.334**	**5.575**	**3.549**
Acrobeloides	Lysigamasus	**-1.171**	**0.407**	**5.575**	**3.248**
Acrobeloides	Pergamasus	**-1.171**	**1.081**	**5.575**	**3.248**
Acrobeloides	Aporcelaimellus	**-1.171**	**0.548**	**5.575**	**5.030**

Acrobeloides	Dorylaimoidea	**-1.171**	**-0.604**	**5.575**	**5.274**
Acrobeloides	Qudsianematidae	**-1.171**	**-0.207**	**5.575**	**4.729**
Acrobeloides	Eupodes	**-1.171**	**0.005**	**5.575**	**4.026**
Acrobeloides	Protodinychus	**-1.171**	**0.549**	**5.575**	**3.549**
Acrobeloides	Scheloribates	**-1.171**	**0.202**	**5.575**	**3.549**
Acrobeloides	Scutacarus	**-1.171**	**-0.608**	**5.575**	**4.093**
Acrobeloides	Tarsonemus	**-1.171**	**-0.701**	**5.575**	**3.248**
Anaplectus	Arctoseius	**-0.519**	**-0.152**	**4.729**	**3.248**
Anaplectus	Hypoaspis	**-0.519**	**0.334**	**4.729**	**3.549**
Anaplectus	Lysigamasus	**-0.519**	**0.407**	**4.729**	**3.248**
Anaplectus	Pergamasus	**-0.519**	**1.081**	**4.729**	**3.248**
Anaplectus	Aporcelaimellus	**-0.519**	**0.548**	**4.729**	**5.030**
Anaplectus	Dorylaimoidea	**-0.519**	**-0.604**	**4.729**	**5.274**
Anaplectus	Qudsianematidae	**-0.519**	**-0.207**	**4.729**	**4.729**
Anaplectus	Eupodes	**-0.519**	**0.005**	**4.729**	**4.026**
Anaplectus	Protodinychus	**-0.519**	**0.549**	**4.729**	**3.549**
Anaplectus	Scheloribates	**-0.519**	**0.202**	**4.729**	**3.549**
Anaplectus	Scutacarus	**-0.519**	**-0.608**	**4.729**	**4.093**
Anaplectus	Tarsonemus	**-0.519**	**-0.701**	**4.729**	**3.248**
Cephalobidae	Arctoseius	**-1.055**	**-0.152**	**5.030**	**3.248**
Cephalobidae	Hypoaspis	**-1.055**	**0.334**	**5.030**	**3.549**
Cephalobidae	Lysigamasus	**-1.055**	**0.407**	**5.030**	**3.248**
Cephalobidae	Pergamasus	**-1.055**	**1.081**	**5.030**	**3.248**
Cephalobidae	Aporcelaimellus	**-1.055**	**0.548**	**5.030**	**5.030**
Cephalobidae	Dorylaimoidea	**-1.055**	**-0.604**	**5.030**	**5.274**
Cephalobidae	Qudsianematidae	**-1.055**	**-0.207**	**5.030**	**4.729**
Cephalobidae	Eupodes	**-1.055**	**0.005**	**5.030**	**4.026**
Cephalobidae	Protodinychus	**-1.055**	**0.549**	**5.030**	**3.549**
Cephalobidae	Scheloribates	**-1.055**	**0.202**	**5.030**	**3.549**
Cephalobidae	Scutacarus	**-1.055**	**-0.608**	**5.030**	**4.093**
Cephalobidae	Tarsonemus	**-1.055**	**-0.701**	**5.030**	**3.248**
Eucephalobus	Arctoseius	**-0.855**	**-0.152**	**5.542**	**3.248**
Eucephalobus	Hypoaspis	**-0.855**	**0.334**	**5.542**	**3.549**
Eucephalobus	Lysigamasus	**-0.855**	**0.407**	**5.542**	**3.248**
Eucephalobus	Pergamasus	**-0.855**	**1.081**	**5.542**	**3.248**
Eucephalobus	Aporcelaimellus	**-0.855**	**0.548**	**5.542**	**5.030**
Eucephalobus	Dorylaimoidea	**-0.855**	**-0.604**	**5.542**	**5.274**
Eucephalobus	Qudsianematidae	**-0.855**	**-0.207**	**5.542**	**4.729**
Eucephalobus	Eupodes	**-0.855**	**0.005**	**5.542**	**4.026**
Eucephalobus	Protodinychus	**-0.855**	**0.549**	**5.542**	**3.549**
Eucephalobus	Scheloribates	**-0.855**	**0.202**	**5.542**	**3.549**
Eucephalobus	Scutacarus	**-0.855**	**-0.608**	**5.542**	**4.093**
Eucephalobus	Tarsonemus	**-0.855**	**-0.701**	**5.542**	**3.248**
Metateratocephalus	Arctoseius	**-1.506**	**-0.152**	**4.428**	**3.248**
Metateratocephalus	Hypoaspis	**-1.506**	**0.334**	**4.428**	**3.549**
Metateratocephalus	Lysigamasus	**-1.506**	**0.407**	**4.428**	**3.248**
Metateratocephalus	Pergamasus	**-1.506**	**1.081**	**4.428**	**3.248**
Metateratocephalus	Aporcelaimellus	**-1.506**	**0.548**	**4.428**	**5.030**
Metateratocephalus	Dorylaimoidea	**-1.506**	**-0.604**	**4.428**	**5.274**
Metateratocephalus	Qudsianematidae	**-1.506**	**-0.207**	**4.428**	**4.729**
Metateratocephalus	Eupodes	**-1.506**	**0.005**	**4.428**	**4.026**

Metateratocephalus	Protodinychus	**-1.506**	**0.549**	**4.428**	**3.549**
Metateratocephalus	Scheloribates	**-1.506**	**0.202**	**4.428**	**3.549**
Metateratocephalus	Scutacarus	**-1.506**	**-0.608**	**4.428**	**4.093**
Metateratocephalus	Tarsonemus	**-1.506**	**-0.701**	**4.428**	**3.248**
Panagrolaimus	Arctoseius	**-0.945**	**-0.152**	**4.906**	**3.248**
Panagrolaimus	Hypoaspis	**-0.945**	**0.334**	**4.906**	**3.549**
Panagrolaimus	Lysigamasus	**-0.945**	**0.407**	**4.906**	**3.248**
Panagrolaimus	Pergamasus	**-0.945**	**1.081**	**4.906**	**3.248**
Panagrolaimus	Aporcelaimellus	**-0.945**	**0.548**	**4.906**	**5.030**
Panagrolaimus	Dorylaimoidea	**-0.945**	**-0.604**	**4.906**	**5.274**
Panagrolaimus	Qudsianematidae	**-0.945**	**-0.207**	**4.906**	**4.729**
Panagrolaimus	Eupodes	**-0.945**	**0.005**	**4.906**	**4.026**
Panagrolaimus	Protodinychus	**-0.945**	**0.549**	**4.906**	**3.549**
Panagrolaimus	Scheloribates	**-0.945**	**0.202**	**4.906**	**3.549**
Panagrolaimus	Scutacarus	**-0.945**	**-0.608**	**4.906**	**4.093**
Panagrolaimus	Tarsonemus	**-0.945**	**-0.701**	**4.906**	**3.248**
Plectus	Arctoseius	**-0.583**	**-0.152**	**5.542**	**3.248**
Plectus	Hypoaspis	**-0.583**	**0.334**	**5.542**	**3.549**
Plectus	Lysigamasus	**-0.583**	**0.407**	**5.542**	**3.248**
Plectus	Pergamasus	**-0.583**	**1.081**	**5.542**	**3.248**
Plectus	Aporcelaimellus	**-0.583**	**0.548**	**5.542**	**5.030**
Plectus	Dorylaimoidea	**-0.583**	**-0.604**	**5.542**	**5.274**
Plectus	Qudsianematidae	**-0.583**	**-0.207**	**5.542**	**4.729**
Plectus	Eupodes	**-0.583**	**0.005**	**5.542**	**4.026**
Plectus	Protodinychus	**-0.583**	**0.549**	**5.542**	**3.549**
Plectus	Scheloribates	**-0.583**	**0.202**	**5.542**	**3.549**
Plectus	Scutacarus	**-0.583**	**-0.608**	**5.542**	**4.093**
Plectus	Tarsonemus	**-0.583**	**-0.701**	**5.542**	**3.248**
Prismatolaimus	Arctoseius	**-1.280**	**-0.152**	**5.332**	**3.248**
Prismatolaimus	Hypoaspis	**-1.280**	**0.334**	**5.332**	**3.549**
Prismatolaimus	Lysigamasus	**-1.280**	**0.407**	**5.332**	**3.248**
Prismatolaimus	Pergamasus	**-1.280**	**1.081**	**5.332**	**3.248**
Prismatolaimus	Aporcelaimellus	**-1.280**	**0.548**	**5.332**	**5.030**
Prismatolaimus	Dorylaimoidea	**-1.280**	**-0.604**	**5.332**	**5.274**
Prismatolaimus	Qudsianematidae	**-1.280**	**-0.207**	**5.332**	**4.729**
Prismatolaimus	Eupodes	**-1.280**	**0.005**	**5.332**	**4.026**
Prismatolaimus	Protodinychus	**-1.280**	**0.549**	**5.332**	**3.549**
Prismatolaimus	Scheloribates	**-1.280**	**0.202**	**5.332**	**3.549**
Prismatolaimus	Scutacarus	**-1.280**	**-0.608**	**5.332**	**4.093**
Prismatolaimus	Tarsonemus	**-1.280**	**-0.701**	**5.332**	**3.248**
Rhabditidae	Arctoseius	**-0.692**	**-0.152**	**4.428**	**3.248**
Rhabditidae	Hypoaspis	**-0.692**	**0.334**	**4.428**	**3.549**
Rhabditidae	Lysigamasus	**-0.692**	**0.407**	**4.428**	**3.248**
Rhabditidae	Pergamasus	**-0.692**	**1.081**	**4.428**	**3.248**
Rhabditidae	Aporcelaimellus	**-0.692**	**0.548**	**4.428**	**5.030**
Rhabditidae	Dorylaimoidea	**-0.692**	**-0.604**	**4.428**	**5.274**
Rhabditidae	Qudsianematidae	**-0.692**	**-0.207**	**4.428**	**4.729**
Rhabditidae	Eupodes	**-0.692**	**0.005**	**4.428**	**4.026**
Rhabditidae	Protodinychus	**-0.692**	**0.549**	**4.428**	**3.549**
Rhabditidae	Scheloribates	**-0.692**	**0.202**	**4.428**	**3.549**
Rhabditidae	Scutacarus	**-0.692**	**-0.608**	**4.428**	**4.093**

Rhabditidae	Tarsonemus	**-0.692**	**-0.701**	**4.428**	**3.248**
Teratocephalus	Arctoseius	**-1.630**	**-0.152**	**5.332**	**3.248**
Teratocephalus	Hypoaspis	**-1.630**	**0.334**	**5.332**	**3.549**
Teratocephalus	Lysigamasus	**-1.630**	**0.407**	**5.332**	**3.248**
Teratocephalus	Pergamasus	**-1.630**	**1.081**	**5.332**	**3.248**
Teratocephalus	Aporcelaimellus	**-1.630**	**0.548**	**5.332**	**5.030**
Teratocephalus	Dorylaimoidea	**-1.630**	**-0.604**	**5.332**	**5.274**
Teratocephalus	Qudsianematidae	**-1.630**	**-0.207**	**5.332**	**4.729**
Teratocephalus	Eupodes	**-1.630**	**0.005**	**5.332**	**4.026**
Teratocephalus	Protodinychus	**-1.630**	**0.549**	**5.332**	**3.549**
Teratocephalus	Scheloribates	**-1.630**	**0.202**	**5.332**	**3.549**
Teratocephalus	Scutacarus	**-1.630**	**-0.608**	**5.332**	**4.093**
Teratocephalus	Tarsonemus	**-1.630**	**-0.701**	**5.332**	**3.248**
Eubacteria	Acrobeloides	**-6.656**	**-1.171**	**12.562**	**5.575**
Eubacteria	Anaplectus	**-6.656**	**-0.519**	**12.562**	**4.729**
Eubacteria	Cephalobidae	**-6.656**	**-1.055**	**12.562**	**5.030**
Eubacteria	Eucephalobus	**-6.656**	**-0.855**	**12.562**	**5.542**
Eubacteria	Metateratocephalus	**-6.656**	**-1.506**	**12.562**	**4.428**
Eubacteria	Panagrolaimus	**-6.656**	**-0.945**	**12.562**	**4.906**
Eubacteria	Plectus	**-6.656**	**-0.583**	**12.562**	**5.542**
Eubacteria	Prismatolaimus	**-6.656**	**-1.280**	**12.562**	**5.332**
Eubacteria	Rhabditidae	**-6.656**	**-0.692**	**12.562**	**4.428**
Eubacteria	Teratocephalus	**-6.656**	**-1.630**	**12.562**	**5.332**
Eubacteria	Henlea	**-6.656**	**1.945**	**12.562**	**2.975**
Eubacteria	Marionina	**-6.656**	**1.308**	**12.562**	**2.072**
Henlea	Arctoseius	**1.945**	**-0.152**	**2.975**	**3.248**
Henlea	Hypoaspis	**1.945**	**0.334**	**2.975**	**3.549**
Henlea	Lysigamasus	**1.945**	**0.407**	**2.975**	**3.248**
Henlea	Pergamasus	**1.945**	**1.081**	**2.975**	**3.248**
Henlea	Aporcelaimellus	**1.945**	**0.548**	**2.975**	**5.030**
Henlea	Dorylaimoidea	**1.945**	**-0.604**	**2.975**	**5.274**
Henlea	Qudsianematidae	**1.945**	**-0.207**	**2.975**	**4.729**
Henlea	Eupodes	**1.945**	**0.005**	**2.975**	**4.026**
Henlea	Protodinychus	**1.945**	**0.549**	**2.975**	**3.549**
Henlea	Scheloribates	**1.945**	**0.202**	**2.975**	**3.549**
Henlea	Scutacarus	**1.945**	**-0.608**	**2.975**	**4.093**
Henlea	Tarsonemus	**1.945**	**-0.701**	**2.975**	**3.248**
Marionina	Arctoseius	**1.308**	**-0.152**	**2.072**	**3.248**
Marionina	Hypoaspis	**1.308**	**0.334**	**2.072**	**3.549**
Marionina	Lysigamasus	**1.308**	**0.407**	**2.072**	**3.248**
Marionina	Pergamasus	**1.308**	**1.081**	**2.072**	**3.248**
Marionina	Aporcelaimellus	**1.308**	**0.548**	**2.072**	**5.030**
Marionina	Dorylaimoidea	**1.308**	**-0.604**	**2.072**	**5.274**
Marionina	Qudsianematidae	**1.308**	**-0.207**	**2.072**	**4.729**
Marionina	Eupodes	**1.308**	**0.005**	**2.072**	**4.026**
Marionina	Protodinychus	**1.308**	**0.549**	**2.072**	**3.549**
Marionina	Scheloribates	**1.308**	**0.202**	**2.072**	**3.549**
Marionina	Scutacarus	**1.308**	**-0.608**	**2.072**	**4.093**
Marionina	Tarsonemus	**1.308**	**-0.701**	**2.072**	**3.248**
Hyphae and hair roots	Aglenchus	**5.839**	**-1.053**	**0.000**	**4.906**
Hyphae and hair roots	Coslenchus	**5.839**	**-0.821**	**0.000**	**4.729**

Hyphae and hair roots	Dolichodoridae	**5.839**	**-0.885**	**0.000**	**5.127**
Hyphae and hair roots	Helicotylenchus	**5.839**	**-0.792**	**0.000**	**4.906**
Hyphae and hair roots	Meloidogyne	**5.839**	**-1.287**	**0.000**	**4.729**
Hyphae and hair roots	Paratylenchus	**5.839**	**-1.244**	**0.000**	**5.332**
Hyphae and hair roots	Pratylenchus	**5.839**	**-1.226**	**0.000**	**4.729**
Hyphae and hair roots	Tylenchorhynchus	**5.839**	**-0.664**	**0.000**	**5.030**
Hyphae and hair roots	Pachygnatidae	**5.839**	**-0.113**	**0.000**	**3.248**
Hyphae and hair roots	Platynothrus	**5.839**	**0.710**	**0.000**	**3.248**
Hyphae and hair roots	Aphelenchoides	**5.839**	**-1.496**	**0.000**	**5.428**
Hyphae and hair roots	Tylenchidae	**5.839**	**-1.360**	**0.000**	**5.920**
Hyphae and hair roots	Medioppia	**5.839**	**-0.235**	**0.000**	**3.850**
Hyphae and hair roots	Microppia	**5.839**	**-0.544**	**0.000**	**4.549**
Hyphae and hair roots	Minunthozetes	**5.839**	**-0.249**	**0.000**	**3.248**
Hyphae and hair roots	Oppiella	**5.839**	**-0.447**	**0.000**	**3.947**
Hyphae and hair roots	Punctoribates	**5.839**	**-0.029**	**0.000**	**3.549**
Hyphae and hair roots	Pygmephorus	**5.839**	**-0.376**	**0.000**	**3.549**
Hyphae and hair roots	Tectocepheus	**5.839**	**-0.220**	**0.000**	**3.947**
Hyphae and hair roots	Isotoma	**5.839**	**1.898**	**0.000**	**3.549**
Hyphae and hair roots	Lepidocyrtus	**5.839**	**1.231**	**0.000**	**3.850**
Hyphae and hair roots	Proisotoma	**5.839**	**0.770**	**0.000**	**3.248**
Hyphae and hair roots	Achaeta	**5.839**	**-0.072**	**0.000**	**2.373**
Hyphae and hair roots	Cognettia	**5.839**	**1.311**	**0.000**	**3.609**
Hyphae and hair roots	Fridericia	**5.839**	**2.396**	**0.000**	**2.947**
Hyphae and hair roots	Aporcelaimellus	**5.839**	**0.548**	**0.000**	**5.030**
Hyphae and hair roots	Dorylaimoidea	**5.839**	**-0.604**	**0.000**	**5.274**
Hyphae and hair roots	Qudsianematidae	**5.839**	**-0.207**	**0.000**	**4.729**
Hyphae and hair roots	Eupodes	**5.839**	**0.005**	**0.000**	**4.026**
Hyphae and hair roots	Protodinychus	**5.839**	**0.549**	**0.000**	**3.549**
Hyphae and hair roots	Scheloribates	**5.839**	**0.202**	**0.000**	**3.549**
Hyphae and hair roots	Scutacarus	**5.839**	**-0.608**	**0.000**	**4.093**
Hyphae and hair roots	Tarsonemus	**5.839**	**-0.701**	**0.000**	**3.248**
Arctoseius	Aporcelaimellus	**-0.152**	**0.548**	**3.248**	**5.030**
Arctoseius	Dorylaimoidea	**-0.152**	**-0.604**	**3.248**	**5.274**
Arctoseius	Qudsianematidae	**-0.152**	**-0.207**	**3.248**	**4.729**
Arctoseius	Eupodes	**-0.152**	**0.005**	**3.248**	**4.026**
Arctoseius	Protodinychus	**-0.152**	**0.549**	**3.248**	**3.549**
Arctoseius	Scheloribates	**-0.152**	**0.202**	**3.248**	**3.549**
Arctoseius	Scutacarus	**-0.152**	**-0.608**	**3.248**	**4.093**
Arctoseius	Tarsonemus	**-0.152**	**-0.701**	**3.248**	**3.248**
Hypoaspis	Aporcelaimellus	**0.334**	**0.548**	**3.549**	**5.030**
Hypoaspis	Dorylaimoidea	**0.334**	**-0.604**	**3.549**	**5.274**
Hypoaspis	Qudsianematidae	**0.334**	**-0.207**	**3.549**	**4.729**
Hypoaspis	Eupodes	**0.334**	**0.005**	**3.549**	**4.026**
Hypoaspis	Protodinychus	**0.334**	**0.549**	**3.549**	**3.549**
Hypoaspis	Scheloribates	**0.334**	**0.202**	**3.549**	**3.549**
Hypoaspis	Scutacarus	**0.334**	**-0.608**	**3.549**	**4.093**
Hypoaspis	Tarsonemus	**0.334**	**-0.701**	**3.549**	**3.248**
Lysigamasus	Aporcelaimellus	**0.407**	**0.548**	**3.248**	**5.030**
Lysigamasus	Dorylaimoidea	**0.407**	**-0.604**	**3.248**	**5.274**
Lysigamasus	Qudsianematidae	**0.407**	**-0.207**	**3.248**	**4.729**
Lysigamasus	Eupodes	**0.407**	**0.005**	**3.248**	**4.026**

Lysigamasus	Protodinychus	**0.407**	**0.549**	**3.248**	**3.549**
Lysigamasus	Scheloribates	**0.407**	**0.202**	**3.248**	**3.549**
Lysigamasus	Scutacarus	**0.407**	**-0.608**	**3.248**	**4.093**
Lysigamasus	Tarsonemus	**0.407**	**-0.701**	**3.248**	**3.248**
Pergamasus	Aporcelaimellus	**1.081**	**0.548**	**3.248**	**5.030**
Pergamasus	Dorylaimoidea	**1.081**	**-0.604**	**3.248**	**5.274**
Pergamasus	Qudsianematidae	**1.081**	**-0.207**	**3.248**	**4.729**
Pergamasus	Eupodes	**1.081**	**0.005**	**3.248**	**4.026**
Pergamasus	Protodinychus	**1.081**	**0.549**	**3.248**	**3.549**
Pergamasus	Scheloribates	**1.081**	**0.202**	**3.248**	**3.549**
Pergamasus	Scutacarus	**1.081**	**-0.608**	**3.248**	**4.093**
Pergamasus	Tarsonemus	**1.081**	**-0.701**	**3.248**	**3.248**
Aporcelaimellus	Arctoseius	**0.548**	**-0.152**	**5.030**	**3.248**
Aporcelaimellus	Hypoaspis	**0.548**	**0.334**	**5.030**	**3.549**
Aporcelaimellus	Lysigamasus	**0.548**	**0.407**	**5.030**	**3.248**
Aporcelaimellus	Pergamasus	**0.548**	**1.081**	**5.030**	**3.248**
Aporcelaimellus	Aporcelaimellus	**0.548**	**0.548**	**5.030**	**5.030**
Aporcelaimellus	Dorylaimoidea	**0.548**	**-0.604**	**5.030**	**5.274**
Aporcelaimellus	Qudsianematidae	**0.548**	**-0.207**	**5.030**	**4.729**
Aporcelaimellus	Eupodes	**0.548**	**0.005**	**5.030**	**4.026**
Aporcelaimellus	Protodinychus	**0.548**	**0.549**	**5.030**	**3.549**
Aporcelaimellus	Scheloribates	**0.548**	**0.202**	**5.030**	**3.549**
Aporcelaimellus	Scutacarus	**0.548**	**-0.608**	**5.030**	**4.093**
Aporcelaimellus	Tarsonemus	**0.548**	**-0.701**	**5.030**	**3.248**
Dorylaimoidea	Arctoseius	**-0.604**	**-0.152**	**5.274**	**3.248**
Dorylaimoidea	Hypoaspis	**-0.604**	**0.334**	**5.274**	**3.549**
Dorylaimoidea	Lysigamasus	**-0.604**	**0.407**	**5.274**	**3.248**
Dorylaimoidea	Pergamasus	**-0.604**	**1.081**	**5.274**	**3.248**
Dorylaimoidea	Aporcelaimellus	**-0.604**	**0.548**	**5.274**	**5.030**
Dorylaimoidea	Dorylaimoidea	**-0.604**	**-0.604**	**5.274**	**5.274**
Dorylaimoidea	Qudsianematidae	**-0.604**	**-0.207**	**5.274**	**4.729**
Dorylaimoidea	Eupodes	**-0.604**	**0.005**	**5.274**	**4.026**
Dorylaimoidea	Protodinychus	**-0.604**	**0.549**	**5.274**	**3.549**
Dorylaimoidea	Scheloribates	**-0.604**	**0.202**	**5.274**	**3.549**
Dorylaimoidea	Scutacarus	**-0.604**	**-0.608**	**5.274**	**4.093**
Dorylaimoidea	Tarsonemus	**-0.604**	**-0.701**	**5.274**	**3.248**
Qudsianematidae	Arctoseius	**-0.207**	**-0.152**	**4.729**	**3.248**
Qudsianematidae	Hypoaspis	**-0.207**	**0.334**	**4.729**	**3.549**
Qudsianematidae	Lysigamasus	**-0.207**	**0.407**	**4.729**	**3.248**
Qudsianematidae	Pergamasus	**-0.207**	**1.081**	**4.729**	**3.248**
Qudsianematidae	Aporcelaimellus	**-0.207**	**0.548**	**4.729**	**5.030**
Qudsianematidae	Dorylaimoidea	**-0.207**	**-0.604**	**4.729**	**5.274**
Qudsianematidae	Qudsianematidae	**-0.207**	**-0.207**	**4.729**	**4.729**
Qudsianematidae	Eupodes	**-0.207**	**0.005**	**4.729**	**4.026**
Qudsianematidae	Protodinychus	**-0.207**	**0.549**	**4.729**	**3.549**
Qudsianematidae	Scheloribates	**-0.207**	**0.202**	**4.729**	**3.549**
Qudsianematidae	Scutacarus	**-0.207**	**-0.608**	**4.729**	**4.093**
Qudsianematidae	Tarsonemus	**-0.207**	**-0.701**	**4.729**	**3.248**
Eupodes	Arctoseius	**0.005**	**-0.152**	**4.026**	**3.248**
Eupodes	Hypoaspis	**0.005**	**0.334**	**4.026**	**3.549**
Eupodes	Lysigamasus	**0.005**	**0.407**	**4.026**	**3.248**

Eupodes	Pergamasus	**0.005**	**1.081**	**4.026**	**3.248**
Eupodes	Aporcelaimellus	**0.005**	**0.548**	**4.026**	**5.030**
Eupodes	Dorylaimoidea	**0.005**	**-0.604**	**4.026**	**5.274**
Eupodes	Qudsianematidae	**0.005**	**-0.207**	**4.026**	**4.729**
Eupodes	Eupodes	**0.005**	**0.005**	**4.026**	**4.026**
Eupodes	Protodinychus	**0.005**	**0.549**	**4.026**	**3.549**
Eupodes	Scheloribates	**0.005**	**0.202**	**4.026**	**3.549**
Eupodes	Scutacarus	**0.005**	**-0.608**	**4.026**	**4.093**
Eupodes	Tarsonemus	**0.005**	**-0.701**	**4.026**	**3.248**
Protodinychus	Arctoseius	**0.549**	**-0.152**	**3.549**	**3.248**
Protodinychus	Hypoaspis	**0.549**	**0.334**	**3.549**	**3.549**
Protodinychus	Lysigamasus	**0.549**	**0.407**	**3.549**	**3.248**
Protodinychus	Pergamasus	**0.549**	**1.081**	**3.549**	**3.248**
Protodinychus	Aporcelaimellus	**0.549**	**0.548**	**3.549**	**5.030**
Protodinychus	Dorylaimoidea	**0.549**	**-0.604**	**3.549**	**5.274**
Protodinychus	Qudsianematidae	**0.549**	**-0.207**	**3.549**	**4.729**
Protodinychus	Eupodes	**0.549**	**0.005**	**3.549**	**4.026**
Protodinychus	Protodinychus	**0.549**	**0.549**	**3.549**	**3.549**
Protodinychus	Scheloribates	**0.549**	**0.202**	**3.549**	**3.549**
Protodinychus	Scutacarus	**0.549**	**-0.608**	**3.549**	**4.093**
Protodinychus	Tarsonemus	**0.549**	**-0.701**	**3.549**	**3.248**
Scheloribates	Arctoseius	**0.202**	**-0.152**	**3.549**	**3.248**
Scheloribates	Hypoaspis	**0.202**	**0.334**	**3.549**	**3.549**
Scheloribates	Lysigamasus	**0.202**	**0.407**	**3.549**	**3.248**
Scheloribates	Pergamasus	**0.202**	**1.081**	**3.549**	**3.248**
Scheloribates	Aporcelaimellus	**0.202**	**0.548**	**3.549**	**5.030**
Scheloribates	Dorylaimoidea	**0.202**	**-0.604**	**3.549**	**5.274**
Scheloribates	Qudsianematidae	**0.202**	**-0.207**	**3.549**	**4.729**
Scheloribates	Eupodes	**0.202**	**0.005**	**3.549**	**4.026**
Scheloribates	Protodinychus	**0.202**	**0.549**	**3.549**	**3.549**
Scheloribates	Scheloribates	**0.202**	**0.202**	**3.549**	**3.549**
Scheloribates	Scutacarus	**0.202**	**-0.608**	**3.549**	**4.093**
Scheloribates	Tarsonemus	**0.202**	**-0.701**	**3.549**	**3.248**
Scutacarus	Arctoseius	**-0.608**	**-0.152**	**4.093**	**3.248**
Scutacarus	Hypoaspis	**-0.608**	**0.334**	**4.093**	**3.549**
Scutacarus	Lysigamasus	**-0.608**	**0.407**	**4.093**	**3.248**
Scutacarus	Pergamasus	**-0.608**	**1.081**	**4.093**	**3.248**
Scutacarus	Aporcelaimellus	**-0.608**	**0.548**	**4.093**	**5.030**
Scutacarus	Dorylaimoidea	**-0.608**	**-0.604**	**4.093**	**5.274**
Scutacarus	Qudsianematidae	**-0.608**	**-0.207**	**4.093**	**4.729**
Scutacarus	Eupodes	**-0.608**	**0.005**	**4.093**	**4.026**
Scutacarus	Protodinychus	**-0.608**	**0.549**	**4.093**	**3.549**
Scutacarus	Scheloribates	**-0.608**	**0.202**	**4.093**	**3.549**
Scutacarus	Scutacarus	**-0.608**	**-0.608**	**4.093**	**4.093**
Scutacarus	Tarsonemus	**-0.608**	**-0.701**	**4.093**	**3.248**
Tarsonemus	Arctoseius	**-0.701**	**-0.152**	**3.248**	**3.248**
Tarsonemus	Hypoaspis	**-0.701**	**0.334**	**3.248**	**3.549**
Tarsonemus	Lysigamasus	**-0.701**	**0.407**	**3.248**	**3.248**
Tarsonemus	Pergamasus	**-0.701**	**1.081**	**3.248**	**3.248**
Tarsonemus	Aporcelaimellus	**-0.701**	**0.548**	**3.248**	**5.030**
Tarsonemus	Dorylaimoidea	**-0.701**	**-0.604**	**3.248**	**5.274**

Tarsonemus	Qudsianematidae	**-0.701**	**-0.207**	**3.248**	**4.729**
Tarsonemus	Eupodes	**-0.701**	**0.005**	**3.248**	**4.026**
Tarsonemus	Protodinychus	**-0.701**	**0.549**	**3.248**	**3.549**
Tarsonemus	Scheloribates	**-0.701**	**0.202**	**3.248**	**3.549**
Tarsonemus	Scutacarus	**-0.701**	**-0.608**	**3.248**	**4.093**
Tarsonemus	Tarsonemus	**-0.701**	**-0.701**	**3.248**	**3.248**

Resource	Consumer	Mres	Mconsumer	Nres	Nconsumer
Aglenchus	Tripyla	**-1.053**	**-0.420**	**5.642**	**4.739**
Aglenchus	Alliphis	**-1.053**	**0.053**	**5.642**	**2.546**
Aglenchus	Dendrolaelaps	**-1.053**	**0.027**	**5.642**	**2.722**
Aglenchus	Lysigamasus	**-1.053**	**0.407**	**5.642**	**2.722**
Aglenchus	Parasitus	**-1.053**	**0.859**	**5.642**	**2.546**
Aglenchus	Uropoda	**-1.053**	**0.481**	**5.642**	**2.245**
Aglenchus	Aporcelaimellus	**-1.053**	**0.548**	**5.642**	**5.216**
Aglenchus	Dorylaimoidea	**-1.053**	**-0.604**	**5.642**	**5.216**
Aglenchus	Qudsianematidae	**-1.053**	**-0.207**	**5.642**	**4.739**
Aglenchus	Thornematidae	**-1.053**	**-0.470**	**5.642**	**4.739**
Aglenchus	Eupodes	**-1.053**	**0.005**	**5.642**	**2.245**
Aglenchus	Tarsonemus	**-1.053**	**-0.701**	**5.642**	**2.722**
Filenchus	Tripyla	**-1.033**	**-0.420**	**5.517**	**4.739**
Filenchus	Alliphis	**-1.033**	**0.053**	**5.517**	**2.546**
Filenchus	Dendrolaelaps	**-1.033**	**0.027**	**5.517**	**2.722**
Filenchus	Lysigamasus	**-1.033**	**0.407**	**5.517**	**2.722**
Filenchus	Parasitus	**-1.033**	**0.859**	**5.517**	**2.546**
Filenchus	Uropoda	**-1.033**	**0.481**	**5.517**	**2.245**
Filenchus	Aporcelaimellus	**-1.033**	**0.548**	**5.517**	**5.216**
Filenchus	Dorylaimoidea	**-1.033**	**-0.604**	**5.517**	**5.216**
Filenchus	Qudsianematidae	**-1.033**	**-0.207**	**5.517**	**4.739**
Filenchus	Thornematidae	**-1.033**	**-0.470**	**5.517**	**4.739**
Filenchus	Eupodes	**-1.033**	**0.005**	**5.517**	**2.245**
Filenchus	Tarsonemus	**-1.033**	**-0.701**	**5.517**	**2.722**
Helicotylenchus	Tripyla	**-0.792**	**-0.420**	**5.438**	**4.739**
Helicotylenchus	Alliphis	**-0.792**	**0.053**	**5.438**	**2.546**
Helicotylenchus	Dendrolaelaps	**-0.792**	**0.027**	**5.438**	**2.722**
Helicotylenchus	Lysigamasus	**-0.792**	**0.407**	**5.438**	**2.722**
Helicotylenchus	Parasitus	**-0.792**	**0.859**	**5.438**	**2.546**
Helicotylenchus	Uropoda	**-0.792**	**0.481**	**5.438**	**2.245**
Helicotylenchus	Aporcelaimellus	**-0.792**	**0.548**	**5.438**	**5.216**
Helicotylenchus	Dorylaimoidea	**-0.792**	**-0.604**	**5.438**	**5.216**
Helicotylenchus	Qudsianematidae	**-0.792**	**-0.207**	**5.438**	**4.739**
Helicotylenchus	Thornematidae	**-0.792**	**-0.470**	**5.438**	**4.739**
Helicotylenchus	Eupodes	**-0.792**	**0.005**	**5.438**	**2.245**
Helicotylenchus	Tarsonemus	**-0.792**	**-0.701**	**5.438**	**2.722**
Heterodera	Tripyla	**-0.883**	**-0.420**	**5.040**	**4.739**
Heterodera	Alliphis	**-0.883**	**0.053**	**5.040**	**2.546**
Heterodera	Dendrolaelaps	**-0.883**	**0.027**	**5.040**	**2.722**
Heterodera	Lysigamasus	**-0.883**	**0.407**	**5.040**	**2.722**
Heterodera	Parasitus	**-0.883**	**0.859**	**5.040**	**2.546**
Heterodera	Uropoda	**-0.883**	**0.481**	**5.040**	**2.245**
Heterodera	Aporcelaimellus	**-0.883**	**0.548**	**5.040**	**5.216**
Heterodera	Dorylaimoidea	**-0.883**	**-0.604**	**5.040**	**5.216**
Heterodera	Qudsianematidae	**-0.883**	**-0.207**	**5.040**	**4.739**
Heterodera	Thornematidae	**-0.883**	**-0.470**	**5.040**	**4.739**
Heterodera	Eupodes	**-0.883**	**0.005**	**5.040**	**2.245**
Heterodera	Tarsonemus	**-0.883**	**-0.701**	**5.040**	**2.722**
Paratylenchus	Tripyla	**-1.244**	**-0.420**	**5.040**	**4.739**
Paratylenchus	Alliphis	**-1.244**	**0.053**	**5.040**	**2.546**

Paratylenchus	Dendrolaelaps	**-1.244**	**0.027**	**5.040**	**2.722**
Paratylenchus	Lysigamasus	**-1.244**	**0.407**	**5.040**	**2.722**
Paratylenchus	Parasitus	**-1.244**	**0.859**	**5.040**	**2.546**
Paratylenchus	Uropoda	**-1.244**	**0.481**	**5.040**	**2.245**
Paratylenchus	Aporcelaimellus	**-1.244**	**0.548**	**5.040**	**5.216**
Paratylenchus	Dorylaimoidea	**-1.244**	**-0.604**	**5.040**	**5.216**
Paratylenchus	Qudsianematidae	**-1.244**	**-0.207**	**5.040**	**4.739**
Paratylenchus	Thornematidae	**-1.244**	**-0.470**	**5.040**	**4.739**
Paratylenchus	Eupodes	**-1.244**	**0.005**	**5.040**	**2.245**
Paratylenchus	Tarsonemus	**-1.244**	**-0.701**	**5.040**	**2.722**
Pratylenchus	Tripyla	**-1.226**	**-0.420**	**4.739**	**4.739**
Pratylenchus	Alliphis	**-1.226**	**0.053**	**4.739**	**2.546**
Pratylenchus	Dendrolaelaps	**-1.226**	**0.027**	**4.739**	**2.722**
Pratylenchus	Lysigamasus	**-1.226**	**0.407**	**4.739**	**2.722**
Pratylenchus	Parasitus	**-1.226**	**0.859**	**4.739**	**2.546**
Pratylenchus	Uropoda	**-1.226**	**0.481**	**4.739**	**2.245**
Pratylenchus	Aporcelaimellus	**-1.226**	**0.548**	**4.739**	**5.216**
Pratylenchus	Dorylaimoidea	**-1.226**	**-0.604**	**4.739**	**5.216**
Pratylenchus	Qudsianematidae	**-1.226**	**-0.207**	**4.739**	**4.739**
Pratylenchus	Thornematidae	**-1.226**	**-0.470**	**4.739**	**4.739**
Pratylenchus	Eupodes	**-1.226**	**0.005**	**4.739**	**2.245**
Pratylenchus	Tarsonemus	**-1.226**	**-0.701**	**4.739**	**2.722**
Tylenchorhynchus	Tripyla	**-0.664**	**-0.420**	**5.517**	**4.739**
Tylenchorhynchus	Alliphis	**-0.664**	**0.053**	**5.517**	**2.546**
Tylenchorhynchus	Dendrolaelaps	**-0.664**	**0.027**	**5.517**	**2.722**
Tylenchorhynchus	Lysigamasus	**-0.664**	**0.407**	**5.517**	**2.722**
Tylenchorhynchus	Parasitus	**-0.664**	**0.859**	**5.517**	**2.546**
Tylenchorhynchus	Uropoda	**-0.664**	**0.481**	**5.517**	**2.245**
Tylenchorhynchus	Aporcelaimellus	**-0.664**	**0.548**	**5.517**	**5.216**
Tylenchorhynchus	Dorylaimoidea	**-0.664**	**-0.604**	**5.517**	**5.216**
Tylenchorhynchus	Qudsianematidae	**-0.664**	**-0.207**	**5.517**	**4.739**
Tylenchorhynchus	Thornematidae	**-0.664**	**-0.470**	**5.517**	**4.739**
Tylenchorhynchus	Eupodes	**-0.664**	**0.005**	**5.517**	**2.245**
Tylenchorhynchus	Tarsonemus	**-0.664**	**-0.701**	**5.517**	**2.722**
Tydeidae	Bdella	**-0.608**	**0.816**	**2.546**	**2.245**
Tydeidae	Dendrolaelaps	**-0.608**	**0.027**	**2.546**	**2.722**
Tydeidae	Lysigamasus	**-0.608**	**0.407**	**2.546**	**2.722**
Tydeidae	Parasitus	**-0.608**	**0.859**	**2.546**	**2.546**
Tydeidae	Uropoda	**-0.608**	**0.481**	**2.546**	**2.245**
Tydeidae	Aporcelaimellus	**-0.608**	**0.548**	**2.546**	**5.216**
Tydeidae	Dorylaimoidea	**-0.608**	**-0.604**	**2.546**	**5.216**
Tydeidae	Qudsianematidae	**-0.608**	**-0.207**	**2.546**	**4.739**
Tydeidae	Thornematidae	**-0.608**	**-0.470**	**2.546**	**4.739**
Tydeidae	Eupodes	**-0.608**	**0.005**	**2.546**	**2.245**
Tydeidae	Tarsonemus	**-0.608**	**-0.701**	**2.546**	**2.722**
Sminthurinus	Dendrolaelaps	**0.618**	**0.027**	**3.421**	**2.722**
Sminthurinus	Lysigamasus	**0.618**	**0.407**	**3.421**	**2.722**
Sminthurinus	Parasitus	**0.618**	**0.859**	**3.421**	**2.546**
Sminthurinus	Uropoda	**0.618**	**0.481**	**3.421**	**2.245**
Sminthurinus	Aporcelaimellus	**0.618**	**0.548**	**3.421**	**5.216**
Sminthurinus	Dorylaimoidea	**0.618**	**-0.604**	**3.421**	**5.216**

Sminthurinus	Qudsianematidae	**0.618**	**-0.207**	**3.421**	**4.739**
Sminthurinus	Thornematidae	**0.618**	**-0.470**	**3.421**	**4.739**
Sminthurinus	Eupodes	**0.618**	**0.005**	**3.421**	**2.245**
Sminthurinus	Tarsonemus	**0.618**	**-0.701**	**3.421**	**2.722**
Aphelenchoides	Tripyla	**-1.496**	**-0.420**	**5.040**	**4.739**
Aphelenchoides	Alliphis	**-1.496**	**0.053**	**5.040**	**2.546**
Aphelenchoides	Dendrolaelaps	**-1.496**	**0.027**	**5.040**	**2.722**
Aphelenchoides	Lysigamasus	**-1.496**	**0.407**	**5.040**	**2.722**
Aphelenchoides	Parasitus	**-1.496**	**0.859**	**5.040**	**2.546**
Aphelenchoides	Uropoda	**-1.496**	**0.481**	**5.040**	**2.245**
Aphelenchoides	Aporcelaimellus	**-1.496**	**0.548**	**5.040**	**5.216**
Aphelenchoides	Dorylaimoidea	**-1.496**	**-0.604**	**5.040**	**5.216**
Aphelenchoides	Qudsianematidae	**-1.496**	**-0.207**	**5.040**	**4.739**
Aphelenchoides	Thornematidae	**-1.496**	**-0.470**	**5.040**	**4.739**
Aphelenchoides	Eupodes	**-1.496**	**0.005**	**5.040**	**2.245**
Aphelenchoides	Tarsonemus	**-1.496**	**-0.701**	**5.040**	**2.722**
Tylenchidae	Tripyla	**-1.360**	**-0.420**	**5.915**	**4.739**
Tylenchidae	Alliphis	**-1.360**	**0.053**	**5.915**	**2.546**
Tylenchidae	Dendrolaelaps	**-1.360**	**0.027**	**5.915**	**2.722**
Tylenchidae	Lysigamasus	**-1.360**	**0.407**	**5.915**	**2.722**
Tylenchidae	Parasitus	**-1.360**	**0.859**	**5.915**	**2.546**
Tylenchidae	Uropoda	**-1.360**	**0.481**	**5.915**	**2.245**
Tylenchidae	Aporcelaimellus	**-1.360**	**0.548**	**5.915**	**5.216**
Tylenchidae	Dorylaimoidea	**-1.360**	**-0.604**	**5.915**	**5.216**
Tylenchidae	Qudsianematidae	**-1.360**	**-0.207**	**5.915**	**4.739**
Tylenchidae	Thornematidae	**-1.360**	**-0.470**	**5.915**	**4.739**
Tylenchidae	Eupodes	**-1.360**	**0.005**	**5.915**	**2.245**
Tylenchidae	Tarsonemus	**-1.360**	**-0.701**	**5.915**	**2.722**
Pygmephorus	Bdella	**-0.376**	**0.816**	**2.245**	**2.245**
Pygmephorus	Dendrolaelaps	**-0.376**	**0.027**	**2.245**	**2.722**
Pygmephorus	Lysigamasus	**-0.376**	**0.407**	**2.245**	**2.722**
Pygmephorus	Parasitus	**-0.376**	**0.859**	**2.245**	**2.546**
Pygmephorus	Uropoda	**-0.376**	**0.481**	**2.245**	**2.245**
Pygmephorus	Aporcelaimellus	**-0.376**	**0.548**	**2.245**	**5.216**
Pygmephorus	Dorylaimoidea	**-0.376**	**-0.604**	**2.245**	**5.216**
Pygmephorus	Qudsianematidae	**-0.376**	**-0.207**	**2.245**	**4.739**
Pygmephorus	Thornematidae	**-0.376**	**-0.470**	**2.245**	**4.739**
Pygmephorus	Eupodes	**-0.376**	**0.005**	**2.245**	**2.245**
Pygmephorus	Tarsonemus	**-0.376**	**-0.701**	**2.245**	**2.722**
Tyrophagus	Bdella	**0.005**	**0.816**	**2.245**	**2.245**
Tyrophagus	Dendrolaelaps	**0.005**	**0.027**	**2.245**	**2.722**
Tyrophagus	Lysigamasus	**0.005**	**0.407**	**2.245**	**2.722**
Tyrophagus	Parasitus	**0.005**	**0.859**	**2.245**	**2.546**
Tyrophagus	Uropoda	**0.005**	**0.481**	**2.245**	**2.245**
Tyrophagus	Aporcelaimellus	**0.005**	**0.548**	**2.245**	**5.216**
Tyrophagus	Dorylaimoidea	**0.005**	**-0.604**	**2.245**	**5.216**
Tyrophagus	Qudsianematidae	**0.005**	**-0.207**	**2.245**	**4.739**
Tyrophagus	Thornematidae	**0.005**	**-0.470**	**2.245**	**4.739**
Tyrophagus	Eupodes	**0.005**	**0.005**	**2.245**	**2.245**
Tyrophagus	Tarsonemus	**0.005**	**-0.701**	**2.245**	**2.722**
Friesea	Dendrolaelaps	**0.434**	**0.027**	**2.847**	**2.722**

Friesea	Lysigamasus	**0.434**	**0.407**	**2.847**	**2.722**
Friesea	Parasitus	**0.434**	**0.859**	**2.847**	**2.546**
Friesea	Uropoda	**0.434**	**0.481**	**2.847**	**2.245**
Friesea	Aporcelaimellus	**0.434**	**0.548**	**2.847**	**5.216**
Friesea	Dorylaimoidea	**0.434**	**-0.604**	**2.847**	**5.216**
Friesea	Qudsianematidae	**0.434**	**-0.207**	**2.847**	**4.739**
Friesea	Thornematidae	**0.434**	**-0.470**	**2.847**	**4.739**
Friesea	Eupodes	**0.434**	**0.005**	**2.847**	**2.245**
Friesea	Tarsonemus	**0.434**	**-0.701**	**2.847**	**2.722**
Isotoma	Dendrolaelaps	**1.898**	**0.027**	**3.587**	**2.722**
Isotoma	Lysigamasus	**1.898**	**0.407**	**3.587**	**2.722**
Isotoma	Parasitus	**1.898**	**0.859**	**3.587**	**2.546**
Isotoma	Uropoda	**1.898**	**0.481**	**3.587**	**2.245**
Isotoma	Aporcelaimellus	**1.898**	**0.548**	**3.587**	**5.216**
Isotoma	Dorylaimoidea	**1.898**	**-0.604**	**3.587**	**5.216**
Isotoma	Qudsianematidae	**1.898**	**-0.207**	**3.587**	**4.739**
Isotoma	Thornematidae	**1.898**	**-0.470**	**3.587**	**4.739**
Isotoma	Eupodes	**1.898**	**0.005**	**3.587**	**2.245**
Isotoma	Tarsonemus	**1.898**	**-0.701**	**3.587**	**2.722**
Achaeta	Dendrolaelaps	**1.542**	**0.027**	**3.850**	**2.722**
Achaeta	Lysigamasus	**1.542**	**0.407**	**3.850**	**2.722**
Achaeta	Parasitus	**1.542**	**0.859**	**3.850**	**2.546**
Achaeta	Uropoda	**1.542**	**0.481**	**3.850**	**2.245**
Achaeta	Aporcelaimellus	**1.542**	**0.548**	**3.850**	**5.216**
Achaeta	Dorylaimoidea	**1.542**	**-0.604**	**3.850**	**5.216**
Achaeta	Qudsianematidae	**1.542**	**-0.207**	**3.850**	**4.739**
Achaeta	Thornematidae	**1.542**	**-0.470**	**3.850**	**4.739**
Achaeta	Eupodes	**1.542**	**0.005**	**3.850**	**2.245**
Achaeta	Tarsonemus	**1.542**	**-0.701**	**3.850**	**2.722**
Cognettia	Dendrolaelaps	**2.300**	**0.027**	**2.549**	**2.722**
Cognettia	Lysigamasus	**2.300**	**0.407**	**2.549**	**2.722**
Cognettia	Parasitus	**2.300**	**0.859**	**2.549**	**2.546**
Cognettia	Uropoda	**2.300**	**0.481**	**2.549**	**2.245**
Cognettia	Aporcelaimellus	**2.300**	**0.548**	**2.549**	**5.216**
Cognettia	Dorylaimoidea	**2.300**	**-0.604**	**2.549**	**5.216**
Cognettia	Qudsianematidae	**2.300**	**-0.207**	**2.549**	**4.739**
Cognettia	Thornematidae	**2.300**	**-0.470**	**2.549**	**4.739**
Cognettia	Eupodes	**2.300**	**0.005**	**2.549**	**2.245**
Cognettia	Tarsonemus	**2.300**	**-0.701**	**2.549**	**2.722**
Fridericia	Dendrolaelaps	**2.706**	**0.027**	**4.792**	**2.722**
Fridericia	Lysigamasus	**2.706**	**0.407**	**4.792**	**2.722**
Fridericia	Parasitus	**2.706**	**0.859**	**4.792**	**2.546**
Fridericia	Uropoda	**2.706**	**0.481**	**4.792**	**2.245**
Fridericia	Aporcelaimellus	**2.706**	**0.548**	**4.792**	**5.216**
Fridericia	Dorylaimoidea	**2.706**	**-0.604**	**4.792**	**5.216**
Fridericia	Qudsianematidae	**2.706**	**-0.207**	**4.792**	**4.739**
Fridericia	Thornematidae	**2.706**	**-0.470**	**4.792**	**4.739**
Fridericia	Eupodes	**2.706**	**0.005**	**4.792**	**2.245**
Fridericia	Tarsonemus	**2.706**	**-0.701**	**4.792**	**2.722**
Acrobeloides	Tripyla	**-1.171**	**-0.420**	**4.739**	**4.739**
Acrobeloides	Alliphis	**-1.171**	**0.053**	**4.739**	**2.546**

Acrobeloides	Dendrolaelaps	**-1.171**	**0.027**	**4.739**	**2.722**
Acrobeloides	Lysigamasus	**-1.171**	**0.407**	**4.739**	**2.722**
Acrobeloides	Parasitus	**-1.171**	**0.859**	**4.739**	**2.546**
Acrobeloides	Uropoda	**-1.171**	**0.481**	**4.739**	**2.245**
Acrobeloides	Aporcelaimellus	**-1.171**	**0.548**	**4.739**	**5.216**
Acrobeloides	Dorylaimoidea	**-1.171**	**-0.604**	**4.739**	**5.216**
Acrobeloides	Qudsianematidae	**-1.171**	**-0.207**	**4.739**	**4.739**
Acrobeloides	Thornematidae	**-1.171**	**-0.470**	**4.739**	**4.739**
Acrobeloides	Eupodes	**-1.171**	**0.005**	**4.739**	**2.245**
Acrobeloides	Tarsonemus	**-1.171**	**-0.701**	**4.739**	**2.722**
Anaplectus	Tripyla	**-0.519**	**-0.420**	**5.438**	**4.739**
Anaplectus	Alliphis	**-0.519**	**0.053**	**5.438**	**2.546**
Anaplectus	Dendrolaelaps	**-0.519**	**0.027**	**5.438**	**2.722**
Anaplectus	Lysigamasus	**-0.519**	**0.407**	**5.438**	**2.722**
Anaplectus	Parasitus	**-0.519**	**0.859**	**5.438**	**2.546**
Anaplectus	Uropoda	**-0.519**	**0.481**	**5.438**	**2.245**
Anaplectus	Aporcelaimellus	**-0.519**	**0.548**	**5.438**	**5.216**
Anaplectus	Dorylaimoidea	**-0.519**	**-0.604**	**5.438**	**5.216**
Anaplectus	Qudsianematidae	**-0.519**	**-0.207**	**5.438**	**4.739**
Anaplectus	Thornematidae	**-0.519**	**-0.470**	**5.438**	**4.739**
Anaplectus	Eupodes	**-0.519**	**0.005**	**5.438**	**2.245**
Anaplectus	Tarsonemus	**-0.519**	**-0.701**	**5.438**	**2.722**
Cephalobidae	Tripyla	**-1.055**	**-0.420**	**5.040**	**4.739**
Cephalobidae	Alliphis	**-1.055**	**0.053**	**5.040**	**2.546**
Cephalobidae	Dendrolaelaps	**-1.055**	**0.027**	**5.040**	**2.722**
Cephalobidae	Lysigamasus	**-1.055**	**0.407**	**5.040**	**2.722**
Cephalobidae	Parasitus	**-1.055**	**0.859**	**5.040**	**2.546**
Cephalobidae	Uropoda	**-1.055**	**0.481**	**5.040**	**2.245**
Cephalobidae	Aporcelaimellus	**-1.055**	**0.548**	**5.040**	**5.216**
Cephalobidae	Dorylaimoidea	**-1.055**	**-0.604**	**5.040**	**5.216**
Cephalobidae	Qudsianematidae	**-1.055**	**-0.207**	**5.040**	**4.739**
Cephalobidae	Thornematidae	**-1.055**	**-0.470**	**5.040**	**4.739**
Cephalobidae	Eupodes	**-1.055**	**0.005**	**5.040**	**2.245**
Cephalobidae	Tarsonemus	**-1.055**	**-0.701**	**5.040**	**2.722**
Eucephalobus	Tripyla	**-1.244**	**-0.420**	**6.101**	**4.739**
Eucephalobus	Alliphis	**-1.244**	**0.053**	**6.101**	**2.546**
Eucephalobus	Dendrolaelaps	**-1.244**	**0.027**	**6.101**	**2.722**
Eucephalobus	Lysigamasus	**-1.244**	**0.407**	**6.101**	**2.722**
Eucephalobus	Parasitus	**-1.244**	**0.859**	**6.101**	**2.546**
Eucephalobus	Uropoda	**-1.244**	**0.481**	**6.101**	**2.245**
Eucephalobus	Aporcelaimellus	**-1.244**	**0.548**	**6.101**	**5.216**
Eucephalobus	Dorylaimoidea	**-1.244**	**-0.604**	**6.101**	**5.216**
Eucephalobus	Qudsianematidae	**-1.244**	**-0.207**	**6.101**	**4.739**
Eucephalobus	Thornematidae	**-1.244**	**-0.470**	**6.101**	**4.739**
Eucephalobus	Eupodes	**-1.244**	**0.005**	**6.101**	**2.245**
Eucephalobus	Tarsonemus	**-1.244**	**-0.701**	**6.101**	**2.722**
Panagrolaimus	Tripyla	**-0.945**	**-0.420**	**5.584**	**4.739**
Panagrolaimus	Alliphis	**-0.945**	**0.053**	**5.584**	**2.546**
Panagrolaimus	Dendrolaelaps	**-0.945**	**0.027**	**5.584**	**2.722**
Panagrolaimus	Lysigamasus	**-0.945**	**0.407**	**5.584**	**2.722**
Panagrolaimus	Parasitus	**-0.945**	**0.859**	**5.584**	**2.546**

Panagrolaimus	Uropoda	-0.945	0.481	5.584	2.245
Panagrolaimus	Aporcelaimellus	-0.945	0.548	5.584	5.216
Panagrolaimus	Dorylaimoidea	-0.945	-0.604	5.584	5.216
Panagrolaimus	Qudsianematidae	-0.945	-0.207	5.584	4.739
Panagrolaimus	Thornematidae	-0.945	-0.470	5.584	4.739
Panagrolaimus	Eupodes	-0.945	0.005	5.584	2.245
Panagrolaimus	Tarsonemus	-0.945	-0.701	5.584	2.722
Plectus	Tripyla	-0.583	-0.420	5.915	4.739
Plectus	Alliphis	-0.583	0.053	5.915	2.546
Plectus	Dendrolaelaps	-0.583	0.027	5.915	2.722
Plectus	Lysigamasus	-0.583	0.407	5.915	2.722
Plectus	Parasitus	-0.583	0.859	5.915	2.546
Plectus	Uropoda	-0.583	0.481	5.915	2.245
Plectus	Aporcelaimellus	-0.583	0.548	5.915	5.216
Plectus	Dorylaimoidea	-0.583	-0.604	5.915	5.216
Plectus	Qudsianematidae	-0.583	-0.207	5.915	4.739
Plectus	Thornematidae	-0.583	-0.470	5.915	4.739
Plectus	Eupodes	-0.583	0.005	5.915	2.245
Plectus	Tarsonemus	-0.583	-0.701	5.915	2.722
Rhabditidae	Tripyla	-0.692	-0.420	6.137	4.739
Rhabditidae	Alliphis	-0.692	0.053	6.137	2.546
Rhabditidae	Dendrolaelaps	-0.692	0.027	6.137	2.722
Rhabditidae	Lysigamasus	-0.692	0.407	6.137	2.722
Rhabditidae	Parasitus	-0.692	0.859	6.137	2.546
Rhabditidae	Uropoda	-0.692	0.481	6.137	2.245
Rhabditidae	Aporcelaimellus	-0.692	0.548	6.137	5.216
Rhabditidae	Dorylaimoidea	-0.692	-0.604	6.137	5.216
Rhabditidae	Qudsianematidae	-0.692	-0.207	6.137	4.739
Rhabditidae	Thornematidae	-0.692	-0.470	6.137	4.739
Rhabditidae	Eupodes	-0.692	0.005	6.137	2.245
Rhabditidae	Tarsonemus	-0.692	-0.701	6.137	2.722
Histiostoma	Bdella	-0.805	0.816	3.199	2.245
Histiostoma	Dendrolaelaps	-0.805	0.027	3.199	2.722
Histiostoma	Lysigamasus	-0.805	0.407	3.199	2.722
Histiostoma	Parasitus	-0.805	0.859	3.199	2.546
Histiostoma	Uropoda	-0.805	0.481	3.199	2.245
Histiostoma	Eupodes	-0.805	0.005	3.199	2.245
Histiostoma	Tarsonemus	-0.805	-0.701	3.199	2.722
Enchytraeus	Dendrolaelaps	2.115	0.027	4.105	2.722
Enchytraeus	Lysigamasus	2.115	0.407	4.105	2.722
Enchytraeus	Parasitus	2.115	0.859	4.105	2.546
Enchytraeus	Uropoda	2.115	0.481	4.105	2.245
Enchytraeus	Aporcelaimellus	2.115	0.548	4.105	5.216
Enchytraeus	Dorylaimoidea	2.115	-0.604	4.105	5.216
Enchytraeus	Qudsianematidae	2.115	-0.207	4.105	4.739
Enchytraeus	Thornematidae	2.115	-0.470	4.105	4.739
Enchytraeus	Eupodes	2.115	0.005	4.105	2.245
Enchytraeus	Tarsonemus	2.115	-0.701	4.105	2.722
Eubacteria	Acrobeloides	-6.586	-1.171	13.515	4.739
Eubacteria	Anaplectus	-6.586	-0.519	13.515	5.438
Eubacteria	Cephalobidae	-6.586	-1.055	13.515	5.040

Eubacteria	Eucephalobus	**-6.586**	**-1.244**	**13.515**	**6.101**
Eubacteria	Panagrolaimus	**-6.586**	**-0.945**	**13.515**	**5.584**
Eubacteria	Plectus	**-6.586**	**-0.583**	**13.515**	**5.915**
Eubacteria	Rhabditidae	**-6.586**	**-0.692**	**13.515**	**6.137**
Eubacteria	Histiostoma	**-6.586**	**-0.805**	**13.515**	**3.199**
Eubacteria	Enchytraeus	**-6.586**	**2.115**	**13.515**	**4.105**
Eubacteria	Dauerlarvae	**-6.586**	**-0.804**	**13.515**	**5.642**
Eubacteria	Henlea	**-6.586**	**2.661**	**13.515**	**3.891**
Eubacteria	Marionina	**-6.586**	**1.674**	**13.515**	**4.488**
Dauerlarvae	Tripyla	**-0.804**	**-0.420**	**5.642**	**4.739**
Dauerlarvae	Alliphis	**-0.804**	**0.053**	**5.642**	**2.546**
Dauerlarvae	Aporcelaimellus	**-0.804**	**0.548**	**5.642**	**5.216**
Dauerlarvae	Dorylaimoidea	**-0.804**	**-0.604**	**5.642**	**5.216**
Dauerlarvae	Qudsianematidae	**-0.804**	**-0.207**	**5.642**	**4.739**
Dauerlarvae	Thornematidae	**-0.804**	**-0.470**	**5.642**	**4.739**
Dauerlarvae	Eupodes	**-0.804**	**0.005**	**5.642**	**2.245**
Dauerlarvae	Tarsonemus	**-0.804**	**-0.701**	**5.642**	**2.722**
Henlea	Dendrolaelaps	**2.661**	**0.027**	**3.891**	**2.722**
Henlea	Lysigamasus	**2.661**	**0.407**	**3.891**	**2.722**
Henlea	Parasitus	**2.661**	**0.859**	**3.891**	**2.546**
Henlea	Uropoda	**2.661**	**0.481**	**3.891**	**2.245**
Henlea	Aporcelaimellus	**2.661**	**0.548**	**3.891**	**5.216**
Henlea	Dorylaimoidea	**2.661**	**-0.604**	**3.891**	**5.216**
Henlea	Qudsianematidae	**2.661**	**-0.207**	**3.891**	**4.739**
Henlea	Thornematidae	**2.661**	**-0.470**	**3.891**	**4.739**
Henlea	Eupodes	**2.661**	**0.005**	**3.891**	**2.245**
Henlea	Tarsonemus	**2.661**	**-0.701**	**3.891**	**2.722**
Marionina	Dendrolaelaps	**1.674**	**0.027**	**4.488**	**2.722**
Marionina	Lysigamasus	**1.674**	**0.407**	**4.488**	**2.722**
Marionina	Parasitus	**1.674**	**0.859**	**4.488**	**2.546**
Marionina	Uropoda	**1.674**	**0.481**	**4.488**	**2.245**
Marionina	Aporcelaimellus	**1.674**	**0.548**	**4.488**	**5.216**
Marionina	Dorylaimoidea	**1.674**	**-0.604**	**4.488**	**5.216**
Marionina	Qudsianematidae	**1.674**	**-0.207**	**4.488**	**4.739**
Marionina	Thornematidae	**1.674**	**-0.470**	**4.488**	**4.739**
Marionina	Eupodes	**1.674**	**0.005**	**4.488**	**2.245**
Marionina	Tarsonemus	**1.674**	**-0.701**	**4.488**	**2.722**
Hyphae and hair roots	Aglenchus	**7.191**	**-1.053**	**0.000**	**5.642**
Hyphae and hair roots	Filenchus	**7.191**	**-1.033**	**0.000**	**5.517**
Hyphae and hair roots	Helicotylenchus	**7.191**	**-0.792**	**0.000**	**5.438**
Hyphae and hair roots	Heterodera	**7.191**	**-0.883**	**0.000**	**5.040**
Hyphae and hair roots	Paratylenchus	**7.191**	**-1.244**	**0.000**	**5.040**
Hyphae and hair roots	Pratylenchus	**7.191**	**-1.226**	**0.000**	**4.739**
Hyphae and hair roots	Tylenchorhynchus	**7.191**	**-0.664**	**0.000**	**5.517**
Hyphae and hair roots	Tydeidae	**7.191**	**-0.608**	**0.000**	**2.546**
Hyphae and hair roots	Sminthurinus	**7.191**	**0.618**	**0.000**	**3.421**
Hyphae and hair roots	Aphelenchoides	**7.191**	**-1.496**	**0.000**	**5.040**
Hyphae and hair roots	Tylenchidae	**7.191**	**-1.360**	**0.000**	**5.915**
Hyphae and hair roots	Pygmephorus	**7.191**	**-0.376**	**0.000**	**2.245**
Hyphae and hair roots	Tyrophagus	**7.191**	**0.005**	**0.000**	**2.245**
Hyphae and hair roots	Friesea	**7.191**	**0.434**	**0.000**	**2.847**

Hyphae and hair roots	Isotoma	**7.191**	**1.898**	**0.000**	**3.587**
Hyphae and hair roots	Achaeta	**7.191**	**1.542**	**0.000**	**3.850**
Hyphae and hair roots	Cognettia	**7.191**	**2.300**	**0.000**	**2.549**
Hyphae and hair roots	Fridericia	**7.191**	**2.706**	**0.000**	**4.792**
Hyphae and hair roots	Aporcelaimellus	**7.191**	**0.548**	**0.000**	**5.216**
Hyphae and hair roots	Dorylaimoidea	**7.191**	**-0.604**	**0.000**	**5.216**
Hyphae and hair roots	Qudsianematidae	**7.191**	**-0.207**	**0.000**	**4.739**
Hyphae and hair roots	Thornematidae	**7.191**	**-0.470**	**0.000**	**4.739**
Hyphae and hair roots	Eupodes	**7.191**	**0.005**	**0.000**	**2.245**
Hyphae and hair roots	Tarsonemus	**7.191**	**-0.701**	**0.000**	**2.722**
Tripyla	Dendrolaelaps	**-0.420**	**0.027**	**4.739**	**2.722**
Tripyla	Lysigamasus	**-0.420**	**0.407**	**4.739**	**2.722**
Tripyla	Parasitus	**-0.420**	**0.859**	**4.739**	**2.546**
Tripyla	Uropoda	**-0.420**	**0.481**	**4.739**	**2.245**
Tripyla	Aporcelaimellus	**-0.420**	**0.548**	**4.739**	**5.216**
Tripyla	Dorylaimoidea	**-0.420**	**-0.604**	**4.739**	**5.216**
Tripyla	Qudsianematidae	**-0.420**	**-0.207**	**4.739**	**4.739**
Tripyla	Thornematidae	**-0.420**	**-0.470**	**4.739**	**4.739**
Tripyla	Eupodes	**-0.420**	**0.005**	**4.739**	**2.245**
Tripyla	Tarsonemus	**-0.420**	**-0.701**	**4.739**	**2.722**
Alliphis	Bdella	**0.053**	**0.816**	**2.546**	**2.245**
Alliphis	Dendrolaelaps	**0.053**	**0.027**	**2.546**	**2.722**
Alliphis	Lysigamasus	**0.053**	**0.407**	**2.546**	**2.722**
Alliphis	Parasitus	**0.053**	**0.859**	**2.546**	**2.546**
Alliphis	Uropoda	**0.053**	**0.481**	**2.546**	**2.245**
Alliphis	Aporcelaimellus	**0.053**	**0.548**	**2.546**	**5.216**
Alliphis	Dorylaimoidea	**0.053**	**-0.604**	**2.546**	**5.216**
Alliphis	Qudsianematidae	**0.053**	**-0.207**	**2.546**	**4.739**
Alliphis	Thornematidae	**0.053**	**-0.470**	**2.546**	**4.739**
Alliphis	Eupodes	**0.053**	**0.005**	**2.546**	**2.245**
Alliphis	Tarsonemus	**0.053**	**-0.701**	**2.546**	**2.722**
Bdella	Bdella	**0.816**	**0.816**	**2.245**	**2.245**
Bdella	Dendrolaelaps	**0.816**	**0.027**	**2.245**	**2.722**
Bdella	Lysigamasus	**0.816**	**0.407**	**2.245**	**2.722**
Bdella	Parasitus	**0.816**	**0.859**	**2.245**	**2.546**
Bdella	Uropoda	**0.816**	**0.481**	**2.245**	**2.245**
Bdella	Eupodes	**0.816**	**0.005**	**2.245**	**2.245**
Bdella	Tarsonemus	**0.816**	**-0.701**	**2.245**	**2.722**
Dendrolaelaps	Bdella	**0.027**	**0.816**	**2.722**	**2.245**
Dendrolaelaps	Aporcelaimellus	**0.027**	**0.548**	**2.722**	**5.216**
Dendrolaelaps	Dorylaimoidea	**0.027**	**-0.604**	**2.722**	**5.216**
Dendrolaelaps	Qudsianematidae	**0.027**	**-0.207**	**2.722**	**4.739**
Dendrolaelaps	Thornematidae	**0.027**	**-0.470**	**2.722**	**4.739**
Dendrolaelaps	Eupodes	**0.027**	**0.005**	**2.722**	**2.245**
Dendrolaelaps	Tarsonemus	**0.027**	**-0.701**	**2.722**	**2.722**
Lysigamasus	Bdella	**0.407**	**0.816**	**2.722**	**2.245**
Lysigamasus	Aporcelaimellus	**0.407**	**0.548**	**2.722**	**5.216**
Lysigamasus	Dorylaimoidea	**0.407**	**-0.604**	**2.722**	**5.216**
Lysigamasus	Qudsianematidae	**0.407**	**-0.207**	**2.722**	**4.739**
Lysigamasus	Thornematidae	**0.407**	**-0.470**	**2.722**	**4.739**
Lysigamasus	Eupodes	**0.407**	**0.005**	**2.722**	**2.245**

Lysigamasus	Tarsonemus	**0.407**	**-0.701**	**2.722**	**2.722**
Parasitus	Bdella	**0.859**	**0.816**	**2.546**	**2.245**
Parasitus	Aporcelaimellus	**0.859**	**0.548**	**2.546**	**5.216**
Parasitus	Dorylaimoidea	**0.859**	**-0.604**	**2.546**	**5.216**
Parasitus	Qudsianematidae	**0.859**	**-0.207**	**2.546**	**4.739**
Parasitus	Thornematidae	**0.859**	**-0.470**	**2.546**	**4.739**
Parasitus	Eupodes	**0.859**	**0.005**	**2.546**	**2.245**
Parasitus	Tarsonemus	**0.859**	**-0.701**	**2.546**	**2.722**
Uropoda	Bdella	**0.481**	**0.816**	**2.245**	**2.245**
Uropoda	Aporcelaimellus	**0.481**	**0.548**	**2.245**	**5.216**
Uropoda	Dorylaimoidea	**0.481**	**-0.604**	**2.245**	**5.216**
Uropoda	Qudsianematidae	**0.481**	**-0.207**	**2.245**	**4.739**
Uropoda	Thornematidae	**0.481**	**-0.470**	**2.245**	**4.739**
Uropoda	Eupodes	**0.481**	**0.005**	**2.245**	**2.245**
Uropoda	Tarsonemus	**0.481**	**-0.701**	**2.245**	**2.722**
Aporcelaimellus	Tripyla	**0.548**	**-0.420**	**5.216**	**4.739**
Aporcelaimellus	Alliphis	**0.548**	**0.053**	**5.216**	**2.546**
Aporcelaimellus	Dendrolaelaps	**0.548**	**0.027**	**5.216**	**2.722**
Aporcelaimellus	Lysigamasus	**0.548**	**0.407**	**5.216**	**2.722**
Aporcelaimellus	Parasitus	**0.548**	**0.859**	**5.216**	**2.546**
Aporcelaimellus	Uropoda	**0.548**	**0.481**	**5.216**	**2.245**
Aporcelaimellus	Aporcelaimellus	**0.548**	**0.548**	**5.216**	**5.216**
Aporcelaimellus	Dorylaimoidea	**0.548**	**-0.604**	**5.216**	**5.216**
Aporcelaimellus	Qudsianematidae	**0.548**	**-0.207**	**5.216**	**4.739**
Aporcelaimellus	Thornematidae	**0.548**	**-0.470**	**5.216**	**4.739**
Aporcelaimellus	Eupodes	**0.548**	**0.005**	**5.216**	**2.245**
Aporcelaimellus	Tarsonemus	**0.548**	**-0.701**	**5.216**	**2.722**
Dorylaimoidea	Tripyla	**-0.604**	**-0.420**	**5.216**	**4.739**
Dorylaimoidea	Alliphis	**-0.604**	**0.053**	**5.216**	**2.546**
Dorylaimoidea	Dendrolaelaps	**-0.604**	**0.027**	**5.216**	**2.722**
Dorylaimoidea	Lysigamasus	**-0.604**	**0.407**	**5.216**	**2.722**
Dorylaimoidea	Parasitus	**-0.604**	**0.859**	**5.216**	**2.546**
Dorylaimoidea	Uropoda	**-0.604**	**0.481**	**5.216**	**2.245**
Dorylaimoidea	Aporcelaimellus	**-0.604**	**0.548**	**5.216**	**5.216**
Dorylaimoidea	Dorylaimoidea	**-0.604**	**-0.604**	**5.216**	**5.216**
Dorylaimoidea	Qudsianematidae	**-0.604**	**-0.207**	**5.216**	**4.739**
Dorylaimoidea	Thornematidae	**-0.604**	**-0.470**	**5.216**	**4.739**
Dorylaimoidea	Eupodes	**-0.604**	**0.005**	**5.216**	**2.245**
Dorylaimoidea	Tarsonemus	**-0.604**	**-0.701**	**5.216**	**2.722**
Qudsianematidae	Tripyla	**-0.207**	**-0.420**	**4.739**	**4.739**
Qudsianematidae	Alliphis	**-0.207**	**0.053**	**4.739**	**2.546**
Qudsianematidae	Dendrolaelaps	**-0.207**	**0.027**	**4.739**	**2.722**
Qudsianematidae	Lysigamasus	**-0.207**	**0.407**	**4.739**	**2.722**
Qudsianematidae	Parasitus	**-0.207**	**0.859**	**4.739**	**2.546**
Qudsianematidae	Uropoda	**-0.207**	**0.481**	**4.739**	**2.245**
Qudsianematidae	Aporcelaimellus	**-0.207**	**0.548**	**4.739**	**5.216**
Qudsianematidae	Dorylaimoidea	**-0.207**	**-0.604**	**4.739**	**5.216**
Qudsianematidae	Qudsianematidae	**-0.207**	**-0.207**	**4.739**	**4.739**
Qudsianematidae	Thornematidae	**-0.207**	**-0.470**	**4.739**	**4.739**
Qudsianematidae	Eupodes	**-0.207**	**0.005**	**4.739**	**2.245**
Qudsianematidae	Tarsonemus	**-0.207**	**-0.701**	**4.739**	**2.722**

Thornematidae	Tripyla	**-0.470**	**-0.420**	**4.739**	**4.739**
Thornematidae	Alliphis	**-0.470**	**0.053**	**4.739**	**2.546**
Thornematidae	Dendrolaelaps	**-0.470**	**0.027**	**4.739**	**2.722**
Thornematidae	Lysigamasus	**-0.470**	**0.407**	**4.739**	**2.722**
Thornematidae	Parasitus	**-0.470**	**0.859**	**4.739**	**2.546**
Thornematidae	Uropoda	**-0.470**	**0.481**	**4.739**	**2.245**
Thornematidae	Aporcelaimellus	**-0.470**	**0.548**	**4.739**	**5.216**
Thornematidae	Dorylaimoidea	**-0.470**	**-0.604**	**4.739**	**5.216**
Thornematidae	Qudsianematidae	**-0.470**	**-0.207**	**4.739**	**4.739**
Thornematidae	Thornematidae	**-0.470**	**-0.470**	**4.739**	**4.739**
Thornematidae	Eupodes	**-0.470**	**0.005**	**4.739**	**2.245**
Thornematidae	Tarsonemus	**-0.470**	**-0.701**	**4.739**	**2.722**
Eupodes	Bdella	**0.005**	**0.816**	**2.245**	**2.245**
Eupodes	Dendrolaelaps	**0.005**	**0.027**	**2.245**	**2.722**
Eupodes	Lysigamasus	**0.005**	**0.407**	**2.245**	**2.722**
Eupodes	Parasitus	**0.005**	**0.859**	**2.245**	**2.546**
Eupodes	Uropoda	**0.005**	**0.481**	**2.245**	**2.245**
Eupodes	Aporcelaimellus	**0.005**	**0.548**	**2.245**	**5.216**
Eupodes	Dorylaimoidea	**0.005**	**-0.604**	**2.245**	**5.216**
Eupodes	Qudsianematidae	**0.005**	**-0.207**	**2.245**	**4.739**
Eupodes	Thornematidae	**0.005**	**-0.470**	**2.245**	**4.739**
Eupodes	Eupodes	**0.005**	**0.005**	**2.245**	**2.245**
Eupodes	Tarsonemus	**0.005**	**-0.701**	**2.245**	**2.722**
Tarsonemus	Bdella	**-0.701**	**0.816**	**2.722**	**2.245**
Tarsonemus	Dendrolaelaps	**-0.701**	**0.027**	**2.722**	**2.722**
Tarsonemus	Lysigamasus	**-0.701**	**0.407**	**2.722**	**2.722**
Tarsonemus	Parasitus	**-0.701**	**0.859**	**2.722**	**2.546**
Tarsonemus	Uropoda	**-0.701**	**0.481**	**2.722**	**2.245**
Tarsonemus	Aporcelaimellus	**-0.701**	**0.548**	**2.722**	**5.216**
Tarsonemus	Dorylaimoidea	**-0.701**	**-0.604**	**2.722**	**5.216**
Tarsonemus	Qudsianematidae	**-0.701**	**-0.207**	**2.722**	**4.739**
Tarsonemus	Thornematidae	**-0.701**	**-0.470**	**2.722**	**4.739**
Tarsonemus	Eupodes	**-0.701**	**0.005**	**2.722**	**2.245**
Tarsonemus	Tarsonemus	**-0.701**	**-0.701**	**2.722**	**2.722**

Resource	Consumer	Mres	Mconsumer	Nres	Nconsumer
Filenchus	Mononchidae	**-1.033**	**-0.827**	**5.114**	**4.415**
Filenchus	Mylonchulus	**-1.033**	**-0.005**	**5.114**	**4.415**
Filenchus	Alliphis	**-1.033**	**0.053**	**5.114**	**3.682**
Filenchus	Lysigamasus	**-1.033**	**0.407**	**5.114**	**2.837**
Filenchus	Parasitus	**-1.033**	**0.859**	**5.114**	**2.837**
Filenchus	Rhodacarellus	**-1.033**	**-0.310**	**5.114**	**2.837**
Filenchus	Rhodacarus	**-1.033**	**0.005**	**5.114**	**3.439**
Filenchus	Aporcelaimellus	**-1.033**	**0.548**	**5.114**	**4.893**
Filenchus	Dorylaimoidea	**-1.033**	**-0.604**	**5.114**	**5.319**
Filenchus	Eudorylaimus	**-1.033**	**-0.166**	**5.114**	**4.716**
Filenchus	Qudsianematidae	**-1.033**	**-0.207**	**5.114**	**4.893**
Filenchus	Thornia	**-1.033**	**0.263**	**5.114**	**4.415**
Filenchus	Eupodes	**-1.033**	**0.005**	**5.114**	**3.682**
Filenchus	Stigmaeidae	**-1.033**	**0.229**	**5.114**	**2.837**
Helicotylenchus	Mononchidae	**-0.792**	**-0.827**	**5.319**	**4.415**
Helicotylenchus	Mylonchulus	**-0.792**	**-0.005**	**5.319**	**4.415**
Helicotylenchus	Alliphis	**-0.792**	**0.053**	**5.319**	**3.682**
Helicotylenchus	Lysigamasus	**-0.792**	**0.407**	**5.319**	**2.837**
Helicotylenchus	Parasitus	**-0.792**	**0.859**	**5.319**	**2.837**
Helicotylenchus	Rhodacarellus	**-0.792**	**-0.310**	**5.319**	**2.837**
Helicotylenchus	Rhodacarus	**-0.792**	**0.005**	**5.319**	**3.439**
Helicotylenchus	Aporcelaimellus	**-0.792**	**0.548**	**5.319**	**4.893**
Helicotylenchus	Dorylaimoidea	**-0.792**	**-0.604**	**5.319**	**5.319**
Helicotylenchus	Eudorylaimus	**-0.792**	**-0.166**	**5.319**	**4.716**
Helicotylenchus	Qudsianematidae	**-0.792**	**-0.207**	**5.319**	**4.893**
Helicotylenchus	Thornia	**-0.792**	**0.263**	**5.319**	**4.415**
Helicotylenchus	Eupodes	**-0.792**	**0.005**	**5.319**	**3.682**
Helicotylenchus	Stigmaeidae	**-0.792**	**0.229**	**5.319**	**2.837**
Heterodera	Mononchidae	**-0.883**	**-0.827**	**4.716**	**4.415**
Heterodera	Mylonchulus	**-0.883**	**-0.005**	**4.716**	**4.415**
Heterodera	Alliphis	**-0.883**	**0.053**	**4.716**	**3.682**
Heterodera	Lysigamasus	**-0.883**	**0.407**	**4.716**	**2.837**
Heterodera	Parasitus	**-0.883**	**0.859**	**4.716**	**2.837**
Heterodera	Rhodacarellus	**-0.883**	**-0.310**	**4.716**	**2.837**
Heterodera	Rhodacarus	**-0.883**	**0.005**	**4.716**	**3.439**
Heterodera	Aporcelaimellus	**-0.883**	**0.548**	**4.716**	**4.893**
Heterodera	Dorylaimoidea	**-0.883**	**-0.604**	**4.716**	**5.319**
Heterodera	Eudorylaimus	**-0.883**	**-0.166**	**4.716**	**4.716**
Heterodera	Qudsianematidae	**-0.883**	**-0.207**	**4.716**	**4.893**
Heterodera	Thornia	**-0.883**	**0.263**	**4.716**	**4.415**
Heterodera	Eupodes	**-0.883**	**0.005**	**4.716**	**3.682**
Heterodera	Stigmaeidae	**-0.883**	**0.229**	**4.716**	**2.837**
Hoplolaimidae	Mononchidae	**-1.090**	**-0.827**	**4.415**	**4.415**
Hoplolaimidae	Mylonchulus	**-1.090**	**-0.005**	**4.415**	**4.415**
Hoplolaimidae	Alliphis	**-1.090**	**0.053**	**4.415**	**3.682**
Hoplolaimidae	Lysigamasus	**-1.090**	**0.407**	**4.415**	**2.837**
Hoplolaimidae	Parasitus	**-1.090**	**0.859**	**4.415**	**2.837**
Hoplolaimidae	Rhodacarellus	**-1.090**	**-0.310**	**4.415**	**2.837**
Hoplolaimidae	Rhodacarus	**-1.090**	**0.005**	**4.415**	**3.439**
Hoplolaimidae	Aporcelaimellus	**-1.090**	**0.548**	**4.415**	**4.893**

Hoplolaimidae	Dorylaimoidea	**-1.090**	**-0.604**	**4.415**	**5.319**
Hoplolaimidae	Eudorylaimus	**-1.090**	**-0.166**	**4.415**	**4.716**
Hoplolaimidae	Qudsianematidae	**-1.090**	**-0.207**	**4.415**	**4.893**
Hoplolaimidae	Thornia	**-1.090**	**0.263**	**4.415**	**4.415**
Hoplolaimidae	Eupodes	**-1.090**	**0.005**	**4.415**	**3.682**
Hoplolaimidae	Stigmaeidae	**-1.090**	**0.229**	**4.415**	**2.837**
Malenchus	Mononchidae	**-1.330**	**-0.827**	**4.415**	**4.415**
Malenchus	Mylonchulus	**-1.330**	**-0.005**	**4.415**	**4.415**
Malenchus	Alliphis	**-1.330**	**0.053**	**4.415**	**3.682**
Malenchus	Lysigamasus	**-1.330**	**0.407**	**4.415**	**2.837**
Malenchus	Parasitus	**-1.330**	**0.859**	**4.415**	**2.837**
Malenchus	Rhodacarellus	**-1.330**	**-0.310**	**4.415**	**2.837**
Malenchus	Rhodacarus	**-1.330**	**0.005**	**4.415**	**3.439**
Malenchus	Aporcelaimellus	**-1.330**	**0.548**	**4.415**	**4.893**
Malenchus	Dorylaimoidea	**-1.330**	**-0.604**	**4.415**	**5.319**
Malenchus	Eudorylaimus	**-1.330**	**-0.166**	**4.415**	**4.716**
Malenchus	Qudsianematidae	**-1.330**	**-0.207**	**4.415**	**4.893**
Malenchus	Thornia	**-1.330**	**0.263**	**4.415**	**4.415**
Malenchus	Eupodes	**-1.330**	**0.005**	**4.415**	**3.682**
Malenchus	Stigmaeidae	**-1.330**	**0.229**	**4.415**	**2.837**
Meloidogyne	Mononchidae	**-1.287**	**-0.827**	**4.415**	**4.415**
Meloidogyne	Mylonchulus	**-1.287**	**-0.005**	**4.415**	**4.415**
Meloidogyne	Alliphis	**-1.287**	**0.053**	**4.415**	**3.682**
Meloidogyne	Lysigamasus	**-1.287**	**0.407**	**4.415**	**2.837**
Meloidogyne	Parasitus	**-1.287**	**0.859**	**4.415**	**2.837**
Meloidogyne	Rhodacarellus	**-1.287**	**-0.310**	**4.415**	**2.837**
Meloidogyne	Rhodacarus	**-1.287**	**0.005**	**4.415**	**3.439**
Meloidogyne	Aporcelaimellus	**-1.287**	**0.548**	**4.415**	**4.893**
Meloidogyne	Dorylaimoidea	**-1.287**	**-0.604**	**4.415**	**5.319**
Meloidogyne	Eudorylaimus	**-1.287**	**-0.166**	**4.415**	**4.716**
Meloidogyne	Qudsianematidae	**-1.287**	**-0.207**	**4.415**	**4.893**
Meloidogyne	Thornia	**-1.287**	**0.263**	**4.415**	**4.415**
Meloidogyne	Eupodes	**-1.287**	**0.005**	**4.415**	**3.682**
Meloidogyne	Stigmaeidae	**-1.287**	**0.229**	**4.415**	**2.837**
Paratylenchus	Mononchidae	**-1.244**	**-0.827**	**4.415**	**4.415**
Paratylenchus	Mylonchulus	**-1.244**	**-0.005**	**4.415**	**4.415**
Paratylenchus	Alliphis	**-1.244**	**0.053**	**4.415**	**3.682**
Paratylenchus	Lysigamasus	**-1.244**	**0.407**	**4.415**	**2.837**
Paratylenchus	Parasitus	**-1.244**	**0.859**	**4.415**	**2.837**
Paratylenchus	Rhodacarellus	**-1.244**	**-0.310**	**4.415**	**2.837**
Paratylenchus	Rhodacarus	**-1.244**	**0.005**	**4.415**	**3.439**
Paratylenchus	Aporcelaimellus	**-1.244**	**0.548**	**4.415**	**4.893**
Paratylenchus	Dorylaimoidea	**-1.244**	**-0.604**	**4.415**	**5.319**
Paratylenchus	Eudorylaimus	**-1.244**	**-0.166**	**4.415**	**4.716**
Paratylenchus	Qudsianematidae	**-1.244**	**-0.207**	**4.415**	**4.893**
Paratylenchus	Thornia	**-1.244**	**0.263**	**4.415**	**4.415**
Paratylenchus	Eupodes	**-1.244**	**0.005**	**4.415**	**3.682**
Paratylenchus	Stigmaeidae	**-1.244**	**0.229**	**4.415**	**2.837**
Pratylenchus	Mononchidae	**-1.226**	**-0.827**	**4.893**	**4.415**
Pratylenchus	Mylonchulus	**-1.226**	**-0.005**	**4.893**	**4.415**
Pratylenchus	Alliphis	**-1.226**	**0.053**	**4.893**	**3.682**

Pratylenchus	Lysigamasus	**-1.226**	**0.407**	**4.893**	**2.837**
Pratylenchus	Parasitus	**-1.226**	**0.859**	**4.893**	**2.837**
Pratylenchus	Rhodacarellus	**-1.226**	**-0.310**	**4.893**	**2.837**
Pratylenchus	Rhodacarus	**-1.226**	**0.005**	**4.893**	**3.439**
Pratylenchus	Aporcelaimellus	**-1.226**	**0.548**	**4.893**	**4.893**
Pratylenchus	Dorylaimoidea	**-1.226**	**-0.604**	**4.893**	**5.319**
Pratylenchus	Eudorylaimus	**-1.226**	**-0.166**	**4.893**	**4.716**
Pratylenchus	Qudsianematidae	**-1.226**	**-0.207**	**4.893**	**4.893**
Pratylenchus	Thornia	**-1.226**	**0.263**	**4.893**	**4.415**
Pratylenchus	Eupodes	**-1.226**	**0.005**	**4.893**	**3.682**
Pratylenchus	Stigmaeidae	**-1.226**	**0.229**	**4.893**	**2.837**
Trichodorus	Mononchidae	**-0.744**	**-0.827**	**4.716**	**4.415**
Trichodorus	Mylonchulus	**-0.744**	**-0.005**	**4.716**	**4.415**
Trichodorus	Alliphis	**-0.744**	**0.053**	**4.716**	**3.682**
Trichodorus	Lysigamasus	**-0.744**	**0.407**	**4.716**	**2.837**
Trichodorus	Parasitus	**-0.744**	**0.859**	**4.716**	**2.837**
Trichodorus	Rhodacarellus	**-0.744**	**-0.310**	**4.716**	**2.837**
Trichodorus	Rhodacarus	**-0.744**	**0.005**	**4.716**	**3.439**
Trichodorus	Aporcelaimellus	**-0.744**	**0.548**	**4.716**	**4.893**
Trichodorus	Dorylaimoidea	**-0.744**	**-0.604**	**4.716**	**5.319**
Trichodorus	Eudorylaimus	**-0.744**	**-0.166**	**4.716**	**4.716**
Trichodorus	Qudsianematidae	**-0.744**	**-0.207**	**4.716**	**4.893**
Trichodorus	Thornia	**-0.744**	**0.263**	**4.716**	**4.415**
Trichodorus	Eupodes	**-0.744**	**0.005**	**4.716**	**3.682**
Trichodorus	Stigmaeidae	**-0.744**	**0.229**	**4.716**	**2.837**
Tylenchorhynchus	Mononchidae	**-0.664**	**-0.827**	**5.495**	**4.415**
Tylenchorhynchus	Mylonchulus	**-0.664**	**-0.005**	**5.495**	**4.415**
Tylenchorhynchus	Alliphis	**-0.664**	**0.053**	**5.495**	**3.682**
Tylenchorhynchus	Lysigamasus	**-0.664**	**0.407**	**5.495**	**2.837**
Tylenchorhynchus	Parasitus	**-0.664**	**0.859**	**5.495**	**2.837**
Tylenchorhynchus	Rhodacarellus	**-0.664**	**-0.310**	**5.495**	**2.837**
Tylenchorhynchus	Rhodacarus	**-0.664**	**0.005**	**5.495**	**3.439**
Tylenchorhynchus	Aporcelaimellus	**-0.664**	**0.548**	**5.495**	**4.893**
Tylenchorhynchus	Dorylaimoidea	**-0.664**	**-0.604**	**5.495**	**5.319**
Tylenchorhynchus	Eudorylaimus	**-0.664**	**-0.166**	**5.495**	**4.716**
Tylenchorhynchus	Qudsianematidae	**-0.664**	**-0.207**	**5.495**	**4.893**
Tylenchorhynchus	Thornia	**-0.664**	**0.263**	**5.495**	**4.415**
Tylenchorhynchus	Eupodes	**-0.664**	**0.005**	**5.495**	**3.682**
Tylenchorhynchus	Stigmaeidae	**-0.664**	**0.229**	**5.495**	**2.837**
Achipteria	Lysigamasus	**0.341**	**0.407**	**2.837**	**2.837**
Achipteria	Parasitus	**0.341**	**0.859**	**2.837**	**2.837**
Achipteria	Rhodacarellus	**0.341**	**-0.310**	**2.837**	**2.837**
Achipteria	Rhodacarus	**0.341**	**0.005**	**2.837**	**3.439**
Achipteria	Aporcelaimellus	**0.341**	**0.548**	**2.837**	**4.893**
Achipteria	Dorylaimoidea	**0.341**	**-0.604**	**2.837**	**5.319**
Achipteria	Eudorylaimus	**0.341**	**-0.166**	**2.837**	**4.716**
Achipteria	Qudsianematidae	**0.341**	**-0.207**	**2.837**	**4.893**
Achipteria	Thornia	**0.341**	**0.263**	**2.837**	**4.415**
Achipteria	Eupodes	**0.341**	**0.005**	**2.837**	**3.682**
Achipteria	Stigmaeidae	**0.341**	**0.229**	**2.837**	**2.837**
Tydeidae	Lysigamasus	**-0.608**	**0.407**	**3.536**	**2.837**

Tydeidae	Parasitus	**-0.608**	**0.859**	**3.536**	**2.837**
Tydeidae	Rhodacarellus	**-0.608**	**-0.310**	**3.536**	**2.837**
Tydeidae	Rhodacarus	**-0.608**	**0.005**	**3.536**	**3.439**
Tydeidae	Aporcelaimellus	**-0.608**	**0.548**	**3.536**	**4.893**
Tydeidae	Dorylaimoidea	**-0.608**	**-0.604**	**3.536**	**5.319**
Tydeidae	Eudorylaimus	**-0.608**	**-0.166**	**3.536**	**4.716**
Tydeidae	Qudsianematidae	**-0.608**	**-0.207**	**3.536**	**4.893**
Tydeidae	Thornia	**-0.608**	**0.263**	**3.536**	**4.415**
Tydeidae	Eupodes	**-0.608**	**0.005**	**3.536**	**3.682**
Tydeidae	Stigmaeidae	**-0.608**	**0.229**	**3.536**	**2.837**
Sminthuridae	Lysigamasus	**-0.608**	**0.407**	**3.439**	**2.837**
Sminthuridae	Parasitus	**-0.608**	**0.859**	**3.439**	**2.837**
Sminthuridae	Rhodacarellus	**-0.608**	**-0.310**	**3.439**	**2.837**
Sminthuridae	Rhodacarus	**-0.608**	**0.005**	**3.439**	**3.439**
Sminthuridae	Aporcelaimellus	**-0.608**	**0.548**	**3.439**	**4.893**
Sminthuridae	Dorylaimoidea	**-0.608**	**-0.604**	**3.439**	**5.319**
Sminthuridae	Eudorylaimus	**-0.608**	**-0.166**	**3.439**	**4.716**
Sminthuridae	Qudsianematidae	**-0.608**	**-0.207**	**3.439**	**4.893**
Sminthuridae	Thornia	**-0.608**	**0.263**	**3.439**	**4.415**
Sminthuridae	Eupodes	**-0.608**	**0.005**	**3.439**	**3.682**
Sminthuridae	Stigmaeidae	**-0.608**	**0.229**	**3.439**	**2.837**
Sminthurus	Lysigamasus	**1.429**	**0.407**	**2.837**	**2.837**
Sminthurus	Parasitus	**1.429**	**0.859**	**2.837**	**2.837**
Sminthurus	Rhodacarellus	**1.429**	**-0.310**	**2.837**	**2.837**
Sminthurus	Rhodacarus	**1.429**	**0.005**	**2.837**	**3.439**
Sminthurus	Aporcelaimellus	**1.429**	**0.548**	**2.837**	**4.893**
Sminthurus	Dorylaimoidea	**1.429**	**-0.604**	**2.837**	**5.319**
Sminthurus	Eudorylaimus	**1.429**	**-0.166**	**2.837**	**4.716**
Sminthurus	Qudsianematidae	**1.429**	**-0.207**	**2.837**	**4.893**
Sminthurus	Thornia	**1.429**	**0.263**	**2.837**	**4.415**
Sminthurus	Eupodes	**1.429**	**0.005**	**2.837**	**3.682**
Sminthurus	Stigmaeidae	**1.429**	**0.229**	**2.837**	**2.837**
Aphelenchoides	Mononchidae	**-1.496**	**-0.827**	**5.194**	**4.415**
Aphelenchoides	Mylonchulus	**-1.496**	**-0.005**	**5.194**	**4.415**
Aphelenchoides	Alliphis	**-1.496**	**0.053**	**5.194**	**3.682**
Aphelenchoides	Lysigamasus	**-1.496**	**0.407**	**5.194**	**2.837**
Aphelenchoides	Parasitus	**-1.496**	**0.859**	**5.194**	**2.837**
Aphelenchoides	Rhodacarellus	**-1.496**	**-0.310**	**5.194**	**2.837**
Aphelenchoides	Rhodacarus	**-1.496**	**0.005**	**5.194**	**3.439**
Aphelenchoides	Aporcelaimellus	**-1.496**	**0.548**	**5.194**	**4.893**
Aphelenchoides	Dorylaimoidea	**-1.496**	**-0.604**	**5.194**	**5.319**
Aphelenchoides	Eudorylaimus	**-1.496**	**-0.166**	**5.194**	**4.716**
Aphelenchoides	Qudsianematidae	**-1.496**	**-0.207**	**5.194**	**4.893**
Aphelenchoides	Thornia	**-1.496**	**0.263**	**5.194**	**4.415**
Aphelenchoides	Eupodes	**-1.496**	**0.005**	**5.194**	**3.682**
Aphelenchoides	Stigmaeidae	**-1.496**	**0.229**	**5.194**	**2.837**
Tylenchidae	Mononchidae	**-1.360**	**-0.827**	**4.716**	**4.415**
Tylenchidae	Mylonchulus	**-1.360**	**-0.005**	**4.716**	**4.415**
Tylenchidae	Alliphis	**-1.360**	**0.053**	**4.716**	**3.682**
Tylenchidae	Lysigamasus	**-1.360**	**0.407**	**4.716**	**2.837**
Tylenchidae	Parasitus	**-1.360**	**0.859**	**4.716**	**2.837**

Tylenchidae	Rhodacarellus	**-1.360**	**-0.310**	**4.716**	**2.837**
Tylenchidae	Rhodacarus	**-1.360**	**0.005**	**4.716**	**3.439**
Tylenchidae	Aporcelaimellus	**-1.360**	**0.548**	**4.716**	**4.893**
Tylenchidae	Dorylaimoidea	**-1.360**	**-0.604**	**4.716**	**5.319**
Tylenchidae	Eudorylaimus	**-1.360**	**-0.166**	**4.716**	**4.716**
Tylenchidae	Qudsianematidae	**-1.360**	**-0.207**	**4.716**	**4.893**
Tylenchidae	Thornia	**-1.360**	**0.263**	**4.716**	**4.415**
Tylenchidae	Eupodes	**-1.360**	**0.005**	**4.716**	**3.682**
Tylenchidae	Stigmaeidae	**-1.360**	**0.229**	**4.716**	**2.837**
Liebstadia	Lysigamasus	**0.270**	**0.407**	**2.837**	**2.837**
Liebstadia	Parasitus	**0.270**	**0.859**	**2.837**	**2.837**
Liebstadia	Rhodacarellus	**0.270**	**-0.310**	**2.837**	**2.837**
Liebstadia	Rhodacarus	**0.270**	**0.005**	**2.837**	**3.439**
Liebstadia	Aporcelaimellus	**0.270**	**0.548**	**2.837**	**4.893**
Liebstadia	Dorylaimoidea	**0.270**	**-0.604**	**2.837**	**5.319**
Liebstadia	Eudorylaimus	**0.270**	**-0.166**	**2.837**	**4.716**
Liebstadia	Qudsianematidae	**0.270**	**-0.207**	**2.837**	**4.893**
Liebstadia	Thornia	**0.270**	**0.263**	**2.837**	**4.415**
Liebstadia	Eupodes	**0.270**	**0.005**	**2.837**	**3.682**
Liebstadia	Stigmaeidae	**0.270**	**0.229**	**2.837**	**2.837**
Medioppia	Lysigamasus	**-0.235**	**0.407**	**3.314**	**2.837**
Medioppia	Parasitus	**-0.235**	**0.859**	**3.314**	**2.837**
Medioppia	Rhodacarellus	**-0.235**	**-0.310**	**3.314**	**2.837**
Medioppia	Rhodacarus	**-0.235**	**0.005**	**3.314**	**3.439**
Medioppia	Aporcelaimellus	**-0.235**	**0.548**	**3.314**	**4.893**
Medioppia	Dorylaimoidea	**-0.235**	**-0.604**	**3.314**	**5.319**
Medioppia	Eudorylaimus	**-0.235**	**-0.166**	**3.314**	**4.716**
Medioppia	Qudsianematidae	**-0.235**	**-0.207**	**3.314**	**4.893**
Medioppia	Thornia	**-0.235**	**0.263**	**3.314**	**4.415**
Medioppia	Eupodes	**-0.235**	**0.005**	**3.314**	**3.682**
Medioppia	Stigmaeidae	**-0.235**	**0.229**	**3.314**	**2.837**
Ceratophysella	Lysigamasus	**1.335**	**0.407**	**3.138**	**2.837**
Ceratophysella	Parasitus	**1.335**	**0.859**	**3.138**	**2.837**
Ceratophysella	Rhodacarellus	**1.335**	**-0.310**	**3.138**	**2.837**
Ceratophysella	Rhodacarus	**1.335**	**0.005**	**3.138**	**3.439**
Ceratophysella	Aporcelaimellus	**1.335**	**0.548**	**3.138**	**4.893**
Ceratophysella	Dorylaimoidea	**1.335**	**-0.604**	**3.138**	**5.319**
Ceratophysella	Eudorylaimus	**1.335**	**-0.166**	**3.138**	**4.716**
Ceratophysella	Qudsianematidae	**1.335**	**-0.207**	**3.138**	**4.893**
Ceratophysella	Thornia	**1.335**	**0.263**	**3.138**	**4.415**
Ceratophysella	Eupodes	**1.335**	**0.005**	**3.138**	**3.682**
Ceratophysella	Stigmaeidae	**1.335**	**0.229**	**3.138**	**2.837**
Folsomia	Lysigamasus	**0.900**	**0.407**	**2.837**	**2.837**
Folsomia	Parasitus	**0.900**	**0.859**	**2.837**	**2.837**
Folsomia	Rhodacarellus	**0.900**	**-0.310**	**2.837**	**2.837**
Folsomia	Rhodacarus	**0.900**	**0.005**	**2.837**	**3.439**
Folsomia	Aporcelaimellus	**0.900**	**0.548**	**2.837**	**4.893**
Folsomia	Dorylaimoidea	**0.900**	**-0.604**	**2.837**	**5.319**
Folsomia	Eudorylaimus	**0.900**	**-0.166**	**2.837**	**4.716**
Folsomia	Qudsianematidae	**0.900**	**-0.207**	**2.837**	**4.893**
Folsomia	Thornia	**0.900**	**0.263**	**2.837**	**4.415**

Folsomia	Eupodes	**0.900**	**0.005**	**2.837**	**3.682**
Folsomia	Stigmaeidae	**0.900**	**0.229**	**2.837**	**2.837**
Hypogastrura	Lysigamasus	**0.977**	**0.407**	**3.878**	**2.837**
Hypogastrura	Parasitus	**0.977**	**0.859**	**3.878**	**2.837**
Hypogastrura	Rhodacarellus	**0.977**	**-0.310**	**3.878**	**2.837**
Hypogastrura	Rhodacarus	**0.977**	**0.005**	**3.878**	**3.439**
Hypogastrura	Aporcelaimellus	**0.977**	**0.548**	**3.878**	**4.893**
Hypogastrura	Dorylaimoidea	**0.977**	**-0.604**	**3.878**	**5.319**
Hypogastrura	Eudorylaimus	**0.977**	**-0.166**	**3.878**	**4.716**
Hypogastrura	Qudsianematidae	**0.977**	**-0.207**	**3.878**	**4.893**
Hypogastrura	Thornia	**0.977**	**0.263**	**3.878**	**4.415**
Hypogastrura	Eupodes	**0.977**	**0.005**	**3.878**	**3.682**
Hypogastrura	Stigmaeidae	**0.977**	**0.229**	**3.878**	**2.837**
Isotoma	Lysigamasus	**1.898**	**0.407**	**4.067**	**2.837**
Isotoma	Parasitus	**1.898**	**0.859**	**4.067**	**2.837**
Isotoma	Rhodacarellus	**1.898**	**-0.310**	**4.067**	**2.837**
Isotoma	Rhodacarus	**1.898**	**0.005**	**4.067**	**3.439**
Isotoma	Aporcelaimellus	**1.898**	**0.548**	**4.067**	**4.893**
Isotoma	Dorylaimoidea	**1.898**	**-0.604**	**4.067**	**5.319**
Isotoma	Eudorylaimus	**1.898**	**-0.166**	**4.067**	**4.716**
Isotoma	Qudsianematidae	**1.898**	**-0.207**	**4.067**	**4.893**
Isotoma	Thornia	**1.898**	**0.263**	**4.067**	**4.415**
Isotoma	Eupodes	**1.898**	**0.005**	**4.067**	**3.682**
Isotoma	Stigmaeidae	**1.898**	**0.229**	**4.067**	**2.837**
Lepidocyrtus	Lysigamasus	**1.231**	**0.407**	**2.837**	**2.837**
Lepidocyrtus	Parasitus	**1.231**	**0.859**	**2.837**	**2.837**
Lepidocyrtus	Rhodacarellus	**1.231**	**-0.310**	**2.837**	**2.837**
Lepidocyrtus	Rhodacarus	**1.231**	**0.005**	**2.837**	**3.439**
Lepidocyrtus	Aporcelaimellus	**1.231**	**0.548**	**2.837**	**4.893**
Lepidocyrtus	Dorylaimoidea	**1.231**	**-0.604**	**2.837**	**5.319**
Lepidocyrtus	Eudorylaimus	**1.231**	**-0.166**	**2.837**	**4.716**
Lepidocyrtus	Qudsianematidae	**1.231**	**-0.207**	**2.837**	**4.893**
Lepidocyrtus	Thornia	**1.231**	**0.263**	**2.837**	**4.415**
Lepidocyrtus	Eupodes	**1.231**	**0.005**	**2.837**	**3.682**
Lepidocyrtus	Stigmaeidae	**1.231**	**0.229**	**2.837**	**2.837**
Proisotoma	Lysigamasus	**0.770**	**0.407**	**2.837**	**2.837**
Proisotoma	Parasitus	**0.770**	**0.859**	**2.837**	**2.837**
Proisotoma	Rhodacarellus	**0.770**	**-0.310**	**2.837**	**2.837**
Proisotoma	Rhodacarus	**0.770**	**0.005**	**2.837**	**3.439**
Proisotoma	Aporcelaimellus	**0.770**	**0.548**	**2.837**	**4.893**
Proisotoma	Dorylaimoidea	**0.770**	**-0.604**	**2.837**	**5.319**
Proisotoma	Eudorylaimus	**0.770**	**-0.166**	**2.837**	**4.716**
Proisotoma	Qudsianematidae	**0.770**	**-0.207**	**2.837**	**4.893**
Proisotoma	Thornia	**0.770**	**0.263**	**2.837**	**4.415**
Proisotoma	Eupodes	**0.770**	**0.005**	**2.837**	**3.682**
Proisotoma	Stigmaeidae	**0.770**	**0.229**	**2.837**	**2.837**
Achaeta	Lysigamasus	**1.589**	**0.407**	**3.980**	**2.837**
Achaeta	Parasitus	**1.589**	**0.859**	**3.980**	**2.837**
Achaeta	Rhodacarellus	**1.589**	**-0.310**	**3.980**	**2.837**
Achaeta	Rhodacarus	**1.589**	**0.005**	**3.980**	**3.439**
Achaeta	Aporcelaimellus	**1.589**	**0.548**	**3.980**	**4.893**

Achaeta	Dorylaimoidea	**1.589**	**-0.604**	**3.980**	**5.319**
Achaeta	Eudorylaimus	**1.589**	**-0.166**	**3.980**	**4.716**
Achaeta	Qudsianematidae	**1.589**	**-0.207**	**3.980**	**4.893**
Achaeta	Thornia	**1.589**	**0.263**	**3.980**	**4.415**
Achaeta	Eupodes	**1.589**	**0.005**	**3.980**	**3.682**
Achaeta	Stigmaeidae	**1.589**	**0.229**	**3.980**	**2.837**
Cognettia	Lysigamasus	**2.248**	**0.407**	**4.639**	**2.837**
Cognettia	Parasitus	**2.248**	**0.859**	**4.639**	**2.837**
Cognettia	Rhodacarellus	**2.248**	**-0.310**	**4.639**	**2.837**
Cognettia	Rhodacarus	**2.248**	**0.005**	**4.639**	**3.439**
Cognettia	Aporcelaimellus	**2.248**	**0.548**	**4.639**	**4.893**
Cognettia	Dorylaimoidea	**2.248**	**-0.604**	**4.639**	**5.319**
Cognettia	Eudorylaimus	**2.248**	**-0.166**	**4.639**	**4.716**
Cognettia	Qudsianematidae	**2.248**	**-0.207**	**4.639**	**4.893**
Cognettia	Thornia	**2.248**	**0.263**	**4.639**	**4.415**
Cognettia	Eupodes	**2.248**	**0.005**	**4.639**	**3.682**
Cognettia	Stigmaeidae	**2.248**	**0.229**	**4.639**	**2.837**
Fridericia	Lysigamasus	**3.083**	**0.407**	**3.891**	**2.837**
Fridericia	Parasitus	**3.083**	**0.859**	**3.891**	**2.837**
Fridericia	Rhodacarellus	**3.083**	**-0.310**	**3.891**	**2.837**
Fridericia	Rhodacarus	**3.083**	**0.005**	**3.891**	**3.439**
Fridericia	Aporcelaimellus	**3.083**	**0.548**	**3.891**	**4.893**
Fridericia	Dorylaimoidea	**3.083**	**-0.604**	**3.891**	**5.319**
Fridericia	Eudorylaimus	**3.083**	**-0.166**	**3.891**	**4.716**
Fridericia	Qudsianematidae	**3.083**	**-0.207**	**3.891**	**4.893**
Fridericia	Thornia	**3.083**	**0.263**	**3.891**	**4.415**
Fridericia	Eupodes	**3.083**	**0.005**	**3.891**	**3.682**
Fridericia	Stigmaeidae	**3.083**	**0.229**	**3.891**	**2.837**
Acrobeles	Mononchidae	**-0.721**	**-0.827**	**5.017**	**4.415**
Acrobeles	Mylonchulus	**-0.721**	**-0.005**	**5.017**	**4.415**
Acrobeles	Alliphis	**-0.721**	**0.053**	**5.017**	**3.682**
Acrobeles	Lysigamasus	**-0.721**	**0.407**	**5.017**	**2.837**
Acrobeles	Parasitus	**-0.721**	**0.859**	**5.017**	**2.837**
Acrobeles	Rhodacarellus	**-0.721**	**-0.310**	**5.017**	**2.837**
Acrobeles	Rhodacarus	**-0.721**	**0.005**	**5.017**	**3.439**
Acrobeles	Aporcelaimellus	**-0.721**	**0.548**	**5.017**	**4.893**
Acrobeles	Dorylaimoidea	**-0.721**	**-0.604**	**5.017**	**5.319**
Acrobeles	Eudorylaimus	**-0.721**	**-0.166**	**5.017**	**4.716**
Acrobeles	Qudsianematidae	**-0.721**	**-0.207**	**5.017**	**4.893**
Acrobeles	Thornia	**-0.721**	**0.263**	**5.017**	**4.415**
Acrobeles	Eupodes	**-0.721**	**0.005**	**5.017**	**3.682**
Acrobeles	Stigmaeidae	**-0.721**	**0.229**	**5.017**	**2.837**
Acrobeloides	Mononchidae	**-1.171**	**-0.827**	**4.415**	**4.415**
Acrobeloides	Mylonchulus	**-1.171**	**-0.005**	**4.415**	**4.415**
Acrobeloides	Alliphis	**-1.171**	**0.053**	**4.415**	**3.682**
Acrobeloides	Lysigamasus	**-1.171**	**0.407**	**4.415**	**2.837**
Acrobeloides	Parasitus	**-1.171**	**0.859**	**4.415**	**2.837**
Acrobeloides	Rhodacarellus	**-1.171**	**-0.310**	**4.415**	**2.837**
Acrobeloides	Rhodacarus	**-1.171**	**0.005**	**4.415**	**3.439**
Acrobeloides	Aporcelaimellus	**-1.171**	**0.548**	**4.415**	**4.893**
Acrobeloides	Dorylaimoidea	**-1.171**	**-0.604**	**4.415**	**5.319**

Acrobeloides	Eudorylaimus	**-1.171**	**-0.166**	**4.415**	**4.716**
Acrobeloides	Qudsianematidae	**-1.171**	**-0.207**	**4.415**	**4.893**
Acrobeloides	Thornia	**-1.171**	**0.263**	**4.415**	**4.415**
Acrobeloides	Eupodes	**-1.171**	**0.005**	**4.415**	**3.682**
Acrobeloides	Stigmaeidae	**-1.171**	**0.229**	**4.415**	**2.837**
Cephalobidae	Mononchidae	**-1.055**	**-0.827**	**4.415**	**4.415**
Cephalobidae	Mylonchulus	**-1.055**	**-0.005**	**4.415**	**4.415**
Cephalobidae	Alliphis	**-1.055**	**0.053**	**4.415**	**3.682**
Cephalobidae	Lysigamasus	**-1.055**	**0.407**	**4.415**	**2.837**
Cephalobidae	Parasitus	**-1.055**	**0.859**	**4.415**	**2.837**
Cephalobidae	Rhodacarellus	**-1.055**	**-0.310**	**4.415**	**2.837**
Cephalobidae	Rhodacarus	**-1.055**	**0.005**	**4.415**	**3.439**
Cephalobidae	Aporcelaimellus	**-1.055**	**0.548**	**4.415**	**4.893**
Cephalobidae	Dorylaimoidea	**-1.055**	**-0.604**	**4.415**	**5.319**
Cephalobidae	Eudorylaimus	**-1.055**	**-0.166**	**4.415**	**4.716**
Cephalobidae	Qudsianematidae	**-1.055**	**-0.207**	**4.415**	**4.893**
Cephalobidae	Thornia	**-1.055**	**0.263**	**4.415**	**4.415**
Cephalobidae	Eupodes	**-1.055**	**0.005**	**4.415**	**3.682**
Cephalobidae	Stigmaeidae	**-1.055**	**0.229**	**4.415**	**2.837**
Eucephalobus	Mononchidae	**-1.244**	**-0.827**	**5.529**	**4.415**
Eucephalobus	Mylonchulus	**-1.244**	**-0.005**	**5.529**	**4.415**
Eucephalobus	Alliphis	**-1.244**	**0.053**	**5.529**	**3.682**
Eucephalobus	Lysigamasus	**-1.244**	**0.407**	**5.529**	**2.837**
Eucephalobus	Parasitus	**-1.244**	**0.859**	**5.529**	**2.837**
Eucephalobus	Rhodacarellus	**-1.244**	**-0.310**	**5.529**	**2.837**
Eucephalobus	Rhodacarus	**-1.244**	**0.005**	**5.529**	**3.439**
Eucephalobus	Aporcelaimellus	**-1.244**	**0.548**	**5.529**	**4.893**
Eucephalobus	Dorylaimoidea	**-1.244**	**-0.604**	**5.529**	**5.319**
Eucephalobus	Eudorylaimus	**-1.244**	**-0.166**	**5.529**	**4.716**
Eucephalobus	Qudsianematidae	**-1.244**	**-0.207**	**5.529**	**4.893**
Eucephalobus	Thornia	**-1.244**	**0.263**	**5.529**	**4.415**
Eucephalobus	Eupodes	**-1.244**	**0.005**	**5.529**	**3.682**
Eucephalobus	Stigmaeidae	**-1.244**	**0.229**	**5.529**	**2.837**
Metateratocephalus	Mononchidae	**-1.506**	**-0.827**	**4.415**	**4.415**
Metateratocephalus	Mylonchulus	**-1.506**	**-0.005**	**4.415**	**4.415**
Metateratocephalus	Alliphis	**-1.506**	**0.053**	**4.415**	**3.682**
Metateratocephalus	Lysigamasus	**-1.506**	**0.407**	**4.415**	**2.837**
Metateratocephalus	Parasitus	**-1.506**	**0.859**	**4.415**	**2.837**
Metateratocephalus	Rhodacarellus	**-1.506**	**-0.310**	**4.415**	**2.837**
Metateratocephalus	Rhodacarus	**-1.506**	**0.005**	**4.415**	**3.439**
Metateratocephalus	Aporcelaimellus	**-1.506**	**0.548**	**4.415**	**4.893**
Metateratocephalus	Dorylaimoidea	**-1.506**	**-0.604**	**4.415**	**5.319**
Metateratocephalus	Eudorylaimus	**-1.506**	**-0.166**	**4.415**	**4.716**
Metateratocephalus	Qudsianematidae	**-1.506**	**-0.207**	**4.415**	**4.893**
Metateratocephalus	Thornia	**-1.506**	**0.263**	**4.415**	**4.415**
Metateratocephalus	Eupodes	**-1.506**	**0.005**	**4.415**	**3.682**
Metateratocephalus	Stigmaeidae	**-1.506**	**0.229**	**4.415**	**2.837**
Monhysteridae	Mononchidae	**-0.600**	**-0.827**	**4.415**	**4.415**
Monhysteridae	Mylonchulus	**-0.600**	**-0.005**	**4.415**	**4.415**
Monhysteridae	Alliphis	**-0.600**	**0.053**	**4.415**	**3.682**
Monhysteridae	Lysigamasus	**-0.600**	**0.407**	**4.415**	**2.837**

Monhysteridae	Parasitus	**-0.600**	**0.859**	**4.415**	**2.837**
Monhysteridae	Rhodacarellus	**-0.600**	**-0.310**	**4.415**	**2.837**
Monhysteridae	Rhodacarus	**-0.600**	**0.005**	**4.415**	**3.439**
Monhysteridae	Aporcelaimellus	**-0.600**	**0.548**	**4.415**	**4.893**
Monhysteridae	Dorylaimoidea	**-0.600**	**-0.604**	**4.415**	**5.319**
Monhysteridae	Eudorylaimus	**-0.600**	**-0.166**	**4.415**	**4.716**
Monhysteridae	Qudsianematidae	**-0.600**	**-0.207**	**4.415**	**4.893**
Monhysteridae	Thornia	**-0.600**	**0.263**	**4.415**	**4.415**
Monhysteridae	Eupodes	**-0.600**	**0.005**	**4.415**	**3.682**
Monhysteridae	Stigmaeidae	**-0.600**	**0.229**	**4.415**	**2.837**
Panagrolaimus	Mononchidae	**-0.945**	**-0.827**	**5.457**	**4.415**
Panagrolaimus	Mylonchulus	**-0.945**	**-0.005**	**5.457**	**4.415**
Panagrolaimus	Alliphis	**-0.945**	**0.053**	**5.457**	**3.682**
Panagrolaimus	Lysigamasus	**-0.945**	**0.407**	**5.457**	**2.837**
Panagrolaimus	Parasitus	**-0.945**	**0.859**	**5.457**	**2.837**
Panagrolaimus	Rhodacarellus	**-0.945**	**-0.310**	**5.457**	**2.837**
Panagrolaimus	Rhodacarus	**-0.945**	**0.005**	**5.457**	**3.439**
Panagrolaimus	Aporcelaimellus	**-0.945**	**0.548**	**5.457**	**4.893**
Panagrolaimus	Dorylaimoidea	**-0.945**	**-0.604**	**5.457**	**5.319**
Panagrolaimus	Eudorylaimus	**-0.945**	**-0.166**	**5.457**	**4.716**
Panagrolaimus	Qudsianematidae	**-0.945**	**-0.207**	**5.457**	**4.893**
Panagrolaimus	Thornia	**-0.945**	**0.263**	**5.457**	**4.415**
Panagrolaimus	Eupodes	**-0.945**	**0.005**	**5.457**	**3.682**
Panagrolaimus	Stigmaeidae	**-0.945**	**0.229**	**5.457**	**2.837**
Plectus	Mononchidae	**-0.583**	**-0.827**	**4.893**	**4.415**
Plectus	Mylonchulus	**-0.583**	**-0.005**	**4.893**	**4.415**
Plectus	Alliphis	**-0.583**	**0.053**	**4.893**	**3.682**
Plectus	Lysigamasus	**-0.583**	**0.407**	**4.893**	**2.837**
Plectus	Parasitus	**-0.583**	**0.859**	**4.893**	**2.837**
Plectus	Rhodacarellus	**-0.583**	**-0.310**	**4.893**	**2.837**
Plectus	Rhodacarus	**-0.583**	**0.005**	**4.893**	**3.439**
Plectus	Aporcelaimellus	**-0.583**	**0.548**	**4.893**	**4.893**
Plectus	Dorylaimoidea	**-0.583**	**-0.604**	**4.893**	**5.319**
Plectus	Eudorylaimus	**-0.583**	**-0.166**	**4.893**	**4.716**
Plectus	Qudsianematidae	**-0.583**	**-0.207**	**4.893**	**4.893**
Plectus	Thornia	**-0.583**	**0.263**	**4.893**	**4.415**
Plectus	Eupodes	**-0.583**	**0.005**	**4.893**	**3.682**
Plectus	Stigmaeidae	**-0.583**	**0.229**	**4.893**	**2.837**
Rhabditidae	Mononchidae	**-0.692**	**-0.827**	**5.777**	**4.415**
Rhabditidae	Mylonchulus	**-0.692**	**-0.005**	**5.777**	**4.415**
Rhabditidae	Alliphis	**-0.692**	**0.053**	**5.777**	**3.682**
Rhabditidae	Lysigamasus	**-0.692**	**0.407**	**5.777**	**2.837**
Rhabditidae	Parasitus	**-0.692**	**0.859**	**5.777**	**2.837**
Rhabditidae	Rhodacarellus	**-0.692**	**-0.310**	**5.777**	**2.837**
Rhabditidae	Rhodacarus	**-0.692**	**0.005**	**5.777**	**3.439**
Rhabditidae	Aporcelaimellus	**-0.692**	**0.548**	**5.777**	**4.893**
Rhabditidae	Dorylaimoidea	**-0.692**	**-0.604**	**5.777**	**5.319**
Rhabditidae	Eudorylaimus	**-0.692**	**-0.166**	**5.777**	**4.716**
Rhabditidae	Qudsianematidae	**-0.692**	**-0.207**	**5.777**	**4.893**
Rhabditidae	Thornia	**-0.692**	**0.263**	**5.777**	**4.415**
Rhabditidae	Eupodes	**-0.692**	**0.005**	**5.777**	**3.682**

Rhabditidae	Stigmaeidae	**-0.692**	**0.229**	**5.777**	**2.837**
Teratocephalus	Mononchidae	**-1.630**	**-0.827**	**5.114**	**4.415**
Teratocephalus	Mylonchulus	**-1.630**	**-0.005**	**5.114**	**4.415**
Teratocephalus	Alliphis	**-1.630**	**0.053**	**5.114**	**3.682**
Teratocephalus	Lysigamasus	**-1.630**	**0.407**	**5.114**	**2.837**
Teratocephalus	Parasitus	**-1.630**	**0.859**	**5.114**	**2.837**
Teratocephalus	Rhodacarellus	**-1.630**	**-0.310**	**5.114**	**2.837**
Teratocephalus	Rhodacarus	**-1.630**	**0.005**	**5.114**	**3.439**
Teratocephalus	Aporcelaimellus	**-1.630**	**0.548**	**5.114**	**4.893**
Teratocephalus	Dorylaimoidea	**-1.630**	**-0.604**	**5.114**	**5.319**
Teratocephalus	Eudorylaimus	**-1.630**	**-0.166**	**5.114**	**4.716**
Teratocephalus	Qudsianematidae	**-1.630**	**-0.207**	**5.114**	**4.893**
Teratocephalus	Thornia	**-1.630**	**0.263**	**5.114**	**4.415**
Teratocephalus	Eupodes	**-1.630**	**0.005**	**5.114**	**3.682**
Teratocephalus	Stigmaeidae	**-1.630**	**0.229**	**5.114**	**2.837**
Enchytraeus	Lysigamasus	**1.974**	**0.407**	**4.304**	**2.837**
Enchytraeus	Parasitus	**1.974**	**0.859**	**4.304**	**2.837**
Enchytraeus	Rhodacarellus	**1.974**	**-0.310**	**4.304**	**2.837**
Enchytraeus	Rhodacarus	**1.974**	**0.005**	**4.304**	**3.439**
Enchytraeus	Aporcelaimellus	**1.974**	**0.548**	**4.304**	**4.893**
Enchytraeus	Dorylaimoidea	**1.974**	**-0.604**	**4.304**	**5.319**
Enchytraeus	Eudorylaimus	**1.974**	**-0.166**	**4.304**	**4.716**
Enchytraeus	Qudsianematidae	**1.974**	**-0.207**	**4.304**	**4.893**
Enchytraeus	Thornia	**1.974**	**0.263**	**4.304**	**4.415**
Enchytraeus	Eupodes	**1.974**	**0.005**	**4.304**	**3.682**
Enchytraeus	Stigmaeidae	**1.974**	**0.229**	**4.304**	**2.837**
Eubacteria	Acrobeles	**-6.568**	**-0.721**	**13.066**	**5.017**
Eubacteria	Acrobeloides	**-6.568**	**-1.171**	**13.066**	**4.415**
Eubacteria	Cephalobidae	**-6.568**	**-1.055**	**13.066**	**4.415**
Eubacteria	Eucephalobus	**-6.568**	**-1.244**	**13.066**	**5.529**
Eubacteria	Metateratocephalus	**-6.568**	**-1.506**	**13.066**	**4.415**
Eubacteria	Monhysteridae	**-6.568**	**-0.600**	**13.066**	**4.415**
Eubacteria	Panagrolaimus	**-6.568**	**-0.945**	**13.066**	**5.457**
Eubacteria	Plectus	**-6.568**	**-0.583**	**13.066**	**4.893**
Eubacteria	Rhabditidae	**-6.568**	**-0.692**	**13.066**	**5.777**
Eubacteria	Teratocephalus	**-6.568**	**-1.630**	**13.066**	**5.114**
Eubacteria	Enchytraeus	**-6.568**	**1.974**	**13.066**	**4.304**
Eubacteria	Dauerlarvae	**-6.568**	**-0.804**	**13.066**	**5.646**
Eubacteria	Marionina	**-6.568**	**1.493**	**13.066**	**4.722**
Dauerlarvae	Mononchidae	**-0.804**	**-0.827**	**5.646**	**4.415**
Dauerlarvae	Mylonchulus	**-0.804**	**-0.005**	**5.646**	**4.415**
Dauerlarvae	Alliphis	**-0.804**	**0.053**	**5.646**	**3.682**
Dauerlarvae	Aporcelaimellus	**-0.804**	**0.548**	**5.646**	**4.893**
Dauerlarvae	Dorylaimoidea	**-0.804**	**-0.604**	**5.646**	**5.319**
Dauerlarvae	Eudorylaimus	**-0.804**	**-0.166**	**5.646**	**4.716**
Dauerlarvae	Qudsianematidae	**-0.804**	**-0.207**	**5.646**	**4.893**
Dauerlarvae	Thornia	**-0.804**	**0.263**	**5.646**	**4.415**
Dauerlarvae	Eupodes	**-0.804**	**0.005**	**5.646**	**3.682**
Dauerlarvae	Stigmaeidae	**-0.804**	**0.229**	**5.646**	**2.837**
Marionina	Lysigamasus	**1.493**	**0.407**	**4.722**	**2.837**
Marionina	Parasitus	**1.493**	**0.859**	**4.722**	**2.837**

Marionina	Rhodacarellus	**1.493**	**-0.310**	**4.722**	**2.837**
Marionina	Rhodacarus	**1.493**	**0.005**	**4.722**	**3.439**
Marionina	Aporcelaimellus	**1.493**	**0.548**	**4.722**	**4.893**
Marionina	Dorylaimoidea	**1.493**	**-0.604**	**4.722**	**5.319**
Marionina	Eudorylaimus	**1.493**	**-0.166**	**4.722**	**4.716**
Marionina	Qudsianematidae	**1.493**	**-0.207**	**4.722**	**4.893**
Marionina	Thornia	**1.493**	**0.263**	**4.722**	**4.415**
Marionina	Eupodes	**1.493**	**0.005**	**4.722**	**3.682**
Marionina	Stigmaeidae	**1.493**	**0.229**	**4.722**	**2.837**
Hyphae and hair roots	Filenchus	**7.082**	**-1.033**	**0.000**	**5.114**
Hyphae and hair roots	Helicotylenchus	**7.082**	**-0.792**	**0.000**	**5.319**
Hyphae and hair roots	Heterodera	**7.082**	**-0.883**	**0.000**	**4.716**
Hyphae and hair roots	Hoplolaimidae	**7.082**	**-1.090**	**0.000**	**4.415**
Hyphae and hair roots	Malenchus	**7.082**	**-1.330**	**0.000**	**4.415**
Hyphae and hair roots	Meloidogyne	**7.082**	**-1.287**	**0.000**	**4.415**
Hyphae and hair roots	Paratylenchus	**7.082**	**-1.244**	**0.000**	**4.415**
Hyphae and hair roots	Pratylenchus	**7.082**	**-1.226**	**0.000**	**4.893**
Hyphae and hair roots	Trichodorus	**7.082**	**-0.744**	**0.000**	**4.716**
Hyphae and hair roots	Tylenchorhynchus	**7.082**	**-0.664**	**0.000**	**5.495**
Hyphae and hair roots	Achipteria	**7.082**	**0.341**	**0.000**	**2.837**
Hyphae and hair roots	Tydeidae	**7.082**	**-0.608**	**0.000**	**3.536**
Hyphae and hair roots	Sminthuridae	**7.082**	**-0.608**	**0.000**	**3.439**
Hyphae and hair roots	Sminthurus	**7.082**	**1.429**	**0.000**	**2.837**
Hyphae and hair roots	Aphelenchoides	**7.082**	**-1.496**	**0.000**	**5.194**
Hyphae and hair roots	Tylenchidae	**7.082**	**-1.360**	**0.000**	**4.716**
Hyphae and hair roots	Liebstadia	**7.082**	**0.270**	**0.000**	**2.837**
Hyphae and hair roots	Medioppia	**7.082**	**-0.235**	**0.000**	**3.314**
Hyphae and hair roots	Ceratophysella	**7.082**	**1.335**	**0.000**	**3.138**
Hyphae and hair roots	Folsomia	**7.082**	**0.900**	**0.000**	**2.837**
Hyphae and hair roots	Hypogastrura	**7.082**	**0.977**	**0.000**	**3.878**
Hyphae and hair roots	Isotoma	**7.082**	**1.898**	**0.000**	**4.067**
Hyphae and hair roots	Lepidocyrtus	**7.082**	**1.231**	**0.000**	**2.837**
Hyphae and hair roots	Proisotoma	**7.082**	**0.770**	**0.000**	**2.837**
Hyphae and hair roots	Achaeta	**7.082**	**1.589**	**0.000**	**3.980**
Hyphae and hair roots	Cognettia	**7.082**	**2.248**	**0.000**	**4.639**
Hyphae and hair roots	Fridericia	**7.082**	**3.083**	**0.000**	**3.891**
Hyphae and hair roots	Aporcelaimellus	**7.082**	**0.548**	**0.000**	**4.893**
Hyphae and hair roots	Dorylaimoidea	**7.082**	**-0.604**	**0.000**	**5.319**
Hyphae and hair roots	Eudorylaimus	**7.082**	**-0.166**	**0.000**	**4.716**
Hyphae and hair roots	Qudsianematidae	**7.082**	**-0.207**	**0.000**	**4.893**
Hyphae and hair roots	Thornia	**7.082**	**0.263**	**0.000**	**4.415**
Hyphae and hair roots	Eupodes	**7.082**	**0.005**	**0.000**	**3.682**
Hyphae and hair roots	Stigmaeidae	**7.082**	**0.229**	**0.000**	**2.837**
Mononchidae	Lysigamasus	**-0.827**	**0.407**	**4.415**	**2.837**
Mononchidae	Parasitus	**-0.827**	**0.859**	**4.415**	**2.837**
Mononchidae	Rhodacarellus	**-0.827**	**-0.310**	**4.415**	**2.837**
Mononchidae	Rhodacarus	**-0.827**	**0.005**	**4.415**	**3.439**
Mononchidae	Aporcelaimellus	**-0.827**	**0.548**	**4.415**	**4.893**
Mononchidae	Dorylaimoidea	**-0.827**	**-0.604**	**4.415**	**5.319**
Mononchidae	Eudorylaimus	**-0.827**	**-0.166**	**4.415**	**4.716**
Mononchidae	Qudsianematidae	**-0.827**	**-0.207**	**4.415**	**4.893**

Mononchidae	Thornia	**-0.827**	**0.263**	**4.415**	**4.415**
Mononchidae	Eupodes	**-0.827**	**0.005**	**4.415**	**3.682**
Mononchidae	Stigmaeidae	**-0.827**	**0.229**	**4.415**	**2.837**
Mylonchulus	Lysigamasus	**-0.005**	**0.407**	**4.415**	**2.837**
Mylonchulus	Parasitus	**-0.005**	**0.859**	**4.415**	**2.837**
Mylonchulus	Rhodacarellus	**-0.005**	**-0.310**	**4.415**	**2.837**
Mylonchulus	Rhodacarus	**-0.005**	**0.005**	**4.415**	**3.439**
Mylonchulus	Aporcelaimellus	**-0.005**	**0.548**	**4.415**	**4.893**
Mylonchulus	Dorylaimoidea	**-0.005**	**-0.604**	**4.415**	**5.319**
Mylonchulus	Eudorylaimus	**-0.005**	**-0.166**	**4.415**	**4.716**
Mylonchulus	Qudsianematidae	**-0.005**	**-0.207**	**4.415**	**4.893**
Mylonchulus	Thornia	**-0.005**	**0.263**	**4.415**	**4.415**
Mylonchulus	Eupodes	**-0.005**	**0.005**	**4.415**	**3.682**
Mylonchulus	Stigmaeidae	**-0.005**	**0.229**	**4.415**	**2.837**
Alliphis	Lysigamasus	**0.053**	**0.407**	**3.682**	**2.837**
Alliphis	Parasitus	**0.053**	**0.859**	**3.682**	**2.837**
Alliphis	Rhodacarellus	**0.053**	**-0.310**	**3.682**	**2.837**
Alliphis	Rhodacarus	**0.053**	**0.005**	**3.682**	**3.439**
Alliphis	Aporcelaimellus	**0.053**	**0.548**	**3.682**	**4.893**
Alliphis	Dorylaimoidea	**0.053**	**-0.604**	**3.682**	**5.319**
Alliphis	Eudorylaimus	**0.053**	**-0.166**	**3.682**	**4.716**
Alliphis	Qudsianematidae	**0.053**	**-0.207**	**3.682**	**4.893**
Alliphis	Thornia	**0.053**	**0.263**	**3.682**	**4.415**
Alliphis	Eupodes	**0.053**	**0.005**	**3.682**	**3.682**
Alliphis	Stigmaeidae	**0.053**	**0.229**	**3.682**	**2.837**
Lysigamasus	Aporcelaimellus	**0.407**	**0.548**	**2.837**	**4.893**
Lysigamasus	Dorylaimoidea	**0.407**	**-0.604**	**2.837**	**5.319**
Lysigamasus	Eudorylaimus	**0.407**	**-0.166**	**2.837**	**4.716**
Lysigamasus	Qudsianematidae	**0.407**	**-0.207**	**2.837**	**4.893**
Lysigamasus	Thornia	**0.407**	**0.263**	**2.837**	**4.415**
Lysigamasus	Eupodes	**0.407**	**0.005**	**2.837**	**3.682**
Lysigamasus	Stigmaeidae	**0.407**	**0.229**	**2.837**	**2.837**
Parasitus	Aporcelaimellus	**0.859**	**0.548**	**2.837**	**4.893**
Parasitus	Dorylaimoidea	**0.859**	**-0.604**	**2.837**	**5.319**
Parasitus	Eudorylaimus	**0.859**	**-0.166**	**2.837**	**4.716**
Parasitus	Qudsianematidae	**0.859**	**-0.207**	**2.837**	**4.893**
Parasitus	Thornia	**0.859**	**0.263**	**2.837**	**4.415**
Parasitus	Eupodes	**0.859**	**0.005**	**2.837**	**3.682**
Parasitus	Stigmaeidae	**0.859**	**0.229**	**2.837**	**2.837**
Rhodacarellus	Aporcelaimellus	**-0.310**	**0.548**	**2.837**	**4.893**
Rhodacarellus	Dorylaimoidea	**-0.310**	**-0.604**	**2.837**	**5.319**
Rhodacarellus	Eudorylaimus	**-0.310**	**-0.166**	**2.837**	**4.716**
Rhodacarellus	Qudsianematidae	**-0.310**	**-0.207**	**2.837**	**4.893**
Rhodacarellus	Thornia	**-0.310**	**0.263**	**2.837**	**4.415**
Rhodacarellus	Eupodes	**-0.310**	**0.005**	**2.837**	**3.682**
Rhodacarellus	Stigmaeidae	**-0.310**	**0.229**	**2.837**	**2.837**
Rhodacarus	Aporcelaimellus	**0.005**	**0.548**	**3.439**	**4.893**
Rhodacarus	Dorylaimoidea	**0.005**	**-0.604**	**3.439**	**5.319**
Rhodacarus	Eudorylaimus	**0.005**	**-0.166**	**3.439**	**4.716**
Rhodacarus	Qudsianematidae	**0.005**	**-0.207**	**3.439**	**4.893**
Rhodacarus	Thornia	**0.005**	**0.263**	**3.439**	**4.415**

Rhodacarus	Eupodes	**0.005**	**0.005**	**3.439**	**3.682**
Rhodacarus	Stigmaeidae	**0.005**	**0.229**	**3.439**	**2.837**
Aporcelaimellus	Mononchidae	**0.548**	**-0.827**	**4.893**	**4.415**
Aporcelaimellus	Mylonchulus	**0.548**	**-0.005**	**4.893**	**4.415**
Aporcelaimellus	Alliphis	**0.548**	**0.053**	**4.893**	**3.682**
Aporcelaimellus	Lysigamasus	**0.548**	**0.407**	**4.893**	**2.837**
Aporcelaimellus	Parasitus	**0.548**	**0.859**	**4.893**	**2.837**
Aporcelaimellus	Rhodacarellus	**0.548**	**-0.310**	**4.893**	**2.837**
Aporcelaimellus	Rhodacarus	**0.548**	**0.005**	**4.893**	**3.439**
Aporcelaimellus	Aporcelaimellus	**0.548**	**0.548**	**4.893**	**4.893**
Aporcelaimellus	Dorylaimoidea	**0.548**	**-0.604**	**4.893**	**5.319**
Aporcelaimellus	Eudorylaimus	**0.548**	**-0.166**	**4.893**	**4.716**
Aporcelaimellus	Qudsianematidae	**0.548**	**-0.207**	**4.893**	**4.893**
Aporcelaimellus	Thornia	**0.548**	**0.263**	**4.893**	**4.415**
Aporcelaimellus	Eupodes	**0.548**	**0.005**	**4.893**	**3.682**
Aporcelaimellus	Stigmaeidae	**0.548**	**0.229**	**4.893**	**2.837**
Dorylaimoidea	Mononchidae	**-0.604**	**-0.827**	**5.319**	**4.415**
Dorylaimoidea	Mylonchulus	**-0.604**	**-0.005**	**5.319**	**4.415**
Dorylaimoidea	Alliphis	**-0.604**	**0.053**	**5.319**	**3.682**
Dorylaimoidea	Lysigamasus	**-0.604**	**0.407**	**5.319**	**2.837**
Dorylaimoidea	Parasitus	**-0.604**	**0.859**	**5.319**	**2.837**
Dorylaimoidea	Rhodacarellus	**-0.604**	**-0.310**	**5.319**	**2.837**
Dorylaimoidea	Rhodacarus	**-0.604**	**0.005**	**5.319**	**3.439**
Dorylaimoidea	Aporcelaimellus	**-0.604**	**0.548**	**5.319**	**4.893**
Dorylaimoidea	Dorylaimoidea	**-0.604**	**-0.604**	**5.319**	**5.319**
Dorylaimoidea	Eudorylaimus	**-0.604**	**-0.166**	**5.319**	**4.716**
Dorylaimoidea	Qudsianematidae	**-0.604**	**-0.207**	**5.319**	**4.893**
Dorylaimoidea	Thornia	**-0.604**	**0.263**	**5.319**	**4.415**
Dorylaimoidea	Eupodes	**-0.604**	**0.005**	**5.319**	**3.682**
Dorylaimoidea	Stigmaeidae	**-0.604**	**0.229**	**5.319**	**2.837**
Eudorylaimus	Mononchidae	**-0.166**	**-0.827**	**4.716**	**4.415**
Eudorylaimus	Mylonchulus	**-0.166**	**-0.005**	**4.716**	**4.415**
Eudorylaimus	Alliphis	**-0.166**	**0.053**	**4.716**	**3.682**
Eudorylaimus	Lysigamasus	**-0.166**	**0.407**	**4.716**	**2.837**
Eudorylaimus	Parasitus	**-0.166**	**0.859**	**4.716**	**2.837**
Eudorylaimus	Rhodacarellus	**-0.166**	**-0.310**	**4.716**	**2.837**
Eudorylaimus	Rhodacarus	**-0.166**	**0.005**	**4.716**	**3.439**
Eudorylaimus	Aporcelaimellus	**-0.166**	**0.548**	**4.716**	**4.893**
Eudorylaimus	Dorylaimoidea	**-0.166**	**-0.604**	**4.716**	**5.319**
Eudorylaimus	Eudorylaimus	**-0.166**	**-0.166**	**4.716**	**4.716**
Eudorylaimus	Qudsianematidae	**-0.166**	**-0.207**	**4.716**	**4.893**
Eudorylaimus	Thornia	**-0.166**	**0.263**	**4.716**	**4.415**
Eudorylaimus	Eupodes	**-0.166**	**0.005**	**4.716**	**3.682**
Eudorylaimus	Stigmaeidae	**-0.166**	**0.229**	**4.716**	**2.837**
Qudsianematidae	Mononchidae	**-0.207**	**-0.827**	**4.893**	**4.415**
Qudsianematidae	Mylonchulus	**-0.207**	**-0.005**	**4.893**	**4.415**
Qudsianematidae	Alliphis	**-0.207**	**0.053**	**4.893**	**3.682**
Qudsianematidae	Lysigamasus	**-0.207**	**0.407**	**4.893**	**2.837**
Qudsianematidae	Parasitus	**-0.207**	**0.859**	**4.893**	**2.837**
Qudsianematidae	Rhodacarellus	**-0.207**	**-0.310**	**4.893**	**2.837**
Qudsianematidae	Rhodacarus	**-0.207**	**0.005**	**4.893**	**3.439**

Qudsianematidae	Aporcelaimellus	**-0.207**	**0.548**	**4.893**	**4.893**
Qudsianematidae	Dorylaimoidea	**-0.207**	**-0.604**	**4.893**	**5.319**
Qudsianematidae	Eudorylaimus	**-0.207**	**-0.166**	**4.893**	**4.716**
Qudsianematidae	Qudsianematidae	**-0.207**	**-0.207**	**4.893**	**4.893**
Qudsianematidae	Thornia	**-0.207**	**0.263**	**4.893**	**4.415**
Qudsianematidae	Eupodes	**-0.207**	**0.005**	**4.893**	**3.682**
Qudsianematidae	Stigmaeidae	**-0.207**	**0.229**	**4.893**	**2.837**
Thornia	Mononchidae	**0.263**	**-0.827**	**4.415**	**4.415**
Thornia	Mylonchulus	**0.263**	**-0.005**	**4.415**	**4.415**
Thornia	Alliphis	**0.263**	**0.053**	**4.415**	**3.682**
Thornia	Lysigamasus	**0.263**	**0.407**	**4.415**	**2.837**
Thornia	Parasitus	**0.263**	**0.859**	**4.415**	**2.837**
Thornia	Rhodacarellus	**0.263**	**-0.310**	**4.415**	**2.837**
Thornia	Rhodacarus	**0.263**	**0.005**	**4.415**	**3.439**
Thornia	Aporcelaimellus	**0.263**	**0.548**	**4.415**	**4.893**
Thornia	Dorylaimoidea	**0.263**	**-0.604**	**4.415**	**5.319**
Thornia	Eudorylaimus	**0.263**	**-0.166**	**4.415**	**4.716**
Thornia	Qudsianematidae	**0.263**	**-0.207**	**4.415**	**4.893**
Thornia	Thornia	**0.263**	**0.263**	**4.415**	**4.415**
Thornia	Eupodes	**0.263**	**0.005**	**4.415**	**3.682**
Thornia	Stigmaeidae	**0.263**	**0.229**	**4.415**	**2.837**
Eupodes	Lysigamasus	**0.005**	**0.407**	**3.682**	**2.837**
Eupodes	Parasitus	**0.005**	**0.859**	**3.682**	**2.837**
Eupodes	Rhodacarellus	**0.005**	**-0.310**	**3.682**	**2.837**
Eupodes	Rhodacarus	**0.005**	**0.005**	**3.682**	**3.439**
Eupodes	Aporcelaimellus	**0.005**	**0.548**	**3.682**	**4.893**
Eupodes	Dorylaimoidea	**0.005**	**-0.604**	**3.682**	**5.319**
Eupodes	Eudorylaimus	**0.005**	**-0.166**	**3.682**	**4.716**
Eupodes	Qudsianematidae	**0.005**	**-0.207**	**3.682**	**4.893**
Eupodes	Thornia	**0.005**	**0.263**	**3.682**	**4.415**
Eupodes	Eupodes	**0.005**	**0.005**	**3.682**	**3.682**
Eupodes	Stigmaeidae	**0.005**	**0.229**	**3.682**	**2.837**
Stigmaeidae	Lysigamasus	**0.229**	**0.407**	**2.837**	**2.837**
Stigmaeidae	Parasitus	**0.229**	**0.859**	**2.837**	**2.837**
Stigmaeidae	Rhodacarellus	**0.229**	**-0.310**	**2.837**	**2.837**
Stigmaeidae	Rhodacarus	**0.229**	**0.005**	**2.837**	**3.439**
Stigmaeidae	Aporcelaimellus	**0.229**	**0.548**	**2.837**	**4.893**
Stigmaeidae	Dorylaimoidea	**0.229**	**-0.604**	**2.837**	**5.319**
Stigmaeidae	Eudorylaimus	**0.229**	**-0.166**	**2.837**	**4.716**
Stigmaeidae	Qudsianematidae	**0.229**	**-0.207**	**2.837**	**4.893**
Stigmaeidae	Thornia	**0.229**	**0.263**	**2.837**	**4.415**
Stigmaeidae	Eupodes	**0.229**	**0.005**	**2.837**	**3.682**
Stigmaeidae	Stigmaeidae	**0.229**	**0.229**	**2.837**	**2.837**

Resource	Consumer	Mres	Mconsumer	Nres	Nconsumer
Aglenchus	Mononchus	**-1.053**	**-0.938**	**5.600**	**4.558**
Aglenchus	Mylonchulus	**-1.053**	**-0.005**	**5.600**	**4.558**
Aglenchus	Tripyla	**-1.053**	**-0.420**	**5.600**	**5.035**
Aglenchus	Cheiroseius	**-1.053**	**0.356**	**5.600**	**2.371**
Aglenchus	Dendrolaelaps	**-1.053**	**0.027**	**5.600**	**2.371**
Aglenchus	Lysigamasus	**-1.053**	**0.407**	**5.600**	**2.371**
Aglenchus	Pachylaelaps	**-1.053**	**0.375**	**5.600**	**2.371**
Aglenchus	Veigaia	**-1.053**	**0.613**	**5.600**	**2.371**
Aglenchus	Aporcelaimellus	**-1.053**	**0.548**	**5.600**	**5.257**
Aglenchus	Dorylaimoidea	**-1.053**	**-0.604**	**5.600**	**5.160**
Aglenchus	Epidorylaimus	**-1.053**	**0.199**	**5.600**	**4.558**
Aglenchus	Eudorylaimus	**-1.053**	**-0.166**	**5.600**	**4.859**
Aglenchus	Mesodorylaimus	**-1.053**	**-0.277**	**5.600**	**4.859**
Aglenchus	Prodorylaimus	**-1.053**	**-0.836**	**5.600**	**4.558**
Aglenchus	Pungentus	**-1.053**	**0.263**	**5.600**	**4.558**
Aglenchus	Thornematidae	**-1.053**	**-0.470**	**5.600**	**4.859**
Aglenchus	Scutacarus	**-1.053**	**-0.608**	**5.600**	**2.849**
Aglenchus	Tarsonemus	**-1.053**	**-0.701**	**5.600**	**2.371**
Filenchus	Mononchus	**-1.033**	**-0.938**	**5.513**	**4.558**
Filenchus	Mylonchulus	**-1.033**	**-0.005**	**5.513**	**4.558**
Filenchus	Tripyla	**-1.033**	**-0.420**	**5.513**	**5.035**
Filenchus	Cheiroseius	**-1.033**	**0.356**	**5.513**	**2.371**
Filenchus	Dendrolaelaps	**-1.033**	**0.027**	**5.513**	**2.371**
Filenchus	Lysigamasus	**-1.033**	**0.407**	**5.513**	**2.371**
Filenchus	Pachylaelaps	**-1.033**	**0.375**	**5.513**	**2.371**
Filenchus	Veigaia	**-1.033**	**0.613**	**5.513**	**2.371**
Filenchus	Aporcelaimellus	**-1.033**	**0.548**	**5.513**	**5.257**
Filenchus	Dorylaimoidea	**-1.033**	**-0.604**	**5.513**	**5.160**
Filenchus	Epidorylaimus	**-1.033**	**0.199**	**5.513**	**4.558**
Filenchus	Eudorylaimus	**-1.033**	**-0.166**	**5.513**	**4.859**
Filenchus	Mesodorylaimus	**-1.033**	**-0.277**	**5.513**	**4.859**
Filenchus	Prodorylaimus	**-1.033**	**-0.836**	**5.513**	**4.558**
Filenchus	Pungentus	**-1.033**	**0.263**	**5.513**	**4.558**
Filenchus	Thornematidae	**-1.033**	**-0.470**	**5.513**	**4.859**
Filenchus	Scutacarus	**-1.033**	**-0.608**	**5.513**	**2.849**
Filenchus	Tarsonemus	**-1.033**	**-0.701**	**5.513**	**2.371**
Helicotylenchus	Mononchus	**-0.792**	**-0.938**	**5.403**	**4.558**
Helicotylenchus	Mylonchulus	**-0.792**	**-0.005**	**5.403**	**4.558**
Helicotylenchus	Tripyla	**-0.792**	**-0.420**	**5.403**	**5.035**
Helicotylenchus	Cheiroseius	**-0.792**	**0.356**	**5.403**	**2.371**
Helicotylenchus	Dendrolaelaps	**-0.792**	**0.027**	**5.403**	**2.371**
Helicotylenchus	Lysigamasus	**-0.792**	**0.407**	**5.403**	**2.371**
Helicotylenchus	Pachylaelaps	**-0.792**	**0.375**	**5.403**	**2.371**
Helicotylenchus	Veigaia	**-0.792**	**0.613**	**5.403**	**2.371**
Helicotylenchus	Aporcelaimellus	**-0.792**	**0.548**	**5.403**	**5.257**
Helicotylenchus	Dorylaimoidea	**-0.792**	**-0.604**	**5.403**	**5.160**
Helicotylenchus	Epidorylaimus	**-0.792**	**0.199**	**5.403**	**4.558**
Helicotylenchus	Eudorylaimus	**-0.792**	**-0.166**	**5.403**	**4.859**
Helicotylenchus	Mesodorylaimus	**-0.792**	**-0.277**	**5.403**	**4.859**
Helicotylenchus	Prodorylaimus	**-0.792**	**-0.836**	**5.403**	**4.558**

Helicotylenchus	Pungentus	**-0.792**	**0.263**	**5.403**	**4.558**
Helicotylenchus	Thornematidae	**-0.792**	**-0.470**	**5.403**	**4.859**
Helicotylenchus	Scutacarus	**-0.792**	**-0.608**	**5.403**	**2.849**
Helicotylenchus	Tarsonemus	**-0.792**	**-0.701**	**5.403**	**2.371**
Heterodera	Mononchus	**-0.883**	**-0.938**	**5.558**	**4.558**
Heterodera	Mylonchulus	**-0.883**	**-0.005**	**5.558**	**4.558**
Heterodera	Tripyla	**-0.883**	**-0.420**	**5.558**	**5.035**
Heterodera	Cheiroseius	**-0.883**	**0.356**	**5.558**	**2.371**
Heterodera	Dendrolaelaps	**-0.883**	**0.027**	**5.558**	**2.371**
Heterodera	Lysigamasus	**-0.883**	**0.407**	**5.558**	**2.371**
Heterodera	Pachylaelaps	**-0.883**	**0.375**	**5.558**	**2.371**
Heterodera	Veigaia	**-0.883**	**0.613**	**5.558**	**2.371**
Heterodera	Aporcelaimellus	**-0.883**	**0.548**	**5.558**	**5.257**
Heterodera	Dorylaimoidea	**-0.883**	**-0.604**	**5.558**	**5.160**
Heterodera	Epidorylaimus	**-0.883**	**0.199**	**5.558**	**4.558**
Heterodera	Eudorylaimus	**-0.883**	**-0.166**	**5.558**	**4.859**
Heterodera	Mesodorylaimus	**-0.883**	**-0.277**	**5.558**	**4.859**
Heterodera	Prodorylaimus	**-0.883**	**-0.836**	**5.558**	**4.558**
Heterodera	Pungentus	**-0.883**	**0.263**	**5.558**	**4.558**
Heterodera	Thornematidae	**-0.883**	**-0.470**	**5.558**	**4.859**
Heterodera	Scutacarus	**-0.883**	**-0.608**	**5.558**	**2.849**
Heterodera	Tarsonemus	**-0.883**	**-0.701**	**5.558**	**2.371**
Malenchus	Mononchus	**-1.330**	**-0.938**	**4.859**	**4.558**
Malenchus	Mylonchulus	**-1.330**	**-0.005**	**4.859**	**4.558**
Malenchus	Tripyla	**-1.330**	**-0.420**	**4.859**	**5.035**
Malenchus	Cheiroseius	**-1.330**	**0.356**	**4.859**	**2.371**
Malenchus	Dendrolaelaps	**-1.330**	**0.027**	**4.859**	**2.371**
Malenchus	Lysigamasus	**-1.330**	**0.407**	**4.859**	**2.371**
Malenchus	Pachylaelaps	**-1.330**	**0.375**	**4.859**	**2.371**
Malenchus	Veigaia	**-1.330**	**0.613**	**4.859**	**2.371**
Malenchus	Aporcelaimellus	**-1.330**	**0.548**	**4.859**	**5.257**
Malenchus	Dorylaimoidea	**-1.330**	**-0.604**	**4.859**	**5.160**
Malenchus	Epidorylaimus	**-1.330**	**0.199**	**4.859**	**4.558**
Malenchus	Eudorylaimus	**-1.330**	**-0.166**	**4.859**	**4.859**
Malenchus	Mesodorylaimus	**-1.330**	**-0.277**	**4.859**	**4.859**
Malenchus	Prodorylaimus	**-1.330**	**-0.836**	**4.859**	**4.558**
Malenchus	Pungentus	**-1.330**	**0.263**	**4.859**	**4.558**
Malenchus	Thornematidae	**-1.330**	**-0.470**	**4.859**	**4.859**
Malenchus	Scutacarus	**-1.330**	**-0.608**	**4.859**	**2.849**
Malenchus	Tarsonemus	**-1.330**	**-0.701**	**4.859**	**2.371**
Meloidogyne	Mononchus	**-1.287**	**-0.938**	**4.859**	**4.558**
Meloidogyne	Mylonchulus	**-1.287**	**-0.005**	**4.859**	**4.558**
Meloidogyne	Tripyla	**-1.287**	**-0.420**	**4.859**	**5.035**
Meloidogyne	Cheiroseius	**-1.287**	**0.356**	**4.859**	**2.371**
Meloidogyne	Dendrolaelaps	**-1.287**	**0.027**	**4.859**	**2.371**
Meloidogyne	Lysigamasus	**-1.287**	**0.407**	**4.859**	**2.371**
Meloidogyne	Pachylaelaps	**-1.287**	**0.375**	**4.859**	**2.371**
Meloidogyne	Veigaia	**-1.287**	**0.613**	**4.859**	**2.371**
Meloidogyne	Aporcelaimellus	**-1.287**	**0.548**	**4.859**	**5.257**
Meloidogyne	Dorylaimoidea	**-1.287**	**-0.604**	**4.859**	**5.160**
Meloidogyne	Epidorylaimus	**-1.287**	**0.199**	**4.859**	**4.558**

Meloidogyne	Eudorylaimus	**-1.287**	**-0.166**	**4.859**	**4.859**
Meloidogyne	Mesodorylaimus	**-1.287**	**-0.277**	**4.859**	**4.859**
Meloidogyne	Prodorylaimus	**-1.287**	**-0.836**	**4.859**	**4.558**
Meloidogyne	Pungentus	**-1.287**	**0.263**	**4.859**	**4.558**
Meloidogyne	Thornematidae	**-1.287**	**-0.470**	**4.859**	**4.859**
Meloidogyne	Scutacarus	**-1.287**	**-0.608**	**4.859**	**2.849**
Meloidogyne	Tarsonemus	**-1.287**	**-0.701**	**4.859**	**2.371**
Paratylenchus	Mononchus	**-1.244**	**-0.938**	**5.257**	**4.558**
Paratylenchus	Mylonchulus	**-1.244**	**-0.005**	**5.257**	**4.558**
Paratylenchus	Tripyla	**-1.244**	**-0.420**	**5.257**	**5.035**
Paratylenchus	Cheiroseius	**-1.244**	**0.356**	**5.257**	**2.371**
Paratylenchus	Dendrolaelaps	**-1.244**	**0.027**	**5.257**	**2.371**
Paratylenchus	Lysigamasus	**-1.244**	**0.407**	**5.257**	**2.371**
Paratylenchus	Pachylaelaps	**-1.244**	**0.375**	**5.257**	**2.371**
Paratylenchus	Veigaia	**-1.244**	**0.613**	**5.257**	**2.371**
Paratylenchus	Aporcelaimellus	**-1.244**	**0.548**	**5.257**	**5.257**
Paratylenchus	Dorylaimoidea	**-1.244**	**-0.604**	**5.257**	**5.160**
Paratylenchus	Epidorylaimus	**-1.244**	**0.199**	**5.257**	**4.558**
Paratylenchus	Eudorylaimus	**-1.244**	**-0.166**	**5.257**	**4.859**
Paratylenchus	Mesodorylaimus	**-1.244**	**-0.277**	**5.257**	**4.859**
Paratylenchus	Prodorylaimus	**-1.244**	**-0.836**	**5.257**	**4.558**
Paratylenchus	Pungentus	**-1.244**	**0.263**	**5.257**	**4.558**
Paratylenchus	Thornematidae	**-1.244**	**-0.470**	**5.257**	**4.859**
Paratylenchus	Scutacarus	**-1.244**	**-0.608**	**5.257**	**2.849**
Paratylenchus	Tarsonemus	**-1.244**	**-0.701**	**5.257**	**2.371**
Pratylenchus	Mononchus	**-1.226**	**-0.938**	**4.859**	**4.558**
Pratylenchus	Mylonchulus	**-1.226**	**-0.005**	**4.859**	**4.558**
Pratylenchus	Tripyla	**-1.226**	**-0.420**	**4.859**	**5.035**
Pratylenchus	Cheiroseius	**-1.226**	**0.356**	**4.859**	**2.371**
Pratylenchus	Dendrolaelaps	**-1.226**	**0.027**	**4.859**	**2.371**
Pratylenchus	Lysigamasus	**-1.226**	**0.407**	**4.859**	**2.371**
Pratylenchus	Pachylaelaps	**-1.226**	**0.375**	**4.859**	**2.371**
Pratylenchus	Veigaia	**-1.226**	**0.613**	**4.859**	**2.371**
Pratylenchus	Aporcelaimellus	**-1.226**	**0.548**	**4.859**	**5.257**
Pratylenchus	Dorylaimoidea	**-1.226**	**-0.604**	**4.859**	**5.160**
Pratylenchus	Epidorylaimus	**-1.226**	**0.199**	**4.859**	**4.558**
Pratylenchus	Eudorylaimus	**-1.226**	**-0.166**	**4.859**	**4.859**
Pratylenchus	Mesodorylaimus	**-1.226**	**-0.277**	**4.859**	**4.859**
Pratylenchus	Prodorylaimus	**-1.226**	**-0.836**	**4.859**	**4.558**
Pratylenchus	Pungentus	**-1.226**	**0.263**	**4.859**	**4.558**
Pratylenchus	Thornematidae	**-1.226**	**-0.470**	**4.859**	**4.859**
Pratylenchus	Scutacarus	**-1.226**	**-0.608**	**4.859**	**2.849**
Pratylenchus	Tarsonemus	**-1.226**	**-0.701**	**4.859**	**2.371**
Trichodorus	Mononchus	**-0.744**	**-0.938**	**4.558**	**4.558**
Trichodorus	Mylonchulus	**-0.744**	**-0.005**	**4.558**	**4.558**
Trichodorus	Tripyla	**-0.744**	**-0.420**	**4.558**	**5.035**
Trichodorus	Cheiroseius	**-0.744**	**0.356**	**4.558**	**2.371**
Trichodorus	Dendrolaelaps	**-0.744**	**0.027**	**4.558**	**2.371**
Trichodorus	Lysigamasus	**-0.744**	**0.407**	**4.558**	**2.371**
Trichodorus	Pachylaelaps	**-0.744**	**0.375**	**4.558**	**2.371**
Trichodorus	Veigaia	**-0.744**	**0.613**	**4.558**	**2.371**

Trichodorus	Aporcelaimellus	**-0.744**	**0.548**	**4.558**	**5.257**
Trichodorus	Dorylaimoidea	**-0.744**	**-0.604**	**4.558**	**5.160**
Trichodorus	Epidorylaimus	**-0.744**	**0.199**	**4.558**	**4.558**
Trichodorus	Eudorylaimus	**-0.744**	**-0.166**	**4.558**	**4.859**
Trichodorus	Mesodorylaimus	**-0.744**	**-0.277**	**4.558**	**4.859**
Trichodorus	Prodorylaimus	**-0.744**	**-0.836**	**4.558**	**4.558**
Trichodorus	Pungentus	**-0.744**	**0.263**	**4.558**	**4.558**
Trichodorus	Thornematidae	**-0.744**	**-0.470**	**4.558**	**4.859**
Trichodorus	Scutacarus	**-0.744**	**-0.608**	**4.558**	**2.849**
Trichodorus	Tarsonemus	**-0.744**	**-0.701**	**4.558**	**2.371**
Tylenchorhynchus	Mononchus	**-0.664**	**-0.938**	**5.637**	**4.558**
Tylenchorhynchus	Mylonchulus	**-0.664**	**-0.005**	**5.637**	**4.558**
Tylenchorhynchus	Tripyla	**-0.664**	**-0.420**	**5.637**	**5.035**
Tylenchorhynchus	Cheiroseius	**-0.664**	**0.356**	**5.637**	**2.371**
Tylenchorhynchus	Dendrolaelaps	**-0.664**	**0.027**	**5.637**	**2.371**
Tylenchorhynchus	Lysigamasus	**-0.664**	**0.407**	**5.637**	**2.371**
Tylenchorhynchus	Pachylaelaps	**-0.664**	**0.375**	**5.637**	**2.371**
Tylenchorhynchus	Veigaia	**-0.664**	**0.613**	**5.637**	**2.371**
Tylenchorhynchus	Aporcelaimellus	**-0.664**	**0.548**	**5.637**	**5.257**
Tylenchorhynchus	Dorylaimoidea	**-0.664**	**-0.604**	**5.637**	**5.160**
Tylenchorhynchus	Epidorylaimus	**-0.664**	**0.199**	**5.637**	**4.558**
Tylenchorhynchus	Eudorylaimus	**-0.664**	**-0.166**	**5.637**	**4.859**
Tylenchorhynchus	Mesodorylaimus	**-0.664**	**-0.277**	**5.637**	**4.859**
Tylenchorhynchus	Prodorylaimus	**-0.664**	**-0.836**	**5.637**	**4.558**
Tylenchorhynchus	Pungentus	**-0.664**	**0.263**	**5.637**	**4.558**
Tylenchorhynchus	Thornematidae	**-0.664**	**-0.470**	**5.637**	**4.859**
Tylenchorhynchus	Scutacarus	**-0.664**	**-0.608**	**5.637**	**2.849**
Tylenchorhynchus	Tarsonemus	**-0.664**	**-0.701**	**5.637**	**2.371**
Platynothrus	Bdella	**0.710**	**0.816**	**2.849**	**2.371**
Platynothrus	Cheiroseius	**0.710**	**0.356**	**2.849**	**2.371**
Platynothrus	Dendrolaelaps	**0.710**	**0.027**	**2.849**	**2.371**
Platynothrus	Lysigamasus	**0.710**	**0.407**	**2.849**	**2.371**
Platynothrus	Pachylaelaps	**0.710**	**0.375**	**2.849**	**2.371**
Platynothrus	Veigaia	**0.710**	**0.613**	**2.849**	**2.371**
Platynothrus	Aporcelaimellus	**0.710**	**0.548**	**2.849**	**5.257**
Platynothrus	Dorylaimoidea	**0.710**	**-0.604**	**2.849**	**5.160**
Platynothrus	Epidorylaimus	**0.710**	**0.199**	**2.849**	**4.558**
Platynothrus	Eudorylaimus	**0.710**	**-0.166**	**2.849**	**4.859**
Platynothrus	Mesodorylaimus	**0.710**	**-0.277**	**2.849**	**4.859**
Platynothrus	Prodorylaimus	**0.710**	**-0.836**	**2.849**	**4.558**
Platynothrus	Pungentus	**0.710**	**0.263**	**2.849**	**4.558**
Platynothrus	Thornematidae	**0.710**	**-0.470**	**2.849**	**4.859**
Platynothrus	Scutacarus	**0.710**	**-0.608**	**2.849**	**2.849**
Platynothrus	Tarsonemus	**0.710**	**-0.701**	**2.849**	**2.371**
Rhizoglyphus	Bdella	**0.005**	**0.816**	**2.371**	**2.371**
Rhizoglyphus	Cheiroseius	**0.005**	**0.356**	**2.371**	**2.371**
Rhizoglyphus	Dendrolaelaps	**0.005**	**0.027**	**2.371**	**2.371**
Rhizoglyphus	Lysigamasus	**0.005**	**0.407**	**2.371**	**2.371**
Rhizoglyphus	Pachylaelaps	**0.005**	**0.375**	**2.371**	**2.371**
Rhizoglyphus	Veigaia	**0.005**	**0.613**	**2.371**	**2.371**
Rhizoglyphus	Aporcelaimellus	**0.005**	**0.548**	**2.371**	**5.257**

Rhizoglyphus	Dorylaimoidea	**0.005**	**-0.604**	**2.371**	**5.160**
Rhizoglyphus	Epidorylaimus	**0.005**	**0.199**	**2.371**	**4.558**
Rhizoglyphus	Eudorylaimus	**0.005**	**-0.166**	**2.371**	**4.859**
Rhizoglyphus	Mesodorylaimus	**0.005**	**-0.277**	**2.371**	**4.859**
Rhizoglyphus	Prodorylaimus	**0.005**	**-0.836**	**2.371**	**4.558**
Rhizoglyphus	Pungentus	**0.005**	**0.263**	**2.371**	**4.558**
Rhizoglyphus	Thornematidae	**0.005**	**-0.470**	**2.371**	**4.859**
Rhizoglyphus	Scutacarus	**0.005**	**-0.608**	**2.371**	**2.849**
Rhizoglyphus	Tarsonemus	**0.005**	**-0.701**	**2.371**	**2.371**
Tydeidae	Bdella	**-0.608**	**0.816**	**2.371**	**2.371**
Tydeidae	Cheiroseius	**-0.608**	**0.356**	**2.371**	**2.371**
Tydeidae	Dendrolaelaps	**-0.608**	**0.027**	**2.371**	**2.371**
Tydeidae	Lysigamasus	**-0.608**	**0.407**	**2.371**	**2.371**
Tydeidae	Pachylaelaps	**-0.608**	**0.375**	**2.371**	**2.371**
Tydeidae	Veigaia	**-0.608**	**0.613**	**2.371**	**2.371**
Tydeidae	Aporcelaimellus	**-0.608**	**0.548**	**2.371**	**5.257**
Tydeidae	Dorylaimoidea	**-0.608**	**-0.604**	**2.371**	**5.160**
Tydeidae	Epidorylaimus	**-0.608**	**0.199**	**2.371**	**4.558**
Tydeidae	Eudorylaimus	**-0.608**	**-0.166**	**2.371**	**4.859**
Tydeidae	Mesodorylaimus	**-0.608**	**-0.277**	**2.371**	**4.859**
Tydeidae	Prodorylaimus	**-0.608**	**-0.836**	**2.371**	**4.558**
Tydeidae	Pungentus	**-0.608**	**0.263**	**2.371**	**4.558**
Tydeidae	Thornematidae	**-0.608**	**-0.470**	**2.371**	**4.859**
Tydeidae	Scutacarus	**-0.608**	**-0.608**	**2.371**	**2.849**
Tydeidae	Tarsonemus	**-0.608**	**-0.701**	**2.371**	**2.371**
Sminthuridae	Cheiroseius	**-0.608**	**0.356**	**3.070**	**2.371**
Sminthuridae	Dendrolaelaps	**-0.608**	**0.027**	**3.070**	**2.371**
Sminthuridae	Lysigamasus	**-0.608**	**0.407**	**3.070**	**2.371**
Sminthuridae	Pachylaelaps	**-0.608**	**0.375**	**3.070**	**2.371**
Sminthuridae	Veigaia	**-0.608**	**0.613**	**3.070**	**2.371**
Sminthuridae	Aporcelaimellus	**-0.608**	**0.548**	**3.070**	**5.257**
Sminthuridae	Dorylaimoidea	**-0.608**	**-0.604**	**3.070**	**5.160**
Sminthuridae	Epidorylaimus	**-0.608**	**0.199**	**3.070**	**4.558**
Sminthuridae	Eudorylaimus	**-0.608**	**-0.166**	**3.070**	**4.859**
Sminthuridae	Mesodorylaimus	**-0.608**	**-0.277**	**3.070**	**4.859**
Sminthuridae	Prodorylaimus	**-0.608**	**-0.836**	**3.070**	**4.558**
Sminthuridae	Pungentus	**-0.608**	**0.263**	**3.070**	**4.558**
Sminthuridae	Thornematidae	**-0.608**	**-0.470**	**3.070**	**4.859**
Sminthuridae	Scutacarus	**-0.608**	**-0.608**	**3.070**	**2.849**
Sminthuridae	Tarsonemus	**-0.608**	**-0.701**	**3.070**	**2.371**
Sminthurinus	Cheiroseius	**0.618**	**0.356**	**3.150**	**2.371**
Sminthurinus	Dendrolaelaps	**0.618**	**0.027**	**3.150**	**2.371**
Sminthurinus	Lysigamasus	**0.618**	**0.407**	**3.150**	**2.371**
Sminthurinus	Pachylaelaps	**0.618**	**0.375**	**3.150**	**2.371**
Sminthurinus	Veigaia	**0.618**	**0.613**	**3.150**	**2.371**
Sminthurinus	Aporcelaimellus	**0.618**	**0.548**	**3.150**	**5.257**
Sminthurinus	Dorylaimoidea	**0.618**	**-0.604**	**3.150**	**5.160**
Sminthurinus	Epidorylaimus	**0.618**	**0.199**	**3.150**	**4.558**
Sminthurinus	Eudorylaimus	**0.618**	**-0.166**	**3.150**	**4.859**
Sminthurinus	Mesodorylaimus	**0.618**	**-0.277**	**3.150**	**4.859**
Sminthurinus	Prodorylaimus	**0.618**	**-0.836**	**3.150**	**4.558**

Sminthurinus	Pungentus	**0.618**	**0.263**	**3.150**	**4.558**
Sminthurinus	Thornematidae	**0.618**	**-0.470**	**3.150**	**4.859**
Sminthurinus	Scutacarus	**0.618**	**-0.608**	**3.150**	**2.849**
Sminthurinus	Tarsonemus	**0.618**	**-0.701**	**3.150**	**2.371**
Sphaeridia	Cheiroseius	**0.202**	**0.356**	**2.672**	**2.371**
Sphaeridia	Dendrolaelaps	**0.202**	**0.027**	**2.672**	**2.371**
Sphaeridia	Lysigamasus	**0.202**	**0.407**	**2.672**	**2.371**
Sphaeridia	Pachylaelaps	**0.202**	**0.375**	**2.672**	**2.371**
Sphaeridia	Veigaia	**0.202**	**0.613**	**2.672**	**2.371**
Sphaeridia	Aporcelaimellus	**0.202**	**0.548**	**2.672**	**5.257**
Sphaeridia	Dorylaimoidea	**0.202**	**-0.604**	**2.672**	**5.160**
Sphaeridia	Epidorylaimus	**0.202**	**0.199**	**2.672**	**4.558**
Sphaeridia	Eudorylaimus	**0.202**	**-0.166**	**2.672**	**4.859**
Sphaeridia	Mesodorylaimus	**0.202**	**-0.277**	**2.672**	**4.859**
Sphaeridia	Prodorylaimus	**0.202**	**-0.836**	**2.672**	**4.558**
Sphaeridia	Pungentus	**0.202**	**0.263**	**2.672**	**4.558**
Sphaeridia	Thornematidae	**0.202**	**-0.470**	**2.672**	**4.859**
Sphaeridia	Scutacarus	**0.202**	**-0.608**	**2.672**	**2.849**
Sphaeridia	Tarsonemus	**0.202**	**-0.701**	**2.672**	**2.371**
Aphelenchoides	Mononchus	**-1.496**	**-0.938**	**4.558**	**4.558**
Aphelenchoides	Mylonchulus	**-1.496**	**-0.005**	**4.558**	**4.558**
Aphelenchoides	Tripyla	**-1.496**	**-0.420**	**4.558**	**5.035**
Aphelenchoides	Cheiroseius	**-1.496**	**0.356**	**4.558**	**2.371**
Aphelenchoides	Dendrolaelaps	**-1.496**	**0.027**	**4.558**	**2.371**
Aphelenchoides	Lysigamasus	**-1.496**	**0.407**	**4.558**	**2.371**
Aphelenchoides	Pachylaelaps	**-1.496**	**0.375**	**4.558**	**2.371**
Aphelenchoides	Veigaia	**-1.496**	**0.613**	**4.558**	**2.371**
Aphelenchoides	Aporcelaimellus	**-1.496**	**0.548**	**4.558**	**5.257**
Aphelenchoides	Dorylaimoidea	**-1.496**	**-0.604**	**4.558**	**5.160**
Aphelenchoides	Epidorylaimus	**-1.496**	**0.199**	**4.558**	**4.558**
Aphelenchoides	Eudorylaimus	**-1.496**	**-0.166**	**4.558**	**4.859**
Aphelenchoides	Mesodorylaimus	**-1.496**	**-0.277**	**4.558**	**4.859**
Aphelenchoides	Prodorylaimus	**-1.496**	**-0.836**	**4.558**	**4.558**
Aphelenchoides	Pungentus	**-1.496**	**0.263**	**4.558**	**4.558**
Aphelenchoides	Thornematidae	**-1.496**	**-0.470**	**4.558**	**4.859**
Aphelenchoides	Scutacarus	**-1.496**	**-0.608**	**4.558**	**2.849**
Aphelenchoides	Tarsonemus	**-1.496**	**-0.701**	**4.558**	**2.371**
Aphelenchus	Mononchus	**-1.129**	**-0.938**	**4.558**	**4.558**
Aphelenchus	Mylonchulus	**-1.129**	**-0.005**	**4.558**	**4.558**
Aphelenchus	Tripyla	**-1.129**	**-0.420**	**4.558**	**5.035**
Aphelenchus	Cheiroseius	**-1.129**	**0.356**	**4.558**	**2.371**
Aphelenchus	Dendrolaelaps	**-1.129**	**0.027**	**4.558**	**2.371**
Aphelenchus	Lysigamasus	**-1.129**	**0.407**	**4.558**	**2.371**
Aphelenchus	Pachylaelaps	**-1.129**	**0.375**	**4.558**	**2.371**
Aphelenchus	Veigaia	**-1.129**	**0.613**	**4.558**	**2.371**
Aphelenchus	Aporcelaimellus	**-1.129**	**0.548**	**4.558**	**5.257**
Aphelenchus	Dorylaimoidea	**-1.129**	**-0.604**	**4.558**	**5.160**
Aphelenchus	Epidorylaimus	**-1.129**	**0.199**	**4.558**	**4.558**
Aphelenchus	Eudorylaimus	**-1.129**	**-0.166**	**4.558**	**4.859**
Aphelenchus	Mesodorylaimus	**-1.129**	**-0.277**	**4.558**	**4.859**
Aphelenchus	Prodorylaimus	**-1.129**	**-0.836**	**4.558**	**4.558**

Aphelenchus	Pungentus	**-1.129**	**0.263**	**4.558**	**4.558**
Aphelenchus	Thornematidae	**-1.129**	**-0.470**	**4.558**	**4.859**
Aphelenchus	Scutacarus	**-1.129**	**-0.608**	**4.558**	**2.849**
Aphelenchus	Tarsonemus	**-1.129**	**-0.701**	**4.558**	**2.371**
Tylenchidae	Mononchus	**-1.360**	**-0.938**	**5.600**	**4.558**
Tylenchidae	Mylonchulus	**-1.360**	**-0.005**	**5.600**	**4.558**
Tylenchidae	Tripyla	**-1.360**	**-0.420**	**5.600**	**5.035**
Tylenchidae	Cheiroseius	**-1.360**	**0.356**	**5.600**	**2.371**
Tylenchidae	Dendrolaelaps	**-1.360**	**0.027**	**5.600**	**2.371**
Tylenchidae	Lysigamasus	**-1.360**	**0.407**	**5.600**	**2.371**
Tylenchidae	Pachylaelaps	**-1.360**	**0.375**	**5.600**	**2.371**
Tylenchidae	Veigaia	**-1.360**	**0.613**	**5.600**	**2.371**
Tylenchidae	Aporcelaimellus	**-1.360**	**0.548**	**5.600**	**5.257**
Tylenchidae	Dorylaimoidea	**-1.360**	**-0.604**	**5.600**	**5.160**
Tylenchidae	Epidorylaimus	**-1.360**	**0.199**	**5.600**	**4.558**
Tylenchidae	Eudorylaimus	**-1.360**	**-0.166**	**5.600**	**4.859**
Tylenchidae	Mesodorylaimus	**-1.360**	**-0.277**	**5.600**	**4.859**
Tylenchidae	Prodorylaimus	**-1.360**	**-0.836**	**5.600**	**4.558**
Tylenchidae	Pungentus	**-1.360**	**0.263**	**5.600**	**4.558**
Tylenchidae	Thornematidae	**-1.360**	**-0.470**	**5.600**	**4.859**
Tylenchidae	Scutacarus	**-1.360**	**-0.608**	**5.600**	**2.849**
Tylenchidae	Tarsonemus	**-1.360**	**-0.701**	**5.600**	**2.371**
Microtydeus	Bdella	**-0.863**	**0.816**	**2.371**	**2.371**
Microtydeus	Cheiroseius	**-0.863**	**0.356**	**2.371**	**2.371**
Microtydeus	Dendrolaelaps	**-0.863**	**0.027**	**2.371**	**2.371**
Microtydeus	Lysigamasus	**-0.863**	**0.407**	**2.371**	**2.371**
Microtydeus	Pachylaelaps	**-0.863**	**0.375**	**2.371**	**2.371**
Microtydeus	Veigaia	**-0.863**	**0.613**	**2.371**	**2.371**
Microtydeus	Aporcelaimellus	**-0.863**	**0.548**	**2.371**	**5.257**
Microtydeus	Dorylaimoidea	**-0.863**	**-0.604**	**2.371**	**5.160**
Microtydeus	Epidorylaimus	**-0.863**	**0.199**	**2.371**	**4.558**
Microtydeus	Eudorylaimus	**-0.863**	**-0.166**	**2.371**	**4.859**
Microtydeus	Mesodorylaimus	**-0.863**	**-0.277**	**2.371**	**4.859**
Microtydeus	Prodorylaimus	**-0.863**	**-0.836**	**2.371**	**4.558**
Microtydeus	Pungentus	**-0.863**	**0.263**	**2.371**	**4.558**
Microtydeus	Thornematidae	**-0.863**	**-0.470**	**2.371**	**4.859**
Microtydeus	Scutacarus	**-0.863**	**-0.608**	**2.371**	**2.849**
Microtydeus	Tarsonemus	**-0.863**	**-0.701**	**2.371**	**2.371**
Pygmephorus	Bdella	**-0.376**	**0.816**	**2.371**	**2.371**
Pygmephorus	Cheiroseius	**-0.376**	**0.356**	**2.371**	**2.371**
Pygmephorus	Dendrolaelaps	**-0.376**	**0.027**	**2.371**	**2.371**
Pygmephorus	Lysigamasus	**-0.376**	**0.407**	**2.371**	**2.371**
Pygmephorus	Pachylaelaps	**-0.376**	**0.375**	**2.371**	**2.371**
Pygmephorus	Veigaia	**-0.376**	**0.613**	**2.371**	**2.371**
Pygmephorus	Aporcelaimellus	**-0.376**	**0.548**	**2.371**	**5.257**
Pygmephorus	Dorylaimoidea	**-0.376**	**-0.604**	**2.371**	**5.160**
Pygmephorus	Epidorylaimus	**-0.376**	**0.199**	**2.371**	**4.558**
Pygmephorus	Eudorylaimus	**-0.376**	**-0.166**	**2.371**	**4.859**
Pygmephorus	Mesodorylaimus	**-0.376**	**-0.277**	**2.371**	**4.859**
Pygmephorus	Prodorylaimus	**-0.376**	**-0.836**	**2.371**	**4.558**
Pygmephorus	Pungentus	**-0.376**	**0.263**	**2.371**	**4.558**

Pygmephorus	Thornematidae	**-0.376**	**-0.470**	**2.371**	**4.859**
Pygmephorus	Scutacarus	**-0.376**	**-0.608**	**2.371**	**2.849**
Pygmephorus	Tarsonemus	**-0.376**	**-0.701**	**2.371**	**2.371**
Isotoma	Cheiroseius	**1.898**	**0.356**	**3.890**	**2.371**
Isotoma	Dendrolaelaps	**1.898**	**0.027**	**3.890**	**2.371**
Isotoma	Lysigamasus	**1.898**	**0.407**	**3.890**	**2.371**
Isotoma	Pachylaelaps	**1.898**	**0.375**	**3.890**	**2.371**
Isotoma	Veigaia	**1.898**	**0.613**	**3.890**	**2.371**
Isotoma	Aporcelaimellus	**1.898**	**0.548**	**3.890**	**5.257**
Isotoma	Dorylaimoidea	**1.898**	**-0.604**	**3.890**	**5.160**
Isotoma	Epidorylaimus	**1.898**	**0.199**	**3.890**	**4.558**
Isotoma	Eudorylaimus	**1.898**	**-0.166**	**3.890**	**4.859**
Isotoma	Mesodorylaimus	**1.898**	**-0.277**	**3.890**	**4.859**
Isotoma	Prodorylaimus	**1.898**	**-0.836**	**3.890**	**4.558**
Isotoma	Pungentus	**1.898**	**0.263**	**3.890**	**4.558**
Isotoma	Thornematidae	**1.898**	**-0.470**	**3.890**	**4.859**
Isotoma	Scutacarus	**1.898**	**-0.608**	**3.890**	**2.849**
Isotoma	Tarsonemus	**1.898**	**-0.701**	**3.890**	**2.371**
Isotomiella	Cheiroseius	**0.816**	**0.356**	**2.371**	**2.371**
Isotomiella	Dendrolaelaps	**0.816**	**0.027**	**2.371**	**2.371**
Isotomiella	Lysigamasus	**0.816**	**0.407**	**2.371**	**2.371**
Isotomiella	Pachylaelaps	**0.816**	**0.375**	**2.371**	**2.371**
Isotomiella	Veigaia	**0.816**	**0.613**	**2.371**	**2.371**
Isotomiella	Aporcelaimellus	**0.816**	**0.548**	**2.371**	**5.257**
Isotomiella	Dorylaimoidea	**0.816**	**-0.604**	**2.371**	**5.160**
Isotomiella	Epidorylaimus	**0.816**	**0.199**	**2.371**	**4.558**
Isotomiella	Eudorylaimus	**0.816**	**-0.166**	**2.371**	**4.859**
Isotomiella	Mesodorylaimus	**0.816**	**-0.277**	**2.371**	**4.859**
Isotomiella	Prodorylaimus	**0.816**	**-0.836**	**2.371**	**4.558**
Isotomiella	Pungentus	**0.816**	**0.263**	**2.371**	**4.558**
Isotomiella	Thornematidae	**0.816**	**-0.470**	**2.371**	**4.859**
Isotomiella	Scutacarus	**0.816**	**-0.608**	**2.371**	**2.849**
Isotomiella	Tarsonemus	**0.816**	**-0.701**	**2.371**	**2.371**
Lepidocyrtus	Cheiroseius	**1.231**	**0.356**	**3.070**	**2.371**
Lepidocyrtus	Dendrolaelaps	**1.231**	**0.027**	**3.070**	**2.371**
Lepidocyrtus	Lysigamasus	**1.231**	**0.407**	**3.070**	**2.371**
Lepidocyrtus	Pachylaelaps	**1.231**	**0.375**	**3.070**	**2.371**
Lepidocyrtus	Veigaia	**1.231**	**0.613**	**3.070**	**2.371**
Lepidocyrtus	Aporcelaimellus	**1.231**	**0.548**	**3.070**	**5.257**
Lepidocyrtus	Dorylaimoidea	**1.231**	**-0.604**	**3.070**	**5.160**
Lepidocyrtus	Epidorylaimus	**1.231**	**0.199**	**3.070**	**4.558**
Lepidocyrtus	Eudorylaimus	**1.231**	**-0.166**	**3.070**	**4.859**
Lepidocyrtus	Mesodorylaimus	**1.231**	**-0.277**	**3.070**	**4.859**
Lepidocyrtus	Prodorylaimus	**1.231**	**-0.836**	**3.070**	**4.558**
Lepidocyrtus	Pungentus	**1.231**	**0.263**	**3.070**	**4.558**
Lepidocyrtus	Thornematidae	**1.231**	**-0.470**	**3.070**	**4.859**
Lepidocyrtus	Scutacarus	**1.231**	**-0.608**	**3.070**	**2.849**
Lepidocyrtus	Tarsonemus	**1.231**	**-0.701**	**3.070**	**2.371**
Parisotoma	Cheiroseius	**0.722**	**0.356**	**2.371**	**2.371**
Parisotoma	Dendrolaelaps	**0.722**	**0.027**	**2.371**	**2.371**
Parisotoma	Lysigamasus	**0.722**	**0.407**	**2.371**	**2.371**

Parisotoma	Pachylaelaps	**0.722**	**0.375**	**2.371**	**2.371**
Parisotoma	Veigaia	**0.722**	**0.613**	**2.371**	**2.371**
Parisotoma	Aporcelaimellus	**0.722**	**0.548**	**2.371**	**5.257**
Parisotoma	Dorylaimoidea	**0.722**	**-0.604**	**2.371**	**5.160**
Parisotoma	Epidorylaimus	**0.722**	**0.199**	**2.371**	**4.558**
Parisotoma	Eudorylaimus	**0.722**	**-0.166**	**2.371**	**4.859**
Parisotoma	Mesodorylaimus	**0.722**	**-0.277**	**2.371**	**4.859**
Parisotoma	Prodorylaimus	**0.722**	**-0.836**	**2.371**	**4.558**
Parisotoma	Pungentus	**0.722**	**0.263**	**2.371**	**4.558**
Parisotoma	Thornematidae	**0.722**	**-0.470**	**2.371**	**4.859**
Parisotoma	Scutacarus	**0.722**	**-0.608**	**2.371**	**2.849**
Parisotoma	Tarsonemus	**0.722**	**-0.701**	**2.371**	**2.371**
Achaeta	Cheiroseius	**0.977**	**0.356**	**3.891**	**2.371**
Achaeta	Dendrolaelaps	**0.977**	**0.027**	**3.891**	**2.371**
Achaeta	Lysigamasus	**0.977**	**0.407**	**3.891**	**2.371**
Achaeta	Pachylaelaps	**0.977**	**0.375**	**3.891**	**2.371**
Achaeta	Veigaia	**0.977**	**0.613**	**3.891**	**2.371**
Achaeta	Aporcelaimellus	**0.977**	**0.548**	**3.891**	**5.257**
Achaeta	Dorylaimoidea	**0.977**	**-0.604**	**3.891**	**5.160**
Achaeta	Epidorylaimus	**0.977**	**0.199**	**3.891**	**4.558**
Achaeta	Eudorylaimus	**0.977**	**-0.166**	**3.891**	**4.859**
Achaeta	Mesodorylaimus	**0.977**	**-0.277**	**3.891**	**4.859**
Achaeta	Prodorylaimus	**0.977**	**-0.836**	**3.891**	**4.558**
Achaeta	Pungentus	**0.977**	**0.263**	**3.891**	**4.558**
Achaeta	Thornematidae	**0.977**	**-0.470**	**3.891**	**4.859**
Achaeta	Scutacarus	**0.977**	**-0.608**	**3.891**	**2.849**
Achaeta	Tarsonemus	**0.977**	**-0.701**	**3.891**	**2.371**
Cognettia	Cheiroseius	**2.110**	**0.356**	**4.211**	**2.371**
Cognettia	Dendrolaelaps	**2.110**	**0.027**	**4.211**	**2.371**
Cognettia	Lysigamasus	**2.110**	**0.407**	**4.211**	**2.371**
Cognettia	Pachylaelaps	**2.110**	**0.375**	**4.211**	**2.371**
Cognettia	Veigaia	**2.110**	**0.613**	**4.211**	**2.371**
Cognettia	Aporcelaimellus	**2.110**	**0.548**	**4.211**	**5.257**
Cognettia	Dorylaimoidea	**2.110**	**-0.604**	**4.211**	**5.160**
Cognettia	Epidorylaimus	**2.110**	**0.199**	**4.211**	**4.558**
Cognettia	Eudorylaimus	**2.110**	**-0.166**	**4.211**	**4.859**
Cognettia	Mesodorylaimus	**2.110**	**-0.277**	**4.211**	**4.859**
Cognettia	Prodorylaimus	**2.110**	**-0.836**	**4.211**	**4.558**
Cognettia	Pungentus	**2.110**	**0.263**	**4.211**	**4.558**
Cognettia	Thornematidae	**2.110**	**-0.470**	**4.211**	**4.859**
Cognettia	Scutacarus	**2.110**	**-0.608**	**4.211**	**2.849**
Cognettia	Tarsonemus	**2.110**	**-0.701**	**4.211**	**2.371**
Fridericia	Cheiroseius	**2.882**	**0.356**	**4.843**	**2.371**
Fridericia	Dendrolaelaps	**2.882**	**0.027**	**4.843**	**2.371**
Fridericia	Lysigamasus	**2.882**	**0.407**	**4.843**	**2.371**
Fridericia	Pachylaelaps	**2.882**	**0.375**	**4.843**	**2.371**
Fridericia	Veigaia	**2.882**	**0.613**	**4.843**	**2.371**
Fridericia	Aporcelaimellus	**2.882**	**0.548**	**4.843**	**5.257**
Fridericia	Dorylaimoidea	**2.882**	**-0.604**	**4.843**	**5.160**
Fridericia	Epidorylaimus	**2.882**	**0.199**	**4.843**	**4.558**
Fridericia	Eudorylaimus	**2.882**	**-0.166**	**4.843**	**4.859**

Fridericia	Mesodorylaimus	**2.882**	**-0.277**	**4.843**	**4.859**
Fridericia	Prodorylaimus	**2.882**	**-0.836**	**4.843**	**4.558**
Fridericia	Pungentus	**2.882**	**0.263**	**4.843**	**4.558**
Fridericia	Thornematidae	**2.882**	**-0.470**	**4.843**	**4.859**
Fridericia	Scutacarus	**2.882**	**-0.608**	**4.843**	**2.849**
Fridericia	Tarsonemus	**2.882**	**-0.701**	**4.843**	**2.371**
Acrobeloides	Mononchus	**-1.171**	**-0.938**	**5.035**	**4.558**
Acrobeloides	Mylonchulus	**-1.171**	**-0.005**	**5.035**	**4.558**
Acrobeloides	Tripyla	**-1.171**	**-0.420**	**5.035**	**5.035**
Acrobeloides	Cheiroseius	**-1.171**	**0.356**	**5.035**	**2.371**
Acrobeloides	Dendrolaelaps	**-1.171**	**0.027**	**5.035**	**2.371**
Acrobeloides	Lysigamasus	**-1.171**	**0.407**	**5.035**	**2.371**
Acrobeloides	Pachylaelaps	**-1.171**	**0.375**	**5.035**	**2.371**
Acrobeloides	Veigaia	**-1.171**	**0.613**	**5.035**	**2.371**
Acrobeloides	Aporcelaimellus	**-1.171**	**0.548**	**5.035**	**5.257**
Acrobeloides	Dorylaimoidea	**-1.171**	**-0.604**	**5.035**	**5.160**
Acrobeloides	Epidorylaimus	**-1.171**	**0.199**	**5.035**	**4.558**
Acrobeloides	Eudorylaimus	**-1.171**	**-0.166**	**5.035**	**4.859**
Acrobeloides	Mesodorylaimus	**-1.171**	**-0.277**	**5.035**	**4.859**
Acrobeloides	Prodorylaimus	**-1.171**	**-0.836**	**5.035**	**4.558**
Acrobeloides	Pungentus	**-1.171**	**0.263**	**5.035**	**4.558**
Acrobeloides	Thornematidae	**-1.171**	**-0.470**	**5.035**	**4.859**
Acrobeloides	Scutacarus	**-1.171**	**-0.608**	**5.035**	**2.849**
Acrobeloides	Tarsonemus	**-1.171**	**-0.701**	**5.035**	**2.371**
Alaimus	Mononchus	**-0.858**	**-0.938**	**4.558**	**4.558**
Alaimus	Mylonchulus	**-0.858**	**-0.005**	**4.558**	**4.558**
Alaimus	Tripyla	**-0.858**	**-0.420**	**4.558**	**5.035**
Alaimus	Cheiroseius	**-0.858**	**0.356**	**4.558**	**2.371**
Alaimus	Dendrolaelaps	**-0.858**	**0.027**	**4.558**	**2.371**
Alaimus	Lysigamasus	**-0.858**	**0.407**	**4.558**	**2.371**
Alaimus	Pachylaelaps	**-0.858**	**0.375**	**4.558**	**2.371**
Alaimus	Veigaia	**-0.858**	**0.613**	**4.558**	**2.371**
Alaimus	Aporcelaimellus	**-0.858**	**0.548**	**4.558**	**5.257**
Alaimus	Dorylaimoidea	**-0.858**	**-0.604**	**4.558**	**5.160**
Alaimus	Epidorylaimus	**-0.858**	**0.199**	**4.558**	**4.558**
Alaimus	Eudorylaimus	**-0.858**	**-0.166**	**4.558**	**4.859**
Alaimus	Mesodorylaimus	**-0.858**	**-0.277**	**4.558**	**4.859**
Alaimus	Prodorylaimus	**-0.858**	**-0.836**	**4.558**	**4.558**
Alaimus	Pungentus	**-0.858**	**0.263**	**4.558**	**4.558**
Alaimus	Thornematidae	**-0.858**	**-0.470**	**4.558**	**4.859**
Alaimus	Scutacarus	**-0.858**	**-0.608**	**4.558**	**2.849**
Alaimus	Tarsonemus	**-0.858**	**-0.701**	**4.558**	**2.371**
Anaplectus	Mononchus	**-0.519**	**-0.938**	**4.859**	**4.558**
Anaplectus	Mylonchulus	**-0.519**	**-0.005**	**4.859**	**4.558**
Anaplectus	Tripyla	**-0.519**	**-0.420**	**4.859**	**5.035**
Anaplectus	Cheiroseius	**-0.519**	**0.356**	**4.859**	**2.371**
Anaplectus	Dendrolaelaps	**-0.519**	**0.027**	**4.859**	**2.371**
Anaplectus	Lysigamasus	**-0.519**	**0.407**	**4.859**	**2.371**
Anaplectus	Pachylaelaps	**-0.519**	**0.375**	**4.859**	**2.371**
Anaplectus	Veigaia	**-0.519**	**0.613**	**4.859**	**2.371**
Anaplectus	Aporcelaimellus	**-0.519**	**0.548**	**4.859**	**5.257**

Anaplectus	Dorylaimoidea	**-0.519**	**-0.604**	**4.859**	**5.160**
Anaplectus	Epidorylaimus	**-0.519**	**0.199**	**4.859**	**4.558**
Anaplectus	Eudorylaimus	**-0.519**	**-0.166**	**4.859**	**4.859**
Anaplectus	Mesodorylaimus	**-0.519**	**-0.277**	**4.859**	**4.859**
Anaplectus	Prodorylaimus	**-0.519**	**-0.836**	**4.859**	**4.558**
Anaplectus	Pungentus	**-0.519**	**0.263**	**4.859**	**4.558**
Anaplectus	Thornematidae	**-0.519**	**-0.470**	**4.859**	**4.859**
Anaplectus	Scutacarus	**-0.519**	**-0.608**	**4.859**	**2.849**
Anaplectus	Tarsonemus	**-0.519**	**-0.701**	**4.859**	**2.371**
Cephalobidae	Mononchus	**-1.055**	**-0.938**	**4.558**	**4.558**
Cephalobidae	Mylonchulus	**-1.055**	**-0.005**	**4.558**	**4.558**
Cephalobidae	Tripyla	**-1.055**	**-0.420**	**4.558**	**5.035**
Cephalobidae	Cheiroseius	**-1.055**	**0.356**	**4.558**	**2.371**
Cephalobidae	Dendrolaelaps	**-1.055**	**0.027**	**4.558**	**2.371**
Cephalobidae	Lysigamasus	**-1.055**	**0.407**	**4.558**	**2.371**
Cephalobidae	Pachylaelaps	**-1.055**	**0.375**	**4.558**	**2.371**
Cephalobidae	Veigaia	**-1.055**	**0.613**	**4.558**	**2.371**
Cephalobidae	Aporcelaimellus	**-1.055**	**0.548**	**4.558**	**5.257**
Cephalobidae	Dorylaimoidea	**-1.055**	**-0.604**	**4.558**	**5.160**
Cephalobidae	Epidorylaimus	**-1.055**	**0.199**	**4.558**	**4.558**
Cephalobidae	Eudorylaimus	**-1.055**	**-0.166**	**4.558**	**4.859**
Cephalobidae	Mesodorylaimus	**-1.055**	**-0.277**	**4.558**	**4.859**
Cephalobidae	Prodorylaimus	**-1.055**	**-0.836**	**4.558**	**4.558**
Cephalobidae	Pungentus	**-1.055**	**0.263**	**4.558**	**4.558**
Cephalobidae	Thornematidae	**-1.055**	**-0.470**	**4.558**	**4.859**
Cephalobidae	Scutacarus	**-1.055**	**-0.608**	**4.558**	**2.849**
Cephalobidae	Tarsonemus	**-1.055**	**-0.701**	**4.558**	**2.371**
Eucephalobus	Mononchus	**-1.244**	**-0.938**	**5.814**	**4.558**
Eucephalobus	Mylonchulus	**-1.244**	**-0.005**	**5.814**	**4.558**
Eucephalobus	Tripyla	**-1.244**	**-0.420**	**5.814**	**5.035**
Eucephalobus	Cheiroseius	**-1.244**	**0.356**	**5.814**	**2.371**
Eucephalobus	Dendrolaelaps	**-1.244**	**0.027**	**5.814**	**2.371**
Eucephalobus	Lysigamasus	**-1.244**	**0.407**	**5.814**	**2.371**
Eucephalobus	Pachylaelaps	**-1.244**	**0.375**	**5.814**	**2.371**
Eucephalobus	Veigaia	**-1.244**	**0.613**	**5.814**	**2.371**
Eucephalobus	Aporcelaimellus	**-1.244**	**0.548**	**5.814**	**5.257**
Eucephalobus	Dorylaimoidea	**-1.244**	**-0.604**	**5.814**	**5.160**
Eucephalobus	Epidorylaimus	**-1.244**	**0.199**	**5.814**	**4.558**
Eucephalobus	Eudorylaimus	**-1.244**	**-0.166**	**5.814**	**4.859**
Eucephalobus	Mesodorylaimus	**-1.244**	**-0.277**	**5.814**	**4.859**
Eucephalobus	Prodorylaimus	**-1.244**	**-0.836**	**5.814**	**4.558**
Eucephalobus	Pungentus	**-1.244**	**0.263**	**5.814**	**4.558**
Eucephalobus	Thornematidae	**-1.244**	**-0.470**	**5.814**	**4.859**
Eucephalobus	Scutacarus	**-1.244**	**-0.608**	**5.814**	**2.849**
Eucephalobus	Tarsonemus	**-1.244**	**-0.701**	**5.814**	**2.371**
Metateratocephalus	Mononchus	**-1.506**	**-0.938**	**4.558**	**4.558**
Metateratocephalus	Mylonchulus	**-1.506**	**-0.005**	**4.558**	**4.558**
Metateratocephalus	Tripyla	**-1.506**	**-0.420**	**4.558**	**5.035**
Metateratocephalus	Cheiroseius	**-1.506**	**0.356**	**4.558**	**2.371**
Metateratocephalus	Dendrolaelaps	**-1.506**	**0.027**	**4.558**	**2.371**
Metateratocephalus	Lysigamasus	**-1.506**	**0.407**	**4.558**	**2.371**

Metateratocephalus	Pachylaelaps	**-1.506**	**0.375**	**4.558**	**2.371**
Metateratocephalus	Veigaia	**-1.506**	**0.613**	**4.558**	**2.371**
Metateratocephalus	Aporcelaimellus	**-1.506**	**0.548**	**4.558**	**5.257**
Metateratocephalus	Dorylaimoidea	**-1.506**	**-0.604**	**4.558**	**5.160**
Metateratocephalus	Epidorylaimus	**-1.506**	**0.199**	**4.558**	**4.558**
Metateratocephalus	Eudorylaimus	**-1.506**	**-0.166**	**4.558**	**4.859**
Metateratocephalus	Mesodorylaimus	**-1.506**	**-0.277**	**4.558**	**4.859**
Metateratocephalus	Prodorylaimus	**-1.506**	**-0.836**	**4.558**	**4.558**
Metateratocephalus	Pungentus	**-1.506**	**0.263**	**4.558**	**4.558**
Metateratocephalus	Thornematidae	**-1.506**	**-0.470**	**4.558**	**4.859**
Metateratocephalus	Scutacarus	**-1.506**	**-0.608**	**4.558**	**2.849**
Metateratocephalus	Tarsonemus	**-1.506**	**-0.701**	**4.558**	**2.371**
Panagrolaimus	Mononchus	**-0.945**	**-0.938**	**4.859**	**4.558**
Panagrolaimus	Mylonchulus	**-0.945**	**-0.005**	**4.859**	**4.558**
Panagrolaimus	Tripyla	**-0.945**	**-0.420**	**4.859**	**5.035**
Panagrolaimus	Cheiroseius	**-0.945**	**0.356**	**4.859**	**2.371**
Panagrolaimus	Dendrolaelaps	**-0.945**	**0.027**	**4.859**	**2.371**
Panagrolaimus	Lysigamasus	**-0.945**	**0.407**	**4.859**	**2.371**
Panagrolaimus	Pachylaelaps	**-0.945**	**0.375**	**4.859**	**2.371**
Panagrolaimus	Veigaia	**-0.945**	**0.613**	**4.859**	**2.371**
Panagrolaimus	Aporcelaimellus	**-0.945**	**0.548**	**4.859**	**5.257**
Panagrolaimus	Dorylaimoidea	**-0.945**	**-0.604**	**4.859**	**5.160**
Panagrolaimus	Epidorylaimus	**-0.945**	**0.199**	**4.859**	**4.558**
Panagrolaimus	Eudorylaimus	**-0.945**	**-0.166**	**4.859**	**4.859**
Panagrolaimus	Mesodorylaimus	**-0.945**	**-0.277**	**4.859**	**4.859**
Panagrolaimus	Prodorylaimus	**-0.945**	**-0.836**	**4.859**	**4.558**
Panagrolaimus	Pungentus	**-0.945**	**0.263**	**4.859**	**4.558**
Panagrolaimus	Thornematidae	**-0.945**	**-0.470**	**4.859**	**4.859**
Panagrolaimus	Scutacarus	**-0.945**	**-0.608**	**4.859**	**2.849**
Panagrolaimus	Tarsonemus	**-0.945**	**-0.701**	**4.859**	**2.371**
Plectus	Mononchus	**-0.583**	**-0.938**	**5.637**	**4.558**
Plectus	Mylonchulus	**-0.583**	**-0.005**	**5.637**	**4.558**
Plectus	Tripyla	**-0.583**	**-0.420**	**5.637**	**5.035**
Plectus	Cheiroseius	**-0.583**	**0.356**	**5.637**	**2.371**
Plectus	Dendrolaelaps	**-0.583**	**0.027**	**5.637**	**2.371**
Plectus	Lysigamasus	**-0.583**	**0.407**	**5.637**	**2.371**
Plectus	Pachylaelaps	**-0.583**	**0.375**	**5.637**	**2.371**
Plectus	Veigaia	**-0.583**	**0.613**	**5.637**	**2.371**
Plectus	Aporcelaimellus	**-0.583**	**0.548**	**5.637**	**5.257**
Plectus	Dorylaimoidea	**-0.583**	**-0.604**	**5.637**	**5.160**
Plectus	Epidorylaimus	**-0.583**	**0.199**	**5.637**	**4.558**
Plectus	Eudorylaimus	**-0.583**	**-0.166**	**5.637**	**4.859**
Plectus	Mesodorylaimus	**-0.583**	**-0.277**	**5.637**	**4.859**
Plectus	Prodorylaimus	**-0.583**	**-0.836**	**5.637**	**4.558**
Plectus	Pungentus	**-0.583**	**0.263**	**5.637**	**4.558**
Plectus	Thornematidae	**-0.583**	**-0.470**	**5.637**	**4.859**
Plectus	Scutacarus	**-0.583**	**-0.608**	**5.637**	**2.849**
Plectus	Tarsonemus	**-0.583**	**-0.701**	**5.637**	**2.371**
Prismatolaimus	Mononchus	**-1.280**	**-0.938**	**4.859**	**4.558**
Prismatolaimus	Mylonchulus	**-1.280**	**-0.005**	**4.859**	**4.558**
Prismatolaimus	Tripyla	**-1.280**	**-0.420**	**4.859**	**5.035**

Prismatolaimus	Cheiroseius	**-1.280**	**0.356**	**4.859**	**2.371**
Prismatolaimus	Dendrolaelaps	**-1.280**	**0.027**	**4.859**	**2.371**
Prismatolaimus	Lysigamasus	**-1.280**	**0.407**	**4.859**	**2.371**
Prismatolaimus	Pachylaelaps	**-1.280**	**0.375**	**4.859**	**2.371**
Prismatolaimus	Veigaia	**-1.280**	**0.613**	**4.859**	**2.371**
Prismatolaimus	Aporcelaimellus	**-1.280**	**0.548**	**4.859**	**5.257**
Prismatolaimus	Dorylaimoidea	**-1.280**	**-0.604**	**4.859**	**5.160**
Prismatolaimus	Epidorylaimus	**-1.280**	**0.199**	**4.859**	**4.558**
Prismatolaimus	Eudorylaimus	**-1.280**	**-0.166**	**4.859**	**4.859**
Prismatolaimus	Mesodorylaimus	**-1.280**	**-0.277**	**4.859**	**4.859**
Prismatolaimus	Prodorylaimus	**-1.280**	**-0.836**	**4.859**	**4.558**
Prismatolaimus	Pungentus	**-1.280**	**0.263**	**4.859**	**4.558**
Prismatolaimus	Thornematidae	**-1.280**	**-0.470**	**4.859**	**4.859**
Prismatolaimus	Scutacarus	**-1.280**	**-0.608**	**4.859**	**2.849**
Prismatolaimus	Tarsonemus	**-1.280**	**-0.701**	**4.859**	**2.371**
Rhabditidae	Mononchus	**-0.692**	**-0.938**	**5.035**	**4.558**
Rhabditidae	Mylonchulus	**-0.692**	**-0.005**	**5.035**	**4.558**
Rhabditidae	Tripyla	**-0.692**	**-0.420**	**5.035**	**5.035**
Rhabditidae	Cheiroseius	**-0.692**	**0.356**	**5.035**	**2.371**
Rhabditidae	Dendrolaelaps	**-0.692**	**0.027**	**5.035**	**2.371**
Rhabditidae	Lysigamasus	**-0.692**	**0.407**	**5.035**	**2.371**
Rhabditidae	Pachylaelaps	**-0.692**	**0.375**	**5.035**	**2.371**
Rhabditidae	Veigaia	**-0.692**	**0.613**	**5.035**	**2.371**
Rhabditidae	Aporcelaimellus	**-0.692**	**0.548**	**5.035**	**5.257**
Rhabditidae	Dorylaimoidea	**-0.692**	**-0.604**	**5.035**	**5.160**
Rhabditidae	Epidorylaimus	**-0.692**	**0.199**	**5.035**	**4.558**
Rhabditidae	Eudorylaimus	**-0.692**	**-0.166**	**5.035**	**4.859**
Rhabditidae	Mesodorylaimus	**-0.692**	**-0.277**	**5.035**	**4.859**
Rhabditidae	Prodorylaimus	**-0.692**	**-0.836**	**5.035**	**4.558**
Rhabditidae	Pungentus	**-0.692**	**0.263**	**5.035**	**4.558**
Rhabditidae	Thornematidae	**-0.692**	**-0.470**	**5.035**	**4.859**
Rhabditidae	Scutacarus	**-0.692**	**-0.608**	**5.035**	**2.849**
Rhabditidae	Tarsonemus	**-0.692**	**-0.701**	**5.035**	**2.371**
Teratocephalus	Mononchus	**-1.630**	**-0.938**	**4.859**	**4.558**
Teratocephalus	Mylonchulus	**-1.630**	**-0.005**	**4.859**	**4.558**
Teratocephalus	Tripyla	**-1.630**	**-0.420**	**4.859**	**5.035**
Teratocephalus	Cheiroseius	**-1.630**	**0.356**	**4.859**	**2.371**
Teratocephalus	Dendrolaelaps	**-1.630**	**0.027**	**4.859**	**2.371**
Teratocephalus	Lysigamasus	**-1.630**	**0.407**	**4.859**	**2.371**
Teratocephalus	Pachylaelaps	**-1.630**	**0.375**	**4.859**	**2.371**
Teratocephalus	Veigaia	**-1.630**	**0.613**	**4.859**	**2.371**
Teratocephalus	Aporcelaimellus	**-1.630**	**0.548**	**4.859**	**5.257**
Teratocephalus	Dorylaimoidea	**-1.630**	**-0.604**	**4.859**	**5.160**
Teratocephalus	Epidorylaimus	**-1.630**	**0.199**	**4.859**	**4.558**
Teratocephalus	Eudorylaimus	**-1.630**	**-0.166**	**4.859**	**4.859**
Teratocephalus	Mesodorylaimus	**-1.630**	**-0.277**	**4.859**	**4.859**
Teratocephalus	Prodorylaimus	**-1.630**	**-0.836**	**4.859**	**4.558**
Teratocephalus	Pungentus	**-1.630**	**0.263**	**4.859**	**4.558**
Teratocephalus	Thornematidae	**-1.630**	**-0.470**	**4.859**	**4.859**
Teratocephalus	Scutacarus	**-1.630**	**-0.608**	**4.859**	**2.849**
Teratocephalus	Tarsonemus	**-1.630**	**-0.701**	**4.859**	**2.371**

Enchytraeus	Cheiroseius	**2.293**	**0.356**	**3.871**	**2.371**
Enchytraeus	Dendrolaelaps	**2.293**	**0.027**	**3.871**	**2.371**
Enchytraeus	Lysigamasus	**2.293**	**0.407**	**3.871**	**2.371**
Enchytraeus	Pachylaelaps	**2.293**	**0.375**	**3.871**	**2.371**
Enchytraeus	Veigaia	**2.293**	**0.613**	**3.871**	**2.371**
Enchytraeus	Aporcelaimellus	**2.293**	**0.548**	**3.871**	**5.257**
Enchytraeus	Dorylaimoidea	**2.293**	**-0.604**	**3.871**	**5.160**
Enchytraeus	Epidorylaimus	**2.293**	**0.199**	**3.871**	**4.558**
Enchytraeus	Eudorylaimus	**2.293**	**-0.166**	**3.871**	**4.859**
Enchytraeus	Mesodorylaimus	**2.293**	**-0.277**	**3.871**	**4.859**
Enchytraeus	Prodorylaimus	**2.293**	**-0.836**	**3.871**	**4.558**
Enchytraeus	Pungentus	**2.293**	**0.263**	**3.871**	**4.558**
Enchytraeus	Thornematidae	**2.293**	**-0.470**	**3.871**	**4.859**
Enchytraeus	Scutacarus	**2.293**	**-0.608**	**3.871**	**2.849**
Enchytraeus	Tarsonemus	**2.293**	**-0.701**	**3.871**	**2.371**
Eubacteria	Acrobeloides	**-6.597**	**-1.171**	**13.533**	**5.035**
Eubacteria	Alaimus	**-6.597**	**-0.858**	**13.533**	**4.558**
Eubacteria	Anaplectus	**-6.597**	**-0.519**	**13.533**	**4.859**
Eubacteria	Cephalobidae	**-6.597**	**-1.055**	**13.533**	**4.558**
Eubacteria	Eucephalobus	**-6.597**	**-1.244**	**13.533**	**5.814**
Eubacteria	Metateratocephalus	**-6.597**	**-1.506**	**13.533**	**4.558**
Eubacteria	Panagrolaimus	**-6.597**	**-0.945**	**13.533**	**4.859**
Eubacteria	Plectus	**-6.597**	**-0.583**	**13.533**	**5.637**
Eubacteria	Prismatolaimus	**-6.597**	**-1.280**	**13.533**	**4.859**
Eubacteria	Rhabditidae	**-6.597**	**-0.692**	**13.533**	**5.035**
Eubacteria	Teratocephalus	**-6.597**	**-1.630**	**13.533**	**4.859**
Eubacteria	Enchytraeus	**-6.597**	**2.293**	**13.533**	**3.871**
Eubacteria	Dauerlarvae	**-6.597**	**-0.804**	**13.533**	**5.160**
Eubacteria	Buchholzia	**-6.597**	**2.301**	**13.533**	**3.503**
Eubacteria	Henlea	**-6.597**	**2.320**	**13.533**	**3.628**
Eubacteria	Marionina	**-6.597**	**1.947**	**13.533**	**4.202**
Dauerlarvae	Mononchus	**-0.804**	**-0.938**	**5.160**	**4.558**
Dauerlarvae	Mylonchulus	**-0.804**	**-0.005**	**5.160**	**4.558**
Dauerlarvae	Tripyla	**-0.804**	**-0.420**	**5.160**	**5.035**
Dauerlarvae	Aporcelaimellus	**-0.804**	**0.548**	**5.160**	**5.257**
Dauerlarvae	Dorylaimoidea	**-0.804**	**-0.604**	**5.160**	**5.160**
Dauerlarvae	Epidorylaimus	**-0.804**	**0.199**	**5.160**	**4.558**
Dauerlarvae	Eudorylaimus	**-0.804**	**-0.166**	**5.160**	**4.859**
Dauerlarvae	Mesodorylaimus	**-0.804**	**-0.277**	**5.160**	**4.859**
Dauerlarvae	Prodorylaimus	**-0.804**	**-0.836**	**5.160**	**4.558**
Dauerlarvae	Pungentus	**-0.804**	**0.263**	**5.160**	**4.558**
Dauerlarvae	Thornematidae	**-0.804**	**-0.470**	**5.160**	**4.859**
Dauerlarvae	Scutacarus	**-0.804**	**-0.608**	**5.160**	**2.849**
Dauerlarvae	Tarsonemus	**-0.804**	**-0.701**	**5.160**	**2.371**
Buchholzia	Cheiroseius	**2.301**	**0.356**	**3.503**	**2.371**
Buchholzia	Dendrolaelaps	**2.301**	**0.027**	**3.503**	**2.371**
Buchholzia	Lysigamasus	**2.301**	**0.407**	**3.503**	**2.371**
Buchholzia	Pachylaelaps	**2.301**	**0.375**	**3.503**	**2.371**
Buchholzia	Veigaia	**2.301**	**0.613**	**3.503**	**2.371**
Buchholzia	Aporcelaimellus	**2.301**	**0.548**	**3.503**	**5.257**
Buchholzia	Dorylaimoidea	**2.301**	**-0.604**	**3.503**	**5.160**

Buchholzia	Epidorylaimus	**2.301**	**0.199**	**3.503**	**4.558**
Buchholzia	Eudorylaimus	**2.301**	**-0.166**	**3.503**	**4.859**
Buchholzia	Mesodorylaimus	**2.301**	**-0.277**	**3.503**	**4.859**
Buchholzia	Prodorylaimus	**2.301**	**-0.836**	**3.503**	**4.558**
Buchholzia	Pungentus	**2.301**	**0.263**	**3.503**	**4.558**
Buchholzia	Thornematidae	**2.301**	**-0.470**	**3.503**	**4.859**
Buchholzia	Scutacarus	**2.301**	**-0.608**	**3.503**	**2.849**
Buchholzia	Tarsonemus	**2.301**	**-0.701**	**3.503**	**2.371**
Henlea	Cheiroseius	**2.320**	**0.356**	**3.628**	**2.371**
Henlea	Dendrolaelaps	**2.320**	**0.027**	**3.628**	**2.371**
Henlea	Lysigamasus	**2.320**	**0.407**	**3.628**	**2.371**
Henlea	Pachylaelaps	**2.320**	**0.375**	**3.628**	**2.371**
Henlea	Veigaia	**2.320**	**0.613**	**3.628**	**2.371**
Henlea	Aporcelaimellus	**2.320**	**0.548**	**3.628**	**5.257**
Henlea	Dorylaimoidea	**2.320**	**-0.604**	**3.628**	**5.160**
Henlea	Epidorylaimus	**2.320**	**0.199**	**3.628**	**4.558**
Henlea	Eudorylaimus	**2.320**	**-0.166**	**3.628**	**4.859**
Henlea	Mesodorylaimus	**2.320**	**-0.277**	**3.628**	**4.859**
Henlea	Prodorylaimus	**2.320**	**-0.836**	**3.628**	**4.558**
Henlea	Pungentus	**2.320**	**0.263**	**3.628**	**4.558**
Henlea	Thornematidae	**2.320**	**-0.470**	**3.628**	**4.859**
Henlea	Scutacarus	**2.320**	**-0.608**	**3.628**	**2.849**
Henlea	Tarsonemus	**2.320**	**-0.701**	**3.628**	**2.371**
Marionina	Cheiroseius	**1.947**	**0.356**	**4.202**	**2.371**
Marionina	Dendrolaelaps	**1.947**	**0.027**	**4.202**	**2.371**
Marionina	Lysigamasus	**1.947**	**0.407**	**4.202**	**2.371**
Marionina	Pachylaelaps	**1.947**	**0.375**	**4.202**	**2.371**
Marionina	Veigaia	**1.947**	**0.613**	**4.202**	**2.371**
Marionina	Aporcelaimellus	**1.947**	**0.548**	**4.202**	**5.257**
Marionina	Dorylaimoidea	**1.947**	**-0.604**	**4.202**	**5.160**
Marionina	Epidorylaimus	**1.947**	**0.199**	**4.202**	**4.558**
Marionina	Eudorylaimus	**1.947**	**-0.166**	**4.202**	**4.859**
Marionina	Mesodorylaimus	**1.947**	**-0.277**	**4.202**	**4.859**
Marionina	Prodorylaimus	**1.947**	**-0.836**	**4.202**	**4.558**
Marionina	Pungentus	**1.947**	**0.263**	**4.202**	**4.558**
Marionina	Thornematidae	**1.947**	**-0.470**	**4.202**	**4.859**
Marionina	Scutacarus	**1.947**	**-0.608**	**4.202**	**2.849**
Marionina	Tarsonemus	**1.947**	**-0.701**	**4.202**	**2.371**
Hyphae and hair roots	Aglenchus	**7.139**	**-1.053**	**0.000**	**5.600**
Hyphae and hair roots	Filenchus	**7.139**	**-1.033**	**0.000**	**5.513**
Hyphae and hair roots	Helicotylenchus	**7.139**	**-0.792**	**0.000**	**5.403**
Hyphae and hair roots	Heterodera	**7.139**	**-0.883**	**0.000**	**5.558**
Hyphae and hair roots	Malenchus	**7.139**	**-1.330**	**0.000**	**4.859**
Hyphae and hair roots	Meloidogyne	**7.139**	**-1.287**	**0.000**	**4.859**
Hyphae and hair roots	Paratylenchus	**7.139**	**-1.244**	**0.000**	**5.257**
Hyphae and hair roots	Pratylenchus	**7.139**	**-1.226**	**0.000**	**4.859**
Hyphae and hair roots	Trichodorus	**7.139**	**-0.744**	**0.000**	**4.558**
Hyphae and hair roots	Tylenchorhynchus	**7.139**	**-0.664**	**0.000**	**5.637**
Hyphae and hair roots	Platynothrus	**7.139**	**0.710**	**0.000**	**2.849**
Hyphae and hair roots	Rhizoglyphus	**7.139**	**0.005**	**0.000**	**2.371**
Hyphae and hair roots	Tydeidae	**7.139**	**-0.608**	**0.000**	**2.371**

Hyphae and hair roots	Sminthuridae	7.139	-0.608	0.000	3.070
Hyphae and hair roots	Sminthurinus	7.139	0.618	0.000	3.150
Hyphae and hair roots	Sphaeridia	7.139	0.202	0.000	2.672
Hyphae and hair roots	Aphelenchoides	7.139	-1.496	0.000	4.558
Hyphae and hair roots	Aphelenchus	7.139	-1.129	0.000	4.558
Hyphae and hair roots	Tylenchidae	7.139	-1.360	0.000	5.600
Hyphae and hair roots	Microtydeus	7.139	-0.863	0.000	2.371
Hyphae and hair roots	Pygmephorus	7.139	-0.376	0.000	2.371
Hyphae and hair roots	Isotoma	7.139	1.898	0.000	3.890
Hyphae and hair roots	Isotomiella	7.139	0.816	0.000	2.371
Hyphae and hair roots	Lepidocyrtus	7.139	1.231	0.000	3.070
Hyphae and hair roots	Parisotoma	7.139	0.722	0.000	2.371
Hyphae and hair roots	Achaeta	7.139	0.977	0.000	3.891
Hyphae and hair roots	Cognettia	7.139	2.110	0.000	4.211
Hyphae and hair roots	Fridericia	7.139	2.882	0.000	4.843
Hyphae and hair roots	Aporcelaimellus	7.139	0.548	0.000	5.257
Hyphae and hair roots	Dorylaimoidea	7.139	-0.604	0.000	5.160
Hyphae and hair roots	Epidorylaimus	7.139	0.199	0.000	4.558
Hyphae and hair roots	Eudorylaimus	7.139	-0.166	0.000	4.859
Hyphae and hair roots	Mesodorylaimus	7.139	-0.277	0.000	4.859
Hyphae and hair roots	Prodorylaimus	7.139	-0.836	0.000	4.558
Hyphae and hair roots	Pungentus	7.139	0.263	0.000	4.558
Hyphae and hair roots	Thornematidae	7.139	-0.470	0.000	4.859
Hyphae and hair roots	Scutacarus	7.139	-0.608	0.000	2.849
Hyphae and hair roots	Tarsonemus	7.139	-0.701	0.000	2.371
Mononchus	Cheiroseius	-0.938	0.356	4.558	2.371
Mononchus	Dendrolaelaps	-0.938	0.027	4.558	2.371
Mononchus	Lysigamasus	-0.938	0.407	4.558	2.371
Mononchus	Pachylaelaps	-0.938	0.375	4.558	2.371
Mononchus	Veigaia	-0.938	0.613	4.558	2.371
Mononchus	Aporcelaimellus	-0.938	0.548	4.558	5.257
Mononchus	Dorylaimoidea	-0.938	-0.604	4.558	5.160
Mononchus	Epidorylaimus	-0.938	0.199	4.558	4.558
Mononchus	Eudorylaimus	-0.938	-0.166	4.558	4.859
Mononchus	Mesodorylaimus	-0.938	-0.277	4.558	4.859
Mononchus	Prodorylaimus	-0.938	-0.836	4.558	4.558
Mononchus	Pungentus	-0.938	0.263	4.558	4.558
Mononchus	Thornematidae	-0.938	-0.470	4.558	4.859
Mononchus	Scutacarus	-0.938	-0.608	4.558	2.849
Mononchus	Tarsonemus	-0.938	-0.701	4.558	2.371
Mylonchulus	Cheiroseius	-0.005	0.356	4.558	2.371
Mylonchulus	Dendrolaelaps	-0.005	0.027	4.558	2.371
Mylonchulus	Lysigamasus	-0.005	0.407	4.558	2.371
Mylonchulus	Pachylaelaps	-0.005	0.375	4.558	2.371
Mylonchulus	Veigaia	-0.005	0.613	4.558	2.371
Mylonchulus	Aporcelaimellus	-0.005	0.548	4.558	5.257
Mylonchulus	Dorylaimoidea	-0.005	-0.604	4.558	5.160
Mylonchulus	Epidorylaimus	-0.005	0.199	4.558	4.558
Mylonchulus	Eudorylaimus	-0.005	-0.166	4.558	4.859
Mylonchulus	Mesodorylaimus	-0.005	-0.277	4.558	4.859
Mylonchulus	Prodorylaimus	-0.005	-0.836	4.558	4.558

Mylonchulus	Pungentus	**-0.005**	**0.263**	**4.558**	**4.558**
Mylonchulus	Thornematidae	**-0.005**	**-0.470**	**4.558**	**4.859**
Mylonchulus	Scutacarus	**-0.005**	**-0.608**	**4.558**	**2.849**
Mylonchulus	Tarsonemus	**-0.005**	**-0.701**	**4.558**	**2.371**
Tripyla	Cheiroseius	**-0.420**	**0.356**	**5.035**	**2.371**
Tripyla	Dendrolaelaps	**-0.420**	**0.027**	**5.035**	**2.371**
Tripyla	Lysigamasus	**-0.420**	**0.407**	**5.035**	**2.371**
Tripyla	Pachylaelaps	**-0.420**	**0.375**	**5.035**	**2.371**
Tripyla	Veigaia	**-0.420**	**0.613**	**5.035**	**2.371**
Tripyla	Aporcelaimellus	**-0.420**	**0.548**	**5.035**	**5.257**
Tripyla	Dorylaimoidea	**-0.420**	**-0.604**	**5.035**	**5.160**
Tripyla	Epidorylaimus	**-0.420**	**0.199**	**5.035**	**4.558**
Tripyla	Eudorylaimus	**-0.420**	**-0.166**	**5.035**	**4.859**
Tripyla	Mesodorylaimus	**-0.420**	**-0.277**	**5.035**	**4.859**
Tripyla	Prodorylaimus	**-0.420**	**-0.836**	**5.035**	**4.558**
Tripyla	Pungentus	**-0.420**	**0.263**	**5.035**	**4.558**
Tripyla	Thornematidae	**-0.420**	**-0.470**	**5.035**	**4.859**
Tripyla	Scutacarus	**-0.420**	**-0.608**	**5.035**	**2.849**
Tripyla	Tarsonemus	**-0.420**	**-0.701**	**5.035**	**2.371**
Bdella	Bdella	**0.816**	**0.816**	**2.371**	**2.371**
Bdella	Cheiroseius	**0.816**	**0.356**	**2.371**	**2.371**
Bdella	Dendrolaelaps	**0.816**	**0.027**	**2.371**	**2.371**
Bdella	Lysigamasus	**0.816**	**0.407**	**2.371**	**2.371**
Bdella	Pachylaelaps	**0.816**	**0.375**	**2.371**	**2.371**
Bdella	Veigaia	**0.816**	**0.613**	**2.371**	**2.371**
Bdella	Scutacarus	**0.816**	**-0.608**	**2.371**	**2.849**
Bdella	Tarsonemus	**0.816**	**-0.701**	**2.371**	**2.371**
Cheiroseius	Bdella	**0.356**	**0.816**	**2.371**	**2.371**
Cheiroseius	Aporcelaimellus	**0.356**	**0.548**	**2.371**	**5.257**
Cheiroseius	Dorylaimoidea	**0.356**	**-0.604**	**2.371**	**5.160**
Cheiroseius	Epidorylaimus	**0.356**	**0.199**	**2.371**	**4.558**
Cheiroseius	Eudorylaimus	**0.356**	**-0.166**	**2.371**	**4.859**
Cheiroseius	Mesodorylaimus	**0.356**	**-0.277**	**2.371**	**4.859**
Cheiroseius	Prodorylaimus	**0.356**	**-0.836**	**2.371**	**4.558**
Cheiroseius	Pungentus	**0.356**	**0.263**	**2.371**	**4.558**
Cheiroseius	Thornematidae	**0.356**	**-0.470**	**2.371**	**4.859**
Cheiroseius	Scutacarus	**0.356**	**-0.608**	**2.371**	**2.849**
Cheiroseius	Tarsonemus	**0.356**	**-0.701**	**2.371**	**2.371**
Dendrolaelaps	Bdella	**0.027**	**0.816**	**2.371**	**2.371**
Dendrolaelaps	Aporcelaimellus	**0.027**	**0.548**	**2.371**	**5.257**
Dendrolaelaps	Dorylaimoidea	**0.027**	**-0.604**	**2.371**	**5.160**
Dendrolaelaps	Epidorylaimus	**0.027**	**0.199**	**2.371**	**4.558**
Dendrolaelaps	Eudorylaimus	**0.027**	**-0.166**	**2.371**	**4.859**
Dendrolaelaps	Mesodorylaimus	**0.027**	**-0.277**	**2.371**	**4.859**
Dendrolaelaps	Prodorylaimus	**0.027**	**-0.836**	**2.371**	**4.558**
Dendrolaelaps	Pungentus	**0.027**	**0.263**	**2.371**	**4.558**
Dendrolaelaps	Thornematidae	**0.027**	**-0.470**	**2.371**	**4.859**
Dendrolaelaps	Scutacarus	**0.027**	**-0.608**	**2.371**	**2.849**
Dendrolaelaps	Tarsonemus	**0.027**	**-0.701**	**2.371**	**2.371**
Lysigamasus	Bdella	**0.407**	**0.816**	**2.371**	**2.371**
Lysigamasus	Aporcelaimellus	**0.407**	**0.548**	**2.371**	**5.257**

Lysigamasus	Dorylaimoidea	**0.407**	**-0.604**	**2.371**	**5.160**
Lysigamasus	Epidorylaimus	**0.407**	**0.199**	**2.371**	**4.558**
Lysigamasus	Eudorylaimus	**0.407**	**-0.166**	**2.371**	**4.859**
Lysigamasus	Mesodorylaimus	**0.407**	**-0.277**	**2.371**	**4.859**
Lysigamasus	Prodorylaimus	**0.407**	**-0.836**	**2.371**	**4.558**
Lysigamasus	Pungentus	**0.407**	**0.263**	**2.371**	**4.558**
Lysigamasus	Thornematidae	**0.407**	**-0.470**	**2.371**	**4.859**
Lysigamasus	Scutacarus	**0.407**	**-0.608**	**2.371**	**2.849**
Lysigamasus	Tarsonemus	**0.407**	**-0.701**	**2.371**	**2.371**
Pachylaelaps	Bdella	**0.375**	**0.816**	**2.371**	**2.371**
Pachylaelaps	Aporcelaimellus	**0.375**	**0.548**	**2.371**	**5.257**
Pachylaelaps	Dorylaimoidea	**0.375**	**-0.604**	**2.371**	**5.160**
Pachylaelaps	Epidorylaimus	**0.375**	**0.199**	**2.371**	**4.558**
Pachylaelaps	Eudorylaimus	**0.375**	**-0.166**	**2.371**	**4.859**
Pachylaelaps	Mesodorylaimus	**0.375**	**-0.277**	**2.371**	**4.859**
Pachylaelaps	Prodorylaimus	**0.375**	**-0.836**	**2.371**	**4.558**
Pachylaelaps	Pungentus	**0.375**	**0.263**	**2.371**	**4.558**
Pachylaelaps	Thornematidae	**0.375**	**-0.470**	**2.371**	**4.859**
Pachylaelaps	Scutacarus	**0.375**	**-0.608**	**2.371**	**2.849**
Pachylaelaps	Tarsonemus	**0.375**	**-0.701**	**2.371**	**2.371**
Veigaia	Bdella	**0.613**	**0.816**	**2.371**	**2.371**
Veigaia	Aporcelaimellus	**0.613**	**0.548**	**2.371**	**5.257**
Veigaia	Dorylaimoidea	**0.613**	**-0.604**	**2.371**	**5.160**
Veigaia	Epidorylaimus	**0.613**	**0.199**	**2.371**	**4.558**
Veigaia	Eudorylaimus	**0.613**	**-0.166**	**2.371**	**4.859**
Veigaia	Mesodorylaimus	**0.613**	**-0.277**	**2.371**	**4.859**
Veigaia	Prodorylaimus	**0.613**	**-0.836**	**2.371**	**4.558**
Veigaia	Pungentus	**0.613**	**0.263**	**2.371**	**4.558**
Veigaia	Thornematidae	**0.613**	**-0.470**	**2.371**	**4.859**
Veigaia	Scutacarus	**0.613**	**-0.608**	**2.371**	**2.849**
Veigaia	Tarsonemus	**0.613**	**-0.701**	**2.371**	**2.371**
Aporcelaimellus	Mononchus	**0.548**	**-0.938**	**5.257**	**4.558**
Aporcelaimellus	Mylonchulus	**0.548**	**-0.005**	**5.257**	**4.558**
Aporcelaimellus	Tripyla	**0.548**	**-0.420**	**5.257**	**5.035**
Aporcelaimellus	Cheiroseius	**0.548**	**0.356**	**5.257**	**2.371**
Aporcelaimellus	Dendrolaelaps	**0.548**	**0.027**	**5.257**	**2.371**
Aporcelaimellus	Lysigamasus	**0.548**	**0.407**	**5.257**	**2.371**
Aporcelaimellus	Pachylaelaps	**0.548**	**0.375**	**5.257**	**2.371**
Aporcelaimellus	Veigaia	**0.548**	**0.613**	**5.257**	**2.371**
Aporcelaimellus	Aporcelaimellus	**0.548**	**0.548**	**5.257**	**5.257**
Aporcelaimellus	Dorylaimoidea	**0.548**	**-0.604**	**5.257**	**5.160**
Aporcelaimellus	Epidorylaimus	**0.548**	**0.199**	**5.257**	**4.558**
Aporcelaimellus	Eudorylaimus	**0.548**	**-0.166**	**5.257**	**4.859**
Aporcelaimellus	Mesodorylaimus	**0.548**	**-0.277**	**5.257**	**4.859**
Aporcelaimellus	Prodorylaimus	**0.548**	**-0.836**	**5.257**	**4.558**
Aporcelaimellus	Pungentus	**0.548**	**0.263**	**5.257**	**4.558**
Aporcelaimellus	Thornematidae	**0.548**	**-0.470**	**5.257**	**4.859**
Aporcelaimellus	Scutacarus	**0.548**	**-0.608**	**5.257**	**2.849**
Aporcelaimellus	Tarsonemus	**0.548**	**-0.701**	**5.257**	**2.371**
Dorylaimoidea	Mononchus	**-0.604**	**-0.938**	**5.160**	**4.558**
Dorylaimoidea	Mylonchulus	**-0.604**	**-0.005**	**5.160**	**4.558**

Dorylaimoidea	Tripyla	**-0.604**	**-0.420**	**5.160**	**5.035**
Dorylaimoidea	Cheiroseius	**-0.604**	**0.356**	**5.160**	**2.371**
Dorylaimoidea	Dendrolaelaps	**-0.604**	**0.027**	**5.160**	**2.371**
Dorylaimoidea	Lysigamasus	**-0.604**	**0.407**	**5.160**	**2.371**
Dorylaimoidea	Pachylaelaps	**-0.604**	**0.375**	**5.160**	**2.371**
Dorylaimoidea	Veigaia	**-0.604**	**0.613**	**5.160**	**2.371**
Dorylaimoidea	Aporcelaimellus	**-0.604**	**0.548**	**5.160**	**5.257**
Dorylaimoidea	Dorylaimoidea	**-0.604**	**-0.604**	**5.160**	**5.160**
Dorylaimoidea	Epidorylaimus	**-0.604**	**0.199**	**5.160**	**4.558**
Dorylaimoidea	Eudorylaimus	**-0.604**	**-0.166**	**5.160**	**4.859**
Dorylaimoidea	Mesodorylaimus	**-0.604**	**-0.277**	**5.160**	**4.859**
Dorylaimoidea	Prodorylaimus	**-0.604**	**-0.836**	**5.160**	**4.558**
Dorylaimoidea	Pungentus	**-0.604**	**0.263**	**5.160**	**4.558**
Dorylaimoidea	Thornematidae	**-0.604**	**-0.470**	**5.160**	**4.859**
Dorylaimoidea	Scutacarus	**-0.604**	**-0.608**	**5.160**	**2.849**
Dorylaimoidea	Tarsonemus	**-0.604**	**-0.701**	**5.160**	**2.371**
Epidorylaimus	Mononchus	**0.199**	**-0.938**	**4.558**	**4.558**
Epidorylaimus	Mylonchulus	**0.199**	**-0.005**	**4.558**	**4.558**
Epidorylaimus	Tripyla	**0.199**	**-0.420**	**4.558**	**5.035**
Epidorylaimus	Cheiroseius	**0.199**	**0.356**	**4.558**	**2.371**
Epidorylaimus	Dendrolaelaps	**0.199**	**0.027**	**4.558**	**2.371**
Epidorylaimus	Lysigamasus	**0.199**	**0.407**	**4.558**	**2.371**
Epidorylaimus	Pachylaelaps	**0.199**	**0.375**	**4.558**	**2.371**
Epidorylaimus	Veigaia	**0.199**	**0.613**	**4.558**	**2.371**
Epidorylaimus	Aporcelaimellus	**0.199**	**0.548**	**4.558**	**5.257**
Epidorylaimus	Dorylaimoidea	**0.199**	**-0.604**	**4.558**	**5.160**
Epidorylaimus	Epidorylaimus	**0.199**	**0.199**	**4.558**	**4.558**
Epidorylaimus	Eudorylaimus	**0.199**	**-0.166**	**4.558**	**4.859**
Epidorylaimus	Mesodorylaimus	**0.199**	**-0.277**	**4.558**	**4.859**
Epidorylaimus	Prodorylaimus	**0.199**	**-0.836**	**4.558**	**4.558**
Epidorylaimus	Pungentus	**0.199**	**0.263**	**4.558**	**4.558**
Epidorylaimus	Thornematidae	**0.199**	**-0.470**	**4.558**	**4.859**
Epidorylaimus	Scutacarus	**0.199**	**-0.608**	**4.558**	**2.849**
Epidorylaimus	Tarsonemus	**0.199**	**-0.701**	**4.558**	**2.371**
Eudorylaimus	Mononchus	**-0.166**	**-0.938**	**4.859**	**4.558**
Eudorylaimus	Mylonchulus	**-0.166**	**-0.005**	**4.859**	**4.558**
Eudorylaimus	Tripyla	**-0.166**	**-0.420**	**4.859**	**5.035**
Eudorylaimus	Cheiroseius	**-0.166**	**0.356**	**4.859**	**2.371**
Eudorylaimus	Dendrolaelaps	**-0.166**	**0.027**	**4.859**	**2.371**
Eudorylaimus	Lysigamasus	**-0.166**	**0.407**	**4.859**	**2.371**
Eudorylaimus	Pachylaelaps	**-0.166**	**0.375**	**4.859**	**2.371**
Eudorylaimus	Veigaia	**-0.166**	**0.613**	**4.859**	**2.371**
Eudorylaimus	Aporcelaimellus	**-0.166**	**0.548**	**4.859**	**5.257**
Eudorylaimus	Dorylaimoidea	**-0.166**	**-0.604**	**4.859**	**5.160**
Eudorylaimus	Epidorylaimus	**-0.166**	**0.199**	**4.859**	**4.558**
Eudorylaimus	Eudorylaimus	**-0.166**	**-0.166**	**4.859**	**4.859**
Eudorylaimus	Mesodorylaimus	**-0.166**	**-0.277**	**4.859**	**4.859**
Eudorylaimus	Prodorylaimus	**-0.166**	**-0.836**	**4.859**	**4.558**
Eudorylaimus	Pungentus	**-0.166**	**0.263**	**4.859**	**4.558**
Eudorylaimus	Thornematidae	**-0.166**	**-0.470**	**4.859**	**4.859**
Eudorylaimus	Scutacarus	**-0.166**	**-0.608**	**4.859**	**2.849**

Eudorylaimus	Tarsonemus	**-0.166**	**-0.701**	**4.859**	**2.371**
Mesodorylaimus	Mononchus	**-0.277**	**-0.938**	**4.859**	**4.558**
Mesodorylaimus	Mylonchulus	**-0.277**	**-0.005**	**4.859**	**4.558**
Mesodorylaimus	Tripyla	**-0.277**	**-0.420**	**4.859**	**5.035**
Mesodorylaimus	Cheiroseius	**-0.277**	**0.356**	**4.859**	**2.371**
Mesodorylaimus	Dendrolaelaps	**-0.277**	**0.027**	**4.859**	**2.371**
Mesodorylaimus	Lysigamasus	**-0.277**	**0.407**	**4.859**	**2.371**
Mesodorylaimus	Pachylaelaps	**-0.277**	**0.375**	**4.859**	**2.371**
Mesodorylaimus	Veigaia	**-0.277**	**0.613**	**4.859**	**2.371**
Mesodorylaimus	Aporcelaimellus	**-0.277**	**0.548**	**4.859**	**5.257**
Mesodorylaimus	Dorylaimoidea	**-0.277**	**-0.604**	**4.859**	**5.160**
Mesodorylaimus	Epidorylaimus	**-0.277**	**0.199**	**4.859**	**4.558**
Mesodorylaimus	Eudorylaimus	**-0.277**	**-0.166**	**4.859**	**4.859**
Mesodorylaimus	Mesodorylaimus	**-0.277**	**-0.277**	**4.859**	**4.859**
Mesodorylaimus	Prodorylaimus	**-0.277**	**-0.836**	**4.859**	**4.558**
Mesodorylaimus	Pungentus	**-0.277**	**0.263**	**4.859**	**4.558**
Mesodorylaimus	Thornematidae	**-0.277**	**-0.470**	**4.859**	**4.859**
Mesodorylaimus	Scutacarus	**-0.277**	**-0.608**	**4.859**	**2.849**
Mesodorylaimus	Tarsonemus	**-0.277**	**-0.701**	**4.859**	**2.371**
Prodorylaimus	Mononchus	**-0.836**	**-0.938**	**4.558**	**4.558**
Prodorylaimus	Mylonchulus	**-0.836**	**-0.005**	**4.558**	**4.558**
Prodorylaimus	Tripyla	**-0.836**	**-0.420**	**4.558**	**5.035**
Prodorylaimus	Cheiroseius	**-0.836**	**0.356**	**4.558**	**2.371**
Prodorylaimus	Dendrolaelaps	**-0.836**	**0.027**	**4.558**	**2.371**
Prodorylaimus	Lysigamasus	**-0.836**	**0.407**	**4.558**	**2.371**
Prodorylaimus	Pachylaelaps	**-0.836**	**0.375**	**4.558**	**2.371**
Prodorylaimus	Veigaia	**-0.836**	**0.613**	**4.558**	**2.371**
Prodorylaimus	Aporcelaimellus	**-0.836**	**0.548**	**4.558**	**5.257**
Prodorylaimus	Dorylaimoidea	**-0.836**	**-0.604**	**4.558**	**5.160**
Prodorylaimus	Epidorylaimus	**-0.836**	**0.199**	**4.558**	**4.558**
Prodorylaimus	Eudorylaimus	**-0.836**	**-0.166**	**4.558**	**4.859**
Prodorylaimus	Mesodorylaimus	**-0.836**	**-0.277**	**4.558**	**4.859**
Prodorylaimus	Prodorylaimus	**-0.836**	**-0.836**	**4.558**	**4.558**
Prodorylaimus	Pungentus	**-0.836**	**0.263**	**4.558**	**4.558**
Prodorylaimus	Thornematidae	**-0.836**	**-0.470**	**4.558**	**4.859**
Prodorylaimus	Scutacarus	**-0.836**	**-0.608**	**4.558**	**2.849**
Prodorylaimus	Tarsonemus	**-0.836**	**-0.701**	**4.558**	**2.371**
Pungentus	Mononchus	**0.263**	**-0.938**	**4.558**	**4.558**
Pungentus	Mylonchulus	**0.263**	**-0.005**	**4.558**	**4.558**
Pungentus	Tripyla	**0.263**	**-0.420**	**4.558**	**5.035**
Pungentus	Cheiroseius	**0.263**	**0.356**	**4.558**	**2.371**
Pungentus	Dendrolaelaps	**0.263**	**0.027**	**4.558**	**2.371**
Pungentus	Lysigamasus	**0.263**	**0.407**	**4.558**	**2.371**
Pungentus	Pachylaelaps	**0.263**	**0.375**	**4.558**	**2.371**
Pungentus	Veigaia	**0.263**	**0.613**	**4.558**	**2.371**
Pungentus	Aporcelaimellus	**0.263**	**0.548**	**4.558**	**5.257**
Pungentus	Dorylaimoidea	**0.263**	**-0.604**	**4.558**	**5.160**
Pungentus	Epidorylaimus	**0.263**	**0.199**	**4.558**	**4.558**
Pungentus	Eudorylaimus	**0.263**	**-0.166**	**4.558**	**4.859**
Pungentus	Mesodorylaimus	**0.263**	**-0.277**	**4.558**	**4.859**
Pungentus	Prodorylaimus	**0.263**	**-0.836**	**4.558**	**4.558**

Pungentus	Pungentus	**0.263**	**0.263**	**4.558**	**4.558**
Pungentus	Thornematidae	**0.263**	**-0.470**	**4.558**	**4.859**
Pungentus	Scutacarus	**0.263**	**-0.608**	**4.558**	**2.849**
Pungentus	Tarsonemus	**0.263**	**-0.701**	**4.558**	**2.371**
Thornematidae	Mononchus	**-0.470**	**-0.938**	**4.859**	**4.558**
Thornematidae	Mylonchulus	**-0.470**	**-0.005**	**4.859**	**4.558**
Thornematidae	Tripyla	**-0.470**	**-0.420**	**4.859**	**5.035**
Thornematidae	Cheiroseius	**-0.470**	**0.356**	**4.859**	**2.371**
Thornematidae	Dendrolaelaps	**-0.470**	**0.027**	**4.859**	**2.371**
Thornematidae	Lysigamasus	**-0.470**	**0.407**	**4.859**	**2.371**
Thornematidae	Pachylaelaps	**-0.470**	**0.375**	**4.859**	**2.371**
Thornematidae	Veigaia	**-0.470**	**0.613**	**4.859**	**2.371**
Thornematidae	Aporcelaimellus	**-0.470**	**0.548**	**4.859**	**5.257**
Thornematidae	Dorylaimoidea	**-0.470**	**-0.604**	**4.859**	**5.160**
Thornematidae	Epidorylaimus	**-0.470**	**0.199**	**4.859**	**4.558**
Thornematidae	Eudorylaimus	**-0.470**	**-0.166**	**4.859**	**4.859**
Thornematidae	Mesodorylaimus	**-0.470**	**-0.277**	**4.859**	**4.859**
Thornematidae	Prodorylaimus	**-0.470**	**-0.836**	**4.859**	**4.558**
Thornematidae	Pungentus	**-0.470**	**0.263**	**4.859**	**4.558**
Thornematidae	Thornematidae	**-0.470**	**-0.470**	**4.859**	**4.859**
Thornematidae	Scutacarus	**-0.470**	**-0.608**	**4.859**	**2.849**
Thornematidae	Tarsonemus	**-0.470**	**-0.701**	**4.859**	**2.371**
Scutacarus	Bdella	**-0.608**	**0.816**	**2.849**	**2.371**
Scutacarus	Cheiroseius	**-0.608**	**0.356**	**2.849**	**2.371**
Scutacarus	Dendrolaelaps	**-0.608**	**0.027**	**2.849**	**2.371**
Scutacarus	Lysigamasus	**-0.608**	**0.407**	**2.849**	**2.371**
Scutacarus	Pachylaelaps	**-0.608**	**0.375**	**2.849**	**2.371**
Scutacarus	Veigaia	**-0.608**	**0.613**	**2.849**	**2.371**
Scutacarus	Aporcelaimellus	**-0.608**	**0.548**	**2.849**	**5.257**
Scutacarus	Dorylaimoidea	**-0.608**	**-0.604**	**2.849**	**5.160**
Scutacarus	Epidorylaimus	**-0.608**	**0.199**	**2.849**	**4.558**
Scutacarus	Eudorylaimus	**-0.608**	**-0.166**	**2.849**	**4.859**
Scutacarus	Mesodorylaimus	**-0.608**	**-0.277**	**2.849**	**4.859**
Scutacarus	Prodorylaimus	**-0.608**	**-0.836**	**2.849**	**4.558**
Scutacarus	Pungentus	**-0.608**	**0.263**	**2.849**	**4.558**
Scutacarus	Thornematidae	**-0.608**	**-0.470**	**2.849**	**4.859**
Scutacarus	Scutacarus	**-0.608**	**-0.608**	**2.849**	**2.849**
Scutacarus	Tarsonemus	**-0.608**	**-0.701**	**2.849**	**2.371**
Tarsonemus	Bdella	**-0.701**	**0.816**	**2.371**	**2.371**
Tarsonemus	Cheiroseius	**-0.701**	**0.356**	**2.371**	**2.371**
Tarsonemus	Dendrolaelaps	**-0.701**	**0.027**	**2.371**	**2.371**
Tarsonemus	Lysigamasus	**-0.701**	**0.407**	**2.371**	**2.371**
Tarsonemus	Pachylaelaps	**-0.701**	**0.375**	**2.371**	**2.371**
Tarsonemus	Veigaia	**-0.701**	**0.613**	**2.371**	**2.371**
Tarsonemus	Aporcelaimellus	**-0.701**	**0.548**	**2.371**	**5.257**
Tarsonemus	Dorylaimoidea	**-0.701**	**-0.604**	**2.371**	**5.160**
Tarsonemus	Epidorylaimus	**-0.701**	**0.199**	**2.371**	**4.558**
Tarsonemus	Eudorylaimus	**-0.701**	**-0.166**	**2.371**	**4.859**
Tarsonemus	Mesodorylaimus	**-0.701**	**-0.277**	**2.371**	**4.859**
Tarsonemus	Prodorylaimus	**-0.701**	**-0.836**	**2.371**	**4.558**
Tarsonemus	Pungentus	**-0.701**	**0.263**	**2.371**	**4.558**

Tarsonemus	Thornematidae	**-0.701**	**-0.470**	**2.371**	**4.859**
Tarsonemus	Scutacarus	**-0.701**	**-0.608**	**2.371**	**2.849**
Tarsonemus	Tarsonemus	**-0.701**	**-0.701**	**2.371**	**2.371**

Resource	Consumer	Mres	Mconsumer	Nres	Nconsumer
Dolichodoridae	Tripyla	**-0.885**	**-0.420**	**5.366**	**4.044**
Dolichodoridae	Alliphis	**-0.885**	**0.053**	**5.366**	**3.407**
Dolichodoridae	Arctoseius	**-0.885**	**-0.152**	**5.366**	**2.453**
Dolichodoridae	Dendrolaelaps	**-0.885**	**0.027**	**5.366**	**2.821**
Dolichodoridae	Dendroseius	**-0.885**	**-0.310**	**5.366**	**1.976**
Dolichodoridae	Lasioseius	**-0.885**	**0.114**	**5.366**	**1.976**
Dolichodoridae	Lysigamasus	**-0.885**	**0.407**	**5.366**	**1.976**
Dolichodoridae	Parasitus	**-0.885**	**0.859**	**5.366**	**2.277**
Dolichodoridae	Pergamasus	**-0.885**	**1.081**	**5.366**	**1.976**
Dolichodoridae	Rhodacarellus	**-0.885**	**-0.310**	**5.366**	**2.930**
Dolichodoridae	Aporcelaimellus	**-0.885**	**0.548**	**5.366**	**4.889**
Dolichodoridae	Dorylaimoidea	**-0.885**	**-0.604**	**5.366**	**4.345**
Dolichodoridae	Qudsianematidae	**-0.885**	**-0.207**	**5.366**	**4.044**
Dolichodoridae	Eupodes	**-0.885**	**0.005**	**5.366**	**3.090**
Dolichodoridae	Mesostigmata	**-0.885**	**-0.411**	**5.366**	**2.277**
Dolichodoridae	Scutacarus	**-0.885**	**-0.608**	**5.366**	**3.481**
Dolichodoridae	Stigmaeidae	**-0.885**	**0.229**	**5.366**	**2.578**
Helicotylenchus	Tripyla	**-0.792**	**-0.420**	**4.822**	**4.044**
Helicotylenchus	Alliphis	**-0.792**	**0.053**	**4.822**	**3.407**
Helicotylenchus	Arctoseius	**-0.792**	**-0.152**	**4.822**	**2.453**
Helicotylenchus	Dendrolaelaps	**-0.792**	**0.027**	**4.822**	**2.821**
Helicotylenchus	Dendroseius	**-0.792**	**-0.310**	**4.822**	**1.976**
Helicotylenchus	Lasioseius	**-0.792**	**0.114**	**4.822**	**1.976**
Helicotylenchus	Lysigamasus	**-0.792**	**0.407**	**4.822**	**1.976**
Helicotylenchus	Parasitus	**-0.792**	**0.859**	**4.822**	**2.277**
Helicotylenchus	Pergamasus	**-0.792**	**1.081**	**4.822**	**1.976**
Helicotylenchus	Rhodacarellus	**-0.792**	**-0.310**	**4.822**	**2.930**
Helicotylenchus	Aporcelaimellus	**-0.792**	**0.548**	**4.822**	**4.889**
Helicotylenchus	Dorylaimoidea	**-0.792**	**-0.604**	**4.822**	**4.345**
Helicotylenchus	Qudsianematidae	**-0.792**	**-0.207**	**4.822**	**4.044**
Helicotylenchus	Eupodes	**-0.792**	**0.005**	**4.822**	**3.090**
Helicotylenchus	Mesostigmata	**-0.792**	**-0.411**	**4.822**	**2.277**
Helicotylenchus	Scutacarus	**-0.792**	**-0.608**	**4.822**	**3.481**
Helicotylenchus	Stigmaeidae	**-0.792**	**0.229**	**4.822**	**2.578**
Hoplolaimidae	Tripyla	**-1.090**	**-0.420**	**4.044**	**4.044**
Hoplolaimidae	Alliphis	**-1.090**	**0.053**	**4.044**	**3.407**
Hoplolaimidae	Arctoseius	**-1.090**	**-0.152**	**4.044**	**2.453**
Hoplolaimidae	Dendrolaelaps	**-1.090**	**0.027**	**4.044**	**2.821**
Hoplolaimidae	Dendroseius	**-1.090**	**-0.310**	**4.044**	**1.976**
Hoplolaimidae	Lasioseius	**-1.090**	**0.114**	**4.044**	**1.976**
Hoplolaimidae	Lysigamasus	**-1.090**	**0.407**	**4.044**	**1.976**
Hoplolaimidae	Parasitus	**-1.090**	**0.859**	**4.044**	**2.277**
Hoplolaimidae	Pergamasus	**-1.090**	**1.081**	**4.044**	**1.976**
Hoplolaimidae	Rhodacarellus	**-1.090**	**-0.310**	**4.044**	**2.930**
Hoplolaimidae	Aporcelaimellus	**-1.090**	**0.548**	**4.044**	**4.889**
Hoplolaimidae	Dorylaimoidea	**-1.090**	**-0.604**	**4.044**	**4.345**
Hoplolaimidae	Qudsianematidae	**-1.090**	**-0.207**	**4.044**	**4.044**
Hoplolaimidae	Eupodes	**-1.090**	**0.005**	**4.044**	**3.090**
Hoplolaimidae	Mesostigmata	**-1.090**	**-0.411**	**4.044**	**2.277**
Hoplolaimidae	Scutacarus	**-1.090**	**-0.608**	**4.044**	**3.481**

Hoplolaimidae	Stigmaeidae	**-1.090**	**0.229**	**4.044**	**2.578**
Malenchus	Tripyla	**-1.330**	**-0.420**	**4.646**	**4.044**
Malenchus	Alliphis	**-1.330**	**0.053**	**4.646**	**3.407**
Malenchus	Arctoseius	**-1.330**	**-0.152**	**4.646**	**2.453**
Malenchus	Dendrolaelaps	**-1.330**	**0.027**	**4.646**	**2.821**
Malenchus	Dendroseius	**-1.330**	**-0.310**	**4.646**	**1.976**
Malenchus	Lasioseius	**-1.330**	**0.114**	**4.646**	**1.976**
Malenchus	Lysigamasus	**-1.330**	**0.407**	**4.646**	**1.976**
Malenchus	Parasitus	**-1.330**	**0.859**	**4.646**	**2.277**
Malenchus	Pergamasus	**-1.330**	**1.081**	**4.646**	**1.976**
Malenchus	Rhodacarellus	**-1.330**	**-0.310**	**4.646**	**2.930**
Malenchus	Aporcelaimellus	**-1.330**	**0.548**	**4.646**	**4.889**
Malenchus	Dorylaimoidea	**-1.330**	**-0.604**	**4.646**	**4.345**
Malenchus	Qudsianematidae	**-1.330**	**-0.207**	**4.646**	**4.044**
Malenchus	Eupodes	**-1.330**	**0.005**	**4.646**	**3.090**
Malenchus	Mesostigmata	**-1.330**	**-0.411**	**4.646**	**2.277**
Malenchus	Scutacarus	**-1.330**	**-0.608**	**4.646**	**3.481**
Malenchus	Stigmaeidae	**-1.330**	**0.229**	**4.646**	**2.578**
Paratylenchus	Tripyla	**-1.244**	**-0.420**	**4.646**	**4.044**
Paratylenchus	Alliphis	**-1.244**	**0.053**	**4.646**	**3.407**
Paratylenchus	Arctoseius	**-1.244**	**-0.152**	**4.646**	**2.453**
Paratylenchus	Dendrolaelaps	**-1.244**	**0.027**	**4.646**	**2.821**
Paratylenchus	Dendroseius	**-1.244**	**-0.310**	**4.646**	**1.976**
Paratylenchus	Lasioseius	**-1.244**	**0.114**	**4.646**	**1.976**
Paratylenchus	Lysigamasus	**-1.244**	**0.407**	**4.646**	**1.976**
Paratylenchus	Parasitus	**-1.244**	**0.859**	**4.646**	**2.277**
Paratylenchus	Pergamasus	**-1.244**	**1.081**	**4.646**	**1.976**
Paratylenchus	Rhodacarellus	**-1.244**	**-0.310**	**4.646**	**2.930**
Paratylenchus	Aporcelaimellus	**-1.244**	**0.548**	**4.646**	**4.889**
Paratylenchus	Dorylaimoidea	**-1.244**	**-0.604**	**4.646**	**4.345**
Paratylenchus	Qudsianematidae	**-1.244**	**-0.207**	**4.646**	**4.044**
Paratylenchus	Eupodes	**-1.244**	**0.005**	**4.646**	**3.090**
Paratylenchus	Mesostigmata	**-1.244**	**-0.411**	**4.646**	**2.277**
Paratylenchus	Scutacarus	**-1.244**	**-0.608**	**4.646**	**3.481**
Paratylenchus	Stigmaeidae	**-1.244**	**0.229**	**4.646**	**2.578**
Pratylenchus	Tripyla	**-1.226**	**-0.420**	**4.044**	**4.044**
Pratylenchus	Alliphis	**-1.226**	**0.053**	**4.044**	**3.407**
Pratylenchus	Arctoseius	**-1.226**	**-0.152**	**4.044**	**2.453**
Pratylenchus	Dendrolaelaps	**-1.226**	**0.027**	**4.044**	**2.821**
Pratylenchus	Dendroseius	**-1.226**	**-0.310**	**4.044**	**1.976**
Pratylenchus	Lasioseius	**-1.226**	**0.114**	**4.044**	**1.976**
Pratylenchus	Lysigamasus	**-1.226**	**0.407**	**4.044**	**1.976**
Pratylenchus	Parasitus	**-1.226**	**0.859**	**4.044**	**2.277**
Pratylenchus	Pergamasus	**-1.226**	**1.081**	**4.044**	**1.976**
Pratylenchus	Rhodacarellus	**-1.226**	**-0.310**	**4.044**	**2.930**
Pratylenchus	Aporcelaimellus	**-1.226**	**0.548**	**4.044**	**4.889**
Pratylenchus	Dorylaimoidea	**-1.226**	**-0.604**	**4.044**	**4.345**
Pratylenchus	Qudsianematidae	**-1.226**	**-0.207**	**4.044**	**4.044**
Pratylenchus	Eupodes	**-1.226**	**0.005**	**4.044**	**3.090**
Pratylenchus	Mesostigmata	**-1.226**	**-0.411**	**4.044**	**2.277**
Pratylenchus	Scutacarus	**-1.226**	**-0.608**	**4.044**	**3.481**

Pratylenchus	Stigmaeidae	**-1.226**	**0.229**	**4.044**	**2.578**
Trichodorus	Tripyla	**-0.744**	**-0.420**	**4.521**	**4.044**
Trichodorus	Alliphis	**-0.744**	**0.053**	**4.521**	**3.407**
Trichodorus	Arctoseius	**-0.744**	**-0.152**	**4.521**	**2.453**
Trichodorus	Dendrolaelaps	**-0.744**	**0.027**	**4.521**	**2.821**
Trichodorus	Dendroseius	**-0.744**	**-0.310**	**4.521**	**1.976**
Trichodorus	Lasioseius	**-0.744**	**0.114**	**4.521**	**1.976**
Trichodorus	Lysigamasus	**-0.744**	**0.407**	**4.521**	**1.976**
Trichodorus	Parasitus	**-0.744**	**0.859**	**4.521**	**2.277**
Trichodorus	Pergamasus	**-0.744**	**1.081**	**4.521**	**1.976**
Trichodorus	Rhodacarellus	**-0.744**	**-0.310**	**4.521**	**2.930**
Trichodorus	Aporcelaimellus	**-0.744**	**0.548**	**4.521**	**4.889**
Trichodorus	Dorylaimoidea	**-0.744**	**-0.604**	**4.521**	**4.345**
Trichodorus	Qudsianematidae	**-0.744**	**-0.207**	**4.521**	**4.044**
Trichodorus	Eupodes	**-0.744**	**0.005**	**4.521**	**3.090**
Trichodorus	Mesostigmata	**-0.744**	**-0.411**	**4.521**	**2.277**
Trichodorus	Scutacarus	**-0.744**	**-0.608**	**4.521**	**3.481**
Trichodorus	Stigmaeidae	**-0.744**	**0.229**	**4.521**	**2.578**
Tylenchorhynchus	Tripyla	**-0.664**	**-0.420**	**4.646**	**4.044**
Tylenchorhynchus	Alliphis	**-0.664**	**0.053**	**4.646**	**3.407**
Tylenchorhynchus	Arctoseius	**-0.664**	**-0.152**	**4.646**	**2.453**
Tylenchorhynchus	Dendrolaelaps	**-0.664**	**0.027**	**4.646**	**2.821**
Tylenchorhynchus	Dendroseius	**-0.664**	**-0.310**	**4.646**	**1.976**
Tylenchorhynchus	Lasioseius	**-0.664**	**0.114**	**4.646**	**1.976**
Tylenchorhynchus	Lysigamasus	**-0.664**	**0.407**	**4.646**	**1.976**
Tylenchorhynchus	Parasitus	**-0.664**	**0.859**	**4.646**	**2.277**
Tylenchorhynchus	Pergamasus	**-0.664**	**1.081**	**4.646**	**1.976**
Tylenchorhynchus	Rhodacarellus	**-0.664**	**-0.310**	**4.646**	**2.930**
Tylenchorhynchus	Aporcelaimellus	**-0.664**	**0.548**	**4.646**	**4.889**
Tylenchorhynchus	Dorylaimoidea	**-0.664**	**-0.604**	**4.646**	**4.345**
Tylenchorhynchus	Qudsianematidae	**-0.664**	**-0.207**	**4.646**	**4.044**
Tylenchorhynchus	Eupodes	**-0.664**	**0.005**	**4.646**	**3.090**
Tylenchorhynchus	Mesostigmata	**-0.664**	**-0.411**	**4.646**	**2.277**
Tylenchorhynchus	Scutacarus	**-0.664**	**-0.608**	**4.646**	**3.481**
Tylenchorhynchus	Stigmaeidae	**-0.664**	**0.229**	**4.646**	**2.578**
Pachygnatidae	Bdella	**-0.113**	**0.816**	**2.821**	**1.976**
Pachygnatidae	Arctoseius	**-0.113**	**-0.152**	**2.821**	**2.453**
Pachygnatidae	Dendrolaelaps	**-0.113**	**0.027**	**2.821**	**2.821**
Pachygnatidae	Dendroseius	**-0.113**	**-0.310**	**2.821**	**1.976**
Pachygnatidae	Lasioseius	**-0.113**	**0.114**	**2.821**	**1.976**
Pachygnatidae	Lysigamasus	**-0.113**	**0.407**	**2.821**	**1.976**
Pachygnatidae	Parasitus	**-0.113**	**0.859**	**2.821**	**2.277**
Pachygnatidae	Pergamasus	**-0.113**	**1.081**	**2.821**	**1.976**
Pachygnatidae	Rhodacarellus	**-0.113**	**-0.310**	**2.821**	**2.930**
Pachygnatidae	Aporcelaimellus	**-0.113**	**0.548**	**2.821**	**4.889**
Pachygnatidae	Dorylaimoidea	**-0.113**	**-0.604**	**2.821**	**4.345**
Pachygnatidae	Qudsianematidae	**-0.113**	**-0.207**	**2.821**	**4.044**
Pachygnatidae	Eupodes	**-0.113**	**0.005**	**2.821**	**3.090**
Pachygnatidae	Mesostigmata	**-0.113**	**-0.411**	**2.821**	**2.277**
Pachygnatidae	Scutacarus	**-0.113**	**-0.608**	**2.821**	**3.481**
Pachygnatidae	Stigmaeidae	**-0.113**	**0.229**	**2.821**	**2.578**

Rhizoglyphus	Bdella	**0.005**	**0.816**	**1.976**	**1.976**
Rhizoglyphus	Arctoseius	**0.005**	**-0.152**	**1.976**	**2.453**
Rhizoglyphus	Dendrolaelaps	**0.005**	**0.027**	**1.976**	**2.821**
Rhizoglyphus	Dendroseius	**0.005**	**-0.310**	**1.976**	**1.976**
Rhizoglyphus	Lasioseius	**0.005**	**0.114**	**1.976**	**1.976**
Rhizoglyphus	Lysigamasus	**0.005**	**0.407**	**1.976**	**1.976**
Rhizoglyphus	Parasitus	**0.005**	**0.859**	**1.976**	**2.277**
Rhizoglyphus	Pergamasus	**0.005**	**1.081**	**1.976**	**1.976**
Rhizoglyphus	Rhodacarellus	**0.005**	**-0.310**	**1.976**	**2.930**
Rhizoglyphus	Aporcelaimellus	**0.005**	**0.548**	**1.976**	**4.889**
Rhizoglyphus	Dorylaimoidea	**0.005**	**-0.604**	**1.976**	**4.345**
Rhizoglyphus	Qudsianematidae	**0.005**	**-0.207**	**1.976**	**4.044**
Rhizoglyphus	Eupodes	**0.005**	**0.005**	**1.976**	**3.090**
Rhizoglyphus	Mesostigmata	**0.005**	**-0.411**	**1.976**	**2.277**
Rhizoglyphus	Scutacarus	**0.005**	**-0.608**	**1.976**	**3.481**
Rhizoglyphus	Stigmaeidae	**0.005**	**0.229**	**1.976**	**2.578**
Tydeidae	Bdella	**-0.608**	**0.816**	**2.976**	**1.976**
Tydeidae	Arctoseius	**-0.608**	**-0.152**	**2.976**	**2.453**
Tydeidae	Dendrolaelaps	**-0.608**	**0.027**	**2.976**	**2.821**
Tydeidae	Dendroseius	**-0.608**	**-0.310**	**2.976**	**1.976**
Tydeidae	Lasioseius	**-0.608**	**0.114**	**2.976**	**1.976**
Tydeidae	Lysigamasus	**-0.608**	**0.407**	**2.976**	**1.976**
Tydeidae	Parasitus	**-0.608**	**0.859**	**2.976**	**2.277**
Tydeidae	Pergamasus	**-0.608**	**1.081**	**2.976**	**1.976**
Tydeidae	Rhodacarellus	**-0.608**	**-0.310**	**2.976**	**2.930**
Tydeidae	Aporcelaimellus	**-0.608**	**0.548**	**2.976**	**4.889**
Tydeidae	Dorylaimoidea	**-0.608**	**-0.604**	**2.976**	**4.345**
Tydeidae	Qudsianematidae	**-0.608**	**-0.207**	**2.976**	**4.044**
Tydeidae	Eupodes	**-0.608**	**0.005**	**2.976**	**3.090**
Tydeidae	Mesostigmata	**-0.608**	**-0.411**	**2.976**	**2.277**
Tydeidae	Scutacarus	**-0.608**	**-0.608**	**2.976**	**3.481**
Tydeidae	Stigmaeidae	**-0.608**	**0.229**	**2.976**	**2.578**
Sminthuridae	Arctoseius	**-0.608**	**-0.152**	**2.277**	**2.453**
Sminthuridae	Dendrolaelaps	**-0.608**	**0.027**	**2.277**	**2.821**
Sminthuridae	Dendroseius	**-0.608**	**-0.310**	**2.277**	**1.976**
Sminthuridae	Lasioseius	**-0.608**	**0.114**	**2.277**	**1.976**
Sminthuridae	Lysigamasus	**-0.608**	**0.407**	**2.277**	**1.976**
Sminthuridae	Parasitus	**-0.608**	**0.859**	**2.277**	**2.277**
Sminthuridae	Pergamasus	**-0.608**	**1.081**	**2.277**	**1.976**
Sminthuridae	Rhodacarellus	**-0.608**	**-0.310**	**2.277**	**2.930**
Sminthuridae	Aporcelaimellus	**-0.608**	**0.548**	**2.277**	**4.889**
Sminthuridae	Dorylaimoidea	**-0.608**	**-0.604**	**2.277**	**4.345**
Sminthuridae	Qudsianematidae	**-0.608**	**-0.207**	**2.277**	**4.044**
Sminthuridae	Eupodes	**-0.608**	**0.005**	**2.277**	**3.090**
Sminthuridae	Mesostigmata	**-0.608**	**-0.411**	**2.277**	**2.277**
Sminthuridae	Scutacarus	**-0.608**	**-0.608**	**2.277**	**3.481**
Sminthuridae	Stigmaeidae	**-0.608**	**0.229**	**2.277**	**2.578**
Sminthurinus	Arctoseius	**0.618**	**-0.152**	**3.277**	**2.453**
Sminthurinus	Dendrolaelaps	**0.618**	**0.027**	**3.277**	**2.821**
Sminthurinus	Dendroseius	**0.618**	**-0.310**	**3.277**	**1.976**
Sminthurinus	Lasioseius	**0.618**	**0.114**	**3.277**	**1.976**

Sminthurinus	Lysigamasus	**0.618**	**0.407**	**3.277**	**1.976**
Sminthurinus	Parasitus	**0.618**	**0.859**	**3.277**	**2.277**
Sminthurinus	Pergamasus	**0.618**	**1.081**	**3.277**	**1.976**
Sminthurinus	Rhodacarellus	**0.618**	**-0.310**	**3.277**	**2.930**
Sminthurinus	Aporcelaimellus	**0.618**	**0.548**	**3.277**	**4.889**
Sminthurinus	Dorylaimoidea	**0.618**	**-0.604**	**3.277**	**4.345**
Sminthurinus	Qudsianematidae	**0.618**	**-0.207**	**3.277**	**4.044**
Sminthurinus	Eupodes	**0.618**	**0.005**	**3.277**	**3.090**
Sminthurinus	Mesostigmata	**0.618**	**-0.411**	**3.277**	**2.277**
Sminthurinus	Scutacarus	**0.618**	**-0.608**	**3.277**	**3.481**
Sminthurinus	Stigmaeidae	**0.618**	**0.229**	**3.277**	**2.578**
Sphaeridia	Arctoseius	**0.202**	**-0.152**	**3.017**	**2.453**
Sphaeridia	Dendrolaelaps	**0.202**	**0.027**	**3.017**	**2.821**
Sphaeridia	Dendroseius	**0.202**	**-0.310**	**3.017**	**1.976**
Sphaeridia	Lasioseius	**0.202**	**0.114**	**3.017**	**1.976**
Sphaeridia	Lysigamasus	**0.202**	**0.407**	**3.017**	**1.976**
Sphaeridia	Parasitus	**0.202**	**0.859**	**3.017**	**2.277**
Sphaeridia	Pergamasus	**0.202**	**1.081**	**3.017**	**1.976**
Sphaeridia	Rhodacarellus	**0.202**	**-0.310**	**3.017**	**2.930**
Sphaeridia	Aporcelaimellus	**0.202**	**0.548**	**3.017**	**4.889**
Sphaeridia	Dorylaimoidea	**0.202**	**-0.604**	**3.017**	**4.345**
Sphaeridia	Qudsianematidae	**0.202**	**-0.207**	**3.017**	**4.044**
Sphaeridia	Eupodes	**0.202**	**0.005**	**3.017**	**3.090**
Sphaeridia	Mesostigmata	**0.202**	**-0.411**	**3.017**	**2.277**
Sphaeridia	Scutacarus	**0.202**	**-0.608**	**3.017**	**3.481**
Sphaeridia	Stigmaeidae	**0.202**	**0.229**	**3.017**	**2.578**
Aphelenchoides	Tripyla	**-1.496**	**-0.420**	**4.822**	**4.044**
Aphelenchoides	Alliphis	**-1.496**	**0.053**	**4.822**	**3.407**
Aphelenchoides	Arctoseius	**-1.496**	**-0.152**	**4.822**	**2.453**
Aphelenchoides	Dendrolaelaps	**-1.496**	**0.027**	**4.822**	**2.821**
Aphelenchoides	Dendroseius	**-1.496**	**-0.310**	**4.822**	**1.976**
Aphelenchoides	Lasioseius	**-1.496**	**0.114**	**4.822**	**1.976**
Aphelenchoides	Lysigamasus	**-1.496**	**0.407**	**4.822**	**1.976**
Aphelenchoides	Parasitus	**-1.496**	**0.859**	**4.822**	**2.277**
Aphelenchoides	Pergamasus	**-1.496**	**1.081**	**4.822**	**1.976**
Aphelenchoides	Rhodacarellus	**-1.496**	**-0.310**	**4.822**	**2.930**
Aphelenchoides	Aporcelaimellus	**-1.496**	**0.548**	**4.822**	**4.889**
Aphelenchoides	Dorylaimoidea	**-1.496**	**-0.604**	**4.822**	**4.345**
Aphelenchoides	Qudsianematidae	**-1.496**	**-0.207**	**4.822**	**4.044**
Aphelenchoides	Eupodes	**-1.496**	**0.005**	**4.822**	**3.090**
Aphelenchoides	Mesostigmata	**-1.496**	**-0.411**	**4.822**	**2.277**
Aphelenchoides	Scutacarus	**-1.496**	**-0.608**	**4.822**	**3.481**
Aphelenchoides	Stigmaeidae	**-1.496**	**0.229**	**4.822**	**2.578**
Tylenchidae	Tripyla	**-1.360**	**-0.420**	**4.743**	**4.044**
Tylenchidae	Alliphis	**-1.360**	**0.053**	**4.743**	**3.407**
Tylenchidae	Arctoseius	**-1.360**	**-0.152**	**4.743**	**2.453**
Tylenchidae	Dendrolaelaps	**-1.360**	**0.027**	**4.743**	**2.821**
Tylenchidae	Dendroseius	**-1.360**	**-0.310**	**4.743**	**1.976**
Tylenchidae	Lasioseius	**-1.360**	**0.114**	**4.743**	**1.976**
Tylenchidae	Lysigamasus	**-1.360**	**0.407**	**4.743**	**1.976**
Tylenchidae	Parasitus	**-1.360**	**0.859**	**4.743**	**2.277**

Tylenchidae	Pergamasus	**-1.360**	**1.081**	**4.743**	**1.976**
Tylenchidae	Rhodacarellus	**-1.360**	**-0.310**	**4.743**	**2.930**
Tylenchidae	Aporcelaimellus	**-1.360**	**0.548**	**4.743**	**4.889**
Tylenchidae	Dorylaimoidea	**-1.360**	**-0.604**	**4.743**	**4.345**
Tylenchidae	Qudsianematidae	**-1.360**	**-0.207**	**4.743**	**4.044**
Tylenchidae	Eupodes	**-1.360**	**0.005**	**4.743**	**3.090**
Tylenchidae	Mesostigmata	**-1.360**	**-0.411**	**4.743**	**2.277**
Tylenchidae	Scutacarus	**-1.360**	**-0.608**	**4.743**	**3.481**
Tylenchidae	Stigmaeidae	**-1.360**	**0.229**	**4.743**	**2.578**
Medioppia	Bdella	**-0.235**	**0.816**	**2.277**	**1.976**
Medioppia	Arctoseius	**-0.235**	**-0.152**	**2.277**	**2.453**
Medioppia	Dendrolaelaps	**-0.235**	**0.027**	**2.277**	**2.821**
Medioppia	Dendroseius	**-0.235**	**-0.310**	**2.277**	**1.976**
Medioppia	Lasioseius	**-0.235**	**0.114**	**2.277**	**1.976**
Medioppia	Lysigamasus	**-0.235**	**0.407**	**2.277**	**1.976**
Medioppia	Parasitus	**-0.235**	**0.859**	**2.277**	**2.277**
Medioppia	Pergamasus	**-0.235**	**1.081**	**2.277**	**1.976**
Medioppia	Rhodacarellus	**-0.235**	**-0.310**	**2.277**	**2.930**
Medioppia	Aporcelaimellus	**-0.235**	**0.548**	**2.277**	**4.889**
Medioppia	Dorylaimoidea	**-0.235**	**-0.604**	**2.277**	**4.345**
Medioppia	Qudsianematidae	**-0.235**	**-0.207**	**2.277**	**4.044**
Medioppia	Eupodes	**-0.235**	**0.005**	**2.277**	**3.090**
Medioppia	Mesostigmata	**-0.235**	**-0.411**	**2.277**	**2.277**
Medioppia	Scutacarus	**-0.235**	**-0.608**	**2.277**	**3.481**
Medioppia	Stigmaeidae	**-0.235**	**0.229**	**2.277**	**2.578**
Pygmephorus	Bdella	**-0.376**	**0.816**	**3.318**	**1.976**
Pygmephorus	Arctoseius	**-0.376**	**-0.152**	**3.318**	**2.453**
Pygmephorus	Dendrolaelaps	**-0.376**	**0.027**	**3.318**	**2.821**
Pygmephorus	Dendroseius	**-0.376**	**-0.310**	**3.318**	**1.976**
Pygmephorus	Lasioseius	**-0.376**	**0.114**	**3.318**	**1.976**
Pygmephorus	Lysigamasus	**-0.376**	**0.407**	**3.318**	**1.976**
Pygmephorus	Parasitus	**-0.376**	**0.859**	**3.318**	**2.277**
Pygmephorus	Pergamasus	**-0.376**	**1.081**	**3.318**	**1.976**
Pygmephorus	Rhodacarellus	**-0.376**	**-0.310**	**3.318**	**2.930**
Pygmephorus	Aporcelaimellus	**-0.376**	**0.548**	**3.318**	**4.889**
Pygmephorus	Dorylaimoidea	**-0.376**	**-0.604**	**3.318**	**4.345**
Pygmephorus	Qudsianematidae	**-0.376**	**-0.207**	**3.318**	**4.044**
Pygmephorus	Eupodes	**-0.376**	**0.005**	**3.318**	**3.090**
Pygmephorus	Mesostigmata	**-0.376**	**-0.411**	**3.318**	**2.277**
Pygmephorus	Scutacarus	**-0.376**	**-0.608**	**3.318**	**3.481**
Pygmephorus	Stigmaeidae	**-0.376**	**0.229**	**3.318**	**2.578**
Siteroptes	Bdella	**-0.376**	**0.816**	**1.976**	**1.976**
Siteroptes	Arctoseius	**-0.376**	**-0.152**	**1.976**	**2.453**
Siteroptes	Dendrolaelaps	**-0.376**	**0.027**	**1.976**	**2.821**
Siteroptes	Dendroseius	**-0.376**	**-0.310**	**1.976**	**1.976**
Siteroptes	Lasioseius	**-0.376**	**0.114**	**1.976**	**1.976**
Siteroptes	Lysigamasus	**-0.376**	**0.407**	**1.976**	**1.976**
Siteroptes	Parasitus	**-0.376**	**0.859**	**1.976**	**2.277**
Siteroptes	Pergamasus	**-0.376**	**1.081**	**1.976**	**1.976**
Siteroptes	Rhodacarellus	**-0.376**	**-0.310**	**1.976**	**2.930**
Siteroptes	Aporcelaimellus	**-0.376**	**0.548**	**1.976**	**4.889**

Siteroptes	Dorylaimoidea	**-0.376**	**-0.604**	**1.976**	**4.345**
Siteroptes	Qudsianematidae	**-0.376**	**-0.207**	**1.976**	**4.044**
Siteroptes	Eupodes	**-0.376**	**0.005**	**1.976**	**3.090**
Siteroptes	Mesostigmata	**-0.376**	**-0.411**	**1.976**	**2.277**
Siteroptes	Scutacarus	**-0.376**	**-0.608**	**1.976**	**3.481**
Siteroptes	Stigmaeidae	**-0.376**	**0.229**	**1.976**	**2.578**
Tyrophagus	Bdella	**0.005**	**0.816**	**2.754**	**1.976**
Tyrophagus	Arctoseius	**0.005**	**-0.152**	**2.754**	**2.453**
Tyrophagus	Dendrolaelaps	**0.005**	**0.027**	**2.754**	**2.821**
Tyrophagus	Dendroseius	**0.005**	**-0.310**	**2.754**	**1.976**
Tyrophagus	Lasioseius	**0.005**	**0.114**	**2.754**	**1.976**
Tyrophagus	Lysigamasus	**0.005**	**0.407**	**2.754**	**1.976**
Tyrophagus	Parasitus	**0.005**	**0.859**	**2.754**	**2.277**
Tyrophagus	Pergamasus	**0.005**	**1.081**	**2.754**	**1.976**
Tyrophagus	Rhodacarellus	**0.005**	**-0.310**	**2.754**	**2.930**
Tyrophagus	Aporcelaimellus	**0.005**	**0.548**	**2.754**	**4.889**
Tyrophagus	Dorylaimoidea	**0.005**	**-0.604**	**2.754**	**4.345**
Tyrophagus	Qudsianematidae	**0.005**	**-0.207**	**2.754**	**4.044**
Tyrophagus	Eupodes	**0.005**	**0.005**	**2.754**	**3.090**
Tyrophagus	Mesostigmata	**0.005**	**-0.411**	**2.754**	**2.277**
Tyrophagus	Scutacarus	**0.005**	**-0.608**	**2.754**	**3.481**
Tyrophagus	Stigmaeidae	**0.005**	**0.229**	**2.754**	**2.578**
Brachystomella	Arctoseius	**0.977**	**-0.152**	**2.976**	**2.453**
Brachystomella	Dendrolaelaps	**0.977**	**0.027**	**2.976**	**2.821**
Brachystomella	Dendroseius	**0.977**	**-0.310**	**2.976**	**1.976**
Brachystomella	Lasioseius	**0.977**	**0.114**	**2.976**	**1.976**
Brachystomella	Lysigamasus	**0.977**	**0.407**	**2.976**	**1.976**
Brachystomella	Parasitus	**0.977**	**0.859**	**2.976**	**2.277**
Brachystomella	Pergamasus	**0.977**	**1.081**	**2.976**	**1.976**
Brachystomella	Rhodacarellus	**0.977**	**-0.310**	**2.976**	**2.930**
Brachystomella	Aporcelaimellus	**0.977**	**0.548**	**2.976**	**4.889**
Brachystomella	Dorylaimoidea	**0.977**	**-0.604**	**2.976**	**4.345**
Brachystomella	Qudsianematidae	**0.977**	**-0.207**	**2.976**	**4.044**
Brachystomella	Eupodes	**0.977**	**0.005**	**2.976**	**3.090**
Brachystomella	Mesostigmata	**0.977**	**-0.411**	**2.976**	**2.277**
Brachystomella	Scutacarus	**0.977**	**-0.608**	**2.976**	**3.481**
Brachystomella	Stigmaeidae	**0.977**	**0.229**	**2.976**	**2.578**
Friesea	Arctoseius	**0.434**	**-0.152**	**1.976**	**2.453**
Friesea	Dendrolaelaps	**0.434**	**0.027**	**1.976**	**2.821**
Friesea	Dendroseius	**0.434**	**-0.310**	**1.976**	**1.976**
Friesea	Lasioseius	**0.434**	**0.114**	**1.976**	**1.976**
Friesea	Lysigamasus	**0.434**	**0.407**	**1.976**	**1.976**
Friesea	Parasitus	**0.434**	**0.859**	**1.976**	**2.277**
Friesea	Pergamasus	**0.434**	**1.081**	**1.976**	**1.976**
Friesea	Rhodacarellus	**0.434**	**-0.310**	**1.976**	**2.930**
Friesea	Aporcelaimellus	**0.434**	**0.548**	**1.976**	**4.889**
Friesea	Dorylaimoidea	**0.434**	**-0.604**	**1.976**	**4.345**
Friesea	Qudsianematidae	**0.434**	**-0.207**	**1.976**	**4.044**
Friesea	Eupodes	**0.434**	**0.005**	**1.976**	**3.090**
Friesea	Mesostigmata	**0.434**	**-0.411**	**1.976**	**2.277**
Friesea	Scutacarus	**0.434**	**-0.608**	**1.976**	**3.481**

Friesea	Stigmaeidae	**0.434**	**0.229**	**1.976**	**2.578**
Isotoma	Arctoseius	**1.898**	**-0.152**	**3.700**	**2.453**
Isotoma	Dendrolaelaps	**1.898**	**0.027**	**3.700**	**2.821**
Isotoma	Dendroseius	**1.898**	**-0.310**	**3.700**	**1.976**
Isotoma	Lasioseius	**1.898**	**0.114**	**3.700**	**1.976**
Isotoma	Lysigamasus	**1.898**	**0.407**	**3.700**	**1.976**
Isotoma	Parasitus	**1.898**	**0.859**	**3.700**	**2.277**
Isotoma	Pergamasus	**1.898**	**1.081**	**3.700**	**1.976**
Isotoma	Rhodacarellus	**1.898**	**-0.310**	**3.700**	**2.930**
Isotoma	Aporcelaimellus	**1.898**	**0.548**	**3.700**	**4.889**
Isotoma	Dorylaimoidea	**1.898**	**-0.604**	**3.700**	**4.345**
Isotoma	Qudsianematidae	**1.898**	**-0.207**	**3.700**	**4.044**
Isotoma	Eupodes	**1.898**	**0.005**	**3.700**	**3.090**
Isotoma	Mesostigmata	**1.898**	**-0.411**	**3.700**	**2.277**
Isotoma	Scutacarus	**1.898**	**-0.608**	**3.700**	**3.481**
Isotoma	Stigmaeidae	**1.898**	**0.229**	**3.700**	**2.578**
Isotomurus	Arctoseius	**1.787**	**-0.152**	**2.453**	**2.453**
Isotomurus	Dendrolaelaps	**1.787**	**0.027**	**2.453**	**2.821**
Isotomurus	Dendroseius	**1.787**	**-0.310**	**2.453**	**1.976**
Isotomurus	Lasioseius	**1.787**	**0.114**	**2.453**	**1.976**
Isotomurus	Lysigamasus	**1.787**	**0.407**	**2.453**	**1.976**
Isotomurus	Parasitus	**1.787**	**0.859**	**2.453**	**2.277**
Isotomurus	Pergamasus	**1.787**	**1.081**	**2.453**	**1.976**
Isotomurus	Rhodacarellus	**1.787**	**-0.310**	**2.453**	**2.930**
Isotomurus	Aporcelaimellus	**1.787**	**0.548**	**2.453**	**4.889**
Isotomurus	Dorylaimoidea	**1.787**	**-0.604**	**2.453**	**4.345**
Isotomurus	Qudsianematidae	**1.787**	**-0.207**	**2.453**	**4.044**
Isotomurus	Eupodes	**1.787**	**0.005**	**2.453**	**3.090**
Isotomurus	Mesostigmata	**1.787**	**-0.411**	**2.453**	**2.277**
Isotomurus	Scutacarus	**1.787**	**-0.608**	**2.453**	**3.481**
Isotomurus	Stigmaeidae	**1.787**	**0.229**	**2.453**	**2.578**
Parisotoma	Arctoseius	**0.722**	**-0.152**	**3.507**	**2.453**
Parisotoma	Dendrolaelaps	**0.722**	**0.027**	**3.507**	**2.821**
Parisotoma	Dendroseius	**0.722**	**-0.310**	**3.507**	**1.976**
Parisotoma	Lasioseius	**0.722**	**0.114**	**3.507**	**1.976**
Parisotoma	Lysigamasus	**0.722**	**0.407**	**3.507**	**1.976**
Parisotoma	Parasitus	**0.722**	**0.859**	**3.507**	**2.277**
Parisotoma	Pergamasus	**0.722**	**1.081**	**3.507**	**1.976**
Parisotoma	Rhodacarellus	**0.722**	**-0.310**	**3.507**	**2.930**
Parisotoma	Aporcelaimellus	**0.722**	**0.548**	**3.507**	**4.889**
Parisotoma	Dorylaimoidea	**0.722**	**-0.604**	**3.507**	**4.345**
Parisotoma	Qudsianematidae	**0.722**	**-0.207**	**3.507**	**4.044**
Parisotoma	Eupodes	**0.722**	**0.005**	**3.507**	**3.090**
Parisotoma	Mesostigmata	**0.722**	**-0.411**	**3.507**	**2.277**
Parisotoma	Scutacarus	**0.722**	**-0.608**	**3.507**	**3.481**
Parisotoma	Stigmaeidae	**0.722**	**0.229**	**3.507**	**2.578**
Proisotoma	Arctoseius	**0.770**	**-0.152**	**3.231**	**2.453**
Proisotoma	Dendrolaelaps	**0.770**	**0.027**	**3.231**	**2.821**
Proisotoma	Dendroseius	**0.770**	**-0.310**	**3.231**	**1.976**
Proisotoma	Lasioseius	**0.770**	**0.114**	**3.231**	**1.976**
Proisotoma	Lysigamasus	**0.770**	**0.407**	**3.231**	**1.976**

Proisotoma	Parasitus	**0.770**	**0.859**	**3.231**	**2.277**
Proisotoma	Pergamasus	**0.770**	**1.081**	**3.231**	**1.976**
Proisotoma	Rhodacarellus	**0.770**	**-0.310**	**3.231**	**2.930**
Proisotoma	Aporcelaimellus	**0.770**	**0.548**	**3.231**	**4.889**
Proisotoma	Dorylaimoidea	**0.770**	**-0.604**	**3.231**	**4.345**
Proisotoma	Qudsianematidae	**0.770**	**-0.207**	**3.231**	**4.044**
Proisotoma	Eupodes	**0.770**	**0.005**	**3.231**	**3.090**
Proisotoma	Mesostigmata	**0.770**	**-0.411**	**3.231**	**2.277**
Proisotoma	Scutacarus	**0.770**	**-0.608**	**3.231**	**3.481**
Proisotoma	Stigmaeidae	**0.770**	**0.229**	**3.231**	**2.578**
Achaeta	Arctoseius	**0.584**	**-0.152**	**3.836**	**2.453**
Achaeta	Dendrolaelaps	**0.584**	**0.027**	**3.836**	**2.821**
Achaeta	Dendroseius	**0.584**	**-0.310**	**3.836**	**1.976**
Achaeta	Lasioseius	**0.584**	**0.114**	**3.836**	**1.976**
Achaeta	Lysigamasus	**0.584**	**0.407**	**3.836**	**1.976**
Achaeta	Parasitus	**0.584**	**0.859**	**3.836**	**2.277**
Achaeta	Pergamasus	**0.584**	**1.081**	**3.836**	**1.976**
Achaeta	Rhodacarellus	**0.584**	**-0.310**	**3.836**	**2.930**
Achaeta	Aporcelaimellus	**0.584**	**0.548**	**3.836**	**4.889**
Achaeta	Dorylaimoidea	**0.584**	**-0.604**	**3.836**	**4.345**
Achaeta	Qudsianematidae	**0.584**	**-0.207**	**3.836**	**4.044**
Achaeta	Eupodes	**0.584**	**0.005**	**3.836**	**3.090**
Achaeta	Mesostigmata	**0.584**	**-0.411**	**3.836**	**2.277**
Achaeta	Scutacarus	**0.584**	**-0.608**	**3.836**	**3.481**
Achaeta	Stigmaeidae	**0.584**	**0.229**	**3.836**	**2.578**
Fridericia	Arctoseius	**1.447**	**-0.152**	**4.232**	**2.453**
Fridericia	Dendrolaelaps	**1.447**	**0.027**	**4.232**	**2.821**
Fridericia	Dendroseius	**1.447**	**-0.310**	**4.232**	**1.976**
Fridericia	Lasioseius	**1.447**	**0.114**	**4.232**	**1.976**
Fridericia	Lysigamasus	**1.447**	**0.407**	**4.232**	**1.976**
Fridericia	Parasitus	**1.447**	**0.859**	**4.232**	**2.277**
Fridericia	Pergamasus	**1.447**	**1.081**	**4.232**	**1.976**
Fridericia	Rhodacarellus	**1.447**	**-0.310**	**4.232**	**2.930**
Fridericia	Aporcelaimellus	**1.447**	**0.548**	**4.232**	**4.889**
Fridericia	Dorylaimoidea	**1.447**	**-0.604**	**4.232**	**4.345**
Fridericia	Qudsianematidae	**1.447**	**-0.207**	**4.232**	**4.044**
Fridericia	Eupodes	**1.447**	**0.005**	**4.232**	**3.090**
Fridericia	Mesostigmata	**1.447**	**-0.411**	**4.232**	**2.277**
Fridericia	Scutacarus	**1.447**	**-0.608**	**4.232**	**3.481**
Fridericia	Stigmaeidae	**1.447**	**0.229**	**4.232**	**2.578**
Acrobeles	Tripyla	**-0.721**	**-0.420**	**4.044**	**4.044**
Acrobeles	Alliphis	**-0.721**	**0.053**	**4.044**	**3.407**
Acrobeles	Arctoseius	**-0.721**	**-0.152**	**4.044**	**2.453**
Acrobeles	Dendrolaelaps	**-0.721**	**0.027**	**4.044**	**2.821**
Acrobeles	Dendroseius	**-0.721**	**-0.310**	**4.044**	**1.976**
Acrobeles	Lasioseius	**-0.721**	**0.114**	**4.044**	**1.976**
Acrobeles	Lysigamasus	**-0.721**	**0.407**	**4.044**	**1.976**
Acrobeles	Parasitus	**-0.721**	**0.859**	**4.044**	**2.277**
Acrobeles	Pergamasus	**-0.721**	**1.081**	**4.044**	**1.976**
Acrobeles	Rhodacarellus	**-0.721**	**-0.310**	**4.044**	**2.930**
Acrobeles	Aporcelaimellus	**-0.721**	**0.548**	**4.044**	**4.889**

Acrobeles	Dorylaimoidea	**-0.721**	**-0.604**	**4.044**	**4.345**
Acrobeles	Qudsianematidae	**-0.721**	**-0.207**	**4.044**	**4.044**
Acrobeles	Eupodes	**-0.721**	**0.005**	**4.044**	**3.090**
Acrobeles	Mesostigmata	**-0.721**	**-0.411**	**4.044**	**2.277**
Acrobeles	Scutacarus	**-0.721**	**-0.608**	**4.044**	**3.481**
Acrobeles	Stigmaeidae	**-0.721**	**0.229**	**4.044**	**2.578**
Acrobeloides	Tripyla	**-1.171**	**-0.420**	**4.345**	**4.044**
Acrobeloides	Alliphis	**-1.171**	**0.053**	**4.345**	**3.407**
Acrobeloides	Arctoseius	**-1.171**	**-0.152**	**4.345**	**2.453**
Acrobeloides	Dendrolaelaps	**-1.171**	**0.027**	**4.345**	**2.821**
Acrobeloides	Dendroseius	**-1.171**	**-0.310**	**4.345**	**1.976**
Acrobeloides	Lasioseius	**-1.171**	**0.114**	**4.345**	**1.976**
Acrobeloides	Lysigamasus	**-1.171**	**0.407**	**4.345**	**1.976**
Acrobeloides	Parasitus	**-1.171**	**0.859**	**4.345**	**2.277**
Acrobeloides	Pergamasus	**-1.171**	**1.081**	**4.345**	**1.976**
Acrobeloides	Rhodacarellus	**-1.171**	**-0.310**	**4.345**	**2.930**
Acrobeloides	Aporcelaimellus	**-1.171**	**0.548**	**4.345**	**4.889**
Acrobeloides	Dorylaimoidea	**-1.171**	**-0.604**	**4.345**	**4.345**
Acrobeloides	Qudsianematidae	**-1.171**	**-0.207**	**4.345**	**4.044**
Acrobeloides	Eupodes	**-1.171**	**0.005**	**4.345**	**3.090**
Acrobeloides	Mesostigmata	**-1.171**	**-0.411**	**4.345**	**2.277**
Acrobeloides	Scutacarus	**-1.171**	**-0.608**	**4.345**	**3.481**
Acrobeloides	Stigmaeidae	**-1.171**	**0.229**	**4.345**	**2.578**
Alaimus	Tripyla	**-0.858**	**-0.420**	**4.345**	**4.044**
Alaimus	Alliphis	**-0.858**	**0.053**	**4.345**	**3.407**
Alaimus	Arctoseius	**-0.858**	**-0.152**	**4.345**	**2.453**
Alaimus	Dendrolaelaps	**-0.858**	**0.027**	**4.345**	**2.821**
Alaimus	Dendroseius	**-0.858**	**-0.310**	**4.345**	**1.976**
Alaimus	Lasioseius	**-0.858**	**0.114**	**4.345**	**1.976**
Alaimus	Lysigamasus	**-0.858**	**0.407**	**4.345**	**1.976**
Alaimus	Parasitus	**-0.858**	**0.859**	**4.345**	**2.277**
Alaimus	Pergamasus	**-0.858**	**1.081**	**4.345**	**1.976**
Alaimus	Rhodacarellus	**-0.858**	**-0.310**	**4.345**	**2.930**
Alaimus	Aporcelaimellus	**-0.858**	**0.548**	**4.345**	**4.889**
Alaimus	Dorylaimoidea	**-0.858**	**-0.604**	**4.345**	**4.345**
Alaimus	Qudsianematidae	**-0.858**	**-0.207**	**4.345**	**4.044**
Alaimus	Eupodes	**-0.858**	**0.005**	**4.345**	**3.090**
Alaimus	Mesostigmata	**-0.858**	**-0.411**	**4.345**	**2.277**
Alaimus	Scutacarus	**-0.858**	**-0.608**	**4.345**	**3.481**
Alaimus	Stigmaeidae	**-0.858**	**0.229**	**4.345**	**2.578**
Anaplectus	Tripyla	**-0.519**	**-0.420**	**4.044**	**4.044**
Anaplectus	Alliphis	**-0.519**	**0.053**	**4.044**	**3.407**
Anaplectus	Arctoseius	**-0.519**	**-0.152**	**4.044**	**2.453**
Anaplectus	Dendrolaelaps	**-0.519**	**0.027**	**4.044**	**2.821**
Anaplectus	Dendroseius	**-0.519**	**-0.310**	**4.044**	**1.976**
Anaplectus	Lasioseius	**-0.519**	**0.114**	**4.044**	**1.976**
Anaplectus	Lysigamasus	**-0.519**	**0.407**	**4.044**	**1.976**
Anaplectus	Parasitus	**-0.519**	**0.859**	**4.044**	**2.277**
Anaplectus	Pergamasus	**-0.519**	**1.081**	**4.044**	**1.976**
Anaplectus	Rhodacarellus	**-0.519**	**-0.310**	**4.044**	**2.930**
Anaplectus	Aporcelaimellus	**-0.519**	**0.548**	**4.044**	**4.889**

Anaplectus	Dorylaimoidea	**-0.519**	**-0.604**	**4.044**	**4.345**
Anaplectus	Qudsianematidae	**-0.519**	**-0.207**	**4.044**	**4.044**
Anaplectus	Eupodes	**-0.519**	**0.005**	**4.044**	**3.090**
Anaplectus	Mesostigmata	**-0.519**	**-0.411**	**4.044**	**2.277**
Anaplectus	Scutacarus	**-0.519**	**-0.608**	**4.044**	**3.481**
Anaplectus	Stigmaeidae	**-0.519**	**0.229**	**4.044**	**2.578**
Cephalobidae	Tripyla	**-1.055**	**-0.420**	**4.044**	**4.044**
Cephalobidae	Alliphis	**-1.055**	**0.053**	**4.044**	**3.407**
Cephalobidae	Arctoseius	**-1.055**	**-0.152**	**4.044**	**2.453**
Cephalobidae	Dendrolaelaps	**-1.055**	**0.027**	**4.044**	**2.821**
Cephalobidae	Dendroseius	**-1.055**	**-0.310**	**4.044**	**1.976**
Cephalobidae	Lasioseius	**-1.055**	**0.114**	**4.044**	**1.976**
Cephalobidae	Lysigamasus	**-1.055**	**0.407**	**4.044**	**1.976**
Cephalobidae	Parasitus	**-1.055**	**0.859**	**4.044**	**2.277**
Cephalobidae	Pergamasus	**-1.055**	**1.081**	**4.044**	**1.976**
Cephalobidae	Rhodacarellus	**-1.055**	**-0.310**	**4.044**	**2.930**
Cephalobidae	Aporcelaimellus	**-1.055**	**0.548**	**4.044**	**4.889**
Cephalobidae	Dorylaimoidea	**-1.055**	**-0.604**	**4.044**	**4.345**
Cephalobidae	Qudsianematidae	**-1.055**	**-0.207**	**4.044**	**4.044**
Cephalobidae	Eupodes	**-1.055**	**0.005**	**4.044**	**3.090**
Cephalobidae	Mesostigmata	**-1.055**	**-0.411**	**4.044**	**2.277**
Cephalobidae	Scutacarus	**-1.055**	**-0.608**	**4.044**	**3.481**
Cephalobidae	Stigmaeidae	**-1.055**	**0.229**	**4.044**	**2.578**
Eucephalobus	Tripyla	**-0.855**	**-0.420**	**4.947**	**4.044**
Eucephalobus	Alliphis	**-0.855**	**0.053**	**4.947**	**3.407**
Eucephalobus	Arctoseius	**-0.855**	**-0.152**	**4.947**	**2.453**
Eucephalobus	Dendrolaelaps	**-0.855**	**0.027**	**4.947**	**2.821**
Eucephalobus	Dendroseius	**-0.855**	**-0.310**	**4.947**	**1.976**
Eucephalobus	Lasioseius	**-0.855**	**0.114**	**4.947**	**1.976**
Eucephalobus	Lysigamasus	**-0.855**	**0.407**	**4.947**	**1.976**
Eucephalobus	Parasitus	**-0.855**	**0.859**	**4.947**	**2.277**
Eucephalobus	Pergamasus	**-0.855**	**1.081**	**4.947**	**1.976**
Eucephalobus	Rhodacarellus	**-0.855**	**-0.310**	**4.947**	**2.930**
Eucephalobus	Aporcelaimellus	**-0.855**	**0.548**	**4.947**	**4.889**
Eucephalobus	Dorylaimoidea	**-0.855**	**-0.604**	**4.947**	**4.345**
Eucephalobus	Qudsianematidae	**-0.855**	**-0.207**	**4.947**	**4.044**
Eucephalobus	Eupodes	**-0.855**	**0.005**	**4.947**	**3.090**
Eucephalobus	Mesostigmata	**-0.855**	**-0.411**	**4.947**	**2.277**
Eucephalobus	Scutacarus	**-0.855**	**-0.608**	**4.947**	**3.481**
Eucephalobus	Stigmaeidae	**-0.855**	**0.229**	**4.947**	**2.578**
Panagrolaimus	Tripyla	**-0.945**	**-0.420**	**5.248**	**4.044**
Panagrolaimus	Alliphis	**-0.945**	**0.053**	**5.248**	**3.407**
Panagrolaimus	Arctoseius	**-0.945**	**-0.152**	**5.248**	**2.453**
Panagrolaimus	Dendrolaelaps	**-0.945**	**0.027**	**5.248**	**2.821**
Panagrolaimus	Dendroseius	**-0.945**	**-0.310**	**5.248**	**1.976**
Panagrolaimus	Lasioseius	**-0.945**	**0.114**	**5.248**	**1.976**
Panagrolaimus	Lysigamasus	**-0.945**	**0.407**	**5.248**	**1.976**
Panagrolaimus	Parasitus	**-0.945**	**0.859**	**5.248**	**2.277**
Panagrolaimus	Pergamasus	**-0.945**	**1.081**	**5.248**	**1.976**
Panagrolaimus	Rhodacarellus	**-0.945**	**-0.310**	**5.248**	**2.930**
Panagrolaimus	Aporcelaimellus	**-0.945**	**0.548**	**5.248**	**4.889**

Panagrolaimus	Dorylaimoidea	**-0.945**	**-0.604**	**5.248**	**4.345**
Panagrolaimus	Qudsianematidae	**-0.945**	**-0.207**	**5.248**	**4.044**
Panagrolaimus	Eupodes	**-0.945**	**0.005**	**5.248**	**3.090**
Panagrolaimus	Mesostigmata	**-0.945**	**-0.411**	**5.248**	**2.277**
Panagrolaimus	Scutacarus	**-0.945**	**-0.608**	**5.248**	**3.481**
Panagrolaimus	Stigmaeidae	**-0.945**	**0.229**	**5.248**	**2.578**
Plectus	Tripyla	**-0.583**	**-0.420**	**4.947**	**4.044**
Plectus	Alliphis	**-0.583**	**0.053**	**4.947**	**3.407**
Plectus	Arctoseius	**-0.583**	**-0.152**	**4.947**	**2.453**
Plectus	Dendrolaelaps	**-0.583**	**0.027**	**4.947**	**2.821**
Plectus	Dendroseius	**-0.583**	**-0.310**	**4.947**	**1.976**
Plectus	Lasioseius	**-0.583**	**0.114**	**4.947**	**1.976**
Plectus	Lysigamasus	**-0.583**	**0.407**	**4.947**	**1.976**
Plectus	Parasitus	**-0.583**	**0.859**	**4.947**	**2.277**
Plectus	Pergamasus	**-0.583**	**1.081**	**4.947**	**1.976**
Plectus	Rhodacarellus	**-0.583**	**-0.310**	**4.947**	**2.930**
Plectus	Aporcelaimellus	**-0.583**	**0.548**	**4.947**	**4.889**
Plectus	Dorylaimoidea	**-0.583**	**-0.604**	**4.947**	**4.345**
Plectus	Qudsianematidae	**-0.583**	**-0.207**	**4.947**	**4.044**
Plectus	Eupodes	**-0.583**	**0.005**	**4.947**	**3.090**
Plectus	Mesostigmata	**-0.583**	**-0.411**	**4.947**	**2.277**
Plectus	Scutacarus	**-0.583**	**-0.608**	**4.947**	**3.481**
Plectus	Stigmaeidae	**-0.583**	**0.229**	**4.947**	**2.578**
Prismatolaimus	Tripyla	**-1.280**	**-0.420**	**4.345**	**4.044**
Prismatolaimus	Alliphis	**-1.280**	**0.053**	**4.345**	**3.407**
Prismatolaimus	Arctoseius	**-1.280**	**-0.152**	**4.345**	**2.453**
Prismatolaimus	Dendrolaelaps	**-1.280**	**0.027**	**4.345**	**2.821**
Prismatolaimus	Dendroseius	**-1.280**	**-0.310**	**4.345**	**1.976**
Prismatolaimus	Lasioseius	**-1.280**	**0.114**	**4.345**	**1.976**
Prismatolaimus	Lysigamasus	**-1.280**	**0.407**	**4.345**	**1.976**
Prismatolaimus	Parasitus	**-1.280**	**0.859**	**4.345**	**2.277**
Prismatolaimus	Pergamasus	**-1.280**	**1.081**	**4.345**	**1.976**
Prismatolaimus	Rhodacarellus	**-1.280**	**-0.310**	**4.345**	**2.930**
Prismatolaimus	Aporcelaimellus	**-1.280**	**0.548**	**4.345**	**4.889**
Prismatolaimus	Dorylaimoidea	**-1.280**	**-0.604**	**4.345**	**4.345**
Prismatolaimus	Qudsianematidae	**-1.280**	**-0.207**	**4.345**	**4.044**
Prismatolaimus	Eupodes	**-1.280**	**0.005**	**4.345**	**3.090**
Prismatolaimus	Mesostigmata	**-1.280**	**-0.411**	**4.345**	**2.277**
Prismatolaimus	Scutacarus	**-1.280**	**-0.608**	**4.345**	**3.481**
Prismatolaimus	Stigmaeidae	**-1.280**	**0.229**	**4.345**	**2.578**
Rhabditidae	Tripyla	**-0.692**	**-0.420**	**5.646**	**4.044**
Rhabditidae	Alliphis	**-0.692**	**0.053**	**5.646**	**3.407**
Rhabditidae	Arctoseius	**-0.692**	**-0.152**	**5.646**	**2.453**
Rhabditidae	Dendrolaelaps	**-0.692**	**0.027**	**5.646**	**2.821**
Rhabditidae	Dendroseius	**-0.692**	**-0.310**	**5.646**	**1.976**
Rhabditidae	Lasioseius	**-0.692**	**0.114**	**5.646**	**1.976**
Rhabditidae	Lysigamasus	**-0.692**	**0.407**	**5.646**	**1.976**
Rhabditidae	Parasitus	**-0.692**	**0.859**	**5.646**	**2.277**
Rhabditidae	Pergamasus	**-0.692**	**1.081**	**5.646**	**1.976**
Rhabditidae	Rhodacarellus	**-0.692**	**-0.310**	**5.646**	**2.930**
Rhabditidae	Aporcelaimellus	**-0.692**	**0.548**	**5.646**	**4.889**

Rhabditidae	Dorylaimoidea	**-0.692**	**-0.604**	**5.646**	**4.345**
Rhabditidae	Qudsianematidae	**-0.692**	**-0.207**	**5.646**	**4.044**
Rhabditidae	Eupodes	**-0.692**	**0.005**	**5.646**	**3.090**
Rhabditidae	Mesostigmata	**-0.692**	**-0.411**	**5.646**	**2.277**
Rhabditidae	Scutacarus	**-0.692**	**-0.608**	**5.646**	**3.481**
Rhabditidae	Stigmaeidae	**-0.692**	**0.229**	**5.646**	**2.578**
Eupelops	Bdella	**0.326**	**0.816**	**2.453**	**1.976**
Eupelops	Arctoseius	**0.326**	**-0.152**	**2.453**	**2.453**
Eupelops	Dendrolaelaps	**0.326**	**0.027**	**2.453**	**2.821**
Eupelops	Dendroseius	**0.326**	**-0.310**	**2.453**	**1.976**
Eupelops	Lasioseius	**0.326**	**0.114**	**2.453**	**1.976**
Eupelops	Lysigamasus	**0.326**	**0.407**	**2.453**	**1.976**
Eupelops	Parasitus	**0.326**	**0.859**	**2.453**	**2.277**
Eupelops	Pergamasus	**0.326**	**1.081**	**2.453**	**1.976**
Eupelops	Rhodacarellus	**0.326**	**-0.310**	**2.453**	**2.930**
Eupelops	Eupodes	**0.326**	**0.005**	**2.453**	**3.090**
Eupelops	Mesostigmata	**0.326**	**-0.411**	**2.453**	**2.277**
Eupelops	Scutacarus	**0.326**	**-0.608**	**2.453**	**3.481**
Eupelops	Stigmaeidae	**0.326**	**0.229**	**2.453**	**2.578**
Enchytraeus	Arctoseius	**1.114**	**-0.152**	**4.568**	**2.453**
Enchytraeus	Dendrolaelaps	**1.114**	**0.027**	**4.568**	**2.821**
Enchytraeus	Dendroseius	**1.114**	**-0.310**	**4.568**	**1.976**
Enchytraeus	Lasioseius	**1.114**	**0.114**	**4.568**	**1.976**
Enchytraeus	Lysigamasus	**1.114**	**0.407**	**4.568**	**1.976**
Enchytraeus	Parasitus	**1.114**	**0.859**	**4.568**	**2.277**
Enchytraeus	Pergamasus	**1.114**	**1.081**	**4.568**	**1.976**
Enchytraeus	Rhodacarellus	**1.114**	**-0.310**	**4.568**	**2.930**
Enchytraeus	Aporcelaimellus	**1.114**	**0.548**	**4.568**	**4.889**
Enchytraeus	Dorylaimoidea	**1.114**	**-0.604**	**4.568**	**4.345**
Enchytraeus	Qudsianematidae	**1.114**	**-0.207**	**4.568**	**4.044**
Enchytraeus	Eupodes	**1.114**	**0.005**	**4.568**	**3.090**
Enchytraeus	Mesostigmata	**1.114**	**-0.411**	**4.568**	**2.277**
Enchytraeus	Scutacarus	**1.114**	**-0.608**	**4.568**	**3.481**
Enchytraeus	Stigmaeidae	**1.114**	**0.229**	**4.568**	**2.578**
Eubacteria	Acrobeles	**-6.649**	**-0.721**	**12.819**	**4.044**
Eubacteria	Acrobeloides	**-6.649**	**-1.171**	**12.819**	**4.345**
Eubacteria	Alaimus	**-6.649**	**-0.858**	**12.819**	**4.345**
Eubacteria	Anaplectus	**-6.649**	**-0.519**	**12.819**	**4.044**
Eubacteria	Cephalobidae	**-6.649**	**-1.055**	**12.819**	**4.044**
Eubacteria	Eucephalobus	**-6.649**	**-0.855**	**12.819**	**4.947**
Eubacteria	Panagrolaimus	**-6.649**	**-0.945**	**12.819**	**5.248**
Eubacteria	Plectus	**-6.649**	**-0.583**	**12.819**	**4.947**
Eubacteria	Prismatolaimus	**-6.649**	**-1.280**	**12.819**	**4.345**
Eubacteria	Rhabditidae	**-6.649**	**-0.692**	**12.819**	**5.646**
Eubacteria	Eupelops	**-6.649**	**0.326**	**12.819**	**2.453**
Eubacteria	Enchytraeus	**-6.649**	**1.114**	**12.819**	**4.568**
Eubacteria	Dauerlarvae	**-6.649**	**-0.804**	**12.819**	**4.521**
Eubacteria	Juveniles	**-6.649**	**1.363**	**12.819**	**3.714**
Eubacteria	Henlea	**-6.649**	**1.899**	**12.819**	**4.511**
Eubacteria	Marionina	**-6.649**	**0.879**	**12.819**	**4.559**
Dauerlarvae	Tripyla	**-0.804**	**-0.420**	**4.521**	**4.044**

Dauerlarvae	Alliphis	**-0.804**	**0.053**	**4.521**	**3.407**
Dauerlarvae	Aporcelaimellus	**-0.804**	**0.548**	**4.521**	**4.889**
Dauerlarvae	Dorylaimoidea	**-0.804**	**-0.604**	**4.521**	**4.345**
Dauerlarvae	Qudsianematidae	**-0.804**	**-0.207**	**4.521**	**4.044**
Dauerlarvae	Eupodes	**-0.804**	**0.005**	**4.521**	**3.090**
Dauerlarvae	Mesostigmata	**-0.804**	**-0.411**	**4.521**	**2.277**
Dauerlarvae	Scutacarus	**-0.804**	**-0.608**	**4.521**	**3.481**
Dauerlarvae	Stigmaeidae	**-0.804**	**0.229**	**4.521**	**2.578**
Juveniles	Arctoseius	**1.363**	**-0.152**	**3.714**	**2.453**
Juveniles	Dendrolaelaps	**1.363**	**0.027**	**3.714**	**2.821**
Juveniles	Dendroseius	**1.363**	**-0.310**	**3.714**	**1.976**
Juveniles	Lasioseius	**1.363**	**0.114**	**3.714**	**1.976**
Juveniles	Lysigamasus	**1.363**	**0.407**	**3.714**	**1.976**
Juveniles	Parasitus	**1.363**	**0.859**	**3.714**	**2.277**
Juveniles	Pergamasus	**1.363**	**1.081**	**3.714**	**1.976**
Juveniles	Rhodacarellus	**1.363**	**-0.310**	**3.714**	**2.930**
Juveniles	Aporcelaimellus	**1.363**	**0.548**	**3.714**	**4.889**
Juveniles	Dorylaimoidea	**1.363**	**-0.604**	**3.714**	**4.345**
Juveniles	Qudsianematidae	**1.363**	**-0.207**	**3.714**	**4.044**
Juveniles	Eupodes	**1.363**	**0.005**	**3.714**	**3.090**
Juveniles	Mesostigmata	**1.363**	**-0.411**	**3.714**	**2.277**
Juveniles	Scutacarus	**1.363**	**-0.608**	**3.714**	**3.481**
Juveniles	Stigmaeidae	**1.363**	**0.229**	**3.714**	**2.578**
Henlea	Arctoseius	**1.899**	**-0.152**	**4.511**	**2.453**
Henlea	Dendrolaelaps	**1.899**	**0.027**	**4.511**	**2.821**
Henlea	Dendroseius	**1.899**	**-0.310**	**4.511**	**1.976**
Henlea	Lasioseius	**1.899**	**0.114**	**4.511**	**1.976**
Henlea	Lysigamasus	**1.899**	**0.407**	**4.511**	**1.976**
Henlea	Parasitus	**1.899**	**0.859**	**4.511**	**2.277**
Henlea	Pergamasus	**1.899**	**1.081**	**4.511**	**1.976**
Henlea	Rhodacarellus	**1.899**	**-0.310**	**4.511**	**2.930**
Henlea	Aporcelaimellus	**1.899**	**0.548**	**4.511**	**4.889**
Henlea	Dorylaimoidea	**1.899**	**-0.604**	**4.511**	**4.345**
Henlea	Qudsianematidae	**1.899**	**-0.207**	**4.511**	**4.044**
Henlea	Eupodes	**1.899**	**0.005**	**4.511**	**3.090**
Henlea	Mesostigmata	**1.899**	**-0.411**	**4.511**	**2.277**
Henlea	Scutacarus	**1.899**	**-0.608**	**4.511**	**3.481**
Henlea	Stigmaeidae	**1.899**	**0.229**	**4.511**	**2.578**
Marionina	Arctoseius	**0.879**	**-0.152**	**4.559**	**2.453**
Marionina	Dendrolaelaps	**0.879**	**0.027**	**4.559**	**2.821**
Marionina	Dendroseius	**0.879**	**-0.310**	**4.559**	**1.976**
Marionina	Lasioseius	**0.879**	**0.114**	**4.559**	**1.976**
Marionina	Lysigamasus	**0.879**	**0.407**	**4.559**	**1.976**
Marionina	Parasitus	**0.879**	**0.859**	**4.559**	**2.277**
Marionina	Pergamasus	**0.879**	**1.081**	**4.559**	**1.976**
Marionina	Rhodacarellus	**0.879**	**-0.310**	**4.559**	**2.930**
Marionina	Aporcelaimellus	**0.879**	**0.548**	**4.559**	**4.889**
Marionina	Dorylaimoidea	**0.879**	**-0.604**	**4.559**	**4.345**
Marionina	Qudsianematidae	**0.879**	**-0.207**	**4.559**	**4.044**
Marionina	Eupodes	**0.879**	**0.005**	**4.559**	**3.090**
Marionina	Mesostigmata	**0.879**	**-0.411**	**4.559**	**2.277**

Marionina	Scutacarus	**0.879**	**-0.608**	**4.559**	**3.481**
Marionina	Stigmaeidae	**0.879**	**0.229**	**4.559**	**2.578**
Hyphae and hair roots	Dolichodoridae	**6.672**	**-0.885**	**0.000**	**5.366**
Hyphae and hair roots	Helicotylenchus	**6.672**	**-0.792**	**0.000**	**4.822**
Hyphae and hair roots	Hoplolaimidae	**6.672**	**-1.090**	**0.000**	**4.044**
Hyphae and hair roots	Malenchus	**6.672**	**-1.330**	**0.000**	**4.646**
Hyphae and hair roots	Paratylenchus	**6.672**	**-1.244**	**0.000**	**4.646**
Hyphae and hair roots	Pratylenchus	**6.672**	**-1.226**	**0.000**	**4.044**
Hyphae and hair roots	Trichodorus	**6.672**	**-0.744**	**0.000**	**4.521**
Hyphae and hair roots	Tylenchorhynchus	**6.672**	**-0.664**	**0.000**	**4.646**
Hyphae and hair roots	Pachygnatidae	**6.672**	**-0.113**	**0.000**	**2.821**
Hyphae and hair roots	Rhizoglyphus	**6.672**	**0.005**	**0.000**	**1.976**
Hyphae and hair roots	Tydeidae	**6.672**	**-0.608**	**0.000**	**2.976**
Hyphae and hair roots	Sminthuridae	**6.672**	**-0.608**	**0.000**	**2.277**
Hyphae and hair roots	Sminthurinus	**6.672**	**0.618**	**0.000**	**3.277**
Hyphae and hair roots	Sphaeridia	**6.672**	**0.202**	**0.000**	**3.017**
Hyphae and hair roots	Aphelenchoides	**6.672**	**-1.496**	**0.000**	**4.822**
Hyphae and hair roots	Tylenchidae	**6.672**	**-1.360**	**0.000**	**4.743**
Hyphae and hair roots	Medioppia	**6.672**	**-0.235**	**0.000**	**2.277**
Hyphae and hair roots	Pygmephorus	**6.672**	**-0.376**	**0.000**	**3.318**
Hyphae and hair roots	Siteroptes	**6.672**	**-0.376**	**0.000**	**1.976**
Hyphae and hair roots	Tyrophagus	**6.672**	**0.005**	**0.000**	**2.754**
Hyphae and hair roots	Brachystomella	**6.672**	**0.977**	**0.000**	**2.976**
Hyphae and hair roots	Friesea	**6.672**	**0.434**	**0.000**	**1.976**
Hyphae and hair roots	Isotoma	**6.672**	**1.898**	**0.000**	**3.700**
Hyphae and hair roots	Isotomurus	**6.672**	**1.787**	**0.000**	**2.453**
Hyphae and hair roots	Parisotoma	**6.672**	**0.722**	**0.000**	**3.507**
Hyphae and hair roots	Proisotoma	**6.672**	**0.770**	**0.000**	**3.231**
Hyphae and hair roots	Achaeta	**6.672**	**0.584**	**0.000**	**3.836**
Hyphae and hair roots	Fridericia	**6.672**	**1.447**	**0.000**	**4.232**
Hyphae and hair roots	Aporcelaimellus	**6.672**	**0.548**	**0.000**	**4.889**
Hyphae and hair roots	Dorylaimoidea	**6.672**	**-0.604**	**0.000**	**4.345**
Hyphae and hair roots	Qudsianematidae	**6.672**	**-0.207**	**0.000**	**4.044**
Hyphae and hair roots	Eupodes	**6.672**	**0.005**	**0.000**	**3.090**
Hyphae and hair roots	Mesostigmata	**6.672**	**-0.411**	**0.000**	**2.277**
Hyphae and hair roots	Scutacarus	**6.672**	**-0.608**	**0.000**	**3.481**
Hyphae and hair roots	Stigmaeidae	**6.672**	**0.229**	**0.000**	**2.578**
Tripyla	Arctoseius	**-0.420**	**-0.152**	**4.044**	**2.453**
Tripyla	Dendrolaelaps	**-0.420**	**0.027**	**4.044**	**2.821**
Tripyla	Dendroseius	**-0.420**	**-0.310**	**4.044**	**1.976**
Tripyla	Lasioseius	**-0.420**	**0.114**	**4.044**	**1.976**
Tripyla	Lysigamasus	**-0.420**	**0.407**	**4.044**	**1.976**
Tripyla	Parasitus	**-0.420**	**0.859**	**4.044**	**2.277**
Tripyla	Pergamasus	**-0.420**	**1.081**	**4.044**	**1.976**
Tripyla	Rhodacarellus	**-0.420**	**-0.310**	**4.044**	**2.930**
Tripyla	Aporcelaimellus	**-0.420**	**0.548**	**4.044**	**4.889**
Tripyla	Dorylaimoidea	**-0.420**	**-0.604**	**4.044**	**4.345**
Tripyla	Qudsianematidae	**-0.420**	**-0.207**	**4.044**	**4.044**
Tripyla	Eupodes	**-0.420**	**0.005**	**4.044**	**3.090**
Tripyla	Mesostigmata	**-0.420**	**-0.411**	**4.044**	**2.277**
Tripyla	Scutacarus	**-0.420**	**-0.608**	**4.044**	**3.481**

Tripyla	Stigmaeidae	**-0.420**	**0.229**	**4.044**	**2.578**
Alliphis	Bdella	**0.053**	**0.816**	**3.407**	**1.976**
Alliphis	Arctoseius	**0.053**	**-0.152**	**3.407**	**2.453**
Alliphis	Dendrolaelaps	**0.053**	**0.027**	**3.407**	**2.821**
Alliphis	Dendroseius	**0.053**	**-0.310**	**3.407**	**1.976**
Alliphis	Lasioseius	**0.053**	**0.114**	**3.407**	**1.976**
Alliphis	Lysigamasus	**0.053**	**0.407**	**3.407**	**1.976**
Alliphis	Parasitus	**0.053**	**0.859**	**3.407**	**2.277**
Alliphis	Pergamasus	**0.053**	**1.081**	**3.407**	**1.976**
Alliphis	Rhodacarellus	**0.053**	**-0.310**	**3.407**	**2.930**
Alliphis	Aporcelaimellus	**0.053**	**0.548**	**3.407**	**4.889**
Alliphis	Dorylaimoidea	**0.053**	**-0.604**	**3.407**	**4.345**
Alliphis	Qudsianematidae	**0.053**	**-0.207**	**3.407**	**4.044**
Alliphis	Eupodes	**0.053**	**0.005**	**3.407**	**3.090**
Alliphis	Mesostigmata	**0.053**	**-0.411**	**3.407**	**2.277**
Alliphis	Scutacarus	**0.053**	**-0.608**	**3.407**	**3.481**
Alliphis	Stigmaeidae	**0.053**	**0.229**	**3.407**	**2.578**
Bdella	Bdella	**0.816**	**0.816**	**1.976**	**1.976**
Bdella	Arctoseius	**0.816**	**-0.152**	**1.976**	**2.453**
Bdella	Dendrolaelaps	**0.816**	**0.027**	**1.976**	**2.821**
Bdella	Dendroseius	**0.816**	**-0.310**	**1.976**	**1.976**
Bdella	Lasioseius	**0.816**	**0.114**	**1.976**	**1.976**
Bdella	Lysigamasus	**0.816**	**0.407**	**1.976**	**1.976**
Bdella	Parasitus	**0.816**	**0.859**	**1.976**	**2.277**
Bdella	Pergamasus	**0.816**	**1.081**	**1.976**	**1.976**
Bdella	Rhodacarellus	**0.816**	**-0.310**	**1.976**	**2.930**
Bdella	Eupodes	**0.816**	**0.005**	**1.976**	**3.090**
Bdella	Mesostigmata	**0.816**	**-0.411**	**1.976**	**2.277**
Bdella	Scutacarus	**0.816**	**-0.608**	**1.976**	**3.481**
Bdella	Stigmaeidae	**0.816**	**0.229**	**1.976**	**2.578**
Arctoseius	Bdella	**-0.152**	**0.816**	**2.453**	**1.976**
Arctoseius	Aporcelaimellus	**-0.152**	**0.548**	**2.453**	**4.889**
Arctoseius	Dorylaimoidea	**-0.152**	**-0.604**	**2.453**	**4.345**
Arctoseius	Qudsianematidae	**-0.152**	**-0.207**	**2.453**	**4.044**
Arctoseius	Eupodes	**-0.152**	**0.005**	**2.453**	**3.090**
Arctoseius	Mesostigmata	**-0.152**	**-0.411**	**2.453**	**2.277**
Arctoseius	Scutacarus	**-0.152**	**-0.608**	**2.453**	**3.481**
Arctoseius	Stigmaeidae	**-0.152**	**0.229**	**2.453**	**2.578**
Dendrolaelaps	Bdella	**0.027**	**0.816**	**2.821**	**1.976**
Dendrolaelaps	Aporcelaimellus	**0.027**	**0.548**	**2.821**	**4.889**
Dendrolaelaps	Dorylaimoidea	**0.027**	**-0.604**	**2.821**	**4.345**
Dendrolaelaps	Qudsianematidae	**0.027**	**-0.207**	**2.821**	**4.044**
Dendrolaelaps	Eupodes	**0.027**	**0.005**	**2.821**	**3.090**
Dendrolaelaps	Mesostigmata	**0.027**	**-0.411**	**2.821**	**2.277**
Dendrolaelaps	Scutacarus	**0.027**	**-0.608**	**2.821**	**3.481**
Dendrolaelaps	Stigmaeidae	**0.027**	**0.229**	**2.821**	**2.578**
Dendroseius	Bdella	**-0.310**	**0.816**	**1.976**	**1.976**
Dendroseius	Aporcelaimellus	**-0.310**	**0.548**	**1.976**	**4.889**
Dendroseius	Dorylaimoidea	**-0.310**	**-0.604**	**1.976**	**4.345**
Dendroseius	Qudsianematidae	**-0.310**	**-0.207**	**1.976**	**4.044**
Dendroseius	Eupodes	**-0.310**	**0.005**	**1.976**	**3.090**

Dendroseius	Mesostigmata	**-0.310**	**-0.411**	**1.976**	**2.277**
Dendroseius	Scutacarus	**-0.310**	**-0.608**	**1.976**	**3.481**
Dendroseius	Stigmaeidae	**-0.310**	**0.229**	**1.976**	**2.578**
Lasioseius	Bdella	**0.114**	**0.816**	**1.976**	**1.976**
Lasioseius	Aporcelaimellus	**0.114**	**0.548**	**1.976**	**4.889**
Lasioseius	Dorylaimoidea	**0.114**	**-0.604**	**1.976**	**4.345**
Lasioseius	Qudsianematidae	**0.114**	**-0.207**	**1.976**	**4.044**
Lasioseius	Eupodes	**0.114**	**0.005**	**1.976**	**3.090**
Lasioseius	Mesostigmata	**0.114**	**-0.411**	**1.976**	**2.277**
Lasioseius	Scutacarus	**0.114**	**-0.608**	**1.976**	**3.481**
Lasioseius	Stigmaeidae	**0.114**	**0.229**	**1.976**	**2.578**
Lysigamasus	Bdella	**0.407**	**0.816**	**1.976**	**1.976**
Lysigamasus	Aporcelaimellus	**0.407**	**0.548**	**1.976**	**4.889**
Lysigamasus	Dorylaimoidea	**0.407**	**-0.604**	**1.976**	**4.345**
Lysigamasus	Qudsianematidae	**0.407**	**-0.207**	**1.976**	**4.044**
Lysigamasus	Eupodes	**0.407**	**0.005**	**1.976**	**3.090**
Lysigamasus	Mesostigmata	**0.407**	**-0.411**	**1.976**	**2.277**
Lysigamasus	Scutacarus	**0.407**	**-0.608**	**1.976**	**3.481**
Lysigamasus	Stigmaeidae	**0.407**	**0.229**	**1.976**	**2.578**
Parasitus	Bdella	**0.859**	**0.816**	**2.277**	**1.976**
Parasitus	Aporcelaimellus	**0.859**	**0.548**	**2.277**	**4.889**
Parasitus	Dorylaimoidea	**0.859**	**-0.604**	**2.277**	**4.345**
Parasitus	Qudsianematidae	**0.859**	**-0.207**	**2.277**	**4.044**
Parasitus	Eupodes	**0.859**	**0.005**	**2.277**	**3.090**
Parasitus	Mesostigmata	**0.859**	**-0.411**	**2.277**	**2.277**
Parasitus	Scutacarus	**0.859**	**-0.608**	**2.277**	**3.481**
Parasitus	Stigmaeidae	**0.859**	**0.229**	**2.277**	**2.578**
Pergamasus	Bdella	**1.081**	**0.816**	**1.976**	**1.976**
Pergamasus	Aporcelaimellus	**1.081**	**0.548**	**1.976**	**4.889**
Pergamasus	Dorylaimoidea	**1.081**	**-0.604**	**1.976**	**4.345**
Pergamasus	Qudsianematidae	**1.081**	**-0.207**	**1.976**	**4.044**
Pergamasus	Eupodes	**1.081**	**0.005**	**1.976**	**3.090**
Pergamasus	Mesostigmata	**1.081**	**-0.411**	**1.976**	**2.277**
Pergamasus	Scutacarus	**1.081**	**-0.608**	**1.976**	**3.481**
Pergamasus	Stigmaeidae	**1.081**	**0.229**	**1.976**	**2.578**
Rhodacarellus	Bdella	**-0.310**	**0.816**	**2.930**	**1.976**
Rhodacarellus	Aporcelaimellus	**-0.310**	**0.548**	**2.930**	**4.889**
Rhodacarellus	Dorylaimoidea	**-0.310**	**-0.604**	**2.930**	**4.345**
Rhodacarellus	Qudsianematidae	**-0.310**	**-0.207**	**2.930**	**4.044**
Rhodacarellus	Eupodes	**-0.310**	**0.005**	**2.930**	**3.090**
Rhodacarellus	Mesostigmata	**-0.310**	**-0.411**	**2.930**	**2.277**
Rhodacarellus	Scutacarus	**-0.310**	**-0.608**	**2.930**	**3.481**
Rhodacarellus	Stigmaeidae	**-0.310**	**0.229**	**2.930**	**2.578**
Aporcelaimellus	Tripyla	**0.548**	**-0.420**	**4.889**	**4.044**
Aporcelaimellus	Alliphis	**0.548**	**0.053**	**4.889**	**3.407**
Aporcelaimellus	Arctoseius	**0.548**	**-0.152**	**4.889**	**2.453**
Aporcelaimellus	Dendrolaelaps	**0.548**	**0.027**	**4.889**	**2.821**
Aporcelaimellus	Dendroseius	**0.548**	**-0.310**	**4.889**	**1.976**
Aporcelaimellus	Lasioseius	**0.548**	**0.114**	**4.889**	**1.976**
Aporcelaimellus	Lysigamasus	**0.548**	**0.407**	**4.889**	**1.976**
Aporcelaimellus	Parasitus	**0.548**	**0.859**	**4.889**	**2.277**

Aporcelaimellus	Pergamasus	**0.548**	**1.081**	**4.889**	**1.976**
Aporcelaimellus	Rhodacarellus	**0.548**	**-0.310**	**4.889**	**2.930**
Aporcelaimellus	Aporcelaimellus	**0.548**	**0.548**	**4.889**	**4.889**
Aporcelaimellus	Dorylaimoidea	**0.548**	**-0.604**	**4.889**	**4.345**
Aporcelaimellus	Qudsianematidae	**0.548**	**-0.207**	**4.889**	**4.044**
Aporcelaimellus	Eupodes	**0.548**	**0.005**	**4.889**	**3.090**
Aporcelaimellus	Mesostigmata	**0.548**	**-0.411**	**4.889**	**2.277**
Aporcelaimellus	Scutacarus	**0.548**	**-0.608**	**4.889**	**3.481**
Aporcelaimellus	Stigmaeidae	**0.548**	**0.229**	**4.889**	**2.578**
Dorylaimoidea	Tripyla	**-0.604**	**-0.420**	**4.345**	**4.044**
Dorylaimoidea	Alliphis	**-0.604**	**0.053**	**4.345**	**3.407**
Dorylaimoidea	Arctoseius	**-0.604**	**-0.152**	**4.345**	**2.453**
Dorylaimoidea	Dendrolaelaps	**-0.604**	**0.027**	**4.345**	**2.821**
Dorylaimoidea	Dendroseius	**-0.604**	**-0.310**	**4.345**	**1.976**
Dorylaimoidea	Lasioseius	**-0.604**	**0.114**	**4.345**	**1.976**
Dorylaimoidea	Lysigamasus	**-0.604**	**0.407**	**4.345**	**1.976**
Dorylaimoidea	Parasitus	**-0.604**	**0.859**	**4.345**	**2.277**
Dorylaimoidea	Pergamasus	**-0.604**	**1.081**	**4.345**	**1.976**
Dorylaimoidea	Rhodacarellus	**-0.604**	**-0.310**	**4.345**	**2.930**
Dorylaimoidea	Aporcelaimellus	**-0.604**	**0.548**	**4.345**	**4.889**
Dorylaimoidea	Dorylaimoidea	**-0.604**	**-0.604**	**4.345**	**4.345**
Dorylaimoidea	Qudsianematidae	**-0.604**	**-0.207**	**4.345**	**4.044**
Dorylaimoidea	Eupodes	**-0.604**	**0.005**	**4.345**	**3.090**
Dorylaimoidea	Mesostigmata	**-0.604**	**-0.411**	**4.345**	**2.277**
Dorylaimoidea	Scutacarus	**-0.604**	**-0.608**	**4.345**	**3.481**
Dorylaimoidea	Stigmaeidae	**-0.604**	**0.229**	**4.345**	**2.578**
Qudsianematidae	Tripyla	**-0.207**	**-0.420**	**4.044**	**4.044**
Qudsianematidae	Alliphis	**-0.207**	**0.053**	**4.044**	**3.407**
Qudsianematidae	Arctoseius	**-0.207**	**-0.152**	**4.044**	**2.453**
Qudsianematidae	Dendrolaelaps	**-0.207**	**0.027**	**4.044**	**2.821**
Qudsianematidae	Dendroseius	**-0.207**	**-0.310**	**4.044**	**1.976**
Qudsianematidae	Lasioseius	**-0.207**	**0.114**	**4.044**	**1.976**
Qudsianematidae	Lysigamasus	**-0.207**	**0.407**	**4.044**	**1.976**
Qudsianematidae	Parasitus	**-0.207**	**0.859**	**4.044**	**2.277**
Qudsianematidae	Pergamasus	**-0.207**	**1.081**	**4.044**	**1.976**
Qudsianematidae	Rhodacarellus	**-0.207**	**-0.310**	**4.044**	**2.930**
Qudsianematidae	Aporcelaimellus	**-0.207**	**0.548**	**4.044**	**4.889**
Qudsianematidae	Dorylaimoidea	**-0.207**	**-0.604**	**4.044**	**4.345**
Qudsianematidae	Qudsianematidae	**-0.207**	**-0.207**	**4.044**	**4.044**
Qudsianematidae	Eupodes	**-0.207**	**0.005**	**4.044**	**3.090**
Qudsianematidae	Mesostigmata	**-0.207**	**-0.411**	**4.044**	**2.277**
Qudsianematidae	Scutacarus	**-0.207**	**-0.608**	**4.044**	**3.481**
Qudsianematidae	Stigmaeidae	**-0.207**	**0.229**	**4.044**	**2.578**
Eupodes	Bdella	**0.005**	**0.816**	**3.090**	**1.976**
Eupodes	Arctoseius	**0.005**	**-0.152**	**3.090**	**2.453**
Eupodes	Dendrolaelaps	**0.005**	**0.027**	**3.090**	**2.821**
Eupodes	Dendroseius	**0.005**	**-0.310**	**3.090**	**1.976**
Eupodes	Lasioseius	**0.005**	**0.114**	**3.090**	**1.976**
Eupodes	Lysigamasus	**0.005**	**0.407**	**3.090**	**1.976**
Eupodes	Parasitus	**0.005**	**0.859**	**3.090**	**2.277**
Eupodes	Pergamasus	**0.005**	**1.081**	**3.090**	**1.976**

Eupodes	Rhodacarellus	**0.005**	**-0.310**	**3.090**	**2.930**
Eupodes	Aporcelaimellus	**0.005**	**0.548**	**3.090**	**4.889**
Eupodes	Dorylaimoidea	**0.005**	**-0.604**	**3.090**	**4.345**
Eupodes	Qudsianematidae	**0.005**	**-0.207**	**3.090**	**4.044**
Eupodes	Eupodes	**0.005**	**0.005**	**3.090**	**3.090**
Eupodes	Mesostigmata	**0.005**	**-0.411**	**3.090**	**2.277**
Eupodes	Scutacarus	**0.005**	**-0.608**	**3.090**	**3.481**
Eupodes	Stigmaeidae	**0.005**	**0.229**	**3.090**	**2.578**
Mesostigmata	Bdella	**-0.411**	**0.816**	**2.277**	**1.976**
Mesostigmata	Arctoseius	**-0.411**	**-0.152**	**2.277**	**2.453**
Mesostigmata	Dendrolaelaps	**-0.411**	**0.027**	**2.277**	**2.821**
Mesostigmata	Dendroseius	**-0.411**	**-0.310**	**2.277**	**1.976**
Mesostigmata	Lasioseius	**-0.411**	**0.114**	**2.277**	**1.976**
Mesostigmata	Lysigamasus	**-0.411**	**0.407**	**2.277**	**1.976**
Mesostigmata	Parasitus	**-0.411**	**0.859**	**2.277**	**2.277**
Mesostigmata	Pergamasus	**-0.411**	**1.081**	**2.277**	**1.976**
Mesostigmata	Rhodacarellus	**-0.411**	**-0.310**	**2.277**	**2.930**
Mesostigmata	Aporcelaimellus	**-0.411**	**0.548**	**2.277**	**4.889**
Mesostigmata	Dorylaimoidea	**-0.411**	**-0.604**	**2.277**	**4.345**
Mesostigmata	Qudsianematidae	**-0.411**	**-0.207**	**2.277**	**4.044**
Mesostigmata	Eupodes	**-0.411**	**0.005**	**2.277**	**3.090**
Mesostigmata	Mesostigmata	**-0.411**	**-0.411**	**2.277**	**2.277**
Mesostigmata	Scutacarus	**-0.411**	**-0.608**	**2.277**	**3.481**
Mesostigmata	Stigmaeidae	**-0.411**	**0.229**	**2.277**	**2.578**
Scutacarus	Bdella	**-0.608**	**0.816**	**3.481**	**1.976**
Scutacarus	Arctoseius	**-0.608**	**-0.152**	**3.481**	**2.453**
Scutacarus	Dendrolaelaps	**-0.608**	**0.027**	**3.481**	**2.821**
Scutacarus	Dendroseius	**-0.608**	**-0.310**	**3.481**	**1.976**
Scutacarus	Lasioseius	**-0.608**	**0.114**	**3.481**	**1.976**
Scutacarus	Lysigamasus	**-0.608**	**0.407**	**3.481**	**1.976**
Scutacarus	Parasitus	**-0.608**	**0.859**	**3.481**	**2.277**
Scutacarus	Pergamasus	**-0.608**	**1.081**	**3.481**	**1.976**
Scutacarus	Rhodacarellus	**-0.608**	**-0.310**	**3.481**	**2.930**
Scutacarus	Aporcelaimellus	**-0.608**	**0.548**	**3.481**	**4.889**
Scutacarus	Dorylaimoidea	**-0.608**	**-0.604**	**3.481**	**4.345**
Scutacarus	Qudsianematidae	**-0.608**	**-0.207**	**3.481**	**4.044**
Scutacarus	Eupodes	**-0.608**	**0.005**	**3.481**	**3.090**
Scutacarus	Mesostigmata	**-0.608**	**-0.411**	**3.481**	**2.277**
Scutacarus	Scutacarus	**-0.608**	**-0.608**	**3.481**	**3.481**
Scutacarus	Stigmaeidae	**-0.608**	**0.229**	**3.481**	**2.578**
Stigmaeidae	Bdella	**0.229**	**0.816**	**2.578**	**1.976**
Stigmaeidae	Arctoseius	**0.229**	**-0.152**	**2.578**	**2.453**
Stigmaeidae	Dendrolaelaps	**0.229**	**0.027**	**2.578**	**2.821**
Stigmaeidae	Dendroseius	**0.229**	**-0.310**	**2.578**	**1.976**
Stigmaeidae	Lasioseius	**0.229**	**0.114**	**2.578**	**1.976**
Stigmaeidae	Lysigamasus	**0.229**	**0.407**	**2.578**	**1.976**
Stigmaeidae	Parasitus	**0.229**	**0.859**	**2.578**	**2.277**
Stigmaeidae	Pergamasus	**0.229**	**1.081**	**2.578**	**1.976**
Stigmaeidae	Rhodacarellus	**0.229**	**-0.310**	**2.578**	**2.930**
Stigmaeidae	Aporcelaimellus	**0.229**	**0.548**	**2.578**	**4.889**
Stigmaeidae	Dorylaimoidea	**0.229**	**-0.604**	**2.578**	**4.345**

Stigmaeidae	Qudsianematidae	**0.229**	**-0.207**	**2.578**	**4.044**
Stigmaeidae	Eupodes	**0.229**	**0.005**	**2.578**	**3.090**
Stigmaeidae	Mesostigmata	**0.229**	**-0.411**	**2.578**	**2.277**
Stigmaeidae	Scutacarus	**0.229**	**-0.608**	**2.578**	**3.481**
Stigmaeidae	Stigmaeidae	**0.229**	**0.229**	**2.578**	**2.578**

Resource	Consumer	Mres	Mconsumer	Nres	Nconsumer
Dolichodoridae	Tripyla	**-0.885**	**-0.420**	**4.373**	**4.595**
Dolichodoridae	Alliphis	**-0.885**	**0.053**	**4.373**	**2.277**
Dolichodoridae	Cheiroseius	**-0.885**	**0.356**	**4.373**	**1.976**
Dolichodoridae	Dendrolaelaps	**-0.885**	**0.027**	**4.373**	**3.231**
Dolichodoridae	Lysigamasus	**-0.885**	**0.407**	**4.373**	**1.976**
Dolichodoridae	Macrocheles	**-0.885**	**0.761**	**4.373**	**1.976**
Dolichodoridae	Pergamasus	**-0.885**	**1.081**	**4.373**	**1.976**
Dolichodoridae	Uropoda	**-0.885**	**0.481**	**4.373**	**2.578**
Dolichodoridae	Aporcelaimellus	**-0.885**	**0.548**	**4.373**	**3.896**
Dolichodoridae	Dorylaimoidea	**-0.885**	**-0.604**	**4.373**	**4.975**
Dolichodoridae	Eudorylaimus	**-0.885**	**-0.166**	**4.373**	**3.896**
Dolichodoridae	Mesodorylaimus	**-0.885**	**-0.277**	**4.373**	**4.595**
Dolichodoridae	Eupodes	**-0.885**	**0.005**	**4.373**	**2.879**
Dolichodoridae	Mesostigmata	**-0.885**	**-0.411**	**4.373**	**1.976**
Dolichodoridae	Scutacarus	**-0.885**	**-0.608**	**4.373**	**3.055**
Helicotylenchus	Tripyla	**-0.792**	**-0.420**	**4.896**	**4.595**
Helicotylenchus	Alliphis	**-0.792**	**0.053**	**4.896**	**2.277**
Helicotylenchus	Cheiroseius	**-0.792**	**0.356**	**4.896**	**1.976**
Helicotylenchus	Dendrolaelaps	**-0.792**	**0.027**	**4.896**	**3.231**
Helicotylenchus	Lysigamasus	**-0.792**	**0.407**	**4.896**	**1.976**
Helicotylenchus	Macrocheles	**-0.792**	**0.761**	**4.896**	**1.976**
Helicotylenchus	Pergamasus	**-0.792**	**1.081**	**4.896**	**1.976**
Helicotylenchus	Uropoda	**-0.792**	**0.481**	**4.896**	**2.578**
Helicotylenchus	Aporcelaimellus	**-0.792**	**0.548**	**4.896**	**3.896**
Helicotylenchus	Dorylaimoidea	**-0.792**	**-0.604**	**4.896**	**4.975**
Helicotylenchus	Eudorylaimus	**-0.792**	**-0.166**	**4.896**	**3.896**
Helicotylenchus	Mesodorylaimus	**-0.792**	**-0.277**	**4.896**	**4.595**
Helicotylenchus	Eupodes	**-0.792**	**0.005**	**4.896**	**2.879**
Helicotylenchus	Mesostigmata	**-0.792**	**-0.411**	**4.896**	**1.976**
Helicotylenchus	Scutacarus	**-0.792**	**-0.608**	**4.896**	**3.055**
Heterodera	Tripyla	**-0.883**	**-0.420**	**4.197**	**4.595**
Heterodera	Alliphis	**-0.883**	**0.053**	**4.197**	**2.277**
Heterodera	Cheiroseius	**-0.883**	**0.356**	**4.197**	**1.976**
Heterodera	Dendrolaelaps	**-0.883**	**0.027**	**4.197**	**3.231**
Heterodera	Lysigamasus	**-0.883**	**0.407**	**4.197**	**1.976**
Heterodera	Macrocheles	**-0.883**	**0.761**	**4.197**	**1.976**
Heterodera	Pergamasus	**-0.883**	**1.081**	**4.197**	**1.976**
Heterodera	Uropoda	**-0.883**	**0.481**	**4.197**	**2.578**
Heterodera	Aporcelaimellus	**-0.883**	**0.548**	**4.197**	**3.896**
Heterodera	Dorylaimoidea	**-0.883**	**-0.604**	**4.197**	**4.975**
Heterodera	Eudorylaimus	**-0.883**	**-0.166**	**4.197**	**3.896**
Heterodera	Mesodorylaimus	**-0.883**	**-0.277**	**4.197**	**4.595**
Heterodera	Eupodes	**-0.883**	**0.005**	**4.197**	**2.879**
Heterodera	Mesostigmata	**-0.883**	**-0.411**	**4.197**	**1.976**
Heterodera	Scutacarus	**-0.883**	**-0.608**	**4.197**	**3.055**
Malenchus	Tripyla	**-1.330**	**-0.420**	**5.072**	**4.595**
Malenchus	Alliphis	**-1.330**	**0.053**	**5.072**	**2.277**
Malenchus	Cheiroseius	**-1.330**	**0.356**	**5.072**	**1.976**
Malenchus	Dendrolaelaps	**-1.330**	**0.027**	**5.072**	**3.231**
Malenchus	Lysigamasus	**-1.330**	**0.407**	**5.072**	**1.976**

Malenchus	Macrocheles	**-1.330**	**0.761**	**5.072**	**1.976**
Malenchus	Pergamasus	**-1.330**	**1.081**	**5.072**	**1.976**
Malenchus	Uropoda	**-1.330**	**0.481**	**5.072**	**2.578**
Malenchus	Aporcelaimellus	**-1.330**	**0.548**	**5.072**	**3.896**
Malenchus	Dorylaimoidea	**-1.330**	**-0.604**	**5.072**	**4.975**
Malenchus	Eudorylaimus	**-1.330**	**-0.166**	**5.072**	**3.896**
Malenchus	Mesodorylaimus	**-1.330**	**-0.277**	**5.072**	**4.595**
Malenchus	Eupodes	**-1.330**	**0.005**	**5.072**	**2.879**
Malenchus	Mesostigmata	**-1.330**	**-0.411**	**5.072**	**1.976**
Malenchus	Scutacarus	**-1.330**	**-0.608**	**5.072**	**3.055**
Paratylenchus	Tripyla	**-1.244**	**-0.420**	**4.197**	**4.595**
Paratylenchus	Alliphis	**-1.244**	**0.053**	**4.197**	**2.277**
Paratylenchus	Cheiroseius	**-1.244**	**0.356**	**4.197**	**1.976**
Paratylenchus	Dendrolaelaps	**-1.244**	**0.027**	**4.197**	**3.231**
Paratylenchus	Lysigamasus	**-1.244**	**0.407**	**4.197**	**1.976**
Paratylenchus	Macrocheles	**-1.244**	**0.761**	**4.197**	**1.976**
Paratylenchus	Pergamasus	**-1.244**	**1.081**	**4.197**	**1.976**
Paratylenchus	Uropoda	**-1.244**	**0.481**	**4.197**	**2.578**
Paratylenchus	Aporcelaimellus	**-1.244**	**0.548**	**4.197**	**3.896**
Paratylenchus	Dorylaimoidea	**-1.244**	**-0.604**	**4.197**	**4.975**
Paratylenchus	Eudorylaimus	**-1.244**	**-0.166**	**4.197**	**3.896**
Paratylenchus	Mesodorylaimus	**-1.244**	**-0.277**	**4.197**	**4.595**
Paratylenchus	Eupodes	**-1.244**	**0.005**	**4.197**	**2.879**
Paratylenchus	Mesostigmata	**-1.244**	**-0.411**	**4.197**	**1.976**
Paratylenchus	Scutacarus	**-1.244**	**-0.608**	**4.197**	**3.055**
Tylenchorhynchus	Tripyla	**-0.664**	**-0.420**	**4.197**	**4.595**
Tylenchorhynchus	Alliphis	**-0.664**	**0.053**	**4.197**	**2.277**
Tylenchorhynchus	Cheiroseius	**-0.664**	**0.356**	**4.197**	**1.976**
Tylenchorhynchus	Dendrolaelaps	**-0.664**	**0.027**	**4.197**	**3.231**
Tylenchorhynchus	Lysigamasus	**-0.664**	**0.407**	**4.197**	**1.976**
Tylenchorhynchus	Macrocheles	**-0.664**	**0.761**	**4.197**	**1.976**
Tylenchorhynchus	Pergamasus	**-0.664**	**1.081**	**4.197**	**1.976**
Tylenchorhynchus	Uropoda	**-0.664**	**0.481**	**4.197**	**2.578**
Tylenchorhynchus	Aporcelaimellus	**-0.664**	**0.548**	**4.197**	**3.896**
Tylenchorhynchus	Dorylaimoidea	**-0.664**	**-0.604**	**4.197**	**4.975**
Tylenchorhynchus	Eudorylaimus	**-0.664**	**-0.166**	**4.197**	**3.896**
Tylenchorhynchus	Mesodorylaimus	**-0.664**	**-0.277**	**4.197**	**4.595**
Tylenchorhynchus	Eupodes	**-0.664**	**0.005**	**4.197**	**2.879**
Tylenchorhynchus	Mesostigmata	**-0.664**	**-0.411**	**4.197**	**1.976**
Tylenchorhynchus	Scutacarus	**-0.664**	**-0.608**	**4.197**	**3.055**
Tydeidae	Cheiroseius	**-0.608**	**0.356**	**1.976**	**1.976**
Tydeidae	Dendrolaelaps	**-0.608**	**0.027**	**1.976**	**3.231**
Tydeidae	Lysigamasus	**-0.608**	**0.407**	**1.976**	**1.976**
Tydeidae	Macrocheles	**-0.608**	**0.761**	**1.976**	**1.976**
Tydeidae	Pergamasus	**-0.608**	**1.081**	**1.976**	**1.976**
Tydeidae	Uropoda	**-0.608**	**0.481**	**1.976**	**2.578**
Tydeidae	Aporcelaimellus	**-0.608**	**0.548**	**1.976**	**3.896**
Tydeidae	Dorylaimoidea	**-0.608**	**-0.604**	**1.976**	**4.975**
Tydeidae	Eudorylaimus	**-0.608**	**-0.166**	**1.976**	**3.896**
Tydeidae	Mesodorylaimus	**-0.608**	**-0.277**	**1.976**	**4.595**
Tydeidae	Eupodes	**-0.608**	**0.005**	**1.976**	**2.879**

Tydeidae	Mesostigmata	**-0.608**	**-0.411**	**1.976**	**1.976**
Tydeidae	Scutacarus	**-0.608**	**-0.608**	**1.976**	**3.055**
Sminthuridae	Cheiroseius	**-0.608**	**0.356**	**2.578**	**1.976**
Sminthuridae	Dendrolaelaps	**-0.608**	**0.027**	**2.578**	**3.231**
Sminthuridae	Lysigamasus	**-0.608**	**0.407**	**2.578**	**1.976**
Sminthuridae	Macrocheles	**-0.608**	**0.761**	**2.578**	**1.976**
Sminthuridae	Pergamasus	**-0.608**	**1.081**	**2.578**	**1.976**
Sminthuridae	Uropoda	**-0.608**	**0.481**	**2.578**	**2.578**
Sminthuridae	Aporcelaimellus	**-0.608**	**0.548**	**2.578**	**3.896**
Sminthuridae	Dorylaimoidea	**-0.608**	**-0.604**	**2.578**	**4.975**
Sminthuridae	Eudorylaimus	**-0.608**	**-0.166**	**2.578**	**3.896**
Sminthuridae	Mesodorylaimus	**-0.608**	**-0.277**	**2.578**	**4.595**
Sminthuridae	Eupodes	**-0.608**	**0.005**	**2.578**	**2.879**
Sminthuridae	Mesostigmata	**-0.608**	**-0.411**	**2.578**	**1.976**
Sminthuridae	Scutacarus	**-0.608**	**-0.608**	**2.578**	**3.055**
Sminthurinus	Cheiroseius	**0.618**	**0.356**	**3.556**	**1.976**
Sminthurinus	Dendrolaelaps	**0.618**	**0.027**	**3.556**	**3.231**
Sminthurinus	Lysigamasus	**0.618**	**0.407**	**3.556**	**1.976**
Sminthurinus	Macrocheles	**0.618**	**0.761**	**3.556**	**1.976**
Sminthurinus	Pergamasus	**0.618**	**1.081**	**3.556**	**1.976**
Sminthurinus	Uropoda	**0.618**	**0.481**	**3.556**	**2.578**
Sminthurinus	Aporcelaimellus	**0.618**	**0.548**	**3.556**	**3.896**
Sminthurinus	Dorylaimoidea	**0.618**	**-0.604**	**3.556**	**4.975**
Sminthurinus	Eudorylaimus	**0.618**	**-0.166**	**3.556**	**3.896**
Sminthurinus	Mesodorylaimus	**0.618**	**-0.277**	**3.556**	**4.595**
Sminthurinus	Eupodes	**0.618**	**0.005**	**3.556**	**2.879**
Sminthurinus	Mesostigmata	**0.618**	**-0.411**	**3.556**	**1.976**
Sminthurinus	Scutacarus	**0.618**	**-0.608**	**3.556**	**3.055**
Sminthurus	Cheiroseius	**1.429**	**0.356**	**2.277**	**1.976**
Sminthurus	Dendrolaelaps	**1.429**	**0.027**	**2.277**	**3.231**
Sminthurus	Lysigamasus	**1.429**	**0.407**	**2.277**	**1.976**
Sminthurus	Macrocheles	**1.429**	**0.761**	**2.277**	**1.976**
Sminthurus	Pergamasus	**1.429**	**1.081**	**2.277**	**1.976**
Sminthurus	Uropoda	**1.429**	**0.481**	**2.277**	**2.578**
Sminthurus	Aporcelaimellus	**1.429**	**0.548**	**2.277**	**3.896**
Sminthurus	Dorylaimoidea	**1.429**	**-0.604**	**2.277**	**4.975**
Sminthurus	Eudorylaimus	**1.429**	**-0.166**	**2.277**	**3.896**
Sminthurus	Mesodorylaimus	**1.429**	**-0.277**	**2.277**	**4.595**
Sminthurus	Eupodes	**1.429**	**0.005**	**2.277**	**2.879**
Sminthurus	Mesostigmata	**1.429**	**-0.411**	**2.277**	**1.976**
Sminthurus	Scutacarus	**1.429**	**-0.608**	**2.277**	**3.055**
Sphaeridia	Cheiroseius	**0.202**	**0.356**	**2.821**	**1.976**
Sphaeridia	Dendrolaelaps	**0.202**	**0.027**	**2.821**	**3.231**
Sphaeridia	Lysigamasus	**0.202**	**0.407**	**2.821**	**1.976**
Sphaeridia	Macrocheles	**0.202**	**0.761**	**2.821**	**1.976**
Sphaeridia	Pergamasus	**0.202**	**1.081**	**2.821**	**1.976**
Sphaeridia	Uropoda	**0.202**	**0.481**	**2.821**	**2.578**
Sphaeridia	Aporcelaimellus	**0.202**	**0.548**	**2.821**	**3.896**
Sphaeridia	Dorylaimoidea	**0.202**	**-0.604**	**2.821**	**4.975**
Sphaeridia	Eudorylaimus	**0.202**	**-0.166**	**2.821**	**3.896**
Sphaeridia	Mesodorylaimus	**0.202**	**-0.277**	**2.821**	**4.595**

Sphaeridia	Eupodes	**0.202**	**0.005**	**2.821**	**2.879**
Sphaeridia	Mesostigmata	**0.202**	**-0.411**	**2.821**	**1.976**
Sphaeridia	Scutacarus	**0.202**	**-0.608**	**2.821**	**3.055**
Aphelenchoides	Tripyla	**-1.496**	**-0.420**	**4.595**	**4.595**
Aphelenchoides	Alliphis	**-1.496**	**0.053**	**4.595**	**2.277**
Aphelenchoides	Cheiroseius	**-1.496**	**0.356**	**4.595**	**1.976**
Aphelenchoides	Dendrolaelaps	**-1.496**	**0.027**	**4.595**	**3.231**
Aphelenchoides	Lysigamasus	**-1.496**	**0.407**	**4.595**	**1.976**
Aphelenchoides	Macrocheles	**-1.496**	**0.761**	**4.595**	**1.976**
Aphelenchoides	Pergamasus	**-1.496**	**1.081**	**4.595**	**1.976**
Aphelenchoides	Uropoda	**-1.496**	**0.481**	**4.595**	**2.578**
Aphelenchoides	Aporcelaimellus	**-1.496**	**0.548**	**4.595**	**3.896**
Aphelenchoides	Dorylaimoidea	**-1.496**	**-0.604**	**4.595**	**4.975**
Aphelenchoides	Eudorylaimus	**-1.496**	**-0.166**	**4.595**	**3.896**
Aphelenchoides	Mesodorylaimus	**-1.496**	**-0.277**	**4.595**	**4.595**
Aphelenchoides	Eupodes	**-1.496**	**0.005**	**4.595**	**2.879**
Aphelenchoides	Mesostigmata	**-1.496**	**-0.411**	**4.595**	**1.976**
Aphelenchoides	Scutacarus	**-1.496**	**-0.608**	**4.595**	**3.055**
Tylenchidae	Tripyla	**-1.360**	**-0.420**	**5.197**	**4.595**
Tylenchidae	Alliphis	**-1.360**	**0.053**	**5.197**	**2.277**
Tylenchidae	Cheiroseius	**-1.360**	**0.356**	**5.197**	**1.976**
Tylenchidae	Dendrolaelaps	**-1.360**	**0.027**	**5.197**	**3.231**
Tylenchidae	Lysigamasus	**-1.360**	**0.407**	**5.197**	**1.976**
Tylenchidae	Macrocheles	**-1.360**	**0.761**	**5.197**	**1.976**
Tylenchidae	Pergamasus	**-1.360**	**1.081**	**5.197**	**1.976**
Tylenchidae	Uropoda	**-1.360**	**0.481**	**5.197**	**2.578**
Tylenchidae	Aporcelaimellus	**-1.360**	**0.548**	**5.197**	**3.896**
Tylenchidae	Dorylaimoidea	**-1.360**	**-0.604**	**5.197**	**4.975**
Tylenchidae	Eudorylaimus	**-1.360**	**-0.166**	**5.197**	**3.896**
Tylenchidae	Mesodorylaimus	**-1.360**	**-0.277**	**5.197**	**4.595**
Tylenchidae	Eupodes	**-1.360**	**0.005**	**5.197**	**2.879**
Tylenchidae	Mesostigmata	**-1.360**	**-0.411**	**5.197**	**1.976**
Tylenchidae	Scutacarus	**-1.360**	**-0.608**	**5.197**	**3.055**
Pygmephorus	Cheiroseius	**-0.376**	**0.356**	**2.821**	**1.976**
Pygmephorus	Dendrolaelaps	**-0.376**	**0.027**	**2.821**	**3.231**
Pygmephorus	Lysigamasus	**-0.376**	**0.407**	**2.821**	**1.976**
Pygmephorus	Macrocheles	**-0.376**	**0.761**	**2.821**	**1.976**
Pygmephorus	Pergamasus	**-0.376**	**1.081**	**2.821**	**1.976**
Pygmephorus	Uropoda	**-0.376**	**0.481**	**2.821**	**2.578**
Pygmephorus	Aporcelaimellus	**-0.376**	**0.548**	**2.821**	**3.896**
Pygmephorus	Dorylaimoidea	**-0.376**	**-0.604**	**2.821**	**4.975**
Pygmephorus	Eudorylaimus	**-0.376**	**-0.166**	**2.821**	**3.896**
Pygmephorus	Mesodorylaimus	**-0.376**	**-0.277**	**2.821**	**4.595**
Pygmephorus	Eupodes	**-0.376**	**0.005**	**2.821**	**2.879**
Pygmephorus	Mesostigmata	**-0.376**	**-0.411**	**2.821**	**1.976**
Pygmephorus	Scutacarus	**-0.376**	**-0.608**	**2.821**	**3.055**
Tyrophagus	Cheiroseius	**0.005**	**0.356**	**1.976**	**1.976**
Tyrophagus	Dendrolaelaps	**0.005**	**0.027**	**1.976**	**3.231**
Tyrophagus	Lysigamasus	**0.005**	**0.407**	**1.976**	**1.976**
Tyrophagus	Macrocheles	**0.005**	**0.761**	**1.976**	**1.976**
Tyrophagus	Pergamasus	**0.005**	**1.081**	**1.976**	**1.976**

Tyrophagus	Uropoda	**0.005**	**0.481**	**1.976**	**2.578**
Tyrophagus	Aporcelaimellus	**0.005**	**0.548**	**1.976**	**3.896**
Tyrophagus	Dorylaimoidea	**0.005**	**-0.604**	**1.976**	**4.975**
Tyrophagus	Eudorylaimus	**0.005**	**-0.166**	**1.976**	**3.896**
Tyrophagus	Mesodorylaimus	**0.005**	**-0.277**	**1.976**	**4.595**
Tyrophagus	Eupodes	**0.005**	**0.005**	**1.976**	**2.879**
Tyrophagus	Mesostigmata	**0.005**	**-0.411**	**1.976**	**1.976**
Tyrophagus	Scutacarus	**0.005**	**-0.608**	**1.976**	**3.055**
Friesea	Cheiroseius	**0.434**	**0.356**	**2.578**	**1.976**
Friesea	Dendrolaelaps	**0.434**	**0.027**	**2.578**	**3.231**
Friesea	Lysigamasus	**0.434**	**0.407**	**2.578**	**1.976**
Friesea	Macrocheles	**0.434**	**0.761**	**2.578**	**1.976**
Friesea	Pergamasus	**0.434**	**1.081**	**2.578**	**1.976**
Friesea	Uropoda	**0.434**	**0.481**	**2.578**	**2.578**
Friesea	Aporcelaimellus	**0.434**	**0.548**	**2.578**	**3.896**
Friesea	Dorylaimoidea	**0.434**	**-0.604**	**2.578**	**4.975**
Friesea	Eudorylaimus	**0.434**	**-0.166**	**2.578**	**3.896**
Friesea	Mesodorylaimus	**0.434**	**-0.277**	**2.578**	**4.595**
Friesea	Eupodes	**0.434**	**0.005**	**2.578**	**2.879**
Friesea	Mesostigmata	**0.434**	**-0.411**	**2.578**	**1.976**
Friesea	Scutacarus	**0.434**	**-0.608**	**2.578**	**3.055**
Isotoma	Cheiroseius	**1.898**	**0.356**	**3.684**	**1.976**
Isotoma	Dendrolaelaps	**1.898**	**0.027**	**3.684**	**3.231**
Isotoma	Lysigamasus	**1.898**	**0.407**	**3.684**	**1.976**
Isotoma	Macrocheles	**1.898**	**0.761**	**3.684**	**1.976**
Isotoma	Pergamasus	**1.898**	**1.081**	**3.684**	**1.976**
Isotoma	Uropoda	**1.898**	**0.481**	**3.684**	**2.578**
Isotoma	Aporcelaimellus	**1.898**	**0.548**	**3.684**	**3.896**
Isotoma	Dorylaimoidea	**1.898**	**-0.604**	**3.684**	**4.975**
Isotoma	Eudorylaimus	**1.898**	**-0.166**	**3.684**	**3.896**
Isotoma	Mesodorylaimus	**1.898**	**-0.277**	**3.684**	**4.595**
Isotoma	Eupodes	**1.898**	**0.005**	**3.684**	**2.879**
Isotoma	Mesostigmata	**1.898**	**-0.411**	**3.684**	**1.976**
Isotoma	Scutacarus	**1.898**	**-0.608**	**3.684**	**3.055**
Isotomiella	Cheiroseius	**0.816**	**0.356**	**2.277**	**1.976**
Isotomiella	Dendrolaelaps	**0.816**	**0.027**	**2.277**	**3.231**
Isotomiella	Lysigamasus	**0.816**	**0.407**	**2.277**	**1.976**
Isotomiella	Macrocheles	**0.816**	**0.761**	**2.277**	**1.976**
Isotomiella	Pergamasus	**0.816**	**1.081**	**2.277**	**1.976**
Isotomiella	Uropoda	**0.816**	**0.481**	**2.277**	**2.578**
Isotomiella	Aporcelaimellus	**0.816**	**0.548**	**2.277**	**3.896**
Isotomiella	Dorylaimoidea	**0.816**	**-0.604**	**2.277**	**4.975**
Isotomiella	Eudorylaimus	**0.816**	**-0.166**	**2.277**	**3.896**
Isotomiella	Mesodorylaimus	**0.816**	**-0.277**	**2.277**	**4.595**
Isotomiella	Eupodes	**0.816**	**0.005**	**2.277**	**2.879**
Isotomiella	Mesostigmata	**0.816**	**-0.411**	**2.277**	**1.976**
Isotomiella	Scutacarus	**0.816**	**-0.608**	**2.277**	**3.055**
Isotomurus	Cheiroseius	**1.787**	**0.356**	**3.277**	**1.976**
Isotomurus	Dendrolaelaps	**1.787**	**0.027**	**3.277**	**3.231**
Isotomurus	Lysigamasus	**1.787**	**0.407**	**3.277**	**1.976**
Isotomurus	Macrocheles	**1.787**	**0.761**	**3.277**	**1.976**

Isotomurus	Pergamasus	**1.787**	**1.081**	**3.277**	**1.976**
Isotomurus	Uropoda	**1.787**	**0.481**	**3.277**	**2.578**
Isotomurus	Aporcelaimellus	**1.787**	**0.548**	**3.277**	**3.896**
Isotomurus	Dorylaimoidea	**1.787**	**-0.604**	**3.277**	**4.975**
Isotomurus	Eudorylaimus	**1.787**	**-0.166**	**3.277**	**3.896**
Isotomurus	Mesodorylaimus	**1.787**	**-0.277**	**3.277**	**4.595**
Isotomurus	Eupodes	**1.787**	**0.005**	**3.277**	**2.879**
Isotomurus	Mesostigmata	**1.787**	**-0.411**	**3.277**	**1.976**
Isotomurus	Scutacarus	**1.787**	**-0.608**	**3.277**	**3.055**
Lepidocyrtus	Cheiroseius	**1.231**	**0.356**	**2.675**	**1.976**
Lepidocyrtus	Dendrolaelaps	**1.231**	**0.027**	**2.675**	**3.231**
Lepidocyrtus	Lysigamasus	**1.231**	**0.407**	**2.675**	**1.976**
Lepidocyrtus	Macrocheles	**1.231**	**0.761**	**2.675**	**1.976**
Lepidocyrtus	Pergamasus	**1.231**	**1.081**	**2.675**	**1.976**
Lepidocyrtus	Uropoda	**1.231**	**0.481**	**2.675**	**2.578**
Lepidocyrtus	Aporcelaimellus	**1.231**	**0.548**	**2.675**	**3.896**
Lepidocyrtus	Dorylaimoidea	**1.231**	**-0.604**	**2.675**	**4.975**
Lepidocyrtus	Eudorylaimus	**1.231**	**-0.166**	**2.675**	**3.896**
Lepidocyrtus	Mesodorylaimus	**1.231**	**-0.277**	**2.675**	**4.595**
Lepidocyrtus	Eupodes	**1.231**	**0.005**	**2.675**	**2.879**
Lepidocyrtus	Mesostigmata	**1.231**	**-0.411**	**2.675**	**1.976**
Lepidocyrtus	Scutacarus	**1.231**	**-0.608**	**2.675**	**3.055**
Proisotoma	Cheiroseius	**0.770**	**0.356**	**2.754**	**1.976**
Proisotoma	Dendrolaelaps	**0.770**	**0.027**	**2.754**	**3.231**
Proisotoma	Lysigamasus	**0.770**	**0.407**	**2.754**	**1.976**
Proisotoma	Macrocheles	**0.770**	**0.761**	**2.754**	**1.976**
Proisotoma	Pergamasus	**0.770**	**1.081**	**2.754**	**1.976**
Proisotoma	Uropoda	**0.770**	**0.481**	**2.754**	**2.578**
Proisotoma	Aporcelaimellus	**0.770**	**0.548**	**2.754**	**3.896**
Proisotoma	Dorylaimoidea	**0.770**	**-0.604**	**2.754**	**4.975**
Proisotoma	Eudorylaimus	**0.770**	**-0.166**	**2.754**	**3.896**
Proisotoma	Mesodorylaimus	**0.770**	**-0.277**	**2.754**	**4.595**
Proisotoma	Eupodes	**0.770**	**0.005**	**2.754**	**2.879**
Proisotoma	Mesostigmata	**0.770**	**-0.411**	**2.754**	**1.976**
Proisotoma	Scutacarus	**0.770**	**-0.608**	**2.754**	**3.055**
Achaeta	Cheiroseius	**1.300**	**0.356**	**3.460**	**1.976**
Achaeta	Dendrolaelaps	**1.300**	**0.027**	**3.460**	**3.231**
Achaeta	Lysigamasus	**1.300**	**0.407**	**3.460**	**1.976**
Achaeta	Macrocheles	**1.300**	**0.761**	**3.460**	**1.976**
Achaeta	Pergamasus	**1.300**	**1.081**	**3.460**	**1.976**
Achaeta	Uropoda	**1.300**	**0.481**	**3.460**	**2.578**
Achaeta	Aporcelaimellus	**1.300**	**0.548**	**3.460**	**3.896**
Achaeta	Dorylaimoidea	**1.300**	**-0.604**	**3.460**	**4.975**
Achaeta	Eudorylaimus	**1.300**	**-0.166**	**3.460**	**3.896**
Achaeta	Mesodorylaimus	**1.300**	**-0.277**	**3.460**	**4.595**
Achaeta	Eupodes	**1.300**	**0.005**	**3.460**	**2.879**
Achaeta	Mesostigmata	**1.300**	**-0.411**	**3.460**	**1.976**
Achaeta	Scutacarus	**1.300**	**-0.608**	**3.460**	**3.055**
Fridericia	Cheiroseius	**1.985**	**0.356**	**4.023**	**1.976**
Fridericia	Dendrolaelaps	**1.985**	**0.027**	**4.023**	**3.231**
Fridericia	Lysigamasus	**1.985**	**0.407**	**4.023**	**1.976**

Fridericia	Macrocheles	**1.985**	**0.761**	**4.023**	**1.976**
Fridericia	Pergamasus	**1.985**	**1.081**	**4.023**	**1.976**
Fridericia	Uropoda	**1.985**	**0.481**	**4.023**	**2.578**
Fridericia	Aporcelaimellus	**1.985**	**0.548**	**4.023**	**3.896**
Fridericia	Dorylaimoidea	**1.985**	**-0.604**	**4.023**	**4.975**
Fridericia	Eudorylaimus	**1.985**	**-0.166**	**4.023**	**3.896**
Fridericia	Mesodorylaimus	**1.985**	**-0.277**	**4.023**	**4.595**
Fridericia	Eupodes	**1.985**	**0.005**	**4.023**	**2.879**
Fridericia	Mesostigmata	**1.985**	**-0.411**	**4.023**	**1.976**
Fridericia	Scutacarus	**1.985**	**-0.608**	**4.023**	**3.055**
Acrobeloides	Tripyla	**-1.171**	**-0.420**	**3.896**	**4.595**
Acrobeloides	Alliphis	**-1.171**	**0.053**	**3.896**	**2.277**
Acrobeloides	Cheiroseius	**-1.171**	**0.356**	**3.896**	**1.976**
Acrobeloides	Dendrolaelaps	**-1.171**	**0.027**	**3.896**	**3.231**
Acrobeloides	Lysigamasus	**-1.171**	**0.407**	**3.896**	**1.976**
Acrobeloides	Macrocheles	**-1.171**	**0.761**	**3.896**	**1.976**
Acrobeloides	Pergamasus	**-1.171**	**1.081**	**3.896**	**1.976**
Acrobeloides	Uropoda	**-1.171**	**0.481**	**3.896**	**2.578**
Acrobeloides	Aporcelaimellus	**-1.171**	**0.548**	**3.896**	**3.896**
Acrobeloides	Dorylaimoidea	**-1.171**	**-0.604**	**3.896**	**4.975**
Acrobeloides	Eudorylaimus	**-1.171**	**-0.166**	**3.896**	**3.896**
Acrobeloides	Mesodorylaimus	**-1.171**	**-0.277**	**3.896**	**4.595**
Acrobeloides	Eupodes	**-1.171**	**0.005**	**3.896**	**2.879**
Acrobeloides	Mesostigmata	**-1.171**	**-0.411**	**3.896**	**1.976**
Acrobeloides	Scutacarus	**-1.171**	**-0.608**	**3.896**	**3.055**
Cephalobidae	Tripyla	**-1.055**	**-0.420**	**4.498**	**4.595**
Cephalobidae	Alliphis	**-1.055**	**0.053**	**4.498**	**2.277**
Cephalobidae	Cheiroseius	**-1.055**	**0.356**	**4.498**	**1.976**
Cephalobidae	Dendrolaelaps	**-1.055**	**0.027**	**4.498**	**3.231**
Cephalobidae	Lysigamasus	**-1.055**	**0.407**	**4.498**	**1.976**
Cephalobidae	Macrocheles	**-1.055**	**0.761**	**4.498**	**1.976**
Cephalobidae	Pergamasus	**-1.055**	**1.081**	**4.498**	**1.976**
Cephalobidae	Uropoda	**-1.055**	**0.481**	**4.498**	**2.578**
Cephalobidae	Aporcelaimellus	**-1.055**	**0.548**	**4.498**	**3.896**
Cephalobidae	Dorylaimoidea	**-1.055**	**-0.604**	**4.498**	**4.975**
Cephalobidae	Eudorylaimus	**-1.055**	**-0.166**	**4.498**	**3.896**
Cephalobidae	Mesodorylaimus	**-1.055**	**-0.277**	**4.498**	**4.595**
Cephalobidae	Eupodes	**-1.055**	**0.005**	**4.498**	**2.879**
Cephalobidae	Mesostigmata	**-1.055**	**-0.411**	**4.498**	**1.976**
Cephalobidae	Scutacarus	**-1.055**	**-0.608**	**4.498**	**3.055**
Eucephalobus	Tripyla	**-0.855**	**-0.420**	**4.850**	**4.595**
Eucephalobus	Alliphis	**-0.855**	**0.053**	**4.850**	**2.277**
Eucephalobus	Cheiroseius	**-0.855**	**0.356**	**4.850**	**1.976**
Eucephalobus	Dendrolaelaps	**-0.855**	**0.027**	**4.850**	**3.231**
Eucephalobus	Lysigamasus	**-0.855**	**0.407**	**4.850**	**1.976**
Eucephalobus	Macrocheles	**-0.855**	**0.761**	**4.850**	**1.976**
Eucephalobus	Pergamasus	**-0.855**	**1.081**	**4.850**	**1.976**
Eucephalobus	Uropoda	**-0.855**	**0.481**	**4.850**	**2.578**
Eucephalobus	Aporcelaimellus	**-0.855**	**0.548**	**4.850**	**3.896**
Eucephalobus	Dorylaimoidea	**-0.855**	**-0.604**	**4.850**	**4.975**
Eucephalobus	Eudorylaimus	**-0.855**	**-0.166**	**4.850**	**3.896**

Eucephalobus	Mesodorylaimus	**-0.855**	**-0.277**	**4.850**	**4.595**
Eucephalobus	Eupodes	**-0.855**	**0.005**	**4.850**	**2.879**
Eucephalobus	Mesostigmata	**-0.855**	**-0.411**	**4.850**	**1.976**
Eucephalobus	Scutacarus	**-0.855**	**-0.608**	**4.850**	**3.055**
Metateratocephalus	Tripyla	**-1.506**	**-0.420**	**4.373**	**4.595**
Metateratocephalus	Alliphis	**-1.506**	**0.053**	**4.373**	**2.277**
Metateratocephalus	Cheiroseius	**-1.506**	**0.356**	**4.373**	**1.976**
Metateratocephalus	Dendrolaelaps	**-1.506**	**0.027**	**4.373**	**3.231**
Metateratocephalus	Lysigamasus	**-1.506**	**0.407**	**4.373**	**1.976**
Metateratocephalus	Macrocheles	**-1.506**	**0.761**	**4.373**	**1.976**
Metateratocephalus	Pergamasus	**-1.506**	**1.081**	**4.373**	**1.976**
Metateratocephalus	Uropoda	**-1.506**	**0.481**	**4.373**	**2.578**
Metateratocephalus	Aporcelaimellus	**-1.506**	**0.548**	**4.373**	**3.896**
Metateratocephalus	Dorylaimoidea	**-1.506**	**-0.604**	**4.373**	**4.975**
Metateratocephalus	Eudorylaimus	**-1.506**	**-0.166**	**4.373**	**3.896**
Metateratocephalus	Mesodorylaimus	**-1.506**	**-0.277**	**4.373**	**4.595**
Metateratocephalus	Eupodes	**-1.506**	**0.005**	**4.373**	**2.879**
Metateratocephalus	Mesostigmata	**-1.506**	**-0.411**	**4.373**	**1.976**
Metateratocephalus	Scutacarus	**-1.506**	**-0.608**	**4.373**	**3.055**
Plectus	Tripyla	**-0.583**	**-0.420**	**4.799**	**4.595**
Plectus	Alliphis	**-0.583**	**0.053**	**4.799**	**2.277**
Plectus	Cheiroseius	**-0.583**	**0.356**	**4.799**	**1.976**
Plectus	Dendrolaelaps	**-0.583**	**0.027**	**4.799**	**3.231**
Plectus	Lysigamasus	**-0.583**	**0.407**	**4.799**	**1.976**
Plectus	Macrocheles	**-0.583**	**0.761**	**4.799**	**1.976**
Plectus	Pergamasus	**-0.583**	**1.081**	**4.799**	**1.976**
Plectus	Uropoda	**-0.583**	**0.481**	**4.799**	**2.578**
Plectus	Aporcelaimellus	**-0.583**	**0.548**	**4.799**	**3.896**
Plectus	Dorylaimoidea	**-0.583**	**-0.604**	**4.799**	**4.975**
Plectus	Eudorylaimus	**-0.583**	**-0.166**	**4.799**	**3.896**
Plectus	Mesodorylaimus	**-0.583**	**-0.277**	**4.799**	**4.595**
Plectus	Eupodes	**-0.583**	**0.005**	**4.799**	**2.879**
Plectus	Mesostigmata	**-0.583**	**-0.411**	**4.799**	**1.976**
Plectus	Scutacarus	**-0.583**	**-0.608**	**4.799**	**3.055**
Prismatolaimus	Tripyla	**-1.280**	**-0.420**	**4.197**	**4.595**
Prismatolaimus	Alliphis	**-1.280**	**0.053**	**4.197**	**2.277**
Prismatolaimus	Cheiroseius	**-1.280**	**0.356**	**4.197**	**1.976**
Prismatolaimus	Dendrolaelaps	**-1.280**	**0.027**	**4.197**	**3.231**
Prismatolaimus	Lysigamasus	**-1.280**	**0.407**	**4.197**	**1.976**
Prismatolaimus	Macrocheles	**-1.280**	**0.761**	**4.197**	**1.976**
Prismatolaimus	Pergamasus	**-1.280**	**1.081**	**4.197**	**1.976**
Prismatolaimus	Uropoda	**-1.280**	**0.481**	**4.197**	**2.578**
Prismatolaimus	Aporcelaimellus	**-1.280**	**0.548**	**4.197**	**3.896**
Prismatolaimus	Dorylaimoidea	**-1.280**	**-0.604**	**4.197**	**4.975**
Prismatolaimus	Eudorylaimus	**-1.280**	**-0.166**	**4.197**	**3.896**
Prismatolaimus	Mesodorylaimus	**-1.280**	**-0.277**	**4.197**	**4.595**
Prismatolaimus	Eupodes	**-1.280**	**0.005**	**4.197**	**2.879**
Prismatolaimus	Mesostigmata	**-1.280**	**-0.411**	**4.197**	**1.976**
Prismatolaimus	Scutacarus	**-1.280**	**-0.608**	**4.197**	**3.055**
Rhabditidae	Tripyla	**-0.692**	**-0.420**	**5.475**	**4.595**
Rhabditidae	Alliphis	**-0.692**	**0.053**	**5.475**	**2.277**

Rhabditidae	Cheiroseius	**-0.692**	**0.356**	**5.475**	**1.976**
Rhabditidae	Dendrolaelaps	**-0.692**	**0.027**	**5.475**	**3.231**
Rhabditidae	Lysigamasus	**-0.692**	**0.407**	**5.475**	**1.976**
Rhabditidae	Macrocheles	**-0.692**	**0.761**	**5.475**	**1.976**
Rhabditidae	Pergamasus	**-0.692**	**1.081**	**5.475**	**1.976**
Rhabditidae	Uropoda	**-0.692**	**0.481**	**5.475**	**2.578**
Rhabditidae	Aporcelaimellus	**-0.692**	**0.548**	**5.475**	**3.896**
Rhabditidae	Dorylaimoidea	**-0.692**	**-0.604**	**5.475**	**4.975**
Rhabditidae	Eudorylaimus	**-0.692**	**-0.166**	**5.475**	**3.896**
Rhabditidae	Mesodorylaimus	**-0.692**	**-0.277**	**5.475**	**4.595**
Rhabditidae	Eupodes	**-0.692**	**0.005**	**5.475**	**2.879**
Rhabditidae	Mesostigmata	**-0.692**	**-0.411**	**5.475**	**1.976**
Rhabditidae	Scutacarus	**-0.692**	**-0.608**	**5.475**	**3.055**
Enchytraeus	Cheiroseius	**1.043**	**0.356**	**4.437**	**1.976**
Enchytraeus	Dendrolaelaps	**1.043**	**0.027**	**4.437**	**3.231**
Enchytraeus	Lysigamasus	**1.043**	**0.407**	**4.437**	**1.976**
Enchytraeus	Macrocheles	**1.043**	**0.761**	**4.437**	**1.976**
Enchytraeus	Pergamasus	**1.043**	**1.081**	**4.437**	**1.976**
Enchytraeus	Uropoda	**1.043**	**0.481**	**4.437**	**2.578**
Enchytraeus	Aporcelaimellus	**1.043**	**0.548**	**4.437**	**3.896**
Enchytraeus	Dorylaimoidea	**1.043**	**-0.604**	**4.437**	**4.975**
Enchytraeus	Eudorylaimus	**1.043**	**-0.166**	**4.437**	**3.896**
Enchytraeus	Mesodorylaimus	**1.043**	**-0.277**	**4.437**	**4.595**
Enchytraeus	Eupodes	**1.043**	**0.005**	**4.437**	**2.879**
Enchytraeus	Mesostigmata	**1.043**	**-0.411**	**4.437**	**1.976**
Enchytraeus	Scutacarus	**1.043**	**-0.608**	**4.437**	**3.055**
Eubacteria	Acrobeloides	**-6.738**	**-1.171**	**13.680**	**3.896**
Eubacteria	Cephalobidae	**-6.738**	**-1.055**	**13.680**	**4.498**
Eubacteria	Eucephalobus	**-6.738**	**-0.855**	**13.680**	**4.850**
Eubacteria	Metateratocephalus	**-6.738**	**-1.506**	**13.680**	**4.373**
Eubacteria	Plectus	**-6.738**	**-0.583**	**13.680**	**4.799**
Eubacteria	Prismatolaimus	**-6.738**	**-1.280**	**13.680**	**4.197**
Eubacteria	Rhabditidae	**-6.738**	**-0.692**	**13.680**	**5.475**
Eubacteria	Enchytraeus	**-6.738**	**1.043**	**13.680**	**4.437**
Eubacteria	Dauerlarvae	**-6.738**	**-0.804**	**13.680**	**4.741**
Eubacteria	Henlea	**-6.738**	**1.522**	**13.680**	**4.316**
Dauerlarvae	Tripyla	**-0.804**	**-0.420**	**4.741**	**4.595**
Dauerlarvae	Alliphis	**-0.804**	**0.053**	**4.741**	**2.277**
Dauerlarvae	Aporcelaimellus	**-0.804**	**0.548**	**4.741**	**3.896**
Dauerlarvae	Dorylaimoidea	**-0.804**	**-0.604**	**4.741**	**4.975**
Dauerlarvae	Eudorylaimus	**-0.804**	**-0.166**	**4.741**	**3.896**
Dauerlarvae	Mesodorylaimus	**-0.804**	**-0.277**	**4.741**	**4.595**
Dauerlarvae	Eupodes	**-0.804**	**0.005**	**4.741**	**2.879**
Dauerlarvae	Mesostigmata	**-0.804**	**-0.411**	**4.741**	**1.976**
Dauerlarvae	Scutacarus	**-0.804**	**-0.608**	**4.741**	**3.055**
Henlea	Cheiroseius	**1.522**	**0.356**	**4.316**	**1.976**
Henlea	Dendrolaelaps	**1.522**	**0.027**	**4.316**	**3.231**
Henlea	Lysigamasus	**1.522**	**0.407**	**4.316**	**1.976**
Henlea	Macrocheles	**1.522**	**0.761**	**4.316**	**1.976**
Henlea	Pergamasus	**1.522**	**1.081**	**4.316**	**1.976**
Henlea	Uropoda	**1.522**	**0.481**	**4.316**	**2.578**

Henlea	Aporcelaimellus	**1.522**	**0.548**	**4.316**	**3.896**
Henlea	Dorylaimoidea	**1.522**	**-0.604**	**4.316**	**4.975**
Henlea	Eudorylaimus	**1.522**	**-0.166**	**4.316**	**3.896**
Henlea	Mesodorylaimus	**1.522**	**-0.277**	**4.316**	**4.595**
Henlea	Eupodes	**1.522**	**0.005**	**4.316**	**2.879**
Henlea	Mesostigmata	**1.522**	**-0.411**	**4.316**	**1.976**
Henlea	Scutacarus	**1.522**	**-0.608**	**4.316**	**3.055**
Hyphae and hair roots	Dolichodoridae	**6.491**	**-0.885**	**0.000**	**4.373**
Hyphae and hair roots	Helicotylenchus	**6.491**	**-0.792**	**0.000**	**4.896**
Hyphae and hair roots	Heterodera	**6.491**	**-0.883**	**0.000**	**4.197**
Hyphae and hair roots	Malenchus	**6.491**	**-1.330**	**0.000**	**5.072**
Hyphae and hair roots	Paratylenchus	**6.491**	**-1.244**	**0.000**	**4.197**
Hyphae and hair roots	Tylenchorhynchus	**6.491**	**-0.664**	**0.000**	**4.197**
Hyphae and hair roots	Tydeidae	**6.491**	**-0.608**	**0.000**	**1.976**
Hyphae and hair roots	Sminthuridae	**6.491**	**-0.608**	**0.000**	**2.578**
Hyphae and hair roots	Sminthurinus	**6.491**	**0.618**	**0.000**	**3.556**
Hyphae and hair roots	Sminthurus	**6.491**	**1.429**	**0.000**	**2.277**
Hyphae and hair roots	Sphaeridia	**6.491**	**0.202**	**0.000**	**2.821**
Hyphae and hair roots	Aphelenchoides	**6.491**	**-1.496**	**0.000**	**4.595**
Hyphae and hair roots	Tylenchidae	**6.491**	**-1.360**	**0.000**	**5.197**
Hyphae and hair roots	Pygmephorus	**6.491**	**-0.376**	**0.000**	**2.821**
Hyphae and hair roots	Tyrophagus	**6.491**	**0.005**	**0.000**	**1.976**
Hyphae and hair roots	Friesea	**6.491**	**0.434**	**0.000**	**2.578**
Hyphae and hair roots	Isotoma	**6.491**	**1.898**	**0.000**	**3.684**
Hyphae and hair roots	Isotomiella	**6.491**	**0.816**	**0.000**	**2.277**
Hyphae and hair roots	Isotomurus	**6.491**	**1.787**	**0.000**	**3.277**
Hyphae and hair roots	Lepidocyrtus	**6.491**	**1.231**	**0.000**	**2.675**
Hyphae and hair roots	Proisotoma	**6.491**	**0.770**	**0.000**	**2.754**
Hyphae and hair roots	Achaeta	**6.491**	**1.300**	**0.000**	**3.460**
Hyphae and hair roots	Fridericia	**6.491**	**1.985**	**0.000**	**4.023**
Hyphae and hair roots	Aporcelaimellus	**6.491**	**0.548**	**0.000**	**3.896**
Hyphae and hair roots	Dorylaimoidea	**6.491**	**-0.604**	**0.000**	**4.975**
Hyphae and hair roots	Eudorylaimus	**6.491**	**-0.166**	**0.000**	**3.896**
Hyphae and hair roots	Mesodorylaimus	**6.491**	**-0.277**	**0.000**	**4.595**
Hyphae and hair roots	Eupodes	**6.491**	**0.005**	**0.000**	**2.879**
Hyphae and hair roots	Mesostigmata	**6.491**	**-0.411**	**0.000**	**1.976**
Hyphae and hair roots	Scutacarus	**6.491**	**-0.608**	**0.000**	**3.055**
Tripyla	Cheiroseius	**-0.420**	**0.356**	**4.595**	**1.976**
Tripyla	Dendrolaelaps	**-0.420**	**0.027**	**4.595**	**3.231**
Tripyla	Lysigamasus	**-0.420**	**0.407**	**4.595**	**1.976**
Tripyla	Macrocheles	**-0.420**	**0.761**	**4.595**	**1.976**
Tripyla	Pergamasus	**-0.420**	**1.081**	**4.595**	**1.976**
Tripyla	Uropoda	**-0.420**	**0.481**	**4.595**	**2.578**
Tripyla	Aporcelaimellus	**-0.420**	**0.548**	**4.595**	**3.896**
Tripyla	Dorylaimoidea	**-0.420**	**-0.604**	**4.595**	**4.975**
Tripyla	Eudorylaimus	**-0.420**	**-0.166**	**4.595**	**3.896**
Tripyla	Mesodorylaimus	**-0.420**	**-0.277**	**4.595**	**4.595**
Tripyla	Eupodes	**-0.420**	**0.005**	**4.595**	**2.879**
Tripyla	Mesostigmata	**-0.420**	**-0.411**	**4.595**	**1.976**
Tripyla	Scutacarus	**-0.420**	**-0.608**	**4.595**	**3.055**
Alliphis	Cheiroseius	**0.053**	**0.356**	**2.277**	**1.976**

Alliphis	Dendrolaelaps	**0.053**	**0.027**	**2.277**	**3.231**
Alliphis	Lysigamasus	**0.053**	**0.407**	**2.277**	**1.976**
Alliphis	Macrocheles	**0.053**	**0.761**	**2.277**	**1.976**
Alliphis	Pergamasus	**0.053**	**1.081**	**2.277**	**1.976**
Alliphis	Uropoda	**0.053**	**0.481**	**2.277**	**2.578**
Alliphis	Aporcelaimellus	**0.053**	**0.548**	**2.277**	**3.896**
Alliphis	Dorylaimoidea	**0.053**	**-0.604**	**2.277**	**4.975**
Alliphis	Eudorylaimus	**0.053**	**-0.166**	**2.277**	**3.896**
Alliphis	Mesodorylaimus	**0.053**	**-0.277**	**2.277**	**4.595**
Alliphis	Eupodes	**0.053**	**0.005**	**2.277**	**2.879**
Alliphis	Mesostigmata	**0.053**	**-0.411**	**2.277**	**1.976**
Alliphis	Scutacarus	**0.053**	**-0.608**	**2.277**	**3.055**
Cheiroseius	Aporcelaimellus	**0.356**	**0.548**	**1.976**	**3.896**
Cheiroseius	Dorylaimoidea	**0.356**	**-0.604**	**1.976**	**4.975**
Cheiroseius	Eudorylaimus	**0.356**	**-0.166**	**1.976**	**3.896**
Cheiroseius	Mesodorylaimus	**0.356**	**-0.277**	**1.976**	**4.595**
Cheiroseius	Eupodes	**0.356**	**0.005**	**1.976**	**2.879**
Cheiroseius	Mesostigmata	**0.356**	**-0.411**	**1.976**	**1.976**
Cheiroseius	Scutacarus	**0.356**	**-0.608**	**1.976**	**3.055**
Dendrolaelaps	Aporcelaimellus	**0.027**	**0.548**	**3.231**	**3.896**
Dendrolaelaps	Dorylaimoidea	**0.027**	**-0.604**	**3.231**	**4.975**
Dendrolaelaps	Eudorylaimus	**0.027**	**-0.166**	**3.231**	**3.896**
Dendrolaelaps	Mesodorylaimus	**0.027**	**-0.277**	**3.231**	**4.595**
Dendrolaelaps	Eupodes	**0.027**	**0.005**	**3.231**	**2.879**
Dendrolaelaps	Mesostigmata	**0.027**	**-0.411**	**3.231**	**1.976**
Dendrolaelaps	Scutacarus	**0.027**	**-0.608**	**3.231**	**3.055**
Lysigamasus	Aporcelaimellus	**0.407**	**0.548**	**1.976**	**3.896**
Lysigamasus	Dorylaimoidea	**0.407**	**-0.604**	**1.976**	**4.975**
Lysigamasus	Eudorylaimus	**0.407**	**-0.166**	**1.976**	**3.896**
Lysigamasus	Mesodorylaimus	**0.407**	**-0.277**	**1.976**	**4.595**
Lysigamasus	Eupodes	**0.407**	**0.005**	**1.976**	**2.879**
Lysigamasus	Mesostigmata	**0.407**	**-0.411**	**1.976**	**1.976**
Lysigamasus	Scutacarus	**0.407**	**-0.608**	**1.976**	**3.055**
Macrocheles	Aporcelaimellus	**0.761**	**0.548**	**1.976**	**3.896**
Macrocheles	Dorylaimoidea	**0.761**	**-0.604**	**1.976**	**4.975**
Macrocheles	Eudorylaimus	**0.761**	**-0.166**	**1.976**	**3.896**
Macrocheles	Mesodorylaimus	**0.761**	**-0.277**	**1.976**	**4.595**
Macrocheles	Eupodes	**0.761**	**0.005**	**1.976**	**2.879**
Macrocheles	Mesostigmata	**0.761**	**-0.411**	**1.976**	**1.976**
Macrocheles	Scutacarus	**0.761**	**-0.608**	**1.976**	**3.055**
Pergamasus	Aporcelaimellus	**1.081**	**0.548**	**1.976**	**3.896**
Pergamasus	Dorylaimoidea	**1.081**	**-0.604**	**1.976**	**4.975**
Pergamasus	Eudorylaimus	**1.081**	**-0.166**	**1.976**	**3.896**
Pergamasus	Mesodorylaimus	**1.081**	**-0.277**	**1.976**	**4.595**
Pergamasus	Eupodes	**1.081**	**0.005**	**1.976**	**2.879**
Pergamasus	Mesostigmata	**1.081**	**-0.411**	**1.976**	**1.976**
Pergamasus	Scutacarus	**1.081**	**-0.608**	**1.976**	**3.055**
Uropoda	Aporcelaimellus	**0.481**	**0.548**	**2.578**	**3.896**
Uropoda	Dorylaimoidea	**0.481**	**-0.604**	**2.578**	**4.975**
Uropoda	Eudorylaimus	**0.481**	**-0.166**	**2.578**	**3.896**
Uropoda	Mesodorylaimus	**0.481**	**-0.277**	**2.578**	**4.595**

Uropoda	Eupodes	**0.481**	**0.005**	**2.578**	**2.879**
Uropoda	Mesostigmata	**0.481**	**-0.411**	**2.578**	**1.976**
Uropoda	Scutacarus	**0.481**	**-0.608**	**2.578**	**3.055**
Aporcelaimellus	Tripyla	**0.548**	**-0.420**	**3.896**	**4.595**
Aporcelaimellus	Alliphis	**0.548**	**0.053**	**3.896**	**2.277**
Aporcelaimellus	Cheiroseius	**0.548**	**0.356**	**3.896**	**1.976**
Aporcelaimellus	Dendrolaelaps	**0.548**	**0.027**	**3.896**	**3.231**
Aporcelaimellus	Lysigamasus	**0.548**	**0.407**	**3.896**	**1.976**
Aporcelaimellus	Macrocheles	**0.548**	**0.761**	**3.896**	**1.976**
Aporcelaimellus	Pergamasus	**0.548**	**1.081**	**3.896**	**1.976**
Aporcelaimellus	Uropoda	**0.548**	**0.481**	**3.896**	**2.578**
Aporcelaimellus	Aporcelaimellus	**0.548**	**0.548**	**3.896**	**3.896**
Aporcelaimellus	Dorylaimoidea	**0.548**	**-0.604**	**3.896**	**4.975**
Aporcelaimellus	Eudorylaimus	**0.548**	**-0.166**	**3.896**	**3.896**
Aporcelaimellus	Mesodorylaimus	**0.548**	**-0.277**	**3.896**	**4.595**
Aporcelaimellus	Eupodes	**0.548**	**0.005**	**3.896**	**2.879**
Aporcelaimellus	Mesostigmata	**0.548**	**-0.411**	**3.896**	**1.976**
Aporcelaimellus	Scutacarus	**0.548**	**-0.608**	**3.896**	**3.055**
Dorylaimoidea	Tripyla	**-0.604**	**-0.420**	**4.975**	**4.595**
Dorylaimoidea	Alliphis	**-0.604**	**0.053**	**4.975**	**2.277**
Dorylaimoidea	Cheiroseius	**-0.604**	**0.356**	**4.975**	**1.976**
Dorylaimoidea	Dendrolaelaps	**-0.604**	**0.027**	**4.975**	**3.231**
Dorylaimoidea	Lysigamasus	**-0.604**	**0.407**	**4.975**	**1.976**
Dorylaimoidea	Macrocheles	**-0.604**	**0.761**	**4.975**	**1.976**
Dorylaimoidea	Pergamasus	**-0.604**	**1.081**	**4.975**	**1.976**
Dorylaimoidea	Uropoda	**-0.604**	**0.481**	**4.975**	**2.578**
Dorylaimoidea	Aporcelaimellus	**-0.604**	**0.548**	**4.975**	**3.896**
Dorylaimoidea	Dorylaimoidea	**-0.604**	**-0.604**	**4.975**	**4.975**
Dorylaimoidea	Eudorylaimus	**-0.604**	**-0.166**	**4.975**	**3.896**
Dorylaimoidea	Mesodorylaimus	**-0.604**	**-0.277**	**4.975**	**4.595**
Dorylaimoidea	Eupodes	**-0.604**	**0.005**	**4.975**	**2.879**
Dorylaimoidea	Mesostigmata	**-0.604**	**-0.411**	**4.975**	**1.976**
Dorylaimoidea	Scutacarus	**-0.604**	**-0.608**	**4.975**	**3.055**
Eudorylaimus	Tripyla	**-0.166**	**-0.420**	**3.896**	**4.595**
Eudorylaimus	Alliphis	**-0.166**	**0.053**	**3.896**	**2.277**
Eudorylaimus	Cheiroseius	**-0.166**	**0.356**	**3.896**	**1.976**
Eudorylaimus	Dendrolaelaps	**-0.166**	**0.027**	**3.896**	**3.231**
Eudorylaimus	Lysigamasus	**-0.166**	**0.407**	**3.896**	**1.976**
Eudorylaimus	Macrocheles	**-0.166**	**0.761**	**3.896**	**1.976**
Eudorylaimus	Pergamasus	**-0.166**	**1.081**	**3.896**	**1.976**
Eudorylaimus	Uropoda	**-0.166**	**0.481**	**3.896**	**2.578**
Eudorylaimus	Aporcelaimellus	**-0.166**	**0.548**	**3.896**	**3.896**
Eudorylaimus	Dorylaimoidea	**-0.166**	**-0.604**	**3.896**	**4.975**
Eudorylaimus	Eudorylaimus	**-0.166**	**-0.166**	**3.896**	**3.896**
Eudorylaimus	Mesodorylaimus	**-0.166**	**-0.277**	**3.896**	**4.595**
Eudorylaimus	Eupodes	**-0.166**	**0.005**	**3.896**	**2.879**
Eudorylaimus	Mesostigmata	**-0.166**	**-0.411**	**3.896**	**1.976**
Eudorylaimus	Scutacarus	**-0.166**	**-0.608**	**3.896**	**3.055**
Mesodorylaimus	Tripyla	**-0.277**	**-0.420**	**4.595**	**4.595**
Mesodorylaimus	Alliphis	**-0.277**	**0.053**	**4.595**	**2.277**
Mesodorylaimus	Cheiroseius	**-0.277**	**0.356**	**4.595**	**1.976**

Mesodorylaimus	Dendrolaelaps	**-0.277**	**0.027**	**4.595**	**3.231**
Mesodorylaimus	Lysigamasus	**-0.277**	**0.407**	**4.595**	**1.976**
Mesodorylaimus	Macrocheles	**-0.277**	**0.761**	**4.595**	**1.976**
Mesodorylaimus	Pergamasus	**-0.277**	**1.081**	**4.595**	**1.976**
Mesodorylaimus	Uropoda	**-0.277**	**0.481**	**4.595**	**2.578**
Mesodorylaimus	Aporcelaimellus	**-0.277**	**0.548**	**4.595**	**3.896**
Mesodorylaimus	Dorylaimoidea	**-0.277**	**-0.604**	**4.595**	**4.975**
Mesodorylaimus	Eudorylaimus	**-0.277**	**-0.166**	**4.595**	**3.896**
Mesodorylaimus	Mesodorylaimus	**-0.277**	**-0.277**	**4.595**	**4.595**
Mesodorylaimus	Eupodes	**-0.277**	**0.005**	**4.595**	**2.879**
Mesodorylaimus	Mesostigmata	**-0.277**	**-0.411**	**4.595**	**1.976**
Mesodorylaimus	Scutacarus	**-0.277**	**-0.608**	**4.595**	**3.055**
Eupodes	Cheiroseius	**0.005**	**0.356**	**2.879**	**1.976**
Eupodes	Dendrolaelaps	**0.005**	**0.027**	**2.879**	**3.231**
Eupodes	Lysigamasus	**0.005**	**0.407**	**2.879**	**1.976**
Eupodes	Macrocheles	**0.005**	**0.761**	**2.879**	**1.976**
Eupodes	Pergamasus	**0.005**	**1.081**	**2.879**	**1.976**
Eupodes	Uropoda	**0.005**	**0.481**	**2.879**	**2.578**
Eupodes	Aporcelaimellus	**0.005**	**0.548**	**2.879**	**3.896**
Eupodes	Dorylaimoidea	**0.005**	**-0.604**	**2.879**	**4.975**
Eupodes	Eudorylaimus	**0.005**	**-0.166**	**2.879**	**3.896**
Eupodes	Mesodorylaimus	**0.005**	**-0.277**	**2.879**	**4.595**
Eupodes	Eupodes	**0.005**	**0.005**	**2.879**	**2.879**
Eupodes	Mesostigmata	**0.005**	**-0.411**	**2.879**	**1.976**
Eupodes	Scutacarus	**0.005**	**-0.608**	**2.879**	**3.055**
Mesostigmata	Cheiroseius	**-0.411**	**0.356**	**1.976**	**1.976**
Mesostigmata	Dendrolaelaps	**-0.411**	**0.027**	**1.976**	**3.231**
Mesostigmata	Lysigamasus	**-0.411**	**0.407**	**1.976**	**1.976**
Mesostigmata	Macrocheles	**-0.411**	**0.761**	**1.976**	**1.976**
Mesostigmata	Pergamasus	**-0.411**	**1.081**	**1.976**	**1.976**
Mesostigmata	Uropoda	**-0.411**	**0.481**	**1.976**	**2.578**
Mesostigmata	Aporcelaimellus	**-0.411**	**0.548**	**1.976**	**3.896**
Mesostigmata	Dorylaimoidea	**-0.411**	**-0.604**	**1.976**	**4.975**
Mesostigmata	Eudorylaimus	**-0.411**	**-0.166**	**1.976**	**3.896**
Mesostigmata	Mesodorylaimus	**-0.411**	**-0.277**	**1.976**	**4.595**
Mesostigmata	Eupodes	**-0.411**	**0.005**	**1.976**	**2.879**
Mesostigmata	Mesostigmata	**-0.411**	**-0.411**	**1.976**	**1.976**
Mesostigmata	Scutacarus	**-0.411**	**-0.608**	**1.976**	**3.055**
Scutacarus	Cheiroseius	**-0.608**	**0.356**	**3.055**	**1.976**
Scutacarus	Dendrolaelaps	**-0.608**	**0.027**	**3.055**	**3.231**
Scutacarus	Lysigamasus	**-0.608**	**0.407**	**3.055**	**1.976**
Scutacarus	Macrocheles	**-0.608**	**0.761**	**3.055**	**1.976**
Scutacarus	Pergamasus	**-0.608**	**1.081**	**3.055**	**1.976**
Scutacarus	Uropoda	**-0.608**	**0.481**	**3.055**	**2.578**
Scutacarus	Aporcelaimellus	**-0.608**	**0.548**	**3.055**	**3.896**
Scutacarus	Dorylaimoidea	**-0.608**	**-0.604**	**3.055**	**4.975**
Scutacarus	Eudorylaimus	**-0.608**	**-0.166**	**3.055**	**3.896**
Scutacarus	Mesodorylaimus	**-0.608**	**-0.277**	**3.055**	**4.595**
Scutacarus	Eupodes	**-0.608**	**0.005**	**3.055**	**2.879**
Scutacarus	Mesostigmata	**-0.608**	**-0.411**	**3.055**	**1.976**
Scutacarus	Scutacarus	**-0.608**	**-0.608**	**3.055**	**3.055**

Resource	Consumer	Mres	Mconsumer	Nres	Nconsumer
Dolichodoridae	Mylonchulus	**-0.885**	**-0.005**	**5.173**	**3.918**
Dolichodoridae	Alliphis	**-0.885**	**0.053**	**5.173**	**3.438**
Dolichodoridae	Arctoseius	**-0.885**	**-0.152**	**5.173**	**2.821**
Dolichodoridae	Dendrolaelaps	**-0.885**	**0.027**	**5.173**	**1.976**
Dolichodoridae	Hypoaspis	**-0.885**	**0.334**	**5.173**	**2.754**
Dolichodoridae	Lysigamasus	**-0.885**	**0.407**	**5.173**	**3.318**
Dolichodoridae	Macrocheles	**-0.885**	**0.761**	**5.173**	**2.277**
Dolichodoridae	Pergamasus	**-0.885**	**1.081**	**5.173**	**1.976**
Dolichodoridae	Aporcelaimellus	**-0.885**	**0.548**	**5.173**	**4.219**
Dolichodoridae	Dorylaimoidea	**-0.885**	**-0.604**	**5.173**	**4.763**
Dolichodoridae	Qudsianematidae	**-0.885**	**-0.207**	**5.173**	**3.918**
Dolichodoridae	Eupodes	**-0.885**	**0.005**	**5.173**	**2.277**
Dolichodoridae	Mesostigmata	**-0.885**	**-0.411**	**5.173**	**2.453**
Dolichodoridae	Scutacarus	**-0.885**	**-0.608**	**5.173**	**3.481**
Dolichodoridae	Stigmaeidae	**-0.885**	**0.229**	**5.173**	**1.976**
Helicotylenchus	Mylonchulus	**-0.792**	**-0.005**	**4.696**	**3.918**
Helicotylenchus	Alliphis	**-0.792**	**0.053**	**4.696**	**3.438**
Helicotylenchus	Arctoseius	**-0.792**	**-0.152**	**4.696**	**2.821**
Helicotylenchus	Dendrolaelaps	**-0.792**	**0.027**	**4.696**	**1.976**
Helicotylenchus	Hypoaspis	**-0.792**	**0.334**	**4.696**	**2.754**
Helicotylenchus	Lysigamasus	**-0.792**	**0.407**	**4.696**	**3.318**
Helicotylenchus	Macrocheles	**-0.792**	**0.761**	**4.696**	**2.277**
Helicotylenchus	Pergamasus	**-0.792**	**1.081**	**4.696**	**1.976**
Helicotylenchus	Aporcelaimellus	**-0.792**	**0.548**	**4.696**	**4.219**
Helicotylenchus	Dorylaimoidea	**-0.792**	**-0.604**	**4.696**	**4.763**
Helicotylenchus	Qudsianematidae	**-0.792**	**-0.207**	**4.696**	**3.918**
Helicotylenchus	Eupodes	**-0.792**	**0.005**	**4.696**	**2.277**
Helicotylenchus	Mesostigmata	**-0.792**	**-0.411**	**4.696**	**2.453**
Helicotylenchus	Scutacarus	**-0.792**	**-0.608**	**4.696**	**3.481**
Helicotylenchus	Stigmaeidae	**-0.792**	**0.229**	**4.696**	**1.976**
Malenchus	Mylonchulus	**-1.330**	**-0.005**	**4.219**	**3.918**
Malenchus	Alliphis	**-1.330**	**0.053**	**4.219**	**3.438**
Malenchus	Arctoseius	**-1.330**	**-0.152**	**4.219**	**2.821**
Malenchus	Dendrolaelaps	**-1.330**	**0.027**	**4.219**	**1.976**
Malenchus	Hypoaspis	**-1.330**	**0.334**	**4.219**	**2.754**
Malenchus	Lysigamasus	**-1.330**	**0.407**	**4.219**	**3.318**
Malenchus	Macrocheles	**-1.330**	**0.761**	**4.219**	**2.277**
Malenchus	Pergamasus	**-1.330**	**1.081**	**4.219**	**1.976**
Malenchus	Aporcelaimellus	**-1.330**	**0.548**	**4.219**	**4.219**
Malenchus	Dorylaimoidea	**-1.330**	**-0.604**	**4.219**	**4.763**
Malenchus	Qudsianematidae	**-1.330**	**-0.207**	**4.219**	**3.918**
Malenchus	Eupodes	**-1.330**	**0.005**	**4.219**	**2.277**
Malenchus	Mesostigmata	**-1.330**	**-0.411**	**4.219**	**2.453**
Malenchus	Scutacarus	**-1.330**	**-0.608**	**4.219**	**3.481**
Malenchus	Stigmaeidae	**-1.330**	**0.229**	**4.219**	**1.976**
Meloidogyne	Mylonchulus	**-1.287**	**-0.005**	**3.918**	**3.918**
Meloidogyne	Alliphis	**-1.287**	**0.053**	**3.918**	**3.438**
Meloidogyne	Arctoseius	**-1.287**	**-0.152**	**3.918**	**2.821**
Meloidogyne	Dendrolaelaps	**-1.287**	**0.027**	**3.918**	**1.976**
Meloidogyne	Hypoaspis	**-1.287**	**0.334**	**3.918**	**2.754**

Meloidogyne	Lysigamasus	**-1.287**	**0.407**	**3.918**	**3.318**
Meloidogyne	Macrocheles	**-1.287**	**0.761**	**3.918**	**2.277**
Meloidogyne	Pergamasus	**-1.287**	**1.081**	**3.918**	**1.976**
Meloidogyne	Aporcelaimellus	**-1.287**	**0.548**	**3.918**	**4.219**
Meloidogyne	Dorylaimoidea	**-1.287**	**-0.604**	**3.918**	**4.763**
Meloidogyne	Qudsianematidae	**-1.287**	**-0.207**	**3.918**	**3.918**
Meloidogyne	Eupodes	**-1.287**	**0.005**	**3.918**	**2.277**
Meloidogyne	Mesostigmata	**-1.287**	**-0.411**	**3.918**	**2.453**
Meloidogyne	Scutacarus	**-1.287**	**-0.608**	**3.918**	**3.481**
Meloidogyne	Stigmaeidae	**-1.287**	**0.229**	**3.918**	**1.976**
Paratylenchus	Mylonchulus	**-1.244**	**-0.005**	**4.395**	**3.918**
Paratylenchus	Alliphis	**-1.244**	**0.053**	**4.395**	**3.438**
Paratylenchus	Arctoseius	**-1.244**	**-0.152**	**4.395**	**2.821**
Paratylenchus	Dendrolaelaps	**-1.244**	**0.027**	**4.395**	**1.976**
Paratylenchus	Hypoaspis	**-1.244**	**0.334**	**4.395**	**2.754**
Paratylenchus	Lysigamasus	**-1.244**	**0.407**	**4.395**	**3.318**
Paratylenchus	Macrocheles	**-1.244**	**0.761**	**4.395**	**2.277**
Paratylenchus	Pergamasus	**-1.244**	**1.081**	**4.395**	**1.976**
Paratylenchus	Aporcelaimellus	**-1.244**	**0.548**	**4.395**	**4.219**
Paratylenchus	Dorylaimoidea	**-1.244**	**-0.604**	**4.395**	**4.763**
Paratylenchus	Qudsianematidae	**-1.244**	**-0.207**	**4.395**	**3.918**
Paratylenchus	Eupodes	**-1.244**	**0.005**	**4.395**	**2.277**
Paratylenchus	Mesostigmata	**-1.244**	**-0.411**	**4.395**	**2.453**
Paratylenchus	Scutacarus	**-1.244**	**-0.608**	**4.395**	**3.481**
Paratylenchus	Stigmaeidae	**-1.244**	**0.229**	**4.395**	**1.976**
Pratylenchus	Mylonchulus	**-1.226**	**-0.005**	**4.219**	**3.918**
Pratylenchus	Alliphis	**-1.226**	**0.053**	**4.219**	**3.438**
Pratylenchus	Arctoseius	**-1.226**	**-0.152**	**4.219**	**2.821**
Pratylenchus	Dendrolaelaps	**-1.226**	**0.027**	**4.219**	**1.976**
Pratylenchus	Hypoaspis	**-1.226**	**0.334**	**4.219**	**2.754**
Pratylenchus	Lysigamasus	**-1.226**	**0.407**	**4.219**	**3.318**
Pratylenchus	Macrocheles	**-1.226**	**0.761**	**4.219**	**2.277**
Pratylenchus	Pergamasus	**-1.226**	**1.081**	**4.219**	**1.976**
Pratylenchus	Aporcelaimellus	**-1.226**	**0.548**	**4.219**	**4.219**
Pratylenchus	Dorylaimoidea	**-1.226**	**-0.604**	**4.219**	**4.763**
Pratylenchus	Qudsianematidae	**-1.226**	**-0.207**	**4.219**	**3.918**
Pratylenchus	Eupodes	**-1.226**	**0.005**	**4.219**	**2.277**
Pratylenchus	Mesostigmata	**-1.226**	**-0.411**	**4.219**	**2.453**
Pratylenchus	Scutacarus	**-1.226**	**-0.608**	**4.219**	**3.481**
Pratylenchus	Stigmaeidae	**-1.226**	**0.229**	**4.219**	**1.976**
Trichodorus	Mylonchulus	**-0.744**	**-0.005**	**4.219**	**3.918**
Trichodorus	Alliphis	**-0.744**	**0.053**	**4.219**	**3.438**
Trichodorus	Arctoseius	**-0.744**	**-0.152**	**4.219**	**2.821**
Trichodorus	Dendrolaelaps	**-0.744**	**0.027**	**4.219**	**1.976**
Trichodorus	Hypoaspis	**-0.744**	**0.334**	**4.219**	**2.754**
Trichodorus	Lysigamasus	**-0.744**	**0.407**	**4.219**	**3.318**
Trichodorus	Macrocheles	**-0.744**	**0.761**	**4.219**	**2.277**
Trichodorus	Pergamasus	**-0.744**	**1.081**	**4.219**	**1.976**
Trichodorus	Aporcelaimellus	**-0.744**	**0.548**	**4.219**	**4.219**
Trichodorus	Dorylaimoidea	**-0.744**	**-0.604**	**4.219**	**4.763**
Trichodorus	Qudsianematidae	**-0.744**	**-0.207**	**4.219**	**3.918**

Trichodorus	Eupodes	**-0.744**	**0.005**	**4.219**	**2.277**
Trichodorus	Mesostigmata	**-0.744**	**-0.411**	**4.219**	**2.453**
Trichodorus	Scutacarus	**-0.744**	**-0.608**	**4.219**	**3.481**
Trichodorus	Stigmaeidae	**-0.744**	**0.229**	**4.219**	**1.976**
Tylenchorhynchus	Mylonchulus	**-0.664**	**-0.005**	**3.918**	**3.918**
Tylenchorhynchus	Alliphis	**-0.664**	**0.053**	**3.918**	**3.438**
Tylenchorhynchus	Arctoseius	**-0.664**	**-0.152**	**3.918**	**2.821**
Tylenchorhynchus	Dendrolaelaps	**-0.664**	**0.027**	**3.918**	**1.976**
Tylenchorhynchus	Hypoaspis	**-0.664**	**0.334**	**3.918**	**2.754**
Tylenchorhynchus	Lysigamasus	**-0.664**	**0.407**	**3.918**	**3.318**
Tylenchorhynchus	Macrocheles	**-0.664**	**0.761**	**3.918**	**2.277**
Tylenchorhynchus	Pergamasus	**-0.664**	**1.081**	**3.918**	**1.976**
Tylenchorhynchus	Aporcelaimellus	**-0.664**	**0.548**	**3.918**	**4.219**
Tylenchorhynchus	Dorylaimoidea	**-0.664**	**-0.604**	**3.918**	**4.763**
Tylenchorhynchus	Qudsianematidae	**-0.664**	**-0.207**	**3.918**	**3.918**
Tylenchorhynchus	Eupodes	**-0.664**	**0.005**	**3.918**	**2.277**
Tylenchorhynchus	Mesostigmata	**-0.664**	**-0.411**	**3.918**	**2.453**
Tylenchorhynchus	Scutacarus	**-0.664**	**-0.608**	**3.918**	**3.481**
Tylenchorhynchus	Stigmaeidae	**-0.664**	**0.229**	**3.918**	**1.976**
Pachygnatidae	Arctoseius	**-0.113**	**-0.152**	**2.578**	**2.821**
Pachygnatidae	Dendrolaelaps	**-0.113**	**0.027**	**2.578**	**1.976**
Pachygnatidae	Hypoaspis	**-0.113**	**0.334**	**2.578**	**2.754**
Pachygnatidae	Lysigamasus	**-0.113**	**0.407**	**2.578**	**3.318**
Pachygnatidae	Macrocheles	**-0.113**	**0.761**	**2.578**	**2.277**
Pachygnatidae	Pergamasus	**-0.113**	**1.081**	**2.578**	**1.976**
Pachygnatidae	Aporcelaimellus	**-0.113**	**0.548**	**2.578**	**4.219**
Pachygnatidae	Dorylaimoidea	**-0.113**	**-0.604**	**2.578**	**4.763**
Pachygnatidae	Qudsianematidae	**-0.113**	**-0.207**	**2.578**	**3.918**
Pachygnatidae	Eupodes	**-0.113**	**0.005**	**2.578**	**2.277**
Pachygnatidae	Mesostigmata	**-0.113**	**-0.411**	**2.578**	**2.453**
Pachygnatidae	Scutacarus	**-0.113**	**-0.608**	**2.578**	**3.481**
Pachygnatidae	Stigmaeidae	**-0.113**	**0.229**	**2.578**	**1.976**
Tydeidae	Arctoseius	**-0.608**	**-0.152**	**2.879**	**2.821**
Tydeidae	Dendrolaelaps	**-0.608**	**0.027**	**2.879**	**1.976**
Tydeidae	Hypoaspis	**-0.608**	**0.334**	**2.879**	**2.754**
Tydeidae	Lysigamasus	**-0.608**	**0.407**	**2.879**	**3.318**
Tydeidae	Macrocheles	**-0.608**	**0.761**	**2.879**	**2.277**
Tydeidae	Pergamasus	**-0.608**	**1.081**	**2.879**	**1.976**
Tydeidae	Aporcelaimellus	**-0.608**	**0.548**	**2.879**	**4.219**
Tydeidae	Dorylaimoidea	**-0.608**	**-0.604**	**2.879**	**4.763**
Tydeidae	Qudsianematidae	**-0.608**	**-0.207**	**2.879**	**3.918**
Tydeidae	Eupodes	**-0.608**	**0.005**	**2.879**	**2.277**
Tydeidae	Mesostigmata	**-0.608**	**-0.411**	**2.879**	**2.453**
Tydeidae	Scutacarus	**-0.608**	**-0.608**	**2.879**	**3.481**
Tydeidae	Stigmaeidae	**-0.608**	**0.229**	**2.879**	**1.976**
Sminthuridae	Arctoseius	**-0.608**	**-0.152**	**2.578**	**2.821**
Sminthuridae	Dendrolaelaps	**-0.608**	**0.027**	**2.578**	**1.976**
Sminthuridae	Hypoaspis	**-0.608**	**0.334**	**2.578**	**2.754**
Sminthuridae	Lysigamasus	**-0.608**	**0.407**	**2.578**	**3.318**
Sminthuridae	Macrocheles	**-0.608**	**0.761**	**2.578**	**2.277**
Sminthuridae	Pergamasus	**-0.608**	**1.081**	**2.578**	**1.976**

Sminthuridae	Aporcelaimellus	**-0.608**	**0.548**	**2.578**	**4.219**
Sminthuridae	Dorylaimoidea	**-0.608**	**-0.604**	**2.578**	**4.763**
Sminthuridae	Qudsianematidae	**-0.608**	**-0.207**	**2.578**	**3.918**
Sminthuridae	Eupodes	**-0.608**	**0.005**	**2.578**	**2.277**
Sminthuridae	Mesostigmata	**-0.608**	**-0.411**	**2.578**	**2.453**
Sminthuridae	Scutacarus	**-0.608**	**-0.608**	**2.578**	**3.481**
Sminthuridae	Stigmaeidae	**-0.608**	**0.229**	**2.578**	**1.976**
Sminthurinus	Arctoseius	**0.618**	**-0.152**	**3.152**	**2.821**
Sminthurinus	Dendrolaelaps	**0.618**	**0.027**	**3.152**	**1.976**
Sminthurinus	Hypoaspis	**0.618**	**0.334**	**3.152**	**2.754**
Sminthurinus	Lysigamasus	**0.618**	**0.407**	**3.152**	**3.318**
Sminthurinus	Macrocheles	**0.618**	**0.761**	**3.152**	**2.277**
Sminthurinus	Pergamasus	**0.618**	**1.081**	**3.152**	**1.976**
Sminthurinus	Aporcelaimellus	**0.618**	**0.548**	**3.152**	**4.219**
Sminthurinus	Dorylaimoidea	**0.618**	**-0.604**	**3.152**	**4.763**
Sminthurinus	Qudsianematidae	**0.618**	**-0.207**	**3.152**	**3.918**
Sminthurinus	Eupodes	**0.618**	**0.005**	**3.152**	**2.277**
Sminthurinus	Mesostigmata	**0.618**	**-0.411**	**3.152**	**2.453**
Sminthurinus	Scutacarus	**0.618**	**-0.608**	**3.152**	**3.481**
Sminthurinus	Stigmaeidae	**0.618**	**0.229**	**3.152**	**1.976**
Sminthurus	Arctoseius	**1.429**	**-0.152**	**2.277**	**2.821**
Sminthurus	Dendrolaelaps	**1.429**	**0.027**	**2.277**	**1.976**
Sminthurus	Hypoaspis	**1.429**	**0.334**	**2.277**	**2.754**
Sminthurus	Lysigamasus	**1.429**	**0.407**	**2.277**	**3.318**
Sminthurus	Macrocheles	**1.429**	**0.761**	**2.277**	**2.277**
Sminthurus	Pergamasus	**1.429**	**1.081**	**2.277**	**1.976**
Sminthurus	Aporcelaimellus	**1.429**	**0.548**	**2.277**	**4.219**
Sminthurus	Dorylaimoidea	**1.429**	**-0.604**	**2.277**	**4.763**
Sminthurus	Qudsianematidae	**1.429**	**-0.207**	**2.277**	**3.918**
Sminthurus	Eupodes	**1.429**	**0.005**	**2.277**	**2.277**
Sminthurus	Mesostigmata	**1.429**	**-0.411**	**2.277**	**2.453**
Sminthurus	Scutacarus	**1.429**	**-0.608**	**2.277**	**3.481**
Sminthurus	Stigmaeidae	**1.429**	**0.229**	**2.277**	**1.976**
Sphaeridia	Arctoseius	**0.202**	**-0.152**	**3.122**	**2.821**
Sphaeridia	Dendrolaelaps	**0.202**	**0.027**	**3.122**	**1.976**
Sphaeridia	Hypoaspis	**0.202**	**0.334**	**3.122**	**2.754**
Sphaeridia	Lysigamasus	**0.202**	**0.407**	**3.122**	**3.318**
Sphaeridia	Macrocheles	**0.202**	**0.761**	**3.122**	**2.277**
Sphaeridia	Pergamasus	**0.202**	**1.081**	**3.122**	**1.976**
Sphaeridia	Aporcelaimellus	**0.202**	**0.548**	**3.122**	**4.219**
Sphaeridia	Dorylaimoidea	**0.202**	**-0.604**	**3.122**	**4.763**
Sphaeridia	Qudsianematidae	**0.202**	**-0.207**	**3.122**	**3.918**
Sphaeridia	Eupodes	**0.202**	**0.005**	**3.122**	**2.277**
Sphaeridia	Mesostigmata	**0.202**	**-0.411**	**3.122**	**2.453**
Sphaeridia	Scutacarus	**0.202**	**-0.608**	**3.122**	**3.481**
Sphaeridia	Stigmaeidae	**0.202**	**0.229**	**3.122**	**1.976**
Aphelenchoides	Mylonchulus	**-1.496**	**-0.005**	**4.219**	**3.918**
Aphelenchoides	Alliphis	**-1.496**	**0.053**	**4.219**	**3.438**
Aphelenchoides	Arctoseius	**-1.496**	**-0.152**	**4.219**	**2.821**
Aphelenchoides	Dendrolaelaps	**-1.496**	**0.027**	**4.219**	**1.976**
Aphelenchoides	Hypoaspis	**-1.496**	**0.334**	**4.219**	**2.754**

Aphelenchoides	Lysigamasus	**-1.496**	**0.407**	**4.219**	**3.318**
Aphelenchoides	Macrocheles	**-1.496**	**0.761**	**4.219**	**2.277**
Aphelenchoides	Pergamasus	**-1.496**	**1.081**	**4.219**	**1.976**
Aphelenchoides	Aporcelaimellus	**-1.496**	**0.548**	**4.219**	**4.219**
Aphelenchoides	Dorylaimoidea	**-1.496**	**-0.604**	**4.219**	**4.763**
Aphelenchoides	Qudsianematidae	**-1.496**	**-0.207**	**4.219**	**3.918**
Aphelenchoides	Eupodes	**-1.496**	**0.005**	**4.219**	**2.277**
Aphelenchoides	Mesostigmata	**-1.496**	**-0.411**	**4.219**	**2.453**
Aphelenchoides	Scutacarus	**-1.496**	**-0.608**	**4.219**	**3.481**
Aphelenchoides	Stigmaeidae	**-1.496**	**0.229**	**4.219**	**1.976**
Tylenchidae	Mylonchulus	**-1.360**	**-0.005**	**5.122**	**3.918**
Tylenchidae	Alliphis	**-1.360**	**0.053**	**5.122**	**3.438**
Tylenchidae	Arctoseius	**-1.360**	**-0.152**	**5.122**	**2.821**
Tylenchidae	Dendrolaelaps	**-1.360**	**0.027**	**5.122**	**1.976**
Tylenchidae	Hypoaspis	**-1.360**	**0.334**	**5.122**	**2.754**
Tylenchidae	Lysigamasus	**-1.360**	**0.407**	**5.122**	**3.318**
Tylenchidae	Macrocheles	**-1.360**	**0.761**	**5.122**	**2.277**
Tylenchidae	Pergamasus	**-1.360**	**1.081**	**5.122**	**1.976**
Tylenchidae	Aporcelaimellus	**-1.360**	**0.548**	**5.122**	**4.219**
Tylenchidae	Dorylaimoidea	**-1.360**	**-0.604**	**5.122**	**4.763**
Tylenchidae	Qudsianematidae	**-1.360**	**-0.207**	**5.122**	**3.918**
Tylenchidae	Eupodes	**-1.360**	**0.005**	**5.122**	**2.277**
Tylenchidae	Mesostigmata	**-1.360**	**-0.411**	**5.122**	**2.453**
Tylenchidae	Scutacarus	**-1.360**	**-0.608**	**5.122**	**3.481**
Tylenchidae	Stigmaeidae	**-1.360**	**0.229**	**5.122**	**1.976**
Medioppia	Arctoseius	**-0.235**	**-0.152**	**2.453**	**2.821**
Medioppia	Dendrolaelaps	**-0.235**	**0.027**	**2.453**	**1.976**
Medioppia	Hypoaspis	**-0.235**	**0.334**	**2.453**	**2.754**
Medioppia	Lysigamasus	**-0.235**	**0.407**	**2.453**	**3.318**
Medioppia	Macrocheles	**-0.235**	**0.761**	**2.453**	**2.277**
Medioppia	Pergamasus	**-0.235**	**1.081**	**2.453**	**1.976**
Medioppia	Aporcelaimellus	**-0.235**	**0.548**	**2.453**	**4.219**
Medioppia	Dorylaimoidea	**-0.235**	**-0.604**	**2.453**	**4.763**
Medioppia	Qudsianematidae	**-0.235**	**-0.207**	**2.453**	**3.918**
Medioppia	Eupodes	**-0.235**	**0.005**	**2.453**	**2.277**
Medioppia	Mesostigmata	**-0.235**	**-0.411**	**2.453**	**2.453**
Medioppia	Scutacarus	**-0.235**	**-0.608**	**2.453**	**3.481**
Medioppia	Stigmaeidae	**-0.235**	**0.229**	**2.453**	**1.976**
Microppia	Arctoseius	**-0.544**	**-0.152**	**1.976**	**2.821**
Microppia	Dendrolaelaps	**-0.544**	**0.027**	**1.976**	**1.976**
Microppia	Hypoaspis	**-0.544**	**0.334**	**1.976**	**2.754**
Microppia	Lysigamasus	**-0.544**	**0.407**	**1.976**	**3.318**
Microppia	Macrocheles	**-0.544**	**0.761**	**1.976**	**2.277**
Microppia	Pergamasus	**-0.544**	**1.081**	**1.976**	**1.976**
Microppia	Aporcelaimellus	**-0.544**	**0.548**	**1.976**	**4.219**
Microppia	Dorylaimoidea	**-0.544**	**-0.604**	**1.976**	**4.763**
Microppia	Qudsianematidae	**-0.544**	**-0.207**	**1.976**	**3.918**
Microppia	Eupodes	**-0.544**	**0.005**	**1.976**	**2.277**
Microppia	Mesostigmata	**-0.544**	**-0.411**	**1.976**	**2.453**
Microppia	Scutacarus	**-0.544**	**-0.608**	**1.976**	**3.481**
Microppia	Stigmaeidae	**-0.544**	**0.229**	**1.976**	**1.976**

Microtydeus	Arctoseius	**-0.863**	**-0.152**	**2.578**	**2.821**
Microtydeus	Dendrolaelaps	**-0.863**	**0.027**	**2.578**	**1.976**
Microtydeus	Hypoaspis	**-0.863**	**0.334**	**2.578**	**2.754**
Microtydeus	Lysigamasus	**-0.863**	**0.407**	**2.578**	**3.318**
Microtydeus	Macrocheles	**-0.863**	**0.761**	**2.578**	**2.277**
Microtydeus	Pergamasus	**-0.863**	**1.081**	**2.578**	**1.976**
Microtydeus	Aporcelaimellus	**-0.863**	**0.548**	**2.578**	**4.219**
Microtydeus	Dorylaimoidea	**-0.863**	**-0.604**	**2.578**	**4.763**
Microtydeus	Qudsianematidae	**-0.863**	**-0.207**	**2.578**	**3.918**
Microtydeus	Eupodes	**-0.863**	**0.005**	**2.578**	**2.277**
Microtydeus	Mesostigmata	**-0.863**	**-0.411**	**2.578**	**2.453**
Microtydeus	Scutacarus	**-0.863**	**-0.608**	**2.578**	**3.481**
Microtydeus	Stigmaeidae	**-0.863**	**0.229**	**2.578**	**1.976**
Oppiella	Arctoseius	**-0.447**	**-0.152**	**1.976**	**2.821**
Oppiella	Dendrolaelaps	**-0.447**	**0.027**	**1.976**	**1.976**
Oppiella	Hypoaspis	**-0.447**	**0.334**	**1.976**	**2.754**
Oppiella	Lysigamasus	**-0.447**	**0.407**	**1.976**	**3.318**
Oppiella	Macrocheles	**-0.447**	**0.761**	**1.976**	**2.277**
Oppiella	Pergamasus	**-0.447**	**1.081**	**1.976**	**1.976**
Oppiella	Aporcelaimellus	**-0.447**	**0.548**	**1.976**	**4.219**
Oppiella	Dorylaimoidea	**-0.447**	**-0.604**	**1.976**	**4.763**
Oppiella	Qudsianematidae	**-0.447**	**-0.207**	**1.976**	**3.918**
Oppiella	Eupodes	**-0.447**	**0.005**	**1.976**	**2.277**
Oppiella	Mesostigmata	**-0.447**	**-0.411**	**1.976**	**2.453**
Oppiella	Scutacarus	**-0.447**	**-0.608**	**1.976**	**3.481**
Oppiella	Stigmaeidae	**-0.447**	**0.229**	**1.976**	**1.976**
Pygmephorus	Arctoseius	**-0.376**	**-0.152**	**3.017**	**2.821**
Pygmephorus	Dendrolaelaps	**-0.376**	**0.027**	**3.017**	**1.976**
Pygmephorus	Hypoaspis	**-0.376**	**0.334**	**3.017**	**2.754**
Pygmephorus	Lysigamasus	**-0.376**	**0.407**	**3.017**	**3.318**
Pygmephorus	Macrocheles	**-0.376**	**0.761**	**3.017**	**2.277**
Pygmephorus	Pergamasus	**-0.376**	**1.081**	**3.017**	**1.976**
Pygmephorus	Aporcelaimellus	**-0.376**	**0.548**	**3.017**	**4.219**
Pygmephorus	Dorylaimoidea	**-0.376**	**-0.604**	**3.017**	**4.763**
Pygmephorus	Qudsianematidae	**-0.376**	**-0.207**	**3.017**	**3.918**
Pygmephorus	Eupodes	**-0.376**	**0.005**	**3.017**	**2.277**
Pygmephorus	Mesostigmata	**-0.376**	**-0.411**	**3.017**	**2.453**
Pygmephorus	Scutacarus	**-0.376**	**-0.608**	**3.017**	**3.481**
Pygmephorus	Stigmaeidae	**-0.376**	**0.229**	**3.017**	**1.976**
Tectocepheus	Arctoseius	**-0.220**	**-0.152**	**1.976**	**2.821**
Tectocepheus	Dendrolaelaps	**-0.220**	**0.027**	**1.976**	**1.976**
Tectocepheus	Hypoaspis	**-0.220**	**0.334**	**1.976**	**2.754**
Tectocepheus	Lysigamasus	**-0.220**	**0.407**	**1.976**	**3.318**
Tectocepheus	Macrocheles	**-0.220**	**0.761**	**1.976**	**2.277**
Tectocepheus	Pergamasus	**-0.220**	**1.081**	**1.976**	**1.976**
Tectocepheus	Aporcelaimellus	**-0.220**	**0.548**	**1.976**	**4.219**
Tectocepheus	Dorylaimoidea	**-0.220**	**-0.604**	**1.976**	**4.763**
Tectocepheus	Qudsianematidae	**-0.220**	**-0.207**	**1.976**	**3.918**
Tectocepheus	Eupodes	**-0.220**	**0.005**	**1.976**	**2.277**
Tectocepheus	Mesostigmata	**-0.220**	**-0.411**	**1.976**	**2.453**
Tectocepheus	Scutacarus	**-0.220**	**-0.608**	**1.976**	**3.481**

Tectocepheus	Stigmaeidae	**-0.220**	**0.229**	**1.976**	**1.976**
Brachystomella	Arctoseius	**0.977**	**-0.152**	**2.453**	**2.821**
Brachystomella	Dendrolaelaps	**0.977**	**0.027**	**2.453**	**1.976**
Brachystomella	Hypoaspis	**0.977**	**0.334**	**2.453**	**2.754**
Brachystomella	Lysigamasus	**0.977**	**0.407**	**2.453**	**3.318**
Brachystomella	Macrocheles	**0.977**	**0.761**	**2.453**	**2.277**
Brachystomella	Pergamasus	**0.977**	**1.081**	**2.453**	**1.976**
Brachystomella	Aporcelaimellus	**0.977**	**0.548**	**2.453**	**4.219**
Brachystomella	Dorylaimoidea	**0.977**	**-0.604**	**2.453**	**4.763**
Brachystomella	Qudsianematidae	**0.977**	**-0.207**	**2.453**	**3.918**
Brachystomella	Eupodes	**0.977**	**0.005**	**2.453**	**2.277**
Brachystomella	Mesostigmata	**0.977**	**-0.411**	**2.453**	**2.453**
Brachystomella	Scutacarus	**0.977**	**-0.608**	**2.453**	**3.481**
Brachystomella	Stigmaeidae	**0.977**	**0.229**	**2.453**	**1.976**
Friesea	Arctoseius	**0.434**	**-0.152**	**2.675**	**2.821**
Friesea	Dendrolaelaps	**0.434**	**0.027**	**2.675**	**1.976**
Friesea	Hypoaspis	**0.434**	**0.334**	**2.675**	**2.754**
Friesea	Lysigamasus	**0.434**	**0.407**	**2.675**	**3.318**
Friesea	Macrocheles	**0.434**	**0.761**	**2.675**	**2.277**
Friesea	Pergamasus	**0.434**	**1.081**	**2.675**	**1.976**
Friesea	Aporcelaimellus	**0.434**	**0.548**	**2.675**	**4.219**
Friesea	Dorylaimoidea	**0.434**	**-0.604**	**2.675**	**4.763**
Friesea	Qudsianematidae	**0.434**	**-0.207**	**2.675**	**3.918**
Friesea	Eupodes	**0.434**	**0.005**	**2.675**	**2.277**
Friesea	Mesostigmata	**0.434**	**-0.411**	**2.675**	**2.453**
Friesea	Scutacarus	**0.434**	**-0.608**	**2.675**	**3.481**
Friesea	Stigmaeidae	**0.434**	**0.229**	**2.675**	**1.976**
Hypogastrura	Arctoseius	**0.977**	**-0.152**	**2.453**	**2.821**
Hypogastrura	Dendrolaelaps	**0.977**	**0.027**	**2.453**	**1.976**
Hypogastrura	Hypoaspis	**0.977**	**0.334**	**2.453**	**2.754**
Hypogastrura	Lysigamasus	**0.977**	**0.407**	**2.453**	**3.318**
Hypogastrura	Macrocheles	**0.977**	**0.761**	**2.453**	**2.277**
Hypogastrura	Pergamasus	**0.977**	**1.081**	**2.453**	**1.976**
Hypogastrura	Aporcelaimellus	**0.977**	**0.548**	**2.453**	**4.219**
Hypogastrura	Dorylaimoidea	**0.977**	**-0.604**	**2.453**	**4.763**
Hypogastrura	Qudsianematidae	**0.977**	**-0.207**	**2.453**	**3.918**
Hypogastrura	Eupodes	**0.977**	**0.005**	**2.453**	**2.277**
Hypogastrura	Mesostigmata	**0.977**	**-0.411**	**2.453**	**2.453**
Hypogastrura	Scutacarus	**0.977**	**-0.608**	**2.453**	**3.481**
Hypogastrura	Stigmaeidae	**0.977**	**0.229**	**2.453**	**1.976**
Isotoma	Arctoseius	**1.898**	**-0.152**	**4.119**	**2.821**
Isotoma	Dendrolaelaps	**1.898**	**0.027**	**4.119**	**1.976**
Isotoma	Hypoaspis	**1.898**	**0.334**	**4.119**	**2.754**
Isotoma	Lysigamasus	**1.898**	**0.407**	**4.119**	**3.318**
Isotoma	Macrocheles	**1.898**	**0.761**	**4.119**	**2.277**
Isotoma	Pergamasus	**1.898**	**1.081**	**4.119**	**1.976**
Isotoma	Aporcelaimellus	**1.898**	**0.548**	**4.119**	**4.219**
Isotoma	Dorylaimoidea	**1.898**	**-0.604**	**4.119**	**4.763**
Isotoma	Qudsianematidae	**1.898**	**-0.207**	**4.119**	**3.918**
Isotoma	Eupodes	**1.898**	**0.005**	**4.119**	**2.277**
Isotoma	Mesostigmata	**1.898**	**-0.411**	**4.119**	**2.453**

Isotoma	Scutacarus	**1.898**	**-0.608**	**4.119**	**3.481**
Isotoma	Stigmaeidae	**1.898**	**0.229**	**4.119**	**1.976**
Isotomiella	Arctoseius	**0.816**	**-0.152**	**1.976**	**2.821**
Isotomiella	Dendrolaelaps	**0.816**	**0.027**	**1.976**	**1.976**
Isotomiella	Hypoaspis	**0.816**	**0.334**	**1.976**	**2.754**
Isotomiella	Lysigamasus	**0.816**	**0.407**	**1.976**	**3.318**
Isotomiella	Macrocheles	**0.816**	**0.761**	**1.976**	**2.277**
Isotomiella	Pergamasus	**0.816**	**1.081**	**1.976**	**1.976**
Isotomiella	Aporcelaimellus	**0.816**	**0.548**	**1.976**	**4.219**
Isotomiella	Dorylaimoidea	**0.816**	**-0.604**	**1.976**	**4.763**
Isotomiella	Qudsianematidae	**0.816**	**-0.207**	**1.976**	**3.918**
Isotomiella	Eupodes	**0.816**	**0.005**	**1.976**	**2.277**
Isotomiella	Mesostigmata	**0.816**	**-0.411**	**1.976**	**2.453**
Isotomiella	Scutacarus	**0.816**	**-0.608**	**1.976**	**3.481**
Isotomiella	Stigmaeidae	**0.816**	**0.229**	**1.976**	**1.976**
Isotomurus	Arctoseius	**1.787**	**-0.152**	**3.152**	**2.821**
Isotomurus	Dendrolaelaps	**1.787**	**0.027**	**3.152**	**1.976**
Isotomurus	Hypoaspis	**1.787**	**0.334**	**3.152**	**2.754**
Isotomurus	Lysigamasus	**1.787**	**0.407**	**3.152**	**3.318**
Isotomurus	Macrocheles	**1.787**	**0.761**	**3.152**	**2.277**
Isotomurus	Pergamasus	**1.787**	**1.081**	**3.152**	**1.976**
Isotomurus	Aporcelaimellus	**1.787**	**0.548**	**3.152**	**4.219**
Isotomurus	Dorylaimoidea	**1.787**	**-0.604**	**3.152**	**4.763**
Isotomurus	Qudsianematidae	**1.787**	**-0.207**	**3.152**	**3.918**
Isotomurus	Eupodes	**1.787**	**0.005**	**3.152**	**2.277**
Isotomurus	Mesostigmata	**1.787**	**-0.411**	**3.152**	**2.453**
Isotomurus	Scutacarus	**1.787**	**-0.608**	**3.152**	**3.481**
Isotomurus	Stigmaeidae	**1.787**	**0.229**	**3.152**	**1.976**
Lepidocyrtus	Arctoseius	**1.231**	**-0.152**	**2.821**	**2.821**
Lepidocyrtus	Dendrolaelaps	**1.231**	**0.027**	**2.821**	**1.976**
Lepidocyrtus	Hypoaspis	**1.231**	**0.334**	**2.821**	**2.754**
Lepidocyrtus	Lysigamasus	**1.231**	**0.407**	**2.821**	**3.318**
Lepidocyrtus	Macrocheles	**1.231**	**0.761**	**2.821**	**2.277**
Lepidocyrtus	Pergamasus	**1.231**	**1.081**	**2.821**	**1.976**
Lepidocyrtus	Aporcelaimellus	**1.231**	**0.548**	**2.821**	**4.219**
Lepidocyrtus	Dorylaimoidea	**1.231**	**-0.604**	**2.821**	**4.763**
Lepidocyrtus	Qudsianematidae	**1.231**	**-0.207**	**2.821**	**3.918**
Lepidocyrtus	Eupodes	**1.231**	**0.005**	**2.821**	**2.277**
Lepidocyrtus	Mesostigmata	**1.231**	**-0.411**	**2.821**	**2.453**
Lepidocyrtus	Scutacarus	**1.231**	**-0.608**	**2.821**	**3.481**
Lepidocyrtus	Stigmaeidae	**1.231**	**0.229**	**2.821**	**1.976**
Parisotoma	Arctoseius	**0.722**	**-0.152**	**3.090**	**2.821**
Parisotoma	Dendrolaelaps	**0.722**	**0.027**	**3.090**	**1.976**
Parisotoma	Hypoaspis	**0.722**	**0.334**	**3.090**	**2.754**
Parisotoma	Lysigamasus	**0.722**	**0.407**	**3.090**	**3.318**
Parisotoma	Macrocheles	**0.722**	**0.761**	**3.090**	**2.277**
Parisotoma	Pergamasus	**0.722**	**1.081**	**3.090**	**1.976**
Parisotoma	Aporcelaimellus	**0.722**	**0.548**	**3.090**	**4.219**
Parisotoma	Dorylaimoidea	**0.722**	**-0.604**	**3.090**	**4.763**
Parisotoma	Qudsianematidae	**0.722**	**-0.207**	**3.090**	**3.918**
Parisotoma	Eupodes	**0.722**	**0.005**	**3.090**	**2.277**

Parisotoma	Mesostigmata	**0.722**	**-0.411**	**3.090**	**2.453**
Parisotoma	Scutacarus	**0.722**	**-0.608**	**3.090**	**3.481**
Parisotoma	Stigmaeidae	**0.722**	**0.229**	**3.090**	**1.976**
Proisotoma	Arctoseius	**0.770**	**-0.152**	**2.879**	**2.821**
Proisotoma	Dendrolaelaps	**0.770**	**0.027**	**2.879**	**1.976**
Proisotoma	Hypoaspis	**0.770**	**0.334**	**2.879**	**2.754**
Proisotoma	Lysigamasus	**0.770**	**0.407**	**2.879**	**3.318**
Proisotoma	Macrocheles	**0.770**	**0.761**	**2.879**	**2.277**
Proisotoma	Pergamasus	**0.770**	**1.081**	**2.879**	**1.976**
Proisotoma	Aporcelaimellus	**0.770**	**0.548**	**2.879**	**4.219**
Proisotoma	Dorylaimoidea	**0.770**	**-0.604**	**2.879**	**4.763**
Proisotoma	Qudsianematidae	**0.770**	**-0.207**	**2.879**	**3.918**
Proisotoma	Eupodes	**0.770**	**0.005**	**2.879**	**2.277**
Proisotoma	Mesostigmata	**0.770**	**-0.411**	**2.879**	**2.453**
Proisotoma	Scutacarus	**0.770**	**-0.608**	**2.879**	**3.481**
Proisotoma	Stigmaeidae	**0.770**	**0.229**	**2.879**	**1.976**
Achaeta	Arctoseius	**0.768**	**-0.152**	**2.849**	**2.821**
Achaeta	Dendrolaelaps	**0.768**	**0.027**	**2.849**	**1.976**
Achaeta	Hypoaspis	**0.768**	**0.334**	**2.849**	**2.754**
Achaeta	Lysigamasus	**0.768**	**0.407**	**2.849**	**3.318**
Achaeta	Macrocheles	**0.768**	**0.761**	**2.849**	**2.277**
Achaeta	Pergamasus	**0.768**	**1.081**	**2.849**	**1.976**
Achaeta	Aporcelaimellus	**0.768**	**0.548**	**2.849**	**4.219**
Achaeta	Dorylaimoidea	**0.768**	**-0.604**	**2.849**	**4.763**
Achaeta	Qudsianematidae	**0.768**	**-0.207**	**2.849**	**3.918**
Achaeta	Eupodes	**0.768**	**0.005**	**2.849**	**2.277**
Achaeta	Mesostigmata	**0.768**	**-0.411**	**2.849**	**2.453**
Achaeta	Scutacarus	**0.768**	**-0.608**	**2.849**	**3.481**
Achaeta	Stigmaeidae	**0.768**	**0.229**	**2.849**	**1.976**
Fridericia	Arctoseius	**1.644**	**-0.152**	**3.620**	**2.821**
Fridericia	Dendrolaelaps	**1.644**	**0.027**	**3.620**	**1.976**
Fridericia	Hypoaspis	**1.644**	**0.334**	**3.620**	**2.754**
Fridericia	Lysigamasus	**1.644**	**0.407**	**3.620**	**3.318**
Fridericia	Macrocheles	**1.644**	**0.761**	**3.620**	**2.277**
Fridericia	Pergamasus	**1.644**	**1.081**	**3.620**	**1.976**
Fridericia	Aporcelaimellus	**1.644**	**0.548**	**3.620**	**4.219**
Fridericia	Dorylaimoidea	**1.644**	**-0.604**	**3.620**	**4.763**
Fridericia	Qudsianematidae	**1.644**	**-0.207**	**3.620**	**3.918**
Fridericia	Eupodes	**1.644**	**0.005**	**3.620**	**2.277**
Fridericia	Mesostigmata	**1.644**	**-0.411**	**3.620**	**2.453**
Fridericia	Scutacarus	**1.644**	**-0.608**	**3.620**	**3.481**
Fridericia	Stigmaeidae	**1.644**	**0.229**	**3.620**	**1.976**
Acrobeloides	Mylonchulus	**-1.171**	**-0.005**	**4.617**	**3.918**
Acrobeloides	Alliphis	**-1.171**	**0.053**	**4.617**	**3.438**
Acrobeloides	Arctoseius	**-1.171**	**-0.152**	**4.617**	**2.821**
Acrobeloides	Dendrolaelaps	**-1.171**	**0.027**	**4.617**	**1.976**
Acrobeloides	Hypoaspis	**-1.171**	**0.334**	**4.617**	**2.754**
Acrobeloides	Lysigamasus	**-1.171**	**0.407**	**4.617**	**3.318**
Acrobeloides	Macrocheles	**-1.171**	**0.761**	**4.617**	**2.277**
Acrobeloides	Pergamasus	**-1.171**	**1.081**	**4.617**	**1.976**
Acrobeloides	Aporcelaimellus	**-1.171**	**0.548**	**4.617**	**4.219**

Acrobeloides	Dorylaimoidea	**-1.171**	**-0.604**	**4.617**	**4.763**
Acrobeloides	Qudsianematidae	**-1.171**	**-0.207**	**4.617**	**3.918**
Acrobeloides	Eupodes	**-1.171**	**0.005**	**4.617**	**2.277**
Acrobeloides	Mesostigmata	**-1.171**	**-0.411**	**4.617**	**2.453**
Acrobeloides	Scutacarus	**-1.171**	**-0.608**	**4.617**	**3.481**
Acrobeloides	Stigmaeidae	**-1.171**	**0.229**	**4.617**	**1.976**
Anaplectus	Mylonchulus	**-0.519**	**-0.005**	**4.219**	**3.918**
Anaplectus	Alliphis	**-0.519**	**0.053**	**4.219**	**3.438**
Anaplectus	Arctoseius	**-0.519**	**-0.152**	**4.219**	**2.821**
Anaplectus	Dendrolaelaps	**-0.519**	**0.027**	**4.219**	**1.976**
Anaplectus	Hypoaspis	**-0.519**	**0.334**	**4.219**	**2.754**
Anaplectus	Lysigamasus	**-0.519**	**0.407**	**4.219**	**3.318**
Anaplectus	Macrocheles	**-0.519**	**0.761**	**4.219**	**2.277**
Anaplectus	Pergamasus	**-0.519**	**1.081**	**4.219**	**1.976**
Anaplectus	Aporcelaimellus	**-0.519**	**0.548**	**4.219**	**4.219**
Anaplectus	Dorylaimoidea	**-0.519**	**-0.604**	**4.219**	**4.763**
Anaplectus	Qudsianematidae	**-0.519**	**-0.207**	**4.219**	**3.918**
Anaplectus	Eupodes	**-0.519**	**0.005**	**4.219**	**2.277**
Anaplectus	Mesostigmata	**-0.519**	**-0.411**	**4.219**	**2.453**
Anaplectus	Scutacarus	**-0.519**	**-0.608**	**4.219**	**3.481**
Anaplectus	Stigmaeidae	**-0.519**	**0.229**	**4.219**	**1.976**
Cephalobidae	Mylonchulus	**-1.055**	**-0.005**	**4.219**	**3.918**
Cephalobidae	Alliphis	**-1.055**	**0.053**	**4.219**	**3.438**
Cephalobidae	Arctoseius	**-1.055**	**-0.152**	**4.219**	**2.821**
Cephalobidae	Dendrolaelaps	**-1.055**	**0.027**	**4.219**	**1.976**
Cephalobidae	Hypoaspis	**-1.055**	**0.334**	**4.219**	**2.754**
Cephalobidae	Lysigamasus	**-1.055**	**0.407**	**4.219**	**3.318**
Cephalobidae	Macrocheles	**-1.055**	**0.761**	**4.219**	**2.277**
Cephalobidae	Pergamasus	**-1.055**	**1.081**	**4.219**	**1.976**
Cephalobidae	Aporcelaimellus	**-1.055**	**0.548**	**4.219**	**4.219**
Cephalobidae	Dorylaimoidea	**-1.055**	**-0.604**	**4.219**	**4.763**
Cephalobidae	Qudsianematidae	**-1.055**	**-0.207**	**4.219**	**3.918**
Cephalobidae	Eupodes	**-1.055**	**0.005**	**4.219**	**2.277**
Cephalobidae	Mesostigmata	**-1.055**	**-0.411**	**4.219**	**2.453**
Cephalobidae	Scutacarus	**-1.055**	**-0.608**	**4.219**	**3.481**
Cephalobidae	Stigmaeidae	**-1.055**	**0.229**	**4.219**	**1.976**
Eucephalobus	Mylonchulus	**-0.855**	**-0.005**	**5.173**	**3.918**
Eucephalobus	Alliphis	**-0.855**	**0.053**	**5.173**	**3.438**
Eucephalobus	Arctoseius	**-0.855**	**-0.152**	**5.173**	**2.821**
Eucephalobus	Dendrolaelaps	**-0.855**	**0.027**	**5.173**	**1.976**
Eucephalobus	Hypoaspis	**-0.855**	**0.334**	**5.173**	**2.754**
Eucephalobus	Lysigamasus	**-0.855**	**0.407**	**5.173**	**3.318**
Eucephalobus	Macrocheles	**-0.855**	**0.761**	**5.173**	**2.277**
Eucephalobus	Pergamasus	**-0.855**	**1.081**	**5.173**	**1.976**
Eucephalobus	Aporcelaimellus	**-0.855**	**0.548**	**5.173**	**4.219**
Eucephalobus	Dorylaimoidea	**-0.855**	**-0.604**	**5.173**	**4.763**
Eucephalobus	Qudsianematidae	**-0.855**	**-0.207**	**5.173**	**3.918**
Eucephalobus	Eupodes	**-0.855**	**0.005**	**5.173**	**2.277**
Eucephalobus	Mesostigmata	**-0.855**	**-0.411**	**5.173**	**2.453**
Eucephalobus	Scutacarus	**-0.855**	**-0.608**	**5.173**	**3.481**
Eucephalobus	Stigmaeidae	**-0.855**	**0.229**	**5.173**	**1.976**

Panagrolaimus	Mylonchulus	**-0.945**	**-0.005**	**4.395**	**3.918**
Panagrolaimus	Alliphis	**-0.945**	**0.053**	**4.395**	**3.438**
Panagrolaimus	Arctoseius	**-0.945**	**-0.152**	**4.395**	**2.821**
Panagrolaimus	Dendrolaelaps	**-0.945**	**0.027**	**4.395**	**1.976**
Panagrolaimus	Hypoaspis	**-0.945**	**0.334**	**4.395**	**2.754**
Panagrolaimus	Lysigamasus	**-0.945**	**0.407**	**4.395**	**3.318**
Panagrolaimus	Macrocheles	**-0.945**	**0.761**	**4.395**	**2.277**
Panagrolaimus	Pergamasus	**-0.945**	**1.081**	**4.395**	**1.976**
Panagrolaimus	Aporcelaimellus	**-0.945**	**0.548**	**4.395**	**4.219**
Panagrolaimus	Dorylaimoidea	**-0.945**	**-0.604**	**4.395**	**4.763**
Panagrolaimus	Qudsianematidae	**-0.945**	**-0.207**	**4.395**	**3.918**
Panagrolaimus	Eupodes	**-0.945**	**0.005**	**4.395**	**2.277**
Panagrolaimus	Mesostigmata	**-0.945**	**-0.411**	**4.395**	**2.453**
Panagrolaimus	Scutacarus	**-0.945**	**-0.608**	**4.395**	**3.481**
Panagrolaimus	Stigmaeidae	**-0.945**	**0.229**	**4.395**	**1.976**
Plectus	Mylonchulus	**-0.583**	**-0.005**	**4.520**	**3.918**
Plectus	Alliphis	**-0.583**	**0.053**	**4.520**	**3.438**
Plectus	Arctoseius	**-0.583**	**-0.152**	**4.520**	**2.821**
Plectus	Dendrolaelaps	**-0.583**	**0.027**	**4.520**	**1.976**
Plectus	Hypoaspis	**-0.583**	**0.334**	**4.520**	**2.754**
Plectus	Lysigamasus	**-0.583**	**0.407**	**4.520**	**3.318**
Plectus	Macrocheles	**-0.583**	**0.761**	**4.520**	**2.277**
Plectus	Pergamasus	**-0.583**	**1.081**	**4.520**	**1.976**
Plectus	Aporcelaimellus	**-0.583**	**0.548**	**4.520**	**4.219**
Plectus	Dorylaimoidea	**-0.583**	**-0.604**	**4.520**	**4.763**
Plectus	Qudsianematidae	**-0.583**	**-0.207**	**4.520**	**3.918**
Plectus	Eupodes	**-0.583**	**0.005**	**4.520**	**2.277**
Plectus	Mesostigmata	**-0.583**	**-0.411**	**4.520**	**2.453**
Plectus	Scutacarus	**-0.583**	**-0.608**	**4.520**	**3.481**
Plectus	Stigmaeidae	**-0.583**	**0.229**	**4.520**	**1.976**
Prismatolaimus	Mylonchulus	**-1.280**	**-0.005**	**4.219**	**3.918**
Prismatolaimus	Alliphis	**-1.280**	**0.053**	**4.219**	**3.438**
Prismatolaimus	Arctoseius	**-1.280**	**-0.152**	**4.219**	**2.821**
Prismatolaimus	Dendrolaelaps	**-1.280**	**0.027**	**4.219**	**1.976**
Prismatolaimus	Hypoaspis	**-1.280**	**0.334**	**4.219**	**2.754**
Prismatolaimus	Lysigamasus	**-1.280**	**0.407**	**4.219**	**3.318**
Prismatolaimus	Macrocheles	**-1.280**	**0.761**	**4.219**	**2.277**
Prismatolaimus	Pergamasus	**-1.280**	**1.081**	**4.219**	**1.976**
Prismatolaimus	Aporcelaimellus	**-1.280**	**0.548**	**4.219**	**4.219**
Prismatolaimus	Dorylaimoidea	**-1.280**	**-0.604**	**4.219**	**4.763**
Prismatolaimus	Qudsianematidae	**-1.280**	**-0.207**	**4.219**	**3.918**
Prismatolaimus	Eupodes	**-1.280**	**0.005**	**4.219**	**2.277**
Prismatolaimus	Mesostigmata	**-1.280**	**-0.411**	**4.219**	**2.453**
Prismatolaimus	Scutacarus	**-1.280**	**-0.608**	**4.219**	**3.481**
Prismatolaimus	Stigmaeidae	**-1.280**	**0.229**	**4.219**	**1.976**
Rhabditidae	Mylonchulus	**-0.692**	**-0.005**	**5.551**	**3.918**
Rhabditidae	Alliphis	**-0.692**	**0.053**	**5.551**	**3.438**
Rhabditidae	Arctoseius	**-0.692**	**-0.152**	**5.551**	**2.821**
Rhabditidae	Dendrolaelaps	**-0.692**	**0.027**	**5.551**	**1.976**
Rhabditidae	Hypoaspis	**-0.692**	**0.334**	**5.551**	**2.754**
Rhabditidae	Lysigamasus	**-0.692**	**0.407**	**5.551**	**3.318**

Rhabditidae	Macrocheles	**-0.692**	**0.761**	**5.551**	**2.277**
Rhabditidae	Pergamasus	**-0.692**	**1.081**	**5.551**	**1.976**
Rhabditidae	Aporcelaimellus	**-0.692**	**0.548**	**5.551**	**4.219**
Rhabditidae	Dorylaimoidea	**-0.692**	**-0.604**	**5.551**	**4.763**
Rhabditidae	Qudsianematidae	**-0.692**	**-0.207**	**5.551**	**3.918**
Rhabditidae	Eupodes	**-0.692**	**0.005**	**5.551**	**2.277**
Rhabditidae	Mesostigmata	**-0.692**	**-0.411**	**5.551**	**2.453**
Rhabditidae	Scutacarus	**-0.692**	**-0.608**	**5.551**	**3.481**
Rhabditidae	Stigmaeidae	**-0.692**	**0.229**	**5.551**	**1.976**
Enchytraeus	Arctoseius	**0.966**	**-0.152**	**4.714**	**2.821**
Enchytraeus	Dendrolaelaps	**0.966**	**0.027**	**4.714**	**1.976**
Enchytraeus	Hypoaspis	**0.966**	**0.334**	**4.714**	**2.754**
Enchytraeus	Lysigamasus	**0.966**	**0.407**	**4.714**	**3.318**
Enchytraeus	Macrocheles	**0.966**	**0.761**	**4.714**	**2.277**
Enchytraeus	Pergamasus	**0.966**	**1.081**	**4.714**	**1.976**
Enchytraeus	Aporcelaimellus	**0.966**	**0.548**	**4.714**	**4.219**
Enchytraeus	Dorylaimoidea	**0.966**	**-0.604**	**4.714**	**4.763**
Enchytraeus	Qudsianematidae	**0.966**	**-0.207**	**4.714**	**3.918**
Enchytraeus	Eupodes	**0.966**	**0.005**	**4.714**	**2.277**
Enchytraeus	Mesostigmata	**0.966**	**-0.411**	**4.714**	**2.453**
Enchytraeus	Scutacarus	**0.966**	**-0.608**	**4.714**	**3.481**
Enchytraeus	Stigmaeidae	**0.966**	**0.229**	**4.714**	**1.976**
Eubacteria	Acrobeloides	**-6.727**	**-1.171**	**12.577**	**4.617**
Eubacteria	Anaplectus	**-6.727**	**-0.519**	**12.577**	**4.219**
Eubacteria	Cephalobidae	**-6.727**	**-1.055**	**12.577**	**4.219**
Eubacteria	Eucephalobus	**-6.727**	**-0.855**	**12.577**	**5.173**
Eubacteria	Panagrolaimus	**-6.727**	**-0.945**	**12.577**	**4.395**
Eubacteria	Plectus	**-6.727**	**-0.583**	**12.577**	**4.520**
Eubacteria	Prismatolaimus	**-6.727**	**-1.280**	**12.577**	**4.219**
Eubacteria	Rhabditidae	**-6.727**	**-0.692**	**12.577**	**5.551**
Eubacteria	Enchytraeus	**-6.727**	**0.966**	**12.577**	**4.714**
Eubacteria	Dauerlarvae	**-6.727**	**-0.804**	**12.577**	**4.918**
Eubacteria	Henlea	**-6.727**	**1.442**	**12.577**	**3.825**
Eubacteria	Marionina	**-6.727**	**0.798**	**12.577**	**3.327**
Dauerlarvae	Mylonchulus	**-0.804**	**-0.005**	**4.918**	**3.918**
Dauerlarvae	Alliphis	**-0.804**	**0.053**	**4.918**	**3.438**
Dauerlarvae	Aporcelaimellus	**-0.804**	**0.548**	**4.918**	**4.219**
Dauerlarvae	Dorylaimoidea	**-0.804**	**-0.604**	**4.918**	**4.763**
Dauerlarvae	Qudsianematidae	**-0.804**	**-0.207**	**4.918**	**3.918**
Dauerlarvae	Eupodes	**-0.804**	**0.005**	**4.918**	**2.277**
Dauerlarvae	Mesostigmata	**-0.804**	**-0.411**	**4.918**	**2.453**
Dauerlarvae	Scutacarus	**-0.804**	**-0.608**	**4.918**	**3.481**
Dauerlarvae	Stigmaeidae	**-0.804**	**0.229**	**4.918**	**1.976**
Henlea	Arctoseius	**1.442**	**-0.152**	**3.825**	**2.821**
Henlea	Dendrolaelaps	**1.442**	**0.027**	**3.825**	**1.976**
Henlea	Hypoaspis	**1.442**	**0.334**	**3.825**	**2.754**
Henlea	Lysigamasus	**1.442**	**0.407**	**3.825**	**3.318**
Henlea	Macrocheles	**1.442**	**0.761**	**3.825**	**2.277**
Henlea	Pergamasus	**1.442**	**1.081**	**3.825**	**1.976**
Henlea	Aporcelaimellus	**1.442**	**0.548**	**3.825**	**4.219**
Henlea	Dorylaimoidea	**1.442**	**-0.604**	**3.825**	**4.763**

Henlea	Qudsianematidae	**1.442**	**-0.207**	**3.825**	**3.918**
Henlea	Eupodes	**1.442**	**0.005**	**3.825**	**2.277**
Henlea	Mesostigmata	**1.442**	**-0.411**	**3.825**	**2.453**
Henlea	Scutacarus	**1.442**	**-0.608**	**3.825**	**3.481**
Henlea	Stigmaeidae	**1.442**	**0.229**	**3.825**	**1.976**
Marionina	Arctoseius	**0.798**	**-0.152**	**3.327**	**2.821**
Marionina	Dendrolaelaps	**0.798**	**0.027**	**3.327**	**1.976**
Marionina	Hypoaspis	**0.798**	**0.334**	**3.327**	**2.754**
Marionina	Lysigamasus	**0.798**	**0.407**	**3.327**	**3.318**
Marionina	Macrocheles	**0.798**	**0.761**	**3.327**	**2.277**
Marionina	Pergamasus	**0.798**	**1.081**	**3.327**	**1.976**
Marionina	Aporcelaimellus	**0.798**	**0.548**	**3.327**	**4.219**
Marionina	Dorylaimoidea	**0.798**	**-0.604**	**3.327**	**4.763**
Marionina	Qudsianematidae	**0.798**	**-0.207**	**3.327**	**3.918**
Marionina	Eupodes	**0.798**	**0.005**	**3.327**	**2.277**
Marionina	Mesostigmata	**0.798**	**-0.411**	**3.327**	**2.453**
Marionina	Scutacarus	**0.798**	**-0.608**	**3.327**	**3.481**
Marionina	Stigmaeidae	**0.798**	**0.229**	**3.327**	**1.976**
Hyphae and hair roots	Dolichodoridae	**6.897**	**-0.885**	**0.000**	**5.173**
Hyphae and hair roots	Helicotylenchus	**6.897**	**-0.792**	**0.000**	**4.696**
Hyphae and hair roots	Malenchus	**6.897**	**-1.330**	**0.000**	**4.219**
Hyphae and hair roots	Meloidogyne	**6.897**	**-1.287**	**0.000**	**3.918**
Hyphae and hair roots	Paratylenchus	**6.897**	**-1.244**	**0.000**	**4.395**
Hyphae and hair roots	Pratylenchus	**6.897**	**-1.226**	**0.000**	**4.219**
Hyphae and hair roots	Trichodorus	**6.897**	**-0.744**	**0.000**	**4.219**
Hyphae and hair roots	Tylenchorhynchus	**6.897**	**-0.664**	**0.000**	**3.918**
Hyphae and hair roots	Pachygnatidae	**6.897**	**-0.113**	**0.000**	**2.578**
Hyphae and hair roots	Tydeidae	**6.897**	**-0.608**	**0.000**	**2.879**
Hyphae and hair roots	Sminthuridae	**6.897**	**-0.608**	**0.000**	**2.578**
Hyphae and hair roots	Sminthurinus	**6.897**	**0.618**	**0.000**	**3.152**
Hyphae and hair roots	Sminthurus	**6.897**	**1.429**	**0.000**	**2.277**
Hyphae and hair roots	Sphaeridia	**6.897**	**0.202**	**0.000**	**3.122**
Hyphae and hair roots	Aphelenchoides	**6.897**	**-1.496**	**0.000**	**4.219**
Hyphae and hair roots	Tylenchidae	**6.897**	**-1.360**	**0.000**	**5.122**
Hyphae and hair roots	Medioppia	**6.897**	**-0.235**	**0.000**	**2.453**
Hyphae and hair roots	Microppia	**6.897**	**-0.544**	**0.000**	**1.976**
Hyphae and hair roots	Microtydeus	**6.897**	**-0.863**	**0.000**	**2.578**
Hyphae and hair roots	Oppiella	**6.897**	**-0.447**	**0.000**	**1.976**
Hyphae and hair roots	Pygmephorus	**6.897**	**-0.376**	**0.000**	**3.017**
Hyphae and hair roots	Tectocepheus	**6.897**	**-0.220**	**0.000**	**1.976**
Hyphae and hair roots	Brachystomella	**6.897**	**0.977**	**0.000**	**2.453**
Hyphae and hair roots	Friesea	**6.897**	**0.434**	**0.000**	**2.675**
Hyphae and hair roots	Hypogastrura	**6.897**	**0.977**	**0.000**	**2.453**
Hyphae and hair roots	Isotoma	**6.897**	**1.898**	**0.000**	**4.119**
Hyphae and hair roots	Isotomiella	**6.897**	**0.816**	**0.000**	**1.976**
Hyphae and hair roots	Isotomurus	**6.897**	**1.787**	**0.000**	**3.152**
Hyphae and hair roots	Lepidocyrtus	**6.897**	**1.231**	**0.000**	**2.821**
Hyphae and hair roots	Parisotoma	**6.897**	**0.722**	**0.000**	**3.090**
Hyphae and hair roots	Proisotoma	**6.897**	**0.770**	**0.000**	**2.879**
Hyphae and hair roots	Achaeta	**6.897**	**0.768**	**0.000**	**2.849**
Hyphae and hair roots	Fridericia	**6.897**	**1.644**	**0.000**	**3.620**

Hyphae and hair roots	Aporcelaimellus	6.897	0.548	0.000	4.219
Hyphae and hair roots	Dorylaimoidea	6.897	-0.604	0.000	4.763
Hyphae and hair roots	Qudsianematidae	6.897	-0.207	0.000	3.918
Hyphae and hair roots	Eupodes	6.897	0.005	0.000	2.277
Hyphae and hair roots	Mesostigmata	6.897	-0.411	0.000	2.453
Hyphae and hair roots	Scutacarus	6.897	-0.608	0.000	3.481
Hyphae and hair roots	Stigmaeidae	6.897	0.229	0.000	1.976
Mylonchulus	Arctoseius	-0.005	-0.152	3.918	2.821
Mylonchulus	Dendrolaelaps	-0.005	0.027	3.918	1.976
Mylonchulus	Hypoaspis	-0.005	0.334	3.918	2.754
Mylonchulus	Lysigamasus	-0.005	0.407	3.918	3.318
Mylonchulus	Macrocheles	-0.005	0.761	3.918	2.277
Mylonchulus	Pergamasus	-0.005	1.081	3.918	1.976
Mylonchulus	Aporcelaimellus	-0.005	0.548	3.918	4.219
Mylonchulus	Dorylaimoidea	-0.005	-0.604	3.918	4.763
Mylonchulus	Qudsianematidae	-0.005	-0.207	3.918	3.918
Mylonchulus	Eupodes	-0.005	0.005	3.918	2.277
Mylonchulus	Mesostigmata	-0.005	-0.411	3.918	2.453
Mylonchulus	Scutacarus	-0.005	-0.608	3.918	3.481
Mylonchulus	Stigmaeidae	-0.005	0.229	3.918	1.976
Alliphis	Arctoseius	0.053	-0.152	3.438	2.821
Alliphis	Dendrolaelaps	0.053	0.027	3.438	1.976
Alliphis	Hypoaspis	0.053	0.334	3.438	2.754
Alliphis	Lysigamasus	0.053	0.407	3.438	3.318
Alliphis	Macrocheles	0.053	0.761	3.438	2.277
Alliphis	Pergamasus	0.053	1.081	3.438	1.976
Alliphis	Aporcelaimellus	0.053	0.548	3.438	4.219
Alliphis	Dorylaimoidea	0.053	-0.604	3.438	4.763
Alliphis	Qudsianematidae	0.053	-0.207	3.438	3.918
Alliphis	Eupodes	0.053	0.005	3.438	2.277
Alliphis	Mesostigmata	0.053	-0.411	3.438	2.453
Alliphis	Scutacarus	0.053	-0.608	3.438	3.481
Alliphis	Stigmaeidae	0.053	0.229	3.438	1.976
Arctoseius	Aporcelaimellus	-0.152	0.548	2.821	4.219
Arctoseius	Dorylaimoidea	-0.152	-0.604	2.821	4.763
Arctoseius	Qudsianematidae	-0.152	-0.207	2.821	3.918
Arctoseius	Eupodes	-0.152	0.005	2.821	2.277
Arctoseius	Mesostigmata	-0.152	-0.411	2.821	2.453
Arctoseius	Scutacarus	-0.152	-0.608	2.821	3.481
Arctoseius	Stigmaeidae	-0.152	0.229	2.821	1.976
Dendrolaelaps	Aporcelaimellus	0.027	0.548	1.976	4.219
Dendrolaelaps	Dorylaimoidea	0.027	-0.604	1.976	4.763
Dendrolaelaps	Qudsianematidae	0.027	-0.207	1.976	3.918
Dendrolaelaps	Eupodes	0.027	0.005	1.976	2.277
Dendrolaelaps	Mesostigmata	0.027	-0.411	1.976	2.453
Dendrolaelaps	Scutacarus	0.027	-0.608	1.976	3.481
Dendrolaelaps	Stigmaeidae	0.027	0.229	1.976	1.976
Hypoaspis	Aporcelaimellus	0.334	0.548	2.754	4.219
Hypoaspis	Dorylaimoidea	0.334	-0.604	2.754	4.763
Hypoaspis	Qudsianematidae	0.334	-0.207	2.754	3.918
Hypoaspis	Eupodes	0.334	0.005	2.754	2.277

Hypoaspis	Mesostigmata	**0.334**	**-0.411**	**2.754**	**2.453**
Hypoaspis	Scutacarus	**0.334**	**-0.608**	**2.754**	**3.481**
Hypoaspis	Stigmaeidae	**0.334**	**0.229**	**2.754**	**1.976**
Lysigamasus	Aporcelaimellus	**0.407**	**0.548**	**3.318**	**4.219**
Lysigamasus	Dorylaimoidea	**0.407**	**-0.604**	**3.318**	**4.763**
Lysigamasus	Qudsianematidae	**0.407**	**-0.207**	**3.318**	**3.918**
Lysigamasus	Eupodes	**0.407**	**0.005**	**3.318**	**2.277**
Lysigamasus	Mesostigmata	**0.407**	**-0.411**	**3.318**	**2.453**
Lysigamasus	Scutacarus	**0.407**	**-0.608**	**3.318**	**3.481**
Lysigamasus	Stigmaeidae	**0.407**	**0.229**	**3.318**	**1.976**
Macrocheles	Aporcelaimellus	**0.761**	**0.548**	**2.277**	**4.219**
Macrocheles	Dorylaimoidea	**0.761**	**-0.604**	**2.277**	**4.763**
Macrocheles	Qudsianematidae	**0.761**	**-0.207**	**2.277**	**3.918**
Macrocheles	Eupodes	**0.761**	**0.005**	**2.277**	**2.277**
Macrocheles	Mesostigmata	**0.761**	**-0.411**	**2.277**	**2.453**
Macrocheles	Scutacarus	**0.761**	**-0.608**	**2.277**	**3.481**
Macrocheles	Stigmaeidae	**0.761**	**0.229**	**2.277**	**1.976**
Pergamasus	Aporcelaimellus	**1.081**	**0.548**	**1.976**	**4.219**
Pergamasus	Dorylaimoidea	**1.081**	**-0.604**	**1.976**	**4.763**
Pergamasus	Qudsianematidae	**1.081**	**-0.207**	**1.976**	**3.918**
Pergamasus	Eupodes	**1.081**	**0.005**	**1.976**	**2.277**
Pergamasus	Mesostigmata	**1.081**	**-0.411**	**1.976**	**2.453**
Pergamasus	Scutacarus	**1.081**	**-0.608**	**1.976**	**3.481**
Pergamasus	Stigmaeidae	**1.081**	**0.229**	**1.976**	**1.976**
Aporcelaimellus	Mylonchulus	**0.548**	**-0.005**	**4.219**	**3.918**
Aporcelaimellus	Alliphis	**0.548**	**0.053**	**4.219**	**3.438**
Aporcelaimellus	Arctoseius	**0.548**	**-0.152**	**4.219**	**2.821**
Aporcelaimellus	Dendrolaelaps	**0.548**	**0.027**	**4.219**	**1.976**
Aporcelaimellus	Hypoaspis	**0.548**	**0.334**	**4.219**	**2.754**
Aporcelaimellus	Lysigamasus	**0.548**	**0.407**	**4.219**	**3.318**
Aporcelaimellus	Macrocheles	**0.548**	**0.761**	**4.219**	**2.277**
Aporcelaimellus	Pergamasus	**0.548**	**1.081**	**4.219**	**1.976**
Aporcelaimellus	Aporcelaimellus	**0.548**	**0.548**	**4.219**	**4.219**
Aporcelaimellus	Dorylaimoidea	**0.548**	**-0.604**	**4.219**	**4.763**
Aporcelaimellus	Qudsianematidae	**0.548**	**-0.207**	**4.219**	**3.918**
Aporcelaimellus	Eupodes	**0.548**	**0.005**	**4.219**	**2.277**
Aporcelaimellus	Mesostigmata	**0.548**	**-0.411**	**4.219**	**2.453**
Aporcelaimellus	Scutacarus	**0.548**	**-0.608**	**4.219**	**3.481**
Aporcelaimellus	Stigmaeidae	**0.548**	**0.229**	**4.219**	**1.976**
Dorylaimoidea	Mylonchulus	**-0.604**	**-0.005**	**4.763**	**3.918**
Dorylaimoidea	Alliphis	**-0.604**	**0.053**	**4.763**	**3.438**
Dorylaimoidea	Arctoseius	**-0.604**	**-0.152**	**4.763**	**2.821**
Dorylaimoidea	Dendrolaelaps	**-0.604**	**0.027**	**4.763**	**1.976**
Dorylaimoidea	Hypoaspis	**-0.604**	**0.334**	**4.763**	**2.754**
Dorylaimoidea	Lysigamasus	**-0.604**	**0.407**	**4.763**	**3.318**
Dorylaimoidea	Macrocheles	**-0.604**	**0.761**	**4.763**	**2.277**
Dorylaimoidea	Pergamasus	**-0.604**	**1.081**	**4.763**	**1.976**
Dorylaimoidea	Aporcelaimellus	**-0.604**	**0.548**	**4.763**	**4.219**
Dorylaimoidea	Dorylaimoidea	**-0.604**	**-0.604**	**4.763**	**4.763**
Dorylaimoidea	Qudsianematidae	**-0.604**	**-0.207**	**4.763**	**3.918**
Dorylaimoidea	Eupodes	**-0.604**	**0.005**	**4.763**	**2.277**

Dorylaimoidea	Mesostigmata	**-0.604**	**-0.411**	**4.763**	**2.453**
Dorylaimoidea	Scutacarus	**-0.604**	**-0.608**	**4.763**	**3.481**
Dorylaimoidea	Stigmaeidae	**-0.604**	**0.229**	**4.763**	**1.976**
Qudsianematidae	Mylonchulus	**-0.207**	**-0.005**	**3.918**	**3.918**
Qudsianematidae	Alliphis	**-0.207**	**0.053**	**3.918**	**3.438**
Qudsianematidae	Arctoseius	**-0.207**	**-0.152**	**3.918**	**2.821**
Qudsianematidae	Dendrolaelaps	**-0.207**	**0.027**	**3.918**	**1.976**
Qudsianematidae	Hypoaspis	**-0.207**	**0.334**	**3.918**	**2.754**
Qudsianematidae	Lysigamasus	**-0.207**	**0.407**	**3.918**	**3.318**
Qudsianematidae	Macrocheles	**-0.207**	**0.761**	**3.918**	**2.277**
Qudsianematidae	Pergamasus	**-0.207**	**1.081**	**3.918**	**1.976**
Qudsianematidae	Aporcelaimellus	**-0.207**	**0.548**	**3.918**	**4.219**
Qudsianematidae	Dorylaimoidea	**-0.207**	**-0.604**	**3.918**	**4.763**
Qudsianematidae	Qudsianematidae	**-0.207**	**-0.207**	**3.918**	**3.918**
Qudsianematidae	Eupodes	**-0.207**	**0.005**	**3.918**	**2.277**
Qudsianematidae	Mesostigmata	**-0.207**	**-0.411**	**3.918**	**2.453**
Qudsianematidae	Scutacarus	**-0.207**	**-0.608**	**3.918**	**3.481**
Qudsianematidae	Stigmaeidae	**-0.207**	**0.229**	**3.918**	**1.976**
Eupodes	Arctoseius	**0.005**	**-0.152**	**2.277**	**2.821**
Eupodes	Dendrolaelaps	**0.005**	**0.027**	**2.277**	**1.976**
Eupodes	Hypoaspis	**0.005**	**0.334**	**2.277**	**2.754**
Eupodes	Lysigamasus	**0.005**	**0.407**	**2.277**	**3.318**
Eupodes	Macrocheles	**0.005**	**0.761**	**2.277**	**2.277**
Eupodes	Pergamasus	**0.005**	**1.081**	**2.277**	**1.976**
Eupodes	Aporcelaimellus	**0.005**	**0.548**	**2.277**	**4.219**
Eupodes	Dorylaimoidea	**0.005**	**-0.604**	**2.277**	**4.763**
Eupodes	Qudsianematidae	**0.005**	**-0.207**	**2.277**	**3.918**
Eupodes	Eupodes	**0.005**	**0.005**	**2.277**	**2.277**
Eupodes	Mesostigmata	**0.005**	**-0.411**	**2.277**	**2.453**
Eupodes	Scutacarus	**0.005**	**-0.608**	**2.277**	**3.481**
Eupodes	Stigmaeidae	**0.005**	**0.229**	**2.277**	**1.976**
Mesostigmata	Arctoseius	**-0.411**	**-0.152**	**2.453**	**2.821**
Mesostigmata	Dendrolaelaps	**-0.411**	**0.027**	**2.453**	**1.976**
Mesostigmata	Hypoaspis	**-0.411**	**0.334**	**2.453**	**2.754**
Mesostigmata	Lysigamasus	**-0.411**	**0.407**	**2.453**	**3.318**
Mesostigmata	Macrocheles	**-0.411**	**0.761**	**2.453**	**2.277**
Mesostigmata	Pergamasus	**-0.411**	**1.081**	**2.453**	**1.976**
Mesostigmata	Aporcelaimellus	**-0.411**	**0.548**	**2.453**	**4.219**
Mesostigmata	Dorylaimoidea	**-0.411**	**-0.604**	**2.453**	**4.763**
Mesostigmata	Qudsianematidae	**-0.411**	**-0.207**	**2.453**	**3.918**
Mesostigmata	Eupodes	**-0.411**	**0.005**	**2.453**	**2.277**
Mesostigmata	Mesostigmata	**-0.411**	**-0.411**	**2.453**	**2.453**
Mesostigmata	Scutacarus	**-0.411**	**-0.608**	**2.453**	**3.481**
Mesostigmata	Stigmaeidae	**-0.411**	**0.229**	**2.453**	**1.976**
Scutacarus	Arctoseius	**-0.608**	**-0.152**	**3.481**	**2.821**
Scutacarus	Dendrolaelaps	**-0.608**	**0.027**	**3.481**	**1.976**
Scutacarus	Hypoaspis	**-0.608**	**0.334**	**3.481**	**2.754**
Scutacarus	Lysigamasus	**-0.608**	**0.407**	**3.481**	**3.318**
Scutacarus	Macrocheles	**-0.608**	**0.761**	**3.481**	**2.277**
Scutacarus	Pergamasus	**-0.608**	**1.081**	**3.481**	**1.976**
Scutacarus	Aporcelaimellus	**-0.608**	**0.548**	**3.481**	**4.219**

Scutacarus	Dorylaimoidea	**-0.608**	**-0.604**	**3.481**	**4.763**
Scutacarus	Qudsianematidae	**-0.608**	**-0.207**	**3.481**	**3.918**
Scutacarus	Eupodes	**-0.608**	**0.005**	**3.481**	**2.277**
Scutacarus	Mesostigmata	**-0.608**	**-0.411**	**3.481**	**2.453**
Scutacarus	Scutacarus	**-0.608**	**-0.608**	**3.481**	**3.481**
Scutacarus	Stigmaeidae	**-0.608**	**0.229**	**3.481**	**1.976**
Stigmaeidae	Arctoseius	**0.229**	**-0.152**	**1.976**	**2.821**
Stigmaeidae	Dendrolaelaps	**0.229**	**0.027**	**1.976**	**1.976**
Stigmaeidae	Hypoaspis	**0.229**	**0.334**	**1.976**	**2.754**
Stigmaeidae	Lysigamasus	**0.229**	**0.407**	**1.976**	**3.318**
Stigmaeidae	Macrocheles	**0.229**	**0.761**	**1.976**	**2.277**
Stigmaeidae	Pergamasus	**0.229**	**1.081**	**1.976**	**1.976**
Stigmaeidae	Aporcelaimellus	**0.229**	**0.548**	**1.976**	**4.219**
Stigmaeidae	Dorylaimoidea	**0.229**	**-0.604**	**1.976**	**4.763**
Stigmaeidae	Qudsianematidae	**0.229**	**-0.207**	**1.976**	**3.918**
Stigmaeidae	Eupodes	**0.229**	**0.005**	**1.976**	**2.277**
Stigmaeidae	Mesostigmata	**0.229**	**-0.411**	**1.976**	**2.453**
Stigmaeidae	Scutacarus	**0.229**	**-0.608**	**1.976**	**3.481**
Stigmaeidae	Stigmaeidae	**0.229**	**0.229**	**1.976**	**1.976**

Resource	Consumer	Mres	Mconsumer	Nres	Nconsumer
Coslenchus	Mononchidae	**-0.821**	**-0.827**	**3.838**	**3.838**
Coslenchus	Mylonchulus	**-0.821**	**-0.005**	**3.838**	**4.139**
Coslenchus	Alliphis	**-0.821**	**0.053**	**3.838**	**2.976**
Coslenchus	Arctoseius	**-0.821**	**-0.152**	**3.838**	**1.976**
Coslenchus	Dendrolaelaps	**-0.821**	**0.027**	**3.838**	**1.976**
Coslenchus	Lysigamasus	**-0.821**	**0.407**	**3.838**	**3.318**
Coslenchus	Macrocheles	**-0.821**	**0.761**	**3.838**	**1.976**
Coslenchus	Parasitus	**-0.821**	**0.859**	**3.838**	**2.578**
Coslenchus	Uropoda	**-0.821**	**0.481**	**3.838**	**1.976**
Coslenchus	Dorylaimoidea	**-0.821**	**-0.604**	**3.838**	**4.139**
Coslenchus	Qudsianematidae	**-0.821**	**-0.207**	**3.838**	**3.838**
Coslenchus	Eupodes	**-0.821**	**0.005**	**3.838**	**2.754**
Coslenchus	Mesostigmata	**-0.821**	**-0.411**	**3.838**	**1.976**
Coslenchus	Scutacarus	**-0.821**	**-0.608**	**3.838**	**3.152**
Dolichodoridae	Mononchidae	**-0.885**	**-0.827**	**4.139**	**3.838**
Dolichodoridae	Mylonchulus	**-0.885**	**-0.005**	**4.139**	**4.139**
Dolichodoridae	Alliphis	**-0.885**	**0.053**	**4.139**	**2.976**
Dolichodoridae	Arctoseius	**-0.885**	**-0.152**	**4.139**	**1.976**
Dolichodoridae	Dendrolaelaps	**-0.885**	**0.027**	**4.139**	**1.976**
Dolichodoridae	Lysigamasus	**-0.885**	**0.407**	**4.139**	**3.318**
Dolichodoridae	Macrocheles	**-0.885**	**0.761**	**4.139**	**1.976**
Dolichodoridae	Parasitus	**-0.885**	**0.859**	**4.139**	**2.578**
Dolichodoridae	Uropoda	**-0.885**	**0.481**	**4.139**	**1.976**
Dolichodoridae	Dorylaimoidea	**-0.885**	**-0.604**	**4.139**	**4.139**
Dolichodoridae	Qudsianematidae	**-0.885**	**-0.207**	**4.139**	**3.838**
Dolichodoridae	Eupodes	**-0.885**	**0.005**	**4.139**	**2.754**
Dolichodoridae	Mesostigmata	**-0.885**	**-0.411**	**4.139**	**1.976**
Dolichodoridae	Scutacarus	**-0.885**	**-0.608**	**4.139**	**3.152**
Helicotylenchus	Mononchidae	**-0.792**	**-0.827**	**3.838**	**3.838**
Helicotylenchus	Mylonchulus	**-0.792**	**-0.005**	**3.838**	**4.139**
Helicotylenchus	Alliphis	**-0.792**	**0.053**	**3.838**	**2.976**
Helicotylenchus	Arctoseius	**-0.792**	**-0.152**	**3.838**	**1.976**
Helicotylenchus	Dendrolaelaps	**-0.792**	**0.027**	**3.838**	**1.976**
Helicotylenchus	Lysigamasus	**-0.792**	**0.407**	**3.838**	**3.318**
Helicotylenchus	Macrocheles	**-0.792**	**0.761**	**3.838**	**1.976**
Helicotylenchus	Parasitus	**-0.792**	**0.859**	**3.838**	**2.578**
Helicotylenchus	Uropoda	**-0.792**	**0.481**	**3.838**	**1.976**
Helicotylenchus	Dorylaimoidea	**-0.792**	**-0.604**	**3.838**	**4.139**
Helicotylenchus	Qudsianematidae	**-0.792**	**-0.207**	**3.838**	**3.838**
Helicotylenchus	Eupodes	**-0.792**	**0.005**	**3.838**	**2.754**
Helicotylenchus	Mesostigmata	**-0.792**	**-0.411**	**3.838**	**1.976**
Helicotylenchus	Scutacarus	**-0.792**	**-0.608**	**3.838**	**3.152**
Heterodera	Mononchidae	**-0.883**	**-0.827**	**3.838**	**3.838**
Heterodera	Mylonchulus	**-0.883**	**-0.005**	**3.838**	**4.139**
Heterodera	Alliphis	**-0.883**	**0.053**	**3.838**	**2.976**
Heterodera	Arctoseius	**-0.883**	**-0.152**	**3.838**	**1.976**
Heterodera	Dendrolaelaps	**-0.883**	**0.027**	**3.838**	**1.976**
Heterodera	Lysigamasus	**-0.883**	**0.407**	**3.838**	**3.318**
Heterodera	Macrocheles	**-0.883**	**0.761**	**3.838**	**1.976**
Heterodera	Parasitus	**-0.883**	**0.859**	**3.838**	**2.578**

Heterodera	Uropoda	**-0.883**	**0.481**	**3.838**	**1.976**
Heterodera	Dorylaimoidea	**-0.883**	**-0.604**	**3.838**	**4.139**
Heterodera	Qudsianematidae	**-0.883**	**-0.207**	**3.838**	**3.838**
Heterodera	Eupodes	**-0.883**	**0.005**	**3.838**	**2.754**
Heterodera	Mesostigmata	**-0.883**	**-0.411**	**3.838**	**1.976**
Heterodera	Scutacarus	**-0.883**	**-0.608**	**3.838**	**3.152**
Malenchus	Mononchidae	**-1.330**	**-0.827**	**4.139**	**3.838**
Malenchus	Mylonchulus	**-1.330**	**-0.005**	**4.139**	**4.139**
Malenchus	Alliphis	**-1.330**	**0.053**	**4.139**	**2.976**
Malenchus	Arctoseius	**-1.330**	**-0.152**	**4.139**	**1.976**
Malenchus	Dendrolaelaps	**-1.330**	**0.027**	**4.139**	**1.976**
Malenchus	Lysigamasus	**-1.330**	**0.407**	**4.139**	**3.318**
Malenchus	Macrocheles	**-1.330**	**0.761**	**4.139**	**1.976**
Malenchus	Parasitus	**-1.330**	**0.859**	**4.139**	**2.578**
Malenchus	Uropoda	**-1.330**	**0.481**	**4.139**	**1.976**
Malenchus	Dorylaimoidea	**-1.330**	**-0.604**	**4.139**	**4.139**
Malenchus	Qudsianematidae	**-1.330**	**-0.207**	**4.139**	**3.838**
Malenchus	Eupodes	**-1.330**	**0.005**	**4.139**	**2.754**
Malenchus	Mesostigmata	**-1.330**	**-0.411**	**4.139**	**1.976**
Malenchus	Scutacarus	**-1.330**	**-0.608**	**4.139**	**3.152**
Meloidogyne	Mononchidae	**-1.287**	**-0.827**	**4.537**	**3.838**
Meloidogyne	Mylonchulus	**-1.287**	**-0.005**	**4.537**	**4.139**
Meloidogyne	Alliphis	**-1.287**	**0.053**	**4.537**	**2.976**
Meloidogyne	Arctoseius	**-1.287**	**-0.152**	**4.537**	**1.976**
Meloidogyne	Dendrolaelaps	**-1.287**	**0.027**	**4.537**	**1.976**
Meloidogyne	Lysigamasus	**-1.287**	**0.407**	**4.537**	**3.318**
Meloidogyne	Macrocheles	**-1.287**	**0.761**	**4.537**	**1.976**
Meloidogyne	Parasitus	**-1.287**	**0.859**	**4.537**	**2.578**
Meloidogyne	Uropoda	**-1.287**	**0.481**	**4.537**	**1.976**
Meloidogyne	Dorylaimoidea	**-1.287**	**-0.604**	**4.537**	**4.139**
Meloidogyne	Qudsianematidae	**-1.287**	**-0.207**	**4.537**	**3.838**
Meloidogyne	Eupodes	**-1.287**	**0.005**	**4.537**	**2.754**
Meloidogyne	Mesostigmata	**-1.287**	**-0.411**	**4.537**	**1.976**
Meloidogyne	Scutacarus	**-1.287**	**-0.608**	**4.537**	**3.152**
Paratylenchus	Mononchidae	**-1.244**	**-0.827**	**4.440**	**3.838**
Paratylenchus	Mylonchulus	**-1.244**	**-0.005**	**4.440**	**4.139**
Paratylenchus	Alliphis	**-1.244**	**0.053**	**4.440**	**2.976**
Paratylenchus	Arctoseius	**-1.244**	**-0.152**	**4.440**	**1.976**
Paratylenchus	Dendrolaelaps	**-1.244**	**0.027**	**4.440**	**1.976**
Paratylenchus	Lysigamasus	**-1.244**	**0.407**	**4.440**	**3.318**
Paratylenchus	Macrocheles	**-1.244**	**0.761**	**4.440**	**1.976**
Paratylenchus	Parasitus	**-1.244**	**0.859**	**4.440**	**2.578**
Paratylenchus	Uropoda	**-1.244**	**0.481**	**4.440**	**1.976**
Paratylenchus	Dorylaimoidea	**-1.244**	**-0.604**	**4.440**	**4.139**
Paratylenchus	Qudsianematidae	**-1.244**	**-0.207**	**4.440**	**3.838**
Paratylenchus	Eupodes	**-1.244**	**0.005**	**4.440**	**2.754**
Paratylenchus	Mesostigmata	**-1.244**	**-0.411**	**4.440**	**1.976**
Paratylenchus	Scutacarus	**-1.244**	**-0.608**	**4.440**	**3.152**
Pratylenchus	Mononchidae	**-1.226**	**-0.827**	**3.838**	**3.838**
Pratylenchus	Mylonchulus	**-1.226**	**-0.005**	**3.838**	**4.139**
Pratylenchus	Alliphis	**-1.226**	**0.053**	**3.838**	**2.976**

Pratylenchus	Arctoseius	**-1.226**	**-0.152**	**3.838**	**1.976**
Pratylenchus	Dendrolaelaps	**-1.226**	**0.027**	**3.838**	**1.976**
Pratylenchus	Lysigamasus	**-1.226**	**0.407**	**3.838**	**3.318**
Pratylenchus	Macrocheles	**-1.226**	**0.761**	**3.838**	**1.976**
Pratylenchus	Parasitus	**-1.226**	**0.859**	**3.838**	**2.578**
Pratylenchus	Uropoda	**-1.226**	**0.481**	**3.838**	**1.976**
Pratylenchus	Dorylaimoidea	**-1.226**	**-0.604**	**3.838**	**4.139**
Pratylenchus	Qudsianematidae	**-1.226**	**-0.207**	**3.838**	**3.838**
Pratylenchus	Eupodes	**-1.226**	**0.005**	**3.838**	**2.754**
Pratylenchus	Mesostigmata	**-1.226**	**-0.411**	**3.838**	**1.976**
Pratylenchus	Scutacarus	**-1.226**	**-0.608**	**3.838**	**3.152**
Tylenchorhynchus	Mononchidae	**-0.664**	**-0.827**	**4.139**	**3.838**
Tylenchorhynchus	Mylonchulus	**-0.664**	**-0.005**	**4.139**	**4.139**
Tylenchorhynchus	Alliphis	**-0.664**	**0.053**	**4.139**	**2.976**
Tylenchorhynchus	Arctoseius	**-0.664**	**-0.152**	**4.139**	**1.976**
Tylenchorhynchus	Dendrolaelaps	**-0.664**	**0.027**	**4.139**	**1.976**
Tylenchorhynchus	Lysigamasus	**-0.664**	**0.407**	**4.139**	**3.318**
Tylenchorhynchus	Macrocheles	**-0.664**	**0.761**	**4.139**	**1.976**
Tylenchorhynchus	Parasitus	**-0.664**	**0.859**	**4.139**	**2.578**
Tylenchorhynchus	Uropoda	**-0.664**	**0.481**	**4.139**	**1.976**
Tylenchorhynchus	Dorylaimoidea	**-0.664**	**-0.604**	**4.139**	**4.139**
Tylenchorhynchus	Qudsianematidae	**-0.664**	**-0.207**	**4.139**	**3.838**
Tylenchorhynchus	Eupodes	**-0.664**	**0.005**	**4.139**	**2.754**
Tylenchorhynchus	Mesostigmata	**-0.664**	**-0.411**	**4.139**	**1.976**
Tylenchorhynchus	Scutacarus	**-0.664**	**-0.608**	**4.139**	**3.152**
Tydeidae	Arctoseius	**-0.608**	**-0.152**	**2.976**	**1.976**
Tydeidae	Dendrolaelaps	**-0.608**	**0.027**	**2.976**	**1.976**
Tydeidae	Lysigamasus	**-0.608**	**0.407**	**2.976**	**3.318**
Tydeidae	Macrocheles	**-0.608**	**0.761**	**2.976**	**1.976**
Tydeidae	Parasitus	**-0.608**	**0.859**	**2.976**	**2.578**
Tydeidae	Uropoda	**-0.608**	**0.481**	**2.976**	**1.976**
Tydeidae	Dorylaimoidea	**-0.608**	**-0.604**	**2.976**	**4.139**
Tydeidae	Qudsianematidae	**-0.608**	**-0.207**	**2.976**	**3.838**
Tydeidae	Eupodes	**-0.608**	**0.005**	**2.976**	**2.754**
Tydeidae	Mesostigmata	**-0.608**	**-0.411**	**2.976**	**1.976**
Tydeidae	Scutacarus	**-0.608**	**-0.608**	**2.976**	**3.152**
Sminthuridae	Arctoseius	**-0.608**	**-0.152**	**2.277**	**1.976**
Sminthuridae	Dendrolaelaps	**-0.608**	**0.027**	**2.277**	**1.976**
Sminthuridae	Lysigamasus	**-0.608**	**0.407**	**2.277**	**3.318**
Sminthuridae	Macrocheles	**-0.608**	**0.761**	**2.277**	**1.976**
Sminthuridae	Parasitus	**-0.608**	**0.859**	**2.277**	**2.578**
Sminthuridae	Uropoda	**-0.608**	**0.481**	**2.277**	**1.976**
Sminthuridae	Dorylaimoidea	**-0.608**	**-0.604**	**2.277**	**4.139**
Sminthuridae	Qudsianematidae	**-0.608**	**-0.207**	**2.277**	**3.838**
Sminthuridae	Eupodes	**-0.608**	**0.005**	**2.277**	**2.754**
Sminthuridae	Mesostigmata	**-0.608**	**-0.411**	**2.277**	**1.976**
Sminthuridae	Scutacarus	**-0.608**	**-0.608**	**2.277**	**3.152**
Sminthurinus	Arctoseius	**0.618**	**-0.152**	**2.976**	**1.976**
Sminthurinus	Dendrolaelaps	**0.618**	**0.027**	**2.976**	**1.976**
Sminthurinus	Lysigamasus	**0.618**	**0.407**	**2.976**	**3.318**
Sminthurinus	Macrocheles	**0.618**	**0.761**	**2.976**	**1.976**

Sminthurinus	Parasitus	**0.618**	**0.859**	**2.976**	**2.578**
Sminthurinus	Uropoda	**0.618**	**0.481**	**2.976**	**1.976**
Sminthurinus	Dorylaimoidea	**0.618**	**-0.604**	**2.976**	**4.139**
Sminthurinus	Qudsianematidae	**0.618**	**-0.207**	**2.976**	**3.838**
Sminthurinus	Eupodes	**0.618**	**0.005**	**2.976**	**2.754**
Sminthurinus	Mesostigmata	**0.618**	**-0.411**	**2.976**	**1.976**
Sminthurinus	Scutacarus	**0.618**	**-0.608**	**2.976**	**3.152**
Sminthurus	Arctoseius	**1.429**	**-0.152**	**2.453**	**1.976**
Sminthurus	Dendrolaelaps	**1.429**	**0.027**	**2.453**	**1.976**
Sminthurus	Lysigamasus	**1.429**	**0.407**	**2.453**	**3.318**
Sminthurus	Macrocheles	**1.429**	**0.761**	**2.453**	**1.976**
Sminthurus	Parasitus	**1.429**	**0.859**	**2.453**	**2.578**
Sminthurus	Uropoda	**1.429**	**0.481**	**2.453**	**1.976**
Sminthurus	Dorylaimoidea	**1.429**	**-0.604**	**2.453**	**4.139**
Sminthurus	Qudsianematidae	**1.429**	**-0.207**	**2.453**	**3.838**
Sminthurus	Eupodes	**1.429**	**0.005**	**2.453**	**2.754**
Sminthurus	Mesostigmata	**1.429**	**-0.411**	**2.453**	**1.976**
Sminthurus	Scutacarus	**1.429**	**-0.608**	**2.453**	**3.152**
Sphaeridia	Arctoseius	**0.202**	**-0.152**	**2.277**	**1.976**
Sphaeridia	Dendrolaelaps	**0.202**	**0.027**	**2.277**	**1.976**
Sphaeridia	Lysigamasus	**0.202**	**0.407**	**2.277**	**3.318**
Sphaeridia	Macrocheles	**0.202**	**0.761**	**2.277**	**1.976**
Sphaeridia	Parasitus	**0.202**	**0.859**	**2.277**	**2.578**
Sphaeridia	Uropoda	**0.202**	**0.481**	**2.277**	**1.976**
Sphaeridia	Dorylaimoidea	**0.202**	**-0.604**	**2.277**	**4.139**
Sphaeridia	Qudsianematidae	**0.202**	**-0.207**	**2.277**	**3.838**
Sphaeridia	Eupodes	**0.202**	**0.005**	**2.277**	**2.754**
Sphaeridia	Mesostigmata	**0.202**	**-0.411**	**2.277**	**1.976**
Sphaeridia	Scutacarus	**0.202**	**-0.608**	**2.277**	**3.152**
Aphelenchoides	Mononchidae	**-1.496**	**-0.827**	**4.616**	**3.838**
Aphelenchoides	Mylonchulus	**-1.496**	**-0.005**	**4.616**	**4.139**
Aphelenchoides	Alliphis	**-1.496**	**0.053**	**4.616**	**2.976**
Aphelenchoides	Arctoseius	**-1.496**	**-0.152**	**4.616**	**1.976**
Aphelenchoides	Dendrolaelaps	**-1.496**	**0.027**	**4.616**	**1.976**
Aphelenchoides	Lysigamasus	**-1.496**	**0.407**	**4.616**	**3.318**
Aphelenchoides	Macrocheles	**-1.496**	**0.761**	**4.616**	**1.976**
Aphelenchoides	Parasitus	**-1.496**	**0.859**	**4.616**	**2.578**
Aphelenchoides	Uropoda	**-1.496**	**0.481**	**4.616**	**1.976**
Aphelenchoides	Dorylaimoidea	**-1.496**	**-0.604**	**4.616**	**4.139**
Aphelenchoides	Qudsianematidae	**-1.496**	**-0.207**	**4.616**	**3.838**
Aphelenchoides	Eupodes	**-1.496**	**0.005**	**4.616**	**2.754**
Aphelenchoides	Mesostigmata	**-1.496**	**-0.411**	**4.616**	**1.976**
Aphelenchoides	Scutacarus	**-1.496**	**-0.608**	**4.616**	**3.152**
Tylenchidae	Mononchidae	**-1.360**	**-0.827**	**4.741**	**3.838**
Tylenchidae	Mylonchulus	**-1.360**	**-0.005**	**4.741**	**4.139**
Tylenchidae	Alliphis	**-1.360**	**0.053**	**4.741**	**2.976**
Tylenchidae	Arctoseius	**-1.360**	**-0.152**	**4.741**	**1.976**
Tylenchidae	Dendrolaelaps	**-1.360**	**0.027**	**4.741**	**1.976**
Tylenchidae	Lysigamasus	**-1.360**	**0.407**	**4.741**	**3.318**
Tylenchidae	Macrocheles	**-1.360**	**0.761**	**4.741**	**1.976**
Tylenchidae	Parasitus	**-1.360**	**0.859**	**4.741**	**2.578**

Tylenchidae	Uropoda	**-1.360**	**0.481**	**4.741**	**1.976**
Tylenchidae	Dorylaimoidea	**-1.360**	**-0.604**	**4.741**	**4.139**
Tylenchidae	Qudsianematidae	**-1.360**	**-0.207**	**4.741**	**3.838**
Tylenchidae	Eupodes	**-1.360**	**0.005**	**4.741**	**2.754**
Tylenchidae	Mesostigmata	**-1.360**	**-0.411**	**4.741**	**1.976**
Tylenchidae	Scutacarus	**-1.360**	**-0.608**	**4.741**	**3.152**
Oppiella	Arctoseius	**-0.447**	**-0.152**	**1.976**	**1.976**
Oppiella	Dendrolaelaps	**-0.447**	**0.027**	**1.976**	**1.976**
Oppiella	Lysigamasus	**-0.447**	**0.407**	**1.976**	**3.318**
Oppiella	Macrocheles	**-0.447**	**0.761**	**1.976**	**1.976**
Oppiella	Parasitus	**-0.447**	**0.859**	**1.976**	**2.578**
Oppiella	Uropoda	**-0.447**	**0.481**	**1.976**	**1.976**
Oppiella	Dorylaimoidea	**-0.447**	**-0.604**	**1.976**	**4.139**
Oppiella	Qudsianematidae	**-0.447**	**-0.207**	**1.976**	**3.838**
Oppiella	Eupodes	**-0.447**	**0.005**	**1.976**	**2.754**
Oppiella	Mesostigmata	**-0.447**	**-0.411**	**1.976**	**1.976**
Oppiella	Scutacarus	**-0.447**	**-0.608**	**1.976**	**3.152**
Pygmephorus	Arctoseius	**-0.376**	**-0.152**	**2.821**	**1.976**
Pygmephorus	Dendrolaelaps	**-0.376**	**0.027**	**2.821**	**1.976**
Pygmephorus	Lysigamasus	**-0.376**	**0.407**	**2.821**	**3.318**
Pygmephorus	Macrocheles	**-0.376**	**0.761**	**2.821**	**1.976**
Pygmephorus	Parasitus	**-0.376**	**0.859**	**2.821**	**2.578**
Pygmephorus	Uropoda	**-0.376**	**0.481**	**2.821**	**1.976**
Pygmephorus	Dorylaimoidea	**-0.376**	**-0.604**	**2.821**	**4.139**
Pygmephorus	Qudsianematidae	**-0.376**	**-0.207**	**2.821**	**3.838**
Pygmephorus	Eupodes	**-0.376**	**0.005**	**2.821**	**2.754**
Pygmephorus	Mesostigmata	**-0.376**	**-0.411**	**2.821**	**1.976**
Pygmephorus	Scutacarus	**-0.376**	**-0.608**	**2.821**	**3.152**
Brachystomella	Arctoseius	**0.977**	**-0.152**	**2.277**	**1.976**
Brachystomella	Dendrolaelaps	**0.977**	**0.027**	**2.277**	**1.976**
Brachystomella	Lysigamasus	**0.977**	**0.407**	**2.277**	**3.318**
Brachystomella	Macrocheles	**0.977**	**0.761**	**2.277**	**1.976**
Brachystomella	Parasitus	**0.977**	**0.859**	**2.277**	**2.578**
Brachystomella	Uropoda	**0.977**	**0.481**	**2.277**	**1.976**
Brachystomella	Dorylaimoidea	**0.977**	**-0.604**	**2.277**	**4.139**
Brachystomella	Qudsianematidae	**0.977**	**-0.207**	**2.277**	**3.838**
Brachystomella	Eupodes	**0.977**	**0.005**	**2.277**	**2.754**
Brachystomella	Mesostigmata	**0.977**	**-0.411**	**2.277**	**1.976**
Brachystomella	Scutacarus	**0.977**	**-0.608**	**2.277**	**3.152**
Friesea	Arctoseius	**0.434**	**-0.152**	**1.976**	**1.976**
Friesea	Dendrolaelaps	**0.434**	**0.027**	**1.976**	**1.976**
Friesea	Lysigamasus	**0.434**	**0.407**	**1.976**	**3.318**
Friesea	Macrocheles	**0.434**	**0.761**	**1.976**	**1.976**
Friesea	Parasitus	**0.434**	**0.859**	**1.976**	**2.578**
Friesea	Uropoda	**0.434**	**0.481**	**1.976**	**1.976**
Friesea	Dorylaimoidea	**0.434**	**-0.604**	**1.976**	**4.139**
Friesea	Qudsianematidae	**0.434**	**-0.207**	**1.976**	**3.838**
Friesea	Eupodes	**0.434**	**0.005**	**1.976**	**2.754**
Friesea	Mesostigmata	**0.434**	**-0.411**	**1.976**	**1.976**
Friesea	Scutacarus	**0.434**	**-0.608**	**1.976**	**3.152**
Isotoma	Arctoseius	**1.898**	**-0.152**	**3.231**	**1.976**

Isotoma	Dendrolaelaps	**1.898**	**0.027**	**3.231**	**1.976**
Isotoma	Lysigamasus	**1.898**	**0.407**	**3.231**	**3.318**
Isotoma	Macrocheles	**1.898**	**0.761**	**3.231**	**1.976**
Isotoma	Parasitus	**1.898**	**0.859**	**3.231**	**2.578**
Isotoma	Uropoda	**1.898**	**0.481**	**3.231**	**1.976**
Isotoma	Dorylaimoidea	**1.898**	**-0.604**	**3.231**	**4.139**
Isotoma	Qudsianematidae	**1.898**	**-0.207**	**3.231**	**3.838**
Isotoma	Eupodes	**1.898**	**0.005**	**3.231**	**2.754**
Isotoma	Mesostigmata	**1.898**	**-0.411**	**3.231**	**1.976**
Isotoma	Scutacarus	**1.898**	**-0.608**	**3.231**	**3.152**
Isotomiella	Arctoseius	**0.816**	**-0.152**	**1.976**	**1.976**
Isotomiella	Dendrolaelaps	**0.816**	**0.027**	**1.976**	**1.976**
Isotomiella	Lysigamasus	**0.816**	**0.407**	**1.976**	**3.318**
Isotomiella	Macrocheles	**0.816**	**0.761**	**1.976**	**1.976**
Isotomiella	Parasitus	**0.816**	**0.859**	**1.976**	**2.578**
Isotomiella	Uropoda	**0.816**	**0.481**	**1.976**	**1.976**
Isotomiella	Dorylaimoidea	**0.816**	**-0.604**	**1.976**	**4.139**
Isotomiella	Qudsianematidae	**0.816**	**-0.207**	**1.976**	**3.838**
Isotomiella	Eupodes	**0.816**	**0.005**	**1.976**	**2.754**
Isotomiella	Mesostigmata	**0.816**	**-0.411**	**1.976**	**1.976**
Isotomiella	Scutacarus	**0.816**	**-0.608**	**1.976**	**3.152**
Isotomurus	Arctoseius	**1.787**	**-0.152**	**2.930**	**1.976**
Isotomurus	Dendrolaelaps	**1.787**	**0.027**	**2.930**	**1.976**
Isotomurus	Lysigamasus	**1.787**	**0.407**	**2.930**	**3.318**
Isotomurus	Macrocheles	**1.787**	**0.761**	**2.930**	**1.976**
Isotomurus	Parasitus	**1.787**	**0.859**	**2.930**	**2.578**
Isotomurus	Uropoda	**1.787**	**0.481**	**2.930**	**1.976**
Isotomurus	Dorylaimoidea	**1.787**	**-0.604**	**2.930**	**4.139**
Isotomurus	Qudsianematidae	**1.787**	**-0.207**	**2.930**	**3.838**
Isotomurus	Eupodes	**1.787**	**0.005**	**2.930**	**2.754**
Isotomurus	Mesostigmata	**1.787**	**-0.411**	**2.930**	**1.976**
Isotomurus	Scutacarus	**1.787**	**-0.608**	**2.930**	**3.152**
Lepidocyrtus	Arctoseius	**1.231**	**-0.152**	**2.930**	**1.976**
Lepidocyrtus	Dendrolaelaps	**1.231**	**0.027**	**2.930**	**1.976**
Lepidocyrtus	Lysigamasus	**1.231**	**0.407**	**2.930**	**3.318**
Lepidocyrtus	Macrocheles	**1.231**	**0.761**	**2.930**	**1.976**
Lepidocyrtus	Parasitus	**1.231**	**0.859**	**2.930**	**2.578**
Lepidocyrtus	Uropoda	**1.231**	**0.481**	**2.930**	**1.976**
Lepidocyrtus	Dorylaimoidea	**1.231**	**-0.604**	**2.930**	**4.139**
Lepidocyrtus	Qudsianematidae	**1.231**	**-0.207**	**2.930**	**3.838**
Lepidocyrtus	Eupodes	**1.231**	**0.005**	**2.930**	**2.754**
Lepidocyrtus	Mesostigmata	**1.231**	**-0.411**	**2.930**	**1.976**
Lepidocyrtus	Scutacarus	**1.231**	**-0.608**	**2.930**	**3.152**
Mesaphorura	Arctoseius	**0.618**	**-0.152**	**2.277**	**1.976**
Mesaphorura	Dendrolaelaps	**0.618**	**0.027**	**2.277**	**1.976**
Mesaphorura	Lysigamasus	**0.618**	**0.407**	**2.277**	**3.318**
Mesaphorura	Macrocheles	**0.618**	**0.761**	**2.277**	**1.976**
Mesaphorura	Parasitus	**0.618**	**0.859**	**2.277**	**2.578**
Mesaphorura	Uropoda	**0.618**	**0.481**	**2.277**	**1.976**
Mesaphorura	Dorylaimoidea	**0.618**	**-0.604**	**2.277**	**4.139**
Mesaphorura	Qudsianematidae	**0.618**	**-0.207**	**2.277**	**3.838**

Mesaphorura	Eupodes	**0.618**	**0.005**	**2.277**	**2.754**
Mesaphorura	Mesostigmata	**0.618**	**-0.411**	**2.277**	**1.976**
Mesaphorura	Scutacarus	**0.618**	**-0.608**	**2.277**	**3.152**
Parisotoma	Arctoseius	**0.722**	**-0.152**	**1.976**	**1.976**
Parisotoma	Dendrolaelaps	**0.722**	**0.027**	**1.976**	**1.976**
Parisotoma	Lysigamasus	**0.722**	**0.407**	**1.976**	**3.318**
Parisotoma	Macrocheles	**0.722**	**0.761**	**1.976**	**1.976**
Parisotoma	Parasitus	**0.722**	**0.859**	**1.976**	**2.578**
Parisotoma	Uropoda	**0.722**	**0.481**	**1.976**	**1.976**
Parisotoma	Dorylaimoidea	**0.722**	**-0.604**	**1.976**	**4.139**
Parisotoma	Qudsianematidae	**0.722**	**-0.207**	**1.976**	**3.838**
Parisotoma	Eupodes	**0.722**	**0.005**	**1.976**	**2.754**
Parisotoma	Mesostigmata	**0.722**	**-0.411**	**1.976**	**1.976**
Parisotoma	Scutacarus	**0.722**	**-0.608**	**1.976**	**3.152**
Proisotoma	Arctoseius	**0.770**	**-0.152**	**3.055**	**1.976**
Proisotoma	Dendrolaelaps	**0.770**	**0.027**	**3.055**	**1.976**
Proisotoma	Lysigamasus	**0.770**	**0.407**	**3.055**	**3.318**
Proisotoma	Macrocheles	**0.770**	**0.761**	**3.055**	**1.976**
Proisotoma	Parasitus	**0.770**	**0.859**	**3.055**	**2.578**
Proisotoma	Uropoda	**0.770**	**0.481**	**3.055**	**1.976**
Proisotoma	Dorylaimoidea	**0.770**	**-0.604**	**3.055**	**4.139**
Proisotoma	Qudsianematidae	**0.770**	**-0.207**	**3.055**	**3.838**
Proisotoma	Eupodes	**0.770**	**0.005**	**3.055**	**2.754**
Proisotoma	Mesostigmata	**0.770**	**-0.411**	**3.055**	**1.976**
Proisotoma	Scutacarus	**0.770**	**-0.608**	**3.055**	**3.152**
Sminthurus	Arctoseius	**0.816**	**-0.152**	**2.277**	**1.976**
Sminthurus	Dendrolaelaps	**0.816**	**0.027**	**2.277**	**1.976**
Sminthurus	Lysigamasus	**0.816**	**0.407**	**2.277**	**3.318**
Sminthurus	Macrocheles	**0.816**	**0.761**	**2.277**	**1.976**
Sminthurus	Parasitus	**0.816**	**0.859**	**2.277**	**2.578**
Sminthurus	Uropoda	**0.816**	**0.481**	**2.277**	**1.976**
Sminthurus	Dorylaimoidea	**0.816**	**-0.604**	**2.277**	**4.139**
Sminthurus	Qudsianematidae	**0.816**	**-0.207**	**2.277**	**3.838**
Sminthurus	Eupodes	**0.816**	**0.005**	**2.277**	**2.754**
Sminthurus	Mesostigmata	**0.816**	**-0.411**	**2.277**	**1.976**
Sminthurus	Scutacarus	**0.816**	**-0.608**	**2.277**	**3.152**
Fridericia	Arctoseius	**1.980**	**-0.152**	**4.027**	**1.976**
Fridericia	Dendrolaelaps	**1.980**	**0.027**	**4.027**	**1.976**
Fridericia	Lysigamasus	**1.980**	**0.407**	**4.027**	**3.318**
Fridericia	Macrocheles	**1.980**	**0.761**	**4.027**	**1.976**
Fridericia	Parasitus	**1.980**	**0.859**	**4.027**	**2.578**
Fridericia	Uropoda	**1.980**	**0.481**	**4.027**	**1.976**
Fridericia	Dorylaimoidea	**1.980**	**-0.604**	**4.027**	**4.139**
Fridericia	Qudsianematidae	**1.980**	**-0.207**	**4.027**	**3.838**
Fridericia	Eupodes	**1.980**	**0.005**	**4.027**	**2.754**
Fridericia	Mesostigmata	**1.980**	**-0.411**	**4.027**	**1.976**
Fridericia	Scutacarus	**1.980**	**-0.608**	**4.027**	**3.152**
Acrobeloides	Mononchidae	**-1.171**	**-0.827**	**4.315**	**3.838**
Acrobeloides	Mylonchulus	**-1.171**	**-0.005**	**4.315**	**4.139**
Acrobeloides	Alliphis	**-1.171**	**0.053**	**4.315**	**2.976**
Acrobeloides	Arctoseius	**-1.171**	**-0.152**	**4.315**	**1.976**

Acrobeloides	Dendrolaelaps	**-1.171**	**0.027**	**4.315**	**1.976**
Acrobeloides	Lysigamasus	**-1.171**	**0.407**	**4.315**	**3.318**
Acrobeloides	Macrocheles	**-1.171**	**0.761**	**4.315**	**1.976**
Acrobeloides	Parasitus	**-1.171**	**0.859**	**4.315**	**2.578**
Acrobeloides	Uropoda	**-1.171**	**0.481**	**4.315**	**1.976**
Acrobeloides	Dorylaimoidea	**-1.171**	**-0.604**	**4.315**	**4.139**
Acrobeloides	Qudsianematidae	**-1.171**	**-0.207**	**4.315**	**3.838**
Acrobeloides	Eupodes	**-1.171**	**0.005**	**4.315**	**2.754**
Acrobeloides	Mesostigmata	**-1.171**	**-0.411**	**4.315**	**1.976**
Acrobeloides	Scutacarus	**-1.171**	**-0.608**	**4.315**	**3.152**
Cephalobidae	Mononchidae	**-1.055**	**-0.827**	**4.792**	**3.838**
Cephalobidae	Mylonchulus	**-1.055**	**-0.005**	**4.792**	**4.139**
Cephalobidae	Alliphis	**-1.055**	**0.053**	**4.792**	**2.976**
Cephalobidae	Arctoseius	**-1.055**	**-0.152**	**4.792**	**1.976**
Cephalobidae	Dendrolaelaps	**-1.055**	**0.027**	**4.792**	**1.976**
Cephalobidae	Lysigamasus	**-1.055**	**0.407**	**4.792**	**3.318**
Cephalobidae	Macrocheles	**-1.055**	**0.761**	**4.792**	**1.976**
Cephalobidae	Parasitus	**-1.055**	**0.859**	**4.792**	**2.578**
Cephalobidae	Uropoda	**-1.055**	**0.481**	**4.792**	**1.976**
Cephalobidae	Dorylaimoidea	**-1.055**	**-0.604**	**4.792**	**4.139**
Cephalobidae	Qudsianematidae	**-1.055**	**-0.207**	**4.792**	**3.838**
Cephalobidae	Eupodes	**-1.055**	**0.005**	**4.792**	**2.754**
Cephalobidae	Mesostigmata	**-1.055**	**-0.411**	**4.792**	**1.976**
Cephalobidae	Scutacarus	**-1.055**	**-0.608**	**4.792**	**3.152**
Eucephalobus	Mononchidae	**-0.855**	**-0.827**	**4.683**	**3.838**
Eucephalobus	Mylonchulus	**-0.855**	**-0.005**	**4.683**	**4.139**
Eucephalobus	Alliphis	**-0.855**	**0.053**	**4.683**	**2.976**
Eucephalobus	Arctoseius	**-0.855**	**-0.152**	**4.683**	**1.976**
Eucephalobus	Dendrolaelaps	**-0.855**	**0.027**	**4.683**	**1.976**
Eucephalobus	Lysigamasus	**-0.855**	**0.407**	**4.683**	**3.318**
Eucephalobus	Macrocheles	**-0.855**	**0.761**	**4.683**	**1.976**
Eucephalobus	Parasitus	**-0.855**	**0.859**	**4.683**	**2.578**
Eucephalobus	Uropoda	**-0.855**	**0.481**	**4.683**	**1.976**
Eucephalobus	Dorylaimoidea	**-0.855**	**-0.604**	**4.683**	**4.139**
Eucephalobus	Qudsianematidae	**-0.855**	**-0.207**	**4.683**	**3.838**
Eucephalobus	Eupodes	**-0.855**	**0.005**	**4.683**	**2.754**
Eucephalobus	Mesostigmata	**-0.855**	**-0.411**	**4.683**	**1.976**
Eucephalobus	Scutacarus	**-0.855**	**-0.608**	**4.683**	**3.152**
Panagrolaimus	Mononchidae	**-0.945**	**-0.827**	**3.838**	**3.838**
Panagrolaimus	Mylonchulus	**-0.945**	**-0.005**	**3.838**	**4.139**
Panagrolaimus	Alliphis	**-0.945**	**0.053**	**3.838**	**2.976**
Panagrolaimus	Arctoseius	**-0.945**	**-0.152**	**3.838**	**1.976**
Panagrolaimus	Dendrolaelaps	**-0.945**	**0.027**	**3.838**	**1.976**
Panagrolaimus	Lysigamasus	**-0.945**	**0.407**	**3.838**	**3.318**
Panagrolaimus	Macrocheles	**-0.945**	**0.761**	**3.838**	**1.976**
Panagrolaimus	Parasitus	**-0.945**	**0.859**	**3.838**	**2.578**
Panagrolaimus	Uropoda	**-0.945**	**0.481**	**3.838**	**1.976**
Panagrolaimus	Dorylaimoidea	**-0.945**	**-0.604**	**3.838**	**4.139**
Panagrolaimus	Qudsianematidae	**-0.945**	**-0.207**	**3.838**	**3.838**
Panagrolaimus	Eupodes	**-0.945**	**0.005**	**3.838**	**2.754**
Panagrolaimus	Mesostigmata	**-0.945**	**-0.411**	**3.838**	**1.976**

Panagrolaimus	Scutacarus	**-0.945**	**-0.608**	**3.838**	**3.152**
Plectus	Mononchidae	**-0.583**	**-0.827**	**3.838**	**3.838**
Plectus	Mylonchulus	**-0.583**	**-0.005**	**3.838**	**4.139**
Plectus	Alliphis	**-0.583**	**0.053**	**3.838**	**2.976**
Plectus	Arctoseius	**-0.583**	**-0.152**	**3.838**	**1.976**
Plectus	Dendrolaelaps	**-0.583**	**0.027**	**3.838**	**1.976**
Plectus	Lysigamasus	**-0.583**	**0.407**	**3.838**	**3.318**
Plectus	Macrocheles	**-0.583**	**0.761**	**3.838**	**1.976**
Plectus	Parasitus	**-0.583**	**0.859**	**3.838**	**2.578**
Plectus	Uropoda	**-0.583**	**0.481**	**3.838**	**1.976**
Plectus	Dorylaimoidea	**-0.583**	**-0.604**	**3.838**	**4.139**
Plectus	Qudsianematidae	**-0.583**	**-0.207**	**3.838**	**3.838**
Plectus	Eupodes	**-0.583**	**0.005**	**3.838**	**2.754**
Plectus	Mesostigmata	**-0.583**	**-0.411**	**3.838**	**1.976**
Plectus	Scutacarus	**-0.583**	**-0.608**	**3.838**	**3.152**
Rhabditidae	Mononchidae	**-0.692**	**-0.827**	**5.736**	**3.838**
Rhabditidae	Mylonchulus	**-0.692**	**-0.005**	**5.736**	**4.139**
Rhabditidae	Alliphis	**-0.692**	**0.053**	**5.736**	**2.976**
Rhabditidae	Arctoseius	**-0.692**	**-0.152**	**5.736**	**1.976**
Rhabditidae	Dendrolaelaps	**-0.692**	**0.027**	**5.736**	**1.976**
Rhabditidae	Lysigamasus	**-0.692**	**0.407**	**5.736**	**3.318**
Rhabditidae	Macrocheles	**-0.692**	**0.761**	**5.736**	**1.976**
Rhabditidae	Parasitus	**-0.692**	**0.859**	**5.736**	**2.578**
Rhabditidae	Uropoda	**-0.692**	**0.481**	**5.736**	**1.976**
Rhabditidae	Dorylaimoidea	**-0.692**	**-0.604**	**5.736**	**4.139**
Rhabditidae	Qudsianematidae	**-0.692**	**-0.207**	**5.736**	**3.838**
Rhabditidae	Eupodes	**-0.692**	**0.005**	**5.736**	**2.754**
Rhabditidae	Mesostigmata	**-0.692**	**-0.411**	**5.736**	**1.976**
Rhabditidae	Scutacarus	**-0.692**	**-0.608**	**5.736**	**3.152**
Histiostoma	Arctoseius	**-0.805**	**-0.152**	**1.976**	**1.976**
Histiostoma	Dendrolaelaps	**-0.805**	**0.027**	**1.976**	**1.976**
Histiostoma	Lysigamasus	**-0.805**	**0.407**	**1.976**	**3.318**
Histiostoma	Macrocheles	**-0.805**	**0.761**	**1.976**	**1.976**
Histiostoma	Parasitus	**-0.805**	**0.859**	**1.976**	**2.578**
Histiostoma	Uropoda	**-0.805**	**0.481**	**1.976**	**1.976**
Histiostoma	Eupodes	**-0.805**	**0.005**	**1.976**	**2.754**
Histiostoma	Mesostigmata	**-0.805**	**-0.411**	**1.976**	**1.976**
Histiostoma	Scutacarus	**-0.805**	**-0.608**	**1.976**	**3.152**
Enchytraeus	Arctoseius	**1.190**	**-0.152**	**4.924**	**1.976**
Enchytraeus	Dendrolaelaps	**1.190**	**0.027**	**4.924**	**1.976**
Enchytraeus	Lysigamasus	**1.190**	**0.407**	**4.924**	**3.318**
Enchytraeus	Macrocheles	**1.190**	**0.761**	**4.924**	**1.976**
Enchytraeus	Parasitus	**1.190**	**0.859**	**4.924**	**2.578**
Enchytraeus	Uropoda	**1.190**	**0.481**	**4.924**	**1.976**
Enchytraeus	Dorylaimoidea	**1.190**	**-0.604**	**4.924**	**4.139**
Enchytraeus	Qudsianematidae	**1.190**	**-0.207**	**4.924**	**3.838**
Enchytraeus	Eupodes	**1.190**	**0.005**	**4.924**	**2.754**
Enchytraeus	Mesostigmata	**1.190**	**-0.411**	**4.924**	**1.976**
Enchytraeus	Scutacarus	**1.190**	**-0.608**	**4.924**	**3.152**
Eubacteria	Acrobeloides	**-6.735**	**-1.171**	**13.228**	**4.315**
Eubacteria	Cephalobidae	**-6.735**	**-1.055**	**13.228**	**4.792**

Eubacteria	Eucephalobus	**-6.735**	**-0.855**	**13.228**	**4.683**
Eubacteria	Panagrolaimus	**-6.735**	**-0.945**	**13.228**	**3.838**
Eubacteria	Plectus	**-6.735**	**-0.583**	**13.228**	**3.838**
Eubacteria	Rhabditidae	**-6.735**	**-0.692**	**13.228**	**5.736**
Eubacteria	Histiostoma	**-6.735**	**-0.805**	**13.228**	**1.976**
Eubacteria	Enchytraeus	**-6.735**	**1.190**	**13.228**	**4.924**
Eubacteria	Dauerlarvae	**-6.735**	**-0.804**	**13.228**	**4.315**
Eubacteria	Buchholzia	**-6.735**	**1.882**	**13.228**	**3.365**
Eubacteria	Henlea	**-6.735**	**1.580**	**13.228**	**3.891**
Eubacteria	Marionina	**-6.735**	**0.887**	**13.228**	**3.663**
Dauerlarvae	Mononchidae	**-0.804**	**-0.827**	**4.315**	**3.838**
Dauerlarvae	Mylonchulus	**-0.804**	**-0.005**	**4.315**	**4.139**
Dauerlarvae	Alliphis	**-0.804**	**0.053**	**4.315**	**2.976**
Dauerlarvae	Dorylaimoidea	**-0.804**	**-0.604**	**4.315**	**4.139**
Dauerlarvae	Qudsianematidae	**-0.804**	**-0.207**	**4.315**	**3.838**
Dauerlarvae	Eupodes	**-0.804**	**0.005**	**4.315**	**2.754**
Dauerlarvae	Mesostigmata	**-0.804**	**-0.411**	**4.315**	**1.976**
Dauerlarvae	Scutacarus	**-0.804**	**-0.608**	**4.315**	**3.152**
Buchholzia	Arctoseius	**1.882**	**-0.152**	**3.365**	**1.976**
Buchholzia	Dendrolaelaps	**1.882**	**0.027**	**3.365**	**1.976**
Buchholzia	Lysigamasus	**1.882**	**0.407**	**3.365**	**3.318**
Buchholzia	Macrocheles	**1.882**	**0.761**	**3.365**	**1.976**
Buchholzia	Parasitus	**1.882**	**0.859**	**3.365**	**2.578**
Buchholzia	Uropoda	**1.882**	**0.481**	**3.365**	**1.976**
Buchholzia	Dorylaimoidea	**1.882**	**-0.604**	**3.365**	**4.139**
Buchholzia	Qudsianematidae	**1.882**	**-0.207**	**3.365**	**3.838**
Buchholzia	Eupodes	**1.882**	**0.005**	**3.365**	**2.754**
Buchholzia	Mesostigmata	**1.882**	**-0.411**	**3.365**	**1.976**
Buchholzia	Scutacarus	**1.882**	**-0.608**	**3.365**	**3.152**
Henlea	Arctoseius	**1.580**	**-0.152**	**3.891**	**1.976**
Henlea	Dendrolaelaps	**1.580**	**0.027**	**3.891**	**1.976**
Henlea	Lysigamasus	**1.580**	**0.407**	**3.891**	**3.318**
Henlea	Macrocheles	**1.580**	**0.761**	**3.891**	**1.976**
Henlea	Parasitus	**1.580**	**0.859**	**3.891**	**2.578**
Henlea	Uropoda	**1.580**	**0.481**	**3.891**	**1.976**
Henlea	Dorylaimoidea	**1.580**	**-0.604**	**3.891**	**4.139**
Henlea	Qudsianematidae	**1.580**	**-0.207**	**3.891**	**3.838**
Henlea	Eupodes	**1.580**	**0.005**	**3.891**	**2.754**
Henlea	Mesostigmata	**1.580**	**-0.411**	**3.891**	**1.976**
Henlea	Scutacarus	**1.580**	**-0.608**	**3.891**	**3.152**
Marionina	Arctoseius	**0.887**	**-0.152**	**3.663**	**1.976**
Marionina	Dendrolaelaps	**0.887**	**0.027**	**3.663**	**1.976**
Marionina	Lysigamasus	**0.887**	**0.407**	**3.663**	**3.318**
Marionina	Macrocheles	**0.887**	**0.761**	**3.663**	**1.976**
Marionina	Parasitus	**0.887**	**0.859**	**3.663**	**2.578**
Marionina	Uropoda	**0.887**	**0.481**	**3.663**	**1.976**
Marionina	Dorylaimoidea	**0.887**	**-0.604**	**3.663**	**4.139**
Marionina	Qudsianematidae	**0.887**	**-0.207**	**3.663**	**3.838**
Marionina	Eupodes	**0.887**	**0.005**	**3.663**	**2.754**
Marionina	Mesostigmata	**0.887**	**-0.411**	**3.663**	**1.976**
Marionina	Scutacarus	**0.887**	**-0.608**	**3.663**	**3.152**

Hyphae and hair roots	Coslenchus	6.607	-0.821	0.000	3.838
Hyphae and hair roots	Dolichodoridae	6.607	-0.885	0.000	4.139
Hyphae and hair roots	Helicotylenchus	6.607	-0.792	0.000	3.838
Hyphae and hair roots	Heterodera	6.607	-0.883	0.000	3.838
Hyphae and hair roots	Malenchus	6.607	-1.330	0.000	4.139
Hyphae and hair roots	Meloidogyne	6.607	-1.287	0.000	4.537
Hyphae and hair roots	Paratylenchus	6.607	-1.244	0.000	4.440
Hyphae and hair roots	Pratylenchus	6.607	-1.226	0.000	3.838
Hyphae and hair roots	Tylenchorhynchus	6.607	-0.664	0.000	4.139
Hyphae and hair roots	Tydeidae	6.607	-0.608	0.000	2.976
Hyphae and hair roots	Sminthuridae	6.607	-0.608	0.000	2.277
Hyphae and hair roots	Sminthurinus	6.607	0.618	0.000	2.976
Hyphae and hair roots	Sminthurus	6.607	1.429	0.000	2.453
Hyphae and hair roots	Sphaeridia	6.607	0.202	0.000	2.277
Hyphae and hair roots	Aphelenchoides	6.607	-1.496	0.000	4.616
Hyphae and hair roots	Tylenchidae	6.607	-1.360	0.000	4.741
Hyphae and hair roots	Oppiella	6.607	-0.447	0.000	1.976
Hyphae and hair roots	Pygmephorus	6.607	-0.376	0.000	2.821
Hyphae and hair roots	Brachystomella	6.607	0.977	0.000	2.277
Hyphae and hair roots	Friesea	6.607	0.434	0.000	1.976
Hyphae and hair roots	Isotoma	6.607	1.898	0.000	3.231
Hyphae and hair roots	Isotomiella	6.607	0.816	0.000	1.976
Hyphae and hair roots	Isotomurus	6.607	1.787	0.000	2.930
Hyphae and hair roots	Lepidocyrtus	6.607	1.231	0.000	2.930
Hyphae and hair roots	Mesaphorura	6.607	0.618	0.000	2.277
Hyphae and hair roots	Parisotoma	6.607	0.722	0.000	1.976
Hyphae and hair roots	Proisotoma	6.607	0.770	0.000	3.055
Hyphae and hair roots	Sminthurus	6.607	0.816	0.000	2.277
Hyphae and hair roots	Fridericia	6.607	1.980	0.000	4.027
Hyphae and hair roots	Dorylaimoidea	6.607	-0.604	0.000	4.139
Hyphae and hair roots	Qudsianematidae	6.607	-0.207	0.000	3.838
Hyphae and hair roots	Eupodes	6.607	0.005	0.000	2.754
Hyphae and hair roots	Mesostigmata	6.607	-0.411	0.000	1.976
Hyphae and hair roots	Scutacarus	6.607	-0.608	0.000	3.152
Mononchidae	Arctoseius	-0.827	-0.152	3.838	1.976
Mononchidae	Dendrolaelaps	-0.827	0.027	3.838	1.976
Mononchidae	Lysigamasus	-0.827	0.407	3.838	3.318
Mononchidae	Macrocheles	-0.827	0.761	3.838	1.976
Mononchidae	Parasitus	-0.827	0.859	3.838	2.578
Mononchidae	Uropoda	-0.827	0.481	3.838	1.976
Mononchidae	Dorylaimoidea	-0.827	-0.604	3.838	4.139
Mononchidae	Qudsianematidae	-0.827	-0.207	3.838	3.838
Mononchidae	Eupodes	-0.827	0.005	3.838	2.754
Mononchidae	Mesostigmata	-0.827	-0.411	3.838	1.976
Mononchidae	Scutacarus	-0.827	-0.608	3.838	3.152
Mylonchulus	Arctoseius	-0.005	-0.152	4.139	1.976
Mylonchulus	Dendrolaelaps	-0.005	0.027	4.139	1.976
Mylonchulus	Lysigamasus	-0.005	0.407	4.139	3.318
Mylonchulus	Macrocheles	-0.005	0.761	4.139	1.976
Mylonchulus	Parasitus	-0.005	0.859	4.139	2.578
Mylonchulus	Uropoda	-0.005	0.481	4.139	1.976

Mylonchulus	Dorylaimoidea	**-0.005**	**-0.604**	**4.139**	**4.139**
Mylonchulus	Qudsianematidae	**-0.005**	**-0.207**	**4.139**	**3.838**
Mylonchulus	Eupodes	**-0.005**	**0.005**	**4.139**	**2.754**
Mylonchulus	Mesostigmata	**-0.005**	**-0.411**	**4.139**	**1.976**
Mylonchulus	Scutacarus	**-0.005**	**-0.608**	**4.139**	**3.152**
Alliphis	Arctoseius	**0.053**	**-0.152**	**2.976**	**1.976**
Alliphis	Dendrolaelaps	**0.053**	**0.027**	**2.976**	**1.976**
Alliphis	Lysigamasus	**0.053**	**0.407**	**2.976**	**3.318**
Alliphis	Macrocheles	**0.053**	**0.761**	**2.976**	**1.976**
Alliphis	Parasitus	**0.053**	**0.859**	**2.976**	**2.578**
Alliphis	Uropoda	**0.053**	**0.481**	**2.976**	**1.976**
Alliphis	Dorylaimoidea	**0.053**	**-0.604**	**2.976**	**4.139**
Alliphis	Qudsianematidae	**0.053**	**-0.207**	**2.976**	**3.838**
Alliphis	Eupodes	**0.053**	**0.005**	**2.976**	**2.754**
Alliphis	Mesostigmata	**0.053**	**-0.411**	**2.976**	**1.976**
Alliphis	Scutacarus	**0.053**	**-0.608**	**2.976**	**3.152**
Arctoseius	Dorylaimoidea	**-0.152**	**-0.604**	**1.976**	**4.139**
Arctoseius	Qudsianematidae	**-0.152**	**-0.207**	**1.976**	**3.838**
Arctoseius	Eupodes	**-0.152**	**0.005**	**1.976**	**2.754**
Arctoseius	Mesostigmata	**-0.152**	**-0.411**	**1.976**	**1.976**
Arctoseius	Scutacarus	**-0.152**	**-0.608**	**1.976**	**3.152**
Dendrolaelaps	Dorylaimoidea	**0.027**	**-0.604**	**1.976**	**4.139**
Dendrolaelaps	Qudsianematidae	**0.027**	**-0.207**	**1.976**	**3.838**
Dendrolaelaps	Eupodes	**0.027**	**0.005**	**1.976**	**2.754**
Dendrolaelaps	Mesostigmata	**0.027**	**-0.411**	**1.976**	**1.976**
Dendrolaelaps	Scutacarus	**0.027**	**-0.608**	**1.976**	**3.152**
Lysigamasus	Dorylaimoidea	**0.407**	**-0.604**	**3.318**	**4.139**
Lysigamasus	Qudsianematidae	**0.407**	**-0.207**	**3.318**	**3.838**
Lysigamasus	Eupodes	**0.407**	**0.005**	**3.318**	**2.754**
Lysigamasus	Mesostigmata	**0.407**	**-0.411**	**3.318**	**1.976**
Lysigamasus	Scutacarus	**0.407**	**-0.608**	**3.318**	**3.152**
Macrocheles	Dorylaimoidea	**0.761**	**-0.604**	**1.976**	**4.139**
Macrocheles	Qudsianematidae	**0.761**	**-0.207**	**1.976**	**3.838**
Macrocheles	Eupodes	**0.761**	**0.005**	**1.976**	**2.754**
Macrocheles	Mesostigmata	**0.761**	**-0.411**	**1.976**	**1.976**
Macrocheles	Scutacarus	**0.761**	**-0.608**	**1.976**	**3.152**
Parasitus	Dorylaimoidea	**0.859**	**-0.604**	**2.578**	**4.139**
Parasitus	Qudsianematidae	**0.859**	**-0.207**	**2.578**	**3.838**
Parasitus	Eupodes	**0.859**	**0.005**	**2.578**	**2.754**
Parasitus	Mesostigmata	**0.859**	**-0.411**	**2.578**	**1.976**
Parasitus	Scutacarus	**0.859**	**-0.608**	**2.578**	**3.152**
Uropoda	Dorylaimoidea	**0.481**	**-0.604**	**1.976**	**4.139**
Uropoda	Qudsianematidae	**0.481**	**-0.207**	**1.976**	**3.838**
Uropoda	Eupodes	**0.481**	**0.005**	**1.976**	**2.754**
Uropoda	Mesostigmata	**0.481**	**-0.411**	**1.976**	**1.976**
Uropoda	Scutacarus	**0.481**	**-0.608**	**1.976**	**3.152**
Dorylaimoidea	Mononchidae	**-0.604**	**-0.827**	**4.139**	**3.838**
Dorylaimoidea	Mylonchulus	**-0.604**	**-0.005**	**4.139**	**4.139**
Dorylaimoidea	Alliphis	**-0.604**	**0.053**	**4.139**	**2.976**
Dorylaimoidea	Arctoseius	**-0.604**	**-0.152**	**4.139**	**1.976**
Dorylaimoidea	Dendrolaelaps	**-0.604**	**0.027**	**4.139**	**1.976**

Dorylaimoidea	Lysigamasus	**-0.604**	**0.407**	**4.139**	**3.318**
Dorylaimoidea	Macrocheles	**-0.604**	**0.761**	**4.139**	**1.976**
Dorylaimoidea	Parasitus	**-0.604**	**0.859**	**4.139**	**2.578**
Dorylaimoidea	Uropoda	**-0.604**	**0.481**	**4.139**	**1.976**
Dorylaimoidea	Dorylaimoidea	**-0.604**	**-0.604**	**4.139**	**4.139**
Dorylaimoidea	Qudsianematidae	**-0.604**	**-0.207**	**4.139**	**3.838**
Dorylaimoidea	Eupodes	**-0.604**	**0.005**	**4.139**	**2.754**
Dorylaimoidea	Mesostigmata	**-0.604**	**-0.411**	**4.139**	**1.976**
Dorylaimoidea	Scutacarus	**-0.604**	**-0.608**	**4.139**	**3.152**
Qudsianematidae	Mononchidae	**-0.207**	**-0.827**	**3.838**	**3.838**
Qudsianematidae	Mylonchulus	**-0.207**	**-0.005**	**3.838**	**4.139**
Qudsianematidae	Alliphis	**-0.207**	**0.053**	**3.838**	**2.976**
Qudsianematidae	Arctoseius	**-0.207**	**-0.152**	**3.838**	**1.976**
Qudsianematidae	Dendrolaelaps	**-0.207**	**0.027**	**3.838**	**1.976**
Qudsianematidae	Lysigamasus	**-0.207**	**0.407**	**3.838**	**3.318**
Qudsianematidae	Macrocheles	**-0.207**	**0.761**	**3.838**	**1.976**
Qudsianematidae	Parasitus	**-0.207**	**0.859**	**3.838**	**2.578**
Qudsianematidae	Uropoda	**-0.207**	**0.481**	**3.838**	**1.976**
Qudsianematidae	Dorylaimoidea	**-0.207**	**-0.604**	**3.838**	**4.139**
Qudsianematidae	Qudsianematidae	**-0.207**	**-0.207**	**3.838**	**3.838**
Qudsianematidae	Eupodes	**-0.207**	**0.005**	**3.838**	**2.754**
Qudsianematidae	Mesostigmata	**-0.207**	**-0.411**	**3.838**	**1.976**
Qudsianematidae	Scutacarus	**-0.207**	**-0.608**	**3.838**	**3.152**
Eupodes	Arctoseius	**0.005**	**-0.152**	**2.754**	**1.976**
Eupodes	Dendrolaelaps	**0.005**	**0.027**	**2.754**	**1.976**
Eupodes	Lysigamasus	**0.005**	**0.407**	**2.754**	**3.318**
Eupodes	Macrocheles	**0.005**	**0.761**	**2.754**	**1.976**
Eupodes	Parasitus	**0.005**	**0.859**	**2.754**	**2.578**
Eupodes	Uropoda	**0.005**	**0.481**	**2.754**	**1.976**
Eupodes	Dorylaimoidea	**0.005**	**-0.604**	**2.754**	**4.139**
Eupodes	Qudsianematidae	**0.005**	**-0.207**	**2.754**	**3.838**
Eupodes	Eupodes	**0.005**	**0.005**	**2.754**	**2.754**
Eupodes	Mesostigmata	**0.005**	**-0.411**	**2.754**	**1.976**
Eupodes	Scutacarus	**0.005**	**-0.608**	**2.754**	**3.152**
Mesostigmata	Arctoseius	**-0.411**	**-0.152**	**1.976**	**1.976**
Mesostigmata	Dendrolaelaps	**-0.411**	**0.027**	**1.976**	**1.976**
Mesostigmata	Lysigamasus	**-0.411**	**0.407**	**1.976**	**3.318**
Mesostigmata	Macrocheles	**-0.411**	**0.761**	**1.976**	**1.976**
Mesostigmata	Parasitus	**-0.411**	**0.859**	**1.976**	**2.578**
Mesostigmata	Uropoda	**-0.411**	**0.481**	**1.976**	**1.976**
Mesostigmata	Dorylaimoidea	**-0.411**	**-0.604**	**1.976**	**4.139**
Mesostigmata	Qudsianematidae	**-0.411**	**-0.207**	**1.976**	**3.838**
Mesostigmata	Eupodes	**-0.411**	**0.005**	**1.976**	**2.754**
Mesostigmata	Mesostigmata	**-0.411**	**-0.411**	**1.976**	**1.976**
Mesostigmata	Scutacarus	**-0.411**	**-0.608**	**1.976**	**3.152**
Scutacarus	Arctoseius	**-0.608**	**-0.152**	**3.152**	**1.976**
Scutacarus	Dendrolaelaps	**-0.608**	**0.027**	**3.152**	**1.976**
Scutacarus	Lysigamasus	**-0.608**	**0.407**	**3.152**	**3.318**
Scutacarus	Macrocheles	**-0.608**	**0.761**	**3.152**	**1.976**
Scutacarus	Parasitus	**-0.608**	**0.859**	**3.152**	**2.578**
Scutacarus	Uropoda	**-0.608**	**0.481**	**3.152**	**1.976**

Scutacarus	Dorylaimoidea	**-0.608**	**-0.604**	**3.152**	**4.139**
Scutacarus	Qudsianematidae	**-0.608**	**-0.207**	**3.152**	**3.838**
Scutacarus	Eupodes	**-0.608**	**0.005**	**3.152**	**2.754**
Scutacarus	Mesostigmata	**-0.608**	**-0.411**	**3.152**	**1.976**
Scutacarus	Scutacarus	**-0.608**	**-0.608**	**3.152**	**3.152**

Resource	Consumer	Mres	Mconsumer	Nres	Nconsumer
Aglenchus	Mononchidae	**-1.053**	**-0.827**	**3.779**	**3.779**
Aglenchus	Alliphis	**-1.053**	**0.053**	**3.779**	**3.090**
Aglenchus	Cheiroseius	**-1.053**	**0.356**	**3.779**	**2.277**
Aglenchus	Dendrolaelaps	**-1.053**	**0.027**	**3.779**	**1.976**
Aglenchus	Lysigamasus	**-1.053**	**0.407**	**3.779**	**2.821**
Aglenchus	Pergamasus	**-1.053**	**1.081**	**3.779**	**2.277**
Aglenchus	Uropoda	**-1.053**	**0.481**	**3.779**	**1.976**
Aglenchus	Dorylaimoidea	**-1.053**	**-0.604**	**3.779**	**4.858**
Aglenchus	Eupodes	**-1.053**	**0.005**	**3.779**	**2.675**
Aglenchus	Mesostigmata	**-1.053**	**-0.411**	**3.779**	**1.976**
Aglenchus	Scutacarus	**-1.053**	**-0.608**	**3.779**	**3.391**
Aglenchus	Tarsonemus	**-1.053**	**-0.701**	**3.779**	**2.976**
Dolichodoridae	Mononchidae	**-0.885**	**-0.827**	**4.779**	**3.779**
Dolichodoridae	Alliphis	**-0.885**	**0.053**	**4.779**	**3.090**
Dolichodoridae	Cheiroseius	**-0.885**	**0.356**	**4.779**	**2.277**
Dolichodoridae	Dendrolaelaps	**-0.885**	**0.027**	**4.779**	**1.976**
Dolichodoridae	Lysigamasus	**-0.885**	**0.407**	**4.779**	**2.821**
Dolichodoridae	Pergamasus	**-0.885**	**1.081**	**4.779**	**2.277**
Dolichodoridae	Uropoda	**-0.885**	**0.481**	**4.779**	**1.976**
Dolichodoridae	Dorylaimoidea	**-0.885**	**-0.604**	**4.779**	**4.858**
Dolichodoridae	Eupodes	**-0.885**	**0.005**	**4.779**	**2.675**
Dolichodoridae	Mesostigmata	**-0.885**	**-0.411**	**4.779**	**1.976**
Dolichodoridae	Scutacarus	**-0.885**	**-0.608**	**4.779**	**3.391**
Dolichodoridae	Tarsonemus	**-0.885**	**-0.701**	**4.779**	**2.976**
Helicotylenchus	Mononchidae	**-0.792**	**-0.827**	**3.779**	**3.779**
Helicotylenchus	Alliphis	**-0.792**	**0.053**	**3.779**	**3.090**
Helicotylenchus	Cheiroseius	**-0.792**	**0.356**	**3.779**	**2.277**
Helicotylenchus	Dendrolaelaps	**-0.792**	**0.027**	**3.779**	**1.976**
Helicotylenchus	Lysigamasus	**-0.792**	**0.407**	**3.779**	**2.821**
Helicotylenchus	Pergamasus	**-0.792**	**1.081**	**3.779**	**2.277**
Helicotylenchus	Uropoda	**-0.792**	**0.481**	**3.779**	**1.976**
Helicotylenchus	Dorylaimoidea	**-0.792**	**-0.604**	**3.779**	**4.858**
Helicotylenchus	Eupodes	**-0.792**	**0.005**	**3.779**	**2.675**
Helicotylenchus	Mesostigmata	**-0.792**	**-0.411**	**3.779**	**1.976**
Helicotylenchus	Scutacarus	**-0.792**	**-0.608**	**3.779**	**3.391**
Helicotylenchus	Tarsonemus	**-0.792**	**-0.701**	**3.779**	**2.976**
Hoplolaimidae	Mononchidae	**-1.090**	**-0.827**	**3.779**	**3.779**
Hoplolaimidae	Alliphis	**-1.090**	**0.053**	**3.779**	**3.090**
Hoplolaimidae	Cheiroseius	**-1.090**	**0.356**	**3.779**	**2.277**
Hoplolaimidae	Dendrolaelaps	**-1.090**	**0.027**	**3.779**	**1.976**
Hoplolaimidae	Lysigamasus	**-1.090**	**0.407**	**3.779**	**2.821**
Hoplolaimidae	Pergamasus	**-1.090**	**1.081**	**3.779**	**2.277**
Hoplolaimidae	Uropoda	**-1.090**	**0.481**	**3.779**	**1.976**
Hoplolaimidae	Dorylaimoidea	**-1.090**	**-0.604**	**3.779**	**4.858**
Hoplolaimidae	Eupodes	**-1.090**	**0.005**	**3.779**	**2.675**
Hoplolaimidae	Mesostigmata	**-1.090**	**-0.411**	**3.779**	**1.976**
Hoplolaimidae	Scutacarus	**-1.090**	**-0.608**	**3.779**	**3.391**
Hoplolaimidae	Tarsonemus	**-1.090**	**-0.701**	**3.779**	**2.976**
Malenchus	Mononchidae	**-1.330**	**-0.827**	**4.478**	**3.779**
Malenchus	Alliphis	**-1.330**	**0.053**	**4.478**	**3.090**

Malenchus	Cheiroseius	**-1.330**	**0.356**	**4.478**	**2.277**
Malenchus	Dendrolaelaps	**-1.330**	**0.027**	**4.478**	**1.976**
Malenchus	Lysigamasus	**-1.330**	**0.407**	**4.478**	**2.821**
Malenchus	Pergamasus	**-1.330**	**1.081**	**4.478**	**2.277**
Malenchus	Uropoda	**-1.330**	**0.481**	**4.478**	**1.976**
Malenchus	Dorylaimoidea	**-1.330**	**-0.604**	**4.478**	**4.858**
Malenchus	Eupodes	**-1.330**	**0.005**	**4.478**	**2.675**
Malenchus	Mesostigmata	**-1.330**	**-0.411**	**4.478**	**1.976**
Malenchus	Scutacarus	**-1.330**	**-0.608**	**4.478**	**3.391**
Malenchus	Tarsonemus	**-1.330**	**-0.701**	**4.478**	**2.976**
Meloidogyne	Mononchidae	**-1.287**	**-0.827**	**3.779**	**3.779**
Meloidogyne	Alliphis	**-1.287**	**0.053**	**3.779**	**3.090**
Meloidogyne	Cheiroseius	**-1.287**	**0.356**	**3.779**	**2.277**
Meloidogyne	Dendrolaelaps	**-1.287**	**0.027**	**3.779**	**1.976**
Meloidogyne	Lysigamasus	**-1.287**	**0.407**	**3.779**	**2.821**
Meloidogyne	Pergamasus	**-1.287**	**1.081**	**3.779**	**2.277**
Meloidogyne	Uropoda	**-1.287**	**0.481**	**3.779**	**1.976**
Meloidogyne	Dorylaimoidea	**-1.287**	**-0.604**	**3.779**	**4.858**
Meloidogyne	Eupodes	**-1.287**	**0.005**	**3.779**	**2.675**
Meloidogyne	Mesostigmata	**-1.287**	**-0.411**	**3.779**	**1.976**
Meloidogyne	Scutacarus	**-1.287**	**-0.608**	**3.779**	**3.391**
Meloidogyne	Tarsonemus	**-1.287**	**-0.701**	**3.779**	**2.976**
Paratrichodorus	Mononchidae	**-0.630**	**-0.827**	**3.779**	**3.779**
Paratrichodorus	Alliphis	**-0.630**	**0.053**	**3.779**	**3.090**
Paratrichodorus	Cheiroseius	**-0.630**	**0.356**	**3.779**	**2.277**
Paratrichodorus	Dendrolaelaps	**-0.630**	**0.027**	**3.779**	**1.976**
Paratrichodorus	Lysigamasus	**-0.630**	**0.407**	**3.779**	**2.821**
Paratrichodorus	Pergamasus	**-0.630**	**1.081**	**3.779**	**2.277**
Paratrichodorus	Uropoda	**-0.630**	**0.481**	**3.779**	**1.976**
Paratrichodorus	Dorylaimoidea	**-0.630**	**-0.604**	**3.779**	**4.858**
Paratrichodorus	Eupodes	**-0.630**	**0.005**	**3.779**	**2.675**
Paratrichodorus	Mesostigmata	**-0.630**	**-0.411**	**3.779**	**1.976**
Paratrichodorus	Scutacarus	**-0.630**	**-0.608**	**3.779**	**3.391**
Paratrichodorus	Tarsonemus	**-0.630**	**-0.701**	**3.779**	**2.976**
Paratylenchus	Mononchidae	**-1.244**	**-0.827**	**4.080**	**3.779**
Paratylenchus	Alliphis	**-1.244**	**0.053**	**4.080**	**3.090**
Paratylenchus	Cheiroseius	**-1.244**	**0.356**	**4.080**	**2.277**
Paratylenchus	Dendrolaelaps	**-1.244**	**0.027**	**4.080**	**1.976**
Paratylenchus	Lysigamasus	**-1.244**	**0.407**	**4.080**	**2.821**
Paratylenchus	Pergamasus	**-1.244**	**1.081**	**4.080**	**2.277**
Paratylenchus	Uropoda	**-1.244**	**0.481**	**4.080**	**1.976**
Paratylenchus	Dorylaimoidea	**-1.244**	**-0.604**	**4.080**	**4.858**
Paratylenchus	Eupodes	**-1.244**	**0.005**	**4.080**	**2.675**
Paratylenchus	Mesostigmata	**-1.244**	**-0.411**	**4.080**	**1.976**
Paratylenchus	Scutacarus	**-1.244**	**-0.608**	**4.080**	**3.391**
Paratylenchus	Tarsonemus	**-1.244**	**-0.701**	**4.080**	**2.976**
Pratylenchus	Mononchidae	**-1.226**	**-0.827**	**4.624**	**3.779**
Pratylenchus	Alliphis	**-1.226**	**0.053**	**4.624**	**3.090**
Pratylenchus	Cheiroseius	**-1.226**	**0.356**	**4.624**	**2.277**
Pratylenchus	Dendrolaelaps	**-1.226**	**0.027**	**4.624**	**1.976**
Pratylenchus	Lysigamasus	**-1.226**	**0.407**	**4.624**	**2.821**

Pratylenchus	Pergamasus	**-1.226**	**1.081**	**4.624**	**2.277**
Pratylenchus	Uropoda	**-1.226**	**0.481**	**4.624**	**1.976**
Pratylenchus	Dorylaimoidea	**-1.226**	**-0.604**	**4.624**	**4.858**
Pratylenchus	Eupodes	**-1.226**	**0.005**	**4.624**	**2.675**
Pratylenchus	Mesostigmata	**-1.226**	**-0.411**	**4.624**	**1.976**
Pratylenchus	Scutacarus	**-1.226**	**-0.608**	**4.624**	**3.391**
Pratylenchus	Tarsonemus	**-1.226**	**-0.701**	**4.624**	**2.976**
Trichodorus	Mononchidae	**-0.744**	**-0.827**	**4.478**	**3.779**
Trichodorus	Alliphis	**-0.744**	**0.053**	**4.478**	**3.090**
Trichodorus	Cheiroseius	**-0.744**	**0.356**	**4.478**	**2.277**
Trichodorus	Dendrolaelaps	**-0.744**	**0.027**	**4.478**	**1.976**
Trichodorus	Lysigamasus	**-0.744**	**0.407**	**4.478**	**2.821**
Trichodorus	Pergamasus	**-0.744**	**1.081**	**4.478**	**2.277**
Trichodorus	Uropoda	**-0.744**	**0.481**	**4.478**	**1.976**
Trichodorus	Dorylaimoidea	**-0.744**	**-0.604**	**4.478**	**4.858**
Trichodorus	Eupodes	**-0.744**	**0.005**	**4.478**	**2.675**
Trichodorus	Mesostigmata	**-0.744**	**-0.411**	**4.478**	**1.976**
Trichodorus	Scutacarus	**-0.744**	**-0.608**	**4.478**	**3.391**
Trichodorus	Tarsonemus	**-0.744**	**-0.701**	**4.478**	**2.976**
Pachygnatidae	Cheiroseius	**-0.113**	**0.356**	**1.976**	**2.277**
Pachygnatidae	Dendrolaelaps	**-0.113**	**0.027**	**1.976**	**1.976**
Pachygnatidae	Lysigamasus	**-0.113**	**0.407**	**1.976**	**2.821**
Pachygnatidae	Pergamasus	**-0.113**	**1.081**	**1.976**	**2.277**
Pachygnatidae	Uropoda	**-0.113**	**0.481**	**1.976**	**1.976**
Pachygnatidae	Dorylaimoidea	**-0.113**	**-0.604**	**1.976**	**4.858**
Pachygnatidae	Eupodes	**-0.113**	**0.005**	**1.976**	**2.675**
Pachygnatidae	Mesostigmata	**-0.113**	**-0.411**	**1.976**	**1.976**
Pachygnatidae	Scutacarus	**-0.113**	**-0.608**	**1.976**	**3.391**
Pachygnatidae	Tarsonemus	**-0.113**	**-0.701**	**1.976**	**2.976**
Pachygnatidae	Pyemotes	**-0.113**	**-0.608**	**1.976**	**2.578**
Tydeidae	Cheiroseius	**-0.608**	**0.356**	**2.754**	**2.277**
Tydeidae	Dendrolaelaps	**-0.608**	**0.027**	**2.754**	**1.976**
Tydeidae	Lysigamasus	**-0.608**	**0.407**	**2.754**	**2.821**
Tydeidae	Pergamasus	**-0.608**	**1.081**	**2.754**	**2.277**
Tydeidae	Uropoda	**-0.608**	**0.481**	**2.754**	**1.976**
Tydeidae	Dorylaimoidea	**-0.608**	**-0.604**	**2.754**	**4.858**
Tydeidae	Eupodes	**-0.608**	**0.005**	**2.754**	**2.675**
Tydeidae	Mesostigmata	**-0.608**	**-0.411**	**2.754**	**1.976**
Tydeidae	Scutacarus	**-0.608**	**-0.608**	**2.754**	**3.391**
Tydeidae	Tarsonemus	**-0.608**	**-0.701**	**2.754**	**2.976**
Tydeidae	Pyemotes	**-0.608**	**-0.608**	**2.754**	**2.578**
Sminthurinus	Cheiroseius	**0.618**	**0.356**	**2.453**	**2.277**
Sminthurinus	Dendrolaelaps	**0.618**	**0.027**	**2.453**	**1.976**
Sminthurinus	Lysigamasus	**0.618**	**0.407**	**2.453**	**2.821**
Sminthurinus	Pergamasus	**0.618**	**1.081**	**2.453**	**2.277**
Sminthurinus	Uropoda	**0.618**	**0.481**	**2.453**	**1.976**
Sminthurinus	Dorylaimoidea	**0.618**	**-0.604**	**2.453**	**4.858**
Sminthurinus	Eupodes	**0.618**	**0.005**	**2.453**	**2.675**
Sminthurinus	Mesostigmata	**0.618**	**-0.411**	**2.453**	**1.976**
Sminthurinus	Scutacarus	**0.618**	**-0.608**	**2.453**	**3.391**
Sminthurinus	Tarsonemus	**0.618**	**-0.701**	**2.453**	**2.976**

Sminthurus	Cheiroseius	**1.429**	**0.356**	**1.976**	**2.277**
Sminthurus	Dendrolaelaps	**1.429**	**0.027**	**1.976**	**1.976**
Sminthurus	Lysigamasus	**1.429**	**0.407**	**1.976**	**2.821**
Sminthurus	Pergamasus	**1.429**	**1.081**	**1.976**	**2.277**
Sminthurus	Uropoda	**1.429**	**0.481**	**1.976**	**1.976**
Sminthurus	Dorylaimoidea	**1.429**	**-0.604**	**1.976**	**4.858**
Sminthurus	Eupodes	**1.429**	**0.005**	**1.976**	**2.675**
Sminthurus	Mesostigmata	**1.429**	**-0.411**	**1.976**	**1.976**
Sminthurus	Scutacarus	**1.429**	**-0.608**	**1.976**	**3.391**
Sminthurus	Tarsonemus	**1.429**	**-0.701**	**1.976**	**2.976**
Sphaeridia	Cheiroseius	**0.202**	**0.356**	**1.976**	**2.277**
Sphaeridia	Dendrolaelaps	**0.202**	**0.027**	**1.976**	**1.976**
Sphaeridia	Lysigamasus	**0.202**	**0.407**	**1.976**	**2.821**
Sphaeridia	Pergamasus	**0.202**	**1.081**	**1.976**	**2.277**
Sphaeridia	Uropoda	**0.202**	**0.481**	**1.976**	**1.976**
Sphaeridia	Dorylaimoidea	**0.202**	**-0.604**	**1.976**	**4.858**
Sphaeridia	Eupodes	**0.202**	**0.005**	**1.976**	**2.675**
Sphaeridia	Mesostigmata	**0.202**	**-0.411**	**1.976**	**1.976**
Sphaeridia	Scutacarus	**0.202**	**-0.608**	**1.976**	**3.391**
Sphaeridia	Tarsonemus	**0.202**	**-0.701**	**1.976**	**2.976**
Aphelenchoides	Mononchidae	**-1.496**	**-0.827**	**4.256**	**3.779**
Aphelenchoides	Alliphis	**-1.496**	**0.053**	**4.256**	**3.090**
Aphelenchoides	Cheiroseius	**-1.496**	**0.356**	**4.256**	**2.277**
Aphelenchoides	Dendrolaelaps	**-1.496**	**0.027**	**4.256**	**1.976**
Aphelenchoides	Lysigamasus	**-1.496**	**0.407**	**4.256**	**2.821**
Aphelenchoides	Pergamasus	**-1.496**	**1.081**	**4.256**	**2.277**
Aphelenchoides	Uropoda	**-1.496**	**0.481**	**4.256**	**1.976**
Aphelenchoides	Dorylaimoidea	**-1.496**	**-0.604**	**4.256**	**4.858**
Aphelenchoides	Eupodes	**-1.496**	**0.005**	**4.256**	**2.675**
Aphelenchoides	Mesostigmata	**-1.496**	**-0.411**	**4.256**	**1.976**
Aphelenchoides	Scutacarus	**-1.496**	**-0.608**	**4.256**	**3.391**
Aphelenchoides	Tarsonemus	**-1.496**	**-0.701**	**4.256**	**2.976**
Diphtherophora	Mononchidae	**-1.080**	**-0.827**	**3.779**	**3.779**
Diphtherophora	Alliphis	**-1.080**	**0.053**	**3.779**	**3.090**
Diphtherophora	Cheiroseius	**-1.080**	**0.356**	**3.779**	**2.277**
Diphtherophora	Dendrolaelaps	**-1.080**	**0.027**	**3.779**	**1.976**
Diphtherophora	Lysigamasus	**-1.080**	**0.407**	**3.779**	**2.821**
Diphtherophora	Pergamasus	**-1.080**	**1.081**	**3.779**	**2.277**
Diphtherophora	Uropoda	**-1.080**	**0.481**	**3.779**	**1.976**
Diphtherophora	Dorylaimoidea	**-1.080**	**-0.604**	**3.779**	**4.858**
Diphtherophora	Eupodes	**-1.080**	**0.005**	**3.779**	**2.675**
Diphtherophora	Mesostigmata	**-1.080**	**-0.411**	**3.779**	**1.976**
Diphtherophora	Scutacarus	**-1.080**	**-0.608**	**3.779**	**3.391**
Diphtherophora	Tarsonemus	**-1.080**	**-0.701**	**3.779**	**2.976**
Tylenchidae	Mononchidae	**-1.360**	**-0.827**	**4.821**	**3.779**
Tylenchidae	Alliphis	**-1.360**	**0.053**	**4.821**	**3.090**
Tylenchidae	Cheiroseius	**-1.360**	**0.356**	**4.821**	**2.277**
Tylenchidae	Dendrolaelaps	**-1.360**	**0.027**	**4.821**	**1.976**
Tylenchidae	Lysigamasus	**-1.360**	**0.407**	**4.821**	**2.821**
Tylenchidae	Pergamasus	**-1.360**	**1.081**	**4.821**	**2.277**
Tylenchidae	Uropoda	**-1.360**	**0.481**	**4.821**	**1.976**

Tylenchidae	Dorylaimoidea	**-1.360**	**-0.604**	**4.821**	**4.858**
Tylenchidae	Eupodes	**-1.360**	**0.005**	**4.821**	**2.675**
Tylenchidae	Mesostigmata	**-1.360**	**-0.411**	**4.821**	**1.976**
Tylenchidae	Scutacarus	**-1.360**	**-0.608**	**4.821**	**3.391**
Tylenchidae	Tarsonemus	**-1.360**	**-0.701**	**4.821**	**2.976**
Microppia	Cheiroseius	**-0.544**	**0.356**	**2.277**	**2.277**
Microppia	Dendrolaelaps	**-0.544**	**0.027**	**2.277**	**1.976**
Microppia	Lysigamasus	**-0.544**	**0.407**	**2.277**	**2.821**
Microppia	Pergamasus	**-0.544**	**1.081**	**2.277**	**2.277**
Microppia	Uropoda	**-0.544**	**0.481**	**2.277**	**1.976**
Microppia	Dorylaimoidea	**-0.544**	**-0.604**	**2.277**	**4.858**
Microppia	Eupodes	**-0.544**	**0.005**	**2.277**	**2.675**
Microppia	Mesostigmata	**-0.544**	**-0.411**	**2.277**	**1.976**
Microppia	Scutacarus	**-0.544**	**-0.608**	**2.277**	**3.391**
Microppia	Tarsonemus	**-0.544**	**-0.701**	**2.277**	**2.976**
Microppia	Pyemotes	**-0.544**	**-0.608**	**2.277**	**2.578**
Microtydeus	Cheiroseius	**-0.863**	**0.356**	**1.976**	**2.277**
Microtydeus	Dendrolaelaps	**-0.863**	**0.027**	**1.976**	**1.976**
Microtydeus	Lysigamasus	**-0.863**	**0.407**	**1.976**	**2.821**
Microtydeus	Pergamasus	**-0.863**	**1.081**	**1.976**	**2.277**
Microtydeus	Uropoda	**-0.863**	**0.481**	**1.976**	**1.976**
Microtydeus	Dorylaimoidea	**-0.863**	**-0.604**	**1.976**	**4.858**
Microtydeus	Eupodes	**-0.863**	**0.005**	**1.976**	**2.675**
Microtydeus	Mesostigmata	**-0.863**	**-0.411**	**1.976**	**1.976**
Microtydeus	Scutacarus	**-0.863**	**-0.608**	**1.976**	**3.391**
Microtydeus	Tarsonemus	**-0.863**	**-0.701**	**1.976**	**2.976**
Microtydeus	Pyemotes	**-0.863**	**-0.608**	**1.976**	**2.578**
Oppiella	Cheiroseius	**-0.447**	**0.356**	**1.976**	**2.277**
Oppiella	Dendrolaelaps	**-0.447**	**0.027**	**1.976**	**1.976**
Oppiella	Lysigamasus	**-0.447**	**0.407**	**1.976**	**2.821**
Oppiella	Pergamasus	**-0.447**	**1.081**	**1.976**	**2.277**
Oppiella	Uropoda	**-0.447**	**0.481**	**1.976**	**1.976**
Oppiella	Dorylaimoidea	**-0.447**	**-0.604**	**1.976**	**4.858**
Oppiella	Eupodes	**-0.447**	**0.005**	**1.976**	**2.675**
Oppiella	Mesostigmata	**-0.447**	**-0.411**	**1.976**	**1.976**
Oppiella	Scutacarus	**-0.447**	**-0.608**	**1.976**	**3.391**
Oppiella	Tarsonemus	**-0.447**	**-0.701**	**1.976**	**2.976**
Oppiella	Pyemotes	**-0.447**	**-0.608**	**1.976**	**2.578**
Pygmephorus	Cheiroseius	**-0.376**	**0.356**	**3.231**	**2.277**
Pygmephorus	Dendrolaelaps	**-0.376**	**0.027**	**3.231**	**1.976**
Pygmephorus	Lysigamasus	**-0.376**	**0.407**	**3.231**	**2.821**
Pygmephorus	Pergamasus	**-0.376**	**1.081**	**3.231**	**2.277**
Pygmephorus	Uropoda	**-0.376**	**0.481**	**3.231**	**1.976**
Pygmephorus	Dorylaimoidea	**-0.376**	**-0.604**	**3.231**	**4.858**
Pygmephorus	Eupodes	**-0.376**	**0.005**	**3.231**	**2.675**
Pygmephorus	Mesostigmata	**-0.376**	**-0.411**	**3.231**	**1.976**
Pygmephorus	Scutacarus	**-0.376**	**-0.608**	**3.231**	**3.391**
Pygmephorus	Tarsonemus	**-0.376**	**-0.701**	**3.231**	**2.976**
Pygmephorus	Pyemotes	**-0.376**	**-0.608**	**3.231**	**2.578**
Tyrophagus	Cheiroseius	**0.005**	**0.356**	**3.017**	**2.277**
Tyrophagus	Dendrolaelaps	**0.005**	**0.027**	**3.017**	**1.976**

Tyrophagus	Lysigamasus	**0.005**	**0.407**	**3.017**	**2.821**
Tyrophagus	Pergamasus	**0.005**	**1.081**	**3.017**	**2.277**
Tyrophagus	Uropoda	**0.005**	**0.481**	**3.017**	**1.976**
Tyrophagus	Dorylaimoidea	**0.005**	**-0.604**	**3.017**	**4.858**
Tyrophagus	Eupodes	**0.005**	**0.005**	**3.017**	**2.675**
Tyrophagus	Mesostigmata	**0.005**	**-0.411**	**3.017**	**1.976**
Tyrophagus	Scutacarus	**0.005**	**-0.608**	**3.017**	**3.391**
Tyrophagus	Tarsonemus	**0.005**	**-0.701**	**3.017**	**2.976**
Tyrophagus	Pyemotes	**0.005**	**-0.608**	**3.017**	**2.578**
Brachystomella	Cheiroseius	**0.977**	**0.356**	**1.976**	**2.277**
Brachystomella	Dendrolaelaps	**0.977**	**0.027**	**1.976**	**1.976**
Brachystomella	Lysigamasus	**0.977**	**0.407**	**1.976**	**2.821**
Brachystomella	Pergamasus	**0.977**	**1.081**	**1.976**	**2.277**
Brachystomella	Uropoda	**0.977**	**0.481**	**1.976**	**1.976**
Brachystomella	Dorylaimoidea	**0.977**	**-0.604**	**1.976**	**4.858**
Brachystomella	Eupodes	**0.977**	**0.005**	**1.976**	**2.675**
Brachystomella	Mesostigmata	**0.977**	**-0.411**	**1.976**	**1.976**
Brachystomella	Scutacarus	**0.977**	**-0.608**	**1.976**	**3.391**
Brachystomella	Tarsonemus	**0.977**	**-0.701**	**1.976**	**2.976**
Ceratophysella	Cheiroseius	**1.335**	**0.356**	**2.675**	**2.277**
Ceratophysella	Dendrolaelaps	**1.335**	**0.027**	**2.675**	**1.976**
Ceratophysella	Lysigamasus	**1.335**	**0.407**	**2.675**	**2.821**
Ceratophysella	Pergamasus	**1.335**	**1.081**	**2.675**	**2.277**
Ceratophysella	Uropoda	**1.335**	**0.481**	**2.675**	**1.976**
Ceratophysella	Dorylaimoidea	**1.335**	**-0.604**	**2.675**	**4.858**
Ceratophysella	Eupodes	**1.335**	**0.005**	**2.675**	**2.675**
Ceratophysella	Mesostigmata	**1.335**	**-0.411**	**2.675**	**1.976**
Ceratophysella	Scutacarus	**1.335**	**-0.608**	**2.675**	**3.391**
Ceratophysella	Tarsonemus	**1.335**	**-0.701**	**2.675**	**2.976**
Folsomia	Cheiroseius	**0.900**	**0.356**	**1.976**	**2.277**
Folsomia	Dendrolaelaps	**0.900**	**0.027**	**1.976**	**1.976**
Folsomia	Lysigamasus	**0.900**	**0.407**	**1.976**	**2.821**
Folsomia	Pergamasus	**0.900**	**1.081**	**1.976**	**2.277**
Folsomia	Uropoda	**0.900**	**0.481**	**1.976**	**1.976**
Folsomia	Dorylaimoidea	**0.900**	**-0.604**	**1.976**	**4.858**
Folsomia	Eupodes	**0.900**	**0.005**	**1.976**	**2.675**
Folsomia	Mesostigmata	**0.900**	**-0.411**	**1.976**	**1.976**
Folsomia	Scutacarus	**0.900**	**-0.608**	**1.976**	**3.391**
Folsomia	Tarsonemus	**0.900**	**-0.701**	**1.976**	**2.976**
Isotoma	Cheiroseius	**1.898**	**0.356**	**3.544**	**2.277**
Isotoma	Dendrolaelaps	**1.898**	**0.027**	**3.544**	**1.976**
Isotoma	Lysigamasus	**1.898**	**0.407**	**3.544**	**2.821**
Isotoma	Pergamasus	**1.898**	**1.081**	**3.544**	**2.277**
Isotoma	Uropoda	**1.898**	**0.481**	**3.544**	**1.976**
Isotoma	Dorylaimoidea	**1.898**	**-0.604**	**3.544**	**4.858**
Isotoma	Eupodes	**1.898**	**0.005**	**3.544**	**2.675**
Isotoma	Mesostigmata	**1.898**	**-0.411**	**3.544**	**1.976**
Isotoma	Scutacarus	**1.898**	**-0.608**	**3.544**	**3.391**
Isotoma	Tarsonemus	**1.898**	**-0.701**	**3.544**	**2.976**
Isotomurus	Cheiroseius	**1.787**	**0.356**	**2.277**	**2.277**
Isotomurus	Dendrolaelaps	**1.787**	**0.027**	**2.277**	**1.976**

Isotomurus	Lysigamasus	**1.787**	**0.407**	**2.277**	**2.821**
Isotomurus	Pergamasus	**1.787**	**1.081**	**2.277**	**2.277**
Isotomurus	Uropoda	**1.787**	**0.481**	**2.277**	**1.976**
Isotomurus	Dorylaimoidea	**1.787**	**-0.604**	**2.277**	**4.858**
Isotomurus	Eupodes	**1.787**	**0.005**	**2.277**	**2.675**
Isotomurus	Mesostigmata	**1.787**	**-0.411**	**2.277**	**1.976**
Isotomurus	Scutacarus	**1.787**	**-0.608**	**2.277**	**3.391**
Isotomurus	Tarsonemus	**1.787**	**-0.701**	**2.277**	**2.976**
Proisotoma	Cheiroseius	**0.770**	**0.356**	**2.675**	**2.277**
Proisotoma	Dendrolaelaps	**0.770**	**0.027**	**2.675**	**1.976**
Proisotoma	Lysigamasus	**0.770**	**0.407**	**2.675**	**2.821**
Proisotoma	Pergamasus	**0.770**	**1.081**	**2.675**	**2.277**
Proisotoma	Uropoda	**0.770**	**0.481**	**2.675**	**1.976**
Proisotoma	Dorylaimoidea	**0.770**	**-0.604**	**2.675**	**4.858**
Proisotoma	Eupodes	**0.770**	**0.005**	**2.675**	**2.675**
Proisotoma	Mesostigmata	**0.770**	**-0.411**	**2.675**	**1.976**
Proisotoma	Scutacarus	**0.770**	**-0.608**	**2.675**	**3.391**
Proisotoma	Tarsonemus	**0.770**	**-0.701**	**2.675**	**2.976**
Fridericia	Cheiroseius	**1.623**	**0.356**	**4.632**	**2.277**
Fridericia	Dendrolaelaps	**1.623**	**0.027**	**4.632**	**1.976**
Fridericia	Lysigamasus	**1.623**	**0.407**	**4.632**	**2.821**
Fridericia	Pergamasus	**1.623**	**1.081**	**4.632**	**2.277**
Fridericia	Uropoda	**1.623**	**0.481**	**4.632**	**1.976**
Fridericia	Dorylaimoidea	**1.623**	**-0.604**	**4.632**	**4.858**
Fridericia	Eupodes	**1.623**	**0.005**	**4.632**	**2.675**
Fridericia	Mesostigmata	**1.623**	**-0.411**	**4.632**	**1.976**
Fridericia	Scutacarus	**1.623**	**-0.608**	**4.632**	**3.391**
Fridericia	Tarsonemus	**1.623**	**-0.701**	**4.632**	**2.976**
Acrobeloides	Mononchidae	**-1.171**	**-0.827**	**5.034**	**3.779**
Acrobeloides	Alliphis	**-1.171**	**0.053**	**5.034**	**3.090**
Acrobeloides	Cheiroseius	**-1.171**	**0.356**	**5.034**	**2.277**
Acrobeloides	Dendrolaelaps	**-1.171**	**0.027**	**5.034**	**1.976**
Acrobeloides	Lysigamasus	**-1.171**	**0.407**	**5.034**	**2.821**
Acrobeloides	Pergamasus	**-1.171**	**1.081**	**5.034**	**2.277**
Acrobeloides	Uropoda	**-1.171**	**0.481**	**5.034**	**1.976**
Acrobeloides	Dorylaimoidea	**-1.171**	**-0.604**	**5.034**	**4.858**
Acrobeloides	Eupodes	**-1.171**	**0.005**	**5.034**	**2.675**
Acrobeloides	Mesostigmata	**-1.171**	**-0.411**	**5.034**	**1.976**
Acrobeloides	Scutacarus	**-1.171**	**-0.608**	**5.034**	**3.391**
Acrobeloides	Tarsonemus	**-1.171**	**-0.701**	**5.034**	**2.976**
Anaplectus	Mononchidae	**-0.519**	**-0.827**	**3.779**	**3.779**
Anaplectus	Alliphis	**-0.519**	**0.053**	**3.779**	**3.090**
Anaplectus	Cheiroseius	**-0.519**	**0.356**	**3.779**	**2.277**
Anaplectus	Dendrolaelaps	**-0.519**	**0.027**	**3.779**	**1.976**
Anaplectus	Lysigamasus	**-0.519**	**0.407**	**3.779**	**2.821**
Anaplectus	Pergamasus	**-0.519**	**1.081**	**3.779**	**2.277**
Anaplectus	Uropoda	**-0.519**	**0.481**	**3.779**	**1.976**
Anaplectus	Dorylaimoidea	**-0.519**	**-0.604**	**3.779**	**4.858**
Anaplectus	Eupodes	**-0.519**	**0.005**	**3.779**	**2.675**
Anaplectus	Mesostigmata	**-0.519**	**-0.411**	**3.779**	**1.976**
Anaplectus	Scutacarus	**-0.519**	**-0.608**	**3.779**	**3.391**

Anaplectus	Tarsonemus	**-0.519**	**-0.701**	**3.779**	**2.976**
Bastiania	Mononchidae	**-0.946**	**-0.827**	**3.779**	**3.779**
Bastiania	Alliphis	**-0.946**	**0.053**	**3.779**	**3.090**
Bastiania	Cheiroseius	**-0.946**	**0.356**	**3.779**	**2.277**
Bastiania	Dendrolaelaps	**-0.946**	**0.027**	**3.779**	**1.976**
Bastiania	Lysigamasus	**-0.946**	**0.407**	**3.779**	**2.821**
Bastiania	Pergamasus	**-0.946**	**1.081**	**3.779**	**2.277**
Bastiania	Uropoda	**-0.946**	**0.481**	**3.779**	**1.976**
Bastiania	Dorylaimoidea	**-0.946**	**-0.604**	**3.779**	**4.858**
Bastiania	Eupodes	**-0.946**	**0.005**	**3.779**	**2.675**
Bastiania	Mesostigmata	**-0.946**	**-0.411**	**3.779**	**1.976**
Bastiania	Scutacarus	**-0.946**	**-0.608**	**3.779**	**3.391**
Bastiania	Tarsonemus	**-0.946**	**-0.701**	**3.779**	**2.976**
Cephalobidae	Mononchidae	**-1.055**	**-0.827**	**4.557**	**3.779**
Cephalobidae	Alliphis	**-1.055**	**0.053**	**4.557**	**3.090**
Cephalobidae	Cheiroseius	**-1.055**	**0.356**	**4.557**	**2.277**
Cephalobidae	Dendrolaelaps	**-1.055**	**0.027**	**4.557**	**1.976**
Cephalobidae	Lysigamasus	**-1.055**	**0.407**	**4.557**	**2.821**
Cephalobidae	Pergamasus	**-1.055**	**1.081**	**4.557**	**2.277**
Cephalobidae	Uropoda	**-1.055**	**0.481**	**4.557**	**1.976**
Cephalobidae	Dorylaimoidea	**-1.055**	**-0.604**	**4.557**	**4.858**
Cephalobidae	Eupodes	**-1.055**	**0.005**	**4.557**	**2.675**
Cephalobidae	Mesostigmata	**-1.055**	**-0.411**	**4.557**	**1.976**
Cephalobidae	Scutacarus	**-1.055**	**-0.608**	**4.557**	**3.391**
Cephalobidae	Tarsonemus	**-1.055**	**-0.701**	**4.557**	**2.976**
Cervidellus	Mononchidae	**-1.244**	**-0.827**	**3.779**	**3.779**
Cervidellus	Alliphis	**-1.244**	**0.053**	**3.779**	**3.090**
Cervidellus	Cheiroseius	**-1.244**	**0.356**	**3.779**	**2.277**
Cervidellus	Dendrolaelaps	**-1.244**	**0.027**	**3.779**	**1.976**
Cervidellus	Lysigamasus	**-1.244**	**0.407**	**3.779**	**2.821**
Cervidellus	Pergamasus	**-1.244**	**1.081**	**3.779**	**2.277**
Cervidellus	Uropoda	**-1.244**	**0.481**	**3.779**	**1.976**
Cervidellus	Dorylaimoidea	**-1.244**	**-0.604**	**3.779**	**4.858**
Cervidellus	Eupodes	**-1.244**	**0.005**	**3.779**	**2.675**
Cervidellus	Mesostigmata	**-1.244**	**-0.411**	**3.779**	**1.976**
Cervidellus	Scutacarus	**-1.244**	**-0.608**	**3.779**	**3.391**
Cervidellus	Tarsonemus	**-1.244**	**-0.701**	**3.779**	**2.976**
Eucephalobus	Mononchidae	**-0.855**	**-0.827**	**4.821**	**3.779**
Eucephalobus	Alliphis	**-0.855**	**0.053**	**4.821**	**3.090**
Eucephalobus	Cheiroseius	**-0.855**	**0.356**	**4.821**	**2.277**
Eucephalobus	Dendrolaelaps	**-0.855**	**0.027**	**4.821**	**1.976**
Eucephalobus	Lysigamasus	**-0.855**	**0.407**	**4.821**	**2.821**
Eucephalobus	Pergamasus	**-0.855**	**1.081**	**4.821**	**2.277**
Eucephalobus	Uropoda	**-0.855**	**0.481**	**4.821**	**1.976**
Eucephalobus	Dorylaimoidea	**-0.855**	**-0.604**	**4.821**	**4.858**
Eucephalobus	Eupodes	**-0.855**	**0.005**	**4.821**	**2.675**
Eucephalobus	Mesostigmata	**-0.855**	**-0.411**	**4.821**	**1.976**
Eucephalobus	Scutacarus	**-0.855**	**-0.608**	**4.821**	**3.391**
Eucephalobus	Tarsonemus	**-0.855**	**-0.701**	**4.821**	**2.976**
Panagrolaimus	Mononchidae	**-0.945**	**-0.827**	**3.779**	**3.779**
Panagrolaimus	Alliphis	**-0.945**	**0.053**	**3.779**	**3.090**

Panagrolaimus	Cheiroseius	**-0.945**	**0.356**	**3.779**	**2.277**
Panagrolaimus	Dendrolaelaps	**-0.945**	**0.027**	**3.779**	**1.976**
Panagrolaimus	Lysigamasus	**-0.945**	**0.407**	**3.779**	**2.821**
Panagrolaimus	Pergamasus	**-0.945**	**1.081**	**3.779**	**2.277**
Panagrolaimus	Uropoda	**-0.945**	**0.481**	**3.779**	**1.976**
Panagrolaimus	Dorylaimoidea	**-0.945**	**-0.604**	**3.779**	**4.858**
Panagrolaimus	Eupodes	**-0.945**	**0.005**	**3.779**	**2.675**
Panagrolaimus	Mesostigmata	**-0.945**	**-0.411**	**3.779**	**1.976**
Panagrolaimus	Scutacarus	**-0.945**	**-0.608**	**3.779**	**3.391**
Panagrolaimus	Tarsonemus	**-0.945**	**-0.701**	**3.779**	**2.976**
Plectus	Mononchidae	**-0.583**	**-0.827**	**4.478**	**3.779**
Plectus	Alliphis	**-0.583**	**0.053**	**4.478**	**3.090**
Plectus	Cheiroseius	**-0.583**	**0.356**	**4.478**	**2.277**
Plectus	Dendrolaelaps	**-0.583**	**0.027**	**4.478**	**1.976**
Plectus	Lysigamasus	**-0.583**	**0.407**	**4.478**	**2.821**
Plectus	Pergamasus	**-0.583**	**1.081**	**4.478**	**2.277**
Plectus	Uropoda	**-0.583**	**0.481**	**4.478**	**1.976**
Plectus	Dorylaimoidea	**-0.583**	**-0.604**	**4.478**	**4.858**
Plectus	Eupodes	**-0.583**	**0.005**	**4.478**	**2.675**
Plectus	Mesostigmata	**-0.583**	**-0.411**	**4.478**	**1.976**
Plectus	Scutacarus	**-0.583**	**-0.608**	**4.478**	**3.391**
Plectus	Tarsonemus	**-0.583**	**-0.701**	**4.478**	**2.976**
Rhabditidae	Mononchidae	**-0.692**	**-0.827**	**5.370**	**3.779**
Rhabditidae	Alliphis	**-0.692**	**0.053**	**5.370**	**3.090**
Rhabditidae	Cheiroseius	**-0.692**	**0.356**	**5.370**	**2.277**
Rhabditidae	Dendrolaelaps	**-0.692**	**0.027**	**5.370**	**1.976**
Rhabditidae	Lysigamasus	**-0.692**	**0.407**	**5.370**	**2.821**
Rhabditidae	Pergamasus	**-0.692**	**1.081**	**5.370**	**2.277**
Rhabditidae	Uropoda	**-0.692**	**0.481**	**5.370**	**1.976**
Rhabditidae	Dorylaimoidea	**-0.692**	**-0.604**	**5.370**	**4.858**
Rhabditidae	Eupodes	**-0.692**	**0.005**	**5.370**	**2.675**
Rhabditidae	Mesostigmata	**-0.692**	**-0.411**	**5.370**	**1.976**
Rhabditidae	Scutacarus	**-0.692**	**-0.608**	**5.370**	**3.391**
Rhabditidae	Tarsonemus	**-0.692**	**-0.701**	**5.370**	**2.976**
Enchytraeus	Cheiroseius	**1.150**	**0.356**	**4.633**	**2.277**
Enchytraeus	Dendrolaelaps	**1.150**	**0.027**	**4.633**	**1.976**
Enchytraeus	Lysigamasus	**1.150**	**0.407**	**4.633**	**2.821**
Enchytraeus	Pergamasus	**1.150**	**1.081**	**4.633**	**2.277**
Enchytraeus	Uropoda	**1.150**	**0.481**	**4.633**	**1.976**
Enchytraeus	Dorylaimoidea	**1.150**	**-0.604**	**4.633**	**4.858**
Enchytraeus	Eupodes	**1.150**	**0.005**	**4.633**	**2.675**
Enchytraeus	Mesostigmata	**1.150**	**-0.411**	**4.633**	**1.976**
Enchytraeus	Scutacarus	**1.150**	**-0.608**	**4.633**	**3.391**
Enchytraeus	Tarsonemus	**1.150**	**-0.701**	**4.633**	**2.976**
Eubacteria	Acrobeloides	**-6.735**	**-1.171**	**13.128**	**5.034**
Eubacteria	Anaplectus	**-6.735**	**-0.519**	**13.128**	**3.779**
Eubacteria	Bastiania	**-6.735**	**-0.946**	**13.128**	**3.779**
Eubacteria	Cephalobidae	**-6.735**	**-1.055**	**13.128**	**4.557**
Eubacteria	Cervidellus	**-6.735**	**-1.244**	**13.128**	**3.779**
Eubacteria	Eucephalobus	**-6.735**	**-0.855**	**13.128**	**4.821**
Eubacteria	Panagrolaimus	**-6.735**	**-0.945**	**13.128**	**3.779**

Eubacteria	Plectus	**-6.735**	**-0.583**	**13.128**	**4.478**
Eubacteria	Rhabditidae	**-6.735**	**-0.692**	**13.128**	**5.370**
Eubacteria	Enchytraeus	**-6.735**	**1.150**	**13.128**	**4.633**
Eubacteria	Dauerlarvae	**-6.735**	**-0.804**	**13.128**	**4.080**
Eubacteria	Juveniles	**-6.735**	**1.363**	**13.128**	**2.719**
Eubacteria	Henlea	**-6.735**	**1.863**	**13.128**	**4.085**
Eubacteria	Marionina	**-6.735**	**1.045**	**13.128**	**3.433**
Dauerlarvae	Mononchidae	**-0.804**	**-0.827**	**4.080**	**3.779**
Dauerlarvae	Alliphis	**-0.804**	**0.053**	**4.080**	**3.090**
Dauerlarvae	Dorylaimoidea	**-0.804**	**-0.604**	**4.080**	**4.858**
Dauerlarvae	Eupodes	**-0.804**	**0.005**	**4.080**	**2.675**
Dauerlarvae	Mesostigmata	**-0.804**	**-0.411**	**4.080**	**1.976**
Dauerlarvae	Scutacarus	**-0.804**	**-0.608**	**4.080**	**3.391**
Dauerlarvae	Tarsonemus	**-0.804**	**-0.701**	**4.080**	**2.976**
Juveniles	Cheiroseius	**1.363**	**0.356**	**2.719**	**2.277**
Juveniles	Dendrolaelaps	**1.363**	**0.027**	**2.719**	**1.976**
Juveniles	Lysigamasus	**1.363**	**0.407**	**2.719**	**2.821**
Juveniles	Pergamasus	**1.363**	**1.081**	**2.719**	**2.277**
Juveniles	Uropoda	**1.363**	**0.481**	**2.719**	**1.976**
Juveniles	Dorylaimoidea	**1.363**	**-0.604**	**2.719**	**4.858**
Juveniles	Eupodes	**1.363**	**0.005**	**2.719**	**2.675**
Juveniles	Mesostigmata	**1.363**	**-0.411**	**2.719**	**1.976**
Juveniles	Scutacarus	**1.363**	**-0.608**	**2.719**	**3.391**
Juveniles	Tarsonemus	**1.363**	**-0.701**	**2.719**	**2.976**
Henlea	Cheiroseius	**1.863**	**0.356**	**4.085**	**2.277**
Henlea	Dendrolaelaps	**1.863**	**0.027**	**4.085**	**1.976**
Henlea	Lysigamasus	**1.863**	**0.407**	**4.085**	**2.821**
Henlea	Pergamasus	**1.863**	**1.081**	**4.085**	**2.277**
Henlea	Uropoda	**1.863**	**0.481**	**4.085**	**1.976**
Henlea	Dorylaimoidea	**1.863**	**-0.604**	**4.085**	**4.858**
Henlea	Eupodes	**1.863**	**0.005**	**4.085**	**2.675**
Henlea	Mesostigmata	**1.863**	**-0.411**	**4.085**	**1.976**
Henlea	Scutacarus	**1.863**	**-0.608**	**4.085**	**3.391**
Henlea	Tarsonemus	**1.863**	**-0.701**	**4.085**	**2.976**
Marionina	Cheiroseius	**1.045**	**0.356**	**3.433**	**2.277**
Marionina	Dendrolaelaps	**1.045**	**0.027**	**3.433**	**1.976**
Marionina	Lysigamasus	**1.045**	**0.407**	**3.433**	**2.821**
Marionina	Pergamasus	**1.045**	**1.081**	**3.433**	**2.277**
Marionina	Uropoda	**1.045**	**0.481**	**3.433**	**1.976**
Marionina	Dorylaimoidea	**1.045**	**-0.604**	**3.433**	**4.858**
Marionina	Eupodes	**1.045**	**0.005**	**3.433**	**2.675**
Marionina	Mesostigmata	**1.045**	**-0.411**	**3.433**	**1.976**
Marionina	Scutacarus	**1.045**	**-0.608**	**3.433**	**3.391**
Marionina	Tarsonemus	**1.045**	**-0.701**	**3.433**	**2.976**
Hyphae and hair roots	Aglenchus	**6.941**	**-1.053**	**0.000**	**3.779**
Hyphae and hair roots	Dolichodoridae	**6.941**	**-0.885**	**0.000**	**4.779**
Hyphae and hair roots	Helicotylenchus	**6.941**	**-0.792**	**0.000**	**3.779**
Hyphae and hair roots	Hoplolaimidae	**6.941**	**-1.090**	**0.000**	**3.779**
Hyphae and hair roots	Malenchus	**6.941**	**-1.330**	**0.000**	**4.478**
Hyphae and hair roots	Meloidogyne	**6.941**	**-1.287**	**0.000**	**3.779**
Hyphae and hair roots	Paratrichodorus	**6.941**	**-0.630**	**0.000**	**3.779**

Hyphae and hair roots	Paratylenchus	6.941	-1.244	0.000	4.080
Hyphae and hair roots	Pratylenchus	6.941	-1.226	0.000	4.624
Hyphae and hair roots	Trichodorus	6.941	-0.744	0.000	4.478
Hyphae and hair roots	Pachygnatidae	6.941	-0.113	0.000	1.976
Hyphae and hair roots	Tydeidae	6.941	-0.608	0.000	2.754
Hyphae and hair roots	Sminthurinus	6.941	0.618	0.000	2.453
Hyphae and hair roots	Sminthurus	6.941	1.429	0.000	1.976
Hyphae and hair roots	Sphaeridia	6.941	0.202	0.000	1.976
Hyphae and hair roots	Aphelenchoides	6.941	-1.496	0.000	4.256
Hyphae and hair roots	Diphtherophora	6.941	-1.080	0.000	3.779
Hyphae and hair roots	Tylenchidae	6.941	-1.360	0.000	4.821
Hyphae and hair roots	Microppia	6.941	-0.544	0.000	2.277
Hyphae and hair roots	Microtydeus	6.941	-0.863	0.000	1.976
Hyphae and hair roots	Oppiella	6.941	-0.447	0.000	1.976
Hyphae and hair roots	Pygmephorus	6.941	-0.376	0.000	3.231
Hyphae and hair roots	Tyrophagus	6.941	0.005	0.000	3.017
Hyphae and hair roots	Brachystomella	6.941	0.977	0.000	1.976
Hyphae and hair roots	Ceratophysella	6.941	1.335	0.000	2.675
Hyphae and hair roots	Folsomia	6.941	0.900	0.000	1.976
Hyphae and hair roots	Isotoma	6.941	1.898	0.000	3.544
Hyphae and hair roots	Isotomurus	6.941	1.787	0.000	2.277
Hyphae and hair roots	Proisotoma	6.941	0.770	0.000	2.675
Hyphae and hair roots	Fridericia	6.941	1.623	0.000	4.632
Hyphae and hair roots	Dorylaimoidea	6.941	-0.604	0.000	4.858
Hyphae and hair roots	Eupodes	6.941	0.005	0.000	2.675
Hyphae and hair roots	Mesostigmata	6.941	-0.411	0.000	1.976
Hyphae and hair roots	Scutacarus	6.941	-0.608	0.000	3.391
Hyphae and hair roots	Tarsonemus	6.941	-0.701	0.000	2.976
Mononchidae	Cheiroseius	-0.827	0.356	3.779	2.277
Mononchidae	Dendrolaelaps	-0.827	0.027	3.779	1.976
Mononchidae	Lysigamasus	-0.827	0.407	3.779	2.821
Mononchidae	Pergamasus	-0.827	1.081	3.779	2.277
Mononchidae	Uropoda	-0.827	0.481	3.779	1.976
Mononchidae	Dorylaimoidea	-0.827	-0.604	3.779	4.858
Mononchidae	Eupodes	-0.827	0.005	3.779	2.675
Mononchidae	Mesostigmata	-0.827	-0.411	3.779	1.976
Mononchidae	Scutacarus	-0.827	-0.608	3.779	3.391
Mononchidae	Tarsonemus	-0.827	-0.701	3.779	2.976
Alliphis	Cheiroseius	0.053	0.356	3.090	2.277
Alliphis	Dendrolaelaps	0.053	0.027	3.090	1.976
Alliphis	Lysigamasus	0.053	0.407	3.090	2.821
Alliphis	Pergamasus	0.053	1.081	3.090	2.277
Alliphis	Uropoda	0.053	0.481	3.090	1.976
Alliphis	Dorylaimoidea	0.053	-0.604	3.090	4.858
Alliphis	Eupodes	0.053	0.005	3.090	2.675
Alliphis	Mesostigmata	0.053	-0.411	3.090	1.976
Alliphis	Scutacarus	0.053	-0.608	3.090	3.391
Alliphis	Tarsonemus	0.053	-0.701	3.090	2.976
Alliphis	Pyemotes	0.053	-0.608	3.090	2.578
Cheiroseius	Dorylaimoidea	0.356	-0.604	2.277	4.858
Cheiroseius	Eupodes	0.356	0.005	2.277	2.675

Cheiroseius	Mesostigmata	**0.356**	**-0.411**	**2.277**	**1.976**
Cheiroseius	Scutacarus	**0.356**	**-0.608**	**2.277**	**3.391**
Cheiroseius	Tarsonemus	**0.356**	**-0.701**	**2.277**	**2.976**
Cheiroseius	Pyemotes	**0.356**	**-0.608**	**2.277**	**2.578**
Dendrolaelaps	Dorylaimoidea	**0.027**	**-0.604**	**1.976**	**4.858**
Dendrolaelaps	Eupodes	**0.027**	**0.005**	**1.976**	**2.675**
Dendrolaelaps	Mesostigmata	**0.027**	**-0.411**	**1.976**	**1.976**
Dendrolaelaps	Scutacarus	**0.027**	**-0.608**	**1.976**	**3.391**
Dendrolaelaps	Tarsonemus	**0.027**	**-0.701**	**1.976**	**2.976**
Dendrolaelaps	Pyemotes	**0.027**	**-0.608**	**1.976**	**2.578**
Lysigamasus	Dorylaimoidea	**0.407**	**-0.604**	**2.821**	**4.858**
Lysigamasus	Eupodes	**0.407**	**0.005**	**2.821**	**2.675**
Lysigamasus	Mesostigmata	**0.407**	**-0.411**	**2.821**	**1.976**
Lysigamasus	Scutacarus	**0.407**	**-0.608**	**2.821**	**3.391**
Lysigamasus	Tarsonemus	**0.407**	**-0.701**	**2.821**	**2.976**
Lysigamasus	Pyemotes	**0.407**	**-0.608**	**2.821**	**2.578**
Pergamasus	Dorylaimoidea	**1.081**	**-0.604**	**2.277**	**4.858**
Pergamasus	Eupodes	**1.081**	**0.005**	**2.277**	**2.675**
Pergamasus	Mesostigmata	**1.081**	**-0.411**	**2.277**	**1.976**
Pergamasus	Scutacarus	**1.081**	**-0.608**	**2.277**	**3.391**
Pergamasus	Tarsonemus	**1.081**	**-0.701**	**2.277**	**2.976**
Pergamasus	Pyemotes	**1.081**	**-0.608**	**2.277**	**2.578**
Uropoda	Dorylaimoidea	**0.481**	**-0.604**	**1.976**	**4.858**
Uropoda	Eupodes	**0.481**	**0.005**	**1.976**	**2.675**
Uropoda	Mesostigmata	**0.481**	**-0.411**	**1.976**	**1.976**
Uropoda	Scutacarus	**0.481**	**-0.608**	**1.976**	**3.391**
Uropoda	Tarsonemus	**0.481**	**-0.701**	**1.976**	**2.976**
Uropoda	Pyemotes	**0.481**	**-0.608**	**1.976**	**2.578**
Dorylaimoidea	Mononchidae	**-0.604**	**-0.827**	**4.858**	**3.779**
Dorylaimoidea	Alliphis	**-0.604**	**0.053**	**4.858**	**3.090**
Dorylaimoidea	Cheiroseius	**-0.604**	**0.356**	**4.858**	**2.277**
Dorylaimoidea	Dendrolaelaps	**-0.604**	**0.027**	**4.858**	**1.976**
Dorylaimoidea	Lysigamasus	**-0.604**	**0.407**	**4.858**	**2.821**
Dorylaimoidea	Pergamasus	**-0.604**	**1.081**	**4.858**	**2.277**
Dorylaimoidea	Uropoda	**-0.604**	**0.481**	**4.858**	**1.976**
Dorylaimoidea	Dorylaimoidea	**-0.604**	**-0.604**	**4.858**	**4.858**
Dorylaimoidea	Eupodes	**-0.604**	**0.005**	**4.858**	**2.675**
Dorylaimoidea	Mesostigmata	**-0.604**	**-0.411**	**4.858**	**1.976**
Dorylaimoidea	Scutacarus	**-0.604**	**-0.608**	**4.858**	**3.391**
Dorylaimoidea	Tarsonemus	**-0.604**	**-0.701**	**4.858**	**2.976**
Eupodes	Cheiroseius	**0.005**	**0.356**	**2.675**	**2.277**
Eupodes	Dendrolaelaps	**0.005**	**0.027**	**2.675**	**1.976**
Eupodes	Lysigamasus	**0.005**	**0.407**	**2.675**	**2.821**
Eupodes	Pergamasus	**0.005**	**1.081**	**2.675**	**2.277**
Eupodes	Uropoda	**0.005**	**0.481**	**2.675**	**1.976**
Eupodes	Dorylaimoidea	**0.005**	**-0.604**	**2.675**	**4.858**
Eupodes	Eupodes	**0.005**	**0.005**	**2.675**	**2.675**
Eupodes	Mesostigmata	**0.005**	**-0.411**	**2.675**	**1.976**
Eupodes	Scutacarus	**0.005**	**-0.608**	**2.675**	**3.391**
Eupodes	Tarsonemus	**0.005**	**-0.701**	**2.675**	**2.976**
Mesostigmata	Cheiroseius	**-0.411**	**0.356**	**1.976**	**2.277**

Mesostigmata	Dendrolaelaps	**-0.411**	**0.027**	**1.976**	**1.976**
Mesostigmata	Lysigamasus	**-0.411**	**0.407**	**1.976**	**2.821**
Mesostigmata	Pergamasus	**-0.411**	**1.081**	**1.976**	**2.277**
Mesostigmata	Uropoda	**-0.411**	**0.481**	**1.976**	**1.976**
Mesostigmata	Dorylaimoidea	**-0.411**	**-0.604**	**1.976**	**4.858**
Mesostigmata	Eupodes	**-0.411**	**0.005**	**1.976**	**2.675**
Mesostigmata	Mesostigmata	**-0.411**	**-0.411**	**1.976**	**1.976**
Mesostigmata	Scutacarus	**-0.411**	**-0.608**	**1.976**	**3.391**
Mesostigmata	Tarsonemus	**-0.411**	**-0.701**	**1.976**	**2.976**
Scutacarus	Cheiroseius	**-0.608**	**0.356**	**3.391**	**2.277**
Scutacarus	Dendrolaelaps	**-0.608**	**0.027**	**3.391**	**1.976**
Scutacarus	Lysigamasus	**-0.608**	**0.407**	**3.391**	**2.821**
Scutacarus	Pergamasus	**-0.608**	**1.081**	**3.391**	**2.277**
Scutacarus	Uropoda	**-0.608**	**0.481**	**3.391**	**1.976**
Scutacarus	Dorylaimoidea	**-0.608**	**-0.604**	**3.391**	**4.858**
Scutacarus	Eupodes	**-0.608**	**0.005**	**3.391**	**2.675**
Scutacarus	Mesostigmata	**-0.608**	**-0.411**	**3.391**	**1.976**
Scutacarus	Scutacarus	**-0.608**	**-0.608**	**3.391**	**3.391**
Scutacarus	Tarsonemus	**-0.608**	**-0.701**	**3.391**	**2.976**
Tarsonemus	Cheiroseius	**-0.701**	**0.356**	**2.976**	**2.277**
Tarsonemus	Dendrolaelaps	**-0.701**	**0.027**	**2.976**	**1.976**
Tarsonemus	Lysigamasus	**-0.701**	**0.407**	**2.976**	**2.821**
Tarsonemus	Pergamasus	**-0.701**	**1.081**	**2.976**	**2.277**
Tarsonemus	Uropoda	**-0.701**	**0.481**	**2.976**	**1.976**
Tarsonemus	Dorylaimoidea	**-0.701**	**-0.604**	**2.976**	**4.858**
Tarsonemus	Eupodes	**-0.701**	**0.005**	**2.976**	**2.675**
Tarsonemus	Mesostigmata	**-0.701**	**-0.411**	**2.976**	**1.976**
Tarsonemus	Scutacarus	**-0.701**	**-0.608**	**2.976**	**3.391**
Tarsonemus	Tarsonemus	**-0.701**	**-0.701**	**2.976**	**2.976**
Pyemotes	Eupodes	**-0.608**	**0.005**	**2.578**	**2.675**
Pyemotes	Mesostigmata	**-0.608**	**-0.411**	**2.578**	**1.976**
Pyemotes	Scutacarus	**-0.608**	**-0.608**	**2.578**	**3.391**
Pyemotes	Tarsonemus	**-0.608**	**-0.701**	**2.578**	**2.976**

Resource	Consumer	Mres	Mconsumer	Nres	Nconsumer
Aglenchus	Alliphis	**-1.053**	**0.053**	**4.238**	**2.879**
Aglenchus	Arctoseius	**-1.053**	**-0.152**	**4.238**	**2.453**
Aglenchus	Cheiroseius	**-1.053**	**0.356**	**4.238**	**2.277**
Aglenchus	Dendrolaelaps	**-1.053**	**0.027**	**4.238**	**3.438**
Aglenchus	Hypoaspis	**-1.053**	**0.334**	**4.238**	**2.930**
Aglenchus	Parasitus	**-1.053**	**0.859**	**4.238**	**3.017**
Aglenchus	Pergamasus	**-1.053**	**1.081**	**4.238**	**2.453**
Aglenchus	Rhodacarellus	**-1.053**	**-0.310**	**4.238**	**1.976**
Aglenchus	Aporcelaimellus	**-1.053**	**0.548**	**4.238**	**4.238**
Aglenchus	Dorylaimoidea	**-1.053**	**-0.604**	**4.238**	**4.636**
Aglenchus	Mesostigmata	**-1.053**	**-0.411**	**4.238**	**2.578**
Aglenchus	Scutacarus	**-1.053**	**-0.608**	**4.238**	**3.374**
Aglenchus	Stigmaeidae	**-1.053**	**0.229**	**4.238**	**2.675**
Dolichodoridae	Alliphis	**-0.885**	**0.053**	**4.238**	**2.879**
Dolichodoridae	Arctoseius	**-0.885**	**-0.152**	**4.238**	**2.453**
Dolichodoridae	Cheiroseius	**-0.885**	**0.356**	**4.238**	**2.277**
Dolichodoridae	Dendrolaelaps	**-0.885**	**0.027**	**4.238**	**3.438**
Dolichodoridae	Hypoaspis	**-0.885**	**0.334**	**4.238**	**2.930**
Dolichodoridae	Parasitus	**-0.885**	**0.859**	**4.238**	**3.017**
Dolichodoridae	Pergamasus	**-0.885**	**1.081**	**4.238**	**2.453**
Dolichodoridae	Rhodacarellus	**-0.885**	**-0.310**	**4.238**	**1.976**
Dolichodoridae	Aporcelaimellus	**-0.885**	**0.548**	**4.238**	**4.238**
Dolichodoridae	Dorylaimoidea	**-0.885**	**-0.604**	**4.238**	**4.636**
Dolichodoridae	Mesostigmata	**-0.885**	**-0.411**	**4.238**	**2.578**
Dolichodoridae	Scutacarus	**-0.885**	**-0.608**	**4.238**	**3.374**
Dolichodoridae	Stigmaeidae	**-0.885**	**0.229**	**4.238**	**2.675**
Helicotylenchus	Alliphis	**-0.792**	**0.053**	**3.937**	**2.879**
Helicotylenchus	Arctoseius	**-0.792**	**-0.152**	**3.937**	**2.453**
Helicotylenchus	Cheiroseius	**-0.792**	**0.356**	**3.937**	**2.277**
Helicotylenchus	Dendrolaelaps	**-0.792**	**0.027**	**3.937**	**3.438**
Helicotylenchus	Hypoaspis	**-0.792**	**0.334**	**3.937**	**2.930**
Helicotylenchus	Parasitus	**-0.792**	**0.859**	**3.937**	**3.017**
Helicotylenchus	Pergamasus	**-0.792**	**1.081**	**3.937**	**2.453**
Helicotylenchus	Rhodacarellus	**-0.792**	**-0.310**	**3.937**	**1.976**
Helicotylenchus	Aporcelaimellus	**-0.792**	**0.548**	**3.937**	**4.238**
Helicotylenchus	Dorylaimoidea	**-0.792**	**-0.604**	**3.937**	**4.636**
Helicotylenchus	Mesostigmata	**-0.792**	**-0.411**	**3.937**	**2.578**
Helicotylenchus	Scutacarus	**-0.792**	**-0.608**	**3.937**	**3.374**
Helicotylenchus	Stigmaeidae	**-0.792**	**0.229**	**3.937**	**2.675**
Hoplolaimidae	Alliphis	**-1.090**	**0.053**	**3.937**	**2.879**
Hoplolaimidae	Arctoseius	**-1.090**	**-0.152**	**3.937**	**2.453**
Hoplolaimidae	Cheiroseius	**-1.090**	**0.356**	**3.937**	**2.277**
Hoplolaimidae	Dendrolaelaps	**-1.090**	**0.027**	**3.937**	**3.438**
Hoplolaimidae	Hypoaspis	**-1.090**	**0.334**	**3.937**	**2.930**
Hoplolaimidae	Parasitus	**-1.090**	**0.859**	**3.937**	**3.017**
Hoplolaimidae	Pergamasus	**-1.090**	**1.081**	**3.937**	**2.453**
Hoplolaimidae	Rhodacarellus	**-1.090**	**-0.310**	**3.937**	**1.976**
Hoplolaimidae	Aporcelaimellus	**-1.090**	**0.548**	**3.937**	**4.238**
Hoplolaimidae	Dorylaimoidea	**-1.090**	**-0.604**	**3.937**	**4.636**
Hoplolaimidae	Mesostigmata	**-1.090**	**-0.411**	**3.937**	**2.578**

Hoplolaimidae	Scutacarus	**-1.090**	**-0.608**	**3.937**	**3.374**
Hoplolaimidae	Stigmaeidae	**-1.090**	**0.229**	**3.937**	**2.675**
Malenchus	Alliphis	**-1.330**	**0.053**	**4.891**	**2.879**
Malenchus	Arctoseius	**-1.330**	**-0.152**	**4.891**	**2.453**
Malenchus	Cheiroseius	**-1.330**	**0.356**	**4.891**	**2.277**
Malenchus	Dendrolaelaps	**-1.330**	**0.027**	**4.891**	**3.438**
Malenchus	Hypoaspis	**-1.330**	**0.334**	**4.891**	**2.930**
Malenchus	Parasitus	**-1.330**	**0.859**	**4.891**	**3.017**
Malenchus	Pergamasus	**-1.330**	**1.081**	**4.891**	**2.453**
Malenchus	Rhodacarellus	**-1.330**	**-0.310**	**4.891**	**1.976**
Malenchus	Aporcelaimellus	**-1.330**	**0.548**	**4.891**	**4.238**
Malenchus	Dorylaimoidea	**-1.330**	**-0.604**	**4.891**	**4.636**
Malenchus	Mesostigmata	**-1.330**	**-0.411**	**4.891**	**2.578**
Malenchus	Scutacarus	**-1.330**	**-0.608**	**4.891**	**3.374**
Malenchus	Stigmaeidae	**-1.330**	**0.229**	**4.891**	**2.675**
Paratrichodorus	Alliphis	**-0.630**	**0.053**	**3.937**	**2.879**
Paratrichodorus	Arctoseius	**-0.630**	**-0.152**	**3.937**	**2.453**
Paratrichodorus	Cheiroseius	**-0.630**	**0.356**	**3.937**	**2.277**
Paratrichodorus	Dendrolaelaps	**-0.630**	**0.027**	**3.937**	**3.438**
Paratrichodorus	Hypoaspis	**-0.630**	**0.334**	**3.937**	**2.930**
Paratrichodorus	Parasitus	**-0.630**	**0.859**	**3.937**	**3.017**
Paratrichodorus	Pergamasus	**-0.630**	**1.081**	**3.937**	**2.453**
Paratrichodorus	Rhodacarellus	**-0.630**	**-0.310**	**3.937**	**1.976**
Paratrichodorus	Aporcelaimellus	**-0.630**	**0.548**	**3.937**	**4.238**
Paratrichodorus	Dorylaimoidea	**-0.630**	**-0.604**	**3.937**	**4.636**
Paratrichodorus	Mesostigmata	**-0.630**	**-0.411**	**3.937**	**2.578**
Paratrichodorus	Scutacarus	**-0.630**	**-0.608**	**3.937**	**3.374**
Paratrichodorus	Stigmaeidae	**-0.630**	**0.229**	**3.937**	**2.675**
Paratylenchus	Alliphis	**-1.244**	**0.053**	**4.539**	**2.879**
Paratylenchus	Arctoseius	**-1.244**	**-0.152**	**4.539**	**2.453**
Paratylenchus	Cheiroseius	**-1.244**	**0.356**	**4.539**	**2.277**
Paratylenchus	Dendrolaelaps	**-1.244**	**0.027**	**4.539**	**3.438**
Paratylenchus	Hypoaspis	**-1.244**	**0.334**	**4.539**	**2.930**
Paratylenchus	Parasitus	**-1.244**	**0.859**	**4.539**	**3.017**
Paratylenchus	Pergamasus	**-1.244**	**1.081**	**4.539**	**2.453**
Paratylenchus	Rhodacarellus	**-1.244**	**-0.310**	**4.539**	**1.976**
Paratylenchus	Aporcelaimellus	**-1.244**	**0.548**	**4.539**	**4.238**
Paratylenchus	Dorylaimoidea	**-1.244**	**-0.604**	**4.539**	**4.636**
Paratylenchus	Mesostigmata	**-1.244**	**-0.411**	**4.539**	**2.578**
Paratylenchus	Scutacarus	**-1.244**	**-0.608**	**4.539**	**3.374**
Paratylenchus	Stigmaeidae	**-1.244**	**0.229**	**4.539**	**2.675**
Pratylenchus	Alliphis	**-1.226**	**0.053**	**4.636**	**2.879**
Pratylenchus	Arctoseius	**-1.226**	**-0.152**	**4.636**	**2.453**
Pratylenchus	Cheiroseius	**-1.226**	**0.356**	**4.636**	**2.277**
Pratylenchus	Dendrolaelaps	**-1.226**	**0.027**	**4.636**	**3.438**
Pratylenchus	Hypoaspis	**-1.226**	**0.334**	**4.636**	**2.930**
Pratylenchus	Parasitus	**-1.226**	**0.859**	**4.636**	**3.017**
Pratylenchus	Pergamasus	**-1.226**	**1.081**	**4.636**	**2.453**
Pratylenchus	Rhodacarellus	**-1.226**	**-0.310**	**4.636**	**1.976**
Pratylenchus	Aporcelaimellus	**-1.226**	**0.548**	**4.636**	**4.238**
Pratylenchus	Dorylaimoidea	**-1.226**	**-0.604**	**4.636**	**4.636**

Pratylenchus	Mesostigmata	**-1.226**	**-0.411**	**4.636**	**2.578**
Pratylenchus	Scutacarus	**-1.226**	**-0.608**	**4.636**	**3.374**
Pratylenchus	Stigmaeidae	**-1.226**	**0.229**	**4.636**	**2.675**
Tylenchorhynchus	Alliphis	**-0.664**	**0.053**	**3.937**	**2.879**
Tylenchorhynchus	Arctoseius	**-0.664**	**-0.152**	**3.937**	**2.453**
Tylenchorhynchus	Cheiroseius	**-0.664**	**0.356**	**3.937**	**2.277**
Tylenchorhynchus	Dendrolaelaps	**-0.664**	**0.027**	**3.937**	**3.438**
Tylenchorhynchus	Hypoaspis	**-0.664**	**0.334**	**3.937**	**2.930**
Tylenchorhynchus	Parasitus	**-0.664**	**0.859**	**3.937**	**3.017**
Tylenchorhynchus	Pergamasus	**-0.664**	**1.081**	**3.937**	**2.453**
Tylenchorhynchus	Rhodacarellus	**-0.664**	**-0.310**	**3.937**	**1.976**
Tylenchorhynchus	Aporcelaimellus	**-0.664**	**0.548**	**3.937**	**4.238**
Tylenchorhynchus	Dorylaimoidea	**-0.664**	**-0.604**	**3.937**	**4.636**
Tylenchorhynchus	Mesostigmata	**-0.664**	**-0.411**	**3.937**	**2.578**
Tylenchorhynchus	Scutacarus	**-0.664**	**-0.608**	**3.937**	**3.374**
Tylenchorhynchus	Stigmaeidae	**-0.664**	**0.229**	**3.937**	**2.675**
Tydeidae	Arctoseius	**-0.608**	**-0.152**	**1.976**	**2.453**
Tydeidae	Cheiroseius	**-0.608**	**0.356**	**1.976**	**2.277**
Tydeidae	Dendrolaelaps	**-0.608**	**0.027**	**1.976**	**3.438**
Tydeidae	Hypoaspis	**-0.608**	**0.334**	**1.976**	**2.930**
Tydeidae	Parasitus	**-0.608**	**0.859**	**1.976**	**3.017**
Tydeidae	Pergamasus	**-0.608**	**1.081**	**1.976**	**2.453**
Tydeidae	Rhodacarellus	**-0.608**	**-0.310**	**1.976**	**1.976**
Tydeidae	Aporcelaimellus	**-0.608**	**0.548**	**1.976**	**4.238**
Tydeidae	Dorylaimoidea	**-0.608**	**-0.604**	**1.976**	**4.636**
Tydeidae	Mesostigmata	**-0.608**	**-0.411**	**1.976**	**2.578**
Tydeidae	Scutacarus	**-0.608**	**-0.608**	**1.976**	**3.374**
Tydeidae	Stigmaeidae	**-0.608**	**0.229**	**1.976**	**2.675**
Sminthuridae	Arctoseius	**-0.608**	**-0.152**	**2.675**	**2.453**
Sminthuridae	Cheiroseius	**-0.608**	**0.356**	**2.675**	**2.277**
Sminthuridae	Dendrolaelaps	**-0.608**	**0.027**	**2.675**	**3.438**
Sminthuridae	Hypoaspis	**-0.608**	**0.334**	**2.675**	**2.930**
Sminthuridae	Parasitus	**-0.608**	**0.859**	**2.675**	**3.017**
Sminthuridae	Pergamasus	**-0.608**	**1.081**	**2.675**	**2.453**
Sminthuridae	Rhodacarellus	**-0.608**	**-0.310**	**2.675**	**1.976**
Sminthuridae	Aporcelaimellus	**-0.608**	**0.548**	**2.675**	**4.238**
Sminthuridae	Dorylaimoidea	**-0.608**	**-0.604**	**2.675**	**4.636**
Sminthuridae	Mesostigmata	**-0.608**	**-0.411**	**2.675**	**2.578**
Sminthuridae	Scutacarus	**-0.608**	**-0.608**	**2.675**	**3.374**
Sminthuridae	Stigmaeidae	**-0.608**	**0.229**	**2.675**	**2.675**
Sminthurinus	Arctoseius	**0.618**	**-0.152**	**3.481**	**2.453**
Sminthurinus	Cheiroseius	**0.618**	**0.356**	**3.481**	**2.277**
Sminthurinus	Dendrolaelaps	**0.618**	**0.027**	**3.481**	**3.438**
Sminthurinus	Hypoaspis	**0.618**	**0.334**	**3.481**	**2.930**
Sminthurinus	Parasitus	**0.618**	**0.859**	**3.481**	**3.017**
Sminthurinus	Pergamasus	**0.618**	**1.081**	**3.481**	**2.453**
Sminthurinus	Rhodacarellus	**0.618**	**-0.310**	**3.481**	**1.976**
Sminthurinus	Aporcelaimellus	**0.618**	**0.548**	**3.481**	**4.238**
Sminthurinus	Dorylaimoidea	**0.618**	**-0.604**	**3.481**	**4.636**
Sminthurinus	Mesostigmata	**0.618**	**-0.411**	**3.481**	**2.578**
Sminthurinus	Scutacarus	**0.618**	**-0.608**	**3.481**	**3.374**

Sminthurinus	Stigmaeidae	**0.618**	**0.229**	**3.481**	**2.675**
Sminthurus	Arctoseius	**1.429**	**-0.152**	**3.055**	**2.453**
Sminthurus	Cheiroseius	**1.429**	**0.356**	**3.055**	**2.277**
Sminthurus	Dendrolaelaps	**1.429**	**0.027**	**3.055**	**3.438**
Sminthurus	Hypoaspis	**1.429**	**0.334**	**3.055**	**2.930**
Sminthurus	Parasitus	**1.429**	**0.859**	**3.055**	**3.017**
Sminthurus	Pergamasus	**1.429**	**1.081**	**3.055**	**2.453**
Sminthurus	Rhodacarellus	**1.429**	**-0.310**	**3.055**	**1.976**
Sminthurus	Aporcelaimellus	**1.429**	**0.548**	**3.055**	**4.238**
Sminthurus	Dorylaimoidea	**1.429**	**-0.604**	**3.055**	**4.636**
Sminthurus	Mesostigmata	**1.429**	**-0.411**	**3.055**	**2.578**
Sminthurus	Scutacarus	**1.429**	**-0.608**	**3.055**	**3.374**
Sminthurus	Stigmaeidae	**1.429**	**0.229**	**3.055**	**2.675**
Sphaeridia	Arctoseius	**0.202**	**-0.152**	**1.976**	**2.453**
Sphaeridia	Cheiroseius	**0.202**	**0.356**	**1.976**	**2.277**
Sphaeridia	Dendrolaelaps	**0.202**	**0.027**	**1.976**	**3.438**
Sphaeridia	Hypoaspis	**0.202**	**0.334**	**1.976**	**2.930**
Sphaeridia	Parasitus	**0.202**	**0.859**	**1.976**	**3.017**
Sphaeridia	Pergamasus	**0.202**	**1.081**	**1.976**	**2.453**
Sphaeridia	Rhodacarellus	**0.202**	**-0.310**	**1.976**	**1.976**
Sphaeridia	Aporcelaimellus	**0.202**	**0.548**	**1.976**	**4.238**
Sphaeridia	Dorylaimoidea	**0.202**	**-0.604**	**1.976**	**4.636**
Sphaeridia	Mesostigmata	**0.202**	**-0.411**	**1.976**	**2.578**
Sphaeridia	Scutacarus	**0.202**	**-0.608**	**1.976**	**3.374**
Sphaeridia	Stigmaeidae	**0.202**	**0.229**	**1.976**	**2.675**
Achromadora	Alliphis	**-1.396**	**0.053**	**3.937**	**2.879**
Achromadora	Arctoseius	**-1.396**	**-0.152**	**3.937**	**2.453**
Achromadora	Cheiroseius	**-1.396**	**0.356**	**3.937**	**2.277**
Achromadora	Dendrolaelaps	**-1.396**	**0.027**	**3.937**	**3.438**
Achromadora	Hypoaspis	**-1.396**	**0.334**	**3.937**	**2.930**
Achromadora	Parasitus	**-1.396**	**0.859**	**3.937**	**3.017**
Achromadora	Pergamasus	**-1.396**	**1.081**	**3.937**	**2.453**
Achromadora	Rhodacarellus	**-1.396**	**-0.310**	**3.937**	**1.976**
Achromadora	Aporcelaimellus	**-1.396**	**0.548**	**3.937**	**4.238**
Achromadora	Dorylaimoidea	**-1.396**	**-0.604**	**3.937**	**4.636**
Achromadora	Mesostigmata	**-1.396**	**-0.411**	**3.937**	**2.578**
Achromadora	Scutacarus	**-1.396**	**-0.608**	**3.937**	**3.374**
Achromadora	Stigmaeidae	**-1.396**	**0.229**	**3.937**	**2.675**
Aphelenchoides	Alliphis	**-1.496**	**0.053**	**3.937**	**2.879**
Aphelenchoides	Arctoseius	**-1.496**	**-0.152**	**3.937**	**2.453**
Aphelenchoides	Cheiroseius	**-1.496**	**0.356**	**3.937**	**2.277**
Aphelenchoides	Dendrolaelaps	**-1.496**	**0.027**	**3.937**	**3.438**
Aphelenchoides	Hypoaspis	**-1.496**	**0.334**	**3.937**	**2.930**
Aphelenchoides	Parasitus	**-1.496**	**0.859**	**3.937**	**3.017**
Aphelenchoides	Pergamasus	**-1.496**	**1.081**	**3.937**	**2.453**
Aphelenchoides	Rhodacarellus	**-1.496**	**-0.310**	**3.937**	**1.976**
Aphelenchoides	Aporcelaimellus	**-1.496**	**0.548**	**3.937**	**4.238**
Aphelenchoides	Dorylaimoidea	**-1.496**	**-0.604**	**3.937**	**4.636**
Aphelenchoides	Mesostigmata	**-1.496**	**-0.411**	**3.937**	**2.578**
Aphelenchoides	Scutacarus	**-1.496**	**-0.608**	**3.937**	**3.374**
Aphelenchoides	Stigmaeidae	**-1.496**	**0.229**	**3.937**	**2.675**

Tylenchidae	Alliphis	**-1.360**	**0.053**	**4.238**	**2.879**
Tylenchidae	Arctoseius	**-1.360**	**-0.152**	**4.238**	**2.453**
Tylenchidae	Cheiroseius	**-1.360**	**0.356**	**4.238**	**2.277**
Tylenchidae	Dendrolaelaps	**-1.360**	**0.027**	**4.238**	**3.438**
Tylenchidae	Hypoaspis	**-1.360**	**0.334**	**4.238**	**2.930**
Tylenchidae	Parasitus	**-1.360**	**0.859**	**4.238**	**3.017**
Tylenchidae	Pergamasus	**-1.360**	**1.081**	**4.238**	**2.453**
Tylenchidae	Rhodacarellus	**-1.360**	**-0.310**	**4.238**	**1.976**
Tylenchidae	Aporcelaimellus	**-1.360**	**0.548**	**4.238**	**4.238**
Tylenchidae	Dorylaimoidea	**-1.360**	**-0.604**	**4.238**	**4.636**
Tylenchidae	Mesostigmata	**-1.360**	**-0.411**	**4.238**	**2.578**
Tylenchidae	Scutacarus	**-1.360**	**-0.608**	**4.238**	**3.374**
Tylenchidae	Stigmaeidae	**-1.360**	**0.229**	**4.238**	**2.675**
Oppiella	Arctoseius	**-0.447**	**-0.152**	**2.675**	**2.453**
Oppiella	Cheiroseius	**-0.447**	**0.356**	**2.675**	**2.277**
Oppiella	Dendrolaelaps	**-0.447**	**0.027**	**2.675**	**3.438**
Oppiella	Hypoaspis	**-0.447**	**0.334**	**2.675**	**2.930**
Oppiella	Parasitus	**-0.447**	**0.859**	**2.675**	**3.017**
Oppiella	Pergamasus	**-0.447**	**1.081**	**2.675**	**2.453**
Oppiella	Rhodacarellus	**-0.447**	**-0.310**	**2.675**	**1.976**
Oppiella	Aporcelaimellus	**-0.447**	**0.548**	**2.675**	**4.238**
Oppiella	Dorylaimoidea	**-0.447**	**-0.604**	**2.675**	**4.636**
Oppiella	Mesostigmata	**-0.447**	**-0.411**	**2.675**	**2.578**
Oppiella	Scutacarus	**-0.447**	**-0.608**	**2.675**	**3.374**
Oppiella	Stigmaeidae	**-0.447**	**0.229**	**2.675**	**2.675**
Pygmephorus	Arctoseius	**-0.376**	**-0.152**	**2.578**	**2.453**
Pygmephorus	Cheiroseius	**-0.376**	**0.356**	**2.578**	**2.277**
Pygmephorus	Dendrolaelaps	**-0.376**	**0.027**	**2.578**	**3.438**
Pygmephorus	Hypoaspis	**-0.376**	**0.334**	**2.578**	**2.930**
Pygmephorus	Parasitus	**-0.376**	**0.859**	**2.578**	**3.017**
Pygmephorus	Pergamasus	**-0.376**	**1.081**	**2.578**	**2.453**
Pygmephorus	Rhodacarellus	**-0.376**	**-0.310**	**2.578**	**1.976**
Pygmephorus	Aporcelaimellus	**-0.376**	**0.548**	**2.578**	**4.238**
Pygmephorus	Dorylaimoidea	**-0.376**	**-0.604**	**2.578**	**4.636**
Pygmephorus	Mesostigmata	**-0.376**	**-0.411**	**2.578**	**2.578**
Pygmephorus	Scutacarus	**-0.376**	**-0.608**	**2.578**	**3.374**
Pygmephorus	Stigmaeidae	**-0.376**	**0.229**	**2.578**	**2.675**
Tyrophagus	Arctoseius	**0.005**	**-0.152**	**1.976**	**2.453**
Tyrophagus	Cheiroseius	**0.005**	**0.356**	**1.976**	**2.277**
Tyrophagus	Dendrolaelaps	**0.005**	**0.027**	**1.976**	**3.438**
Tyrophagus	Hypoaspis	**0.005**	**0.334**	**1.976**	**2.930**
Tyrophagus	Parasitus	**0.005**	**0.859**	**1.976**	**3.017**
Tyrophagus	Pergamasus	**0.005**	**1.081**	**1.976**	**2.453**
Tyrophagus	Rhodacarellus	**0.005**	**-0.310**	**1.976**	**1.976**
Tyrophagus	Aporcelaimellus	**0.005**	**0.548**	**1.976**	**4.238**
Tyrophagus	Dorylaimoidea	**0.005**	**-0.604**	**1.976**	**4.636**
Tyrophagus	Mesostigmata	**0.005**	**-0.411**	**1.976**	**2.578**
Tyrophagus	Scutacarus	**0.005**	**-0.608**	**1.976**	**3.374**
Tyrophagus	Stigmaeidae	**0.005**	**0.229**	**1.976**	**2.675**
Folsomia	Arctoseius	**0.900**	**-0.152**	**2.277**	**2.453**
Folsomia	Cheiroseius	**0.900**	**0.356**	**2.277**	**2.277**

Folsomia	Dendrolaelaps	**0.900**	**0.027**	**2.277**	**3.438**
Folsomia	Hypoaspis	**0.900**	**0.334**	**2.277**	**2.930**
Folsomia	Parasitus	**0.900**	**0.859**	**2.277**	**3.017**
Folsomia	Pergamasus	**0.900**	**1.081**	**2.277**	**2.453**
Folsomia	Rhodacarellus	**0.900**	**-0.310**	**2.277**	**1.976**
Folsomia	Aporcelaimellus	**0.900**	**0.548**	**2.277**	**4.238**
Folsomia	Dorylaimoidea	**0.900**	**-0.604**	**2.277**	**4.636**
Folsomia	Mesostigmata	**0.900**	**-0.411**	**2.277**	**2.578**
Folsomia	Scutacarus	**0.900**	**-0.608**	**2.277**	**3.374**
Folsomia	Stigmaeidae	**0.900**	**0.229**	**2.277**	**2.675**
Friesea	Arctoseius	**0.434**	**-0.152**	**1.976**	**2.453**
Friesea	Cheiroseius	**0.434**	**0.356**	**1.976**	**2.277**
Friesea	Dendrolaelaps	**0.434**	**0.027**	**1.976**	**3.438**
Friesea	Hypoaspis	**0.434**	**0.334**	**1.976**	**2.930**
Friesea	Parasitus	**0.434**	**0.859**	**1.976**	**3.017**
Friesea	Pergamasus	**0.434**	**1.081**	**1.976**	**2.453**
Friesea	Rhodacarellus	**0.434**	**-0.310**	**1.976**	**1.976**
Friesea	Aporcelaimellus	**0.434**	**0.548**	**1.976**	**4.238**
Friesea	Dorylaimoidea	**0.434**	**-0.604**	**1.976**	**4.636**
Friesea	Mesostigmata	**0.434**	**-0.411**	**1.976**	**2.578**
Friesea	Scutacarus	**0.434**	**-0.608**	**1.976**	**3.374**
Friesea	Stigmaeidae	**0.434**	**0.229**	**1.976**	**2.675**
Hypogastrura	Arctoseius	**0.977**	**-0.152**	**2.453**	**2.453**
Hypogastrura	Cheiroseius	**0.977**	**0.356**	**2.453**	**2.277**
Hypogastrura	Dendrolaelaps	**0.977**	**0.027**	**2.453**	**3.438**
Hypogastrura	Hypoaspis	**0.977**	**0.334**	**2.453**	**2.930**
Hypogastrura	Parasitus	**0.977**	**0.859**	**2.453**	**3.017**
Hypogastrura	Pergamasus	**0.977**	**1.081**	**2.453**	**2.453**
Hypogastrura	Rhodacarellus	**0.977**	**-0.310**	**2.453**	**1.976**
Hypogastrura	Aporcelaimellus	**0.977**	**0.548**	**2.453**	**4.238**
Hypogastrura	Dorylaimoidea	**0.977**	**-0.604**	**2.453**	**4.636**
Hypogastrura	Mesostigmata	**0.977**	**-0.411**	**2.453**	**2.578**
Hypogastrura	Scutacarus	**0.977**	**-0.608**	**2.453**	**3.374**
Hypogastrura	Stigmaeidae	**0.977**	**0.229**	**2.453**	**2.675**
Isotoma	Arctoseius	**1.898**	**-0.152**	**3.657**	**2.453**
Isotoma	Cheiroseius	**1.898**	**0.356**	**3.657**	**2.277**
Isotoma	Dendrolaelaps	**1.898**	**0.027**	**3.657**	**3.438**
Isotoma	Hypoaspis	**1.898**	**0.334**	**3.657**	**2.930**
Isotoma	Parasitus	**1.898**	**0.859**	**3.657**	**3.017**
Isotoma	Pergamasus	**1.898**	**1.081**	**3.657**	**2.453**
Isotoma	Rhodacarellus	**1.898**	**-0.310**	**3.657**	**1.976**
Isotoma	Aporcelaimellus	**1.898**	**0.548**	**3.657**	**4.238**
Isotoma	Dorylaimoidea	**1.898**	**-0.604**	**3.657**	**4.636**
Isotoma	Mesostigmata	**1.898**	**-0.411**	**3.657**	**2.578**
Isotoma	Scutacarus	**1.898**	**-0.608**	**3.657**	**3.374**
Isotoma	Stigmaeidae	**1.898**	**0.229**	**3.657**	**2.675**
Isotomurus	Arctoseius	**1.787**	**-0.152**	**2.930**	**2.453**
Isotomurus	Cheiroseius	**1.787**	**0.356**	**2.930**	**2.277**
Isotomurus	Dendrolaelaps	**1.787**	**0.027**	**2.930**	**3.438**
Isotomurus	Hypoaspis	**1.787**	**0.334**	**2.930**	**2.930**
Isotomurus	Parasitus	**1.787**	**0.859**	**2.930**	**3.017**

Isotomurus	Pergamasus	**1.787**	**1.081**	**2.930**	**2.453**
Isotomurus	Rhodacarellus	**1.787**	**-0.310**	**2.930**	**1.976**
Isotomurus	Aporcelaimellus	**1.787**	**0.548**	**2.930**	**4.238**
Isotomurus	Dorylaimoidea	**1.787**	**-0.604**	**2.930**	**4.636**
Isotomurus	Mesostigmata	**1.787**	**-0.411**	**2.930**	**2.578**
Isotomurus	Scutacarus	**1.787**	**-0.608**	**2.930**	**3.374**
Isotomurus	Stigmaeidae	**1.787**	**0.229**	**2.930**	**2.675**
Lepidocyrtus	Arctoseius	**1.231**	**-0.152**	**2.675**	**2.453**
Lepidocyrtus	Cheiroseius	**1.231**	**0.356**	**2.675**	**2.277**
Lepidocyrtus	Dendrolaelaps	**1.231**	**0.027**	**2.675**	**3.438**
Lepidocyrtus	Hypoaspis	**1.231**	**0.334**	**2.675**	**2.930**
Lepidocyrtus	Parasitus	**1.231**	**0.859**	**2.675**	**3.017**
Lepidocyrtus	Pergamasus	**1.231**	**1.081**	**2.675**	**2.453**
Lepidocyrtus	Rhodacarellus	**1.231**	**-0.310**	**2.675**	**1.976**
Lepidocyrtus	Aporcelaimellus	**1.231**	**0.548**	**2.675**	**4.238**
Lepidocyrtus	Dorylaimoidea	**1.231**	**-0.604**	**2.675**	**4.636**
Lepidocyrtus	Mesostigmata	**1.231**	**-0.411**	**2.675**	**2.578**
Lepidocyrtus	Scutacarus	**1.231**	**-0.608**	**2.675**	**3.374**
Lepidocyrtus	Stigmaeidae	**1.231**	**0.229**	**2.675**	**2.675**
Mesaphorura	Arctoseius	**0.618**	**-0.152**	**1.976**	**2.453**
Mesaphorura	Cheiroseius	**0.618**	**0.356**	**1.976**	**2.277**
Mesaphorura	Dendrolaelaps	**0.618**	**0.027**	**1.976**	**3.438**
Mesaphorura	Hypoaspis	**0.618**	**0.334**	**1.976**	**2.930**
Mesaphorura	Parasitus	**0.618**	**0.859**	**1.976**	**3.017**
Mesaphorura	Pergamasus	**0.618**	**1.081**	**1.976**	**2.453**
Mesaphorura	Rhodacarellus	**0.618**	**-0.310**	**1.976**	**1.976**
Mesaphorura	Aporcelaimellus	**0.618**	**0.548**	**1.976**	**4.238**
Mesaphorura	Dorylaimoidea	**0.618**	**-0.604**	**1.976**	**4.636**
Mesaphorura	Mesostigmata	**0.618**	**-0.411**	**1.976**	**2.578**
Mesaphorura	Scutacarus	**0.618**	**-0.608**	**1.976**	**3.374**
Mesaphorura	Stigmaeidae	**0.618**	**0.229**	**1.976**	**2.675**
Proisotoma	Arctoseius	**0.770**	**-0.152**	**1.976**	**2.453**
Proisotoma	Cheiroseius	**0.770**	**0.356**	**1.976**	**2.277**
Proisotoma	Dendrolaelaps	**0.770**	**0.027**	**1.976**	**3.438**
Proisotoma	Hypoaspis	**0.770**	**0.334**	**1.976**	**2.930**
Proisotoma	Parasitus	**0.770**	**0.859**	**1.976**	**3.017**
Proisotoma	Pergamasus	**0.770**	**1.081**	**1.976**	**2.453**
Proisotoma	Rhodacarellus	**0.770**	**-0.310**	**1.976**	**1.976**
Proisotoma	Aporcelaimellus	**0.770**	**0.548**	**1.976**	**4.238**
Proisotoma	Dorylaimoidea	**0.770**	**-0.604**	**1.976**	**4.636**
Proisotoma	Mesostigmata	**0.770**	**-0.411**	**1.976**	**2.578**
Proisotoma	Scutacarus	**0.770**	**-0.608**	**1.976**	**3.374**
Proisotoma	Stigmaeidae	**0.770**	**0.229**	**1.976**	**2.675**
Achaeta	Arctoseius	**1.096**	**-0.152**	**3.462**	**2.453**
Achaeta	Cheiroseius	**1.096**	**0.356**	**3.462**	**2.277**
Achaeta	Dendrolaelaps	**1.096**	**0.027**	**3.462**	**3.438**
Achaeta	Hypoaspis	**1.096**	**0.334**	**3.462**	**2.930**
Achaeta	Parasitus	**1.096**	**0.859**	**3.462**	**3.017**
Achaeta	Pergamasus	**1.096**	**1.081**	**3.462**	**2.453**
Achaeta	Rhodacarellus	**1.096**	**-0.310**	**3.462**	**1.976**
Achaeta	Aporcelaimellus	**1.096**	**0.548**	**3.462**	**4.238**

Achaeta	Dorylaimoidea	**1.096**	**-0.604**	**3.462**	**4.636**
Achaeta	Mesostigmata	**1.096**	**-0.411**	**3.462**	**2.578**
Achaeta	Scutacarus	**1.096**	**-0.608**	**3.462**	**3.374**
Achaeta	Stigmaeidae	**1.096**	**0.229**	**3.462**	**2.675**
Fridericia	Arctoseius	**1.568**	**-0.152**	**4.211**	**2.453**
Fridericia	Cheiroseius	**1.568**	**0.356**	**4.211**	**2.277**
Fridericia	Dendrolaelaps	**1.568**	**0.027**	**4.211**	**3.438**
Fridericia	Hypoaspis	**1.568**	**0.334**	**4.211**	**2.930**
Fridericia	Parasitus	**1.568**	**0.859**	**4.211**	**3.017**
Fridericia	Pergamasus	**1.568**	**1.081**	**4.211**	**2.453**
Fridericia	Rhodacarellus	**1.568**	**-0.310**	**4.211**	**1.976**
Fridericia	Aporcelaimellus	**1.568**	**0.548**	**4.211**	**4.238**
Fridericia	Dorylaimoidea	**1.568**	**-0.604**	**4.211**	**4.636**
Fridericia	Mesostigmata	**1.568**	**-0.411**	**4.211**	**2.578**
Fridericia	Scutacarus	**1.568**	**-0.608**	**4.211**	**3.374**
Fridericia	Stigmaeidae	**1.568**	**0.229**	**4.211**	**2.675**
Acrobeles	Alliphis	**-0.721**	**0.053**	**3.937**	**2.879**
Acrobeles	Arctoseius	**-0.721**	**-0.152**	**3.937**	**2.453**
Acrobeles	Cheiroseius	**-0.721**	**0.356**	**3.937**	**2.277**
Acrobeles	Dendrolaelaps	**-0.721**	**0.027**	**3.937**	**3.438**
Acrobeles	Hypoaspis	**-0.721**	**0.334**	**3.937**	**2.930**
Acrobeles	Parasitus	**-0.721**	**0.859**	**3.937**	**3.017**
Acrobeles	Pergamasus	**-0.721**	**1.081**	**3.937**	**2.453**
Acrobeles	Rhodacarellus	**-0.721**	**-0.310**	**3.937**	**1.976**
Acrobeles	Aporcelaimellus	**-0.721**	**0.548**	**3.937**	**4.238**
Acrobeles	Dorylaimoidea	**-0.721**	**-0.604**	**3.937**	**4.636**
Acrobeles	Mesostigmata	**-0.721**	**-0.411**	**3.937**	**2.578**
Acrobeles	Scutacarus	**-0.721**	**-0.608**	**3.937**	**3.374**
Acrobeles	Stigmaeidae	**-0.721**	**0.229**	**3.937**	**2.675**
Acrobeloides	Alliphis	**-1.171**	**0.053**	**5.141**	**2.879**
Acrobeloides	Arctoseius	**-1.171**	**-0.152**	**5.141**	**2.453**
Acrobeloides	Cheiroseius	**-1.171**	**0.356**	**5.141**	**2.277**
Acrobeloides	Dendrolaelaps	**-1.171**	**0.027**	**5.141**	**3.438**
Acrobeloides	Hypoaspis	**-1.171**	**0.334**	**5.141**	**2.930**
Acrobeloides	Parasitus	**-1.171**	**0.859**	**5.141**	**3.017**
Acrobeloides	Pergamasus	**-1.171**	**1.081**	**5.141**	**2.453**
Acrobeloides	Rhodacarellus	**-1.171**	**-0.310**	**5.141**	**1.976**
Acrobeloides	Aporcelaimellus	**-1.171**	**0.548**	**5.141**	**4.238**
Acrobeloides	Dorylaimoidea	**-1.171**	**-0.604**	**5.141**	**4.636**
Acrobeloides	Mesostigmata	**-1.171**	**-0.411**	**5.141**	**2.578**
Acrobeloides	Scutacarus	**-1.171**	**-0.608**	**5.141**	**3.374**
Acrobeloides	Stigmaeidae	**-1.171**	**0.229**	**5.141**	**2.675**
Cephalobidae	Alliphis	**-1.055**	**0.053**	**3.937**	**2.879**
Cephalobidae	Arctoseius	**-1.055**	**-0.152**	**3.937**	**2.453**
Cephalobidae	Cheiroseius	**-1.055**	**0.356**	**3.937**	**2.277**
Cephalobidae	Dendrolaelaps	**-1.055**	**0.027**	**3.937**	**3.438**
Cephalobidae	Hypoaspis	**-1.055**	**0.334**	**3.937**	**2.930**
Cephalobidae	Parasitus	**-1.055**	**0.859**	**3.937**	**3.017**
Cephalobidae	Pergamasus	**-1.055**	**1.081**	**3.937**	**2.453**
Cephalobidae	Rhodacarellus	**-1.055**	**-0.310**	**3.937**	**1.976**
Cephalobidae	Aporcelaimellus	**-1.055**	**0.548**	**3.937**	**4.238**

Cephalobidae	Dorylaimoidea	**-1.055**	**-0.604**	**3.937**	**4.636**
Cephalobidae	Mesostigmata	**-1.055**	**-0.411**	**3.937**	**2.578**
Cephalobidae	Scutacarus	**-1.055**	**-0.608**	**3.937**	**3.374**
Cephalobidae	Stigmaeidae	**-1.055**	**0.229**	**3.937**	**2.675**
Eucephalobus	Alliphis	**-0.855**	**0.053**	**5.299**	**2.879**
Eucephalobus	Arctoseius	**-0.855**	**-0.152**	**5.299**	**2.453**
Eucephalobus	Cheiroseius	**-0.855**	**0.356**	**5.299**	**2.277**
Eucephalobus	Dendrolaelaps	**-0.855**	**0.027**	**5.299**	**3.438**
Eucephalobus	Hypoaspis	**-0.855**	**0.334**	**5.299**	**2.930**
Eucephalobus	Parasitus	**-0.855**	**0.859**	**5.299**	**3.017**
Eucephalobus	Pergamasus	**-0.855**	**1.081**	**5.299**	**2.453**
Eucephalobus	Rhodacarellus	**-0.855**	**-0.310**	**5.299**	**1.976**
Eucephalobus	Aporcelaimellus	**-0.855**	**0.548**	**5.299**	**4.238**
Eucephalobus	Dorylaimoidea	**-0.855**	**-0.604**	**5.299**	**4.636**
Eucephalobus	Mesostigmata	**-0.855**	**-0.411**	**5.299**	**2.578**
Eucephalobus	Scutacarus	**-0.855**	**-0.608**	**5.299**	**3.374**
Eucephalobus	Stigmaeidae	**-0.855**	**0.229**	**5.299**	**2.675**
Panagrolaimus	Alliphis	**-0.945**	**0.053**	**4.539**	**2.879**
Panagrolaimus	Arctoseius	**-0.945**	**-0.152**	**4.539**	**2.453**
Panagrolaimus	Cheiroseius	**-0.945**	**0.356**	**4.539**	**2.277**
Panagrolaimus	Dendrolaelaps	**-0.945**	**0.027**	**4.539**	**3.438**
Panagrolaimus	Hypoaspis	**-0.945**	**0.334**	**4.539**	**2.930**
Panagrolaimus	Parasitus	**-0.945**	**0.859**	**4.539**	**3.017**
Panagrolaimus	Pergamasus	**-0.945**	**1.081**	**4.539**	**2.453**
Panagrolaimus	Rhodacarellus	**-0.945**	**-0.310**	**4.539**	**1.976**
Panagrolaimus	Aporcelaimellus	**-0.945**	**0.548**	**4.539**	**4.238**
Panagrolaimus	Dorylaimoidea	**-0.945**	**-0.604**	**4.539**	**4.636**
Panagrolaimus	Mesostigmata	**-0.945**	**-0.411**	**4.539**	**2.578**
Panagrolaimus	Scutacarus	**-0.945**	**-0.608**	**4.539**	**3.374**
Panagrolaimus	Stigmaeidae	**-0.945**	**0.229**	**4.539**	**2.675**
Plectus	Alliphis	**-0.583**	**0.053**	**4.414**	**2.879**
Plectus	Arctoseius	**-0.583**	**-0.152**	**4.414**	**2.453**
Plectus	Cheiroseius	**-0.583**	**0.356**	**4.414**	**2.277**
Plectus	Dendrolaelaps	**-0.583**	**0.027**	**4.414**	**3.438**
Plectus	Hypoaspis	**-0.583**	**0.334**	**4.414**	**2.930**
Plectus	Parasitus	**-0.583**	**0.859**	**4.414**	**3.017**
Plectus	Pergamasus	**-0.583**	**1.081**	**4.414**	**2.453**
Plectus	Rhodacarellus	**-0.583**	**-0.310**	**4.414**	**1.976**
Plectus	Aporcelaimellus	**-0.583**	**0.548**	**4.414**	**4.238**
Plectus	Dorylaimoidea	**-0.583**	**-0.604**	**4.414**	**4.636**
Plectus	Mesostigmata	**-0.583**	**-0.411**	**4.414**	**2.578**
Plectus	Scutacarus	**-0.583**	**-0.608**	**4.414**	**3.374**
Plectus	Stigmaeidae	**-0.583**	**0.229**	**4.414**	**2.675**
Prismatolaimus	Alliphis	**-1.280**	**0.053**	**4.238**	**2.879**
Prismatolaimus	Arctoseius	**-1.280**	**-0.152**	**4.238**	**2.453**
Prismatolaimus	Cheiroseius	**-1.280**	**0.356**	**4.238**	**2.277**
Prismatolaimus	Dendrolaelaps	**-1.280**	**0.027**	**4.238**	**3.438**
Prismatolaimus	Hypoaspis	**-1.280**	**0.334**	**4.238**	**2.930**
Prismatolaimus	Parasitus	**-1.280**	**0.859**	**4.238**	**3.017**
Prismatolaimus	Pergamasus	**-1.280**	**1.081**	**4.238**	**2.453**
Prismatolaimus	Rhodacarellus	**-1.280**	**-0.310**	**4.238**	**1.976**

Prismatolaimus	Aporcelaimellus	**-1.280**	**0.548**	**4.238**	**4.238**
Prismatolaimus	Dorylaimoidea	**-1.280**	**-0.604**	**4.238**	**4.636**
Prismatolaimus	Mesostigmata	**-1.280**	**-0.411**	**4.238**	**2.578**
Prismatolaimus	Scutacarus	**-1.280**	**-0.608**	**4.238**	**3.374**
Prismatolaimus	Stigmaeidae	**-1.280**	**0.229**	**4.238**	**2.675**
Rhabditidae	Alliphis	**-0.692**	**0.053**	**5.723**	**2.879**
Rhabditidae	Arctoseius	**-0.692**	**-0.152**	**5.723**	**2.453**
Rhabditidae	Cheiroseius	**-0.692**	**0.356**	**5.723**	**2.277**
Rhabditidae	Dendrolaelaps	**-0.692**	**0.027**	**5.723**	**3.438**
Rhabditidae	Hypoaspis	**-0.692**	**0.334**	**5.723**	**2.930**
Rhabditidae	Parasitus	**-0.692**	**0.859**	**5.723**	**3.017**
Rhabditidae	Pergamasus	**-0.692**	**1.081**	**5.723**	**2.453**
Rhabditidae	Rhodacarellus	**-0.692**	**-0.310**	**5.723**	**1.976**
Rhabditidae	Aporcelaimellus	**-0.692**	**0.548**	**5.723**	**4.238**
Rhabditidae	Dorylaimoidea	**-0.692**	**-0.604**	**5.723**	**4.636**
Rhabditidae	Mesostigmata	**-0.692**	**-0.411**	**5.723**	**2.578**
Rhabditidae	Scutacarus	**-0.692**	**-0.608**	**5.723**	**3.374**
Rhabditidae	Stigmaeidae	**-0.692**	**0.229**	**5.723**	**2.675**
Enchytraeus	Arctoseius	**1.118**	**-0.152**	**4.345**	**2.453**
Enchytraeus	Cheiroseius	**1.118**	**0.356**	**4.345**	**2.277**
Enchytraeus	Dendrolaelaps	**1.118**	**0.027**	**4.345**	**3.438**
Enchytraeus	Hypoaspis	**1.118**	**0.334**	**4.345**	**2.930**
Enchytraeus	Parasitus	**1.118**	**0.859**	**4.345**	**3.017**
Enchytraeus	Pergamasus	**1.118**	**1.081**	**4.345**	**2.453**
Enchytraeus	Rhodacarellus	**1.118**	**-0.310**	**4.345**	**1.976**
Enchytraeus	Aporcelaimellus	**1.118**	**0.548**	**4.345**	**4.238**
Enchytraeus	Dorylaimoidea	**1.118**	**-0.604**	**4.345**	**4.636**
Enchytraeus	Mesostigmata	**1.118**	**-0.411**	**4.345**	**2.578**
Enchytraeus	Scutacarus	**1.118**	**-0.608**	**4.345**	**3.374**
Enchytraeus	Stigmaeidae	**1.118**	**0.229**	**4.345**	**2.675**
Eubacteria	Acrobeles	**-6.726**	**-0.721**	**13.486**	**3.937**
Eubacteria	Acrobeloides	**-6.726**	**-1.171**	**13.486**	**5.141**
Eubacteria	Cephalobidae	**-6.726**	**-1.055**	**13.486**	**3.937**
Eubacteria	Eucephalobus	**-6.726**	**-0.855**	**13.486**	**5.299**
Eubacteria	Panagrolaimus	**-6.726**	**-0.945**	**13.486**	**4.539**
Eubacteria	Plectus	**-6.726**	**-0.583**	**13.486**	**4.414**
Eubacteria	Prismatolaimus	**-6.726**	**-1.280**	**13.486**	**4.238**
Eubacteria	Rhabditidae	**-6.726**	**-0.692**	**13.486**	**5.723**
Eubacteria	Enchytraeus	**-6.726**	**1.118**	**13.486**	**4.345**
Eubacteria	Henlea	**-6.726**	**1.701**	**13.486**	**3.700**
Eubacteria	Marionina	**-6.726**	**0.887**	**13.486**	**4.151**
Henlea	Arctoseius	**1.701**	**-0.152**	**3.700**	**2.453**
Henlea	Cheiroseius	**1.701**	**0.356**	**3.700**	**2.277**
Henlea	Dendrolaelaps	**1.701**	**0.027**	**3.700**	**3.438**
Henlea	Hypoaspis	**1.701**	**0.334**	**3.700**	**2.930**
Henlea	Parasitus	**1.701**	**0.859**	**3.700**	**3.017**
Henlea	Pergamasus	**1.701**	**1.081**	**3.700**	**2.453**
Henlea	Rhodacarellus	**1.701**	**-0.310**	**3.700**	**1.976**
Henlea	Aporcelaimellus	**1.701**	**0.548**	**3.700**	**4.238**
Henlea	Dorylaimoidea	**1.701**	**-0.604**	**3.700**	**4.636**
Henlea	Mesostigmata	**1.701**	**-0.411**	**3.700**	**2.578**

Henlea	Scutacarus	1.701	-0.608	3.700	3.374
Henlea	Stigmaeidae	1.701	0.229	3.700	2.675
Marionina	Arctoseius	0.887	-0.152	4.151	2.453
Marionina	Cheiroseius	0.887	0.356	4.151	2.277
Marionina	Dendrolaelaps	0.887	0.027	4.151	3.438
Marionina	Hypoaspis	0.887	0.334	4.151	2.930
Marionina	Parasitus	0.887	0.859	4.151	3.017
Marionina	Pergamasus	0.887	1.081	4.151	2.453
Marionina	Rhodacarellus	0.887	-0.310	4.151	1.976
Marionina	Aporcelaimellus	0.887	0.548	4.151	4.238
Marionina	Dorylaimoidea	0.887	-0.604	4.151	4.636
Marionina	Mesostigmata	0.887	-0.411	4.151	2.578
Marionina	Scutacarus	0.887	-0.608	4.151	3.374
Marionina	Stigmaeidae	0.887	0.229	4.151	2.675
Hyphae and hair roots	Aglenchus	6.671	-1.053	0.000	4.238
Hyphae and hair roots	Dolichodoridae	6.671	-0.885	0.000	4.238
Hyphae and hair roots	Helicotylenchus	6.671	-0.792	0.000	3.937
Hyphae and hair roots	Hoplolaimidae	6.671	-1.090	0.000	3.937
Hyphae and hair roots	Malenchus	6.671	-1.330	0.000	4.891
Hyphae and hair roots	Paratrichodorus	6.671	-0.630	0.000	3.937
Hyphae and hair roots	Paratylenchus	6.671	-1.244	0.000	4.539
Hyphae and hair roots	Pratylenchus	6.671	-1.226	0.000	4.636
Hyphae and hair roots	Tylenchorhynchus	6.671	-0.664	0.000	3.937
Hyphae and hair roots	Tydeidae	6.671	-0.608	0.000	1.976
Hyphae and hair roots	Sminthuridae	6.671	-0.608	0.000	2.675
Hyphae and hair roots	Sminthurinus	6.671	0.618	0.000	3.481
Hyphae and hair roots	Sminthurus	6.671	1.429	0.000	3.055
Hyphae and hair roots	Sphaeridia	6.671	0.202	0.000	1.976
Hyphae and hair roots	Achromadora	6.671	-1.396	0.000	3.937
Hyphae and hair roots	Aphelenchoides	6.671	-1.496	0.000	3.937
Hyphae and hair roots	Tylenchidae	6.671	-1.360	0.000	4.238
Hyphae and hair roots	Oppiella	6.671	-0.447	0.000	2.675
Hyphae and hair roots	Pygmephorus	6.671	-0.376	0.000	2.578
Hyphae and hair roots	Tyrophagus	6.671	0.005	0.000	1.976
Hyphae and hair roots	Folsomia	6.671	0.900	0.000	2.277
Hyphae and hair roots	Friesea	6.671	0.434	0.000	1.976
Hyphae and hair roots	Hypogastrura	6.671	0.977	0.000	2.453
Hyphae and hair roots	Isotoma	6.671	1.898	0.000	3.657
Hyphae and hair roots	Isotomurus	6.671	1.787	0.000	2.930
Hyphae and hair roots	Lepidocyrtus	6.671	1.231	0.000	2.675
Hyphae and hair roots	Mesaphorura	6.671	0.618	0.000	1.976
Hyphae and hair roots	Proisotoma	6.671	0.770	0.000	1.976
Hyphae and hair roots	Achaeta	6.671	1.096	0.000	3.462
Hyphae and hair roots	Fridericia	6.671	1.568	0.000	4.211
Hyphae and hair roots	Aporcelaimellus	6.671	0.548	0.000	4.238
Hyphae and hair roots	Dorylaimoidea	6.671	-0.604	0.000	4.636
Hyphae and hair roots	Mesostigmata	6.671	-0.411	0.000	2.578
Hyphae and hair roots	Scutacarus	6.671	-0.608	0.000	3.374
Hyphae and hair roots	Stigmaeidae	6.671	0.229	0.000	2.675
Alliphis	Arctoseius	0.053	-0.152	2.879	2.453
Alliphis	Cheiroseius	0.053	0.356	2.879	2.277

Alliphis	Dendrolaelaps	**0.053**	**0.027**	**2.879**	**3.438**
Alliphis	Hypoaspis	**0.053**	**0.334**	**2.879**	**2.930**
Alliphis	Parasitus	**0.053**	**0.859**	**2.879**	**3.017**
Alliphis	Pergamasus	**0.053**	**1.081**	**2.879**	**2.453**
Alliphis	Rhodacarellus	**0.053**	**-0.310**	**2.879**	**1.976**
Alliphis	Aporcelaimellus	**0.053**	**0.548**	**2.879**	**4.238**
Alliphis	Dorylaimoidea	**0.053**	**-0.604**	**2.879**	**4.636**
Alliphis	Mesostigmata	**0.053**	**-0.411**	**2.879**	**2.578**
Alliphis	Scutacarus	**0.053**	**-0.608**	**2.879**	**3.374**
Alliphis	Stigmaeidae	**0.053**	**0.229**	**2.879**	**2.675**
Arctoseius	Aporcelaimellus	**-0.152**	**0.548**	**2.453**	**4.238**
Arctoseius	Dorylaimoidea	**-0.152**	**-0.604**	**2.453**	**4.636**
Arctoseius	Mesostigmata	**-0.152**	**-0.411**	**2.453**	**2.578**
Arctoseius	Scutacarus	**-0.152**	**-0.608**	**2.453**	**3.374**
Arctoseius	Stigmaeidae	**-0.152**	**0.229**	**2.453**	**2.675**
Cheiroseius	Aporcelaimellus	**0.356**	**0.548**	**2.277**	**4.238**
Cheiroseius	Dorylaimoidea	**0.356**	**-0.604**	**2.277**	**4.636**
Cheiroseius	Mesostigmata	**0.356**	**-0.411**	**2.277**	**2.578**
Cheiroseius	Scutacarus	**0.356**	**-0.608**	**2.277**	**3.374**
Cheiroseius	Stigmaeidae	**0.356**	**0.229**	**2.277**	**2.675**
Dendrolaelaps	Aporcelaimellus	**0.027**	**0.548**	**3.438**	**4.238**
Dendrolaelaps	Dorylaimoidea	**0.027**	**-0.604**	**3.438**	**4.636**
Dendrolaelaps	Mesostigmata	**0.027**	**-0.411**	**3.438**	**2.578**
Dendrolaelaps	Scutacarus	**0.027**	**-0.608**	**3.438**	**3.374**
Dendrolaelaps	Stigmaeidae	**0.027**	**0.229**	**3.438**	**2.675**
Hypoaspis	Aporcelaimellus	**0.334**	**0.548**	**2.930**	**4.238**
Hypoaspis	Dorylaimoidea	**0.334**	**-0.604**	**2.930**	**4.636**
Hypoaspis	Mesostigmata	**0.334**	**-0.411**	**2.930**	**2.578**
Hypoaspis	Scutacarus	**0.334**	**-0.608**	**2.930**	**3.374**
Hypoaspis	Stigmaeidae	**0.334**	**0.229**	**2.930**	**2.675**
Parasitus	Aporcelaimellus	**0.859**	**0.548**	**3.017**	**4.238**
Parasitus	Dorylaimoidea	**0.859**	**-0.604**	**3.017**	**4.636**
Parasitus	Mesostigmata	**0.859**	**-0.411**	**3.017**	**2.578**
Parasitus	Scutacarus	**0.859**	**-0.608**	**3.017**	**3.374**
Parasitus	Stigmaeidae	**0.859**	**0.229**	**3.017**	**2.675**
Pergamasus	Aporcelaimellus	**1.081**	**0.548**	**2.453**	**4.238**
Pergamasus	Dorylaimoidea	**1.081**	**-0.604**	**2.453**	**4.636**
Pergamasus	Mesostigmata	**1.081**	**-0.411**	**2.453**	**2.578**
Pergamasus	Scutacarus	**1.081**	**-0.608**	**2.453**	**3.374**
Pergamasus	Stigmaeidae	**1.081**	**0.229**	**2.453**	**2.675**
Rhodacarellus	Aporcelaimellus	**-0.310**	**0.548**	**1.976**	**4.238**
Rhodacarellus	Dorylaimoidea	**-0.310**	**-0.604**	**1.976**	**4.636**
Rhodacarellus	Mesostigmata	**-0.310**	**-0.411**	**1.976**	**2.578**
Rhodacarellus	Scutacarus	**-0.310**	**-0.608**	**1.976**	**3.374**
Rhodacarellus	Stigmaeidae	**-0.310**	**0.229**	**1.976**	**2.675**
Aporcelaimellus	Alliphis	**0.548**	**0.053**	**4.238**	**2.879**
Aporcelaimellus	Arctoseius	**0.548**	**-0.152**	**4.238**	**2.453**
Aporcelaimellus	Cheiroseius	**0.548**	**0.356**	**4.238**	**2.277**
Aporcelaimellus	Dendrolaelaps	**0.548**	**0.027**	**4.238**	**3.438**
Aporcelaimellus	Hypoaspis	**0.548**	**0.334**	**4.238**	**2.930**
Aporcelaimellus	Parasitus	**0.548**	**0.859**	**4.238**	**3.017**

Aporcelaimellus	Pergamasus	**0.548**	**1.081**	**4.238**	**2.453**
Aporcelaimellus	Rhodacarellus	**0.548**	**-0.310**	**4.238**	**1.976**
Aporcelaimellus	Aporcelaimellus	**0.548**	**0.548**	**4.238**	**4.238**
Aporcelaimellus	Dorylaimoidea	**0.548**	**-0.604**	**4.238**	**4.636**
Aporcelaimellus	Mesostigmata	**0.548**	**-0.411**	**4.238**	**2.578**
Aporcelaimellus	Scutacarus	**0.548**	**-0.608**	**4.238**	**3.374**
Aporcelaimellus	Stigmaeidae	**0.548**	**0.229**	**4.238**	**2.675**
Dorylaimoidea	Alliphis	**-0.604**	**0.053**	**4.636**	**2.879**
Dorylaimoidea	Arctoseius	**-0.604**	**-0.152**	**4.636**	**2.453**
Dorylaimoidea	Cheiroseius	**-0.604**	**0.356**	**4.636**	**2.277**
Dorylaimoidea	Dendrolaelaps	**-0.604**	**0.027**	**4.636**	**3.438**
Dorylaimoidea	Hypoaspis	**-0.604**	**0.334**	**4.636**	**2.930**
Dorylaimoidea	Parasitus	**-0.604**	**0.859**	**4.636**	**3.017**
Dorylaimoidea	Pergamasus	**-0.604**	**1.081**	**4.636**	**2.453**
Dorylaimoidea	Rhodacarellus	**-0.604**	**-0.310**	**4.636**	**1.976**
Dorylaimoidea	Aporcelaimellus	**-0.604**	**0.548**	**4.636**	**4.238**
Dorylaimoidea	Dorylaimoidea	**-0.604**	**-0.604**	**4.636**	**4.636**
Dorylaimoidea	Mesostigmata	**-0.604**	**-0.411**	**4.636**	**2.578**
Dorylaimoidea	Scutacarus	**-0.604**	**-0.608**	**4.636**	**3.374**
Dorylaimoidea	Stigmaeidae	**-0.604**	**0.229**	**4.636**	**2.675**
Mesostigmata	Arctoseius	**-0.411**	**-0.152**	**2.578**	**2.453**
Mesostigmata	Cheiroseius	**-0.411**	**0.356**	**2.578**	**2.277**
Mesostigmata	Dendrolaelaps	**-0.411**	**0.027**	**2.578**	**3.438**
Mesostigmata	Hypoaspis	**-0.411**	**0.334**	**2.578**	**2.930**
Mesostigmata	Parasitus	**-0.411**	**0.859**	**2.578**	**3.017**
Mesostigmata	Pergamasus	**-0.411**	**1.081**	**2.578**	**2.453**
Mesostigmata	Rhodacarellus	**-0.411**	**-0.310**	**2.578**	**1.976**
Mesostigmata	Aporcelaimellus	**-0.411**	**0.548**	**2.578**	**4.238**
Mesostigmata	Dorylaimoidea	**-0.411**	**-0.604**	**2.578**	**4.636**
Mesostigmata	Mesostigmata	**-0.411**	**-0.411**	**2.578**	**2.578**
Mesostigmata	Scutacarus	**-0.411**	**-0.608**	**2.578**	**3.374**
Mesostigmata	Stigmaeidae	**-0.411**	**0.229**	**2.578**	**2.675**
Scutacarus	Arctoseius	**-0.608**	**-0.152**	**3.374**	**2.453**
Scutacarus	Cheiroseius	**-0.608**	**0.356**	**3.374**	**2.277**
Scutacarus	Dendrolaelaps	**-0.608**	**0.027**	**3.374**	**3.438**
Scutacarus	Hypoaspis	**-0.608**	**0.334**	**3.374**	**2.930**
Scutacarus	Parasitus	**-0.608**	**0.859**	**3.374**	**3.017**
Scutacarus	Pergamasus	**-0.608**	**1.081**	**3.374**	**2.453**
Scutacarus	Rhodacarellus	**-0.608**	**-0.310**	**3.374**	**1.976**
Scutacarus	Aporcelaimellus	**-0.608**	**0.548**	**3.374**	**4.238**
Scutacarus	Dorylaimoidea	**-0.608**	**-0.604**	**3.374**	**4.636**
Scutacarus	Mesostigmata	**-0.608**	**-0.411**	**3.374**	**2.578**
Scutacarus	Scutacarus	**-0.608**	**-0.608**	**3.374**	**3.374**
Scutacarus	Stigmaeidae	**-0.608**	**0.229**	**3.374**	**2.675**
Stigmaeidae	Arctoseius	**0.229**	**-0.152**	**2.675**	**2.453**
Stigmaeidae	Cheiroseius	**0.229**	**0.356**	**2.675**	**2.277**
Stigmaeidae	Dendrolaelaps	**0.229**	**0.027**	**2.675**	**3.438**
Stigmaeidae	Hypoaspis	**0.229**	**0.334**	**2.675**	**2.930**
Stigmaeidae	Parasitus	**0.229**	**0.859**	**2.675**	**3.017**
Stigmaeidae	Pergamasus	**0.229**	**1.081**	**2.675**	**2.453**
Stigmaeidae	Rhodacarellus	**0.229**	**-0.310**	**2.675**	**1.976**

Stigmaeidae	Aporcelaimellus	**0.229**	**0.548**	**2.675**	**4.238**
Stigmaeidae	Dorylaimoidea	**0.229**	**-0.604**	**2.675**	**4.636**
Stigmaeidae	Mesostigmata	**0.229**	**-0.411**	**2.675**	**2.578**
Stigmaeidae	Scutacarus	**0.229**	**-0.608**	**2.675**	**3.374**
Stigmaeidae	Stigmaeidae	**0.229**	**0.229**	**2.675**	**2.675**
